# Supplementary material for: Whole genome sequences of Treponema pallidum subsp. endemicum isolated from Cuban patients: The non-clonal character of isolates suggests a persistent human infection rather than a single outbreak
Source: PLoS Negl Trop Dis. 2022 Jun 10;16(6):e0009900. doi: 10.1371/journal.pntd.0009900 (PMC9223347; doi:10.1371/journal.pntd.0009900)
Supplement: S1 Text — (DOCX) [file pntd.0009900.s002.docx]

**S1 Text. Sequence of C75**

>C75 [organism=Treponema pallidum subspecies endemicum][strain=C75][topology=circular][location=chromosome][technique=NGS]

nnnnnnnnnnnnnnnnnnnnnnnnnnnnnnnnnnnnnnnnnnnnnnnnnnnnnnnnnnnnnnnnnnnnnnnnnnnnnnnnnnnnnnnnnnnnnnnnnnnnnnnnnnnnnnnnnnnnnnnnnnnnnnnnnnnnnnnnnnnnnnnnnnnnnnnnnnnnnnnnnnnnnnnnnnnnnnnnnnnnnnnnnnnnnnnnnnnnnnnnnnnnnnnnnnnnnnnnnnnnnnnnnnnnnnnnnnnnnnnnnnnnnnnnnnnnnnnnnnnnnnnnnntggaaatactgctccccccaaacacgtgcatacctacctggagaaaaactctcctgcagaggttccttccaaaaagagctttcaccccgacctgaacagagannnnnnnnnnnnnnnnnnnnnnnnnnnnnnnnnnnnnnnnnnnnnnnnnnnnnnnnnnnnnnnnnnnnnnnnnnnnnnnnnnacttcctacaatccgttacttatctacggtggagtgggactaggaaaaacccaccttatgcaggctattggacacgagatctacaagacaacagacctgaacgtcatatacgtcactgcggagannnnnnnnnnnnnnnnnnnnnnnnnnnnnnnnnnnnnnnnnnnnnnnnnnnnnnnnnnnnnnnnnnnnnnnnnnnnnnnnnnnnnnnnnnnnnnnnnnnnnnnnnnnnnnnnnnnnnnnnnnnnnnnnnnnnnnnnnnnnnnnnnnnnnnnnnnnnnnnnnnnnnnnnnnnnnnnnnnnnnnnnnnnnnnnnnnnnnnnnnnnnnnnnnnnnnnnnnnnnnctctcttctcGCTTACGCTCGAGGTGCTCCCGAGGGCTTAGCACTGATCTGAATATGCCATGTTTTGAAACGCGCTGTGCTATCTtgattaaaaaaatacaaaactataacagcacctatcctcacaaagccatCCACATTTCAGACGATGTTGTCCGACTTGTTTCTGAAAACATTTCTTCAAATATCAGGGATCTTGAGGGGGCATTAaCnAAAATTATCGCTTTCATTGAAGTGTCGGGATCCATCACGATAGATATCGTTCCCTCTCTCCTAAAAGAGTTCTTCCTCTCTGCAAGGCCAAAACACATCACAGTAGAAACTATTCTTCATGTAGTTGCAGATCACTTTAACATTTCGTATTCAGATCTAAAGGGTAAGAAACGCAATAAAAGCGTTGTTTATCCTCGGCAAATCGCTATGTTTCTCTCAAAGnnnnnnnnnnnnnnnnnnnnnnnnnnnnnnnnnnnnnnnnnnnnnnnnnnnnnnnnnnnnnnnnnnnnnnnnnnnnnnnnnnnnnnnnnnnnnnnnnnnnnnnnnnnnnnnnnnnnnnnnnnnnnnnnnnnnnnnnnnnnnnnnnnnnnnnnnnnnnnnnnnnnnnnnnnnnnnnnnnnnnnnnnnnnnnnnnnnnnnnnnnnnnnnnnnnnnnnnnnnnnnnnnnnnnnnnnnnnnnnnnnnnnnnnnnnnnnnnnnnnnnnnnnnnnnnnnnnnnnnnnnnnnnnnnnnnnnnnnnnnnnnnnnnnnnnnnnnnnnnnnnnnnnnnnnnnnnnnnnnnnnnnnnnnnnnnnnnnnnnnnnnnnnnnnnnnnnnnnnnnnnnnnnnnnnnnnnnnnnnnnnnnnnnnnnnnnnnnnnnnnnnnnnnnnnnnnnnnnnnnnnnnnnnnnnnnnnnnnnnnnnnnnnnnnnnnnnnnnnnnnnnnnnnnnnnnnnnnnnnnnnnnnnnnnnnnnnnnnnnnnnnnnnnnnnnnnnnnnnnnnnnnnnnnnnnnnnnnnnnnnnnnnnnnnnnnnnnnnnnnnnnnnnnnnnnnnnnnnnnnnnnnnnnnnnnnnnnnnnnnnnnnnnnnnnnnnnnnnnnnnnnnnnnnnnnnnnnnnnnnnnnnnnnnnnnnnnnnnnnnnnnnnnnnnnnnnnnnnnnnnnnnnnnnnnnnnnnnnnnnnnnnnnnnnnnnnnnnnnnnnnnnnnnnnnnnnnnnnnnnnnnnnnnnnnnnnnnnnnnnnnnnnnnnnnnnnnnnnnnnnnnnnnnnnnnnnnnnnnnnnnnnnnnnnnnnnnnnnnnnnnnnnnnnnnnnnnnnnnnnnnnnnnnnnnnnnnnnnnnnnnnnnnnnnnnnnnnnnnnnnnnnnnnnnnnnnnnnnnnnnnnnnnnnnnnnnnnnnnnnnnnnnnnnnnnnnnnnnnnngagaatcctctccccaatccttttcgggtgttattgtgccaactaaGATCTTAGGCATAGTAAACCGTAAGCTTACCCCTGAAGGATCAGTGACGCTATGCATTACGTCGCAGnnnnnnnnnnnnnnnnnnnnnGGATATAAGTTTTCTTCTGTGCTTATTGAGGGGCAATTTCCTAATTACAAAAGAGTAATCCCTGATCATCAGGAGCGTTCTTTTTGTGTTGGACGTGTGGAGCTAATGGAGGCACTTAAACGAGTCTCGTTGTTGGTAGAACAAAAATCTCACAGGATATTTATTACCATACAGCAGGGTTTGTTGACTTTAAGCTCAAAAGCTCACACTCAAGAAAATGAAATAGGTGATGCTCAGGAAGAAATAGCCTGTGCTTATACAGGAGAAAGTGAGGTCATAGCTCTTAACTATCTATACCTTGAAGAACCGCTTAAGGTTTTTACTTCGAAGGAGGTTCAAGTGGAATTTACCGATCCTGCAAAAGCACTCACGCTTCGTGCTGTACnAAACACGGACTGCTTTCACATCATTATGCCTATGCAAACGGAGTGATTCTTTGCCTTTTCTCACAGTGACTGCAATAAATTTCAGAAATCTTGCACatcacacgattgatatatctnnnnnnnnnnnnnnnnnnnnnnnnnnnnnnnnnnnnnnnnnnnnnnnnnnnnnnnnnnnnnnnnnnnnnnnnnnnnnnnnnnnnnnnnnnnnnnnnnnnnnnnnnnnnnnnnnnnnnnnnnnnnnnnnnnnnnnnnnnnnnnnnnnnnnnnnnnnnnnnnnnnnnnnnnnnnnnnnnnnnnnnnnnnnnnnnnnnnnnnnnnnnnnnnnnnnnnnnnnnnnnnnnnnnnnnnnnnnnnnnnnnnnnnnnnnnnnnnnnnnnnnnnnnnnnnnnnnnnnnnnnnnnnnnnnnnnnnnnnnnnnnnnnnnnnnnnnnnnnnnnnnnnnnnnnnnnnnnnnnnnnnnnnnnnnnnnnnnnnnnnnnnnnnnnnnnnnnnnnnnnnnnnnnnnnnnnnnnnnnnnnnnnnnnnnnnnnnnnnnnnnnnnnnnnnnnnnnnnnnnnnnnnnnnnnnnnnnnnnnnnnnnnnnnnnnattgcaaccgtgggttttgatctcgtgcagtggaGAACTCAGCTTGTCCGTGACTTTAACGTGATTTTTACTAAGTATTATGAGcgccttggagaccttgcgcaggtgcgcattgagtataagccTTCATGGTCTGACTCCTCAGTTGAGGagatcgtacattctctttacaagagacgtaagcannnncttgcgatggggatgagtatgtcaggtcCTCATAGAGATAAGATTCACTTTACTCGGTCGCAGGCGCTTTTCATTCCTCAGGCTTCTACCGGACAGAGGCGGTTGGTTTCGTTGGTACTGAGGATGTCGCAGGCTGTGTTCTACACAGGAGTAACGGGAAAACTGCCCGTACTCTTAATGGATGATGTCTTGTTAGAGCTTGATCCTGAGAAGCGGGAAAGGTTCATGATGAGTTTGCCTCCGTATGATCAGCTGTTTTGTACATTTTTGCCAGGGGAAGCGTACAGGcgatACGGGCGTgaaaaaacgcgggtatattttgtttctgaaggggcgtgtcatgaataatggnnnnnnnnnnnnnnnnnnnnnnnnnnnnnnnnnnnnnnnnnnnatccaagcttcctatgaaacggaggcgtttgatgcgcatcgagaatgggnnnnnnnnnnnnnnnnnnnnnTTGCGTTACACAGCACGCTGGTAGATATCAGAAATGGGAAAGTTGTGGTCAAGGTGACTCATCCTGGTTGGGCACAATACCTTTTGTTAAAGAAAGACGAAATTGTACATGCCCTTCGTAGGCGATATCCGTCGTTGGGAGTGACGGGTATGAGTAcnnnnnnnnnnnnnnnnnnnnnnnnnnnnnnnnnnnnnnnnnnnnnnnnnnnnnnnnnnnnnnnnnnnnnnnnnnnnnnnnnnnnnnnnnnnnnnnnnnAGGTATTTGAACAGCTCCGAACGCTTTTTCAGGTGAAAACGGAAGAACCGTCACATTAGTTTTGCGGATGGGATTCGACGGATCTGTTCAAAGTCCATAGGACTGCGGTTTTTCTTGCGTGCAGCCTATGCACGACTGTGTnnnnccttgaacgcagtatggctttgcgttagaatgcccgccctatggaagaaattagcaccccagagggtggcgttcttgtgcccaTTTCTATAGAGACAGAAGTCAAGCGTGCTTACATAGACTATTCTATGTCCGTCATAGTTTCTCGTGCGCTTCCGGATGTCCGCGACGGTTTAAAGCCTGTTCACAGACGTATTCTCTACGCGATGGAGGAAAAAGGGCTACGCTTTTCAGGACctacacggaagtgtgccaagataGTGGGGGACGTTTTGGGAAGctttcatcctcatggggatgcgtccgtctatgacgcgctagtgcgtcttgggcaagatTTTTCCCttcgttatccagtCATTCATCCTCAAGGAAATTTCGGGACTATCGGGGGCGACCCTCCGGCAGCGTatcggtacaccgaagcgaagatggcgcgtattgcagaatctatggtagagGACATAAAAAAGGAAACGGTTTCCTTTGTTCCCAATTTTGACGATTCTGACGTAGAGCCCACGGTTCTTCCTGGAAggtttccttttcttcttgngaatgggtccagtggtattgcagttggtatgactacaaacatgccaccgcataattnnnnnnnnnnnnnnnnnnnnnnnnnnnnnnnnnnnnnnnnnnnnnnnnnnnnnnnnnnnnnnnnnnnnnnnnnnnnnnnnnnnnnnnnnnnnnnnnnnnnnnnnnnnnnnnnnnnnnnnnnnnnnnnnnnnnnnnnnnnnnnnnnnnnnnnnnnnaaaattgttgtccgtgctcgcnttaccatcgagacggattcaaagggtagggataccattatttttacagaagttccgtatcaagttaatactaccatgcttgTTATGCGTATTGGGGAacttgcacgtgcgaaagtgatcgaaggtattgnnnnnnnnnnnnnnnnnnnnnnnnnnnnnnnnnnnnnnnnnnnnnnnnnnnnnnnnnnnnnnnnnnnnnnnnnnnnnnnnnnnnnnnnnnnnnngtttgcaaannnnnnnnnnnnnnnnnnnnnnnnnnnnnnnnnnnnnnnnnnnnnnnngggaagaCCTCGAATGCTCACGCTCAAGGACCTAGTGCGCTACTTTGTAGAACACCGGGTCGATGTAGTGACTCGGCGTGCGCATTTTGAATTACGTAAGGCTCAGGAGCGCATACACTTGGTGCGTGCGCTGATACGTGCCttggatgccnnnnnnnnnnnnnnnnnnnnTATCCGTCATTCGCAGAACACAGAGCTTGCAAAACAGCGTTTGCGTGAACAATTTGACTTTGACAAnnnnnnnnnnnnnnnnnnnnnnnnnnnnnnnnngaagcgcttgacaggtttggaAGTCGAGAGTTTGCGTACGGAATTGAAagatttgacggagctgatttcttctctggaggagttacttacttctccccaaaaggtctTGGGAGTTGTTAAGAAAGAGACGCGTGATATCGCAGATATGTTTggggatgatcggcgtacagatnnnnnnnnnnnnnnnnnnnnnnnnnnnnnnnnnnnnnnnnnnnnnnnnnnnnnnnnnnnnnnnnnnnnnnnnnnnnnnnnnnnnnnnnnnnnnnnnnnnnnnnnnnnnnnnnnnnnnnnnnnnnnnnnnnnnnnnnnnnnnnnnnnnnnnnnnnnnnnnnnnnnnnnnnnnnnnnnnnnnnnnnnnnnnnnnnnnnnnnnnnnnnnnnnnnnnnnnnnnnnnnnnnnnnnnnnnnnnnnnnnnnnnnnnnnnnnnnnnnnnnnnnnnnnnnnnnnnnnnnnnnnnnnnnnnnnnnntattaagtcgcttCTCATGGTAGCGACGGACGAGGAGATCACGGCCATCGTATCTTTGAGAGAGTTTAGTAATAAAAGTTATGTTTTTATGGCTACTGCGCGAGGTGTAGTTAAAAAGGTAACTACTGATAATTTTGTGAATGCGAAGACGCGCGGTATTATAGCGCTTAAGCTGAGCGGAGGTGACACGCTGGTGAGCGCAGTGTTGGTGCAGGACGAAGATGAAGTAATGCTTATTACGCGTCAGGGAAAAGCATTGCGCATGTCGGGGAGGGAGGTGCGCGAAATGGGTCGCAATTCCAGTGGGGTGATTGGGATAAAATTGACGTCCGAGGACCTAGTGGCGGGGGTTTTGCGAGTAAGCGAACAACGGAAAGTACTGATAATGACGGAGAATGGATATGGTAAGCGGGTCAGTTTTTCAGAATTTTCTGTACatgggcgagggactgcaggacagaagatttacacacaaacggatagaaaaggtgctataatnnnnnnnnnnnnnnnnnnnnnnnnnnnnnagtgtatgtgtattactggtcagggaaaaacgattcgcgtggacgtgtgtgCaatcagcGTGCTGGGGCGTGGTGCGCAGGGCGTGCGTGTGTTGGATATCGAGCCATCGGATTTAGTAGTAGGACTTAGTTGTGTAATGCAGGGGTAATGGGCTCTGGGGTATATTTCTCCGTGAGTGGCTGTGTATATGTTGTGAGTATTGTGGATAATGTGCGTGCAGAAGTTGATGTTTCACGTGAAACGTGTGGGATGAGGAGTGGGATCAAATCTACCCTAATTCTGGAGGATTATTTGGGTTCACGTTCATGTAAACTTTATGGGGGTTGTGTATGGGGACTCGTGTCAGATTTTCCTTCTGCGGTATTGCGGGTGTATGTTTACTCGCACnnnnnnnnnnnnnnnnnnnnnnnnnnnnnnnnnnnnnnnnnnnnnnnnnnnnnnnnnnnnnnnnnnnnnCTTCTTATCGGATCGGTCTCATGACAAGTACGGGATCTCAGTCTGTAGATGATGTCCTTGCGAAGACACGCCTCGTCAGtatctacggagaggctcgtggggaaacgggtggaaGGATTGTCCATGTTACTTACTCCGATAACTTCTCCCACGACCATGAAGCAACCGTTTCTAAGTTGCTTGCACTCGCTGAGGATTCGACTATAAAGGCCATTGTGGTTAGTCAGGCAGTTCCCGGCGTTTCAAAGGCGTTTGGGATCATTAAGTCTAAACGTCCTGATGTTTTGCTTTTTGCGGGAGAACCACTTGAGCCGGTAGAGATGCTGCAGGAGTCTGCAGACAtcgtggtcagtcagGACTACTTGttcgGTGGATATGCCGTTCCGTGGGTTGCGGAAAGGATGGGGGnnnnnnnnnnnnnnnnnnnnnnnnnnnnnnnnnnnnnnnnnnnnnnnnnnnnnnnnnnnnnnnnnnnnnnnnnnnnnnnnncatgtaccgatttgggacnnnnnnnnnnnnnnnnnnnnnnnnnnnnnnnnnnnnnnnnnnnnnnnnnnnnnnnnnnnnnnnnnnnnnnnnnnnnnnnnnnnnnnnnnnnnnnnnnnnnnnnnnnnnnnnnnnnnnnnnnctactgcacnnnnnnnnnnnnnnnnnnnnnnnnnnnnnnnnnnnnnnnnnnnnnnnnnnnnnnnnnnnnnnnnnnnnnnnnnnnnnnnnnnnnnnnnnnnnnnnnnnnnnnnnnnnnnnnnnnnnnnnnnnnnnnnnnnnnnnnnnnnnnnnnnnnnnnnnnnnnnnnnnnnnnnnnnnnnnnnnnnnnnnnnnnnnnnnnnnnnnnnnnnnnnnnnnnnnnnnnnnnnnnnnnnnnnnnnnnnnnnnnnnnnnnnnnnnnnnnnnnnnnnnnnnnnnnnnnnnnnnnnnnnnnnnnnnnnnnnnnnnnnnnnnnnnnnnnnnnnnnnnnnnnnnnnnnnnnnnnnnnnnnnnnnnnnagactgaaaacgcacgaaattgttccgaatcacgtgctggtgtatacggacacatacgtcctgggTAAATTTACCTTGCCCGTCACAGACCAAGTACTCCcagaagggtattgggcattgaccgctaaggaataagaactccgttcgggttnnnnnnnnnnnnnnnnnnnnnnnnnnnnnnnnnnnnnnnnnnnnnnnnnnnnnnnnnnnnnnnnnnnnnnnnnnnnnnnnnnnnnnnnnnnnnnnnnnnnnnnnnnnnnngccagctccagttctcttctatcaacatgtagggatcctgtgaaagcaacccttgctcccaccgcacggaaaactccacaggtttgataggacttgcacgcagctcaacagcgtattggaaacaaagttctccctttaaannnnnnnnnnnnnnnnnnnnnnnnnnnnnnnnnnnnnnnnnnnnnnnnnnnnnnnnnnnnnnnnnnnnnnnnnnnnnnnnnnnnnnnnnnnnnnnnnnnnnnnnnnnnnnnnnnnnnnnnnnnnnnnnnnnnnnnnnnnnnnnnnnnnctgttgctatcgcgtctgccggtaaccctgccgcgcgtgctacaaagtttgtggcactattcaagaaattaagaagacctaaaatacctactctggCAGCATTGGCGACCTGCTGTGCTACCCCTTGGGCAGGTACGACGTCTGGGGGGAGACCTCCGTTGTCAAGATAACTTTTGTAGTCCAGGGGAAGGTATACACGnnnnnnnnnnnnnnnnnnnnnnnnnnnnnnnnnnnnnnnnnnnnnnnCTCCCGAACGAGTTTCTAGTCTGAGAAACGCAGCAAAGTCCGTGTATTGAaaagttgactttacaaagggaccactcccaaaaacnnnnnnnnnnnnnnnnnnnnnnnnnnnnnnnnnnnnnnnnnnnnnnnnnnnnnnnnnnnnnnnnnnnnnnnnnnnnnnnnnnnnnnnnnnnnnnnnnatccagctgaatatacgacGCACCGTCCGTAATGACGGATACGTCTTCTCCGCGAGTTCTAGTGCCTGTTCGACGACACGTGCTCTCGCACTGTTCGTATCCCGGTAGGTATTTCCGGCATCCGTAGCTAAAATGAAGCGGAATCCTGCACCAGGCATGATCGAAAGACGccctCCCACACTCCATAAGagnnnnnnnnnnnnnnnnnnnnnnnnnnnnnnnnnnnnnnnnnnnnnnnnnnnnnnnnnnnnnnnnnnnnnnnnnnnnnnnnnnnnnnnnnnnnnnnnnnnnnnnnnnnnnnnnnnnnnnnnnnnnnnnnnnnnnnnnnnnnnnnnnnnnnnnnnnnnnnnnnnnnnnnnnnnnnnnnnnnnnnnnnnnnnnnnnnnnnnnnnnnnnnnnnnnnnnnnnnnnnnnnnnnnnnnnnnnnnnnnnnnnnnnnnnnnnnnnnnnnnnnnnnnnnnnnnnnnnnnnnnnnnnnnnnnnnnnnnnnnnnnnnnnnnnnnnnnnnnnnnnnnnnnnnnnnnnnnnnnnnnnnnnnnnnnnnnnnnnnnnnnnnnnnnnnnnnnnnnnnnnnnnnnnnnnnnnnnnnnnCCGTAGAAGCGAAGGGTCGCTTCTACGGAGAAGCTTTGTTTTTCTGTGGGCGTGAGTTCGCCTAAAGCCTCACCAGATAAGGAACCGAGGGGGagcgcacactctaggttcnnnnnnnnnnnnnnnncatacacaccgctgcttttatgcagattcgaccgagttttcggtggacaaagcgtcagcgagaaaaagagagTACTGGAACtcctaaaaccgtgtctgcgatgacggaagcgatgattggggttctctnnnnnnnnnnnnnnnnnnnnnnnnnnnnnnnnnnnnnnnnnnnnnnnnnnnnnnnnnnnnnnnnnnnnnnnnnnnnnnnnnnnnnnnnnnnnnnnnnnnnnnnnnnnnnnnnnnnnnnnnnnnnGCGATACGGTAGCGCGCCCTCGGAGTAAAAGTATAAAAATGCACGAGCCTAACACTAGACGCATGCATACAGTAGTGTACCGTGTTTTCACCACAGTTTAGTACAACGATGtcatGTGTCAGATCTAGCAGTATCTGTAATGTATGTTGGTGTACATTAGATATTCGTGGGTGGGAAGAAGAGTCACTTTCTGGGGAGGCGTATAGAAGGAACGGGGCGTGGTGTTGTGCCAtttgtgcgaaaactnnnnnnnnnnnnnnnnnnnnnnnnnnnnnnnnnnnnnnnnnnnnnnnnnnnnnnnnnnnnnnnnnnnnnnnnnnnnnnnnnnnnnnnnnnnnnnnnnnnnnnnnnnnnnnnnnnnnnnnnnnnnnnnnnnnnnnnnnnnnnnnnnnnnnnnnnnnnnnnnnnnnnnnnnnnnnnnnnnnnnnnnnnnnnnnnnnnnnnnnnnnnnnnnnnnnnnnnnnnnnnnnnnnnnnnnnnnnnnnnnnnnnnnnnnnnnnnnnnnnnnnnnnnnnnnnnnnnnnnnnnnnnnnnnnnnnnnnnnnnnnnnnnnnnnnnnnnnnnnnnnnnnnnnnnnnnnnnnnnnnnnnnnnnnntttcaggtgaggatcccatatgggttgagctttCTCTCAAGGGATTGAAGGTGGATTTTGAAAGTGCTTTAGGGTCGGGAACTGCGGATCCAAGTATGACGACGCGTTCTCCTTTCTTAAAGTCnnnnnnnnnnnnnnnnnnnnnnnnnnnnnnnnnnnncctctacgatgtctctttttcngtnnnnnnnnnnnnnnnnnnnnnnnnnnnnnnnnnnnnnnnnnnnnnnnnnnnnnnnnnnnnnnnnnnnnnnnnnnnnnnnnnnnnnnnnnnnnnnnnnnttggtggggtngggggaaaaatcgcatatnnnnnnnnnnnnnnnnnnnnnnnnnnnnnnnnnnnnnnnnnnnnnnnnnnnnnnnnnnnnnnnnnnnnnnnnnnnnnnnnnnnnnnnnnnnnnnnnnnnnnnnnnnnnnnnnnnnnnAATAGTCGGCGCATGCCAGCGGACCCGCACAGTAAATATGGCCTTGGTACTGAGTTCACGCTCGTATACGCCCGAAAGGGGCGGGAACAGGTCaggcttgaggcagcaagttgtgctactctttctgcagggtacagaactggacctgatcnnnnnnnnnnnnnnnnnnnnnnnnnnnnnnnnnnnnnnnnnnnnnnnnnnnnnnnnnnnnnnnnnnnnnnnnnnnnnnnnnnnnnnnnnnnnnnnnnnnnnnnnnnACCGTACAAGAAGGGTGCCGCGAGGGAGTCCCTCGCTGAAACGCTTGCGGCACAGCGTGGTTGTAATCGTTTTGACACCGCGCTCATGCACGCGCTTGGGTTACTTGTTGCTGCTGCGAAGACACGCAATGAACTCGCCGCACAGATGCGAnnnnnnnnnccnccaggtgtgtgggaaaaatttgaacagGCGGTGCAATCGTTACCTCCTATAACGCAGGGAAAGCCTGGCGTCGTTGGGGCGGAGGTCCGCCCGGGTACGATGTGGATGGAACTTTCCCCGGTAAGGAAAGCACTTGTCGATGTACTTTCTGTACTTGAGCAGGGTGGTTTTGATCGTGTCGCCTTTGACGCATTGCTGATTGTGCAATGGCGCTGGATTTCGCTGGGAGCATACGTAGCAAGTGCTCCTACCAATGTGTTTGGCTCAATGCTTTTTCCGCGTGGGAGTAGTGACCATTTTGActgtgccgcattcgtgcgggtggaaagtaagtggtacgATTCTCTTTCTAAGCTTGTGTCCGGTCnnnnnnnnnnnnnnnnnnnGCGTTTATATATTCCTTTCACTCATGGCTTGTACCTGGAACCTGGCTCCTGTACGCCGAATTCCACACGGGGAAAGAAGCCTCAGGCGTTTGTGCTTCCTCCACCCGGTGTTGTCCATCCGGGTGCACACAGCGCGTTGCCTGTTGTGGGGAAACTGTGGCTTAACTACCGGATTACTCTTGCTGCTCACGCATGGATAAGACCGATGGTTTCGCTGTATGGCGCTACATATGGTGCGCAGGGTTTTTCTTACGGACCAGGTGGTGCTGCCGGAACGGTAAAGAGGAACCGCACGTTCCGCGCAGGTAAAAATCTGTATTATCAGGTAGGGGTGGCGGTGAGTCCGTTTGAAAGATGTGAGTTGCTCATTGAGTGGTCGCAGGGAATGCTTGCGCGTCGTCCCTACATAAGTTTGGAGCATGGttcnnnnnnnnnnnnnnnnnnnnnnnnnnnnnnnnnnnnnnnnnnnnnnnnnnnnnnnnnnnnnnnnnnnnnnnnnnnnnnnnnnnncctttttggtttgcacacgatgattttttgatgctgtatcataaatnnnnnnnnnnnnnnnnnnnnnnnnnnnnnnnnnnnnnnnnnnnnnnnnnnnnnnnnnnnnnnnnnnnnnnnnnnnnnnnnnnnnnnnnnnnnnnnnnnnnnnnnnnnnnnnnnnnnnnnnnnnnnnnnnnnnnnnnnnGTTGTGTCGCTGTGGGGGCGCGCGTGCTCCACAAAATACCGGGGGCACGAAAGCGGTGGAGGATTATCACATCGGGGTTATGACCGGTCCGGGTAGTCACTCTAGCCATGATGTAGTTGCGGCAAAAAGnnnnnnnnnnnnnnnnnnnnnnnnnnnnnnnnnnnnnnnnnnnnnnnnnnngcatgtcacgtatcctgaagattttgtnnnnnnnnnnnnnnnnnnnnnnnnnnnnnnnnnnnnnnnnnnnnnnnnnnnnnnnnnnnnnGCTATTGTTGTCAGCGAAGGAATTCATGGAACTGCAGAGGCTTTTAACAAAATTAGGGCGAAGCGTTCCGACATCATGCTCTTTGTAGGGGATCCGCATGAGTATCCTGAGCTTATCCAAAAATCTGCTGATATCGTGnnnnnnnnnnnnnnnnnnnnnnnnnnnnnnnnnnnnnnnnnnnnnnnnnnnnnnnnnnnnnnnnnnnnnnnnnnnnnnnnnnnnnnnnnnnnnnnnnnnnnnnnnnnnnnnnnnnnnnnnnnnnnnnnnnnnnnnnnnnnnnnnnnnnnnnnnnTCGGTATAGAGTTTGCGTCTGAGGAGGCTCCAGATCCCGCAGGGGAGTCAGGTGTTGAAGGGGCGCGTGCGTTTATCGGCACACACACGAAGCAATGGATCGGTAAGTATGGGAAGAACACTATGTTCTTTTGCACTAATGATACGCACCGTGTTTCCCTGATGAGAGAACTGGTAAGCAAAGATGGCATGCTTCTGGGGGCGAATGTGTTTGACtgtgcngaagcactcggggtggagtatgctgacgatgaggatnnnnnnnnnatcctggagcgcgtcgagtcggcggtagaagagaagaggttggtaggtcgtttcggggtgaatgtaagttcgcatatttttgtcagcaCACTTGGGTTAACTGAATATGCGCGCAGGATATTGCnnnnnnnnnnnnnnnnnnnnnnnnnnnnnnnnnnnnnnnnnnnnnnnnnnnnnnnnnnnnnnnnnnnnnnnnnnnnnnnnnnnnnnnnnnnnnnnnnnnnnnnnnnnnnnnnnnnnnnnnnnnnnnntgtgtcagtgttctctgacatacatattttgggtaagttttctttaccggtaacagaccaggaatttccggaaaagtatcggagnnnnnnnnnnnnnnnnnnnnnnnnnnnnnnnnnnnnnnnnnnnnnnnnnnnnnnnnnnnnnnnnnnnnnnnnnnnnnnnnnnnnnnnnnnnnnnnnnnnnnnnnnnnnnnnnnnnnnnnnnnnnnnnnnnnnnnnnnnnnnnnnnnnnnnnnnnnnnnnnnnnnnnnnnnnnnnnnnnnnnnnnnnnnnnnnnnnnnnnnnnnnnnnnnnnnnnnnnnnnnnnnnnnnnnnnnnnnnnnntgtnttttgagctgctcggngaacggtgcgacgatgatacgttggagcgacgtttgcgaagtgctaaggcnnnnnnnnnnnnnnnnnnnnnnnnnnngagggaaGATAAAGAGGGAGGGAGCCTTTCGCCAGGAGGGGAGGTGTGCGATCCTGTGCAGAGGACGAGGGGTCAGGCCAGCTGTGTACGTGAGGGTGCGTTTGATGCGCGTCTTTTAAAAATCGAACTTAACGACACAAATCGTCCGGATTTGTGGTctacagcagggcnnnnnnnCCTCTTGCGTGTTCACGCAGGGGGGGCTTCGCGCGCAGCGCAGTATCGTGCATTTCTTTCAGATCGCTGTACGGTGCGCGACTCGTATGGGCGATGCGTAGTAGTGGATGCGCGTTTGCAAACTATACGCCCTTTTATTGCTGGCTTTGTGGCTCGGGGTACTGGACTGAGCGAAGTAAGACTGTGGGATCTAATACAAACACAGGAAAGACTCGCCAGTAATTTTGGACGTAGGAGGTGTACCCTTTCGCTGGGGTGTTACCGTGCACAGGATATATGTTGGCCACTCGCATACCGCGCTGTCCTACTCGCAGAGGTAAGTTTCACGCCTCTCGGTATGTCCATGCCGCnGTCTGGTGAGCAGATTCTGACGCAACATCCGAAAGGGCGTGAATATGGTCACCTCCTCAAGGATTATTCTTTCGTTCCCTTATTAGTCGATGCTCGTGGTGAGGTGCTTTCGCTTATTCCGATCACTAATAGCGCGTCGCTGGGTGCAGTAGTGACCGGGGATACAGAACTATTTATCGAGTGTAGTGGTACGGATATGTGCGCAGTGCTAGTAGCGGTGAATAGCCTTGCGTGTGATCTTTCTGATATGGGTATGCAGATTGAACCTGTGCAAATTACCTATAGTTTTGACACCCCATGGGGGAGGTCTGTTACTACCCCCTTTTATTTTCAAGAAAAAAGGGAGGTTGCGCACGAGCAGATAGATCGTCTTTTGGGGATGCCCTTGCCTGTTGCAGATATAACCGAAGCCTTTGCAAGGATGGACTGTGCCGTGCAAGTTAAGCAGGGAACCTATGTCGTCGAGCCTGCAGCGTACCGTAATGATTTTCTCCATGCGGTAGATCTGATTGAAGAAGTGATGCTGGGTAGAACGCTTGACCGTTTTTCTCCGCAGGTTCCATGTTCTTTTACAGTGGGCAGACTGAGTGATCTGACCCTTTTGACGCGCAAAATTAAGCACCTTCTAGTTGGATTTGGTTATCAGGAGATGATTTTTCATTATCTTGGCTCTGCCAGGGAGTTTTGCACGCGTATGCGATGCACGGCAGATGATTTAATCGAGATAGAAAATCCTCTTACTGAAAGCTACCGATTCGTGCGCAGGTCTATCATTCCGTGTCTTCTGTCAGCTGAGCTAAAGTCAGCACATGCGTTGTATCCACATCGTATTTTCGAAATTGnnnnnnnnnnnnnnnnnnnnnnnnnnnnnnnnnnnnnnnnnnnnnnnnnnnnnnnnnnnnnnnnnnnnnnnnnnnnnnnnnnnnnnnnnnnnnATGAGGTGGCGAGCCTAGTTTCAGGACTTTTGTATTGTTTGAAACTTCCGTATCAGGTAGAGGnnnnnnnnnnnnnnnnnnnngtcttaggcagacaagcgagtatnnnnnnnnnnnnnnnnnnnnnnnnnnnnnnnnnnnnnnnnnnnnnnnnnnnnnnnnnnaattgggatatccgcatgccgtgctttgctggggaacttgatgttggggccctattgccgtagctcgctcnnnnnnnnnnnnnnnnnnnnnnnnnnnnnnnnnnnnnnnnnnnnnnnnnnnnnnnnnnnnnnnnnnnnnnnnnnnnnnnnnnnnnnnnnnnnnnnnnnnnnngaacgtgcctgcgctgcgatacgtctTGGGATGAGAATGTACGGTGACGGCTACAATATCTGTGTGATTGGCGCGTCAGGCACcgggaagcgcactacgctccaatatctattaaaagatTTTGTACCTnnnnnnnnnnnnnnnnnnnnnnnAGCGTATGCATACAATTTTGTGCATCCGCACGAACCGCAGGTGCTGCAGTTTCCGGCGGGAGAAGGGATGCCTTTTGCTACGGCGTTGAGGAGATCGGTTAACGCTATTCTCAATACnnnnnnnnnnnnnnnnnnnnnnnnnnnCTTCTTACGTGAGCGGCGCACATTGCTGGCTGATATTGAAACACGTGAGnnnnnnnnnnnnnnnnnnnnnnnnnnnnnnnnnnnnnnnnnnnnnnnnnnnnnnnnnnnnnnnnnnnnnnnnnnnnnnnnnnnnnnnnnnnnnnnnnnnnnnnnnnnnnnnnnnnnnnnnnnnnnnnnnnnnnnnnacgatttagcttcccgcgcgaagctttctagatgtgtagtacacgaactccatgcgcgatatcgtctttcctgtgatgaggtttcttcgctgctccatacgttgcgcactgcGCGGCGGGCCgcgcgtaggcgtcttgcgcaGTACTACCGTGCGCGTTTGCGGTTAATTATCCTCGAGCAGATGGCCTGTATTAAAAAACGCGTGGCGTGTTATGCACCGGTTTTTTCCCCTCCACCTGTTTCGTGTGCGGCGGAACnnnnnnnnnnnnnnnnnnnnnnnnnnnnnnnnnnnnnnnnnnnnnnnnnnnnnnnnnnnnnnnnnnnnnnnnnnnnnatatcgTACATAGAGCGGATACAAACCGATGTGCAGGTGCGGGTACAGTGTTTAATGTCAATGCGTATTTCTGCACTAGTAAAAAAGCGGTTTTTCGATCGCTATACCTTAAATTTCGTGtgtgtgcacacagaacatgcggggtatgtgtnnnnnnnnnnnnnnnnnnnnnnnnnnnnnnnnnnnnnnnnnnnnnnnnnnnnnnnnnnnnnnnnnnnnnnnnnnnnnnnnnnnnnnnnnnnnnnnnnnncaggtgcgctgcatcgagcgcatgcaggtgtactcatcgtgcAGCTCGAAGATCTACTGGCGGAGGAAGAAGCATGGACACATTTGAAGCGTGCGCTGCGTACCAAGCAGGTGTTACTCCCAGCGGGTAGTTCCCAGTCCCAAGGTATGTTgcggcctgaaggagttccgctcacatgcaagctTGTTTTGnnnnnnnnnnnnnnnnnnnnnnnncgtctatctcaggaagattcctcgttccgAGAACTATTTAAGGTGTGTGCAGAATTTGATACGAGCATGCCGAATTCTGATAAGAATCAAGTTGCTTTGATAGCCTACCTTGATAGAGTTGTGGCGCGTTATGGCACCTTCTCACTTGATTCCTCAGCGTACGCGCGATTGTTAGCATATGCGGAAGAACTGGCAGAGAGTCATACACGGCTCAGTACTTCTTTTGTACAAATTGCAGATTTAGTATTGGAGTCTCATGCAGTGGCCGTGGATATGCATCCAGATGTGTCGGTGATTACTGCGCATGTGGTGCAGGAAGCGCTGAATCGCAGGCAGTACGTGTGTTCCCGCGCGCGGGAGCGTTTTCGGCGCATGATAGCCTGTGGAGAGTTATTGGTAGAGGTACAGGGATATCGAATAGGGAGAATTAACGCGCTGGCAATCGAAGAACattgcggccatagctttggcatagtaatttcgttaacggcacaggcatccgcgGGTAAGGAAGGAGTGATGAATATTGAGCGGGAGGCAGGTCTTTCGGGGGAAATTTATGATAAGGCGCATTTAATCATCACTTCGCTATTGCGTGAGAAGTGCTTGTCTGCTTTTGCAGATGCTGCATTTGATCCAGCTGTGGATGACCTTGGAAAGGGGCAGGATAGTTTTCCACTGTGTCTTTCTGCAGCGCTTTGTTTTGaacagtcgtatcacnnnnnnnnnnnnnnnnnnnnnnnnnnnnnnnnnnnnnnnnnnnnnnnnnnnnnnnnnnnCCGGTTTcctcttcggcaggaccgcgcaATAACAGGAAGTGTGAACCAGTTGGGACAAGTGCAAGCTATTGGAGGAGTGAGCGAGAAGATCAGTGGCTTTTATGACGTCTGTGCTCTGAACGGACTGACGGGAATGCAGGGAGTTTTGATCCCAAAGAGTAGTTGTGCACAATTGTTTTTGCCnnAACGTGTGCAGnnnnnnnnnnnnnnnnnnacgtttcacgtgtgggcggtatgcacaattgatgatgcgctggaattgatggtgccggacgATTTGAAAGAGCAGGGAAGTGCTCAGCTCTACCAGTGTGTGCGTGCGCGTTTGCGGGATTTTTATGTCACAGTGAAAGGACACTGCGAGTAGCTCGTGTTTATTTACATAGCGTTGGTTTTGTTTTTGAAAAAGTACTTTCGTTGACGTGGCGCTTTGATACCGATACAATATCCCGCTATGGTTGCGCgatcgattcgtgtagagcttgagtcagcactGGATTTACTGAAGCAGGGggatctgcgccaggcacaccaggatttgcaggatttgCTTCAAAAAGAGCTAGACaatcgggatgttttgtacgcgcttaccggtattcgcttttgggaggatcggatgcagcgtgtacatactatctctggcnnnnnnnnnnnnnnnnnnnnnnnnnnnnnnnnnnnnnnnnnnnnnnnnnnnnnnnnnnnnnnnnnnnnnnnnnnnnnnnnnnnnnnnnnnnnnnnnttgcgctgttgtgtgttcacttacgcattgcggttttatgggagtcTTATCACCGGTGATGCGCGCGAGCATCCCGCGGAAGTCTATCGTAAGACTGGGCTGTGCTATAAAGCATTGGGCAATTACGATGCTGCGCGTGAATGCCTTGAGTTTGCAGTCTCTTTGGAGCCAGACTCTTCTGCAATTCTTGCTGAACTTGCTGATGCATACGCTCTTGCGGGAGATGAGCGCTGGTCGAAGTTGTATTTTCGCGAGGCCTTGTTCaaggnnnnnnnnnnnnnnnnnnnnnnnnnnnnnnnnnnnnnnnnnnnnnnnnnnnnnnnnnnnnnnnnnnnnnnnnnnnnnnnnnnnnnnnnnnnnnnnnnnnnnnnnnnnnnnnnnnnnnnnnnnnnnnnnnnnnnnnnnnnnnnnnnnnnnnnnnnnnnnnnnnnnnnnnnnnnnnnnnnnnnnnnnnnnnnnnnnnnnnnnnnnnnnnnnnnnnnnnnnnnnnnnnnnnnnnnnnnnnnnnnnnnnnnnnnnnnnnnnnnnnnnnnnnnnnnnnnnnnnnnnnnnnnnnnnnnnnnnnnnnnnnnnnnnnnnnnnnnnnnnnnnnnnnnnnnnnnnnnnnnnnnnnnnnnnnnnnnnnnnnnnnnnnnnnnnnnnnnnnnnnnnnnnnnnnnnnnnnnnGGGTGAAGTAATGTCAGAACACATAGAACACGACGTTCGGGAAATGCTCAATGAAGAAAAATGGACACGCGCGACGCTTACCGCGTATTCtgcggaaaagtttaAGGAACTTGACAGAATCATTGCGGAGGCGAAAAGACAATCTATCCTTGATGTACTGAAAGGTATCTGTGACGAACATCTGGCGCACTCGAAGAACAGTATAATCGCGTTATACATTTCTGGGATTATTTCGCTTTCTAAGCAGTTGTTAGATGATTCGTGTTTAGTGACGCTGCTGACTATCTTTGGTGATAATCACAAGAATCAAATAGTTGAGCACCTCTGTACCCGTGTGCTTGAGTACggtgaatcaaagcttgcgttgcgtgcgttaggagaATGTTACAAnnnnnnnnnnnnnnnnnnnnnnnnnnnnnnnnnnnnnnngttagttaggatcgattacgaagaggcggaaatcactcgtgtgctggcggataaatacgagcaggaagggaataaagagaaagctacggagttttacaaaaaannnnnnnnnnnnnnnnnnnnnnnnnnnnnnnnnnnnnnnnnnnnnnnnnnnnnnnnnnnnnnnnnnnnnnnnnnnnnnnnnnnnnnnnnnnnnnnnnnnnnnnnnnnnnnnnnnnnnnnnnnnnnnnnnnnnnnnnnnnnnnnnnnnnnnnnnnnnnnnnnnnnnnnnnnnnnnnnnnnnnnnnnnnnnnnnnnnnnnnnnnnnnnnnnnnnnnnnnnnnATATTCTTGAACATGATGAGAAGGATGTTTGGGCGCGTAAGGAAATCATAGAGAATTTTCGGTGTAAGTATCGCGGACATAGCcagcttgaggagtacctaaagatatcgaacattagccaatcttggcgcnnnnnnnnnnnnnnnnnnnnnnnnnnnnnnnnnnnnnnnnnnnnnnnnnnnnnnnnnnnnnnnnnnnnnnnnnnnnnnnnnnnnnnnnnnnnngcgaaggtGTGTAACGATGAGTTACTGATCGATTTTGCGAAAAGGCGTGCGCATACCATGCTTTTGAAGATGGCTATTAGCGCGTTGCAAACCCTTGGCAAAGAGCATATCnGGGTGCTTAAGTCGGTACTGAAGCGGCAGGATCTTGCTGCGAAAATAaagcaggatcctgaatgggcactgaaggtgatcatnnnnnnnnnnnnnnnnnnnnnnnnnnnnnnnnnnnnnAAGCAGGAATTAGTTCcttctttgctttctgtgggggagtggacgagttggagtacgaaagcacggaagattttgaaagaaagtactggatttgctgcgaatcccagcaatatcgatttttatacggtgcggagcnnnnnnnnnnnnnnnnnnnnnnnnnnnnnnnnnnnnnnnnnnnnnnnnnnnnnnnnnnnnnnnnnnnnnnnnnnnnnnnnnnnnnnnnnnnnnnnnnnnnnnnnnnnnnnnnnnnnnnnnnnnnnnnnnnnnnnnnnnnnnnnnnnnnnnnnnnnnnnnnnnnnnnnnnnnnnnnnnnnnnnnnnnnnnnnnnnnnnnnnnnnnnnnnnnnnnnnnnnnnnnnnnnnnnnnnnnnnnnnnnnnnnnnnnnnnnnnnnnnnnnnnnnnnnnnnnnnnnnnnnnnnnnnnnnnnnnnnnnacacagagataaaggataagggcctcaagtgggaattcgtgaatagtGTGAAAAACTTTGTGAGCAATTGGAGCGATGAGTATGTCnnnnnnnnnnnnnnnnnnnnnnnnnnnnnnnnnnnnnnnnnnnnnnnnnnngagGGATATAAGGAAAAGGCGCTGAGGGTGGTCGAGGCTTGCTTTGAATACTATGCGGATAATCGTGCGGCGGTTATTTGGTTATTCAAGACGGTAAGGGATGAGCCCTGGTTCCAGGAGCTGCGCATTACCGCAGAACAGCGGATTATCGTCCTCATCCACATTGTGGACATTACTTATCGGGAAATCGCTAACCGGCGGAACACCACTGAGAACCGAAAACTTAACAAGCAGGCTCTTTCGGTACTCTTTGGGAATGATCATTTGCTAGAACnnnnnnnnnnnnnnnnnnnnnnnnnnnnnnnnnnnnnnnnnnnnnnnnnnnnnnnnnnnnnnnnnnnnnnnnnnnnnnnnnnnnnnnnnnnnnnnnnnnnnnnnnnnnnnnnnnnnnnnnnnnnnnnnnnnnnnnnnnnnnnnnnnnnnnnnnnnnnnnnnnnnnnnnnnnnnnnnnnnnnnnnnnnnnnnnnnnnnnnnnnnnnnnnnnnnnnnnnnnnnnnnnnnnnnnnnnnnnnnnnnnnnnnnnnnnnnnnnnnnnnnnnnnnnnnnnnnnnnnnnnnnnnnnnnnnnnnnnnnnnnnnnnnnnnnnnnnnnnnnnnnnnnnnnnnnnnnnnnnnnnnnnnnnnnnnnnnnnnnnnnnnnnnnnnnnnnnnnnnnnnnnnnnnnnnnnnnnnnnnnnnnnnnnnnnnnnnnnnnnnnnnnnnngtaattagcttaaaaaatcacacaagtggagaagatgagacatacactattcttggtccgtgggagtcggctccagaacgtggtattatttcgtacatgtctccgttagnnnnnnnnnnnnnnnnnnnnnnnnnnnnnnnnnnnnnnnnnnnnnnnnnnnnnnnnnnnnnnnnnnnnnnnnnnnnnnnnnnnnnnnnnnnnnnnnnnnnnnnnnnnnnnnnnnnnnnnnnnnnnnnnnnnnnnnnnnnnnnnnnnnnnnnnnnnnnnnnnnnnnnnnnnnnnnnnnnnnnnnnnnnnnnnnnnnnnnnnnnnnnnnnnnnnnnnnnnnnnnnnnnnnnnnnnnnnnnnnnnnnnnnnnnnnnnnnnnGTACCGTTCCGTGGTAAGCGGTAGTGTGCTAAAAGATATCGCTACTCGTTTTGTGCGTTTGGGTGATTCGTTCCATATTATTTCGTTTAGTGCCACGccacgtcacgagatttctcaggttatccgtagtgagtttgatctttctcagGTnnnnnnnnnnnnnnnnnnnnnnnnnnnnnnnnnnnnnnnnnnnnnnnnnnnnnnnnnnnnnnnnnnnnnnnnnnnnnnnnnnnnnnnnnnnnnnnnnnnnnnnnnnnnnattttgATTGTTGTGTCTGACGGTATTTTTAACCCGCctgcgcgtaGCGCATATAAAAACTACAACAAGGATCAGGTAAAAATTAACCTTGCACGGGCTGCCGCGGATCTGAGACGAGAGCAGGTGCGTGTGTTTTACATAAAACTTCCCTTTCCCCAGGACATCCAGATCCGCGATTTGGATGnnnnnnnnnnnnnnnnnnnnnnnnnnnnnnnnnnnnnnnnnnnnnnnnnnnnnnnnnnnnnnnnnnnnnnnnnnnnnnnnnnnnnnnnnnnnnnnnctgtgggtgtggtttccgatcaaacgggcggcgttgcagataaccatgcagttgctacGCACGGAAGGGAGGACGGGACAGTCCAAGGGGTTGTTGGCAGCCATGTGGAGGTGGCACGCACACAGGACAGACGCAGTAATGCAGATCCTGCTAAAAGGGAAGGGGTTCGGCCTTCCTCAGAAGCAACTGATGTTTCCCGCGAGTTCACGGAGGATTTGGGAATCAGGGTGAGTCCGGTTGATTCAGATGGTTCTGTGCGTTTTTCCGAGAAGGAGCGCACGCTTCCCGTGTTACACTTTCCAAGGGTCCTTGAGGTACAGGGTAAGTAtgcagaatgtatGTTCGAGGTTGAAAATAGCACggatGCTCCCGTTTTGTTGCATTTGGAGCGGGTGATTTTTGACAATGGCGTTGAGACTGACATAGTTTCGGTGCAAACAGAGTCTTGTGCAGTAGCGTCCGGTGCACGCGCGATGTTGCGAnnnnnnnnnnnnnnnnnnnnnnnnnnnnnnnnnnnnnnnnnnnnnnnnnnnnnnnnnnnnnnnnnnnnnnnnnnnnnnnnnnnnnnnnnnnnnnnnnnnnnnnnnnnnnnnnnnnnnnnnnnnnnnnnnnnnnnnnnnnnnnnnnnnnnnnnnnnnnnnnnnnnnnnnnnnnnnnnnnnnnnnnnnnnnnnnnnnnnnnnnnnnnnnnnnnnnnnnnnnnnnnnnnnnnnnnnnnnnnnnnnnnnnnnnnnnnnnnnnnnnnnnnnnnnnnnnnnnnnnnnnnnnnnnnnnnnnnnngtggtgtgtaggtcaaagcgcgtgttgtctcgtaaGTCATGGCGCGGAAGTCCCCGTACAGAGAATGGGTGTCAGGGTCCTGGTTCGATGTCTGATTTTCGGGCGCATTCTGTTAAGGAACAAAGGCAGGATCAGGAGCGCGTGTATGCAGgcatggagagaattgtatctcagcgtaaaagcgatGTGCAGGATCGCCTCAGTGTATTGAATGCGGCAACTGCATTTGGGCGTGATCGAGTTTCATTTTCCCCCAGGGTAACGCGTGCGGAGCATGGATGTAGTCGGTCAGGAATGACTGAAATTTTTGTGTTTGATCAAACACGTGCGATTGGCAAGCGCAATATTCACGTAATGAAAGCAGGAACCCGTTTAGGGGTTGGCGGGCACAAGGGGGATGACTTCCTAATTTTTTTGGTGCCGTTTCCAAGGcggctagcnnnnnngtattttgacggtgaagtatatnnnnnnnnnnnnnnnnnnnnnnnnnnnnnnnnnnnnnnnnnnnnnnnnnnnnnncacgactgcgtcggcagagtggttacccttgtctctgacAGGGGGTATCATGTGCCCTTCACATTCCGCCAGTATGAGGATCCCGCTGTGAGATTGAACAATCTGCTCACCTCTATCGAATACGCTTGATCAAAGCGATAAAGGAACGAGTcgagaggtggattcgggactcgattgagcagatggaaagnnnnnnnnnnnnnnnnnnnnnnnnnnnnnnnnnnnnnnnnnnnnnnnnnnnnnnnnnnnnnnnnnnnnnnnnnnnnnnnnnnnnnnnnnnnnnnnnnnnnnnnnnnnnnnnnnnnnnnnnnnnnnnnnnnnnnnnnnnnnnnnnnnnnnnnnnnnnnnnnnnnnnnnnnnnnnnnnnnnnnnnnnnnnnnnnnnnnnnnnnnnnnnnnnnnnnnnnnnnnnnnnnnnnnnnnnnnnnnnnnnnnnnnnnnnnnnnnnnnnnnnnnnnnnnnnnnnnnnnnnnnnnnnnnnnnnnnnnnnnnnnnnnnnnnnnnnnnnnnnnnnnnnnnnnnnnnnnnnnnnnnnnnnnnnnnnnnnnnnnnnnnnnnnnnnnnnnnnnnnnnnnnnnnnnnnnnnnnnnnnnnnnnnnnnnnnnnnnnnnnnnnnnnnnnnnnnnnnnnnnnnnnnnnnnnnnnnnnnnnnnnnnnnnnnnnnnnnnnnnnnnnnnnnnnnnnnnnnnnnnnnnnnnnnnnnnnnnnnnnnnnnnnnnnnnnnnnnnnnnnnnnnnnnnnnnnnnnnnnnnnnnnnnnnnnnnnnnnnnnnnnnnnnnnnnnnnnnnnnnnnnnnnnnnnnnnnnnnnnnnnnnnnnnnnnnnnnnnnnnnnnnnnnnnnnnnnnnnnnnnnnnnnnnnnnnnnnnnnnnnnnnnnnnnnnnnnnnnnnnnnnnnnnnnnnnnnnnnnnnnnnnnnnnnnnnnnnnnnnnnnnnnnnnnnnnnnnnnnnnnnnnnnnnnnnnnnnnnnnnnnnnnnnnnnnnnnnnnnnnnnnnnnnnnnnnnnnnnnnnnnnnnnnnnnnnnnnnnnnnnnnnnnnnnnnnnnnnnnnnnnnnnnnnnnnnnnnnnnnnnnnnnnnnnnnnnnnnnnaatggagtgcatctttggtgcagttgcccgatacgcatttttttgatcttatgcgcctctatttgggtgtgcttAAGACTCCATTTCATAAACAGAGGCTTgttcaacaacttagtgccttcctgcaaagaaagtctattcagaacgctgtnnnnnnnnnnnnnnnnnnnnnnnnnnnnnnnnnnnnnnnnnnnnnnnnnnnnnnnnnnnnnnnnnnnnnnnnnnnnnnnnnnnnnnnnnnnnnnnnnnnnnnnnnnnnnnnnnnnnnnnnnnnnnnnnnnnnnnnnnnnnnnnnnnnnnnnnnnnnnnnnnnnnnnnnnnnnnnnnnnnnnnnnnnnnnnnnnnnnnnnnnnnnnnnnnngcgcagaatggtagggtgcggcaaacgccgtgttatggtatcaATCCTCTGTTGCAAAAAGCATTGAGTACGGTAGCTGGACTCAATCTTTTTCTCATTCCGCAAAAGCGGATGCGTCCATCCGCACAGTTATTGACAACAGATTTGATGCTGTGCGCATTGTATTCGTTTTTTACGCACGGGGAAAATTTATTAAAAGTCGATGGGACGTTTAGGAAAAAGGCATTTGTTATGTTCCAGGCATTGTTTCCTGTTGATCCGGATGTGGTGAGTGTGGCACTCCCTGCATATCTGCAGAGAGCAGGGGAGGAAAGGGGTACATCACGTCTTTTACAGGAAGGTCGGCGCGTCTTGGAACATCTGGGATTGATTGTCTGCGAATCAGCACAGGTGCATGTGCAAGATAAACGGTGGGCTTCTTTTTTCTCCTTAACTGCTCTGGAACGTGCGGTGTATTTGACAGTTGCCAGTACGGCTATTCTGCGCAAAGAGGTGCTCGTACAGCGAGCGCAGGCTTTGCGTACACTTCTCtgtgtgttgcaCCCAGATGcgcaatacgcacctgaagatctaacacgcgtgtatcgtatcttggtggaagagnnnnnnnnnnnnnnnnnnnnnnnnnnnnnnnnnnnnnnnnnnnnnnnnnnnnnnnnnnnnnnnnnnnnnnnnnnnnnnnnnnnnnnnnnnnnnnnnnnnnnnnnnnnnnnnnnnnnnnnnnnnnnnnnnnnnnnnnnnnnnnnnnnnnnnnnnnnnnnnnnnnnnnnnnGACTGTGCGCGTTGAATGAGGCTCTATTTAAAGGACAGTACACGCGTGGGCCAGGAATGGTCTTGTCAGCGACGGCAGAGTTAACCATTTTCCCCGATGGAGATATGCAAGGGGTTTTGCCAATTTTATCCTGTGCGCATGTCTGCTCACTACAAACAGTTGCCACGTTTGAGCTCAATAAAAAAAGCTGTACCACTGGCTTTGCGCGCGGATTAACAGTGCAGGCACTTGCACAGGCTTTAGAATGTAAAACAGGTGAGCAGGTGCCACAGAATATACTATCTTCTTTCCGGCAGTGGTATGCACagataaccgcgttgaccttaagacgcggctttgtcatgcaggttgattcatctcagcAAGCTTTTTTTGAATCnnnnnnnCCACTGCACCCGCTAGTGCGCACGCGTCTTGCAGAAGGAGTGTACTTTTTTGATGAATGCCAAGAGTGTATGTTGTATCaGGngctcgcgcgagcgcgtctgtcctacctgtgcgagcCAATTGAnACAGCCACCCCGTTATTCCGCCCTGGTGAGCAGGGTGCACGTGCGCTCCATGTGCCTTCCTTTTCTTTTCCAGTGCGGTCTGCTCGGGGAGTCTCCGAGGAATCAACGCGAGATTTTGCACATTTAGGTGCCTTTGTGTTGGAAACTCCGAACGTTTCGTGCACGCACAGTGCTGCAGATACTCCGTCTATTTCAGAACAGACCGGTGGGGTGGCTCACGTGCAGAGCGAAGAGGATGTAGATCCGTCCACGTCTGGTGCAACGGGTAAGTATTGGGACAAGGCACAATGGCGCAAGGTGCAACGGATGCGACGTGCTGTGCGGCTGCAGCGGCTCAAAGAGTTTGAGGCGCACCTGCAACAACTAAAATTGGACGCAACAGAGCAGACGGAGCTACGTGCCCGCTtgcaacgGGGGTTGATTCTGGATAGAATGCAACTTTCGTCCGAAACGATCCGCAGGGAGAGAACGGAAGCGAGCGGGGTTGATTTTTTAGGCAAGTATCGTCTTGCAGAGTGTGCGTTACGTTCTGGTGCTTTACTTGAGATTGAGACTAGTTCAGGGCAGTCAGTGCATAAGATAGTGGGTACGGTGTGCGCAATTGAAAAATGCGAAGAGGATGCGTTGCTTCACGTGTGTGTACACGCAGAACTTCCCCCTGAGCGAGTATCGATTGCGCGCGCGTCCAGGATAGTGCTACTGAAAAATTCTATTTTTTCTTGAGTCTGTTCTGAAGGGGATCCTTTTGTCTCTTGTAAAAAGGAATAGACGAGCGGGTAGGATATGAGTCGTAGGAAACAGGGACGAGAGTTATTCAACAGTCATGTGGGCGTGGTGTTGTCTTGTGTCGGTGCGGCAATGGGGCTTGCAAACGTGTGGTTGTTCCCTGGACGCCTGGTGGAATTTGGTGGTGTGACGTTTTTAATTCCGTATTTTATTTTTCTATTTGGTCTTTCCCGTTTTGGACTGATGGGGGAGTATGCTTTTGGAAAGACACTGCGCTGCGGTCCTGTGCGTGCGTTTACCCGTGTGTGTGAAACACATCCATCGTGTTTTTTACGAGCACTACGAGGTAGCGGGTGGTTTCCGGTAGGAGTATTGCTCGCTACCTGCTCTTTTTATGTAGTGATTATAGGGTGGATCTTGCGTTATGTAGTATTTTCGTGCACGAATGCACTTGCAGGTACTCAGGCGCACGACCTGTTTTACCAGGTTGCAGGGACAAGTGCGAATGTGCCGTGGACGCTTGCAGCTATCGCGCTCACAGCGTGTGTAGTGAGTGCGGGCGTGCAAAAGGGGGTGGAGCGAGGAAACATTATAATGATGGTACTTTTTTACGGTGTCCTTGCGTTTATTACAGGATATATATTTACTCTTCCTAACGCGTGGATAGGTATGCGTAGAATGTTGGCATTTCAATCTTCATCATTGTGCAATCCGAGACTCTGGTTGTATGCATTAGGCATGTCGTTTTTTAGTCTCAGTTTGGGGGGCGCGGCTATGGTTTTATATGGCAGTTACATGCCAGATACGGTGGACATACCGCGTACTGCATTTCAGACAGCGACCTTAGATTTTTTGGCATCAGGTATGTCcgcattatGTTTAATTCCGAGTGCGTGGGTTTTAGGTATGGACGTCAGCAGTGGACCGGAGTTTTTGTTTGTAACAATAACCCGTGTCGCCTCGCAGATACCGATGGGGGTGATGATAAGTGTGTTATTCTTTTTGTGTGTACTATGTGCAGCGTTAAGTTCTGCAATTGCTATGTTAGAAGTAATACTCGAGTCTTTTGTGCACACGTGTACAGTGGGGCGCCGAACGCTGACGTGGTCACTAGCACTCGTGGTTGCGTTTGTATCTCTTCCTCTGAATGCCTCGATGAGAGTGTTCGAAACGTTTACAGATATAGTGGTGGTTATACTATCTCCGTTATCTGCCCTTATGGGGAGCGTGATGATATTTTGGGTATAtgGtgcagagcgttgccgtgtagctatcaaccgGTGTGCACGCGGTCCGTTGGGTAAATGGTTCACGCCGTATATGCGnnnnnnnnnnnnnnnnnnnnnnnnnnnnnnnnnnnnnnnnnnnnnnnnnnnnnnnnnnnnnnnnnnnnnnnnnnnnnnnnnnnnnnnnnnnnnnnnnnnnnnnnnnnnnnnnnnnnnnnnnnnnnnnnnnnnnnnnnnnnnnnnnnnnnnnnnnnnnnnnnnnnnnnnnnnnnnnngatatgttgacacgtCCGTACTCCTCAGTTTGTGAGGCTCCAGTTATAGGAGGGGGGATAGCTACGCGTGAAAAGATTTGCTCTTATTGGACTTGGAGACTTCGGTCTTAGCATGCTAAAGGAGCTGCTCAAGCTCACTAACAATATAGTCCTCCTGGACAGGGATCGAACGCTCGTTGAAACCTACCGTAGCAGGGTGAGAATCGTGCGCGCAATTGATGTGTTGGACGAATTCACTCTGTGCAAGATGATTCCACAGGATATCAACGCAGCGGTTATTGATCTGGGGGTTAAAATTGAATCATCAATCATGATAACAACGTTTTTAAAAAAATTAGAAATTGCAGATATCGTAGTTAAGGCATACAGCGCTGAACaagGGCATATCCTCTCGAGCGTTGGTGCTACGCACGTAGTGCTCCCGGACCGGGaggcaGCTAAAAAAGTCACTCCTATGATTGCTTTCGATCTTCTTTTCAACTTTATGCCACTTTCTGCGCAGCTGGCAATTGCGGAAATGGCTGTGCACGAGGACTATGTGGGAAGAACTTTGCGTGAAGTGGATGTGCGCAAAAACTTCTCTCTTAATATCATTGCTATCCGTAAGCGCGATGCAGAGGATTTTTGTTTTATCAATGATCCTGAATACTGCTTTGAAGCGAACGATGTGTTGCTCGTTGCCGGTTCTCACAAAGACATCTATGCACAGTCGCAGGACAAGCTGGCACATACCCATAGCTTCAGCGACTTTTTCAAACAATGGTTCCTTACCAGCTGACTTCCCAATGTTCCGCGCACGGGAGTAGGCGCGTGTAATCTTCCCTTTTCCCGCACATGCCTACGTAAAGGGGAATATTTAGAGAGGGGGCTCAGCTTCAAGTTTTGAAAAATAAGGCTCAAGCGTTGCCGCTTCCCGAATTGAGGTTGCAGTGCTTACCACCGCAGCTTCAGCACACGTGCGCTGCGCCCAGAGTAGTTGGGTACACAAGTGCATGTCTGTTACCcgtgcgtctagaatatgctcaatagccgcaatacgctctgtttctgtcgttccgttgagaaagcgcaggaaactcgcaAAACTTTTatgctccggtgtttcaggagttaccgccgtactgtacgcnnnnnnnnnnnnnnnnnnnnnnnnnnnnnnnnnnnnnnnnnnnnnnnnncccttgtgatcggcgtgcaggtgctcaatcgtCTTGAATATCACGTCAAGGGAGTggagcggatttggatcgcggtaagttagtgaggaaaatatgccatgtactggatctggcaGGGTGAATGCACCGTAAGCACCACCTATCGTTCGAATTTTTTCCCAAAACGGCTCnGTACTTAGATATcgggcaaacacctGCTCTACCCCGCGTCTCTCCAAAGGAAGCCGTGGATGTGCAAGGGACAGCGCTGCAAAACCCACTTGCACAGGGGCTGGAAGCAGCGTCAGCATATTGCGGGTGCGCATGTGCTGCAGGGCCTCTTGAAAGAGCACACCGTGAGCGGAGGGTATCTGTTGTTCGTGCGCTGTGGGCGCAGAGGTGTGGTGGATAAAATAAGTGGATAGCGGTGCAnnnnnnnnnnnnnnnnnnTTTGCCAATGCATCTAGCGCTGTGTGTAACGATGTTTCtgnaccacatacacatccgatcactcctgcagtgagcagtttttcatgcagcgctttgagtttngctgccagcgaaggggaggcaacggttTCTGTACACTCTGTCCACAAAGCACGTACCAAGCGAATCTGCGTGACTCCAGTCCAGAGTTCTTCTACGGCCTTTGCTGCATTCACGCGAGCGTTTGCCTTTGCAAGTGCAATGGAATGTCCTGAATGCATGGCAGCGCTATCCAAGTCATTTTTATATTGTGCAAGgannnnnnnnnnnnnnnnnnnnnnnnnnnnnnnnnnnnnnnnnnnnnnnnnnnnnnnnnnnnnnnnnnnnnnnnnnnnnnnnnnnnnnnnnnnnnnnnnnnnnnnnnnnnnnnnnnnnnnnnnnnnnatatcgctgcGTTGCAGTGTGTTCTGTCCCCTCAGTAAGGGGAGTATCTCGCTTCCCTGATCGCCTGCTACTATACAGCGGGCGGCAAAGCCCCCGGTGAGACGGGCAATCTCGGCAGATACCACACTCCAATGATGTGTCTCGGTTCCCATACCTGTCAGTGCGTAGCCATACAGTGGCAGAAGTTGTGCTTCTTTTACACTGAGCATATCTGCTGGGATTGCAAggtgtaagtacgtaATATCGTTCGTGGCAAGCTCATGCACGAGAACAGGAACAGAACCAAAAAACTGCATnnnnnnnnnnnnnnnnnnnnnnnnnnnnnnnnnnnnnnnnnnnnnnnnnnnggggagcaacgcaaggagttCCTCCGGATCGGGTGTTGTCTGTCGTACACGCAGTGATTCTTGGTCAGCTCGGAGGCGCGCCGCTGCTGGCTGCGTGAGTGTACGGGAGAAATCCTGTACGTATTTTTCTAATTGCTCATCAAGTTTTTTTGAGAAGTCTGGGTCTGGGTGTACCGAAAGTACCGTGTACTGCGGGTTGCGCAGCAAGTGCGTGAGGATGAGATTTTCCACGTAGTGTGGATGGTGGTGTACCTTTTCACGCAGTGCCTGCAGTGCGGGGATATAACGCAAAGAACTTTCTGGACCTGCACCGTGCAACCATCCACGCAGCGAACGCTGCATGAGCACGAGAGAAAAAGGACCGTCAGAGCGGCGTACTTCAGTATTtgaaaattngagtgcattcagcgctgtttccacttcCTGTGGAGGGATGCCGTGCGCAACAAGCGnnnnnnnnnnnnnnnnnnnnnnnnnnnnnnnnnnnnnnnnnnnnnnnnnnnnnnnnnnnnnnnnACCTACAAAAAAAAGCATACGCTTTAGATCGATGTGACTGCCGTTATATGCGTATAAATCCTCACCGAGTTCTGATTCTAACAGTGCCTGTGCAAGGGGAGCAGCATCGTGACCGAGCAAAACGTGTTCGAGCAAAAACACGTCCATTAACTGTTCAGCCTTGTCTGATTCTGGGAGTAACCAGCTGAGCAATacggcgnnnnGTGTTAAATCCATCCCCTCGCTCGCCGGTGCGTACCCGGTGTACGTACGGGGACTTTGGTATGCAGGGATAGGGGGGATGGGGGGCAACGCTTTGCGGGCAGAAAATTTTGAAAGGCATTTATCCTCAATAAATGCCATCTGtttttcggtnnnnnnnnnnnnnnnnnnnnnnnnnnnnnnnnnnnnnnnnnnnnnnnnnnnnnnnnnnnnnnnnnnnnnnnnnncgtacgtgagacgaggaataacngttggatgacctcctgaatcgtgtgcatacactgagccacnnnnnnnnnnnnnnnnnnnnnncttatacacaagcgtatgaaagtctGCATACACACCGCGCATTTCATTCAGTACAACGCCCTGGAGGGTAAGTTGGTTGTGCTCATTAAACTCAAAGCGGTGTCCTTCTTGCTTAAAGGTCCACTCTTCGATCAGGGGGAAAAAGACTGCGtctgcatatacactcataacattgannnnnnnnnnnnnnnnnnnnnnnnnnnnnnnnnnnnnnnnnnnnnnnnnnnnnnnnnnnnnnnnnnnnnnnnnnnnnnnnnnnnnnnnnnnnnnnnnnnnnnnnnnnnnnnnnnnnnnnnnnnnnnnnnnnnnnnnnnnnnnnnnnnnnnnnnnnnnnnnnnnnnnnnnnnnnnnnnnnnnnnnnnnnnnnnnaaaacagaaggcaaacaaattctccgggtcttcgttgagaatgtggtacaactcaagccctgtttttttgtgtcgagcaTAGACacccactgccgaaagcTCAGCGAGTGAATGGCGCCAGATAATTTCAAAACCGTGAAGAAGCGTACTCatcggtGATTCTCACTCCTCTTCTTGCAAGCTATTTGGAAGCAATGTGCTGTTGCGCGCCGGCACTGCGCAATGTAGCTaaaaagtgctCAGTGATGGTGCGCGTATCATGCGCTGAAGGCGGGAACGTGTCGTACTCATCGCGCACAAGCTGTGCGCGGTTTTTAGAGAGATTAGAGAGAATTTTCTGAACAAAAGCAGGATGATTGGTGTTGATGAGCGCAGCAAGAGTTTTTTCAGAacnnnnnnnnnnnnnnnnnnnnnnnnnngtatctnnnnnnnnnnnnatatcatccagcgtgaaaagatgcgtgcggacacgcgctgcaagtgttggatttttttctgcaagggcatgaagaattgaATGCTCAGTCGCGCGCTCCATCTTTTTGAGAATTGCCGCAAGCACTGCATGCCCGTCAAGATCACGGCGCTgggacaaatggagcgctgcaaactttttgtgcaaggagncactcatgacttgcagcacctgagggttaacgtGCTTTAACTTTGCAAGGCGAACGATCAAGTCCTTCTTCTCCTCTGTGCTGATATTACTCAAATAGTGCGCAGCGCTTTCTGGAGGCAGCTGCGAGAGGATGAGTGTTTTGGTGGCAGGTAGTTCTCCTTCCAGGAGGGGGAGAAGTTGGGAGGCTTCAAGCGCAGCCAAAAACTCAAAAGGTTTCGGCTGTGCCGCTGGCACCGCCCGCTTCAAGATAAGATCGGCCTTTTCTTCCCCAAACGCTTTGGAAAGCATCGACTGCGCAGCACGCAGTCCACCGGTAACAGGCGACACACGAGCGCAGAGGGCAGAAAACTCCCGTAGGATCTCACGCGCTTCTTCTGGACTGAGGGGTTTGAGTGTCAGGAGCTCGGCAACCACCGCCTCAATCTGTGCAGGCTCAAGTTGCTTGAGCACCAGCGCCGCCTGCTCTTCTCCAATGAGGGAGAGGAACTGGGCAATCTTTTTATAAACGGTTCGGCCTCGGTCTTGTTCACGTACGGTGGCTTTGATTAAGCCACGAGGAGATTCGGTTCTATTCATAGAAAGAGGACTCCGCGCGGTCTCCGGACACGCAGTTCATTGTAGTGGAGGGTGTGCTCTTGACACAAGGGCGTGAGCACCTTAAAAGGTGCCCTCCAGCGGGGAGGgGTCCAAGGATGTATGGCGTTGTCTCTTCGGTTCTGGTGTTTTAGCCCTTCGGGGGAATTACGTGTGAGTGTTCTTTCCGTTTCCGCTGCGTTCGGTGTGTTGGTAGTgctgctggtgcTTTCCATGTTGTTCTCTGCTGCGGAAACAGCGTTCTGTGCGCTAAATACCTTAAGACTTCGCTACCTGTACGAAAAACGTCATGCTCGCGCTCGAGTGGCAATGCGTATCCTTCGACGGAAAAACTTCTATCTTGCTGCTGTGGTTATCGGGAACACCCTGGCGAGCAGTGCGTTGTCTGCAGTCATTGCGCTTTTTGCACGTGCCCTCTTTGGCATCCACGCAGTGGGGTGGAGCATCGGTGCAGGAACGGTGCTTACACTTCTTTTTGGAGAAattattccgaagtcacttgccttgtgccggccgaacgcagtggcactgcatactgcgcgattcttgcagtGGAGCGCTTTGATGCTTACTCCTTTTGTACAGGTGTTCTGTATGGCGCGGAGTGCGCTCTTGCGTCTTGCGCGTGTCGCGTGCCACACTCCCTCGCTGCGTGTTACGGATGACGACCTGCACACCGTACTGTACGCTGGAGAGGCGGATGGCACTGTGACTTCCCGCGAACGCGCATTGTATCAACGtattttgcattctgcatcccttactgcgcgagatattatgacctgtcgtgcgcaattgattgcaGTTCCGCGCGCGAGTTCTCTGGCGGAGGCAATTGCCTGTGCACAGAAGATGCGCGTTTCGCGTGTTCCAGTGTATGAACGGAGTGTTGATTGGATAATTGGTATTTTCGATGTGAAGAAATTCTTGTGCTCAGAAGAGGTAGACGGACGAGATTTAGAAGAGTGCGGAACACTGATGCAACATGTGAGCGCGCCGGTTTTTGTCTTCGAATGTACACGACTTGCATATGTGCAGCACAAATTGCGCGCACACTCACGCGCAGTCGCCATTGTGCTTGATGAATATGGCGGGACGGCAGGTCTTGTGACAAAGCACAATATATACCAAGCATTTTTTAAGAGCAGTGCGCATGAATTCCCTGTGAATTCAACAGGTCCGCAGGTTACGCGTGCGGGGGTACGGGCGTATATATTtCCTGGATCGCTGCGCTTGGATGAGATAAATgatctactcggcaccgatttttcttcgtgtaccagtgagacccttgcAGGTCTTATCATGGAGTATACAGGGTGTATTCCTGATCCTGGCACCACGGTTGTATTCGGTTCTTGGCGTTGTGTGGTGCTACaactgcatgtgcgcagaatcGTACGTGTTCGGTTTGAGTTTCAAGGATAGTAGGGAAGCGCGCGTGGCATACCCTTTTCTGGTAGGATTGGAACTCATGTTCTTACTTGTGTGCTCGGCCCTATGTGCAGGTTCAGAAAGCGCGTTGTCGTCGGTGAACCAAGACGATGAACGTAAGCTTAAGCGGCACAGTACACGTTGTACACAACGCTTATGCTGGCTTCTGGCCCGGCGCGAACAGCTGATTACCACAGTTATTGTGCAAAACACTGCACTGAATATGGTGCTCTCTAGCGTGGTGACGTTAGGCTCTATGGAGTTGTGGGGTGCACAGTCGGTGTGGAAGGCACTGGTTGCGGTGACGTGCGTGATTATTCTTTGTGGAGAAATGTTCCCGAAGGCGCTGGGTGCACGGTACTCACTGGGATTCTTGATGTGGATTGCGCCTTTTTTGCAATTGAGTTACTGGTTGCTGTACCCCTGCGCGTGTGTGTCGTCAGCATTGATGCATGTGCTGGAGGGTATTTTTTTGCCGCGTCATACGACGTGTCTTTCGCGAGAAGAAATTAAAACGCTTATTGCAGTTGGGGCACGAGAAGGGGTGATTTCGTCAACCGAAAATCAATTGTTCCAGCGCGCTTTACAGTTTAAACGCATACCGCTTGCACACGTTATGGTACCGCACACACACTTTGTGTCagtttttcagGACACACCACTATCTCATGTGTGGGATGCTTTTCGTACCTGTTCCTTTTCTCAGTTGCTCGTGCATGATGCTGCGGGGATCGTCTGGGGTTTCGTACACTACTGGGATATttttggagaaagtaaagtattaccctcGCAGTCATCAGATCagatggtgtcggcatgtgtagnnnnnnnnnnnnnnnnnnnnnntcgggtgggtgatttggcacaggagttgtgttgtgtaccgaatgtagctgaccttttttcagtgttagatatgctctcggtatcaaAGCAGCAGatggcgttagtggtagacgagcggggcgatggggaaggattagtaaccatgacagacnnnnnnnnnnnnnnnnnnnnnnnnnnnnnnnnnnnnnnnnnnnnnnnnnnnnnnnnnnnnnnnnnnnnnnnnnnnnnnnnnnnnnnnnnnnnnnnnnnnnnnnnnnnnnnnnnnnnnnnnnnnnnnnnnnnnnnnnnnnnnnnnnnnnnnnnnnnnnnnnnnnnnnnnnnnnnnnnnnnnnnnnnnnnnnnnnnnnnnnnnnnnnnnnnnnnnnnnnnnnnnnnnnnnnnnnnnnnnnnntttggacgtctccctacggtaggggatgagttggtaattgaaggattgcgttttAAGATACGCCGTGTACTCGATCGGTATGTTGTGTCTGCCCTCGTGGACACTCGAGCATGTAGGTCAAGCGTTGGCTGACGCCTAAGTAAGTACACAGGATGGGGCCGTACTTTTGCGGTATTCGTGCAATTAAGTTGTATGTTGATCGGTGTTCTACAGGAATGtgtGCGCAAAGGGGTGAGAGCATGAGTTGCTATCGTGTTGAAGGTGGGTTTcccgtttcaggttgtattcgcgtgtgtggaaataagaatgcaGCGCTTCCCTGTATTGCGGCGGCAGTTCTCACACAAGAGCCAGTGTTACTGCAAAATGTGCCCGACATTGAGGATGTGGCGGTGATGTTAACTATCTTTCGTGCGTTTGGTGGGAGTGTTGAAAGGCGTGGTAATCATGAGTACATGTTACATCTTCCTCAGTTACAGACGTGCGAAGTGCCGTGCGAGGCTGCGCAGAAAGTGCGTGCTTCCATTCTTTTTGCAGGGCCACTTCTTGCGCGTGGTAGAAAAGCAGTGCTTCCACCACCTGGTGGTGATgttattgggcgcaggaGACTTGATACGCATTTTCTTGCGCTTGCCGCACTTGGCGCACAAGTTCGTTTAGATGGGGTGTTTnnnnnnnnnnnnnnnnnnnnnnnnnnnnnnnnnnnnTTCTTAGACGAGGCATCTGTGACGGCGACAGAGAATGTGCTTATGGCTTCAGTTCTTGCCGAGGGGGTTACGGTAATCACGAACGCGGCGAGCGAACCGCATGTGCAAGATCTATGCCATTTGTTGAATGCGATGGGTGCGCGCGTCTCTGGCATCGGATCGAATGTTTTAACAATTGAGGGGGTGAGCGCGTTGCAtggtACcacatatacacttggggctgatttcatggaagtaggttcgcttattgggctnnnnnnnnnnnnnnnnnnnnnnnnnnnnnnnnnnnnnnnnnnnnnnnnnnnnnnnnnnnnnnnnnnnnnnnnnnnnnnnnnnnnnnnnnnnnnnnnnnnnnnnnnnnnnnnnnnnnnnnnnnnnnnnnnnnnnnnngatctgcgggtaaattacgattttggcggtatgattcctaaaatagatgatggcccgtggcctgcatttccgccggatctgactAGCATTATGACAGTGGTGGCGACTCAAGTCGAAGGGGTTATATTGATTCATGAGAAAATGTTTGAGTcgcgtatgttttttgtggacaagttaATTACCATGGGTGCGCGTATCATCCTGTGTGACCCGCATCGGGCGCTTGTTTCAGGTCCGAGTGCATTGCATGGGAGTGACCTGGTATCTCCTGATGTGCGTGCGGGAATGGCGATGGTGCTTGCAGCGTGTTGTGCGCGCGGGGTAAGTATAATCCGTAACGTGTATCAAATTGAGCGTGGATATGAACGTCTAGTGGAGCGTTTGCAGGCAATTGGTGTCCGTATTTGGAAAGAGAGTTGATCGTATTATGCGGTAATTTGATGAAAAGATGTGAAGTGTTGCCAAGAATCTTTTTTTTTAGTACCCTCGGGGACCGTTACATATGAGGtgttaaccttacaTTTAAGGAGTGAGAAGCATGGCGAAGCAATTGCTGTTTAATGAGGAAGCCCGCAAGAAGCTGCTTTCCGGGGTTGAGCAGATTTCGAGTGCGGTGAAGGTAACGCTTGGTCCTAAGGGTCGCAATGTCTTGCTTGAAAAAGGGTACGGGGCTCCCACagtcacgaaggatggggtttccgttgcgaaagaggttgagctcgaaGATCCGTTCGAGAATATGGGTGCACAGCTTTTAAAAGAGGTGGCTACGAAGACGAACGACGTAGCTGGGGATGGCACAACTACTGCGACGGTATTGGCGTATTCGATGGTGCGTGAGGGTCTGAAGGCGGTTGCTGCCGGTATGACGCCCCTTGAGTTGAAGCGTGGTATGGATAAGGCAGTTGCGATTGCAGTCGATGACATTAAGCAAAATTCCAAGGGTATAAAGAGCAATGAAGAAGTCGCTCATGTAGCGTCAGTATCTGCGAATAACGACAAAGAGATTGGAAGGATTCTGGCAAGCGCAATCGAGAAGGTGGGGAATGACGGGGTCATTGACGTTGACGAAGCCCAGACAATGGAaacggtgacggaattcgttgaagggatgcagtttgatcgtgggtacatctcgtcctacttcgtcactgaccgagataggatggaaacggtgtatgaaaatccctacatccnnnnnnnnnnnnnnnnnnnnnnnnnnnnnnnnnnnnnnnnnnnnnnnnnnnnnnnnnnnnnnnnnnnnnnnnnnnnnnnnnnnnnnnnnnnnnnnnnnnnnnnnnnnnnnnnnnnnnnnnnnnnnnnnnnnnnnnnnnnnnnnnnnnnnnnnnnnnnnnnnnnnnnnnnnnnnnnnnnnnnnnnnnnnnnnnnnnnnnnnnnnnnnnnnnnnnnnnnnnnnnnnnnnnnnnnnnnnnnnnnnnnnnnnnnnnnnnnnnnnnnnnnnnnnnnnnnnnnnnnnnnnnnnnnnnnnnnnnnnnnnnnnnnnnnnnnnnnnnnnnnnnnnnnnnnnnnnnnnnnnnnnnnnnnnnnnnnnnnnnnnnnnnnnnnnnnnnnnnnnnnnnnnnnnnnnnnnnnnnnnnnnnnnnnnnnnnnnnnnnnnnnnnnnnnnnnnnnnnnnnnnnnnnnnnnnnnnnnnnnnnnnnnnnnnnnnnnnnnnnnnnnnnnnnnnnnnnnnnnnnnnnnnnnnnnnnnnnnnnnnnnnnnnnnnnnnnnnnnnnnnnnnnnnnnnnnnnnnnnnnnnnnnnnnnnnnnnnnnnnnnnnnnnnnnnnnnnnnnnnnnnnnnnnnnnnnnnnnnnnnnnnnnnnnnnnnnnnnnnnnnnnnnnnnnnnGGCGGTTGGTTTTAAGATTGTGCGTCGTGCTCTCGAGGAGCCGATAcgccagatttcagagaacgcgggtattgatnnnnnnnnnnnnnnnnnnnaggcaaaggagaaacgtggcatcggttttgatgcatccaagatggaatgggttgatatgattaaggtcgggattatcgatccggcgaaggttacacgttcagcactacagaacgcnnnnnnnnnnnnnnnnnnnnnnnnnnnnnnnnnnnnnnnnnnnnnnnnnnnnnnnnnnnnnnnnnnnnnnnnnnnnnnnnnnnnnnnnnnnnnnnnnnnnnnnnnnnnnnnnnnnnnnagttagcgccggtcgatTGGTCATACGTAAGGGCATGATCTCTTGAGATGAGCGTCTCCCGTGGATCTCGGCCAGCGTGTGGTGCGGGTAATTCCCCTGGCGCCGCTTCCGGTCCGTGTATACAACGCCGGTGGTCTCCGTGTGGATTTCTttccgcggttttttgggnnnnnnnnnnnnnnnnnnnnnnnnnnnnnnnnnnnnnnnnnnnnnnnnnnnnnnnnnnnnnnnnnnnnnnnnnnnnnnnnnnnnnnnnnnnnnnnnnnnnnnnnnnnnnnnnnnnnnnnnnnnnnnnnnnnnnnnnnnnnnnnnnnnnnnnnnnnnnngatccggatctagggattctttcgacttatgggcgaagatctcttgcctggccgagggatttttcttttccacGCGTGCGCGTTGGCGAGTACAACGGCGCACGCGGCACAGGCCGCGGTCTCTGTGCGCAAGGTCCGTCTTCCCATGTCTACTGCTCTAAATCCCATGGCGGAGAAAAGTAAACGTTCAGATTCGGTCCATCCACGCTCACTCCCTATTGCAAtccacagtacatccccgcgcagcgcggcgcttcctagatcnnnnnnngtgttcttagtatcaaGTATTAGTTTTTGGTGTGTGCTGTCGCCGAGTATGTGGGTGTGTTGTGAGCAAAAGGTGCGCACCGACTCCGAAACAGTAATGAGGGGAAGTTTCGTGCCTCCTGCCTGTTCTAGGCCACGTATGAGGTACGTGTGCATTTTTTCCATGTGAGCAAGTCCTGAGTCTAGGTAAGATCGCTCCCCTAATTCCGTCCCTACAAGATGGATAGAGGAGATCCCGAGGCTGGACACGTCGCGTAAAATGCGCCTGAGCTGAATGGGACGAGGGAACCCTATGACAAGATGAAGGGGGAAAAGGGCACAATCTTCGTATTCCAGTTTTTCAAAAACGGCTACGAGATACTTTTCTGTGGCTAGGGAGATGCGTGCAGAACCTTTCACCCCATTAATAATCCCTGCTTTGAAGCAGGCTCCCGCACTCAATTTAAGCACACGCTTGATATGGCAAAATCGACTATCCCTGAAAGAAAGTACAGCGCAACCGTGCACTACCTCTTCCTGTTCAAAGAGAACGATATTCATTTGTCATTTCTTGTGTGGGTAAGAAnnnnnnnnnnnnnnnnnnnnnnnnnnnnnnnnnnnnnnnnnnnnnnnnnnnnnnnnnnnnnnnnnnnnncatagaacactggcctgatgacgcggataccaATATCAGAAAGAATAGTGCGTCCATGTAGGACAATGTCCCAGGAATTCCAGCGCAAGAAACGCCCCAGATATATACCGAATGCACAGagatacaattcaaatactgaaattatgaatggnnnnnnnnnnnnnnnnnnnnnnnnnnnnnnnnnnnnnnnnnnnnnnnnnnnnnnnnnnnnnnnnnnnnnnnnnnnnnnnnnnnnnnnnnnnnnnnnnnnnnggtcatagtaaagcaaaaatgacttaccctttcccaagtggataatatcggtaaggatgtacggnnnnnnnnnnnnnnncaatagccagagcagcataaggaacagctgtacactacggacagcgaagcggcgcacgtgcaagatagccgagatgagccaGGGGATGAAGGCAAGAACCAGATTCCACACAAGGAAAGAAAGGAAATTATCCGCCGCCACACAGCGCnnnnnnnnnnnnnnnnnnnnnnnnnnnnnnnnnnnnnnnnnnnnnnnnnnnnnnnnnnnnnnnnnnnnnnnnnnnnnnnnnnnnnnnnnnnnnnnnnnnnnnnnnnnnnnnnnnnnnnnnnnnnnnnnnnnnnnnnnnnnnnnnnnnnnnnnnnnnnnnnnnnnnnnnncgataaatcctgttaatgttgtgtttcatcctttcgtagtacgaaaggttctcctcgtttttttccataGTGTAGATGGTTTCAACTGAAGCACCAATCTCTTGcgcaagaacgcgagcgactttttcactaggaccacgttcactaaaaannnnnnnnnnnnnnnnnnnnnnnnnnnnnnnnnnnnnnnnnnnnnnnnnnnTGATGGAAGGTTCTTctgtgttaaagacgtcctttatactcttttgctggaggtcaaaatccctgcacaagtaaccaaacgCCGCATGCGTGGTCACAAAATATCGATGAGATAAGGCTGCAAACTTGGAGCGGTATTCCTTGTACAACGCCTCAAGCCGTGCGGCCGCATTACGGGCATTCCTTTTGTAGAAGCGCGTATGCTGCGGATCCGCCTTACACnnnnnnnnnnnnnnnnnnnnnnnnnnnnnnnnnnnnnnnnnnnnnnnnnnnnnnnnnnnnnnnnnnnACGCAGACCCGTGCGATGCATGTGCATGTACATGGTGCGCCGTATGCCCACGTGTGTGAGCCTTCAGCGGAACAATGCCCTGCGCAGCTTCTACGCGTATACTGCCGCTATTACGTGCAGcatggagtaccgagtgtatccagggttccatgccaagaccaTTATACACGatgaccttggcatCACTAATGAAGGCCATGTGTTTTGCTTTTGGTTCAAAGTCGTGAGAGTCAACCCCATCAGGAACGATGGACACTAAATGAATTTTTTCTCCACCTACCATCTGTGTTAACTCTTTGAGAGCATTAAATGTCACCACAACAGGCAGCTTATCCGTGTAGGTTATGCGAGAAGGTGTTGATAGCGAACACGCCTGTGCAAGAAAAACCACCCCCGCAAGGGCGGCAACTACTGAGCAGCGTTGCATGATGGCTCCTTTGAAGGGGCTTCCACCCCAAACnnnnnnnnnnnnnnnnnnnnnnnnnnnnnnnnnnnnnnnnnnnnnnnnnnnnnnnnnnnnnnnnnnnnnnnnnnnnnnnnnnnnnnnnnnnnnnnnnnnnnnnnnnnnnnnnnnnnnnnnnnnnnnnnnnnnnnnnnnnnnnnnnnnnnnnnnnnnnnnnnnnnnnnnnnnnnatggctagtcccttcgcggtgcttttacaaaacgttagtttcaggtacaccgcagacgcgcgctttatactgcacnaggtggatcttgcaatACCAAAAGGCGCGTACCTTTCCGTTGTAGGAGAAAATGGTAGTGGGAAAAGCACGCTTGTGAAGCTTGTCCTCAAATTACTTAAGCCGAGCACCGGTACGATCGCTCACTTTGTACAGCGTGTTGGTTCTGTTCCACAGACAAAGATGCACACGCTCTATTTTCCGCTTACGGTATATGAAATGCTTAACTCGTATCGCAGACTTCTGCGCATATCGCACAAGTGGGTTGTCGATGCTGTGCTGGAnnnnnnnnnnnnnnnnnnnnnnnnaaaaaagcttgtgtataccnnnnnnnnnnnnnnnnnnnnnnnnnnnnnnnnngcgcggagcctgatcggagatcctgatttgctggtgctcgatgagctgtcaacaggtattgattctcgaggacagaaagatatttacgctctgctgaagnnnnnnnnnnnnnnnnnnnnnnnnnnnnnnnnnnnnnnnnnnnnnnnnnnnnnnnnnnnnnnnnnnnnnnnnnnnnnnnnnnnnnnnnnnnnnnnnnnnnnnnnnnnnnnnnnnnnnnnnnnnnnnnnnnnnnnnnnnnnnnnnnnnnnnnnnnnnnnnnnnnnnnnnnnnnnnnnnnnnnnnnnnnnnnnnnnnnnnnnnnnnnnnnnnnnnnnnnnnnnnnnnnnnnnnnnnnnnnnnnnnnnnnnnnGTTGTGTCCTCTTGTGGGGATGCACTTAGTGCTCCGCCGTTACGCGCTCATGGGAGATGCACTTGCGCACGGTTCACTTGCAGGAGTATCTATTGCCGTTTCGTGCGGCATCCATCCAGGATGGGGATCCTTTTTTTTCACTGCCTTGGTGGGGGTTTTGATAGAGTTTTTGCGCGCCTTTTTCAAAAACCATCATGACCTGATTCTTTCAATAGTACTCTCCCTGAGTGTGGGTATTGCTGTCACGCTGTTGAGCTCTGGGCTTATCCAGGCGGATATAGACAGCTATCTGTTTGGCAGcatactggttgtttctacacgcgaTTTGTGGATCATGCTCGCACtcagcgtgttctgcgtaggtacgctagcgttgcgctaccatcagctgctgtACTTGGCATTTGATGAAGAGACCGCGCGTATCTGTGGCGTCGCAGCAGACGGGATAAACTACGTCGCGTCGGTGGTAATTTCAGCGACTATAGCAGCTTCTATAAAGATTACCGGTATTTTGGTGCTGAGCTCGCTCATGACCGTACCGGTTGCGACAGCGCTACAGCTGCGGGTGGGTTTCCTGCTTACGTTGGTGGCGGCGTTTCTATTTAGTATGCTGGACACGGCGCTGGGTnnnnnnnnnnnnnnnnnnnnnnnnnnnnnnnnnnnnnnnnnnnnnnnactggtctcggtggtgGTACTGATGTTGGTTATCGCGCTCACACAGGTGGGCCGCGCCCGCACGTAACCGGCTGGACGGGGTCCCGCGGAGCCCACAACCCACCCTGAGCTGGCGACTCCAGACTGACCGATTAGCCAGTCAGACTCACACGGGACCAGGGACACCTGGCGTCAGTTACAGCTTGATTTCAGCGGCACAAGGCTCCCCACGCAGCACCGTGGTGCAGGCGTCAAGCGAATTGAATACCATGTTCTCTATCGCTGTTTCTGTGTAGAAGGCGATATGAGGGGTATAGATGATACGCTCATGTGCGACAAGCCGAGCATAGACCGTATCGGTAATAGGGTTGTTcccgttgtctttaggaatatacggaccctcaanctcgtacgcannnnnnnnnnnnnnnnnnnnnnnnnnnnnnnnnnnnnnnnnnnnnnnnnnnnnnnnnnnnnnnnnnnnnnnnnnnnnnnnnnnnnnnnnnnnnncgccatctttcatctgcgcgattgttntcgcattgatcaggtgatgactgtcctttgtcgcaggcatgtgcaagctgatcacgncgctagtggacagcngctcgtccatactcncgnnnnnnnnnnnnnnnnnnnnnnnnnnnttcgggtannGATCAAAACCAACTACCTGAGCACCAACCCCTTTGAAGAGCCTTGCTGCTGCCTGTCCAATCCTGCCCGTTCCCAAGATACCTACGCGTGAGCAGCGGAGCTCCTTCGAGAGAATTGGTTTTTGCCAGCGAAAATCACGCTTCCTTACAAAAGTTTCAATCTCGCGCGCATGTCGCGTCAGCTGCAACGCGGCGGCGAGTGCATATTCCCCGATAGCATTCGGTGAATAGGACGGTACGTTCGTCAGCCGGATGCCGTGCTTTTTCAGCAAAGTTGCATTATACATATCAAAGCCTGCGGTACGTGTGGAAAGGACTTTTAGTTTGCACGCACCCATGTAGTCATACACctcgtcttccatagcgagaaactgcataaccacgagcccttcatacCCCTTAAGCaacTCCTTGTTCTTTGCGGTCAACGGTTCCTCGTAAGTGTCCACGACTACCCCTGGATGGGACTGCTTCCACTTCTCCACGTAAGGGGCTTCTTCTTCTCGAAGATTAAAGACAACACACCTCATTTCGCACCTCCTTGGTAAGGCAAACAACATTCCTTACCCACGTTCTGCACGTATCTCACGCAGGACCTGGGCGGAAACACATAACATATAGGATATCTACCCTTGTTCGGGGATAAAGAGTTCTCTCCGGTAAAGTTTGAGGCGGTCAACGAGAATAAAGTCGATAAGATACGCAGCAAGgaaggaaagnacgAAATACACGAggaAAAGAATACCCGCGCGCACCATAGGAGTGTACGCCATAAGCCTGTAGGCGTTGATCGGTCCAACAAGTCCAATAAAACCGAAGCCTGCGCTTGCAGGAGTACCCTGCAGATTGAAAAGCCACGCGAGCACGCCGCAAACGAGGCCATTGAGCAGGAGCGGAATATTGaGAATCGGATACCGGATCCAATTTGGCATGAGCATTTTCATCGCTCCTGCGAACATCGCCAACGGAACACCGATCTTGTTGACGCGCATGGTTCCCACAATGAGGGTCATGGCGCAGGAGGAGACGCCGATGTTTGCTGCTCCACTTGCCAGACCGGTGAGCCCCACGGCGATTCCTACCGCGACGGAAGACACAGGGGAGATGATGATGAGCGAGAAAGACATGGACAGCAGGATACTCATGAGTAATGGCTGGAGCGCGATGAACGTGGCGATAACTCTGCCGACGAAAAACGTAATCATTTTTACGTAGGGCAGGGAAAAGGAGCCGACACCCCCTGCGACAACAGCTACGATAACGGGCAACGCGATGATGGTCAGCGAACCAAGTTTCCCCCGCAGAGCACGGACGAGTATGATTGCAAGTGCAGATATGAGCATAACATtgatgacgtCCCCGATACCGGTGATCAACCAGGCGCCATTTTGAAGCGTGACATTTCCTGAGGCAATAACAGAAACAAAGGCGAGGGTAGCCACTTCGGGCGCGGAGCAGTGAAACTGAAGTCCAACAAGGGTACCGATGAGAGCCGGTACACTGAACTGTATGGGCAGCACCACATGGTAGAGCGCGGCGAACAGCGGCGAAAGCGGAGCAAGCGCTCTGAACAACTCCCCCGCGATAGCGGGGGGGACAAGACCGATGACGATGCCGGCAGAAGACCCGTTGAGTnnnnnnnnnnnGAACTGCCTGGGGCTGAGGCTTTGCGTGTGCATGAGACGCTCCTTACGGAGAGACTTAAAATGGCGCGGACTGCAGCCAGTGTGAGGCAAGAGAATAAACCGtgtcaacagttttattttgttttggatgaaaaagcgttgcagtccccactgcgggagaacccttccaaaaacgtgcgcactatccctgatgcgggcgatgaaaattcctcatttgggcatgcccgtgtgnnnnnnnnnnnnnnnnnnnnnnnnnnnnnnnnnnnnnnnnnnnnnnnnnnnnnnnnnnnnnngtgaaatatcgtgccattacgtaCTCGTATGTCTTTGCTTTTGCCCTTTCTGCTGCGGTTTCACACACTGCGCTCCCGGCGCCTCCTTCGGCAGAAAACGGAGTGCTCGATCTCCGTCAGTGGGATTTTTCCTCTGGTGATGCACTGCTTTCTCTCTCGGGAACGTGGGGGTTTcactggaacgagttacttactcactcagctgagcagcctgctgcgtttatgaccgttccctcccnnnnnnnnAAACCGCGGGGCGCGGTTCAGATACCCGCTATTCAGGCGTATCCTCACTACGGATGTGCCACCTATACGCTGAAAATCTTGCTTCCCCCTCGCGCTCCGTCGCTTGCGCTGTCCTGCGAtacgcttaactacgcagcaagaatctacgcaaacgggcaTCTCTTAACAGAACTTGGTACCGTTGCGCGTAACCGAGCAGATGCCGTCCCCTACGTCCACCCTGCCGAAGTGTGGTTGCCAACGCAcgaaCCTGAAATTGACGtgtgCATTCAGGTAAGCAATTACCACAGTCTAAGACCGGGCATTGTCGGTGAAGTTCACTTGGGGTCTGTTTCGCGCGTTCGTACGCGCGTGCTGCGCAAGGACGTGCTTGAGGCGATGACAATTGGTTTTGCGTTTAcgatattcgcgtattacnnnnnnnnnnnnnnnnnnnnnnnnnnnnnnnnnnnnnnnnnnnnnnnntatgcggagaatgagcgggcagATACTGCAGGGGTGTTGGGAGTGAGCGAAAAGAGCCTGTATGCGTGCTCGCTTTTTTCGTTGCTGATTGTGGTnnnnnnnnnnnnnnnnnnnnnnnnnnnnnnnnnnnnnnnnnnnnnnnnnnnnnnnnnnnnnnnnnnnnnnnnnnnnnnnnnnnnnnnnnnnnnnnnnnnnnnnnnnnnnnnnnnnnnnnnnnnnnnnnnnnnnnnnnnnnnnnnnnnnnnnnnnnnnnnnnnnnnnnnnnnnnnnnnnnnnnnnnnnnacgcggtgattatccttttgcttccccctgcctcttttgcccgattacttcccttacagcaactgttcgtattgcttttgctgggtattgtgctctgggtgatgtgcaaagtcttgtaccgcaaaaagcgtggnnnnnnnnnnnnnnnnnnnnnnnnnnnnnnnnnnnnnnnnnnnnnnnnnnnnnnnnnnnnnnnnnnnnnnnnnnnACTGTCAGGAGCTCCCCTGTTGATTTTTGGGTTTGTTGGATTTTTGGTTACGCAAACTGCGCTCATCGAGTGGCAGGTTGTTTTGGATGGGCGGACTGCGCAGCGGCTTTCTCAGGACTTATCTTCCTCGTCGCAGCGTTTGGAGCGGTTGTTTGGTGAAGTTCGTCGCACCGCGCAGGAGCTTTCGCACAATGAACAGAATCTGACCGAGACCATGGATCGTGCAGAGCAGGTAGTGCAAGACTTGACGCGCTACACGGGTGCTGTACGCGAAGAGTTAGCGGTGCAGGGGGAAGGGTTTTCTCAAACGCGTGTGGTGAATGAAGAGCTTGGTTCCTCCTTGCGAGAGTTGGATGAACAAAtggaagcacaaggggaacgtgcacgagaagnnnnnnnnnnnnnnnnnnnnnnnnnnnnnnnnnnnnnnnnnnnnnnnnnnnnnnnnnnnnnnnnnnnnnnnnnnnnnnnnnnnnnnnnnnnnnnnnnnnnnnnnnnnnnnnnnnnnnnnnnnnnnnnnnnnnnnnnnntcgaagaaattacgagtcggncgcgtggtttggcggtgacgaatgcgctggtggtagatatttcaggacggacgaatttacttgcaatgaacgcAGCGATTGAGGCTGCACATGCAGGTGACGCAGGACGCGGTTTTGCGGTGGTAGCGGGggaaatcCGCTCCCTGGCAGCGCGAACAGCAGCTGAGTCTGGTGCAACGGGAAAAATGTTAAAGGAAATAGAGGCAGTGATTGGAGAATCAGGGCACGCGTCGGCAGGAGTTGCCCAGAGTTTTACAGACATTAGAGGAAAGGTGGACGGTTTCAGCACCATCCTTGCGGATATTTCCGGTGCCGTTCAGGTGgtgggagaagaaaatgagcgtaccGTTGAGCGnnnncgcacggtgacagggcagttatgtacggcgcgtgagcaggggaaaGTGATAGCAGAGGTATGCACGCGAGGTGCAGCTCATGCTGAGCGGTTGACACGAGATGCGCAAAAGGTGTGCGCAGAGGTCGAGACGATGATCGGTAACGTGGAAGtgcncacggaagtggttgcacgaacgcgggcagttgagttgcataCGCGGGAGGTAATCGCCCGCCTGAGTGGGCTCTTGGACagcaatataccaacggacgaacctcagtcggaatacaggnnnnnnnnnnnnnnnnnnnnnnnnnnnnnnnnnnnnnnnnnnnnnnnnnnnnnnnnnnnnnnnnnagtctcagcgagcgccggcctcccccncccnnnnnnnnnnnnagattgacaaaagggtttcgcctgactatgctcgggcgggggtgcgctcatgagtcaggagatcggtatcaagctcgcggncgggtcttttttccctctctTTACTGCAGagggtgcgcaGCGCACAACGCTCGAGCTCGTTACCGCGAATGACCAACAGTCTCGTGCTCTTTTGAGCTTTTTTAAACGGGTTTCTCCTGGCGATGGAGAGGCGTCGGTCACCTCTGCAGAACTTGAGCCTATGGGTGCGCTttcgatagacaCGTTGTCCnnnnnnnnnnnnnnnggagctactattcgcctcgaggttgagngtgatgggagncggtgCCGTGCGCGTGCGACGGATGTAGGTTCTGGTGCTTTTCAGGAAACCTCTTTCTCCGTGTTGGAGAGATCCTCAGGAGACGCGACACCGGATGAGCAGCCTGCTGCTCAGATCGAGCATGTCAGTTCTTTCCCTGAAGACGAGGGAGGGGATGGTTCTACCCTCACGAGGAGACGACGGATTTTGTTGGCGGTGTGCGCATTTCTCATTCTGCTCGGTGGTGTCTTGGTAGGTTGGGTTCTGTACATGCACGGCGCCTCTCGTCCTGCGGTCGTGCCGTCACAAAAAGTTGAACTGCCCAGGTCTTCTGCGCATGTTGCAGCGCGTGAGCTTGAGCGTACTGCGGTTGAGGCACGTGTCGTCGATCTCCCATCTGAGACTGCTCTTCCAGAAACCCACAAGGAAAAGGACGTCCCCCCTGCGTCTCCCGGGGCTGAAACGGCTACGAGTGCAGCGGCAGAACCTGTAGAGGAGTCTGGGTCTGGATCCGTCAAGGTTGTGCGtTATACGGTCAAACGGGGGGATACGCTCTGGGATttggcgcggagttattacaagactccgtggcgctacatgagaATTGCTGAGTTTAACCGACTGAAAAACCCTGATCATATCGTTGCAGGAACCTCAATTGAAATTCCGTCACGGTAAGCATCTCGtgtggggaggtggctnnnnnnnnnnnnnnnnnnnnnnnnnnnnnnnnnnnnnnnnnnngtgcgtggtgnnnnnnnnnnnnnnnnnnnnnnnnnnnnnnnnnnnnnnnnnnnnnnnnnnnnnnnnnnnnnnnnnnnnnnnnnnngacaaaaactacactccgtttctaaagcCAACAGATGAGTTTCTCGAGCAGGTCGCCGCGCGGGAAgGTGCAAGTTACTTCATTGGGTTACACCTCAAGCGGGCCAAGTTTTCCGAAGAAGCTCACGAGTACTTCGTACGTGGCGCAGCGCAGGCCGCACCGTACCGCCAGTTATGTGCGCATGAGGCACACAATACAGGGAGTCCACTACAGCGTCTCGCCTTTATTGAAAAACAGCTGCATGTCCTTAATGCAGGCAGCGATGCGCAAACCAAAACGCAGcaacagacacttcgccttttgcgttcaagggtgctctttGAACTCGAACGCTACTACAGTCTGAGAACGGTTGTTGAGTCTTGGTACAACGATAGGGCACTTGCGCCCCATACGAGTGCACAATTTGCTGCGCTTATTGCGGCACTGCCGGATCTGCCGCGTATCTTTAAGGAAGTACATGGTGCTCGTnnnnnnnnnnnnnnnnnnnnnnnnnnnnnnnnnnnnnnnnnnnnnnnnnnnnnnnnnnnnnCTTCTGCGTGGCACACGCGGTACGCTACAAATTCGGTTCTCTCCGATTTTGGGAAAGCAGCGCTTTACGGTAGTGAAAACAGTGTTAAAGCCGCGCAGGTTTTTCTTGACCATCTTGCACATCTATCGCGCTCAACGTTGTCCAATGCGGAATTAGAGgcacgactgcgtttttattgctatttttatgcggcgcgtcttnnnnnnnnnnnnnnnnnnnnnnnnnnnnnnnnnnnnnnnnnnnnnnnnnnnnnnnnnnnnnnnnnnnnnnnnnnnnnnnnnnnnnnnnnnnnnnnnnnnnnnnnnnnnnnnnnnnnnnnnnnnnnnnnnnnnnnnnnnnnnnnnnnnnnnnnnnnnnnnnnnnnnnnnnnnnnnnnnnnnnnnnnnnnnnnnnnnnnnnnnnnnnnnnnnnnnnnnnnnnnnnnnnnnnnnnnnnnnnnnnnngggcgtctggctacactgcaagaggtgctgacgcntagagcattacccgagcgctccgcgcgcgttacgtatgtccttgcgcgTTCAGGTACGCTTTCAGAAGAGAGTGCGCGCCGCGCTTACCGTACCATCTTcgaaactgcgcattcttnnnnttactatcgtgttcttgctgcaTGTGCATTGGGCATTCCTCTGGAAGAGGCTTTGTATAAGGTGCGGAGTAAGAGAACACCGCATCCTTTTCTTACCCCCGATGAGTCGCGTGCTATTCTCCAAGGTTATGTAGACTATCACTTGGACGATATGTTCTACCAAGCTATGGTACAGTTCTATCCTGACATTCCTCTCCACCTTGCAGAGCATTTTGCGAACGCACACATACAAAGATCACGATGGTCCGATGCCGTGCGCATTCAGTCGTACGCAATACGCTCTCACGGTGCGCGCTATTCTGTAGAACACTTGAAGATTGCGTACCCGCGTCCGTGGCTTGACGTCATACAAGGCtatgcggggaagtatcatattgaggaGTATCTGCTGTTTGCGCTCATCCGGAGTGAAAGTCTGnnnnnnnnnnnnnnnnnnnnnnnnnnnnnnnnnnnnnnnnnnnnnnnnnnnnnnnnnnnnnnnnnnnnnnnnnnnnnnnnnnnnnnnnnnnnnnnnnnnnnnnnnnnnnnnnnCCCGGATATCaatgttagattCGGCAGTCTGTTCTTTTCCGATCTCATTCGTCGCTTTGATGGCTCCGTGTTCTGTGCCCTGTTCTCCTACAATGCGGGACCGTCGCGCGTCCGCAAATGGAAAAAACAGAGGGGAAGTCTGCCCGATGATCTGTTCCTCGAAACCCTGCCTcttgcagaacctcgggagtacgggaggaagatcctatccgcagcggtgatgtacgggtacctnnnnnnnnnnnnnnnnnnnnnnnnnnnnnnnnnnnnnnnnnnnnnnnnnnnnnnnnnnnnnnnnnnnnnnnnnnnnnnnnnnnnnnnnnnnnnnnnnnnnnnnnnnnnnnnnnnnnnnnnnnnnnnnnnnnnnnnnnnnnnnnnnnnnnnnnnnnnnnnnnnnnnnnnnnnnnngctggccgctgcccgtatgGGGGAGCACACGTTACTCATCACTCAGACAATCGATAGTATCGGCAGGCTCTCATGTAATCCTTCCATTGGAGGAATTTCCAAGGGGAATATTGTAAGAGAAATCGATGCACTCGGCGGAGAGATGGGAAAGtttgcggatgcatgCATGAttcagtatcgactgctcaacaaaagccggggccctgcnnnnnnnnnnnnnnnnnnnnnnnnnnnnnnnnnTTTGTATGCCCAGAAGGTGAAGTATACGTTGGAATGTACGCAGCATCTTCACCTGTATCAGGACACAGTGGTAGACgttgtgtgttccaataccactgatgcaggatatgtagnnnnnnnnnnnnnnnnnnnnnnnnnnnnnnnnnnnnnnnnnnnnnnnnnnnnnnnnnnnnnnnnnnnnnnnnnnnnnnnnnnnnnnnnnnnnnnnnnnnnnnnnnnnnnnnnnnnnnnnnnnnnnnnnnnnnnnnnnnnnnnnnnnnnnnnnnnnnnnnnnnnnnnnnnnnnnnnnnnnnnnnnnnnnnnnnnnnnnnnnnnnnnnnnnnnnnnnnnnnnnnnnnnnnnnnnnnnnnnnnntctttcggtaatggaaaagcaagaagccgACGCTATCATGCGTCCTTTTTCCTTTGCTCACGTGGAAATCAATCGTCCACATGCGGACTGTTATATTAATTACACCAATGAGAGAACgcatcagctcatccgtgagaattttcatcgttctnnnnnnnnnnnnnnnnnnnnnnnngcggtagggacacgatattgtccgtcnnnnnnnnnnnnnnnnnnnnnnnnnnnnnnnnnnnnnnnnnnnnnnnnnnACATAGAACCGGAAGGATTAGATACGGAAGAGTTATATATAAACGGGCTTTCATCCTGTTTGCCAGAGGATATACnnnnnnnnnnnnnnnnnnnnnnnnnnGGTATGGAACGCGCGGTTATTACGCGTCCTGCATACGCGGTGGATTATGCGGTGCTATTCCCTGTACAACTTGGTATTGATTTGCAAACAAAAAGGGTGAGCGGGCTCTTTTCTGCAGGTCAGATTAACGGAACATCCGGCTATGAAGAAGCTGGAGGTCAGGGTATTATCGCCGGGATTAACGCTGCGCTGTACGCGCGCAGTActaaaacnnnnnnnnnnnnnnnnnnnnnnnnnnnnnnnnnnnnnGAAGCATATATTGGCGTCATGATAGATGATCTTGTAACACAAGGAATAGACGAACCCTATCGGATGTTTACCGCGCGTGCGGAGTATCGTTTGAAACTCCGTCACGATACTGCGGATGAACGTCTTACAGAAAAAGCTTACGCCATTGGGCTGCAGAAGAAATCTGCTGTAGAAACGTTGCAAAAAAAGATGCGTACGAAGCACGAGATCTTGCATCTGCTTCAGACnnnnnnnnnnnnnnnnnnnnnnnnnnnnnnnnnnnnnnnnnnnnnnnnnnnnnnnnnnnnnnnnnnnnnnnnnnnnnnnnnnnnnnnnnnnnnnnnnnnnnnnnnnnnnnnnnnnnnnnnnnnnnnnnnnnnnnnnnnnnnnnnnnnnnnnnnnnnnnnnnnnnnnnnnnnnnnnnnnnnnnnnnnnnnnnnnnnnnnnnnnnnnnnnnnnnnnnnnnnnnnnnnnnnnnnnnnnnnnnnnnnnnnnnnnnnnnnnnnnnnnnnnnnnnnnnnnnnnnnnnnnnnnnnnnnnnnccgtacacgattggaacacgttcgcccggacactatcgggcaggttgggagaatgcgcggaatcagaCCCTCTGACGTAATGCTGTTGCTCGCCCACTTAAAGCGGTAGCAGCTACCGCAGAGATAGAAGAACCGCCTTGTCAGGCAGGTGtttgtacgtacttttaacgcacagcaaggagcgcttcggcgtgaagttcggtgataaggccacaggannnnnnnnnnnnnnnnnnnnnnnnnnnnnnnnnnnnnnnnnnnnnnnnnnnnnnnnnnnnnnacccaacacggacccctgcatccatcattttttttataggatgttTTTCTAGGCATTCCACCGCGTTGAGCATGACnnnnnnnnnnnnnnnnnnnnnnnnnnnnnnnnnnnnnnnnnnnnnnnnnnnnnnnnnnttctCGTCAGTAGCGGCTCCGATGCCATGTTGGACTTCGTCCAGGTTAAATATTTCGACAAAGTGCCGTACCGATTGCGCATCAGAGAACTCGCCTACGTGGGCTTTCTTTTTTAAGTGATACTTCTCGGCCAGTTTAAAAAtatagatgannnnnnnnnnnnnnnnnnnnnnnnnnnnnnnnnnnnnnnnnnnnnnnnnnnnnnnnnnnnnnnnnnnnnnnnnnnnnnnnnnnnnnnnnnnnnnnnnnnnnnnnnnnnnnnnnnnnnnnnnnnnnnnnnnnnnnnnnnnnnnnnnnnnnnnnnnnnnnnnnnnnnnnnnnnnnnnnnnnnnnnnnnnnnnnnnnnnnnnnnnnnnnnnnnnGGTGATAAAGccaatatcaactgatgtctccattacnnnnnnnnnnnnnnnnnnnnnnnnnnnnnnnnnnnnnnnnnnnnnnnnnnnnnnnnnnnnnnnnnnnnnnnnnnnnnnnnnnnnnnnnnnnnnnnnnnnnnnnnnnnnnnnnnnnnnnnnnnnnnnnnnnnnnnnnnnnnnnnnnnnnnnnnnnnnnnnnnnnnnnnnnnnnnnnnnnnnnnnnnnnnnnnnnnnnnnnnnnnnnnnnnnnnnnnnnnnnnnnnnnnnnnnnnnnnnnnnnnnnnnnnnnnnnnnnnnnnnnnnnnnnnnnnnnnnnnnnnnnnnnnnnnnnnnnnnnnnnnnnnnnnnnnnnnnnnnnnnnnnnnnnnnnnnnnnnnnnnnnnnnnnnnnnnnnnnnnnCCAAACGTTCTGCATTTACGAAAATTTCCTAGAGGAGGTGGGCTAAGTAAtggtattgggcagggaaacctgcgagtgtgttctatacgacgggacaAAGACAATCAGCAGCACGAACAGATCAACGTCCCCTTGCTAACnnnCCCCACCACGCTCACATCGGTTGTACACGCGAACACACnnnnnnnnnnnnnnnnnnnnnnnnnnnnnnnnnnnnnACTTAGGTGCGCCACACAGGCTACGTGTGTaacgatcattcctcaaacgtcaaacaacagcgcaatcgtccgcaannnnnnnnnnnnnnnnnnnnnnnnnnnnnnnnnnnnnnnnnnnnnnnnnnnnnnnnnnnnnnnnnnnnnnnnnnnnnnnnnnnnnnnnnnnnnnnnnnnnnnnnnnnnnngcagtcaattccgcctactatccgcgcttcttcccgttcatttatctgtcgcagttcgactctcgtaccaaatgtagctcctaagtctctaactaattctctgaAATCAACACGTGCTGGCGCACTGTATAGAAATACCACGCGTGCTTCTTCTAAAGGATAGTGAcagCAAATACATTTCATCTCAAGTTGATAGTGCTCAATGCGATCCCGAAAAATCATTTCCGCAGCACGTTCCTTTTCCCTATGTATGTGCCATGTGCACAAATCTTGGTCTGATGCAACACGTACCACACTCGCGATATCGGTTTGTACCGGTGtacgtatacgccctacgcaccgagcaatgtcgttgccataacgcgtgggtaccnnnnnnnnnnnnnnnnnnnnnnnnnnnnnnnnnnnnnnnnnnnnnnnnnnnnnnnnnnnnnnnnnnnnnnnnnnnnnnnnnnnnnnnnnnnnTTCCTCCTCCATGGGCTGCGGCGGAAAGGCAGGGGTGGAAGCACACGCTGTGCAGGGGTCAGCATnnnnnnnnnnnnnnnnnnnnnnnnnnnnnnnnnnnnnnnnnnnnnnnnnnnnnnnnnnnnnnnnnnnnnnnnnnnnnnnnnnnnnnnnnnnnnnnnnnnnnnnnnnnnnnnnnnnnnnnnnnnnnnnnnnnnnnnnnnnnnnnnnnnnnnnnnnnnnnnnnnnnnnnnnnnnnnnnnnnnngtatcaggcatacctgttcccctgtctatcctgatacnnnnnnnnnnnnnnnnnnnnnnnnnnnnnnnnnnnnnnnnnnnnnnnnnnnnnnnnnnnnnnnnnnnnnnnnnnnnnnnnnnnnnnnnnnnnnnnnnnnnnnnnnnnnnnnnnnnnnnnnnnnnnnnnnnnnnnnnnnnntacgccaaccgtgtctgatgttcttcgtgaacaagagacggatcctgcactgcagagtgcgggtccgtcttaggatnnnnnnnnnnnnnnnnnnnnnnnnnnnnnnnnnnnnnnnnnnnnnnnnnnnnnnnnnnnnnnnnnnnnnnnnnnnnnnnnnnnnnnnnnnnnnnnnnnnnnnnnnnnnnnnnnnnnnnnnnnnnnnnnnnnnnnnnnnnnnnnnnnnnnnnnnnnnnnnnnnnnnnnnnnnnnnnnnnnnnnnnnnnnnnnnnnnnnnnnnnnnnnnnnnnnnnnnnnnnnnnnnnnnnnnnnnnnnnnnnnnnnnnnnnnnnnnnnnnnnnnnnnnnnnnnncaccacaatttttcgggtaagcactgcgcccagaataactccagaagggagcacctgcaccgcctctgacgcaacgatatctccttcaaccattcctcctacgatgatgctccctacccgtatgannnnnnnnnnnnnnnnnnnnnnnnnnnnnnnnnnnnnnnnnnnnnnnnnnnnnnnnnnnnnnnnnnnnnnnnnnnnnnnnnnnnnaccggggacaacaacgttcccgctgatacgggagcctgcaCCAATAAGcgtgttcatggagcgacgctcaatctttgccatGTATTCCATCCTGTTGAGACACTATCCTGCCCnnnnnnnnnnnnnnnnnnnnnnnnnnnnnnnnnnnnnnnnnnnnnnnnnnnnnnnnnnnnnnnnnnnnnnnnnnnnnnnnnnnnnnnnnnnnnnnnnnnnnnnnnnnnnnnnnnnntgtnnnnnnnnnnnnnnnnnnnnnnnnnnnnnnnnnnnnnnnnnnnnnnnnnnnnnnnnnnnnnnnnnnnnnnnnnnnnnnnnnnnnnnnnnnnnnnnnnnnnnnnnnnnnnnnnnnnnnnnnnnnnnnnnnnnnnnnnnnnnnnnnnnnnnnnnnnnnnnnnnnnnnnnnnnnnnnnnnnnnnnnnnnnnnnnnnnnnnntaccactgccctgtaaatggatgtcggttcttgccaaatgcCATGGAAATGTGACCTACGCCTCCCTTTATGGGCCACAGGCTGGGAATGTCAGAAAAAAGTGCTgtttgcgaattgagcaactttcctatctcccgtactggctgcacagnnnnnnnnnnnnnnnnnnnnnnnnnnnnnnnnnnnnnnnnnnnnnnnnnnnnnnnnnnnnnnnnnnnnnnnnnnnnnnnnnnnnnnnnnnnnnnnnnnnnnnnnnnnnnnnnnnnnnnnnnnnnnnnnnnnnnnnnnnnnnnnnnnnttgtgtcggatacacttcgtactggtttttctgccccagattGGAGCCCACTGAGGCCAACCTGAGAGAAAGTTTGGGAAAGGGAAGTCTGGAACGCCTTAGAAGTGCgaataagttctgctgcttcttggcgcaggagtgcaaggtgcacnnnnnnnnnnnnnnnnnnnnnnnnnnnnnnnnnnnnnnnnnnnnnnnnnnnnnnnnnnnnnnnnnnnnnnnnnnnnnnngaagccaacacgccaacaccgcacagaagaacgcacaagagcgtgcgagcggtagtacagaaagnccgggccgcacactgagagtgtggtactaacatgacggtgagttctgcacggcctgcggcgcccaggcgnnnnnnnnnnnnnnnnnnnnnnnnnnnnnnnnnnnnnnnnnnnnnnnnnnnnnnnnnnnnnnnnnnnnnnnnnnnnnnnnnnnnnnnnnnnnnnnnnnnnnnnnnnnnnnnnnnnnnnnnnnnnnnnnnnnnnnnnnnnnnnnnnnnnnnnnnnnnnnnnnnnnnnnnnnnngtctgagcacgaggacgcgggcacccaacccaagcttcggttctacttgctcttttctttaaagaggaccaagagggcacacgagnnnnnnnnnnnnnnnnnnnnnnnnnnnnnnnnnnnnnnnnnnnnnnnnnnnnnnnnnnnnnnnnnnnnnnnnnnnnnnnngctgattgttgatatcagccaacgttttgcgcagggcaGGGTTCTGCGCGTAGTAGTACTGCTCGAGCTCCGCTGCAGTAAGCCCAGAGAGCTCGTGGctcatGTAATGGAACCACTCGTTATCTGCCTCGCTACGTACCTCGTGCTTGATGCACCAGTAGTCCGGCAACACCGGGGGCTGTTTTTCTCccacgaatactttgtaattgaagacagccacgcggatgtcgccacgcgcaaggcccttttgcatannnnnnnnnnnnnnnTAGTTGATGGTTGCGCCTGAATCGAAGATATCATCTACGATGAGCACCTTATCCCCGACGCGTAGGTACTCAGGAGGGTAGGTCCAGCCATCTACGCTGATGACGCGCCGCTTACGCAAATCACAGTGCGAGTGAGCAACTACCGCTGCGTACAGGATaggaggctctgCCTTGTACGCGATGGTTAAATACTCATTGAGCACGTTACCCAGATATACTCCACCCCGTATGGGGACGTACATAACcgttggcacgaacctgtctgccacgatgcgccgggccataccgaaaccctcatcacggatcacattGTACGGAAnnnnnnnnnnnnnnacgttagcctctcctgcacgAGCACGAAAACACCCTACATCTAATGCTTTTTTAGCATCATGGCAAGCTCTTTTTCTATTCGTGTCGTGGCCTGGAACTGTCTTTGTTGAAAGCTTCGCCTGAATATTTTATGCTCCTGCGCGAGGGCCCCcgtgatagaaaagttggaagnnnnnnnnnnnnnnnnnnnnnnnnnnnnnnnnnnnnnnnnnnnnnnnnnnnnnnnCTCTTCCACTCAGAGTtatcgtgaacgTATGCGCGATCACGCCTATCTTTCCAGACTGATGGAAGAGTATGATCGCTATTTGCTTACTGAGAAGCAGTTGGAAGACGCGCACGTTCTCATCCAAGATGAGTCGGATGCTGATTTTAAGGACGTTATTCGGCAAGAGATCCGTACACTTGAAGCTGCACTGCACACGAGTCAAAAGCGACTAAAGACGCTGCTTATTCCCCCCGACCCTTTGCAAGAGAAGAATATTATCATGGAAATTCGCGGCGGTACCGGCGGTGATGAAGCAGCGCTCTTTGCTGCAGATCTATTTAGAATGTACACGCACTACGCTGAGTCAAAACAATGGCGCTATGAAGTCCTTGCAGTGAGCGAAACAGAGTTGGGAGGATTTAAGGAAATTACGTTCTCTATCTCGGGGCGCGATGTGTATGGCAGTTTACGTTATGAATCGGGTGTGCATCGCGTTCAACGTGTCCCTAGCACTGAAGCGTCGgGGCGCATCCATACCAGTGCGGTTACCGTTGCAGTGCTGCCTGAGATGGAAGAGACTGAAGTGGACATTCGTGCTGAGGACGTGCGTGTTGATGTCATGCGTGCAAGTGGTCCTGGTGGGCAGTGTGTCAACACCACTGATTCTGCGGTGCGTCTTACACATCTACCTACGGGCATTGTCGTTGTCTGTCAGGACGAGAAGAGTCAAATCAAAAACAAAGCCAAGGCCATGCGTGTATtgcgcagcagagtgtnnnnnnnnnnnnnnnnnnnnnnnnnnnnnnnnnnnnnnnnnnnnnnnnnnnnnnnnnnnnnnnnnnnnnnnnnnnnnnnnnnnnnnnnnnnnnnnnnnnnnnnnnnnnnnnnnnnnnnnnnnnnnnnnnnnnnnnnnnnnnnnnnnnnnnnnnnnnnnnnnnnnnnnnnnnnnnnnnnnnnnnnnnnnnnnnnnnnnnnnnnnnnnnnnnnnnnnnnnnnnnnnnnnnnnnnnnnnnnngtacgattcgacaggcgcgnatgtacgcgcgagcgttgnttcnnnnnnnnnnnnnnnnnnnnnnnnnnnnnnnnnnnnnnnnnnnnnnnnnnnnnnnnnnnnnnnnnnnnnnnnnnnnnnnnnnnnnnnnnnnnnnnnnnnnnnnnnnnnnnnnnnnnnnnnnnnnnnnnnnnnnnnnnnnnnnnnnnnnnnnnnnnnnnnnnnnnnnnnnnnnnnnnnnnnnnnnnnnnnnnnnnnnnnnnnnnnnnnnnnnnnnnnnnnnnnnnnnnnnnnnnnnnnnnnnnnnnnnnnnnnnnnnnnnnnnnnnnnnnnnnnnnnnnnnnnnnnnnnnnnnnnnnnnnnnnnnnnnnnnnnnnnnnnnnnnnnnnnnnnnnnnnnnnnnnnnnnnnnnnnnnnnnnnnnnnnnnnnnnnnnnnnnnnnnnnnnnnnnnnnnnnnnnnnnnnnnnngcatatgttgcgtgcgcgtggcacgccaccTCTCTATGTCATTGCATCCGACATTTGCATGCGGGCCCTTGCCGTAGcgcggtataacgcgcgccgactcttggatgtnnnnnnnnnnnnnnnnnnnnnnnnnnnnnnnncagatgtgcgtgCTCCTATTCCGTTCTTTTCTCCTTCTGAAGGCACGGACGTGGTACAGGAGCGCGGGGTGTGCGTTCCGTATGATGTGATATGTGCAAATCCGCCTtacgtaccgagtgcgcangcgcgcgcgctgttgcaggacgggagaggggagcctctcggtgccttggatggGGGTGCAGATGGGCTAGACTTGGTTCGCGCATTCGCACACCACAGTGCCGCAGCGCTAAAGGAAGGCGGGTGCGTGTTTTGCGAGGTCGGCTCAAACCACGCACAACGTGCAGCGCGCATCTTCCAGGCAGCAGGGTTTGCCACGGTGAAAATTTCAAAAGATCTCTCCGGGAAAGAGCGCCTGATTAGCGGGATACTGCGCTCGCAGTCTAGAGCtgtaacagcgccgagtggctagggtgaaacacggcgactgagtggttatcctggcgtttgcaggtggatgtccgcgccgcgttggccgataggctgagtacatgaaggagttagagatcatccnnnnnnnnnnnnnnnnnnnnnnnnnnnnnnnnnnnnnnnnnnnnnnnnnnnnnnnnnnnnnnnnnnnnnnnnnnnnnnnnnnnnncccagctcatcgattccggttctacacggtgcccctcgtacaagggctcaaagcctaaattttcgcaacgaagattacccaaatacnnnnnnnnnnnnnnnnnnnnnnnnnnnnnnnnnnnagggatctgnTCCCCAATGACATAGTCCCCccacttaatttcttgttcgcatccttcgcgaatcatatcgcgaaataAGCGTACATTGCGTGCAGTGAACACCTGTGGCTCTTCCTTTTGCAGTTCTTGAATAATGGATCGAAAAAGCCACAGGTGTGTGTTTTCATCGCGGTTGATATAACGAATTTCCTGCACCGAGCCGGGCATCTTGTTATTACGCCCCAAGTTATaGAAGAACATAAAACCtgaatagaaataaattccttccaaaacataattcgcaattgctaccttcagcagtgcgagtacGCTTTTGTCATCTTGAAACTCGTTGTACAAGTTGCCAATGAATTTATTGCGCGCAAGCAGATGCTCGTCGTCCttccactggtataGAATGTCATTGCGTTCTTCGGGGGAGCAAATGGTGTCCAGCATGTAACTGTAACTCTGCGAATGCACAGCCTCTTGGAAAGCCTGAATGGTTAGGCACAGGTTAATCTCATTTGCGGTAATGTACTGACCAATATTTGGCAGATTCGCAGTCTGGATGCTATCTAGGAAAATAAGGAAGGAGAGGATTTTATCGTACGCGGTTTTCTCgatagcggatagattacgataatcttgcacgtcactgctcatgttGATTTCTTCGGGGATCCAGAAGttgttcattgcctgccgataccactcgctgacccagggataCTTCATATTGTTAAAGTCGTTGAGATTGGTAGTGTTCCCCCCGACCATGCGTCGCTTATGAAGTTCAATGTCTCCTGCCTCATTAAACagcgcgcgTCTTTGCAGTATCGTTGAACTTTCCATCATGATGAAACCTCCTCCTGAGTGCGGGCACAGGGTAGTATACTTGATGGACTTTGAATTCTCAATTGCGGCGGGCGTAAAAATGTCTGTCTGCAGGGCtctgtgtcttgtgtggtgGTCCCTGTTTTGTGGGTAGGTTTGGGGnAACCTAACAGAGACGTAGGAAATCCGTTATGCTCGAGCaCATGAAGCATGAACAAGCACGAAGTCAGCTATCACACGAGCCTCCTAAGCGGCGCCGAGCCTCTCTAACCGTCTGCGGCCTGCGTGCTGTGGAAAcgcttggcnnnnnnnnnnnnnnnnnnnnnnnnnnnnnnnnnnnnnnnnnnnnnnnnnnnnnnnnnnnnnnnnnnnnnnnnnnnnnnnnnnnnnnnnnnnnnnnnnnnnnnnnnnnnnnnnnnacgcaggaacttgagcgattgacgcaatccgttcaccatcagggggttgctgctaccatagacgagccgcgctTTCCAGCCGTGACTCATTCTCAGGTTGAATTTTGGGTACAACGGCGTGAGTTTGTTGTGTTACTCGATCGCGtaggagnngcccacaatctgggggcgATTATACGTAGTGCTGCTTTTTTTGGAGTGCACTCACTGGTGGTGAGTGActgtcgacagcaggcgcaggttacaagcgcaacatatcgggttgcgcagggaggaatggagTTTGTGCAATTGTTGCGCTGTAcaaatgcgcaggaagtatnngaaatgtgtgcaggtaaaatgacccgtgtgggagcctcccctcatgcgttcagatcgcttacacggctttcaaacatactctcgcctgaagaagcggtaatattaGTACTGGGAAACGAGGAGACAGGGCTTTCTGAGCATTTGACTGCGCATTGCGATCATCTCTGTCGGATTGCAGGCAGTGGTCAGGTGGAAAGTCTAAATGTTGCGCAAGCGGGTGCGCTTTTTTTGTCCACTATCGTACAGTTGCGTCAATCTCCTCAGGACTACACGCAGGGACATCGGGCCACGCCACGTGCACAAGAGCGTGTGCACCGCTGTGGGCAATTAGAGGAAAAGGGGCAGAAAAATGGAGCACGTGTTCTTATTCCCCGCTCGGGGGCGCGTGCCAATTCCCGTGAAAGTTGAGAGTAGGGAAAGTAGACGGGTGTGAGGTATGGAACctacagcgcaattggttttgcagtgcgtgnnnnnnnnnnnnnnnnnnnnnnnnnnnnnnnnnnnnnnnnnnnnnnnnnnnnnnnnnnnnnnnnnnnnnnnnnnnnnnnnnnnnnnnnnnnnnnnnnnnnnnnnnnnnnnnnnnnnnnnnnnnnnnnnnnnnnnnnnnnnncatgaatcagatccgcctgtttgcccagagtgcgctTGTGAGCGTCATGGGTATGGGGATGGTTTTTGCCTTCCTCCTTTTGCTCATATGCGTTGTGCGCTGTGTGGGCGCGCTTGTCTCTTCTTTCGGCTGGGATCGCGGTCCTGACGAAGGTGTCGGCGCTGCAGTCCCTGCAGGAGGAGCACTCGCCGCGGCTATCGCAGTCGCCGTTCATGAGAAGGCaaggagtacttcatGAGTACCCCGGTTCGCATTAGCGAAATGGTCCTACGTGATGCGCATCAGTCTTTGCACGCTACGCGCATGACTACCGAAGACATGCTCCCTATTTGTGACAAGCTAGATCGCGTTGGGTATTGGAGTTTGGAGGCGTGGGGAGGCGCCACGTACGACGCCTGCATTCGCTTTCTAAATGAGGATCCCTGGGAGCGTTTGCGTGCTCTCAAAGCTCGGTTACCTAAGACCCCTATTATGATGCTTTTGCGTGGGCAAAACTTGCTAGGCTACCGGCATTACGCGGATGACGTTGTAGATGCGTTCGTAGAGGCCGCTGCGCGCAACGGCGTTGATGTGTTCCGCATCTTCGATGCACTTAATGACCCACGTAACCTCAGTCAGGCTGCGCGTGCTGCAAAGAAAACAGGCAAGCATGTGCAGATGGCTATCTCTTACGCTACCACACCCTATCATACCGCAGAGAAGTACGTAGAGTTAGCAAAGGAGTATGCGCGCTTCGGTGCGGATTCTATTTGCATTAAGGATATGTCGGGGTTGCTGACCCCGTACGGGGCGTACGATCTGGTTTCTGCCATTAAAAAGAGTGTCGATTTGCCCGTTGAGTTGCACACCCACGCCACTACTGGTATGTCTGTTGCAACCCTGGTGAAGGCGGCAGAAGCAGGTGTTGATGTAATTGACaCTGCCATTGCTTCTATGTCCATGGGTACTTCCCACAGCCCTACAGAGACTTTAGTGGAAATCCTACGGCACACGGGCCGTGACTCAGGGCTCGACATAAATCTCCTGCTAGAAATAGCAGCCTACTTCCGTCAGGTACGGAAGTGCTATGCCCAGTTTGAGTCTAGTTTTCTGGGTGCAGACACGCGTATCCTCGTGTCCCAGGTGCCTGGGGGtatgctttccaatttagaaaaccagttgcgtgagcagggagccctggataagatggaccaggttcttaaggaaattcccctggtacaGAAGGACTGCGGTTATATCCcgcttgtgactcctacgagtcagattgtaggtacgcagtcagtattgaacgtgctgtttggccggtaccaccggcttactgctgagannnnnnnnnnnnnnnnnnnnnnnnnnnnnnnnnnnnnnnnnnnnnnnnnnnnnnnnnnnnnnnnnnnnnnnnnnnnnnnnnnnnnnnnnnnnnnnnnnnnnnnnnnnnnnnnnnnnnnnnnnnnnnnnnnnnnnnnnnnnnnnnnnnnnnnngaggcgcgcgccgcaggcgcacaggataCCATTGAGGATGTGCTCACGTATGCTATGTTTCCCAAGATCGCTCCCACATTCTTTGCTTCCCGTGCGCAAGGGCCTATTTCGTTCAGAGGAAAGGGGCAGGGGCAAAAACAGAAGGGTGAGAGTGCAGGGTCGGTAGCTTCTTATGTGGCTACCGTAAATGGTACTGCGTACACAGTTGTGCAGGAAGGCGCTGTTCTCCGGGTAAATGGTACTCCCTACACCGTTAGGGTTGAGGCAGGCCCGTCCGTTGCTTCGGGTACGTCGCAGGGTACCGTGACTACGGCAAAGGTTGGGGCGTGTACTACGCTACCCGCGCCGGTCGCAGGTAGCGTAGTTAAACacaccGTGCAAGATGGAGCTACGGtaaattcgggggagacggtgatcatggtggagtccatgaagatggaacnnnnnnnnnnnnnnnnnnnnnnnnnnnnnnnnnnnnnnnnnnnnnnnnnnnnnnnnnnnnnnnnnnnnnnnnnnnnnnnnnnnnnnnnnnnnnnnnnnnnnnnnnnnngaATACGCGTTTACCCCTTCGAGTACTCCAGTGCGTGTTGGTGGGATTGCTTGTGTGCGGGCCCCTGTGTGCAGCTACGCGCCGCCCGGTACGTGCTTCTGCGCCGGTGCCTATGGTACAGAGTTGTAAAGACACGGGGGCACGATGTGCGCCGGCGtcgtccatgcgtgaggacatgcgtgcgtcacacggagnnnnnnnnnnnnnnnnnnnnnnnnnnnnnnnnnnnnnnnnnnnnnnnnnnnnnnnnnnnnnnnnnnnnnnnnnnnnnnnnnnnnnnnnnnnnnnnnnnnnnnnnnnnnnnnnnnnnnnnnnnnnnnnnnnnnnnnnnnnnnnnnnnnnnnnnnnnnnnnnnnnnnnnnnnnnnnnnnnnnnnnnnnnnnnnnnnnnnnnnnnnctgctgctcattcnnnnnnnnnnnnnnnnnnnnnnnnnnnnnnnnnnnnnnnnnnnnnnnnnnnnnnnnnnnGGTATGCTCAAACTCATTTACGATGCTGGGGTGGGGAATGAGTTTTTCCCTATGCTCATTTTTATGGGTATCGGTGCACttaccgattTTGGACCACTGATTGCGAATCCTAAAATGGCAGTCCTTGGTGCCGCTGCCCAGTTAGGGGtgttccttactctctttggggttgcagnnnnnnnnnnnnnnnnnnnnnnnnnnnnnnnnnnnnnnnnnnnnnnnnnnnnnnnnnnnnnnnnnnnnnnnnnnnnnnnnnnnnnnnnnnnnnnnnnnnnnnnnnnnnnnnnnnnnnnnnnnnnnnnnnnnnnnnnnnnnnnnnnnnnnnnnnnnnnnnnnnnnnnnnnnnnnnnnnnnnnnnnnnnnnnnnnnnnnnnnnnnnnnnnnnnnnnnnnnnnnnnnnnnnnnnnnnnnnnnnnnnnnnnnCGGATAGAGAGAGTACTCTTTCCGCTTGTCTTGCTCTTGCTCTCGGTGCTGCTCATTCCTGCGGCTTCCCCACTCATCGGTATGATCGCCTTCGGGAACTTTGTTAAGGAATGCGGTGTTGTGGAGCGGTTGTCTAAGACGATGGCTAACGAGCTTTTGAACATCGTGTCGATCTTGCTGTCTTTGGGTGTTGGTTCTCAGATGACACCCGATAAGAttATGAACCCCAATGCCTTGGGCATTATCGTGTTGggactcgttgccttttctgtcgcaaccgcannnnnnnnnnnnnnnnnnnnnnnnnnnnnnnnnnnnnnnnnnnnnnnnnnnnnnnnnnnnnnnnnnnnnnnnnnnnnnnnnnnnnTTCctatggccgcgcgngtntctaataaggtggggcnagaggaggatcCTTCTAACTTCTTGCTTATGCACGCGATGGGTCCTaACGTGGCTGGTGTCATTGGGACCGCGATAGCCGCAGGGGTGTTCATCTCGGCCTACGGAGGGTAGGGaggaagagtaaccgcggggttttgccgcttaggtaccctttcctccgtgcgcgggcacaccctctcaggtggctaggggcttttgcagacgaagcgggtaaagctcgcttggaannnnnnnnnnnnnnnnnnnnnnnnnnnnnnnnnnnnnnnnnnnnnnnnnnnnnnnnnnnnnnnnnnnnnnnnnnnnnnnnnnnnnnnnnnnnnnnnnnnnnnnnnnnnnnnnnnnnnnnnnnnnnnnnnnnnnnnnnnnnnnnnnnnnnnnnnnnnnnnnnnnnnnnnnnnnnnnnnnnnnnnnnnnnnnnnnnnnnnnnnnnnnnnnnnnnnnnnnnnnnnnnnnnnnnnnnnnnnnnnnnnnnnnnnnnnnnnnnggggcgcaaagggattGTCTGCTACGATAAGTCCCAAGTAGTCGACAAAAATAATCTggatcttttcttgtacgcataatcgacgagccacggcacggagatccagtagcttcatgtttngcacgtccacgatgtaaagtgggGCGTCGTACatctctcctgcggcgttttggatgcgcccgaaatcggaaagttgtaaaagccctttgcgcaggttcgtcgcggatactcctgactctgcagcgataagtcgctgcatcagaagcaaattagacatttccagagaaaaaaaggnnnnnnnnnnnnnnnnnnnnnnnnnnnnnnnnnnnnnnnnnnnnnnnnnnnnnnnnnnnnnnnnnnnnnnnnnnnnnnnnnnnnnnnnnnnnnnnnnnnnnnnnnnnnnnnnnnnnnnnnnnnnnnnnnnnnnnnnnnnnnnnnnnnnnnnnnnnnnnnnnnnnnnnnnnnnnnnnnnnnnnnnnnnnnnnnnnnnnnnnnnnnnnnnnnnnnnnnnnnnnnnnnnnnnnnnnnnnnnnnctctcCTTGCATTTGTTAGGTCATAGATTTCCCTTTGTGCTGTTTCGAGTACGATGTTGCCCGACACGGTGTCATTGAATGCCTCTGCGGTGATAATGCGGGCTACTTTTAGTAGCGACCGGCGCATGGCagcgtcgcaaacgatgcGTGTGTAGTattcaacattcgcggcgcttgggaccgcatcggtgagagaggcaacatacgcgctgccaccgncgaaatcgagcgcctcacaggagcgcaggtgctcgctgagcacgaggatatcagggcgttgacctaaatcnnnnnnnnnnnnnnnnnnnnnnnnnnnnnnnnnnnnnnnnnnnnnnnnnnnnnnnnnnnnnnnnnnnnnnnnnnnnnnnnnnnnnnnnnnnnnnnnnnnnnnnngaacagcgccgagcacagcccgctcggcctctaggttatgagggggaatttttcccttgagttcctgagtgGGATTAGGCATGCCCGGCACAGAACCTCCTCCGAGGAATACTCAGAGGAGGGAAGGTGGTGAAAAGACAGTCCCCCCCCTCTCCGTGGAAACCCTCTAACGAGGCAAAGGGTTGAGGCCCAACTGCCAAGAACTGTCTCCCTTCCGTGGAATCCAGCCCCnnnnnnnnnnnnnnnnnnnnnnnnnnnnnnnnnnnnnnnnnnnnnnnnnnnnnnnnnnnnnnnnnnnnnnnnnnnnnnnnnnnnnnnnnnnnnnnnnnnnnnnnnnnnnnnnnntggtgacaggaacaacagcacatatttnnnnnnnnnnnnnnnnnnnnnnnnnnnnnnnnnnnnnnnntTTCAGAGTAAGACCAGGGACCTCCACACGCTTGCGCTCAACCTCAAATCCCATGCACGCAAGTTGTTCTGCAACGGTATGACTcgtgacagCGCCGTACAACTTACCGTTGGTACCGGCGGGCATGGCAATAACCACAGGCTGAGCCTCTAAACGAGCCTTAAGATTTGCGGCATCTTGTCGCTTGAGAGACTTTCGCATCTCTATGnCCTGTTGTCGCTGTTTGAAGCGAGCCACGGTaaaacgattatgaggaacagcaaggtttcgagggtagaggtaattacgaaaatagcctgcggcgacctctttcacatcaccttcttcaccaaggatttttnnnnnnnnnnnnnnnnnnnnnnnnnnnnnnnnnnnnnnnnnnnnnnnnnnnnnnnnnnnnnnnnnnnnnnnnnnnnnnnnnnnnnnnnnnnnnnnnnnnnnnnnnnnnnnnnnnnnnnnnnnnnnnnnnnnnnnnnnnnnnnnnnnnnnnnnnnnnnnnnnnnnnnnnnnnnnnnnnnnnnnnnnnnnnnnnnnnnnnnnnnnnnnnnnnnnnnnnnnnnnnnnnnnnnnnnnnnnnnnnnnnnnnnnnnnnnnnnnnnnnnnnnnnnnnagcgtgtccggatccttataatcagctaaaagcttctgngtgcaaaaacngcatactttctttcgatagaattgtctgtttttcttgggtgcgctcnnnnnnnnnnnnnnnnnnnnnnnnnnnnnnnnnnnnnnnnnnnnnnnnnnnnnnnnnnnnnnnnnnnnnnnnnnnnnnnnnnnnnnnnnnnnnnnnnnnnnnnnnnnnnnnnnnnnnnnnnnnnnnnnnnnnnnnnnnnnnnnnnnnnnnnnnnnnnnnnnnnnnnnnnnnnnnnnnnnnnnnnnnnnnnnnnnnnnnnnnnnnnnnnnnnnnnnnnnnnnnnnnnnnnnnnnnnnnnnnnnnnnnnnnnnnnnnnnnnnnnnnnnnnnnnnnnnnnnnnnnnnnnnnnnnnnnnnnnnnnnnnnnnnnnnnnnnnnnnnnnnnnnnnnnnnnnnnnnnnnnnnnnnnnnnnnnnnnnnnnnnnnnnnnnnnnnnnnnnnnnnnnnnnnnnnnnnnnnnnnnnnnnnnnnnnnnnnnnnnnnnnnnnnnnnnnnnnnnnnnnnnnnnnnnnnnnnnnnnnnnnnnnnnnnnnnnnnnnnnnnnnnnnnnnnnnnnnnnnnnnnnnnnnnnnnnnnnnnnnnnnnnnnnnnnnnnnnnnnnnnnnnnnnnnnnnnnnnnnnnnnnnnnnnnnnnnnnnnnnnnnnnnnnnnnnnnnnnnnnnnnnnnnnnnnnnnnnnnnnnnnnnnnnnngagcaccacctgcagaagtgtacttgagctccgcatcacgcgtaagccgaccaacgagcactacatgattgacgtctgccaTATCTTCTCTCAGGAATCGACACGCACAAACAAGTGCGTGAGTAAGTCGTGTCGCAATCTGAGCTTATGATnnnnnnnnnnnnnnnnnnnnnnnnnnnnnnnnnnnnnnnnnnnnnnnnnnnnnnnnnnnnnnnnnnnnnnnnnnnnATACGCAagttcccgctctccaatatggtcttcgcgGGCGATGACTGCGTCGTTTTCCTGTAGGAGGGCACGAAnnnnnnnnnnnnnnnnnnnnnnnnnnnnnnnnnnnnnnnnnnnnnnnnnnnnnnnnnnnnnnnnnnnnntaataactcctatggacggctcaccttatcaggctgcactagacnnnCCCGACAAGGAGGAGTAGCGTGCGCAGAACGCGCGGCGAGACGCCAGCGTAGCGTAAATCTGTGTGCCGCATCAAGTCTTTTGTCCTTTTATCCGATGCtgccgnnnnnnnnnnnnnnnnnnnnnnnnnnnnnnnnnnnnnnnnnnnnnnnnnnnnnnnnnnnnnnnnnnnnnnnnnnnnnnnnnnnnnnnnnnnnnnnnnnnnnnnnnnnnnnnnnnnnnnnnnnnnnnnnnnnnnnnnnnnnnnnnnnnnnnnnnnnnnnnnnnnatCACCTAGCACGTTCTATTGATGTCCTGTATGAGGGGCTTCAGCTTAACTTAGATGAGTGCCTTTATGCGGAGAAAGTTGTGTATGACGTGCGnTTTTTTGACCATGCGTTGCAAAAGTTGTGCGCGCATATCGATCGCCAGTCTCACTTCCCCGATTACTTACCAATTCTTCATTGCCTATTCTCCTGCGGTGCACGATTCTTGAACTTATTGAATTTTCTTATTCATCGTGCCTCTCCTGTGACTGCGCAGGTTGAGTTtnnnnnnnnnnnnnnnnnnnnnnnnnnnnnnnnnnnnnnnnnnnnnnnnnnnnnnnnnnnnnnnnnnnnnnnnnnnnnnnnnnnnnnnnnnnnnnnnnnnnnnnnnnnnnnnnnnnnnnnnnnnnnnnnnnnnnnnnnnnnnnnnnnnnnnnnnnnnnnnnnnnnnnnnnnnnnnnnnnnnnnnnnnnnnnnnnnnnnnnnnnnnnnnnnnnnnnnnnnnnnnnnnnnnnnnnnnnnnnnnnnnnnnnnnnnnnnnnnnnnnnnnnnnnnnnnnnnnnnnnnnnnnnnnnnnnnnnnnnnnnnnnnnnnnnnnnnnnnnnnnnnnnnnnnnnnnnnnnnnnnnnnnnnnnnnnnnnnnnnnnnnnnnnnnnnnnnnnnnnnnnnnnnnnnnnnnnnnnnnnnnnnnnnnnnnnnnnnnnnnnnnnnnnnnnnnnnnnnnnnnnnnnnnnnnnnnnnnnnnnnnnnnnnnnnnnnnnnnnnnnnnnnnnnnnnnnnnnnnnnnnnnnnnnnnnnnnnnnnnntttgtttatcttgatccaccttttccctatcgcnttcacgctgagctgttgcagcggctttctcgtgcgtcattgtgtagagaaggaagcgtggtgatggtgcaccgaccaagagagaaaaaacttgcggataaaatcgattcacttgtgcnnnnnnnnnnnnnnnnnnnnnnnnnnnnnnnnnnnnnnnnnnnnnnnnnnnnnnnnnnnnnnnnnnnnnnnnnnnnnnnnnnnnnnnnnnnnnnnnnnnnnnnnnnnnnnnnnnnnnnnnnnnnnnnnnnnnnnnnnnnnnnnnnnnnnnnnnnnnnggtatacagcgccctgtttccactctctccgatcgcgaccgggcgctgctaaacaggcggggcaatgcatacctgaatgaggggannnnnnnnnnnnnnnnnnnnnnnnnnnnnnnnnnnnnnnnnnnnnnnnnnnnnnnnnnnnnnnnnnnnnnnnnnnnnnnnnnnnnnnnnnnnnnnnnnnnnnnnnnnnnnnnnnnnnnnnnnnnnnnnnnnnnnnnnnnnnnnnnnnnnnnnnnnnnnnnnnnnnnnnnnnnnnnnnnnnnnnnnnnnnnnnnnnnnnnnnnnnnnnnnnnnnnnnnnnnnnnnnnnnnnnnnnnnnnnnnnnnnnnnnnnnnnnnnnnnnnnnnnnnnnnnnnnnnnnnnnnnnnnnnnnnnnnnnnnnnnnnnnnnnnnnnnnnnnnnnnnnnnnnnnnnnnnnnnnnnnnnnnnnnnnnnnnnnnnnnnnnnnnnnnnnnnnnnnnnnnnnnnnnnnnnnnnnnnnnnnnnnnnnnnnnnnnnnnnnnTACCAAGAGGCATCTGACTATTACACGAGGTGCTTACAGCATTACCCTCGCAACAGCTATGCGCTCTTTGGTCTTGCGGACTGTTATAAAAACATGCGTCGGTACGTGAAGGCAGTGGAAATGTGGCAGCAGTACCTGGAGCAGGATAGCCACAACnnnnnnnnnnnnnnnnnnnnnnnnnnnnnnnnnnnnnnaatacatgattttcaaaactcgagaaacctttactcccaggttatcgccctggatgaacataattcctacgcgctaattgggcttgctcannnnnnnnnnnnnnnnnnnnnnnnnnnnnnnnnnnnnnnnnnnnnnnnnnnnnnnnnnnnnnnnnnnnnnnnnnnnGTGGATATCCGTGTACTCACCTCTATCGGGAATTGTTATCGTAAAATGAAACTCTTTAGTnnnnnnnnnnnnnnnnnnnnnnnnnnnnnnnnnnnnnnnnnnnnnnnnnnnnnnnnnnnnnnnnnnnnnnnnnnnnnnnnnnnnnnnnnnnnnnnnnnnnnnnnnnnnnnnnnnnnnnnnnnnnnnnnnnnnnnnnnnnnnnnnnnnnnnnnnnnnnnnnnnnnnnnnnnnnnnnnnnnnnnnnnnnnnnnnnnnnnnnnnnnnnnnnnnnnnnnnnnnnnnnnnnnnnnnnnnnnnnnnnnnnnnnnnnnnnnnnnnnnnnnnnnnnnnnnnnnnnnTTGTAAACTCCAGGGGAGATACGAAGAGGCGGTTGTGAGTCTTGATCGACTCGTGCAGCTTGATCGGAAAAACTATCGCGTATATGTGGagcttgcagactgctaccgcaagctcgggcagnnnnnnnnnnnnnnnnnnnnnnnnnnnnnnnnnnnnnnnnnnnnnnnnnnnnnnnnnnnnnnnnnnnnnnnnnnnnnnnnnnnnnnnnnnnnnnnnnnnnnnnnnnCCTTTTTCTTCCTGGTAAGCTTGGGTATGGGTGGCGTTTGCCAAGTGTGAACGGCTCGTTGCGCAGGTGTGGCTGCGCGATGCCTGTCGATTCggcgctttttgtgtggtggtcggggtcctcctcttcgtcggaggtggtgcaggactttgcgacgaGGGGGTGGAGCGTATATGGAGTGGTGCTGCGCCCTTTCGGGGCTTTTGCCAGAAGAAATCCAGAAGGTGTGTGCGTTTGCTGAGCGCTTTCGTGGGGTGCAGGTGTTCAGATGGATTGCCGCAGGGTGCACTGACTTCCATGCGATGAGTGatctctnnnnnnnnnnnnnnnnnnnnnnnnnnnnnnnnnnnnnnnnnnnnnnnnntCGTGTCTATAccacgctgcgtgatgtggatggtacgctcaagctgggtattgaactgaaagataaacggcgcgtagaggcagtcttactcgtcgatcaagtctcgcgtaagactgcttgtctatcctgtcaagtcggctGCCCTATGGCGtnnnnnnnnnnnnnnnnnggccagttgggtttcgcgcgaaacctttctgcctcagagatcgtcgagcagttccttcatctggaacgatgtgtcggtacattggataatgttgtGTTTATGGGAATGGGTGAGCCCATGCTCAATCTGGATGCGGTGTGTAGAGCTATTGAGATACTGTCTCATCCACAGGGTCGTGACCTATCTGAAAAACGTATTACTATTTCTACGTCTGGACATTGCCGTGGTATTTATTCGCTTGCTGACCGCGCACTGCAGGTTCGCTTGGCnnnnnnnnnnnnnnCCGCGAATGcaccgttgcgcgcacgccncatgccacgtgctgcgcacgacagtttagcnnnnnnnnnnnnnnnnnnnnnnnnnnnnnnnnnnnnnnnnnnnnnnnnnnnnnnnnnnnnnnnnnnnnnnnnnnnnnnnnnnnnnnnnnnnnnnnnnnnnnnnnnnnnnnnnnnnnnnnnnnnnnnnnnnnnnnnnnnnnnnnnnnnnnnnnnnnnnnnnnnnnnnnnnnnntcaatccactttgaaacacctcgggaagtggaggttgcgcattttgaggcgctTCTCATGCGCGCCCGCATCCCCGTGACACGCCGCTATCagcgtgggaatggcattggaggcgcatgcggacaactaggtaaaacagccggcGTGTAACTCTTTCGCTCCGTTTGTAGATGTTTGTACGTGTGGCCATGCTCGTTCCTGTTTTTCAGGAAAGTTTCTTTGAGGCAGCCAGCGCTTTTGCCTTGCTCGAGTAGAGCTGTACGCAGTCCCAGGGTAACCAACCTCTTTCTACGTTcacccacagcgctgactctttatcnnnnnnnnnnnnnnnnnnnnnnnnnnnnnnnnnnnnnnnnnnnnnnnnnnnnnncgcaatGACGATCCCCGTGACACCTGGTTTGTCACGAAGCAAAACATAAGCCTTGGTGATTACTGCGAAGCGACGCGCTTCTCCAATTGGAGAGCTCGAGGGGAACCGTACATCTTCCAGTGAATTTTTCCGCACACAGGCGGAAGTGCTGCTGAGTACGAGCAGTACCaacgcccATGTGCGGCGCTGCAGCACGGGGATACCGCGTACCTTCATAGAAAAAGCGTCGGCGCAAACAGAAGGTGTAGAATGCTCGCTGCGAGAGAAAACAATCCCAAGGGTTTGGCATACTGCACCagcgtgcgcggaataggtgacggcttggaannnnnnnnnnnnnnnnnnnnnnnnnnnnnnnnnnnnnnnnnnnnnnnnnnnnnnnnnnntaTGAGATCCCCAAGCACGGGAAGTTGGGCACCGATAGGATTAACGCACTTGCACactgcggcaattcccgagagaagcgccaGCACTAGAGAGAAAGTCGCGTCATACAGAAAGGGACAGTCGTGCGCACTCTCCTCTTGGGAGTnnnnnnnnnnnnnnnnnnnnnnnnnnnnnnnnnnnnnnnnnnnnnnnnnnnngtaaaacggtaacangatGGTCTCCTCTTACATGTGGCATGACATTGCCATATTTGAATATGCACTCAGGATGTTTGTTCAGTGAAACACAGAGCATCTTTACACATATCCACCACGATAGTTGAGCCGCCTCTGAATCGGCCACTGAGAAtctcacgCGCTAGGGCATtttCCAATTCcgtttggattgcacgcttcagtggtcgtgctccgaaagtgtcgtcgtatccgcgctccgcaagataggctttcgccgcgtcacgcacacgaagttttatatgtcgactttccaaacgctccactaccatcnnnnnnnnnnnnnnnnnnnnnnGGCGAATATGTTTCCGTGTGAGACGCTTAAAAATTAACACTTCGTCAATCCGGTTTAAGAATTCTGGGCGAAAGTATGTGTGCAGTAATCCCCGTATCTGCTCTGGTAGAGTTTGTTCTTCTGTAGATTGTGTCTCGGGTACAGGCAAGTCCGACGTGTGTGTGCGCGACTCGCGTGCAGAAAGAATATGCTCTGATCCGATATTGCTGGTCATGATGATGATCGTGTTGCGGAAATCCACCACnnnnnnnnnnnnnnnnnnnnnnnnnnnnnnnnnnnnnnnnnnnnnnnnnnnnnnnnnnnnnnnnnnnnnnnnnnnnnnnnnnnnnnnnnnnnnnnnnnnnnnnnnnnnnnnnnnnnnnnnnnnnnnnnnnnnnnnnnnnnnnnnnnnnnnnnnnnnnnnnnnnnnnnnnnnnnnnnnnnnnnnnnnnnnnnnnnnnnnnnnnnnnnnnnnnnnnnnnnnnnnnnnnnnnnnnnnnnnnnnnnnnnnnnnnnnnnnnnnnnnnnnnnnnnnnnnnnnnnnnnnnnnnnnnnnnnnnnnnnnnnnnnnnnnnnnnnnnnnnnnnnnnnnnnnnnnnnnnnnnnnnnnnnnnnnnnnnnnnnnnnnnnnnnnnnnnnnnnnnnnnnnnnnnnnnnnnnnnnGCCCTACCACACGTTGCATGAGTACTGACTCAAGCTGCAGATATTTCTGTTGCTCGCTTGCCATCATTTTGGATACCGGAATTCCGGTCCACATAGAAATAATTTTCGCAATGTCCTCTTCACACACTTCCTCGCGCAAGAGCTGTCCTTCnnnnnnnnnnnnnnnnnnnnnnnnnnnnnnnnnnnnnnnnnnnnnnnnnnnnnnnnnnnnnnnnnnnnnnnnnnnnnnnnnnnnnnnnnnnnnnnnnnnnnnnnnnnnnnnnnnnnnnnnnnnnnnnnnnnnnnnnnnnnnnnnnnnnnnnnnnnnnnnnnnnnnnnnnnnnnnnnnnnnnnnnnnnnnnnnnnnnnnnnnnnnnnnnnnnnnnnnnnnnnnnnnnnnnnnnnnnnnnnnnnnnnnnnnnnnnnccaaacgttcctttgaagccggatcactttctttaaggagagaggccttttcgatattcagctgtaatatcttgcgctccacctggtctagctcaacaggnnnnnnnnnnnnnnnnnnnnnnnnnnnnnnnnnnnnnnnnnnnnnnnnnnnnnnnnnnnnnnnnnnnnnnnnnnnnnnnnnnnnnnnnnnnnnnnnnnnnnnnnnnnnnnnnnnnnnnnnnnnnnnnnnnnnnnnnnnnnnnnnnnnnnnnnnnnnnnnnnnnnnnnnnnnnnnnnnnnnnGCAATGGTGTCCTCCACCGTAGGCTGTACGCAGTACnnnnnnnnnnngcggcgttcgagcgCTGCGTCCTTTTCGATATATTTGCGATATTCGTTGAGCGTGGTTGCGCCGATTGAACGCAATTCACCGCGCGCAAGCGCAGGTTTcagaaggttcgacgcatccatagatccctcacttgcgccggcgcctacgagnnnnnnnnnnnnnnnnnnnnnnnnnnnnnnnnnnnnnnnnnnnnnnnnnnnnnnnnnnnnnnnnnnnnnnnnnnnnnnnnnnnnnnnnnnnnnnnnnnnnnnnnnnnnnnnnnnnnnnnnnnnnnnnnnnnnnnnnnnnnnnnnnnnnnnnnnnnnnnnnnnnnnnnnnnnnnnnnnnnnnnnnnnnnnnnnnnnnnnnnnnnnnnnnnnnnnnnnnnnnnnnnnnnnnnnnnnnnnnnnnnnnnnnnnnnnnnnnnnacgtgagagtacctgcataacgcgccggatcTCTTCATCAnnnnnnnnnnnnnnnnnnnnnnnnnnnnnnnnnnnnnGGGTAGTAAGATCTCGGCAGTATTTCTCCAAGCACTGGAATGTTGATTCTGGATCCTGGCTCGTAACGCGCTTGCTGCCGCGTATATCTTTGAGGGCGGCACTGAtagttttactggtaatgccctgactgtgAAGGAGACGtgcagtgttgctatctgtctcactTATGGnnnnnnnnnnatgttcgcaggagacatattcatcttggttcttgagcgcgaggcgttctgcacgtgcacaggctttgctcagcgttggTGCACAGCGCGTTTGGGCGGCAGGACCGGTAACACGTGGTTTGCGGCGCAGGCATTGGagtaattcatcgtacnnnnnnnnnnnnnnnnnnnnnnnnnnnnnnnnnnnnnnnnnnnnnnnnnnnnnnnnnnnnnnnnnnnnnnnnnnnnnnnnnnnnnnnnnnnnnnnnnnnnnnnnnnnnnnnnnnnnnnnnnnnnnnnnnnnnnnnnnnnnnnnnnnnnnnnnnnnnnnnnnnnnnnnnnnnnnnnnnnnnnnnnnnnnnnnnnnnnnnnnnnnnnnnnnnnnnnnnnnnnnnnnnnnnnnnnnnnnnnnnnnnnnnnnnnnnnnnnnnnnnnnnnnngtggagaacaggggtgtacatggtatgcggtgcgctttggcaggcnnnnnnnnnnnnnnnnnnnnnnnnnnnnnnnnnnnnnnnnnnnnnnnnnnnnnnnnnnnnnnnnnnnnnnnnnnnnnnnnnnnnnnnnnnnnnnnnnnnnnaccatnnnnnnnnnngcgctacggcgcttgagtatttgcggagcaatcacattaactttgagtaCGTGGATATCACCGGCAGCGGGAAGAACCTCAAGCGTTTTTTAAAGATGCGTGATTCAATGCCACTCTTTGATGACGTGAAGAAGGAAGGGCGCATTGGTATCCCGTGTCTTTCGGTGAACGACGGAGAACAGGTCTTCCTTGGTGTGGAAggtttggacctctcggcgtttcgctaggggttagtgggccggtgtgttcgGGGgcgngatgtCGTCCCCCTCTTTGCGCTCTTTTCGGGCGACCAGCTAGGCGGTTTGCTCTGCTTCAAAGCTGCTCTGGAGCGTCTGTGCGGTTGTGCGCAGCTGCTGTTCAGATTGAATGCGGAAGAAGGAGAGGAGGCTGCGTGGGATCTGCTCGTAAGGagtttcgcnnnnnnnnnnnnnnnnnnnnnnnnnnnnnnnnnnnnnngtggagannnnnnnnnnnnnnnnnnnnnnnnnnnnnnnnnnnnnnnnnnnnnnnnnnnnnnnnnnnnnnnnnnnnnnnnnnnnnTTCCAGCGCTGTGCGAGCGCCGCGCCAATTTCCGCGTGGCCTACGTCGGACATGATGGTGTCGAGCACATGCGGGGGTATGTTACGCtcCGCCTGAATTTCTGTGAGTTTGATGAGCATCTCAGGGTATGCGGAGGTGAAAACGACTTCCCCCAGGTTGTGCAGGAGGCCGCAGATGTAAGAGTCTGCGATGAGCGCCTGGTCGCCAGTGGCTTTGGCAAGACCGAGCGCAAAGTAGCCGGTGCGGTATGCTTGGTTCCACAGCTGCTTTCGCTCATCGTCGGTAGACTGCAGCACGCGCCCGGCGCCGACTGAGTACAACAGATTCTGTAATTCGCGCAGCCCGACGCGCTTCACCGCTTCGCTGAtatccaaacaACGTTtgttcataccgaagcgcgcggagttgacgagtttgagaaggtcggttacgagcgctacgtcttggctAATGAGAGCTACAATGTCTGAGAGTTGGACGTCAGGATTTTCAATGGCGCGTTGAATTTCCAGTAGTTTGCTTGGCAGCTGGGGTATGTCGTCGATGCGGTCGGCGATAGACGCGGCAAGCTTTGTGGTTTGAGTTTGGATCTCAATGTTGcggggaacnnnnnnnnnnnnAAnGGTTCGATCCTCTTCCACTACGAGGcggtacacgtcttcctcaagtccgagctttttgagcatgagcatcataattacgagcccgaggccggccccctcggaattgtccaggatgtgcgcgagtgcctcttccaaacnnnnnnnnnnnnnnnnnnnnnnnnnnnnnnnnnnnnnnnnnnnnnnnnnnnnnnnnnnnnnnnnnnnnnnnnnnnnnnnnnnnnnnnnnnnnnnnnnnnnnnnnnnnnnnnnnnnnnnnnnnnnnnnnnnnnnnnnnnnnnnnnnnnnnnnnnnnnnnnnnnnnnnnnnnnnnnnnnnnnnnnnnnnnnnnnnnnnnnnnnnnnnnnnnnnnnnnnnnnnnnnnnnnnnnnnnnnnnnnnnnnnnnnnnnnnnnnnnnnnnnnnnnnnnnnnnnnnnnnnnnnnnnnnnnnnnnnnnnnnnnnnnnnnnnnnnnnnnnnnnnnnnnnnnnnnnnnnnnnnnnnnnnnnggaTTGTTTTTGCCACCTGTCCAATATAGATGTCCATATCACGGGGGAGCGTATAGGTGGTGATGGCAATCGGGAGCTGCAGTTCAATTGCCTTACGAATTTTTTCAGTGTCAACAACTATTTGTGTATCACACATGGGCAGACTGTACACgctGTGTATGCTTTnnnnnnnnnnnnnnnnnnnnnnnnnnntccctgtgccagaatgcaaacgcgtgtgggtgcggtgtatgtctttgttgacaacggggagaaaagcgtacgaGAATCTGCCCCGTTAGGTTGAAGGGGGAGCAAACTGTACGTGCTCATGTAATCTTTGTTTTCCCGTATCGATATGGGGTGCGATGCGCGCGGTGGATACGCTCCTTCTCGACTGAGGTCGCAAGATTTTGTAGGACTGCGTAGGGGCGTGCGTCGATTGGTGTGGTGCTATGTGTCGTGCTGGTTTGATGTATCAGGACTGGGGTGTGAGGGGAAAAACGGACAGGATGTGCTTTGCGCGGAgtcggttgttTTCCCGTGGTGCGGTGGGCACGGTGTGCTGCACTGTGTTGTTTCTCGCGTGTCGTGTGCGCACTCCTTCTTCCGTGCCTCTGCGTTCTGGATCGGTCCGTGCCGCAGTACCCGAGGCCACATCCTTTCACTGGCGGCGCTATGCGGGTACGCGCCTGCGCGTGTGTTTTCCGTACCATGCTTCTTATCGCGCACTCAAAGCGATTGTTCCnnnnnnnnnnnnnnnnnnnnnnnnnnnnnnnnnnnnnnnnnnnnnnnnnnnnnnnnnnnnnnnnnnnnnnnnnnnnnnnnnnnnnnnnnnnnnnnnnnnnnnnnnnnnnnnnnnnnnnnnnnnnnnnnnnnnnnnnnnnnnnnnnnnnnnnnnnnggctactccggtcactcgattcgTTTTTTCAAAATCCTTCCCTGTGTATGCCGCATTACGATTTTGAAGATCTCATTCCTGTGTACGTAGAGACAATCGGGTATGTAGGTGGACGCAAACCCTGGCTTGGGGGTCCGGGTGCGTTTTTGTGcgccgtgccctttggagcggagannnnnnnnnnnnnnnnnnnnnnnnnnnnnnnnnnnnnnnnnnnnnnnnnnGTACCAGAAAATTATGATGAGCTTTTGGATGCCTGTAAGAGACTGCGTGAACGCGCGCAACTATACGGTCTTGCAAGCCGCGGCnnnnnnnnnnnnnnnnnnnnnnnnnnnnnnnnnnnnnnnnnnnnnnnnnnnnnnnnnnnnnnnnnnnnnnnnnnnnnnnnnnnnnnnnnnnnnnnnnnnnnnnnnnnnnnnnnnnnnnnnnnnnnnnnnnnnnngttcgcgtacggtccaccgggtatggcgagttttgcccaaaacgaagcattgcaGGCCTTTTTGCAAGGGCAGACGGGGATGTACTTGGATACCAATATGATTGGACCGTTAGTTCGTGATCCGACACGCTCAGCCATACGCCCACACCATGTGGGATTTGCGTTGCACCCGATGGCGCAGGTGCGTGCAGGAGAAGTTGGCGGCTTCGGGCTTGCAATTCCACACAATAGTGCTGCGCCCGAAGCAGCGTTCTTGCTTTTGCAGTGGATTACGGCGCCGCAAACaggacggcgAGTGGTGGAACAGGGTGCGCTACCATTCCGCCAGTCGCAGCTTGCGGACCGCGCATTGCGTGCACGTTTTGCTGAGTTTGAGGTATTAGAGCGTCAGCTTGCACATTGCGATCCAGATTGGCGTCCTATCGTGCCTACCTGGGGGGAGTTGGgaacccttttggGAATTGGGATAAATGAGGTGCTCACCGGTGTGAGTGAGCCAGAGGAAGCGATGAGCGCATTAGTGCTGCCGGCACGTCGTATTnnnnnnnnnnnnnnnnnnnnacgttatgtgccgtaaaaagtctcttcttcttgggggaagagagggcannnnnnnnnnnnnnnnnnnnnnnnnnnnnnnnnnnnnnnnnnnnnnnnnnnnnnnnnnnnnnnnnnnnnnnnnnnnnnnnnnnnnnnnnnnnnnGAGTATGAGcaaaccacgtgataccgtaagcgcgtattgttttagcgctccagcgcTTGTATTGCTGATGTTCGTGCTGGTGnnnnnnnnnnnnnnnnnnnnnnnnnnnnnnnnnnnnnnnnnnnnnnnnnnnnnnnnnnnnnnnnnnnnnnnnnnnnnnnnnnnnnnnnnnnnnnnnnnnnnnnnnnnnnnnattcaCTTTTGGGCcagtgtaagaacgacgcttgtctttacgctctcagtggtggtgcttgaggttgtacttggacttgcactcgcgctggtgttggancacggagtacccgggttgcgttTTTTTCGTACAGTGTTTGTGTTGCCGATGAtgatcnnnnnnnnnnnnnnnnnnnnnnnnnnnnnnnnnnnnnnnnnnnnnnnnnnnnnnnnnnnnnnnnnnnnnnnnnnnnnnnnnnnnnntggaccggtattgtggcttgcgaaTCCGCGTCTTGCGCTTTTGTCTGTGATACTCACTGATGTGTGGCAATGGACGCCTTTCGTATTCCTTGTGTTGCTTGCAGGTTTGCAGGGTATTCCGCAACATCTTTTGTATGCGGCGAAAGTGGACGGAGCAAATTnnnnnnnnnnnnnnnnnnnnnnnaagattccacatattgcgcctgtgcttggcattgcgACCGTCCTCCGTTTGATAGATTCTTTCCGTGGTTTGGTAGTGATTATGACACTGACAAATGGTGGTCCGGGAGTTGCAACAGAAATCCTGCCGCTTCACTTGCAGCGTATTGCCTTTGAGGATCACCGTCTGGGTAAAGCATCGGCAGTTGCTGTGCTTCTGTTTCTCCTGACAAGTCTTTTGACTTGTATTTTCATTCTCCTTACGATGAGGAGACAGGCGCGGTGAGGGTTGCGTACGGGTTAGATAAGAGCAGGAGCAGATAGCATGAACATGATTTTTTTGAAGTGGCGTACCGCGTTGGTGTTGTGTCTGTTAAGCTGTATTGCGTTGGTGAGTATGTTCCCTCTCTATGAAATGGTAGCTACTTCTTTGAAGCGTGATGCGGACGCATTTCGGTTGCCGCCAGCATGGTTTTTTATACcaacaattgaaaactatcggcaactcttgcaggaacatcattttggacgnnnnnngtataacaGCTTGGTGGTGACGTTGAGTTCCACGGTGGTGAGTGTCAGTGCAGGGGCTGCAGCAGCGTATGCAATGCAGCGCTTTCGGTACcgaggtaaaaaggcaatcacggtggcgttgttgctcttgcgagtgattccgccggttgtgnnnnnnnnnnnnnnnnnnnnnnnnnnnnnnnnnnnnnnnnnnnnnnnnnnnnnnnnnnnnnnnnnnnnnnnnnnnnnngcgctcaatgttccatttaatgtgtgggtaatcactacctttgttgcggaaattcccccTTCGCTGGATGAATCTGCAAAATTGGATGGATGTTCTCACTGGATGATTTTTACCCGCATTGTGATGCCACTGATTACACCCGCACTTGCGGTGGTGAGTATTTTTACATTTCGTTTTGCATGGAATGAGTATATGCTTGGATTTGCGCTGACCAATCGGAAAACACGGACACTGCCGGTGGCACTTTCACTTTTTCTCACGGATAGTGGTGTCGAATGGGGGCGGATTACCGCAGCAGCAACGCTGATTGCAATTCCTGCATGTGTTTTTACCTTTGCGGCGGCGAAGTACTTGGTGGTGGGTTTGACCGCAGGTGCGGTAAAGGGATAAnnACtcnnnnnnnnnnnnnnnnnnnnnnnnnnnnnnnnnnnnnnnnnnnnnnnnnnnnnnnnnnnnnnnnnnnnnnnnnnnnnnnnnnnnnnnnnnnnnnnnnnnnnnnnnnnnnnnnnnnnnnnnnnnnnnnnnnnnnnnnnnnnnnnnnnnnnngtgcgcaccgggatggcatctacatcgttggcgcaggattcgcnnnnnnnnnnnnnnnnnnnnnnnnnnnnnnnnnnnnnnnnnnnnnnnnnnnnnnnnnnnnnnnnnnnnnnnnnnnnnnnnnnnnnnnnnnnnnnnnnnnnnnnnnnnnnnnnnnnnnnnnnnnnnnnnnnnnnnnnnnnnnnnnnngtatTACTCCGCATGATCACGCGCTGATTGCAATTCCTTCTATCTCCATTGAGCGTTTGCGTGACATTTACCTGACGCTGCGCGCTGCGGGGTTTACGGTTATCAAACTTCTGCCGGCGCTTGCTCAAATCATCGATGGTACTGCGCATTTAGTGCAAACACGTGAAATTGATCCGCAGGATTTACTCACGCGCACACCGGTGACCATTAATCTTAAAAAGAGCCTGGCGTACCTATGCGGGAAACGCGTGTTAGTTACTGGTGCTGGAGGATCTATCGGCAGCGAACTAGCCTGTCAGTTGCTCTCTGGTGATGTGGAGCGTCTCTATTTGTTCGGTCATGGAGAGAACTCCATCTACCAAATCGATAAAAAAATTCGTAGGCTGCAGGAAGAGGGTGTTGGATCTCGCACAGTTATTGTTCCTGTTATTGGCGATTTAAAGGATGCTGCGTACGTGCGCTATCTTATCGAGCAGCTGCGCTGCGATGCGGTTTTTCATGCTGCCGCGTATAAGCACGTTCCTATGATGGAACTCAATCCTGTTTCAGTGATTGAAAATAATGTCTTCGGCACCAAATTCTTGCTCGATGCCTGTATTGCGTGTAGGGTTAAGCGCTTTGTACTTTTGTCCACTGACAAGGCCGTGGATCCTGTTTCTATCTACGGAGTATCTAAGATGCTCAACGAGAAGAATGTCTTGTATGCTGCTGAGCGTGTGCGCGATTTCGGTCACGATGCCGCGTATATGTTTGTCCGTTTTGGAAACGTATTGGGTTCCCGTGGTTCTATCATGCCGCTCTTTATtgaannnnnnnnnnnnnnnnnGCCCGTTACCGTGACAGATCCTGCCATGACACGATTCTTTATGactATTCCCGAAGCGTGTTCACTCGTTTTGCAAGTCGGTGGAGTAGGAGTAAATGGngcgtcgtatcttttggACATGGGGGAGCCTGTGAGCATTATGGAGACTGCGCAGCAACTTATTCGCTATTTTGGTTACGAGCCAGACAGAGATATTCCTATCCACGTGGTAGGCTTGCGTCCTGGCGAGCGTCTCAGTGAGCCACTCGTTTCCAAAGACGAGCGTATAGAGCCGACGGTATATCCAAAGGTTCTGCGTTTGCGTGAACGTGAACCTTTGGATTTTGCGCACCTTGAACGCCTGTGGGATCAACTGTATCCTTACTGTttnnnnnnnnnnnnnnnnnnnnnnnnnnnnnnnnnnnnnnnnnnnnnnnnnnnnnnnnnnnnnnnnnnnnnnnnnnnnnnnnnnnnnnnnnnnnnnnnnnnnnnnnnnnnnnnnnnnnnnnnnnnnnnnnnnnnnnnnnnnnnnnnnnnnnnnnnnnnnnnnnnnnnnnnnnnnnnnnnnnnnnnnnnnnnnnnnnnnnnnnnnnnnnnnnnnnnnnnnnnnnnnnnnnnnnnnnnnnnnnnnnnnnnnnnnnnnnnnnnnnnnnnnnnnnnnnnnnnnnnnnnnnnnnnnnnnnnnnnnnnnnnnnncgtatgtgggtgCTCCCTatgcgtgtgcggttaactcagcTACCAGTGGTTTGCTTCTCACCTTTGATGCAATGGGCATTGGGCCGGATAGTAAGATACTTACCAGTCCTTATACGTTTGTGTCTACGGCGAGCTCTGCACTCCACCTAGGTGCGCAGGTGGTGTACGCCGATATCGAGCGCGACTCTTATAATATCAGTGCAGAGTGTGTTGAAGCGTGTTTAAAAAAGGATGCGCGCatccgtgctattgtacccatccatattgccgggaatgtatgcaatatgcgtgnnnnnnnnnnnnnnnnnnnnnnnnnnnnnnnnnnnnnnnnnnnnnnnnnnnnnnnnnnnnnnnnnnnnnnnnnnnnnnnnnnnnnnnnnnnnnnnnnnnnnnnnnnnnnnnnnnnnnnnnnnnnnnCCACCAAGCCGTTAACCACCGGTGAAGGAGGTATGGTTTGCACAAATGATGCGAAGCTTGCAGCGCGTATTGCGTGTTTGCGTTCACATGGCATTGACCGGGCTATTTGGGATCGGTACACAAATGGCACCGCACCGTGGCGTTATGACGTAACAAGCCTTGGGTGGAAGTGTAACCTGCCGGATATTTTAGCAGCAATTGGACGCGTACAGTTGCAGAAGGCGGCGCATCTTTTTGCACAACGCGCGCGTATTGCCGCCGCGTTCACGCGTgctttttctcgttatgannnnnnnnnnnnnnnnnnnnnnnnnnnnnnnnnnnnnnnncatttgtatttgttgcgcttagttcctggaacgctttctGTTTCTCGGGACGAGTTCGTCAGATTATTGCAGGAACGGGGATTGGGCGTTTCTATGCATTTTATTCnnnnnnnnnnnnnnnnnnnnnnnnnnnnnnnnnnnnnnnnnnnnnnnnnnnnnnnnnnnnnnnnnnnnnnnnnnnnnnnnnnnnnnnnnnnnnnnnnnnnnnnnnnnnnnnnnnnnnnnnnnnnnnnnnnnnnnnnnnnnnnnnnnnnnnnnnnnnnnnnnnnnnnnnnnnnnnnnnnnnnnnnnnnnnnnnnnnnnnnnnnnnnnnnnnnnnnnnnnnnnnnnnnnnnnnnnnnnnnnnnnnnnnnnnnnnnnnnnncgttgcaggattgctgctgaacatagatattccctctCTCCCTGACGGGTACTCTTTTTATACTGCAGCACATATTCCCGGATGCAATGCCGTTCGGTGTGGGGAAAATACTGTGCCGGTTTTTGCGCATGGAGAGGTGGTGTACGCAGGGGAACCGGTGGGTATCCTCATTGGGCCTGATGAGCATGtggtacgtaatttagtgcaagatgtggtggtgcatacgtgcgcagagcgggcctgtgcgtcggannnnnnnnnnnnaatcagtgannnnnnnnnnnnnnnnnnnnnnnnnnnnnnnnnnnnnnnnnnnnnnnnnnnnnnnnnnnnnnnnnnnnnnnnnnnnnnnnnnnnnnnnnnnnnnnnnnnnngtgtacactactttgcggaaatgccagaagtacaggcactannnnnnnnnnnnnnnnnnnnnnnnnnnnnnnnnnnnnnnnnnnnnnnnnnnnnnnnnnnnnnnnnnnnnnnnnnnnnnnnnnnnnnnnnnnnnGCGGTGCACGTACATCCGCAGCAGGAAGCGCTTTCCTGTGATGGGAGAATATGGTTCCCCTCAGTGAtggcaagtcaggcggcGCTTGCAGCCTATTGTGCGAAAAAGCCGGTACGCTTGTCTTTTTCCTTTCAAGAGTATGTGCAGTActgtcctaagnnnnnnnnnnnnnnnnnnnnnnnnnnnnnnnnnnnnnnnnnnnnnnnnnnnnnnnnnnnnnnnnnnnnnnnnnnnnnnnnnnnnnnnnnnnnnnnnnnnnnnnnnnnnnnnnnnnnnnnnnnnnnnnnnnnnnnnnnnnnnnnnnnnnnnnnnnnnnnnnnnnnnnnnnnnnnnnnnnnnnnnnnnnnnnnnnnnnnnnnnnnnnnnnnnnnnnnnnnnnnnnnnnnnnnnnnnnnnnnnnnnnnnnnnnnnnnnnnnnnnnnnnttaatcagttatgtgctnagcttcatatATTCCCTGACGAGTGGCGTGTGGCGCACATGAAAGATACGCGGGAAACACAGCGTTTTGCGCGGTTGCTCGCCTATCTGTGTGAGGAAGGAGATTTTCGTCGAAAGCACGCAGCCTTCAGCATGGTCAATGCAGTACGAAAAGCACATGACACCCATGCCTGGCGTGGTATTGGACTCGCGTTGGGGTTTCAATATGATCCGTCTGCGATGTTAGCCCGTTCGGGTTTTTCCTATGTATTACAAATGACGCTGCACACTGATGCGcgcattgtnnnnnnnnnnnnnnnnnnnnnnnnnnnnnnnnnnnnnnnnntggttgcgtttctcatcagagaGTTTGCGTGTCTGGAGGATGCGATCTTTTTTAAAAGTAGTGATGAGGCGTatggcgtggatctgttgggtccgtctgTGGAATCAGTGGGGATGAGGGTGTTTGCACGGTTGGTGAGAAAGTGTGTACGAGCAATTCAGAGACAACGCTTCagaaagccacttcctatcacggtacaggggtcctttaacacggccaagaaggggcaggtgtatcaagtggtgactgtnnnnnnnnnnnnnnnnnnnnnnnnnnnnnnnnaatctgagcagtgtgcntcnnnnnnnnnnnnnnnngctGATACTAGCGGAAAATGTGAGGATATGAACGGTTTTACCAAAATGCACGGAATGAGCACGCACACTCCTGCAGCCTGTATTATTGAACTCGAATTAGATGCGTTGTGCGTGCAacctaagattgtcaggttgtggtttgtttgcGATCctgggtatgtcttttgtgaaaaagatgtgtaccgtaccgtgagtcgaagcattactcgtgcgctttcgcacgtatctgtagaaaagatttgggagcgtgcgcgcacacccgagtatgttatcatcgatccatccgatactcctccctnnnnnnnnnnnnnnnnnnnnnnnnnnnnnnnnnnnnnnnnnnnnnnnnnnnnnnnnnnnnnnnnnnnnnnnnnnnnnnnnnnnnnnnnnnnnnnnnnnnnnnnnnnnnnnnnnnnnnnnnnnnnnnnnnnnnnnnnnnnnnnnnnnnnnnnnnnnnnnnnnnnnnnnnnnnnnnnnnnnnnnnnnnnnnnnnnnnnnnnnnnnnnnnnnnnnnnnnnnnnnnnnnnnnnnnnnnnnnnnnnnnnnnnnnnnnnnnnnnnnnnnnnnnnnnnnnnnnnnnnnnnnnnnnnnnnnnnnnnnnnnnnnnnnnnnnnnnnnnnnnnnnnnnnnnnnnnnnnnnnnnnnnnnnnnnnnnnnnnnnnnnnnnnnnnnnnnnnnnnnnnnnnnnnnnnnnnnnnnnnnnnnnnnnnnnnnnnnnnnnnnnnnnnnnnnnnnnnnnnnnnnnnnnnnnnnnnnnnnnnnnnnnnnnnnnnnnnnnnnnnnnnnnnnnnnnnnnnnnnnnnggaaaaattctcaccgcagnnnnnnnnnnacaaaggaattctgtgcccaacgaagaagaggtaagacacgccttttcaggcatgcaatgccgGTGTACTGATATCAATGCGTTGATTCGAGCACTTCAACGTATGCCTGCTTGTCATGAGTtttcctaaaactgtatgcatctgtataagnnnnnnnnnnnnnnnnnnATTTATTACGTAAAAAGTTTGGGTCaATTGTGTGCGGTACTACGTaacgtcgcgcaggtacagccagttgggggtggaacgggcttggtgcaatatnnnnnnnnnnnnnnnnnnnnnnnnnnnnnnnnnnnnnnnnnnnnnnnnnnnnnnnnnnnnnnnnaagatatttctaaaactgagcactttcttgagtttggtggtgctgtgtcgttgcaagcaattgtgcaattgggaagaaaaaatattcccgtggcnnnnnnnnnnnnnnnnnnnnnnnnnnnnnnnnnnnnnnnnnnnnnnnnnnnnnnnnnnnnnnnnnnnnnnnnnnnnnnnnnnnnnnnnnnnnnnnnnnnnnnnnnnnnnnnnnnnnnnnnnnnnnnnnnnnnnnnnnnnnnnnnnnnnnnnnnnnnnnnnnnnnntgtggcacactatgcacatgcgcgttctgacacgccgcgacaccggagtcatgtaattacccgtattcgccttccaacagattactgggacttttcctactacagacgtattgggtcgcnnnnnnnnnnnnnnnnnnnnnnngatttcgtgnnnnnnnnnnnnnnnnnnnnnnnnnnnnnnnnnnnnnnnnnnnnnnnnnnnnnnnnnnnnnnnnnnnnnnnnnnnnnnnnnnnnnnnnnnnnnnnnnnnnnnnnnnnnnnnnnnnnnnnnnnnnnGGATATTGCGGCAATCGtatatcgaagcagagagttctttgcgcctgaatcctttaagAGTGCGTACATCGCGCACTGCTTCTTTCATCTGCTGGAAGACTGTTTGCGCCGCTTAAGAtgaagcTACAGGTGGCGAGTTTTACCCAGGCACGCGCAAACAGCTGACGCTAAAGACCGAGTTTTTTCATTTTGCTTTGCAATGTACTTGGCTTAAGACCAAGAATTTCTGCTGCGCCAtttgctccGTATATCTTACCGTTGCTTGCATCAAGCGCCGCTTGAATTGCTGCGCGTTGCGCCTGATGAAAGTTGACCACCAGCGTAGTTTCTTCCTTTTTCCCCTGTGTGCATCGCACCATGCAAGAGCTATCGCGTATGCACAGAGGTATAGCTGGTTGAACGCTTTCTGACACTTCGGTGTGCTCCCGAGGATAGACAGTAGTCTGCGTGCCGGATTCCGGGGTCCTACATACAAGGTGTTCTGCGCCGATGGTatctccgcgtgcaagaagtgcaGCACGCTCGAGTAGGTTGCGTAaCTCACGCACATTGCCAGGAAACGTGAGCGAGAAGATTTTCTTAAACGCGCTGGGAGAAAGCTGAGnGCGCTCAAACCCCGGGCGGGTCTTAATTTTTTGGATAAAATGCTCCGCTAGAAGCGCAACGTCTTCTGCACGCTCGCGCAAAGGGGGGAGACTGAGGGGAAAAACATCGAGCCGGTAGAGGAGGTCTTCCCTGAATTTCCCTTGGGTGACnnnnnnnnnnnnnnnnnnnnnnnnnnnnnnnnnnnnnnnnnnnnnnnnnnnnnnnnnnnnnnnnnnnnnnnnnnnnnnnnnnnnnnnnnnnnnnnnnnnnnnnnnnnnnnnnnnnnnnnnnnnnnnnnnnnnnnnnnnnnnnnnnnnnnnnnnnnnnnnnnnnnnnnnnnnnnnnnnnnnnnnnnnnnnnnnnnnnnnnnnnnnnnnnnnnnnnnnnnnnnnnnnnnnnnnnnnnnnnnnnnnnnnnnnnnnnnnnnnnnnnnnnnnnnnnnnnnnnnnnnnnnnnnnnnnnnnnnnnnnnnnnnnnnnnnnnnnnnnnnnnnnnnnnnnnnnnnnnnnnnnnnnnnnnnnnnnnnnnnnnnnnnnnnnnnnnnnnnnnnnnnnnnnnnnnnnnnnnnnnnnnnnnnnnnnnnnnnnnnnnnnnnnnnnnnnnnnnnnnnnnnnnnnnntaaaTGCAGGTGATTGGGGAGCTAAGAGCGCATTTCGTTCGGTCAAAAGAGCGTGACTCTTTTGACTCAGTGTCTCGGACGCGTCGGTctgggctactgcgagcgagataagtttagaaagagtagtaatgaagcgtacaacGTCTGGGGTAAACtgctcgcacaggcgatggtcgagcgnnnnnnnnnnnnnnnnnnnnnnnnnnnnnnnnnnnnnnnnnnnnnnnnnnnnnnnnnnnnnnnnnnnnnnnnnnnnnnnnnnnnnngtatcAGTGTGGGCAAGCGTCGGATCGAAAAGGTATGGACTCTTCTGTGATAGGATGCGCGCAAGATCCTGCCTTTTGGTGAGGTCTATGGtgtggtgttggaggcgggGAGTGTACaggggaccacgcgccttgcgaactcgcagtannnnnnnnnnnnnnnnnnnnnnnnnnnnnnnnnnnnnnnnnnnnnnnnnnnnnnnnnnnnnnnnnnnnnnnnnnnnnnnnnnnnnnnnnnnnnnnnnnnnnnnnnnnnnnnnnnnnnnnnnnnnnnnnnnnnnnnnnnnnnnnnnnnnnnnnnnnnnnnnnnnnnnnnnnnnnnnnnnnnnnnnnnnnnnnnnnnnnnnnnnnnnnnnnnnnnnnnnnnnnnnnnnnnnnnnnnnnnnnnnnnnnnnnnnnnnnnnnnnnnnnnnnnnnnnnnnnnnnnnnnnnnnnnnnnnnnnnnnnnnnnnnnnnnnnnnnnnnnnnnnnnnnnnnnnnnnnnnnnnnnnnnnnnnnnnnnnnnnnnnnnnnnnnnnnnnnnnnnnnnnnnnnnnnnnnnnnnnnnnnnnnnnnnnnnnnnnnnnnnnnnnnnnnnnnnnnnnnnnnnnnnnnnnnnnnnnnnnnnnnnnnnnnnnnnnnnnnnnnnnnnnnnnnnnnnnnnnnnnnnnnnnnnnnnnnnnnnnnnnnnnnnnnnnnnnnnnnnnnnnnnnnnnnnnnnnnnnnnnnnnnnnnnnnnnnnnnnnnnnnnnnnnnnnnnnnnnnnnnnnnnnnnnnnnnnnnnnnnnnnnnnnnnnnnnnnnnnnnnnnnnnnnnnnnnnnnnnnnnnnnnnnnnnnnnnnnnnnnnnnnnnnnnnnnnnnnnnnnnnnnnnnnnnnnnnnnnnnnnnnnnnnnnnnnnnnnnnnnnnnnnnnnnnnnnnnnnnnnnnnnnnnnnnnnnnnnnnnnnnnnnnnnnnnnnnnnnnnnnnnnnnnnnnnnnnnnnnnnnnnnnnnnnnnnnnnnnnnnnnnnnnnnnnnnnnnnnnnnnnnnnnnnnnnnnnnnnnnnnnnnnnnnnnnnnnnnnnnnnnnnnnnnnnnnnnnnnnnnnnnnnnnnnnnnnnnnnnnnnnnnnnnnnnnnnnnnnnnnnnnnnnnnnnnnnnnnnnnnnnnnnnnnnnnnnnnnnnnnnnnnnnnnnnnnnnnnnnnnnnnnnnnnnnnnnnnnnnnnnnnnnnnnnnnnnnnnnnnnnnnnnnnnnnnnnnnnnnnnnnnnnnnnnnnnnnnnnnnnnnnnnnnnngCCCCTTCGATTtggcgcgtcttgaacaggatttgcgtcggatcccggatcaaatcttatgtgcgtgggttccgcgtcatgtgtcacagacgggtgatgcgctgtctctttctgagttatggcttttgcacgaccgGTCTAGTCTTGCAAnnnnnnnnngttttgcgcgggcagatttgaaaaagggtatctacgtannggcgcatcacattaaggatcctaagcttcgtgcaatgcacttcaaaacgattgcngacaataaanttaatatgcttgtgtacgnnnnnnnnnnnnnnnnnnnnnnnnnnnnnnnnnnnnnnnnnnnnnnnnnnnnnnnnnnnnnnnnnnnnnnnnnnnnnnnnnnnnnnnnnnnnnnnnnnnnnnnnnnnnnnnnnnnnnnnnnnnnnnnnnnnnnnnnnnnnnnnnnnnnnnnnnnnnnnnnnnnnnnnnnnnnnnnnnnnnnnnnnnnnnnnnnnnnnnnnnnnnnnnnnnnnnnnnnnnnnnnnnnnnnnnnnnnnnnnnnnnnnnnnnnnnnnnnnnnnnnnnnnnnnnnnnnnnnnnnnnnnnnaaaggtgtggcngtanaatgncgccatcgcgaaagaagcaattgagtttggcttcgatgaggtacagttcgattatatncggttccctaccgacggagataaccttcaccnnnnnnnnnnnnnnnnnnnnnnnnnnnnnnnnnnnnnnnnnnnnnnnnnnnnnnnnnnnnnnnnnnnnnnnnnnnnnnnnnnnnnnnnnnnnnnnnnnnnnnnnnnnnnnnnnnnnnnnnnnnnnnnnnnnnnnnnnnnnnnnnnnnnnnnnnnnnnnnnnnnnnnnnnnnnnnnnnnnnnnnnnnnnnnnnnnnnnnnnnnnnnnnnnnnnnnnnnnnnnnnnnnnnnnnnnnnnnnnnnnnnnnnnnnnnnnnnnnnnnnnnnnnnnnnnnnnnnnnnnnnnnnnnnnnnnnnnnnnnnnnnnnnnnnnnnnnnnnnnnnnnnnnnnnnnnnnnnnnnnnnnnnnnnnnnnnnnnnnnnnnnnnnnnnnnnnnnnnnnnnnnnnnnnnnnnnnnnnnnnnnnnnnnnnnnnnnnnnnnnnnnnnnnnnnnnnnnnnnnnnnnnnnnnnnnnnnnnnnnnnnnnnnnnnnnnnnnnnnnnnnnnnnnnnnnnnnnnnnnnnnnnnnnnnnnnnnnnnnnnnnnnnnnnnnnnnnnnnnnnnnnnnnnnnnnnnnnnnnnnnnnnnnnnnnnnnnnnnnnnnnnnnnnnnnnnnnnnnnnnnnnnnnnnnnnnnnnnnnnnnnnnnnnnnnnnnnnnnnnnnnnnnnnnnnnnnnnnnnnnnnnnnnnnnnnnnnnnnnnnnnnnnnnnnnnnnnnnnnnnnnnnnnnnnnnnnnnnnnnnnnnnnnnnnnnnnnnnnTGGCAGACCACTGCAATACTCGCGCAGGCGCGCGCTCGCCTCGTCTGCCCCTACCACAGCGTTGACAACCATAGCnnnnnnnnnnnnnnnCCGTCAGTCTGGGGCTTACCAnCCCnCCCCCTGCaccgataccgcacaagctacgcgagttcaGTCAACTCGGCGCACAGCACACGCAAAAGACCGCTACTCGGTGAGGATTTTAATAACCTCCGCTTTACTTGTTGCATCAAGGATCTCCCGACGTTTCTCAGAACTCTTAAACAGGAGACTGATCTCAGCCAAGAACTGCAGGTGCGGACCAGTGAcgtcaagtggagaaagggtcattatgaaaatacgacaaggttcttgatccaaagagtcgaagtcaaccnnnnnnnnnnnnnnnnnnnnnnnnnnnnnnnnnnnnnnnnnnnnnnnnnnnnnnnnnnnnnnnnnnnnnnnnnnnnnnnnnnnnnnnnnnnnnnnnnnnnnnnnnnnnnnnnnnnnnnnnnnnnnnnnnnnnnnnnnnnnnnnnncccttcctgcacggacgaacatctcgagcatttcgtcgatgatctcctCCTTGGTAGAACCCTTCAGGTGCaggcttacggtttccggcgtcaacacggtctccaaattcattcccccaagctaaaaactctcaaaggaaaagtcaagcttnnnnnnnnnnnnnnnnnnnnnnnnnatcTTGACCTTTTGCCTACTTGGactttaccatgcgcgcatggggttcgccaccagggagcagctgaataggtactacgatttgtacaaggatgtcgatgtaactttctcaaaggatgtgatgcaggcgctctgttttaatgcgcggcaggnnnnnnnnnnnnnnnnnnnnnnnnnnnnnnnnnnnnTAATGAATTCTGTGTCTATGGTGGGTGCGAAAGTTATTCTCAGCAGGAAGAGTAGTCTGCTTGAGAGCATTCAAGTGGAAGGGGCGAGCGTCAGCATACGGTTTTCTTTCTTTGAGTCCGATGCGCGGGATGCGGTTTCCTTCTTCGTTACTGCCAGGGTTCTCGGTGTTGAAGACTATGCCGAGAGTACGGAGCTAGTGGTGTTAAGCGTGGCGTATACGCAGCGCATACCTGATATGCTCATAGAGCGTTTGGGTTTGCTTGTTGAGGCCAACATTAGTTCCAAGAAGCGTAAGTCGGAGCgtattgcnnnnnnnnnnnnnnnnnnnnnnnnnnnnnnnnnnnnnnnAGCGGAGACCATCGTGTTCATTCAGGcgattcctcgccgctgcgttctgcGGGATGTTTCCTTTGGTGGTGCGAAGTTTATCATGATGGGCGTTGCGCCGTTTTTGAAAGGCAAGGAGACGGTGCTGAAGCTTGATTTTGAGGAGCCnAGTACGAGCATGAGTATTAGGGGGCACGTGGTGCGTGCAGATCAGGTTGAGGGGCGTAAAGACCTGGTGGCCGTGGCCATGGAGTACGACTTTGATGtggtgcCTGTCGCGTATCGTATGTGtttgaaccgctacgcatcggaccgctgtcgccgttttcccggtacggacgaggactgctctgcggcgtctgccnnnnnnnnnnnnnnnnnnnnnnnnnnnnnnnnnnnnnnnnnnntttctgtacccttctcnnnnnnnnnnnnnnnnnnnnnnnnnnnnnnnnnnnnnnnnnnnnnnnnnnnnnnnnnnnnnnnnnnnnnnnnnnnnctccgngcactgngcctcgctccgnccagtagtttcgaacgcacaaaccagcctcgtggnnnnnnnnnnnnnnnnnnnnnnnnnnnnnnnnnnnnnnnnnnnnnnnnnnnnnnnnngactaagtcttctatctgtacgatcgccccttGAGcatccaggaatgcgagagagagcgggtgtggggtgtccttcatccaaaaggagaggcgtgtgtcctgtttatacacgaaaagcatgcccgtcccgtcggggatccgtgtacgccccnnnnnnnnnnnnnnnnnnnnnnnnnnnnnnnnnnnnnnnnnnnnnnnnnnnnnnnnnnnnnnnnnnnnnnnnnnnnnnnnnnnnnnnnnnnnnnnnnnnnnnnnnnnnnnnnnnnnnnnnnnnnnnnnnnnnnnnnnnnnnnnnnnnnnnnnnnnnnnnnnnnnnnnnnnnaggtagaaggagacgtgctcttcaaaagccttgtacaaaacgctttcgatccttttcagcattgctctTTTGAGTGTCGCGTGCACTAAGCTGTTCGTTAAAGGCGCGGATATCTATGTACTTGnnnnnnnnnnnnnnnnnnnnnnCTAGGcgtGTGGACGCGTCTTCCCACAAGGCAACCCGAGGACTGAGCTTATGAGGTTCGCCGTATTTTTTACACAAGTTCTCATATACCGAGTAGTAATCTATnnnnnnnnnnnnnnnnnnnnnnnnnnnnnnnnnnnnnnnnnnnnnnnnnnnnnnnnnnnnnnnnnnnnnnnnnnnnnnnnnnnnnnnnnnnnnnnnnnnnnnnnnnnnnnnnnnnnnnnnnnnnnnnnnnnnnnnnnnnnnnnnnnnnnnnnnnnnnnnnnnnnnnnnnnnnnnnnnnnnnnnnnnnnnnnnnnnnnnnnnnnnnnnnnnnnnnnnnnnnnnnnnnnnnnnnnnnnnnnnnnnnnnnnnnnnnnnnnnnnnnnnnnnnnnnnnnnnnnnnnnnnnnnnnnnnnnnnnnnnnnnnnnnnnnnnnnnnnnnnnnnnnnnnnnnnnnnnnnnnnnnnnnnnnnnnnnnnnnnnnnnnnnnnnnnnnnnnnnnnnnnnnnnnnnnnnnnnnnnnnnnnnnnnnnnnncatctactgnnnnnnnnnnnnnnnnnnnnnnnnnnnnnnnnnnnnnnnnnnnnnnnnnnnnnnnnnnnnnnnnnnnnnnnnnnnnnnnnnnnnnnnnnnnnnnnnnnnnnnnnnnnnnnnnnnnnCAATAGGTTCTCGTAAAAAGTCTTCGgttacctgagagacgatgtctttagggaaaccgcacatgtgtgcgagttctatgggaccgaattcaaaatcgtaggccttgccggtgctaggtatgtagcgaatcttttctaactggatggccaannnnnnnnnnnnnnnnnnnnnnnnnnnnnnnnnnaaagtatttGCGAGCTGTCGGTACATCGACCAGATGCGATCTGCGAGCGTGGTGGTAAGACGCGCAGTCAATTGCGGTTGTGTGGCTACCAGCTGTTGGAAGTTCTTTCGGTTCACGGCCAAAAGCTGGCAACCATCAGACATAACAATGGcgcttgcagnnnnnnnnnngttctccagcaacgccatttccccaaACATATCTCCTTCTTTTAAAATCGCCAGCACTACCTCATTGTTATCAACAATCTTAGTAATTTTTACATGTCCTTTTTGAATGATGTAAAACTCATTTCCCAATTGACACTCACAGAACACCATCGCCTCTCGATCGTAGCAGCGCGTGGCTTCAAGTATGTTAGGTTCGAGTATTTCTACTGGTACCTTAACTCCTGTGGATTTAATCGCAACAAATCgtttgcgtgcttcctctgcatacgtccccttgggactttccttgagatnnnnnnnnnnnnnnnnnnnnnnnnnnnnnnnnnnnnnnnnnnnnnnnnnnnnnnnnnnnnnnnnnnnnnnnnnnnnnnnnnnnnnnnnnnnnnnnnnnnnnnnnnnnnnnnnnnnnnnnnnnnnnnnnnnnnnnnnnnnnnnnnnnnnnnnnnnnnnnnnnnnnnnnnnnnnnnnnnnnnnnnnnnnnnnnnnnnnnnnnnnnnnnnnnnnnnnnnnnnnnnnnnnnnnnnnnnnnnnnnnnnnnnnnnnnnnnnnnnnnnnnnnnnnnnnnnnnnnnnnnnnnnnnnnnnnnnnnnnnnnnnnnnnnnnnnnnnnnnnnnnnnnnnnnnnnnnnnnnnnnnnnnnnnnnnnnnnnnnnnnnnnnnnnnnnnnnnnnnnnnnnnnnnnnnnnnnnnnnnnnnnnnnnnnnnnnnnnnnnnnnatgtaagaacccTTCTTGAAGTTAACAAACGTCAGCTGTAACAAAGTATCCCACTCCTTCCTAATCGTCGCTCTATGCTCTGTTTACAagacaacccgtacgtcttgcagtgcacaggtgcccgCCTCATGAGTCTCGCGAACTGGAAGTCatcctcacagatnnnnnnnnnnnnnnnnnnnnnnnnnnnnnnnnnnnnnnnnnnnnnnnnnnnnnnnnnnnnnnnnnnnnnnnnnngagtgtgcgcacctgatgtgcggtggcatTCCCCGTATTGATAATGAGATTTCCATGCCAGGGTGCTACCTGCGCAGCCCCACAGGAGGTGCCCCGTAAACCTGCCTCTTCTATGAGAATGCCAGACGGTTTACCAAAAGCTGGGTTGTTTTTAAACGCGCTGCCTGCTGACGGAAAGCGAAACTGCCCCTTTGAAAtacGATCGGCAATCTTCTCCtgcatgtgcttcctaatctgcgccggattgccgGGAGTGAGACGTACACACAGCGAGAGGATAAGACGCCTTCCTGCATGGAGTTCAACACCGTGAGGACTCTGGAAAGgagagcgcttgtagccccaatccccnnnnnncgcgaagacggtctgaaaactgctgcaggtaaacggtccgccgtnnnnnnnnnnnnnnnnnnnnnnnnnnnnnnnnnnnnnnnnnnnnnnnnnnnnnnnntcttttgcgcgcgaacgcacggggtgaagtacgagcgtgcgcgcagagtgaannnnnnnnnnnnnnnnnnnnnnnnnnnnnnnnnnnnnnnnnnnnnnnnnnnnnnnnnnnnnnnnnnnnnnnnnnnnnnnnnnnnnnnnnnnnnnnnnnnnnnnnnnnnnnnnnnnnnnnnnnnnnnnnnnnnnnnnnnnnnncgcgcctgcatgtacgagcactgagccatcgcgctgtgtttgggtgtgtagactgcgaaagcgacgaaggctcaacatcagacccggtacgccctcgtctgcgattaacacgttagagcctcccnnnnnnnnnnnnnnnnnnatgcgtgcgnnnnnnnnnnnnncaataagcgcgcgcagctgtgtgcaggagcgcggctccgcccaaaactgcgcagcgccaccaatgcggaaagaaCATCGCTCTGCAAGTGGGACGTTACGGCGCGTGATCCGACGCGCGCGTATCCGGTgcgcgaacatggacagaaaacannnnnnnnnnnnnnnnnnnnnnnnnnnnnnnnnnnnnnnnnnnnnnnnnnnnnnnnnnnnnnnnnnnnnnnnnnnnnnnnnnnnnnnnnnnnnnnnnnnnnnnnnnnnnggctgggcgtggtccgcacgtgttctgcgcccggttgggagaaacttcgaaggggcGCACATGGCCTTGCGCGTATAnnnnnnnnnnnnnnnnnnnnnnnnnnnnnnnnnnnnnnnnnnnnnnnnnnnnnnnnnnnnnnnnnnnnnnnnnnnnnnnnnnnnnnnnnnnnnnnnnnnnnnnnnnnnnnnnnnnnnnnnnnnnnnnnnnnnnnnnnnnnnnnnnnnnnnnnnnnnnnnnnnnnnnnnnnnnnnnnnnnnnnnnnnnnnnnnnnnnnnnnnnnnnnnnnnnnnnnnnnnnnnnnnnnnnnnnnnnnnnnnnnnnnnnnnnnnnnnnnnnnnnnnnnnnnnnnnnnnnnnnnnnnnnnnnnnnnnnnnnnnnnnnnnnnnnnnnnnnnnnnnnnnnnnnnnnnnnnnnnnnnnnnnnnnnnnnnnnnnnnnnnnnnnnnnnnnnnnnnnnnnnnnnnnnnnnnnnnnnnnnnnnnnnnnnnnnnnnnnnnnnnnnnnnnnnnnnnnnnnnnnnnnnnnnnnnnnnnnnnnnnnnnnnnnnnnnnnnnnnnnnnnnnnnnnnnnnnnnnnnnnnnnnnnnnnnnnnnnnnnnnnnnnnnnnnnnnnnnnnnnnnnnnnnnnnnnnnnnnnnnnnnnnnnnnnnnnnnnnnnnnnnnnnnnnnnnnnnnnnnnnnnnnnnnnnnnnnnnnnnnnnnCTCCGTGGGGGCGGGGGTACCCCGGGTGGCACATCGGGTGTTCTGCAATGAGCATGAAGTTTTTAGGACCACGTTGCGACATCCACATCGGAGGGGTGGATCATATTCGTGTgcatcaccgtaacgagcgtgctcagtgtgaagcaattactggtgcaccctgggtgaggtactggttacaccacgagttctnnnnnnnnnnnnnnnnnnnnnnnnnnnnnnnnnnnnnnnnnnnnnnnnnnnnnnnnnnnnnnnnnnnnnnnnnnnnnnnnnnnnnnnnnnnnnnnnnnnnnnnnnnnnnnnnnnnnnnnnnnnnnnnnnnnnnnnnnnnnnnnnnnnnnnnnnnnnnnnnnnnnnnnnnnnnnnnnnnnnnnnnnnnnnnnnnnnnnnnnnnnnnnnnnnnnnnnnnnnnnnnnnnnnnnnnnnnnnnnnnnnnnnnnnnnnnnnnnnnnnnngctcgagcaactacaggcagcgtgcgcggcactagtgcagagtgtgccgcagaaaggGTGTGTGAATCGCGCGCATCAGAATCTGAGCTGCTCTTAACTGACTTTCGTGCTGCGTTGGAGGATGACTTTTCTACGCCACGTGCTCTGAGCGCCTTACAAAAATTGGTGCGTGATACCTCGGTGCCGCCATCGCTGTGTGTTTCGGCACTCCAGGTGGCGGATACAGTGCTAGGGTtaggcataatacaggnnnnnnnnnnnnnnnnnnnnnnnnnnnnnnnnnnnnnnnnnnnnnnnnnnnnnnnnnnnnnnnnnnnnnnnnnnnnnnnnnnnnnnnnnnnnnnnnnnnnnnnnnnnnnnnnnnnnnnnnnnnnnnnnnnnnnnnnnnnnnnnnnnnnnnnnnnnnnnnnnnnnnnnnnnnnnnnnnnnnnnnnnnnnnnnnnnnnnnnnatttggaagcgcgtgtaacattttgggagatacattgttgcatgagcaggagcttttaagagcacaggatgatgcagattttaaGCTCATGTACGAGCAGCTTGTGCCAGTGCTCTACcgcgtagcgtacaacgtggtgcgcgaggaggacatcgctgaggggctctgccatgaTGCCTTCATTGCAATGACAGAAAAGAGGATGGAGTTTCCGTCTCTGTCGGACGCAAAGTATtgGTTGAtccgcgtggtgaaaaatgcctcgttaaattacgctaagcgtcgtgtacgtgagcgtcattCTTGTGAGCAAGCGTCGCGCGGGCATGTGTGCGAGCCGGATACCGGTGAGCTTCGCTTGTTAAGAATAGAGACGATTGAGCAGGTGCGCGCGGCCTTAGATnnnnnnnnnnnnnnnnnnnnnnnnnnnnnnnnnnnnnnnnnnnnnnnnnnnnnnnnnnnnnnnnnnntcggacgtatcctgggcatcagcgaggggaatgtaaaggtgagggtgttcagagcgcgcgnnnnnnnnnnnnnnnnnnnnGGAGAGACGGATGCGTACCTGTCCTGATTGTGCTGCTTGGTGTGCTTATGTGGACGGAGAAGGTTCGCAACTGCAACGCCGTGAGATGTGCGCGCATCTGCAGGGTTGCaCACACTGTGCCACGTGTGTGGCGCACTATcGCGCCATGCGGAGTCTTGTCAAGCATGCTGATCGCGTTTCTTCCCGTGATTTTACAATGGCTTTTCCATATTTGCGCGTGCGTCACCGTGTCGCTTCCTGTATGCCGAGGCCGTGGTGGCAGGCACGTTCCTCTCCTCTTTCTGCTGCAGGACCGGTccgtgctgcggcactcGCTGTGGCGGTCGCATCTTTATGTGTATGCACCCTGTTGCTTACTCATATTGTTGAAAGGCGTCCTGTATCCCGTGCGGGTGAGGCGAGTTTTACCCCCATTGTACCTATGCGTGTTCGCGCCCCTGTTGGGTACGCGCGCGGTGTGAAAGTGTTTGGTCCTGCCGTTAGTGCGAATTCCAACGTGCTGCGCAAACCAGCTGCGGTGTTCACCGTCTGTGCGTTTGCGCAGTTGTATGGCTCAGATCCTGCGTATGAAATGGAAACAGTGCCGGTGAGGCtatcggttatTCCTGTGCnnnnnnnnnnnnnnnnnnnnnnnnnnnnnnnnnnnnnnnnnnnnnnnnnnnnnnnnnnnnnnnnnnnnnnnnnnnnnnnnnnnnnnnnnnnnnnnnnnnnnnnnnnnnnnnnnnnnnnnnnnnnnnnnnnnnnnnnnnnnnnnnnnnnnnnnnnnnnnnnnnnnnnnnnnnnnnnnnnnnnnnnnnnnnnnnnnnnnnnnnnnnnnnnnnnnnnnnnnnnnnnnnnnnnnnnnnnnnnnnnnnnnnnnnnnnnnnnnnnnngcgtacgctcgaagccgcccgtttgattgtgttcaGAAACATAGCcgcaaaagtttttcttgtcggatgcgagcgtgatatcaaaaacaccgcagacaggtgcggtatcgACCTTACCGACATGGTCGTCATCGATCCGAGCGTTAGCAAGCACAGAGATCAGTTCGCAGAacgttattttcagaagcgaaaacacaaaggaataagtcttgcccaggctgcagaggatnnnnnnnnnnnnnnnnnnnnnnnnnnnnnnnnnnnnnnnnnAGGTCACGCAGATGCCATGGTTGCCGGTGCAGAAAACACTACCGCGCGCGTTCTTCGTGCAGGCCTCACCATCATCGGAACCCTTCCGAGTGTTAAAACTGCCTCTTCCTGCTTCGTTATGGATACTAATAACCCCCGTCTGGGAGGAACACGTGGTCTATTTATTTTTTCAGACTGTGcagtgnnnnCCACTCCCACCGCAGAACAGTTGGCTGATATCGCCTGCTCTGCTGCAGAAAGCTGCCGCACCTTCATTGGAGAGGAACCGACTGTCGCACTTCTTTCCTACTCTACTAAAGGATCAGGAGGTGATAGTGACGAGAATATCCTGCGTGTACGTGAggcagtcaggattctacacgaannnnnnnnnnnnnnnACCTTCGATGGGGAATTGCAGCTCGATGCTGCGCTCGTACCTAAGATTACCGAAAAAAAAGCGCCTCACAGTCCTATTACGGGAAAGGTGAACAnnnnnnnnnTTCCCGATCTTTCTTCGGGTAATATTGGGTACAAGCTTGTCCnnnnnnnnnnnnnnnnnnnnnnnnnnnnnnnnnnnnnnnnnnnnnnnnnnnnnnnnnnnnnnnnnnnnnngcgtgggtgctcggntgaagatatcgtcgccgcttgtgcagtcacacttgtgcaatcgaatggacgctaatgacgtccacccaggcgcgtatacgtgaggcagtccgtgcagggagcgtccgagattatgcgcgtgctatccgtattcnnnnnnnnnnnnnnnnnnnnnnnnnnnnnnnnnnnnnnnnnnnnnnnnnnnnnnnnnnnnnnnnnnnnnnnnnnnnnnnnnnnnnnnnnnnnnnnnnnnnnnnnnnnnnnnnnnnnnnnnnnnnnnnnnnnnnnnnnnnnnnnnnnnnnnnnnnnnnnnnnnnnnnnnnnnnnnnnnnnnnnnnnnnnnnnnnnnnnnnnnnnnnnnnnnnnnnnnnnnnnnnnnnnnnnnnnnnnnnnnnnnnnnnnnnnnnnnnnnnnnnnnnnnnnnnnnnnnnnnnnnnnnnnctgcatcagggggggtatgcggttgcagcgcttcggcgcagtgtacgagaaaatcctgcctctcttgaggcgcaggcnnnnnnnnnnnnnnnnnnnnnnnnnnnnnnnnnnnnnnnnnnnnnnnnnnnnnnnnnnnnnnnnnnnCTTGCGCAGTATCCAGACAATAAGCGTTTGAACGCAGGGTATTTGAATTCGCTTTTTGTAGAAGCAGTGCAGCATCTAAAACGGGGGAGCGCAGATCTTGCGCGTCAGATGTTTACGTTTCTGATTAATCAGGATGTAGACGGGGTTGCGCCACGTTTATACTTGGCGCACGCGTTTCGTTCTTTGAAACATTTTCCTGAAGCGCTTACCCAGTATCGTGCAGCAAGCGCATTTGCGCCGCACGATCCTGCCCTCAAGTGGTACGAAGCGGCCATGCTTgtagaaatggggtgtctgtcgcaggcggcagcgttgctgtcgnnnnnnnnnnnnnnnnnnnnnnnTGATCAGATTTCGGATCGTTTTCTAGTGATGGGCGCCGTGCGCAAGCACATGGAGGAGGGGGCGTGGGCTCGTGCCGCTTCTGCAGCGCATTTATACCTGAAAACTTTTGGGGGTTCTGTAGaaattcacctgctaatggcagaggttcaccggcgtgcggggcgcgtgaacgtggctttgaaccactacacgcgtgcgatgaAAATAGAACCGAAaaattgttatccgcattatggtcttatggtgtgnnnnnnnnnnnnnnnnnnnnnnnnnnnnnnnnnnnnnnnnnnnnnnnnnnnnnnnnnnnnnnnnnnnnnnnnnnnnnnnnnnnnnnnnnnnnnnnnnnnnnnnnnnnnnnnnnnnnnnnnnnnnnnnnnnnnnnnnnnnnnnnnnnnnnnnnnnnnnnnnnnnnnnnnnnnnnnnnnnnnnnnnnnnnnnnnnnnnnnnnnnnnnnnnnnnnnnnnactgggaatggcagatctcgcacttcgctggtatgaaaaaacccttcttctggatgcagaggacgaagaagcgtgcgtgggactgaTCGCCTGCTACGAGGCGCTCTGCGACGACGCGCGCGCGTACACCCAGTATGGAGcgtacctGTCCcgctggagggacaatcgggttatccgcaaggattttatagcctttcttgagagaacagaaCGGTGGTCCGAAGCGGCGGACCACATCGAGTTGCTCGCCTCGGGTGAGCGnnnnnnnnnnnnnnnnnnnnnnnnnnnnnnnnnnnnnnnnnnnnnnnnnnnnnnnnnnnnnnnnnnnnnnnnnnnnnnnnnnnnnnnnnnnnnnnnnnnnnnnnnnnnnnnnnnnnnnnnnnnnnnnnnnnnnnnnnnnnnnnnnnnnnnnnnnnnnnnnnnnnnnnnnnnnnnnnnnnnnnnnnnnnnnnnnnnnnnnnnnnnnnnnnnnnnnnnnnnnnnnnnnnnnnnnnnnnnnnnnnnnnnnnnnnnnnnnnnnnnnnnnnnaatccgcacactccncgcggtcctagaacagcaaccaggctacaccgcagcgagcgagctcctggcaaaggcctacgcgctggcgagtagccgggcctaggcatgggngcgcttcttcctgaccaatcccccaagaggGAAACGAGCTAGCCACGTTCGGAGCGTGGCGCACCCTAAAAGgcacgccgctttgactcacgcgcgctttggcactcaatgcacatgagcgagtaggggatggcgcgcagccgatcctcggggatagattcgccgcaatcggcgcatttcccgtagcgtncnnnnnnnnnnnnnnnnnnnnnnnnnnnnnnnnnnnnnnnnnnnnnnnnnnnnnnnnnnnnnnnnnnnnnnnnnnnnnnnnnnnnnnnnnnnnnnnnnnnnnnnnnnnnnnnnnnnnnnnnnnnnnnnnnnnnnnnnnnnnnnnnnnnnnnnnnnnnnnnnnnnnnnnnnnnnnnnnnnnnnnnnnnnnnnnnnnnnactaagctcactttcatttcctcaacaaacgactgatccacnnnnnnnnnnnnnnnnnnnnnnnnnnnnnnnnnnnnnnnnnnnnnnnnnnnnnnncacccgacctagagcgtagactgctggagggaccgctccgtccattatacggacgnnnnnnnnnnnnnnnnnnnnnnnnnnnnnnnnnnnnnnnnnnnnnnnnnnnnnnnnnnnnnnnnnnnnnnnnnnnnnnnnnnnnnnnnnnnnnnnnnnnnnnnnnnnnnnnnnnnnnnnnnnnnnnnnnnnnnnnnnnnnnnnnnnnnnnnnnnnnnnnnnnnnnnnnnnnnnnnnnnnnnnnnnnnnnnnnnnnnnnnnnnnnnnnnnnnnnnnnnnnnnnnnnnnnnnnnnnnnnnnnnnctctcccgcggcagaattatgtttcgtgagcgttagattcctctctcgcaggagagaggtgcttgctcctctttagcagcggggttGCTCATCCTTCaaggaggattgagagtcctgccctcagggcgtgcACTCCGCGCGCCAGGCAGGAcaCGCTGAATGCGATCCCGAAGAAGCCAAATACTAAAAAGAAGCGTAGCCCGAATAGAGCCAGGAGGACGAGCGCCGCTCCCTGAATGAGAAGGCGCGCCCCGTCTTTCCTGGAGCCCGTGCGCGCGGTAGGACCCGCCGGATCGGGCGGAGCGTCTGCACTGCTCCAATAACTCCACCCATTTCCAGGAAAACCACCGAAGAAAGTGTACCATCCCTCGAAgtgttcggtttcccgcgcnnnnnnnnnnnnnnnnnnnnnnnnnnnnnnnnnnnnnnnnnnnnnnnnnnnnnnnnnnnnnnnnnnnnnnnnnnnnnnnnnnnnnnnnnnnnnnnnnnnnnnnnnnnnnnnnnnnnnnnnnnnnnnnnnnnnnnnnnnnnnnnnnnnnnnnnnnnnnnnnnnnnnnnnnnnnnnnnnnnnnnnnnnnnnngcgttgatccttttaaactggtcctccgcacacgcgtcgcccggatttttatccggatgatacttaagcgcctgcgcgcgaaaagCTTTCTTAATGTGTTCCTCAGAAGCATCGGcagcaacgcccagtatggcgtaatggtcaggaacagnnnnnnnnnnnnnnnnnnnnnnnnnnnnnnnnnnnnnnnnnnnnnnnnnnnnnnnnnnnnnnnnnnnnnnCGGTAAGTCACGCCCtgttgcgcaAATGACCTGAATTCCCGACTTTTTTGCCCGGACACTAGCGACTGGGTCAAAGGGgacatggctgcccggtacccactccttgtcGACAAGGAGGAGGAAATCATCCCAGGAGagggatgtgagcgccttcgcaTCCTTGTCTGAACGAGGATCCCCCGTGTACACATGCGCAACGTCGGAAAGGTTAATAACCGTCTTTGCAGAATAACGCTCTGCAAGGAGCACAGCGTCGGTGTCGGTGGAAAAACCcggtttccaaccagcagcaacgagCACCnnnnnnnnnnnnnnnnnnnnnnnnnnnnnnnnnnnnnnnnnnnnnnnnnnnnnnnnnnnnnnnnnnnnnnnnnnnnnnnnnnnnnnnnnnnnnnnnnnnnnnnnnnnnnnnATCCAGTCAAGTTCAACGTGTTCGGCCGTGGCATACAGCTCCCTGTCTTCCTGATTATCGCCCTCGTGCGCAGGTTTCGCACAGGCCGGAGAAGACaggctccggcgtagcgcacgataggcgttttgataagtgcgcgcaggtgcaccacnnnnnnnnnnnnnnnnnnnnnnnnnnnnnnnnnnnnnGTATAGGTACcgttgaacggaacgaacgaaccgcccgagaagctctatgtnnnnnnnnnnnnnnnnnnnnnnnnnnnnnnnnnnnnnnnnnnnnnnnnnnnnnnnnnnnnnnnnnnnnnnnnnnnnnnnnnnnnnnnnnnnnnnnnnnnnnnnnnnnnnnnnnnnnnnnnnnnnnnnnnnnnnnnnnnnnnnnnnnnnnnnnnnnnnnnnnnnnnnnnnnnnnnnnnnnnnnnnnnnnnnnnnnnnnnnnnnnnnnnnnnnnnnnnnnnnnnnnnnnnnnnnnnnnnnnnnnnnnnnnnagttgctcccgtacgaccggtgctttacgcgcggtcccccttgtgttccgttccgtcctggtgcttgcggtgtggggtgtttccngcntacaagccgccgatgtggcgcacaatgcggatgtaccttcccgctcGCTGAAGGCGCTCGAGCgtttccgtttttttgtgtatcccnagccgctcgacctttctagtgactttcatgcgnagGCCTTGAAGGGGGAGGCACTGGTTCCTAGCCTTTTCAAGGGAAAGGTGACGCTTTTGAACTTTTGGGCTACGTGGtgtccgccctgtcgtgcggnnnnnnnnnnnnnnnnnnnnnnnnnnnnnnnnnnnnnnnnnnnnnnnnnnnnnnnnnnnnnnnnnnnnnnnnnnnnnnnnnnnnnnnnnnnnnnnnnnnnnnnnnnnnnnnnnnnnnnnnnnnnnnnnnnnnnnnnnnnnnnnnnnnnnnnnnnnnnnnnnnnnnnnnnnnnnnnnnnnnnnnnnnnnnnnnnnnnnnnnnnnnnnnnnnnnnnnnnnnnnnnnnnnnnnnnnnnnnGTGTGGAGTATGACCAACCAGAGCTAGTGGCTCTCTTTAAGGAACTGGCGCGTGACTAGTGTCCCCGGCGTTGTGGGTTCCTTTTTGGCCGGGTTGCTTTCTTTTCTCAGTCCCTGcgtcctgccgcttattccggcgtacgtctctttcatttcgggagaatcGCTCGGTTCTATCCnnnnnnnnnnnnnnnnnnnnnnngtttttctcagcagtgttttttttgtattaggactgncgacggtttttgtgttgttttcaatcgtatttAGCGGAGGGGTGCAGCTTGCAGGTGCGGGTGTGCTCACTGTGCTCACGCGTGTAGCGGGCGTGGGGGTGATACTCCTCGGCTTAAACACAATCTTCGACGTGGTTCCGTTTTTGCGTGTGGAAAGGCGTATGCACACAACGGTGCGACGGGtgggtgtgtttcgtgcgtatCTTTTTGGGTTGCTGTTCGCAACGGGATGGACTCCGTGCGTGGGGCCGATTCTCTCTTCTCTGTTGTTCTATGCGGCGAGTTCTGGGCAGCTGCTCCACGCAGCAGGGCTCCTGACCGTGTATGCACTGGGATTGGGACTTCCCTTCGTGTTTGCAGGGATCTTTTTtggacgtgcggagcgggtgtttgcgtgggtnnnnnnnnnnnnnnnnnnnnnnnnnnnnnnnnnnnnnnnnnnnnnnnnnnnnnnnnnnnnnnnnnnnnnnnnnnnnnnnnnnnnnnnnnnnnnnnnnnnnnnnnnnnnnnnnnnnnnnnnnnnnnnnnnnnnnnnnnnnnnnnnnnnnnnnnnnnnnnnnnnnnnnnnnnnnnnnnnnnnnnnnnnnnnnnnnnnnnnnnnnnnnnnnnnnnnnnnnnnnnnnnnnnnnnnnnnnnnnnnnnnnnnnnnnnnnnnnnnnnnnnnnnnnnnnnnnnnnnnnnnnnnnnnnnnnnnnnnnnnnnnnnnnnnnnnnnnnnnnnnnnnnnnnnnnnnnnnnnnnnnnnnnnnnnnnnnnnnnnnnnnnnnnnnnnnnnnnnnnnnnnnnnnnnnnnnnnnnnnnnnnnnnnnnnnnnnnnnnnnnnnnnnnnnnnnnnnnnnnnnnnnnnnnnnnnnnnnnnnnnnnnnnnnnnnnnnnnnnnnnnnnnnnnnnnnnnnnnnnnnnnnnnnnnnnnnnnnnnnnnnnnnnnnnnnnnnnnnnnnnnnnnnnnnnnnnnnnnnnnnnnnnnnnnnnnnnnnnnnnnnnnnnnnnnnnnnnnnnnnnnnnnnnnnnnnnnnnnnnnnnnnnnnnnnnnnnnnacgcggtttaggtagtcgtacaagcttccgtgttctgaactctgttcccagtgttcgatggattcgactaagnnnnnnnnnnnnnnnnnnnnnnnnnnnnnnnnnnnnnnnnnnnnnnnnnnnnnnnnnnnnnnnnnnnnnnnnnnnnnnnnnnnnnnnnnnnnnnnnnnnnnnnnnnnnnnnnnnnnnnnnnnnnnnnnnnnnnnnnnnnnnnnnnnnnnnnnnnnnnnnnnnnnnnnnnnnnnnncggtacgatgtgattcttttaaatcgactgcnnnnnnnnnnnnnnnnnnnnnnnnnnnnnnnnnnnnnnnnnnnnnnnnnnnnnnnnnnnnnnnnnnnnnnnnnnnnnnnnnnnnnnnnnnnnnnnnnnnnnnnnnnnnnnnnnnnnnnnnnnnnnnnnnnnnnnnnnnnnnnnnnnnnnnnnnnnnnnnnnnnnnnnnnnnnnnnnnnnnnnnnnnnnnnnnnnnnnnnnnnnnnnnnnnnnnnnnnnnnnnnnnnnnnnnnnnnnnnnnnnnnnnnnnnnnnnnnnnnnnnnnnnnnnnnnnnnnnnnnnnnnnnnnnnnnnnnnnnnnnnnnnnnnnnnnnnnnnnnnnnnnnnnnnnnnnnnnnnnnnnnnnnnnnnnnnnnnnnnnnnnnnnnnnnnnnnnnnnnnnnnnnnnnnnnnnnnnnnnnnnnnnnnnnnnnnnnnnnnnnnnnnnnnnnnnnnnnnnnnnnnnnnnnnnnnnnnnnnnnnnnnnnnnnnnnnnnnnnnnnnnnnnnnnnnnnnnnnnnnnnnnnnnnnnnnnnnnnnnnnnnnnnnnngcgtaggggaagtctttttcaaaagagagnATATTGTCGTGGTGTGCTCCACGCCAAGAATAAATTGATTGATCATCGTCCCCTACTACACAGATATTTTGTGTAGCGAGCATTTTCATTAAACGGTACTGCTGtgcactggtgtcttggaattcatccactaaaatgtaatgatagcgacncttatatgaagcaaggatatcaggatattcactgaagatctggattggcagcacgatcaaatcgtcaaagtctaccgcattaaataaTTTCAACGCagtctgatattcgtgccaaagagcacgttccttgtgctgtagctcttttaagttcnnnnnnnnnnnnnnnnnnnnnnaaaagagcgtgctcacgcagtttgtgtcaagaacttctggcaggagatgtacttcctttgctgctnnnnnnnnnnnnnnnnnnnnnnnnnnnnnnnnnnnnnnnnnnnnnnnnnnnnnnnnnnnnnnnnnnnnnnnnnnnnnnnnnnnnnnnnnnnnnnnnnnnnnnnnnnnnnnnnnnnnnnnnnnnnnnnnnnnnnnnnnnnnnnnnnnnnnnnnnnnnnnnnnnnnnnnnnnnnnnnnnnnnnnnnnnnnnnnnnnnnnnnnnnnnnnnnnnnnnnnnnnnnnnnnnnnnnnnnnnnnnnnnnnnnnnnnnnnnnnnnnnnnnnnnnnnnnnnnnnnnnnnnnnnnnnnnnnnnnnnnnnnnnnnnnnnnnnnnnnnnnnnnnnnnnnnnnnnnnnnnnnnnnnnnnnnnnnnnnnnnnnnnnnnnnnnnnnnnnnnnnnnnnnnnnnnnnnnnnnnnnnnnnnnnnnnnnnnnnnnnnnnnnnnnnnnnnnnnnnnnnnnnnnnnnnnnnnnnnnnnnnnnnnnatgtgtATGCCGCGCAGGGTGCGGCCCCCATGCCTGCTCCTACGGATAATCCTGCTTGTGATGCGCACATGTCGCATGACGTCATAGCGCGTACTGCCCAAGCAGTTTTTGGTATTCGTGCGCTGTTTCCTTGGCAGCGCTTGGTAATTGCTAACATactggatgcggcgcatgcgtgtacnnnnacaactcnnnnnnnnnnnnnnnnnnnntctcaaaccgatgctacgagggtgactcatgtggatgacgcgcagcctgaggataatttcgtcggtgccatgcaagacacacgttttgatcaggatggcgtgtcacgcgcacatcaagtggtgcnnnnnnnnnnnnnnnnnnnnnnnnnnnnnnnnnnnnnnnnnnnnnnnnnnnnnnnnnnnnnnnnnnnnnnnnnnnnnnnnnnnnnnnnnnnnnnnnnnnnnnnnnnnnnnnnncgGATGCAGGCAGTGGGATTTTCGGTCATCTTGTTACGTGGTGGACTGAATGCGCAAGAGCGCGCGTACATGTATGCGCAGTTGGATAGGTGTGCTGAGGCGTATGGCCGGATGCGAGGCGTTACGCCTCCTGCACACCnnnnnnnnnnnnnnnnnnnnnnnnnnnnnnnnnnnnnnnnnnnnnnnnnnnnnnnnnnnnnnnnnnnnnnnnnnnnnnnnnnnnnnnATGTGGATGAACGTCTTGCTCAGAAAAGGACAGAAAGTCGAAGAGGTGTATGCatcatcgcaagtccagagatactcacacaacctgcgctgcgcgcacgcgtgcgtgcatgtcgcgttgcgcatttggttattgatgaagcgcactGTGTgtccgagtggggagattCgtttcgtcctgattacgtgcgactaggcgaattggtgcaggatcttgcgcctcaagtgGTGACTGCATTTACGGCGACTGCAAGTCAAACAGTGCTTGCGCGCATCATGGAAGTGCTGTTTGGCGGTCGtgcgcacgtgttgcagggaacnnnnnnnnnnnnnnnnnnnnnnnnnnnnnnnnnnnnnnnnnnnnnnnnnnnnnnnnnnnnnnnnnnnnnnnnnnnnnnnnnnnnnnnnnnnnnnnnnnnnnnnnnnnnnnnnnnnnnnnnnnnnnnnnnnnnnnnnnnnnnnnnnnnnncGTGTCTTTCTGACAcACAGATACGTTTTTATCACGCAGGGTTGCAGAGGGAAGAAAAAGAAACAGTGGAGCGATGGTTTCATACCCATGATTCTGCCGTTTTGGTAACTACTTGCGCGTGGGGAATGGGAGTTGATAAGCCGAATGTACGTACGGTCATTCACGTGGATGCGCCACTGACTGTGGAGGCGTACGTACAGGAGGTTGGAAGAGCAGGACGGGACGGAATGCGTGCAGACGCATTTTTATTGTGGTCACCTCGAGATGCTCGCTCGATAGAAACACTGCCGCATGCACAACGGGtgcgtgcgcacgtgttgcgccactttgctgaaagcggacgttgtcgccgcgcagttttacttgagtctttgggggaacagaatgtgtgtgccggatgtgatgtgTGTGCAGGCactgcacgttttgtatgtgaggATGTAGAAGCGCTCTTACAGTTTTTGAAAAAGAATGCGCGCAGATTCACTGTATCATCGTTGGTGCAGCACCTCGCGCTACATCAGAAAGTGCTCAGTGTGGCGGATGTACATGCCTTGCTATATTACGCGCTCGAAACAGGACGTGTGAAaaaaaacattcaCTCTTGTGGGGTGATGTCCTGTATGTTGCACGTTAACGATTCTGCGAGCAAATCGTATCTGCAGGAAAGCAAGAAGGatggcgagaacatacagttgccttgtatattccgcgagttacgcgcatttttatggccgagtggttagcattacttctacaggttttttatgcaattttatatactgagcnnnngttcatgtgtctccgatacggtgtggtctaggttccgtaccgtgcgggcacggaacacatcgagcggacgcgtctgttcgtggaggatnnnnnnnnnnnnnnnnnnnnnnnnnnnnnnnnnnnnnnnnnnnnnnnnnnnnnnnnnnnnnnnnnnnnnnnnnnnnnnnnnnnnnnnnnnnnnnnnnnnnnnnnnnnnnnnnnnnnnnnnnnnnnnnnnnnnnnnnnnnnnnnnnnnnnnnnnnnnnnnnnnnnnnnnnnnnnnnnnnnnnnnnnnnnnnnngtaggcggttatgcggcgcttgtgcacgagatacaacggttgcgtgcggagtcgaagaacgcattggtactgnnnnnnnnnnnnnnnnnnntaggtacgctgtATTCTACCCTCTTTAGAGGGCGTGCGGACGCGGTGCTGATGnnnnnnnnnnnnnnnnnnntttttacccttggcaatcacgaatttgataatgggaatgagggactcaaagaatttctGCACTATTTGGAAGTGCCAGTTCTCTCTGCAAAtgtggttcctaatgctgccagcacgttgcatggcttgtggaagccgagcgctattgtggannnnnnnnnnnnnnnnnnnnnnnnnnnnnnnnnnnnnnnnnnnnnnnnnnnnnnnnnnnnnnnnnnnnnnnnnnnnnnnnnnnnnnnnnnnnnnnnnnnnnnnnnnnnnnnnnnnnnnnnnnnnnnnnnnnnnnnnnnnnnnnnnnnnnnnnnnnnnnnnnnnnnnnnnnnnnnnnnnnnnnnnnnnnnnnnnnnnnnnnnnnnnnnnnnnngtattgacgncatcgtgtcaggtgatacccactaccttttgggggatgaatcactcggacggctaggtcttccggtagttggtgaatatcccagaaagattatgtcccctgcaggggagcctgtgtatgtggtnnnnnnnnnnnnnnnnnnnnnnnnnnnnnnnnnnnnnnnnnnnnnnnnnnnnnnnnnnnnnnnnnnnnnnnnnnnnnnnnnnnnnnnnnnnnnnnnnnnnnnnnnnnnnnnnnnnnnnnnnnnnnnnnnnnnnnnnnnnnnnnnnnnnnnnnnnnnnnnnnnnnnnnnnnnnnnnnnnnnnnnnnnnnnnnnnnnnnnnnnnnnnnnnnnnnnnnnnnnnnnnnnnnnnnnnnnnnnnnnnnnnnnnnnnnnnnnnnnnnnnnnnnnnnnnnnnnnnnnnnnnnnnnnnnnnnnnnnnnnnnnnnnnnnnnnnnnnnnnnnnnnnnnnnnnnnnnnnnnnnnnnnnnnnnnnnnnnnnnnnnnnnnnnnnnnnnnnnnnnnnnnnnnnacgatgctctcagacattcaaAGTTTTGGTGCGGGGAAGGTAGATTGCGTAATTCAAAATGCAGGCGGTGCGCGGTCAAATATTCAGCCTGGTGAGATTACGTATAATGACGCATACACGCTCCTCCCCTTTAGTAACACGCTGGTGTTGGTGGACGTCAGCGGTGCAGAGTTGAAACAAATTATAGAGGATGCATTGCAGTTTGCACTTGGTGATGGTTCCACGGGAGCCTTCCCCTATGGGGCGGGTGTCCGGTATGAnnnnnnnnnnnnnnnnnnnnnnnnnnnnnnnnnnnnnnnnnnnnnnnnnnnnnnnnnnnnnnnnnnnnnnnnnnnnnnnnnagatgagcgcGCGCCGTATCGGTTGGGTGTGAACTCGTACATTGCGCGGGGAAAAGACGGATATAAAACGCTCGGAGAGATTGTCAGTACGCGCGGAGCTGAGGATACGTATCTGCGTGATGCGGAGTCTTTGATTAAGTTTTTGCGTGCGCATAAAAATTnnnnnnnnnnnnnnnnnnnnnnnnnnnnnnnnnnnnnnnnnnnnnnnnnnnnnnnnnnnnnnnnnnnnnnnnnnnnnnnnnnnnnnnnnnnnnnnnnnnnnnnnnnnnnnnnnnnnnnnnnnnnnnnnnnnnnnnnnnnnnnnnnnnnnnnnnnnnnnnnnnnnnnnnnnnnnnnnnnnnnnnnnnnnnnnnnnnnnnnnnnnnnnnnnnnnnnnnnnnnnnnnnnnnnnnnnnnnnnnnnnnnnnnnnnnnnnnnnnnnnnnnnnnnnnnnnnnnnnnnnnnnnnnnnnnnnnnnnnnnnnnnnnnnnnnnnnnnnnnnnnnnnnnnnnnnnnnnnnnnnnnnnnnnnnnnnnnnnnnnnnnnnnnnnnnnnnncgttattttgttgctgtttttgattctctcacgcctacctttcggcacgtacagtacccagcctataaggcaaaaagggataagacttctgcagagctttatgcgcaaattccccttatcgaagaaatccnnnnnnnnnnnnnnnnnnnnnnnnnnnnnnnnnnnnnnnttgaagctgacgacctcattgcaaccctagcaaaacgagttgcggctgagcactgtcatgttgtgattatctcctcagataaagatgtacttcagcttgtgtgtgatacggtgcaagtgctcagacttgacatagatcataagtgganannnnnnnnnnnnnnnnnnnnnnnnnnnnnnnnnnnnnnnnnnnnnnnnnnnnnnnnnnnnnnnnnnnnnnnnnnnnnnnnnnnnnnnnnnnnnnnnnnnnnnnnnnnnnnnnnnngtcctaagacggctgcacatcttctccactgttttggcacacttGATGGTATnnnnnnnnnnnnnnnnnnnnnnnnnnnnnnnnnnnnnnnnnnnnnnnngtgtGGGAAGAAAGATGCATTTTTTTCTCGTTCACTCATTGAGTTGCGTGACGATGTACCATGTGTTTTTTCGCTCGAAGATTCCTGTTGTATTCCGCTCGATGTAACGTCTGCTGCACGTATTTTTGTGCGAGAAGGATTGCATGCGCTTGCACaacaatatcgtgcttgtgtgcaagaaatagatacagannnnnnnnnnnnnnnnnnnnnnnnnnnnnnnnnnnnnnnnnnnacgtctggncgatgtgcaaatgagtgtttcttatctcaggtagaagggagggctagtacaccgGAGGTGAACTCCGTATTGAAGTCGGAGTTGAAGACGAGTGCTGTGTCTGGCGCCATACCTATAGAAAATAGAGATCTTAGGCAGGATGTTATGCTTGCACGCAGTGCAGGTCATTATCGTGGTGTTACTGACCCTGTAGAACTTAAACGTAttattgattgcgcgtgtgcgaatggtgtggtcgcgtttgattgtgaaacggatggatnnnnnnnnnnnnnnnnnnnnnnnnnnnnnnnnnnnnnnngctttcaggaagcagaggctttttatgttcctcttattgttccggacgtttctcttcataccgagtcaactcagtgtACATGTGCACGTAACACTAATGTcgagactgaaaaggagtgcacagaacagcatgGGGTATCTGCATCTGCTGTGCAGGATCCGGCATATGTCCAAGCTGTCATGCACCAGCTTCGACGTCTTTGGAATGATGAGACGCTCACACTTGTTATGCATAATGGAAAGTTTGATTATCACGTTATGCATCGTgcaggcgtttttgagcactgtgcatgtaatnnnnnnnnnnnnnnnnnnnnnnnnnnnnnnnnnnnnnnnnnnnnnnnnnnnnnnnnnnnnnnnnnnnnnnnnnnnnnnnnnnnnnnnnnnnnnnnnnnnnnnnnnnnnnnnnnnnnnnnnnnnnnnnnnnnnnnnnngcacgtcccttatgagtGTGCAGTCCGCTATGCAGCGGAGGATGCAGATATTACTTTTCGTTTATACCATTATTTAAAACTCCGCTTGGAAACAGCAGGATTGCTTTCTGTGTTTGAGACCATAGAAATGCCGCTTTTGCCTATCCTAGCAcgtatggaagaagtgggnnnnnnnnnnnnnnnnnnnnnnnnnnnnnnnnnnnnnnnnnnnnnnnnnnnnnnnnnnnnnnnnnnnnnnnnnnnnnnnnnnnnnnnnnnnnnnnnnnnnnnnnnnnnnnnnnnnnnnnnnnnnnnnnnnnnnnnnnnnnnnnnnnnnnnnnnnnnnnnnnnnnnnnnnnnnnnnnnnnnnnnnnnnnnnnnnnnnnnnnnnnnnnnnnnnnnnnnnnnnnnnnnnnnnnnnnnnnnnnnnnnnnnnnnnnnnnnnnnnnnnnnnnnnnnnnnnnnnnnnnnnnnnnnnnnnnnnnnnnnnnnnnnnnnnnnnnnnnnnnnnnnnnnnnnnnnnnnnnnnnnnnnnnnnnnnnnnnnnnnnnnnnnnnnnnnnnnnnnnnnnnnnnnnnnnnnnaacattcccattaaaagCACAGAAGGAAGAAAAATAAGGCAGGCGTTTCAAGCTACTGTTGGGCATGAGTTAATTTCGGCAGACTATACACAAATAGAGCTGGTCGTGTTGGCCCATCTATCTCAAGATAGAAATCTTCTCAATGCATTTCnnnnnnnnnnnnnnnnnnnnnnnnnnnnnnnnnnnnnnnnnnnnnnnnnnnnnnnnnnnnnnnnnnnnnnnnnnnnGAGAAGAATCGCAAAAACTATTAACTTTGGAATCGTGTATGGAATGAGCGCTTTTAGATTGAGTGACGAACTTAAAATTTCTCAGAAGGAAGCGCAGAGCTTCATTTACCGTTATTTTGAAACGTACCCGGGGGTGTATGCttttagtacannggttgcagagcagacacgtaaaaccggctatgtgactagcttggctggaagacgacgctacatccgtactatcgatagtcgcaatacgctnnnnnnnnnnnnnnnnnnnnnnnnnnnnnnnnnnnnnnnnnnnnnnnnnnnnnnnnnnnnnnnnnnnnnnnnnnnnnnnnnnnnnnnnnnnnnnnnnnnnnnnnnnnnnnnnnnnnnnnnnnnnnnnnnnnnnnnnnnnnnnnnnnnnnnnnnnnnnnnnnnnnnnnnnnnnnnnnnnnnnnnnnnnnnnnnnnnnnnnnnnnnnnnnnnnnnnnnnnnnnnnnnnnnnnnnnnnnnnnnnnnnnnnnnnnnnnnnnnnnnnnnnnnnnnnnnnnnnnnnnnnnnnnnnnnnnnnnnnnnnnnnnnnnnnnnnnnnnnnnnnnnnnnnnnnnnnnnnnnnnnnnnnnnnnnnnnnnnnnnnnnnnnnnnnnnnnnnnnnnnnnnnnnnnnnnnnnnnnnnnnnnnnnnnnnnnnnnnnnnnnnnnnnnnnnnnnntgggagaatatgagagaagcgggaaggagaATGCACGCAACCGCCCAAAACGATTTCATTATTTGACGTGGTTCTTCTCCCGGTGCAACGCTTTTTTGCGAATAGGAAGCGATGATGAGCGnnnnnnnnnnnnnnnnnnnnnnnnnnnnnnnnnnnnnnnnngctgccaacagcgccataacgatgtacgcgcagggcagtgtnngaataattgcgataatcacctcagcgggtgtattccccgcgcgcagcaagtacgcggcaggaaggaggtggtgcgtttgtaaatagagcccgtagttccctaagacgatgaaggagccgtacgtacnnnnnnnnnnnnnnnnnnnnnnnnnnnnnnnnnnnnnnnnnnnnnnnnnnnnnnnnnnnnnnnnnnnnnnnnnnnnnnnnnnnnnnnnnnnnnnnnnnnnnnnnnnnnnnnnnnnnnnnnnnnnnnnnnnnnnnnnnnnnnnnnnnnnnnnnnnnnnnnnnnnnnnnnnnnnnnnagaaggatccatccacgttgccataagaaagaagttttgtagcattttccctatcgcggtgatacccgtctcgataagatacacggttggtcctgcacacaagaagaaAACAAGAACnnnnnnnnnnnnnnnnnnnnnnnnnnnnnnnnnnnnnnnnnnnnnnnnnnnnnnnnnnnnnnnnnnnnnnnnnnnnnnnnnnnnnnnnnnnnnnnnnnnnnnnnnnnnnnnnnnnnnnnnnnnnnnnnnnnnnnnnnnnnnnnnnnnnnnnnnnnnnnnnnnnnnnnnnnnnnnnnnnnnnnnnnnnnnnnnnnnnnnnnnnnnnnnnnnnnnnnnnnnnnnnnnnnnnnnnnnnnnnnnnnnnnnnnnnnnnnnnnnnnnnnnnnnnnnnnnnnnnnnnnnnnnnnnnnnnnnnnnnnnnnnnnnnnnnnnnnnnnnnnnnnnnnnnnnnnnnnnnnnnnnnnnnnnnnnnnnnnnnnnnnnnnnnnnnnnnnnnnnnnnnnnnnnnnnnnnnnnnnnnnnnnnnnnnnnnnnnnnnnnnnnnnnnnnnnnnnnnnnnnnnnnnnnnnnnnnnnnnnnnnnnnnnnnnnnnnnnnnnnnnnnatacaaaatatccgccgccatggtggacgtaaaaatcatggagccccacgtaaagttggaatAGCGTGCGGTGCGCGTAGTACCCAGATACACCGCACCATACCGTGAAAAAGCAATAGTCAGCGTnnnnnnnnnnnnnnnnnnnnnnnnnnnnnnnnnnnnnnnnnnnnnnnnnnnnnnnnnnnnnnnnnnnnnnnnnnnnnnnnnnnnnnnnnnnnnnnnnnnnnnnnnnnnnnnnnnnnnnnnnnnnnnnnnnnnnnnnnnnnnnnnnnnnnnnnnnnnnnnnnnnnnnnnnnnnnnnnnnngcatGATCTGCCCTATGTGTTTTCAGGAGCGTCTGAACTGCTTCGTAATATTCTTTTGCATATCGGTACATTTTCAGTCCATAATCTCCAAACGAGATACCAAGCGCCTGTTTGTAGCTCGTCCACAAnnnnnnnnnnnnnnnnnnnnnnnnnnnnnnnnnnnnnnnnnnnnnnnnnnnnnnnnnnnnnnnnnnnnnnnnnnnnnnnnnnnnnnnnnnnnnnnnnnnnnnnnnnnnnnnnnnnnnnnnnnnnnnnnnnnnnnnnnnnnnnnnnnnnnnnnnnnnnnnnnnnnnnnnnnnnnnnnnnnnnnnnnnnnnnnnnctgccatcncaaagcaggaagttntctggggttagatcgacatgcgtaagtacgcaCGGTTTGTCCACTGAATCGACAATAGCCAAGAGTGTATTCATTTTTTCTCTCACTGAACGATagtcagcgtaaagaatactgtactggtcgagggnnnnnnnnnnnnnnnnnnnnnnnnnnnnnnnnnnnnnnnnnnnnnnnnnnnnnnnnnnnnnnnnnnnnnnnnnnnnnnnnnnnnnnnnnnnnnnnnnnnnnnnnnctgcnnnnnnnnnagggtttgcactgcggcagtctttgtggaagacggtgattttaataccacgggcaggctcaaggtgaacgagagtgtcacaaatattaaggggcttaattgcttcatacacggcagcttcttggaagcgattgacaagnnnnnnnnnnnnnnnnnnnnnnnnnnnnnnnnnnnnnnnnnnnnnnnnnnnnnnnnnnnnnnnnnnnnnnnnnnnnnnnnnnnnnnnnnnnnnnnnnnnnnnnnnnnnnnnnntttcgggttggttgaaaacaTGGGAAATGACGCGCATCCACTCGTTATCGCTCGAAACCATGTACGCAAAATCAAACTTGCGCAGCTCATCGAGCGACTCAAATTCGTAGATAAGGTTGTCAGCCTGTTTGTTGGCAAACATGGTAAGGGATTTTGTATTGCGCATAAAGAcgtcttcccagaaccacgcnnnnnnnnnnnnnnnnnnnnnnnnnnnnnnnnnnnnnnnnnnnnnnnnnnnnnnnnnnnnnnnnnnnnnnnnnnnnnnnnnnnnnnngatccacccacttctgccaccaattttcacggcggtaattttgtcgtacaatccgGTTTTTAACACCCATTCCTTTGTTTTCCCTTGTGTCTTGACaGCCGTATACnnnnnnnnnnnnnnnnnnnnnnnnnnnnnnnnnnnnnnnnnnnnnnnnnnnnnnnnnnnnnnnnnnnnnnnnnnnnnnnnnnnnnnnnnnnnnnnnnnnnnnnnnnnnnnnnnnnnnnnnnnnnnnnnnnnnnnnnnnnnnnnnnnnnnnnnnnnnnnnnnnnnnnnnnnnnnnnnnnnnnnnnnnnnnnnnnnnnnnnnnnnnnnnnnnnnnnnnnnnnnnnnnnnnnnnnnnnnnnnnnnnnnnnnnnnnnnnnnnnnnnnnnnnnnnnnnnnnnnnnnnnnnnnnggagtagcgtatgtaagAGGCACGCAACGGGAGCCAAAGCCTGCAGCCATTATTACCGCATTATGAACCCGGCAAGACTGATACTGACTATGTGCAAGGGGGGTGAGAGAACGCGTCCGCGCACCAAAGGTCCCGTGGGTTTCAAGGAGTCnnnnnnnnnnnnnnnnnnnnnnnnnnnnnnnnnnnnnnnnnnnnnnnnnnnnnnnnnnnnnnnnnnnnnnnnnnnnnnnnnnnnnnnnnnnnnnnnnnnnnnnnnnnnnnnnnnnnnnnnnnnnnnnnnnnnnnnnnnnnnnnnnnnnnnnnnnnnnnnnnnnnnnnnnnnnnnnnnnnnnnnnnnnnnnnnnnnnnnnnnnnnnnnnnnnnnnnnnnnnnnnnnnnnnnnnnnnnnnnnnnnnnnnnnnnnnnnnnnnnnnnnnnnnnnnnnnnnnnnnnnnnnnnnnnnnnnnnnnnnnnnnnnnnnnnnnnnnnnnnnnnnnnnnnnnnnnnnnnnnnnnnnnnnnnnnnnnnnnnnnnnnnnnnnnnnnnnnnnnnnnnnnnnnnnnnnnnnnnnnnnnnnnnnnnnnnnnnnnnnnnnnnnnnnnnnnnnnnnnnnnnnnnnnnnnnnnnnnnnnnnnnnnnnnnnnnnnnnnnnnnnnnnnnnnnnnnnnnnnnnnnnnnnnnnnnnnnnnnnnnnnnnnnnnnnnnnnnnnnnnnnnnnnnnnnnnnnnnnnnnnnnnnnnnnnnnnnnnnnnnnnnnnnnnnnnnnnnnnnnnnnnnnnnnnnnnnnnnnnnnnnnnnnnnnnnnnnnnnnnnnnnnnnnnnnnnnnnnnnnnnnnnnnnnnnnnnnnnnnnnnnnnnnnnnnnnnnnnnnnnnnnTGCTACAACGAGCACCGCATGCTTACGTAGCGCGATTCGCTTCTCTAGGTGAGCCAAAAGCCCATTTGGACCGTCAAGGTCAAAGCTCACTTCAGGGATGAGTACGAAGTTTGTCTCATGGCTCGCAATcgccgtgtacgtagcgatgaatnnnnnnnnnnnnnnnnnnnnnnnnnnnnnnnnnnnnnnnnnnnnnnnnnnnnnnnnnnnnnnnnnnnnnnnnnnnnnnnnnnnnnnnnnnnnnnnnnnnnnnnnnnnnnnnnnnnnnnnnnnnnnnnnnnnnnnnnnnnnnnnnnnnnnnnnnnnnnnnnnnnnnnnnnnnnnnnnnnnnnnnnnnnnnnnnnnnnnnnnnnnnnnnnnnnnnnnnnnnnnnnnnnnnnnnnnnnnnnnnnnnnnnnnnnnnnnnnnnnnnnngttgagccgctcgataccatcgacgatgtcaaccacacggttccccccaccgcgcgaggtgcctagcagcgaaccacctgttttgtggcagttatcgatgacctcaggggtgagcggcaggatatcgaagttgtaatcgnnnnnnnnnnnnnnnnnnnnnnnnnnnnnnnnnnnnnnnnnnnnnnnnnnnnnnnnnnnnnnnnnnnnnnnnnnnnnnnnnnnnnnnnnnnnnnnnnnnnnnnnnnnnnnnnnnnnnnnnnnnnnnnnnnnnnnnnnnnnnnnnnnnnnnnnnnnnnnnnnnnnnnnnnnnnnnnnnnnnnnnnnnnnnnnnnnnnnnnnnnnnnnnnnnnnnnnnnnnnnnnnnnnnnnnnnnnnnnnnnnnnnnnnnnnnnnnnnnnnnnnnnnnnnnnnnnnnnnnnnnnnnnnnnnnnnnnnnnnnnnnnnnnnnnnnnnnnnnnnnattttgctttcccccaatgactctatagaaaaatcgtactctttaggaagatgtgccatggggtcctccgcgtaccgcgtgcgtagagactccctcaggctaacgcaatggggtgannnnnnnnnnnnnnnnnnnnnnnnnnnnnnnnnnnnnnnnnnnnnnnnnnnnnnnnnnnnnnnnnnnnnnnnnnnnnnnnnnnnnnnnnnnnnnnnnnnnnnnnnnnnnnnnnnnnnnnnnnnnnnnnnnnnnnnnnnnnnnnnnnnnnnnnnnnnnnnnnnnnnnnnnnnnnnnnnnnnnnnnnnnnnnnnnnnnnnnnnnnnnnnnnnnnnnnnnnnnnnnnnnnnnnnnnnnnnnnnnnnnnnnnnnnnnnnnnnnnnnnnnnnnnnnnnnnnnnnnnnnnnnnnnnnnnnnnnnnnnnnnnnnnnnnnnnnnnnnnnnnnnnnnnnnnnnnnnnnnnnnnnnnnnnnnnnnnnnnnnnnnnnnnnnnnnnnnnnnnnnnnnnnnnnnnnnnnnnnnnnnnnnnntctgtacctcctgccccatgatggtgcggcaggagcactgcatnnnnnnnnnnnnnnnnnnnnnnnnnnnnnnnnnnnnnnnnnnnnnnnnnnnnnnnnnnnnnnnnnnnnnnnnnnnnnnnnnnnnnnnnnnnnnnnnnnnnnnnnnnnnnnnnnnnnnnnnnnnnnnnnnnnnnnnnnnnnnnnnnnnnnnnnnnnnnnnnnnnnnnnnnnnnnnnnnnnnnnnnnnnnnnnnnnnnnnnnnGGATACCGCGGTGATCGCGCAGCGCAGGGGGTAAACTACGCGCTAGCTTATCAAGCACAGCACACGTTGTGTGCGTGATCCGTATCCCCCnnnnnnnnnnnnnnnnnnnnnnnnnnnnnnnnnnnnnnnnnnnnnnnnnnnnnnnnnnnnnnnnnnnnnnnnnnnnnnnnnnnnnnnnnnnnnnnnnnnnnnnnnnnnnnnnnnncccttctagcgcagcaggctgcgcgcgcagaaactcttcgatggcgcgtaatcctgaaataaccatacggagtaagtgttctcctccttgtatcgcaGCCAGGCGCGCTTTGCAATAGCGCTACTGACTGGAAGGAGCGACTGCACAATTGtatcggcaatgtgcagttgtgtgtctttgccggaatgcgcgtgtgctgtggggctgctgatggcagctgccagtCTGCGTCGGTCAAGTGGAGGTACcgggnnnnnnnnnnnnnnnnnnnnnnnnnnnnnnnnnnnnnnnnnnnnnnnnnnnnnnnnnnnnnnnnnnnnnnnnnnnnnnnnnnnnnnnnnnnnnnnnnnnnnnnnnnnnnnnnnnnnnnnnnnnnnnnnnnnnnnnnnnnnnnnnnnnnnnnnnnnnnnnnnnnnnnnnnnnnnnnnnnnnnnnnnnnnnnnnnnnnnnnnnnnnnnnnnnnnnnnnnnnnnnnnnnnnnnnnnnnnnnnnnnnnnnnnnnnnnnnnnnnnnnnnnnnnnnnnnnnnnnnnnnnnnnnnnnnnnnnnnnnnnnnnnnnnnnnnnnnnnnnnnnnnnagggactATTGGGGTGTGTATTCCCCTGAGTGCGTGGCTATGGTGACGCTATGAACGAACAGCTCTCAACATTTGATAAATTGGTGGAAACGCTCTCCactgaggacgcgcagcgtatgcttgccgatatcgggaaagcacTGCGTACTAGtgaagctnnnnnnnnnnCTCCCGCGAAAGTCAAGAAAAAGCGCGCCTTTGCTCGCTGGGATACCAGAGAGGATAGCnnnnnnnnnnnnnnnnnnnnnnnnnnnnnnnnnnnnnnnnnnnnnnnnnnnnnnnnnnnnnnnnnnnnnnnnnnnnnnnnnnnnnnnnnnnnnnnnnnnnnnnnnnnnnnnnnnnnnnnnnnnnnnnnnnnnnnnnnnnnnnnnnnnnnnnnnnnnnnnnnnnnnnnnnnnnnnnnnnnnnnnnnnnnnnnnnnnnnnnnnnnnnnnnnnnnnnnnnnnnnnnnnnnnnnnnnnnnnnnnnnnnnnnnnnnnnnnnnnnnnnnnnnnnnnnnnnnnnnnnnnnnnnnnnnnnnnnnnnnnnnnnnnnnnnnnnnnnnnnnnnnnnnnnnnnnnnnnnnagatggtactccttctggtttgcgtgcgtccctccttaaaagaatggacgtcgcccttgccaccctgagtnnnnnnnnnnnnnnnnnnnnnnnnnnnnnnnnnnnnnnnnnnnnnnnnnnnnnnnnnnnnnnnnnnnnnnnnnnnnnnnnnnnnnnnnnnnnnnnnnnnnnnnnnnnnnnnnnnnnnnnnnnnnnnnnnnnnnnnnnnnnnnnnnnnnnnnnnnnnnnnnnnnnnnnnnnnnnnnnnnnnnnnnnnnnnnnnnnnnnnnnnnnnnnnnnnnnnnnnnnnnnnnnnnnnnnnnnnnnnnnnnnnnnnnnnnnnnnnnnnnnnnnnnctgcgcacgctgctgcctttcttaaggaggcgagtgcagcgctcgttgttatcaaggatttatcacacagcattcctattgaggnnnnnnnnnnnnnnnnnnnnnnnnnnnnnnnnnnnnnnnnnnnnnnnnnnnnnnnnnnnnnnnnnnnnnnnnnnnnnnnnnnnnnnnnnnnnnnnnnnnnnnnnnnnnnnnnnnnnnnnnnnnnnnnnnnnnnnnnnnnnnnnnnnnnnnnnnnnnnnnnnnnnnnnnnnnnnnnnnnnnnnnnnnnnnnnnnnnngttgactcatcggccatgggaagggttttggtatcaGCTTGTGTTTAGGCGAGAGATGTCTTTTGTCTTTTTAAAAAATTTGTTCGAAGATGCGTATGCGCGCGTCGTTTCACCGCCGCTGAACGTTATTCTTGCTGAGGGCAGtttctatnnnnnnnnnnnnnnnnnngagtacactgacgcggtaaatgtactggaacagatgggagcaaagannnnnnnnnnnnnnnnnnnnnnnnnnnnnnnnnnnnnnnnnnnnnnnnnnnnnnnnnnnnnnnnnnnnnnnnnnnnnnnnaccatgaaaagtaagggtattctagnnnnnnnnnnnnnnnnnnnnnnnnCTGAGGCGACTGTCTTGCTTGATGCAACCTTAAAGGCATTTCATTGCATTGGCGCTATGTTGGCGAGTATgatcggggcgtcgaaaacaagtgcctacgcaannnnnnnnnnnnnnnnnnnnnnnnnnnnnnnnnnnnnnnnnnnnnnnnnnnnnnnnnnnnnnnnnnnnnnnnnnnnnnnnnnnnnnnnnnnnnnnnnnnnnnnnnnnnnnnnnnnnnnnnnnnnnnnnnnnnnnnnnnnnnnnnnnnnnnnnnnnnnnnnnnnnnnnnnnnnnnnnnnnnnnnnnnnnnnnnnnnnnnnnnnnnnnnnnnnnnnnnnnnnnGGTTAGTTCTCAATCAGCGGTTGGTGCACGGGCTTTGTTTACTGCAGCTTTCTCGGGGGGATTTGAAGACCGAGGTTCTCCGTGCCGTGCAACGCAATCCCCTTTTAGAAATACGTCCTTCGTCCGCTCGTCGCACTGGGAnnnnnnnnnnnnnnnnnnnnnnnnnnnnnnnnnnnnnnnnnnnnnnnnnnnnnnnnnnnnnnnnnnnnnnnnnnnnnnnnnnnnnnnnnnaggttgacaatatcnnnnnnnnnnnnnnnnnnnnnnnnnnnnnnnnnnnnnnnnnnnnnnnnnnnnnnnnnnnnnnnnnnnnnnnnnnnnnnnnnnnnnnnnnnnacggttttttttccatatctcctgcgatctttcagaacatgtgtgnnnnnnnnnnnnnnnnnnnnnnnnnnnnnnnnnnnnnnnnnnnnnnnnnnnnnnnnnnnnnnnAGCCACAAGGGTGTGCTGTTTTTAATTTTAAAGAATCATTGGCAGTCCAAGCGCGGCTGCGCTTTGAGCGTTTCTCTGATCCcttgtatcgatgtgttatcaatctactaacacaccacagcgaattGCTTTTCTGCTCTGACAATATGTGTGATGGACGCGTATCCGTGCACGCGCnnnnnnnnnngattaatagtatggggCTTTGTGTGCAGAAGGTTTCGTCAAATGACGTGAAAGATATTTTGCTCCTCATTAAGGAACTACATCCCTTTCCAGGTAAGTGTGTGTCGAnCGCTCAGCGGGCTgacACGAACATGTTGCTTGAACCAGATGTAttgattacaaaaactgnnnnnnnnnnnnnnnnnnnnnnnnnnnnnnnnnnnnnnnnnnnnnnnnnnnnnnnnnnnnnnnnnnnnnnnnnnnnnnnnnnnnnnnnnnnnnnnnnnncgttgaaagnnnnnnnnnnnnnnnnnnnnnnnnnnnnnnnnnnnnnnnnnnnnnnnnnnnnnnnnnnnnnnnntatcgcgaaaacaatagttcaCTACCAATGCGGTTTCTTTGATCACGGACCTGCGAAGCTCACGCCCCTGAGAATGACagatgttGCACACAGGACCGGATTGAGCGTTTCAACAGTGTCCCGCATAGTGCGCGACAAATGGCTCCAGTACGGATCGCAACACTTTTCCTTAaggtactttttctcccctcgggttttatccaccgaagaatatcgtgatcggtccagtcttggacnnnnnnnnnnnnnnnnnnnnnnnnnnnnnnnnnnnnnnnnnnnnnnnnnnnnnnnnnnnnnnnnnnnnnnnnnnnnnnnnnnnnnnnnnnnnnnnnnnnnnnnnnnnnnnnnnnnnnnnnnnnnnnnnnnnnnnnnnnnnnnnnnnnnnnnnnnnnnnnnnnnnnnnnnnnnnnnnnnnnnnnnnnnnnnnnnnnnnnnnnnnnnnnnnnnnnnnnnnnnnnnnnnnnnnnnnnnnnnnnnnnnnnnnnnnnnnnnnnnnnnnnnnnnnnnnnnnnnnnnnnnnnnnnnnnnnnnnnnnnnnnnnnnnnnnnnnnnnnnnnnnnnnnnnnnnnnnnnnnnnnnnnnnnnnnnnnnnnnnnnnnnnnnnnnnnnnnnnnnnnnnnnnnnnnnnnnnnnnnnnnnnnnnnnnnnnnnnnnnnnnnnnnnnnnnnnnnnnncaaaaccggttatgaccatcgcgtgtgttaaaagactttcgccatagtcaagccgttctgctttattgaattcaggggtagtgccgagcATCGACTCGTACCCGAATATCTCcgtatccatgataccatctttgcgcgtcatCATTTGACCTACATCACAACCAAACCATACCGGCTCCCCGGCTCGGATTGCAGAAGCTGTAGCTTCTTTGAGCACTTCAATGGGngtaTTGACATAGCagatgcacggggcttcctttacggtgcctagaaatttaaccgtgtatgctctgccaaacggtttatccgcagttggcgcgtgaatcaaactcactttgtttttaagatcccntccgacgaaatcgcaaaaaaaCTTCTGcggcgtaaggnctctgactttgtgaaattttntttccttntccttgtacacaaagtcgaatttctccggaggtctcccgagagccttcacgaggatagagtagatggaagaaaggaacgcttcctttttctccctcagggtatgcnnnnnnnnnnnnnnnnnnnnnnnnnnnnnnnnnnnnnnnnnnnnnnnnnnnnnnnnnnnnnnnnnnnnnnnnnnnnnnnnnnnnnnnnnnnnnnnnnnnnnnnnnnnnnnnnnnnnnnnnnnnnnnnnnnnnnnnnnnnnnnnnnnnnnnnnnnnnnnnnnnnnnnnnnnnnnnnnnnnnnnnnnnnnnnnnnnnnnnnnnnnnnnnnnnnnnnnnnnnnnnnnnnnnnnnnnnnnnnnnnnnnnnnnnnnnnnnnnnnnnnnnnnnnnnnnnnnnnnnnnnnnnnnnnnnnnnnnnnnnnnnnnnnnnnnnnnnnnnnnnnnnnnnnnnnnnnnnnnnnnnnnnnnnnnnnnnnnnnnnnnnnnnnnnnnnnnnnnnnnnnnnnnnnnnnnnnnnnnnnnnnnnnnnnnnnnnnnnnnnnnnnnnnnnnnnnnnnnnnnnnnnnnnnnnnnnnnnnnnnnnnnnnnnnnnnnnnnnnnnnnnnnnnnnnnnnnnnnnnnnnnnnnnnnnnnnnnnnnnnngtgtataggatctgcggcgacttcgtgcgcaaacgtatcgagnnnnnnnnnnnnnnnnnnnnnnnnnnnnnnnnngtttttatactgagtgagaatttttattctgtctacaacgaagtctgtgtgaatacggtgagtgttgtcacaggaaaatgtttcccgtaagatgggcgaaaagacgagcgtgtgtaggaaggtgccgtgatgcgaataccnnnnnnnnnnnnnnnnnnnnnnnnnnnnnnnnnnnnnnnnnnnnnnnnnnnnnnnnnnnnnnnnnnnnnnnnnnnnnnnnnnnnnnnnnnnnnnnnnnnnnccactGATAATGGGGTGGTCACCCGTTTTGGCAAGTATCATCGGACGCTAGAACCGGGATTGCATTATCTTATCCCCTTTGTAGAGCGGGTGTATAAAGTGCCGGTAACCAAGGTGCAAAAGGAAGAGTTCGGGTTCAGAACGTCGAAGAGTTCCGAGCAGAGTCACTATGTGAATAATATCAGTCACGAGnnnnnnnnnnnnnnnnnnnnnnnnnnnnnnnnnnnnnnnnnnnnnnnnnnnnnnnnnnnnnnnnnnnnnnnnnnnnnnnnnnnnnnnnnnnnnnnnnnnnnnnnnnnnnnnnnnnnnnnnnnnnnnnnnnnnnnnnnnnnnnnnnnnnnnnnnnnnnnnnnnnnnnnnnnnnnnnnnnnnnnnnnnnnnnnnnnnnnnnnnnnnnnnnnnnnnnnnnnnnnnnnnnnnnnnnnnnnnnnnnnnnnnnnnnnnnnnnnnnnnnnnnnnnnnnnnnnnnnnnnnnnnnnnnnnnnnnnnnnnnnnnnnnnnnnnnnnnnnnnnnnnnnnnnnnnnnnnnnnnnnnnnnnnnnnnnnnnnnnnnnnnnnnnnnnnnnnnnnnnnnnnnnnnnnnnnnnnnnnnnnnnnnnnnnnnnnnnnnnnnnnnnnnnnnnnnnnnnnnnnnnnnnnnnnnnnnnnnnnnnnnnnnnnnnnnnnnnnnnnnnnnnnnnnnnnnnnnnnnnnnnnnnnnnnnnnnnnnnnnnnnnnnnnnnnnnnnnnnnnnnnnnnnnnnnnnnnnnnnnnnnnnnnnnnnnnnnnnnnnnnnnnnnnnnnnnnnnnnnnnnnnnnnnnnnnnnnnnnnnnnnnnnnnnnnnnnnnnnCAGTAAGGTATCGAAGAAGGTCGTGGCAGGAACACGCGAGGAGTAGGGGTATGAGGAAACGTGGACTACAGGTnCACGCACGTGTACGACCTGTTCTGAACATAGGTATAGTCGTTGGGGTGCTGTTAGGTGGTGTTGTGCTCCTGCAACCGTTTTACCTCATCCAGGAAGGACAGGTTGnnnnnnnnnnnnnnnnnnnnnnnnnnnnnnnnnnnnnnnnnnnnnnnggattgtatgtcagagctccttttctgcatcACGTGCATAAGTATACAGCCAAGCTCCTGCGCGTTGATGGAGATnnnnnnnnnnnnnnnnnnnnnnnnnnnnnnnnnnnnnnnnnnnnnnnnnnnnnnnnnnnnnnnnnnnnnnnnnnnnnnnnnnnnnnnnnGTCTTTGGGGACTTATGAGGCGGCTTACTCGCGTATTTCTGACATTATCGATTCCTCAGTTAGGGATATTATCACTGTCAATGGCCTAGACGATGTTGTGCGAAGCACGAACGCTATCAACGAGTCCAATCACTCCGAGCAGTTTGACGTCcCGGTGTCGCAGCTTGCCTTTGATAGGGGTGCGGAGAAAACTGcgcatatnnnnnnnnnnnnnnnnnnnnnnnnnnnnnnnnnnnnnnnnnnnnnnnnnnnnnnnnnnnnnnnnnnnnnnnnnnnnnnnnnnnnnnnnnnnnnnnnnnnnnnnnnnnnnnnnnnnnnnnnnnnnnnnnnnnnnnnnnnnnnnnnnnnnnnnnnnnnnnnnnnnnnnnnnnnnnnnnnnnnnnnnnnnnnnnnnnnnnnnnnnnnnnnnnnnnnnnnnnnnnnnnnnnnnnnnnnnnnnnnnnnnnnnnnnnnnnnnnnnnnnnnnnnnnnnnnnnnnnntatcaagggtgaagcagacgcccgcgccgcggccgtgtacGCGCAGTCGTACGGGAAATCTCCTGAGTTCTACGGCTTCTGGAAAAGCTTGGAGGTGTACAAGAAATCGCTCCCCGACnnnnnnnnnnnnnnnnnnnnnnnnnnnnnnnnnnnnnnnnnnnnnnnnnnnnnnnnnnnnnnnnnnnnnnnnnnnnnnnnnnnnnnnnnnnnnnnnnnnnnnnnnnnnnnnnnnnnnnnnnnnnnnnnnnnnnnnnnnnnnnnnnnnnnnnnnnnnnnnnnnnnnnnnnnnnnnnnnnnnnnnnnnnnnnnnnnnnnnnnnnnnnnnnnnnnnnnnnnnnnnnnnnnnnnnnnnnnnnnnnnnnnnnnnnnnnnnnnnnnnnnnnnnnnnnnnnnnnnnnnnnnnnnnnnnnnnnnnnnnnnnnnnnnnnnnnnnnnnnnnnnnnnnnnnnnnnnnnnnnnnnnnngggagtcttccgccgcctttgacgaacacgtgcgacgcgccgcgctcgtgaatcaaacgtgcaccctccttcatttcgtccactgtccgtggtacgctgataccggcaatctgggcggtttcaaaaagattgggagtaacaactgtcgnnnnnnnnnnnnnnnnnnnnnnnnnnnnnnnnnnnnnnnnnnnnnnnnnnnnnnnnnnnnnnnnnnnnnnnnnnnnnnnnnnnnnnnnnnnnnatgcagcaactgcaaaacgttcgagatactccgcgacacattcgattgcatggacagaggcaagcataccgcttttcaccgcgctgacccccacgcctgcaaatgcggtttcaagctgatcgcgCACACAGTCTTCCTCAAGTGAATGTACACGATGCGACCAGTTCCGGGATGGGTCCATAGTAACGATGGCGGTGAGCGTAGCCACACCGAACGCTCCATACTCTTGGAAAGTTTTCAGATCCGCCTCGATGCCCGCACCACCTGAAGCATCCGATCCACCAATGCTAAGCAACTTGACCATAACGTGCCTCCTCTTAAGCGGGGTCCACTCTATTCACCATGGGTGTGGTTTGTCAACCAGAGGTTCTAAGAGAGCAAAATCAAAGGGGAAGGCACAGCATGTACTACAGATTTTTTAAATCCTATGGTAATAACCGATACTGCGAAGCACATGAAAGAATTTAAATTACACGCTTCTTTCCAACCTGCAGGCGatcagattgccgcaatcgacgcactcgtgcgtggcttgcatgnnnnnnnnnnnnnnnnnnnnnnnnnnnnnnnnnnnnnnnnnnnnnnnnnnnnnnnnnnnnnnnnnnnnnnnnnnnnnnnnnnnnnnnnnnnnnnnnnnnnnnnnnnnnnnnnnnnnnnnnnnnnnnnnnnnnnnnnnnnnnnnnnnnnnnnnnnnnnnnnnnnnnnnnnnnnnnATTTTGTGTCGTACTACGATTATTATCAGCCTGAATCGTATGTCCCTGCTCGTGATCTCTACATTGAGAAAGATGCTTCTATCAATGCAGAGATAAATAGAATGCGTTTATCtgcaacgtttagtctcatggaacgtcgtgatgttATTGTagtcgcaactgtttcgtgtatttacggtcttgggttgcctgaatcctggcgtgatctgcgtatccacgtggAAGTCAACCAGTGTCTTGATCTTGAAGATCTCAAGCGGCAACTTGTATCTCnnnnnnnnnnnnnnnnnnnnGCGGTGCTCGAGTGCGGTCGCTTCCGAGTTCGGGGAGACGTTATTGAAATTTTTCCTGCGTATTTAGAAGAATTTTACCGTATCGAGTGCGATTGGGATCGAGTTGTTCGTATCCGTCGCATTCATCCTGTAAGCGGCGCTGTTCTAAGGGAATTTGAAGaacttaccgtttaccctgcaaagcATTTTGTACTTAAAGAGGATGCAATCCCTCGCGCCATGGACCGTATTAGACAAGAACTCCATGAacggttggtacaattaactcaagagaataagnnnnnnnnnnnnnnnnnnnnnnnnnnnnnnnnnnnnnnnnnnnnnnnnnnnnnnnnnnnnnnnnnnnnnnnnnnnnnnnnnnnnnnnnnnnnnnnnnnnnnnnnnnnnnnnnnnnnnnnnnnnnnnnnnnnnnnnnnnnnnnnnnnnnnnnnnnnnnnnnnnnnnnnnnnnnnnnnnnnnnnnnncgtAACACTTCCGCAGCTTGGTGCAATGTATGAAGGAGATCGGGTGCGTAAACAGAATCTCATCGATTTTGGCTTTCGCTTGCCGTGTGCACGAGATAACCGTCCACTGAAAGATTCGGAATTTGAGGCnnnnnnnnnnnnnnnnnnnnnnnnnnnnnnnnnnnnnnnnnnnnnnnnnnnnnnnnnnnnnnnnnnnnnnnnnnnnnnnnnnnnnnnnnnnnnnnnnnnnnnnnnnnnnnnnnnnnnnnnnnnnnnnnnnnnnnnnnnnnnnnnnnnnnnnnnnnnnnnnnnnnnnnnnnnnnnnnnnnnnnnnnnnnnnnnnnnnnnnnnnnnnnnnnnnnnnnnnnnnnnnnnnnnnnnnnnnnnnnnnnnnnnnnnnnnnnnnnnnnnnnnnnnnnnnnnnnnnnnnnnnnnnnnnnnnnnnnnnnnnnnnnnnnnnnnnnnnnnnnnnnnnnnnnnnnnnnnnnnnnnnnnnnnnnnnnnnnnnnnnnnnnnnnnnnnnnnnnnnnnnnnnnnnnnnnnnnnnnnnnnnnnnnnnnnnnnnnnnnnnnnnnnnnnnnnnnnnnnnnnnnnnnnnnnnnnnnnnnnnnnnnnnnnnnnnnnnnnnnnnnnnnnnnnnnnnnnnnnnnnnnnnnnnnnnnnnnnnnnnnnnnnnnnnnnnnnnnnnnnnnnnnnnnnnnnnnnnnnnnnnnnnnnnnnnnnnnnnnnnnnnnnnnnnnnnnnnnnnnnnnnnnnnnnnnnnnnnnnnnnnnnnnnnnnnnnnnnnnnnnnnnnnnnnnnnnnnnnnnnnnnnnnnnnnnnnnnnnnnnnnnnnnnnnnnnnnnnnnnnnnnnnnnnnnnnnnnnnnnnnnnnnnnnnnnnnnnnnnnnnnnnnnnnnnnnnnnnnnnnnnnnnnnnnnnnnnnnnnnnnnnnnnnnnnnnnnnnnnnnnnnnnnnnnnnnnnnnnnnnnnnnnnnnnnnnnnnnnnnnnnnnnnnnnnnnnnnnnnnnnnnnnnnnnnnnnnnnnnnnnnnnnnnnnnnnnnnnnnnnnnnnnnnnnnnnnnnnnnnnnnnnnnnnnnnnnnnnnnnnnnnnnnnnnnnnnnnnnnnnnnnnnnnnnnnnnnnnnnnnnnnnnnnnnnnnnnnnnnnnnnnnnnnnnnnnnnnnnnnnnnnnnnnnnnnnnnnnnnnnnnnnnnnnnnnnnnnnnnnnnnnnnnnnnnnnnnnnnnnnnnnnnnnnnnnnnnnnnnnnnnnnnnnnnnnnnnnnnnnnnnnnnnnnnnnnnnnnnnnnnnnnnnnnnnnnnnnnnnnnnnnnnnnnnnnnnnnnnnnnnnnnnnnnnnnnnnnnnnnnnnnnnnnnnnnnnnnnnnnnnnnnnnnnnnnnnnnnnnnnnnnnnnnnnnnnnnnnnnnnnnnnnnnnnnnnnnnnnnnnnnnnnnnnnnnnnnnnnnnnnnnnnnnnnnnnnnnnnnnnnnnnnnnnnnnnnnnnnnnnnnnnnnnnnnnnnnnnnnnnnnnnnnnnnnnnnnnnnnnnnnnnnnnnnnnnnnnnnnnnnnnnnnnnnnnnnnnnnnnnnnnnnnnnnnnnnnnnnnnnnnnnnnnnnnnnnnnnnnnnnnnnnnnnnnnnnnnnnnnnnnnnnnnnnnnnnnnnnnnnnnnnnnnnnnnnnnnnnnnnnnnnnnnnnnnnnnnnnnnnnnnnnnnnnnnnnnnnnnnnnnnnnnnnnnnnnnnnnnnnnnnnnnnnnnnnnnnnnnnnnnnnnnnnnnnnnnnnnnnnnnnnnnnnnnnnnnnnnnnnnnnnnnnnnnnnnnnnnnnnnnnnnnnnnnnnnnnnnnnnnnnnnnnnnnnnnnnnnnnnnnnnnnnnnnnnnnnnnnnnnnnnnnnnnnnnnnnnnnnnnnnnnnnnnnnnnnnnnnnnnnnnnnnnnnnnnnnnnnnnnnnnnnnnnnnnnnnnnnnnnnnnnnnnnnnnnnnnnnnnnnnnnnnnnnnnnnnnnnnnnnnnnnnnnnnnnnnnnnnnnnnnnnnnnnnnnnnnnnnnnnnnnnnnnnnnnnnnnnnnnnnnnnnnnnnnnnnnnnnnnnnnnnnnnnnnnnnnnnnnnnnnnnnnnnnnnnnnnnnnnnnnnnnnnnnnnnnnnnnnnnnnnnnnnnnnnnnnnnnnnnnnnnnnnnnnnnnnnnnnnnnnnnnnnnnnnnnnnnnnnnnnnnnnnnnnnnnnnnnnnnnnnnnnnnnnnnnnnnnnnnnnnnnnnnnnnnnnnnnnnnnnnnnnnnnnnnnnnnnnnnnnnnnnnnnnnnnnnnnnnnnnnnnnnnnnnnnnnnnnnnnnnnnnnnnnnnnnnnnnnnnnnnnnnnnnnnnnnnnnnnnnnnnnnnnnnnnnnnnnnnnnnnnnnnnnnnnnnnnnnnnnnnnnnnnnnnnnnnnnnnnnnnnnnnnnnnnnnnnnnnnnnnnnnnnnnnnnnnnnnnnnnnnnnnnnnnnnnnnnnnnnnnnnnnnnnnnnnnnnnnnnnnnnnnnnnnnnnnnnnnnnnnnnnnnnnnnnnnnnnnnnnnnnnnnnnnnnnnnnnnnnnnnnnnnnnnnnnnnnnnnnnnnnnnnnnnnnnnnnnnnnnnnnnnnnnnnnnnnnnnnnnnnnnnnnnnnnnnnnnnnnnnnnnnnnnnnnnnnnnnnnnnnnnnnnnnnnnnnnnnnnnnnnnnnnnnnnnnnnnnnnnnnnnnnnnnnnnnnnnnnnnnnnnnnnnnnnnnnnnnnnnnnnnnnnnnnnnnnnnnnnnnnnnnnnnnnnnnnnnnnnnnnnnnnnnnnnnnnnnnnnnnnnnnnnnnnnnnnnnnnnnnnnnnnnnnnnnnnnnnnnnnnnnnnnnnnnnnnnnnnnnnnnnnnnnnnnnnnnnnnnnnnnnnnnnnnnnnnnnnnnnnnnnnnnnnnnnnnnnnnnnnnncctacacactcgccttctcttgggggacagacagaacttccgaagagagaacaataggttccggcgatgtttcgaatagggtaaggcgttctgacgcctcctctaatgactgcgccagacgCGCTACnnnnnnnnnnnnnnnnnnnnnnnnnnnnnnnnnnnnnnnnnnnnnnnnnnnnnnnnnnnnnnnnnnnnnnnnnnnnnnnnnnnnnnnnnnnnnnnnnnnnnnnnnnnnnnnnnnnnnnnnnnnnnnnnnnnnnnnnnnnnnnnnnnnnnnnnnnnnnnnnnnnnnnnnnnnnnnnnnnnnnnnnnnnnnnnnnnnnnnnnnnnacagcgtgctgagttcatcgggcaccgtcggtcttgtcttctgagataaaggaggctgagcaaatccttcctcggtaaaGAAACAACTTGCGTATGCAGCACCGATGCGCGCGCACAGCGCATTGATATCCTCATCCAGTTCACTGACCTGCGCTGTGAACGCCGCAACGCGCTTCAGCnGCTTGGCACCACAGTCAAGGGCGCGCAGGCGCGCGTCGAGTGCTTCTTGCTCATCCATAAGCTCCTGGCTATGTTGAAGGTTGCTCGCGTAAACnnnnnnnnnnnnnnnnnnnnnnnnnnnnnnnnnnnnnnnnnnnnnnnnnnnnnnnnnnnnnnnnnnnnnnnnnnnnnnnnnnnnnnnnnnnnnnnnnnnnnnnnnnnnnnnnnnnnnnnnnnnnnnnnnnnnnnnnnnnnnnnnnnnnnnnnnnnnnnnnnnnnnnnnnnnnnnnnnnnnnnnnnnnnnnnnnnnnnnnnnnnnnnnnnnnnnnnnnnnnnnnnnnagctggtcaCGCTTGGCGCGTAAATCCTGCAGAGTAGACTCAAGCTCCGCCGTGCGCGCATAGATCGGTTCGAGCGCAGGTAGGCCTACGTGCGCGTAGGTGGCATAGTACTGGGCTACAAAACTCCTGAGTACATCCCGCTCCTGACGAGCATGGCGGTgcaatactttgctcacccgctttccaagagcggcaaGTTCTTCTTGTCTTTGAAGTATCGACTTAATATCAAGGATAGACTCAGCAACCTGATCGCGCTGACGTTGAAGCGCGTGGCATCGGCTGATGTCAGTGTCCTGTACGCCAAGTCCGCTGATGTCACACGCAGCACCGCCGCGCACAATATGttcaCCGAGACTGCAACAATGGCTCTGCAGATCCTGCTGCGCACGCTGACACGCGGCATTCAGCGCGGAAAGACTCTTATCCGCGAACATGACCGCATTGTAAACACTTCCCCTGCGTATGTACAGGGCACCTCACTCCCTCTTTACCCATGCAAGGACAGACGTCCTGCCTAACTCtggcggggtactacctgcgcgctcgtattcgggtacagattatacgcccgATCCATTGCGTTAGCGCAACCTGCGCCAGGATTGCAAnnnnnnnnnnnnnnnnnnnnnnnnnnnnnnnnnnnnnnnnnnnnnnnnnnAnnGCTACGTCTCCTAAAcCTCCTCCCCCAATCGCCCCTGCCATTGCTGAGTATCCAATTAGATTAATTACCATCAGTGCTACACCAGAAACACATGCAGGAGCAGCCTCAGGCAGCATGATTTTTAGTACAAGTTGCCGCATGCTTGAACCCATtgccaccgccgcttggattatccctggctccacctccagcagagcacTTTCAATCACCCGTGCCACgaangGGAGCGCAGCTATCGCAAGCGGGAGGATAGCCGCCGCCGTTCCCACTGTGCGTCCTGTGAGCATACGCGAGAGCGGAAGCAACACCACCAGCAAAATCACAAATGGGAaagcgcgcagcacgttCATtgacgaacttaacacacgatgccacgcaGGATGCGCCCACACGTGTCCAGGCGACATTACGCACAGCAATATCCCCAACGGGGTTCCCGCTGCTAGCGCAATACTTGCTGAAGCACACACCATCAGACTTGTCTGTCCGGTTGCACGAGCTACTAAAAGCCACAGTGTCTGGTTCGCCATTACCAGTGTGCCTTGTCAATAGCATGCACGGCACACGCACCCGAAGACATTCGATGGAAGGGTTCTGACTTTGCAAAAGAGGCAAAAGAACAGCCCGACAACAACTGCTGTGTGATCAGCCGCCGTGGCGCAGCAAAAAGTTGTGTCACCCTTCCTTCTTCCACTATTTCTCCCTCATGCAATACGGCGGCCCGATCGCACAAGTCGCGTACCACCTCCATCTGGTGTGTAATCAATACGACCGTCAGACGCAGTGAGCACTGCACATTTTTCAGCAACGTCAGAATTGACTGTGTTGTTTGagggtcgagagcgcttgttgcttCGTCGCAGAAGAGTATTGCAGGTTCTGCAGCCAAGGCGCGCGcAATGGCTACGCGCTGCTTCTGCCCACCTGACAGCGTGCTGATACGCGCCTGTGCTTTGTCTGCAAGACCAACCAAGTGTAGCAAATGCAACACGCGCGCGTAGGCGTCCTTACGTGnnnnnnnnnnnnnnnnnnnnnnnnnnnnnnnnnnnnnnnnnnnnnnnnnnnnnnnnaacagatgaaattgttgaaaGATCAATCCTATGCGCCTGCGCTGTGCACGCAAGGCAGCACCGCGCAGTAAATCTACCCTCGTGGTGTGATAAAAAACGGCTCCTTCGTCAGGTTTCTCCAAAAGACTCATGATGCGCAGTAGCGTTGATTTTCCTGCACCACTTTTGCCGATGATTCCATACATTCCGTGCGCTGGGATCTCTAGGGAAATATCTCGAAGTATAGGAACACCTGCGTATGTCTTCTTTAGCCGCTCGAGTCTGACCATGGGGACCAGGGTCTCTGCAAATAGGTACGTGTGTCAACACTACGGTATGCGATCCTGCAGCACGGGTGCGGTGCACGGCGTGACTTGTACACGCATGCATTTTTACCGTAGGATGCGCACTCGTGGTCCGTGTCTATGGCTGAGTGTACCCGGGAACAGAGAAAGAGACGAGGTGCAGGGCGTnnnnnnnnnnnnnnnnnnnnnTTGAGTCCTGCCTCTTGCGCGGCAGATGCGCTGACGGAGCATATTTCTCCAGCGTATGCGCATTTAATTGCACAAGCGCAGGGCGCGGACGCGCAGGCGCTGAAACGTCAGGTGTGCTTTGCGCCACAGGAGCGTGTGGTGCATGCTTGCGAGTGTGCCGACCCATTGGGTGAGGACCGGTACTGCGTGACACCCTTTTTGGTGCATCAGTATGCGAATCGTGTGTTGATGTTGGCAACAGGACGTTGCTTTTCACACTGTCGCTATTGTTTnnnnnnnnnnnnnnnnnnnnnacgtgcagggtggatccccaacgaagagcgcgagaagattattacgtatcttcgtgctaccccttcggtgaagGAAATCCtggtttcaggtggngatccactcactggttcttttgcacaggtcacatcgcttttccgcgcactgcgcagtnnnnnnnnnnnnnnnnnnnnnnnnnnnnnnnnnnnnnnnnnnnnnnnnnnnnnnnnnnnnnnnnctcccgagcnnnnnnnnnnnnnnnnnnnnnnnnnnnnnnnnnnnnnnnnnnnnnnnnnnnnnnnnnnnnnnnnnnnnnnnnnnnnnnnnnnnnnnnnnnnnnnnnnnnnnnnnnnnnnnnnnnnccctgtgcaatcgcagtcgGTACTGTTgcgCGGGGTGAACGATTCGGTAGAGACGCTGTGCACACTGTTTCACGCGCTCACTTGTCTGGGGGTTAAGCCGGGGTATCTATTTCAGTTGGATTTGGCGCCTGGAACTGGGGATTTTCGTGTGCCACTTTCTGACACGCTAGCTCTGTGGtgcnnnnnnnnnnnnnnnnnnnnnnnnnnnnnnnnnnnnnnnnnnnnnnnnnnnnnnnnnnnnnnnnnnnnnnnnnnnnnnnnnnnnnnnnnnnnnnnnnnnnnnnnnnnnnnnnnnnnnnnnnnnnnnnnnnnnnnnnnnnnnnnnnnnnnnnnnnnnnnnnnnnnnnnnnnnnnnnnnnnnncgtcaaaacaccccgcgcctcatcgagtggagagtgcgccgcgtggtacacgggnnnnnnnnnnnnnnnnnnnnnnnnnnnnnnnnnnnnnnnnnnnnnnnnnnnnnnnnnnnnnnnnnnnnnnnnnnnnnnnnnnnnnnnnnnnnnnnnnnnnnnnnnnnnnnnnnnnnnnnnnnnnnnnnnnnnnnnnnnnnnnnnnnnnnnnnnnnnnnnnnnnnnnnnnnnnnnnnnnnnnnnnnnnnnnnnnnnnnnnnnCCGTTGATGCGAGCTTCCAAAACCTCGAGTGCATCGCGAAGCTGCTGTGGCAGCCGCGCGCCAAACTTTGGGTAGTGATTCTCTCGGATGTCTTTAATTTCCTTTTTCCATCCGGCTATGTCCACCGACAAAAGCTCTTTCACTGCCTGCGTGCTTACGTTTAACCCCTCTGTGTTCAAGGCTCCCTCTTTGggcatccaaccgatcgctgtttccaccgcgttgtccacaccatcacagcggtcaaagatccacgcgagtactcggctgttatcgccatatccgggnnnnnnnnnnnnnnnnnnnnnnnnnnnnnnnnnnnnnnnnnnnnnnnnnnnnnnnnnnnnnnnnnnnnnnnnnnnnnnnnnnnnnnnnnnnnnnnnnnnnnnnnnnnngtctgccatatggtagccgcagaaggggagcatcgcgaacgggtctcggcgaaTCTGACCTACCTGGTCAGAGATAACTGCTGCAGTTACCTCCGAGCCGATGATGGAACCTAGAAACACCCCGTGATTCCAGTCCCGAGCCTGATGCACCAGGGGAACCGTACTGGGGCGACGGCCGCCAAACAGAAAAGCGTCGATAGGGACcccttcgggatcnnnnnnnnnnnnnnnnnnnnnnnnnnnnnnnnnnnnnnnnnnnnnnnnnnnnnnnnnnnnnnnnnnnnnnnnnnnnnnnnnnnnnnnnnnnnnnnnnnnnnnnnnnnnnnnnnnnnnnnnnnnnnnnnnnnnnnnnnnnnnnnnnnnnnnnnnnnnnnnnnnnnnnnnnnnnnnnnnnnnnnnnnnnnnnnnnnnnnnnnnnnnnnnnnnnnnnnnnnnnnnnnnnnnnnnnnnnnnnnnnnnnnnnnnnnnnnnnnnnnnnnnnnnnnnnnnnnnnnnnnnnnnnnnnnnnnnnnnnnnnnnnnnnnnnnnnnnnnnnnnnnnnnnnnnnnnnnnnnnnnnnnnnnnnnnnnnnnnnnnnnnnnnnnnnnnnggaagggnnnnnnnnnnnnTAGCCAGATTCGTTTTGCCACATGCAGAGGGAAACGCCGCACCAATGTacttggtctttccagcagggttggtgattttaaggatgagcatgtgctctgcaagccacccttcgtctcgtgcgagtactgaagnnnnnnnnnnnnnnnnnnnnnnnnnnnnnnnnnnnnnnnnnnnnnnnnnnnnnnnnnnnnnnnnnnnnnnnnnnnnnnnntcaggaaagtgaGAGATGTATTTGCGCTCCATATCCGCGCAGGGCCACTGGCCTGCGTCAGTTACGCCCGGTCCTAACGGCTTCCCCACAGAGTGCAAACAGGGGACGAACTCACCATCAGTACCCAACGCCTCAAGCACGCGGGTACCCACGCGTGTCATGATGnnnnnnnnnnnnnngacGTACTCAGAATCGGTGATTTCGATGCCATTTTTAGAGATGGGTGAGCCGACCGGTCCCATGGAAAAGGGAATGACGTACATGGTACGGCCCTTCATGCACTGGGAATAGAGACCGGTCATAGTCTTTTTTAATTCTGCAGGnTCGGTCCAAtgGTTAGTGGGTCCTGCATCATCCTCCCTTTTTGAGGCGATGAAGGTGCGCGCTTCGACGCGCGCAACGTCGGAGGGCTGTGAGCGAAAGAGGAAGCAGTTCTTACGTTTTTTTAATGGGGTAGCCAAGCCAGCATCCACCATTTTCTGCATAATGGTGTCGTACTCTTTTTTCGAACCGTCGCAGACGTAGACGGTATCTGGGGCACAGAGTGCGACCATCTCTTCTATCCACGCCTTTGCTCGAGCGTGGGCAATCTCGTGAAGttccataacgccgctCCTTGGTGCGTAGCGTGCTGCACGGGTATTCCAGGCACGTATCGCCCCAGAAGTATAGCGCGCTAAAGGTAAATAAAAAAGACCTCTTCAAACCGAGTGTCTCTGTCaccgcagggagccgacatgagcggtgttctttACCTAGGACGTTTCACTCTCTGTCTATTACCTGTCAGTTGTTTTTCTCAAAAAGTGATGACGTGTGCCGATACCGTCAGGGGTGCGCAAGAGGTTTTTATGCTATGTATCTACGTTGAGCTTCCCTATTACTATCAACTGACGCGCATCTTCCCTGCTGACATCGAATCGCTATGTGCGCGTATGagaaggttcgctgtccacaacggtgCTGCCCTCCACGAGGCATCGTCCGTTCGTATCTTTGCATTTGAAGCACACAGTCTCGGTTCTGTATACGCCGCGGTACGCTGCGTGCGTGCGCTGTATCAAACACTGGACACATACGAAAAGCAGGTGAAGGAATTTCGTATCCTCATGGAcgttgttnnnnnnnnnnnnnnnnnnnnnnnnnnnnnnnnnnnnnnnnnnnnnnnnnnnnnnnnnnnnnnnnnnnnnnnnnnnnnnnnnnnnnnnnnnnnnnnnnnnnnnnttctcaagcattaccttgaatttttgccactgccagcgctgaatatgtaccaggttaatggtttcctttcactttgtgcggaaaaaccttttccacaaggggtaaccacgcactgcatagttgtgcgtaccacttcttcatacatgagtgcnnnnnnnnnnnnnnnnnnnnnnnnnnnnnnnnnnnnnnnnnnnnnnnnnnnnnnnnnnnnnnnnnnnnnnnnnnnnnnnnnnnnnnnnnnnnnnnnnnnnnnnnnnnnnnnnnnnnnnnnnnnnnnnnnnnnnnnnnnnnnnnnnnnnnnnnnnnnnnnnnnnnnnnnnnnnnnnnnnnnnnnnnnnnnnnnnnnnnnnnnnnnnnnnnnnnnnnnnnnnnnnnnnnnnnnnnnnnnnnnnnnnnnnnnnnnnnnnnnnnnnnnnnnnnnnnnnnnnnnnnnnnnnnnnnnnnnnnnnnnnnnnnnnnnnnnnnnnnnnnnnnnnnnnnnnnnnnnnnnnnnnnnnnnnnnnnnnnnnnnnnnnnnnnnnnnnnnnnnnnnnnnnnnnnnnnnnnnnnnnnnnnnnnnnnnnnnnnnnnnnnnnnnnnnnnnnnnnnnnnnnnnnnnnnnnnnnnnnnnnnnnnnnnnnnnnnnnnnnnnnnnnnnnnnnnnnnnnnnnnnnnnnnnnnnnnnnnnnnnnnnnnnnnnnnnnnnnnnnnnnnnnnnnnnnnnnnnnnnnnnnnnnnnnnnnnnnnnnnnnnnnnnnnnnnnnnnnnnnnnnnnnnnnnngAAATATCAGGAAGGATCGCTTTGTGCTAGTACGGATCTGCTCAGAACTTTCCAGGAGCTTCAGTACAAATACACATCCGATTGTGTGTTACACAGCTTGTTTCATACGTATTCTGACGTGCAGATTGCGCACCTACAGGTAGAAGAGTACnnnnnnnnnnnnnnnnnnnnnnnnnnnnnnnnnnnccaacacacgctgctggtgggcatgcgtgaagacgcagaggccgcgttcagagaagcaaaggcttgtctgacaacactgcaggcgcggcgttttgtgtccgctgaataccggaccttttccctcttaggatttctaaccnnnnnnnnnnnnnnnnnnnnnnnnnnnnnnnnnnnnnnnnnnnnnnnnnnnnnnnnnnnnnnnnnnnnnnnnnnnnnnnnnnnnnnnnnnnnnnnnnnnnnnnnnnnnnnnnnnnnnnnnnnnnnnnnnnnnnnnnnnacccaggcgcggctttttctgagtaagctatccgatgcgatatccacgtattttgagcagcgatgGAAAACTGTCAGTCTGTTTATGCAGGGCAGAATTTCTCTCAGCCTCGGGGAGTATGCACnnnnnnnnnnnnnnnnnnnnnnnnnnnnnnnnnnnnnnnnnnnnnnnnnnnnnnnnnnnnnnnnnnnnnnnnnnnnnnnnnnnnnnnnnnnnnnnnnnnnnnnnnnnnnnnnnnatgcagcgcacgcgctgtttcaggacatgtgtgatcaataccctgATGCATATCTCTTTCTTGTAGAAAGCTATGTCCGCGCAGAATGTTTTGACGATCCCACGTTGTTTCAATCGTTTCCTGAGGAAACGACCTCTCGCGAGCCATGTGTGCCGTCCTTCTCTCTTGATACGCCGATTTACTCAGGGTTCTCCTGCGCAGAAGATCTGGTATGGGGCAGGCAGnnnnnnnnnnnnnnnnnnnnnnnnnnnnnnnnnnnnnnnnnnnnnnnnnnnnnnnnnnnnnnnnnnnnnnnnnnnnnnnnnnnnnnnnnnnnnttCCACCACCATAAGCAAAAACTTGAGGCCATTGCACGTCGCGCGTTTCAAATAGGTGATCCGAGTGCTGCGTTGTTTCTGTACCTCTGCTATGATGTGTCCTACCGCGTGCACGGCGCAGAGGCTGCTGTCACGACAGCGCACCTGAGTAGGGCGtttaaagtgatgcagcgcagcgttgcgtatatgtcagaaaataccgttcgcgcacagttcatgcaggataacttttggaatgcaaaannnnnnnnnnnnnnnnnnnnnnnnnnnnnnnnnnnnnnnnnnnnnnnnnnnnnnngtcaattgtggcattatcgntctnccgaatgtggggaagtcgacaattttctccgcgctcactgcaaacgtcgtggaggcggcgaattatcccttttgtactatcgAACCTAACGTGGGTATGGTGACAGTACCTGATGTGCGTCTTGAAGCACTGGCTGGTCATTTTCGGCCAAAGAAAACGGnnnnnnnnnnnnnnnnnnnnnnnnnnnnnnnnnnnnnnnnnnnnnnnnnntcgcagggggagggattgggcaatcgttttcttgcgcatgtgcgagaggttggagtactnnnnnnnnnnnnnnnnnnnnnnnnnnnnnnnnnnnnnnnnnnnnnnnnnnnnnnnnnnnnnnnnnnnnnnnnnnnnnnnnnngtgcatatagnnnnnnnnnnnnnnnnnnnnnnnnnnnnnnnnnnnnnnnnnnnnnnnnnnnnnnnnnnnnnnnnnnnnnnnnnnnnnnnnnnnnnnnnnnnnnnnnnnnnnnnnnnnnnnnnnnnnnnnnnnnnnnnnnnnnnnnnnnnnnnnnnnnnnnnnnnnnnnnnnnnnnnnnnnnnnnnnnnnnnnnnnnnnnnnnnnnnnnnnnnnnnnnnnnnnnnnnnnnnnnnnnnnnnnnnnnnnnnnnnnnnnnnnnnnnnnnnnnnnnnnnnnnnnnnnnnnnnnnnnnnnnnnnnnnnnnnnnnnnnnnnnnnnnnnnnnnnnnnnnnnnnnnnnnnnnnnnnntttgaagcagagcttgcgcagctttctgatgtggcagagcaaaacgcctttttgcaagaattannnnnnnnnnnnnnnnnnnnnnnnnnnnnnnnnnnnnnnnnnnnnnnnnnnnnnnnnnnnnnnnncttttttaccgcggggcctgaggnnnnnnnnnnnnnnnnnnnnnnnnnnnnnnnnnnnnnnnnnnnnnnnnnnnnnnnnnnnnnnnnnnnnnnnnnnnnnnnnnnnnnnnnnnnnnnnnnnnnnnnnnnnnnnnnnnnnnnnTCCTGTGGGagtgtggcaaaggtgagggaggcaaaccgcgttcggcaggaggggaaggaatacgaggtgcaagacggggacgttatcttttttaaattcaatgtgtgaaacacaggcgctccgttccgtctgtgcgccgtgtgcgatacagnnnnnnnnnnnnnnnnnnnnnnnnnnnnnnnnnnnnnnnnnnnnnnnnnnnnnnnnnnnnnnnnnnnnnnnnnnnnnnnnnnnnnnnnnnnnnnnnnnnnnnnnnnnnnnnnnnnnnnnnnnnnnnnnnnnnnnnnnnnnnnnnnnnnnnnnnnnnnnnnnnnnnnnnnnnnnnnnnnnnnnnnnnnnnnnnnnnnnnnnnnnnnnnnnnnnnnnnnnnnnnnnnnnnnnnnnnnnnnnnnnnnnnnnnnnncctggatacagtggcacggcgctgttcagtaagcgcgcgccagatgcggtgcgtttcttcggggttccggcttttgacnnnnnnnnnnnnnnnnnnnnnnnnnnnnnnnnnnnnnnnnnnnnnnnnnnnnnnnnnnnnnnnnnnnnnnnnnnnnnnnnnnnnnnnnnnncgtataagcttgatttttgcgcagcgtttcgtgcgttctgtgatgaagagcgtacggcnnnnnnnnnnnnnnnnnnnnnnnnnnnnnnnnnnnnnnnnnnnnnnnnnnnnnnnnnnnnnnnnnnnnnnnnnnnnnnnnnnnnnnnnnnnnnnnnnnnnnnnnnnnnnnnnnnnnnnnnnnnnnnnnnnnnnnnnnnnnnnnnnnnnnnnnnnnnnnnnnnnnnnnnnnnnnnnnnnnnnnnnnngtggtggagctaccgtgCCCGTGCACGCGCGCGTAACATTGGATGGCGCATCGATTACCAGTGTGTGGACCAAGCCTTTTTAGCGCGCGTGACCTCTTCGCAGATACTGTCCGAGGTGACAGGATCGGATCACTGCCCAGTGTGTTTGACGTACGCGGACTAATCCGTTTCCGGGGTGAGCGGCACGTCCGCGCAAACTAAGACGTACCCGCGCGCACAGGCAGCGTCAGAGGTGGTAGCGAACGTCCACACCCGCGGCTATGAACTGTGCGGTGCGCGTGTTGGTCTGCTGTCTATCTTCTTCAATAATCTTTTCGCATGACCGGGGTACGCCGCTGTACGTGGCGCTTACCCCCAAGGACCAGTGCTCTGTCAGTTGAAAATAGCACCCCGCTGCCGCCTTGAGCACAAGACCGTAGTAGGtagacgtgtagtaatgctgataATTGAAGCCAGCCCCTACCGTCAGTGGCAAGCGGATGCGCCAGAAGGCAACCGTGTACCCGGCAGTGAGGGCAACGGGAATTGCAAGGTAATAGTACggagtagtgggactgtacgtattgtttggatagctgcaatggtactgcacacttgcgtcaatcccgagcgacagnnnnnnnnnnnnnnnnnnnnnnnnnnnnnnnnnnnnnnnnnnnnnnnnnnnnnnnnnnnnnnnnnnnnnnnnnnnnnnnnnnnnnnnnnnnnnnnnnnnnnnnnnnnnnnnnnnnnnnnnnnnnnnnnnnnnnnnnnnnnnnnnnnnnnnnnnnnnnnnnnnnnnnnnnnnnnnnnnnnnnnnnnnnnnnnnnnnnnnnnnnnnnnnnnnnnnnnnnnnnnnnnnnnnnnnnnnnnnnnnnnnnnnnnnnnnnnnnnnnnnnnnnnnnnnnnnnnnnnnnnnnnnnnnnnnnnnnnnnnnnnnnnttacgcgtctcgtcgaggacctactccattctanncccccccacggctgtttgtcgaacccTTTTTAAAGGGTTCGTTCTCGCGCGCTGGGCAGCACGCGCGTGAGGCGCCTATGCCATCGGGAGCTGCGTTTTTCTTATGCCCCACGAGGGGactgcggggtatgtcgtgcgtccgcatgggtgtggtatcggtgagaaagacaccctgaannnnnnnnnnnnnnnnnnnnnnnnncaagacacccccatacaccgggaagtnnnnnncgactccagccccggctgcggctgcggctcgggcatagtcgccagaaagcgtggaattgtagctacaaccgggaagtagatGGCTGGGACTGGGGCTCCGGGCAGGGCTCGGGCTTGGGTGAAGGnnnnnnnnnnnnnnnnnatgtacgccnnnnnnnnnnnnnnnnnnnnnnnnnnnnnnnnnnnnnnnnnnnnnnnnnnnnnnnnnnnnnnnnnnnnnnnnnnnnnnnnnnnnnnnnnnnnnnnnnnnnnnnnnnnnnnnnnnnnnnnnnnnnnnnnnnnnnnnnnntctannnnnnnnnnnnnnnnnnnnnnnnnnnnnnnnnnnnnnnnnnnnnnnnnnnnnnnnnnnnnnnnnnnnnnnnnnnnnnnnnnnnnnnnnnnnnnnnnnnnnnnnnnnnnnnnnnnnnnnnnnnnnnnnnnnnnnnnnnnnnnnnnnnnnnnnnnnnnnnnnnnnnnnnnnnnnnnnnnnnnnnnnnnnnnnnnnnnnnnnnnnnnnnnnnnnnnnnnnnnnnnnnnnnnnnnnnnnnnnnnnnnnnnnnnnnnnnnnnnnnnnnnnnnnnnnnnnnnnnnnnnnnnnnnnnnnnnnnnnnnnnnnnnnnnnnnnnnnnnnnnnnnnnnnnnnnnnnnnnnnnnnnnnnnnnnnnnnnnnnnnnnnnnnnnnnnnnnnnnnnnnnnnnnnnnnnnnnnnnnnnnnnnnnnnnnnnnnnnnnnnnnnnnnnnnnnnnnnnnnnnnnnnnnnnnnnnnnnnnnnnnnnnnnnnnnnnnnnnnnnnnnnnnnnnnnnnnnnnnnnnnnnnnnnnnnnnnnnnnnnnnnnnnnnnnnnnnnnnnnnnnnnnnnnnnnnnnnnnnnnnnnnnnnnnnnnnnnnnnnnnnnnnnnnnnnnnnnnnnnnnnnnnnnnnnnnnnnnnnnnnnnnnnnnnnnnnnnnnnnnnnnnnnnnnnnnnnnnnnnnnnnnnnnnnnnnnnnnnnnnnnnnnnnnnnnnnnnnnnnnnnnnnnnnnnnnnnnnnnnnnnnnnnnnnnnnnnnnnnnnnnnnnnnnnnnnnnnnnnnnnnnnnnnnnnnnnnnnnnnnnnnnnnnnnnnnnnnnnnnnnnnnnnnnnnnnnnnnnnnnnnnnnnnnnnnnnnnnnnnnnnnnnnnnnnnnnnnnnnnnnnnnnnnnnnnnnnnnnnnnnnnnnnnnnnnnnnnnnnnnnnnnnnnnnnnnnnnnnnnnnnnnnnnnnnnnnnnnnnnnnnnnnnnnnnnnnnnnnnnnnnnngggacaccctgggtgaaaagactgcaccatGCTAGGATGGGGAATGGATATGTCCAAAAGTGTGATGCTGTGTTGCCTGTTGAGTGTACAACcctgttannnnnnnnnnnnnnnnnnnnnnnnnnnnnnnnnnnnnnnnnnnnnnnnnnnnnnnnnnnnnnnnnnnnnnnnnnnnnnnnnnnnnnnnnnnnnnnnnnnnnnnnnnnnnnnnnnnnnnnnnnnnnnnnnnnnnnnnnnnnnnnnnnnnnnnnnnnnnnnnnnnnnnnnnnnnnnnnnnnnnnnnnnnnnnnnnnnnnnnnnnnnnnnnnnnnnnnnnnnnnnnnnnnnnnnnnnnnnnnnnnnnnnnnnnnnnnnnnnnnnnnnnnnnnnnnnnnnnnnnnnnnnnnnnnnnnnnnnnnnnnnnnnnnnnnnnnnnnnnnnnnnnnnnnnngtacgagccctacacgaaaaatgagcaggggactactgttgcctccaacaccgtgttcccgtgcacggtcttggagnnnnnnnnnnnnnnnnnnnnnnnnnnnnnnnnnnnnnnnnnnnnnnnnnnnnnnnnnnnnnnnnnnnnnnnnnnnnnnnnnnnnnnnnnnnnnnnnnnnnnnnnnnnnnnnnnnnnnnnnnnnnnnnnnnnnnnnnnnnnnnnnnnnnnnnnnnnnnnnnnnnnnnnnnnnnnnnnnnnnnnnnnnnnnnnnnnnnnnnnnnnnnnnnnnnnnnnnnnnnnnnnnnnnnnnnnnnnnnnnnnnnnnnnnnnnnnnnnnnnnnnnnnnnnnnnnnnnnnnnnnnnnnnnnnnnnnnnnnnnnnnnnnnnnnnnnnnnnnnnnnnnnnnnnnnnnnnnnnnnnnnnnnnnnnnnnnnnnnnnnnnnnnnnnnnnnnnnnnnnnnnnnnnnnnnnnnnnnnnnnnnnnnnnnnnnnnnnnnnnnnnnnnnnnnnnnnnnnnnnnnnnnnnnnnnnnnnnnnnnnnnnnnnnnnnnnnnnnnnnnnnnnnnnnnnnnnnnnnnnnnnnnnnnnnnnnnnnnnnnnnnnnnnnnnnnnnnnnnnnnnnnnnnnnnnnnnnnnnnnnnnnnnnnnnnnnnnnnnnnnnnnnnnnnnnnnnnnnnnnnnnnnnnnnnnnnnnnnnnnnnnnnnnnnnnnnnnnnnnnnnnnnnnnnnnnnnnnnnnnnnnnnnnnnnnnnnnnnnnnnnnnnnnnnnnnnnnnnnnnnnnnnnnnnnnnnnnnnnnnnnnnnttgcgcagtcgtgctctcgcatggctatgtttctagcgagaaagacaccctgaaatnnnnnnnnnnnnnnnnnnnnnnnnnnnnnnnnnnnnnnnnnnnnnnnnnnnnnnnnnnnnnnnnnnnnnnnnnnnnnnnnnnnnnnnnnnnnnnnnnnnnnnnnnnnnnnnnnnnnnnnnnnnnnnnnnnnnnnnnnnnnnnnnnnnnnnnnnnnnnnnnnnnnnnnnnnnnnnnnnnnnnnnnnnnnnnnnnnnnnnnnnnnnnnnnnnnnnnnnnnnnnnnnnnnnnnnnnnnnnnnnnnnnnnnnnnnnnnnnnnnnnnnnnnnnnnnnnnnnnnnnnnnnnnnnnnnnnnnnnnnnnnnnnnnnnnnnnnnnnnnnnnnnnnnnnnnnnnnnnnnnnnnnnnnnnnnnnnnnnnnnnnnnnnnnnnnnnnnnnnnnnnnnnnnnnnnnnngaagctaaacgagcctccacattcacgcagtccgcgcctacgccattgccctgcgctccgtctttcttaCGCCCCACGAGGCTGGCGCAGTCGTGTGCGGTCATGGCCATGTTGAGAAATACTCCCTGAAATACATTGCTGGCCTGCGATGnnnnnnnnnnnnnnnnnnnnnnnnnnnnnnnnnnnnnnnnnnnnnnnggcttggggtCCGGTCAGGACCGTGTAGTGGACGGGCATGTACGCCTGCATCCTATAAGATGTCGTTGTTGACTGCGGCTGCGGCTGCGGCTGCGGGTnnnnnnnnnnnnnnnnnnnnnnnnnnnnnnnnnnnnnnnnnnnnnnnnnnnnnnnnnnnnnnnnnnnnnnnnnnnnnnnnnnnnnnnnnnnnnnnnnnnnnnnnnnnnnnnnnnnnnnnnnnnnnnnnnnnnnnnnccgTACGTGCTGGGAGTTCCTGCTGGGGCCTTGCCCTGATTGTCTTTGCCCTCCCAGTCGGTATGAGGCAGGTCGCGTCTGTCCCTTAATGTGTGCCTCCTTGCCTGAGGGCTCCGGCGCCACCAGTTTCAGATGCAATGCCCACGGCATCGCCtgagaggctgaacgggtctccacactcacacagtctgcgccnnnnnnnnnnnnnnnnnnnnnnnnnnnnnnnnnnnnnnnnnnnnnnnnnnnnnnnnnnnnnnnnnnnnnnnnnnnnnnnnnnnnnnnnnnnnnnnnnnnnnnnnnnnnnnnnnnnnnnnnnnnnnnnnnnnnnnnnnnnnnnnnnnnnnnnnnnnnnnnnnnnnnnnnnnnnnnnnnnnnnnnnnnnnnnnnnnnnnnnnnnnnnnnnnnnnnnnnnnnnnnnnnnnnnnnnnnnnnnnnnnnnnnnnnnnnnnnnnnnnnnnnnnnnnnnnnnnnnnnnnnnnnnnnnnnnnnnnnnnnnnnnnnnnnnnnnnnnnnnnnnnnnnnnnnnnnnnnnnnnnnnnnnnnnnnnnnnnnnnnnnnnnnnnnnnnnnnnnnnnnnnnnnnnnnnnnnnnnngctctggttgtggttgaagcggtttgttgcccataGCACGGATGTGTGTGGTGCAAGCCAGGCGTGGGAACCGagggggatgcgatagctgcaccacgctttgctcaagataggcGTGTTGATGACGCCGTCCGAATTACTTCCCTTGTACTGCGCACCTCCGTTATTTATGTAAAAGATGTAGGTGAGGGGGATGTACACGCGTGCTTCGACGCCGGCGTTCAGGCCGGTGAGCAGGTGGGTGTAGGGGTCACCGCTCTTAGTTTCGAGCTTAAGGAATCCGGCAAAATCAAAGTGATCTGCTTGATTCTTAAAGAAAACACGTTCTCCAAAGATATTAGTGCCTGCGGTGGCAAAGTAtatnccagaanagagccaCTTCCACTGCATACGCAGGAGCGCGTCTATGttgagcgttttggtgtacgtgaattgcaaccagGAAATAAAGAGTGCTGTCCGCATCCGCGCGTCAGGATTCTTCTGCTGCTCTATGAGCCTCATGTTTATTTCTAGGCCTTTGCCATTCAGATGTCTTAGGtaaccaccgcttgacgctaagccgacgatgGTGCGCTGCGCCAAGAGCGTTACTAAGCCAGCTTTCTCCAGAAGACTGCCTTTGCTCAGTTCTTGCACAAAGAATGAAACAACATCTTGTTGCGGGGAGGTCAGCAGCGTGTCCCCAAGACTATTGATGACTTCATGGACCCTCTGTGAGGGAGCCATTCTAAAGTAGAATTTTGTGTcactctggtgttggttaccggcgtcgagggcgaaggagaagcggaagccggcgcctggttcgagggnnnnnnnnnnnnnnnnnnnnnnnnnnnnnnnnnnnnnnnnnnnnnnnnnnnnnnnnnnnnnnnnnnnnnnnnnnnnnnnnnnnnnnnnnnnnnnnnnnnnnnnnnnnnnnnnnnnnnnnnnnnnnnnnnnnnnnnnnnnnnnnnnnnnnnnnnnnnnnnnnnnnnnnnnnnnnnnnnnnnnnnnnnnnnnnnnnnnnnnnnnnnnnnnnnnnnnnnnnnnnnnnnnnnnnnnnnnnnnnnnnnnnnnnnnnnnnnnnnnnnnnnnnnnnnnnnnnnnnnnnnnnnnnnnnnnnnnnnnnnnnnnnnnnnnnnnnnnnnnnnnnnnnnnnnnnnnnnnnnnnnnnnnnnnnnnnnnnnnnnnnnnnnnnnnnnnnnnnnnnnnnnnnnnnnnnnnnnnnnnnnnnnnnnnnnnnnnnnnnnnnnnnnnnnnnnnnnnnnnnnnnnnnnnnnnnnnnnnnnnnnnnnnnnnnnnnnnnnnnnnnnnnnnnnnnnnnnnnnnnnnnnnnnnnnnnnnnnnnnnnnnnnnnnnnnnnnnnnnnnnnnnnnnnnnnnnnnnnnnnnnnnnnnnnnnnnnnnnnnnnnnnnnnnnnnnnnnnnnnnnnnnnnnnnnnnnnnnnnnnnnnnnnnnnnnnnnnnnnnnnnnnnnnnnnnnnnnnnnnnnnnnnnnnnnnnnnnnnnnnnnnnnnnnnnnnnnnnnnnnnnnnnnnnnnnnnnnnnnnnnnnnnnnnnnnnnnnnnnnnnnnnnnnnnnnnnnnnnnnnnnnnnnnnnnnnnnnnnnnnnnnnnnnnnnnnnnnnnnnnnnnnnnnnnnnnnnnnnnnnnnnnnnnnnnnnnnnnnnnnnnnnnnnnnnnnnnnnnnnnnnnnnnnnnnnnnnnnnnnnnnnnnnnnnnnnnnnnnnnnnnnnnnnnnnnnnnnnnnnnnnnnnnnnnnnnnnnnnnnnnnnnnnnnnnnnnnnnnnnnnnnnnnnnnnnnnnnnnnnnnnnnnnnnnnnnnnnnnnnnnnnnnnnnnnnnnnnnnnnnnnnnnnnnnnnnnnnnnnnnnnnnnnnnnnnnnnnnnnnnnnnnnnnnnnnnnnnnnnnnnnnnnnnnnnnnnnnnnnnnnnnnnnnnnnnnnnnnnnnnnnnnnnnnnnnnnnnnnnnnnnnnnnnnnnnnnnnnnnnnnnnnnnnnnnnnnnnnnnnnnnnnnnnnnnnnnnnnnnnnnnnnnnnnnnnnnnnnnnnnnnnnnnnnnnnnnnnnnnnnnnnnnnnnnnnnnnnnnnnnnnnnnnnnnnnnnnnnnnnnnnnnnnnnnnnnnnnnnnnnnnnnnnnnnnnnnnnnnnnnnnnnnnnnnnnnnnnnngcaagaactCGTCGCTGCCGTTTTTGAAGAACGCTATGGAGTGCGCGTCTGCTACCTTATGGGCAACACTGTGTGCCCCGGTTGGCGCCGGCGCTGCTGCGGCAGACTGTGGGCAATCCTGAATTTTCCCTTCGCTTTGATTAACTTTTTTGCAGTACACcttgtctttcgtgaggatataaacgtnnnnnnnnnnnnnnnnnnnnnnnnnnnnnnnnnnnnnnnnnnnnnnnnnnnnnnnnnnnnnnnnnnnnnnnnnnnnnnnnnnnnnnnnnnnnnnnnnnnnnnnnnnnnnnnnnnnnnnnnnnnnnnnnnnnnnnnnnnnnnnnnnnnnnnnnnnnnnnnnnnnnnnnnnnnnnnnnnnnnnnnnnnnnnnnnnnnnnnnnnnnnnnnnnnnnnnnnnnnnnnnnnnnnnnnnnnnnnnnnnnnnnnnnnnnnnnnnnnnnnnnnnnnnnnnnnnnnnnnnnnnnnnnnnnnnnnnnnnnnnnnnnnnnnnnnnnnnnnnnnnnnnnnnnnnnnnnnnnnnnnnnnnnnnnnnnnnnnnnnnnnnnnnnnnnnnnnnnnnnnnnnnnnnnnnnnnnnnnnnnnnnnnnnnnnnnnnnnnnnnnnnnnnnnnnnnnnnnnnnnnnnnnnnnnnnnnnnnnnnnnnnnnnnnnnnnnnnnnnnnnnnnnnnnnnnnnnnnnnnnnnnnnnnnnnnnnnnnnnnnnnnnnnnnnnnnnnnnnnnnnnnnnnnnnnnnnnnnnnnnnnnnnnnnnnnnnnnnnnnnnnnnnnnnnnnnnnnnnnnnnnnnnnnnnnnnnnnnnnnnnnnnnnnnnnnnnnnnnnnnnnnnnnnnnnnnnnnnnnnnnnnnnnnnnnnnnnnnnnnnnnnnnnnnnnnnnnnnnnnnnnnnnnnnnnnnnnnnnnnnnnnnnnnnnnnnnnnnnnnnnnnnnnnnnnnnnnnnnnnnnnnnnnnnnnnnnnnnnnnnnnnnnnnnnnnnnnnnnnnnnnnnnnnnnnnnnnnnnnnnnnnnnnnnnnnnnnnnnnnnnnnnnnnnnnnnnnnnnnnnnnnnnnnnnnnnnnnnnnnnnnnnnnnnnnnnnnnnnnnnnnnnnnnnnnnnnnnnnnnnnnnnnnnnnnnnnnnnnnnnnnnnnnnnnnnnnnnnnnnnnnnnnnnnnnnnnnnnnnnnnnnnnnnnnnnnnnnnnnnnnnnnnnnnnnnnnnnnnnnnnnnnnnnnnnnnnnnnnnnnnnnnnnnnnnnnnnnnnnnnnnnnnnnnnnnnnnnnnnnnnnnnnnnnnnnnnnnnnnnnnnnnnnnnnnnnnnnnnnnnnnnnnnnnnnnnnnnnnnnnnnnnnnnnnnnnnnnnnnnnnnnnnnnnnnnnnnnnnnnnnnnnnnnnnnnnnnnnnnnnnnnnnnnnnnnnnnnnnnnnnnnnnnnnnnnnnnnnnnnnnnnnnnnnnnnnnnnnnnnnnnnnnnnnnnnnnnnnnnnnnnnnnnnnnnnnnnnnnnnnnnnnnnnnnnnnnnnnnnnnnnnnnnnnnnnnnnnnnnnnnnnnnnnnnnnnnnnnnnnnnnnnnnnnnnnnnnnnnnnnnnnnnnnnnnnnnnnnnnnnnnnnnnnnnnnnnnnnnnnnnnnnnnnnnnnnnnnnnnnnnnnnnnnnnnnnnnnnnnnnnnnnnnnnnnnnnnnnnnnnnnnnnnnnnnnnnnnnnnnnnnnnnnnnnnnnnnnnnnnnnnnnnnnnnnnnnnnnnnnnnnnnnnnnnnnnnnnnnnnnnnnnnnnnnnnnnnnnnnnnnnnnnnnnnnnnnnnnnnnnnnnnnnnnnnnnnnnnnnnnnnnnnnnnnnnnnnnnnnnnnnnnnnnnnnnnnnnnnnnnnnnnnnnnnnnnnnnnnnnnnnnnnnnnnnnnnnnnnnnnnnnnnnnnnnnnnnnnnnnnnnnnnnnnnnnnnnnnnnnnnnnnnnnnnnnnnnnnnnnnnnnnnnnnnnnnnnnnnnnnnnnnnnnnnnnnnnnnnnnnnnnnnnnnnnnnnnnnnnnnnnnnnnnnnnnnnnnnnnnnnnnnnnnnnnnnnnnnnnnnnnnnnnnnnnnnnnnnnnnnnnnnnnnnnnnnnnnnnnnnnnnnnnnnnnnnnnnnnnnnnnnnnnnnnnnnnnnnnnnnnnnnnngggtagaattttggaaggctgtgtgggttGCCCGCAGCTTTTTGAAAGGTGTGGAGCGAAAAAACGTATGAAACAGCTAAAACAGTGCGCGGCACACGGGatacgcttnnnnnnnnnnngcgcctgcggggtgggtgttgccgcgggtgcannnnnnGCGCAGGAGGTGGCGCCTGCCGCATCGTGGGATGTAACCGCCGCnnnnnnnnnnnnnnnnnnngtttcgtttattggcgcggtgcccctnttccaggtggactgGTGCAATACcgcccgcggggatctgggggcgttgcagccaacgggaataaggaagtttaccccgcgtttggggtgtcttttggatacgagtattttgtgtatcgcggctnnnnnnnnnnnnnnnnnnnnnnnnnnnnnnnnnnnnnnnnnnnnnnnnnnnnnnnnnnnnnnnnnnnnnnnnnnnnnnnnnnnnnnnnnnnnnnnnnnnnnnnnnnnnnnnnnnnnnnnnnnnnnnnnnnnnnnnnnnnnnnnnnnnnnnnnnnnnnnnnnnnnnnnnnnnnnnnnnnnnnnnnnnnnnnnnnnnnnnnnnnnnnnnnnnnnnnnnnnnnnnnnnnnnnnnnnnnnnnnnnnnnnnnnnnnnnnnnnnnnnnnnnnnnnnnnnnnnnnnnnnnnnnnnnnnnnnnnnnnnnnnnnnnnnnnnnnnnnnnnnnnnnnnnnnnnnnnnnnnnnnnnnnnnnnnnnnnnnnnnnnnnnnnnnnnnnnnnnnnnnnnnnnnnnnnnnnnnnnnnnnnnnnnnnnnnnnnnnnnnnnnnnnnnnnnnnnnGTTGCGCGCCGCGCAGTGTCGGGGTATCATGGAAGGCGGTACAGGCACTGnnnnnnnnnnnnnnnnnnnnnnnnnnnnnnnnnnnnnnnnnnnnnnnnnnnnnnnnnnnnnnnnnnnnnnnnnnnnnnnnnnnnnnnnnnnnnnnnnnnnnnnnnnnnnnnnnnnnnnnnnnnnnnnnnnnnnnnnnnnnnnnnnnnnnnnnnnnnnnnnnnnnnnnnnnnnnnnnnGGTGTGGGTTCCCACCTCCTGTTCGTGTCTTTTCAGGGTGTGTGCGCGTTCCGAGAAGAGGGCGTTTTGTGTGTGGGGAGGAGTACGATGGATACGCAGTATATGAGGCGCCGGGTGTGCACGGTggtgcgcgcggtggtgtgtctactcagcacgagtttgctgacaacctgtgatttcactgGCatctttgcggcnnnnnnnnnnnnnnnnnnnnnnnnnnnnnnnnnnnnnnnnnnnnnnnnnnnnnnnnnnnnnnnnnnnnnnnnnnnnnnnnnnnnnnnnnnnnnnnnnnnnnnnnnnnnnnnnnnnnnnnnnnnnnnnnnnnnnnnnnnnnnnnnnnnnnnnnnnnnnnnnnnnnnnnnnnnnnnnnnnnnnnnnnnnnnnnnnnnnnnnnnnnnnnnnnnnnnnnnnnnnnnnnnnnnnnnnnnnnnnnnnnnnnnnnnnnnnnnnAGCTCGGGCAGCACGAGCACCGGCACAACGACAAGCCCCTCGATTGAAACCTGCTCGAATCATGCGACGCTCGTGGGGGGAACGTCCGAGCCCTTCTnnnnnnnnnnnnnnnnnnnnnnnnnnnnnnnnnnnnnnnnnnnnnnnnnnnnnnnnnnnnnnnnnnnnnnnnnnnnnnnnnnnnnnnnnnnnnnnnnnnnnnnnnnnnnnnnnnnnnnnnnnnnnnnnnnnnnnnnnnnnnnnnnnnnnnnnnnnnnnnnnnnnnnnnnnnnnnnnnnnnnnnnnnnnnnnnnnnnnnnnnnnnnnnnnnnnnnnnnnnnnnnnnnnnnnnnnnnnnnnnnnnnnnnnnnnnnnnnnnnnnnnnnnnnnnnnnnnnnnnnnnnnnnnnnnnnnnnnnnnnnnnnnnnnnnnnnnnnnnnnnnnnnnnnnnnnnnnnnnnnnnnnnnnnnnnnnnnnnnnnnnnnnnnnnnnnnnnnnnnnnnnnnnnnnnnnnnnnnnnnnnnnnnnnnnnnntacattctcaccaaagaaaaagtgtactgcagaaagcagaatgccgcttcctccgctgcgtcgtcaccagcccagtgTCCCTCTTCCCCTTCTTCTTCCTCCTCCTCGACGAATGCGGGATGCGAGGTGGCGCACGGGGTGGAAGACCCGCTGTGTCTTGCGAnnnnnnnnnnnnnnnnnnnnnnnnnnnnnnnnnnnnnnnnnnnnnnnnnnnnnnnnnnnnnnnnnnnnnnnnnnnnnnnnnnnnnnnnnnnnnnnnnnnnnnnnnnnnnnnnnnnnnnnnnnnnnnnnnnnnnnnnnnnnnnnnnnnnnnnnnnnnnnnnnnnnnnnnnnnnnnnnnnnnnnnnnnnnnnnnnnnnnnnnnnnnnnnnnnnnnnnnnnnnnnnnnnnnnnnnnnnnnnnnnnnnnnnnnnnnnnnnnnnngccgcgggtggggcgggctgtcctgcaagcgccagcagtaccagcggagggaccagcagcacgcagcgtccagacctctacgccgcagtgggagacacgannnnnnnnnnnnnnnnnnnnnnnnnnnnnnnnnnnnnnnnnnnnnnnnnnnnnnnnnnnnnnnnnnnnnnnnnnnnnnnnnnnnnnnnnnnnnnnnnnnnnnnnnnnnnnnnnnnnnnnnnnnnnnnnnnnnnnnnnnnnnnnnnnnnnnnnnnnnnnnnnnnnnnnnnnnnnnnnnnnnnnnnnnnnnnnnnnnnnnnnnnnnnnnnnnnnnnnnnnnnnnnnnnnnnnnnnnnnnnnnnnnnnnnnnnnnnnnnnnnnnnnnnnnnnnnnnnnnnnnnnnnnnnnnnnnnnnnnnnnnnnnnnnnnnnnnnnnnnnnnnnnnnnnnnnnnnnnnnnnnnnnnnnnnnnnnnnnnnnnnnnnnnnnnnnnnnnnnnnnnnnnnnnnnnnnnnnnnnnnnnnnnnnnnnnnnnnnnnnnnnnnnnnnnnnnnnnnnnnnnnnnnnnnnnnnnnnnnTTCTTGGACTGCGCGTGTACTGTCTGTTGCCGGAAAGATATGTTCAATCGTGAGGGTTTTGTCTGCGCGCTGCACTCGTTTGCGTGCGCCCTTTGTCATATCGAGGATAGTTCCGTTCAAGTCGATGATAATAACnnnngtggggataagcnnnnnnnnnnnnnnnnnnnnnnnnnnnnnnnnnnnnnnnnnnnnnnnnnnnnnnnnnnnnnnnnnnnnnnnnnnnnnnnnnnnnnnnnnnntctgcgttcctaatacaccaaactttttaaaatgactctctccaatacnnnnnnnnnnnnnnnnnnnnnnnnnnnnnnnnnnnnnnnnnnnnnnnnnnnnnnnnnnnnnnnnnnnnnnnnnnnnnnnnnnnnnnnnnnnnnaagcagcagcnnnnnnnnnnnnnnnnnnnnnnnnnnnnnnnnnnnnnnnnnnnnnnnnnnnnnnnnnnnnnnnnnnnnnnnnnnnnnnnnnnnnnnnnnnnnnnnnnnnnnnnnnnnnnnnnnnnnnnnnnnnnnnnnnnnnnnnnnnnnnnnnnnnnnnnnnnnnnnnnnnnnnnnnnnnnnnnnnnnnnnnnnnnnnnnnnnnnnnnnnnnnnnnnnnnnnnGATTTGGGTTAATTCCCGCAAATCACGATTGTGGCCAAGCACAAACATCCGATCGGCACGTCTGAAGGTGAACGCGTTATCCGGGTAGTAAATATTCTTTTTCGGAAGGGAGTTTCCCGTCGGGTCGATATCCATATCTGTGCGCACCACTGCCACAATCTTCACGTTAAACTTTTCTctcaattccagttcttcaagtgnnnnnnnggtaaaaaagcgtggcgcaacaatttcccgtaaggaaatatCGTCTGTCATAGCAATGGCATCCGTGACGTCAGGAGCAACCAatcgcttggcgatggttgtagcactcgctacttcgatgtttaggatttcgtttgcgcctATCTTGCGCAAGATAGTTTCGTGTATTGGAGAAGTTGCACGCGCAAGGATGTAAGGAATATCCCGTTGCTTCAACAGCGCTGTGGTAAGAATGCTCGCCTGCAGATTGGTTCCAATAGCAACGATTGCGACGTCTACGTCATCCAGCGGCGCTTTCATGAGCTCTGTTTCGCTTGTGGTGTCTATGAGAATAGCAACGGATACAACCTTTTTGACCTTCTCAACGGTCTGGGCATTGTTGTCTATCGCGACAACACTTCCCCCACCTTGGACAAGTACTTCGCAAACGCGATAGCCAAAAGCCCCTAAGCnnnnnnnnnnnnnnnnnnnnnnnnnnGCCATGATGTTCCTTTCACGTCGCGCATGCACACGTTACGCGCATCAACCAATGGCTAAGTCCCCACACGCGTATTCGACGCTCATATCCTGTTCTTCTTTACTTGCTGCAGTTAAAAnnnnnnnnnnnnnnnnnnnnnnnnnnnnnatgannnnnnnnnnnnnnnnnnnnnnnnnnnnnnnnnnnnnnnnnnnnnnnnnnnnnnnnnnnnnnnnnnnnnnnnnnnnnnnnnnnnnnnnnnnnnnnnnnnnnnnnnnnnnnnnnnnnnnnnnnnnnnnnnnnnnnnnnnnnnnnnnnnnnnnnnnnnnnnnnnnnnnnnnnnnnnnnnnnnnnnnnnnnnnnnnnnnnnnnnnnnnnnnnnnnnnnnnnnnnnnnnnnnnnnnnnnnnnnnnnnnnnnnnnnnnnnnnnnnnnnnnnnnnnnnnnnnnnnnnnnnnnnnnnnnnnnnnnnnnnnnnnnnnnnnnnnnnnnnnnnnnnnnnnnnnnnnnnnnnnnnnnnnnnnnnnnnnnnnnnnnnnnnnnnnnnnnnnnnnnnnnnnnnnnnnnnnnnnnnnnnnnnnnnnnnnnnnnnnnnnnnnnnnnnnnnnnnnnnnnnnnnnnnnnnnnnnnnnnnnnnnnnnnnnnnntatgttcgagtagatagataccaacaaaagaaacagcaagaagcagaacagttgctttcaacacgacaaaagtattcaggctgagagagccgaggatattttttcccttaaaaaaaatgtttttgataCTTCCGTGGACACGGCACAGTACTTCATCGATGACCACAAAACTGATTCCCCCCAATATGATGGTAAAGGCAATGGGGAACGTTATCCATGGGTTGGAAACGAACGATTCCAAACTGTTTGGGTACAGGGAGAACCCCGCATTGCAAAACGCAGATATCGCATGGAAGnnnnnnnnnnnnnnnnnnnnnnnnnnnnnnnnnnnnnnnnnnnnnnnnnnnnnnnnnnnnnnnnnnnnnnnnnnnnnnnnnnnnnnnnnnnttnagaaaataatgagctttaggttatgggataggctgcccatgtcactgtcgctcaccagatgcgaggcgacgagtctGCTTTCGAGGCTCATCTTTTTCTTTATGGTGATCATACCAAAAAACGAAAACACCATAAATCCTAGTCCCCcnnnnnnnnnnnnnnnnnnnnnnnnnnnnnnnnnnnnnnnnnnnnnnnnnnnnnnnnnnnnnnnnnnnnnnnnnnnnnnnnnnnnnnnnnnnnnnnnnnnnnnnnnnnnnnnnnnnnnnnnnnnnnnnnnnnnnnnnnnnnnnnnnnnnnnnnnnnnnnnnnnnnnnnnnnnnnnnnnnnnnnnnnnnnnnnnnnnnnnnnnnnnnnnnnnnnnnnnnnnnnnnnnnnnnnnnnnnnnnnnnnnnnnnnnnnnnnnnnnnnnnnnnnnnnnnnCAAAGATACCGAGCGTAATATTCTGTATATGTGTCAGCGTGGTGTAAAGATCACCGGAGGCAGTGGTAAGCGTCACACTTGCACGCACGAAGGTAAGGnnnnnnnnnnnnnnnnnnnnnnnnnnnnnnnnnnnnnnnnnnnnnnnnnnnnnnnnnnnnnnnnnnnnnnnnnnnnnnnnnnnnnnnnnnnnnnnnnnnnnnnnnnnnnnnnnnnnnnnnnnTCCTACAATCGTGAAAAACTGCACCGAGAACGGGAGGAATTGATCACACAAACGAATCGCAAGGCACAACACGAGCATCGTAAGATGAGTGAGATTGCCAATATGCATGGnnnnnnnnnnnnnnnnnnnnnnnnnnnnnnnnnnnnnnnnnnnnnnnnnnnnnnnnnnnnnnnnnnnnnnnnnnnnnnnnnnnnnnnnnnnnnnnnnnnnnnnnnnnnnnnnnnnnnnnnnnnnnnnnnnnnnnnnnnnnnnnnnnnnnnnnnnnnnnnnnnnnnnnnnnnnnnnnnnnnnnnnnnnnnnnnnnnnnnnnnnnnnnnnnnnnnnnnnnnnnnnnnnnnnnnnnnnnnnnnnnnnnnnnnnnnnnnnnnnnnnnnnnnnnnnnnnnnnnnnnnnnnnnnnnnnnnnnnnnnnnnnnnnnnnnnnnnnnnnnnnnnnnnnnnnnnnnnnnnnnnnnnnnncccgggtaaaacggcgctcttgcgtgagacgcagtcccaaattcacgcttacttttgtggagcgCTGCGCGAGTTTTCAGTACCGGTCACGGTAGAGGGCACGCCTTTTATGCAACGCGTCTGGGGCGCCACGCGCAACATCGCGTACGGCACGACAAAAAGCTATGCACAGCTTTCACAGGACATCGGCTGTCCACGCGCCGCACGCGCAGTTGGGCAGGCATTGCATCGCAACCCGCTGCTTTTGCTTATTCCTTGCCATCGGGTAAtaagtgcgcgtgcatcaatcggcggttttgcgtacgggagTGCTCTCAAGCATTTTTTGCTGCAGCAAGAGGCGGCTGTGTGCGCATGTGAATGACggtgtccgcaagggctgccgcttnnnnnnnnnnnnnnnnnnnnnnnnnngcagtgtacccaagtgaacgctGCAGCGCGCGCAGCTCTCCTTGCAacgtagtccgtaaatctgcgtctattGCAGAAAGCGCTTCGTCAAACAATATGAGCGCAGGGCGGACCGCAAGGCTACGTGCAAGCGCCACCCGTTGCTTTTCCCcatgggacagcgtggttaccgnnnnnnnnnnnnnnnnnnnnnnnnnnnnnnnnnnnnnnnnnnnnnnnnnnnnnnnnnnnnnnnnnnnnnnnnnnnnnnnnnnnnnnnnnnnnnnnnnnnnnnnnnnnnnnnnnnnnnnnnnnnnnnnnnnnnnnnnnnnnnnnnnnnnnnnnnnnnnnnnnnnnnnnnnnnnnnnnnnnnnnnnnnnnnnnnnnnnnnnnnnnnnnnnnnnnnnnnnnnnnnnnnnnnnnnnnnnnnnnnnnnnnnnnnnnnnnnnnnnnnnnnnnnnnnnnnnnnnnnnnnntaacagtgcgagcgatgtgccttgcctcagcgagaaagacaggctgatccttttttcttcccatacttttgtcagttcttgtactactacgtacggcctattatccataggagtattccttacatcggtgtacgcctattaaaagnnnnnnnnnnnnnnnnnnnnnnnnnnnnnnnnnnnnnnnnnnnnnnnnnnnnnnnnnnnnnnnnnnnnnnnnnnnnnnnngtaatgctacgctttgaaagtcaggaatttctaacacgagcgGAAATGCAGCGTCCGCCGCACTGAGTGCAAAGGCGTATACGCCAGCACTTACAATTCCTTTTTTACACAAGGGGCATATAGTACGTAAGAAAAAATCTAGTCGGTTGCGCGACAACATCTGTCCTGTAAAAATAACAGGTGTAGGAATGCGCATGAGCGTTGAGTACAGTGACAACCATGCGAAGGGAAATGCAAGTGCACTTTGGGCACACACCAAGAGTATGCGCGAAGCACACCGCGGCATGAGCGACCAGCCAAAGGCGAGCACCATAGGAGAAACTGCGAGGGGTAGAAAGGGTAGGCGTGTTTTCCATCTGGAAACGTGTCGCATGAGTACCATATATCCGTAACACAGCACGCTACCGACAGTGACGCAAAAACTCCAAAAGCCGATCCATATGGTGCTGCGCAGCGCATGCCAGAACGATGGCGCAGAAAAAAGGGTGTGCCATGCAGTGAGGGTGAACGATACGGTCCTTCCTGCACGGTGGCGcgnnnnnnnnnnnnnnnnnnnagaaaaaAGAGGGAGAATGAGAAAAAAAAGAATTCCGCACAGCAGTGCAAAAAATCCTATGCGCTCAATGTTTCCCTGTATGCTTTGTGTGGGCAGAGGGCGTGTGCGCGCTACGCACGACTGCGCCTGCTGCGATTCTTCTGTGGAAACGCCCCAGATCATAAGCngtgcgtaaaacgtctgacnnnnnnnnnnnnnnnnnnnnnnnnnnnnnnnnnnnnnngttcgtatactgcgatagnnnnnnnnnnnnnnnnnnnnnnnnnnnnnnnnnnnnnnnnnnnnnnnnnnnnnnnnnnnnnnnaagcagtacaaaaaaataacaagaaaggaagaacgcagtgcgccggcgagtgcaggaaGGGTGATGGTATagaaAACTCTATACGCGCGCGCGCCTAACAGGCGCGCGGCGTCTTCAGCGTCTGTGGAGATGGTATTCCAGGCTCTAGCGATGATAGCGAGTGCGAGGGGAAAATTATACCAGGCGTGCGCAATCACCACGCCAGAGGCAGAATATAAAAAGGTGCGTGGCGTAGAGACACCCCagagtgtgccgagcgtacgcgnnnnnnnnnnnnnnnnnnnnnnnnnnnnnnnnnnnnnnnnnnnnnnnnnnnnnnnnnnnnnnnnnnnnnnnnnnnnnnnnnnnnnnnnnnnnnnnnnnnnnnnnnnnnnnnnnnnnnnnnnnnnnnnnnnnnnnnnnnnaaaacctactgcgcacgcgagcaccgctgagcagagtgcttGAAAAACGCTCTGCGCGAACAGTGCCCCAAAGCGTGCGGCACCAGGCGTGCCGGGAGGGAAGAAGAAAGGAGTGATGGGGGAACCTGTCTGCAAAGCTCCCGGTGAGGGATGTGCCGCAGTGTTTTCTAATCTCGGTTTAGAGAAAAAGGCGCTTCCCGCCGTGAACACGGGGATAAGGAATGTGCCTATACTCAGCGAGAAGCACAACGCACAGATGAGAGCAACACAGTAGTTGCGCGCGTCCCTTCCTCTTGTCAGATAGAGAGCTCCAGACGCACACGAGCggcggcacgGATGTAAGAGCGTGGTCATGGGGTTGATGCGTGTAATGCCGCTGCTGCAGCGCGGCACATCTGCGGCGAGAGAGACACGCAGGCAGCAGGTTGAGGGACTGCAGAGAATGCGGCAGGCAACGGTACGTCAGGATTGGCCGGGTACATCCACTGAGTGAGGGGAAGGAGCTCCTGCGCTTCAGTGCTGAGCAGAAAGTCGATAAACTGAGTAGCAACCCGCGCATGTTTCGTACGCTTCACCAGGCTAATACCTTCGATAtgcatggggtggccctcgggaannnnnnnnnnnnnnnnnnnnnnnnnnnnnnnnngGTGCAGGTGGTACGCTGCACTTGTGGTGTAACTAAGTACCAGTGCGGCTTCCCCTGCGGTGAACAAGCCGTATGCGGCGCTCCAGCTCGGGGCGAGCGCAAGGAGATTCGCGCGATAGAGCGTATTCCAGAACGCAGGCCATGCAGCGCCGAGTGATGTGTGGGCCCAACCGATAAAGATCGTACCAAGCACGTTAGTATGCGGGtctgcaagtgcaatgctttttgcgtagacgggatttgcaagatnnnnnnnnnnnnnnnnnnnnnnnnnnnnnnnnnnnnnnnnnnnnnnnnnnnnnnnnnnnnnnnnnnnnnnnnnnnnnnnnnnnnnnnnnnnnnnnnnnnnnnnnnnnnnnnnnnnnnnnnnnnnnnnnnnnnnnnnnnnnnnnnnnnnnnnnnnnnnnnnnnnGGGGAGGGTGTATTGGTCAACCCCGATGATAAGATCGGCACCTCTGGATGACGGCGTGTGCCGATCGCGGATGGCACGATGAATAGCGCGCGTGACGTCCTCAAACACCACGTACTGAACGGAATACCCTGTTTTCGCGTAGAAGCGCCGCGCAAGTTCAGGGCCAGGTCCCCATTCAGAAGCAAAGGAGTCGTAGACatagnnnnnnnnnnnnnnnnnnnnnnnnnnnnnnnnnnnnnnnnnnnnnnnnnnnnnnnnnnnnnnnnnnnnnnnnnnnnnnnnnnnnnnnnnnnnnnnnnnnnnnnnnnnnnnnnnnctatagcatagctctagagctcctatccagggtgaggggtacagtgctgctgtcttgctcaaagGGAGTGCATTCGGTACACTCGCCCGCATGAGTGTGTGTTGTACGTCCTCCGAAGAGCGGATTGAACGGAGCnagaatctcgttcaccttgtcgcgcaggttgctggtgcgtacgcgcagctgcctgctcagtatgctaaaagtagctcaggnnnnnnnnnnnnnnnnnnnnnnnnnnnnnnnnnnnnnnnnnnnnnnnnnnnnnnnnnnnnnnnnnnnnnnnnnnnnnnnnnnnnnnnnnnnnnnnnnnnnnnnnnnnnnnnnnnnnnnnnnnnnnnnnnnnnnnnnnnnnnnnnnnnnnnnnnnnnnnnnnnnnnnnnnnnnnnnnnnnnnnnnnnnnctaccgagcgtgatcttgtgcagattctcggtgcggtagaggcacgcnnnnnnnnnGTGGAGAATGACGCGCAGTTGAAAAAACTCGCGCGGTGTTTTGATGCGTTGCCGAGCGTGTCGCAGATCGTGTTGTTGGATGCGGGCGGTGCGTAccaggcgatgaaggatgagtttgtccagaatgaggacggttcnGCTCGGAAGTGCTCTTTTTTCACCTACCAGGACATACTCGCGcatgggacacagtttcgggtgcaccatccaggcaggatagaggatgagattgggcgtaccacgcgcgcggacgttgctactattattttcacatccggtaccaCGGGCAcCCCGAAGGGAGTGGTGCTTTCGCACGAGAATTTTCTTTGTCAGTTAATCGATATCTCCCGCAGGCTGACCGTGTGTCCGGGGGATATCGCGTTGTCAGTGTTGCCTGTGTGGCACGTTTTTGAGCGAATTAGCGAGTATGTGGTACTGTCCCACGCAGGGGGCGTTGCTTACTCCAAGCCGGTAGGGTCGGTGATGCttgcggatttggcaaagcttaatcctcactttttgccttctgtgcctnnnnnnnnnnnnnnnnnnnnnnnnnnnnnnnnnnnnnnnnnnnnnnnnnnnnnnnnnnnnnnnnnnnnnnnnnnnnnnnnnnnnnnnnnnnnnnnnnnnnnnnnnnnnnnnnnnnnnnnnnnnnnnnnnnnnnnnnnnnnnnnnnnnnnnnnnnnnnnnnnnnnnnnnnnnnnnnnnnnnnnnnnnnnnnnnnnnnnnnnnnnnnnnnnnnnnnnnnnnnnnGGAAATgtcttggtgttccgcaaggtacggaagaagtttggtacctcctttaagactgctatctcangggggggcgcgcttcctccaaatgtggatgagtttttgtacgctattggcgtgagaGngcttgaggggtacggtctgacggaaacggcgccggtaattgccatgcncagtgagcgcaggccagtgttcggttccgtgggtacaccctgcgcgtacaATGAAGTGAAGATTGTGGATGACACTGGCGCCCAGCTTCCGGTGGGGTATAAGGGGATAGTGCTTGTACGCGGCAAAAATATAATGCAGGGGTACTATAAAAATCCTGAGCTTACCGCGCAGGTGTTAGATGCAGACGGTTGGTTTAATACgggggatattggctacnnnnnnnnnnnnnnnnnnnnnnnnnnnnnnnnnnnnnnnnnnnnnnnnnnnnnnnnnnnnnnnnnnnnnnnnnnnnnnnnnnnnnnnnnnnnnnnnnnnnnnnnnnnnnnnnnnnnnnnnnnnnnnnnnnnnnnnnnnnnnnnnnnnnnnnnnnnnnnnnnnnnnnnnnnnnnnnnnnnnnnnnnnnnnnnnnnnnnnnnnnnnnnnnnnnnnnnnnnnnnnnnnnnnnnnnnnnnnnnnnnnnnnnnnnnnnnnnnnnnnnnnnnnnnnnnnnnnnaggaaatagcaaagcggatATCACTCGAGAACGGGTTTAAGATATTCGAGCGGATTAACCGTTTTGTGCTTCTGCCGAAGGTCTTTGAAGTGGGAGTGGAGCTTTCCGCAAAGCAGgaggtaatgcgccaccgggtcgccgacctgtacgcnnnnnnnnnnnnnnnnnnnnnnnnnnnnnnnnnnnnnnnnnnnnnnnnnnnnnnnnnnnnnnnnnnnnnnnnnnnnnnnnnnnnnnnnnnnnnnnnnnnnnnnnnnnnnnnnnnnnnnnnnnnnnnnnnnnnnnnnnnnnnnnnnnnnnnnnnnnnnnnnnnnnnnnnnnnnnnnnnnnnnnnnnnnnnnnnnnnnnnnnnnnnnnnnnnnnnnnnnnnnnnnnnnnnnnnnnnnnnnnnnnnnnnnnnnnnnnnnnnnnnnnnnnnnnnnnnnnnnnnnnnnnnnnnnnnnnnnnnnnnnnnnnnnnnnnnnnnnnnnnnnnnnnnnnnnnnnnnnnnnnnnnnnnncttTTGGTGGTGGAGTTGCGATGCTTCCGCTTTTGAAGCGTTTGCTCTGCGAACACAAAAAGTGGGTGGATGAGGAAGAGTTGGTGAATTGTTTTGCCATCGGTCAGTGTACACCTGGCATTATTGCGGTAAACGTCGCTACCTTTTGCGGtgnnnnnnnnnnnnnnnnnnnnnnnnnnnnnnnnnnnnnnnnnnnnnnnnnnnnnnnnnnnnnnnnnnnnnnnnnnnnnnnnnnnnnnnnnnnnnnnnnnnnnnnnnnnnnnnnnnnnnnnnnnnnnnnnnnnnnnnnnnnnnnnnnnnnnnnnnnnnnnnnactgatgcggacgcttttcgcgcttaggaaaaaatcgttacccgatgcaggtgcctgggttactgccatgggtacgtatgggatgatgcatgtatttcattttccctccctccnngccatgttaagtgcagcgcttgcaggattggcaataaaaaccgtacgtatgtggtgtgcgtcacgcgtatgaacttgtggtctttgtgctttttATTCATGTAnnnnnnnnnnnnnnnnnntggaggtggcttggtatcCCTCAGCGTCATGCAGCGTGAGCTTTTAATCCGGGGTATGGTGAGTGCCGAGCATTTTTATTCAATGGTTGCTATTTCAGAGTCTACACCTGGACCAATTGGAATTaacttGGCAACCTACGTGGGCTACGAGnnnnnnnnnnnnnnnnnnnnnnnnnnnnnnnnnnnnnnnnnnnnnnnnnnnnnnnnnnnnnnnnnnnnnnnnnnnnnnnnnnnnnnnnnnnnntttcaacacagcgtgatacttacgcGCATGCTGTATggtctgcgtgccggtgccttgggtatgatcgcaggcgctgtggggactgtGTTTTCTATAGCAGTATTTCCCTATGCACAGTTTTTTCAGTTTGTAATTGGAAAAAGAAATACACCACCATCGCTCGATGTGCATACACTcacgctgtttttggtgttatgcgcactgagctnnnnnnnnnnnnnnnnnnnnnnnnnnnnnnnnnnnnnnnnnnnnnnnnnnnnnnnnnnnnnnnnnnnnnnnnnnnnnnnnnnnnnnnnnnnnnnnnnnnnnnnnnnnnnnnnnnnnnnnnnnnnnnnnnnnnnnnnnnnnnnnnnnnnnnnnnnnnnnnnnnnnnnnnnnnnnnnnnnnnnnnnnnnnnnnnnnnnnnnnnnnnnnnnnnnnnnnnnnnnnnnnnnnnnnnnnnnnnnnnnnnnnnnnnnnnnnnnnnnnnnnnnnnnnnnnnnnnnnnnnnnnnnnnnnnnnnnnnnnnnnnnnnnnnnnnnnnnnnnnnnnnnnnnnnnnnnnnnnnnnnnnnnnnnnnnnnnnnnnnnnnnnnnnnnnnnnnnnnnnnnnnnnnnnnnnnnnnnnnnnnnnnnnnnnnnnnnnnnnnnnnnnnnnnnnnnnnnnnnnnnnnnnnnnnnnnnnnnnnnnnnnnnnnnnnnnnnnnnnnnnnnnnnnnnnnnnnnnnnnnnnnnnnnnnnnnnnnnnnnnnnnnnnnnnnnnnnnnnnnnnnnnnnnnnnnnnnnnnnnnnnnnnnnnnnnnnnnnnnnnnnnnnnnnnnnnnnnnnnnnnnnnnnnnnnnnnnnnnnnnnnnnnnnnnnnnnnnnnnnnnnnnnnnnnnnnnnnnnnnnnnnnnnnnnnnnnnnnnnnnnnnnnnnnnnnnnnnnnnnnnnnnnnnnnnnnnnnnnnnnnnnnnnnnnnnnnnnnnnnnnnnnnnnnnnnnnnnnnnnnnnnnnnnnnnnnnnnnnnnnnnnnnnnnnnnnnnnnnnnnnnnnnnnnnnnnncccgtacaccatcgaaGTTAAAGTCGAACGTAATGAGAGAATTATTAAACACACCAGGAGtgcgtgccactttttctgcagtcaggaggtatttatatgcnnnnnnnnnnnnnnnnnnnnnnnnnnnnnnnnnnnnnnnnnnnnnnnnnnnnnnnnnnnnnnnnnnnnnnnnnnnnnnnnnnnnnnnnnnnnnnnnnnnnnnnnnnnnnnnnnnnnnnnnnnnnnnnnnnnnnnnnnnnnnnnnnnnnnnnnnnnnntacaacaaccgnnnnnnnnnnnnnnnnnnnnnnnnnnnnnnnnnnnnnnnnnnnnnnnnnnnnnnnnnnnnnnnnnnnnnnnnnnnnnnnnnnnnnnnnnnnnnnnnnnnnnnnnnnnnnnnnnnnnnnnnnnnnnnnnnnnnAAAGGGAGagttcggtttttgcacgtattgaataaatgttttaaaccaagtggatgcattgtcgtgtgtaaaaaataatttttggtagcagtcgagcggaagcatgaaAATGAGCACngtaccgggggtgtcgccgtaccgtgcGACAAGTTGCGCATAGAATGTTTCAGCAGGGGTGTCAATCGTCAGTTCGCGGTACGGTAATGCAACAAGGCATTTGCTGCTGTCCTCAAGTACTGCAGGTTTAGTAGCGGGCATATCAGAGGCTTCGAAGAGACGGTAATCTAGGAGACAGtattccattccggcctttacgagtgagggaatgagcgcggcgctccatgcagatgcttccagaaagcagccccgtgggcgttttataacgtgttctcgcaaggtaGAAGTGAGtAGCTCAATTTGACTAATTACATCCGCCTGAGGGAGGAGGGGAAGATACGGCTGGTAAAAACCGCCGCTGAGTAATTCAAGCCGTTTGGAACCAAGCAGATTGTTGATAAGATAAGGATagggagaattctttttttgtataannnnnnnnnnnnnnnnnnnnnnnnnnnnnnnnnnnnnnnnnnnnncgTGCCTGCACAGACCTGAAAAAAATGCCTGGTACTGAGAAGGATCGTGCTCTGCGTGCAGGGACTGAACacatgctcccgttaacttcgcagcaaatgcgatacgtattttggtattgtctccttttttcataatcgggttctcatccccnnnnnnnnnnnnnnnnnnnGCCACAGCTCGTCAGTACCGTACAGGCTCAGTGCAATAAAACccatacttGCTAAGAGAAGTGCAGCTGTTTTCTCCACTGCAGTGCCGTTTCCCTGCGTGATGCGCTGATGTATnnnnnnnnnnnnnnnnnnnnnnnnnnnnnnnnnnnnnnnnnnnnnnnnnnnnnnnnnnnnnnnnnnnnnnnnnnnnnnnnnnnnnnnnnnnnnnnnnnnnnnnnnnnnnnnnnnnnnnnnnnnnnnnnnnnnnnnnnnnnnnnnnnnnnnnnnnnnnnnnnnnnnnnnnnnnnnnnnnnnnnnnnnnnnnnnnnnnnnnnnnnnnnnnnnnnnnnnnnnnnnnnnnnnnnnnnnnnnnnnnnnnnnnnnnnnnnnnnnnnnnnnnnnnnnnnnnnnnnnnattccgcctaggaaaaggaagcgacatgtatggaagagcgtgcaacgtggagagagcgcagCGATGCACAGGCGATCTAATCCGATGCCCTCGGCAAAGAaaagggcgcatacgaaaaacaggggtggcaccattgtcaccattcactccttaaagagggcgtgtggtnnnnnnnnnnnnnnnnnnnnnnnnnnnnnnnnnnnnnnnnnnnnnnnnnnnnnnnnnnnnnnnnnnnnnnnnnnnnnnnnnnnnnGCTGGGTGGAAAAGAGATGATGCGCAAGAACCCCGACGGGGCCGGTAGCGAAACGCACCCGAAGGCAACTAATTCACGTACAAGAGAAATTCCCGCAATCATGATGCCGAAGACACCTACGCATTCGAGCGATTCGGCATCTGCATCGTATgcggtaagacagnnnnnnnnnnnnnnnnnnnnnnnnnnnnnnnnnnnnnnnnnnnnnnnnnnnnnnnnnnnnnnnnnnnnnnnnnnnnnnnnnnnnnnnnnnnnnnnnnnnnnnnnnnnnnnnnnnnnnnnnnnnnnnnnnnnnnnnnnnnnnnnnnnnnnnnnnnnnnnnnnnnnnnnnnnnnnnnnnnnnnnnnnnnnnnnnnnnnnnnnnnnnnnnnnnnnnnnnnnnnnnnnnnnnnnngcaaaggttccggttgcaggaacaaatagtccnnnnnnnnnnnnnnnnnnnnnnnnnnnnnnnnnnnnnnnnnnnnnnnnnnnnnnnnnnnnnnnnnnnnnnnnnnnnnnnnnnnnnnnnnnnnnnnnnnnnnnnnnnnnnnnnnnnnnnnnnnnnnnnnnnnnnnnnnnnnnnnnnnnnnnnnnnnnnnnnnnnnnnnnnnnnnnnnnnnnnnnnnnnnnnnnnnnnnnnnnnnnnnnnnnnnnnnnnnnnnnnnnnnnnnnnnnnnnnnnnnnnnnnnnnnnnnnnnnnnnnnnnnnnnnnnnnnnnnnnnnnnnnnnnnnnnnnnnnnnnnnnnnnnnnnnnnnnnnnnnnnnnnnnnnnnnnnnnnnnnnnnnnnnnnnnnnnnnnnnnnnnnnnnnnnnnnnnnnnnnnnnnnnnnnnnnnnnnnnnnnnnnnnnnnnnnnnnnnnnnnnnnnnnnnnnnnnnnnnnnnnnnnnnnnnnnnnnnnnnnnnnnnnnnnnnnnnnnnnnnnnnnnnnnnnnnnnnnnnnnnnnnnnnnnnnnnnnnnnnnnnnnnnnnnnnnnnnnnnnnnnnnnnnnnnnnnnnnnnnnnnnnnnnnnnnnnnnnnnnnnnnnnnnnnnnnnnnnnnnnnnnnnnnnnnnnnnnnnnnnnnnnnnnnnnnnnnnnnnnnnnnnnnnnnnnnnnnnnnnnnnnnnnnnnnnnnnnnnnnnnnnnnnnnnnnnnnnnnnnnnnnnnnnnnnnnnnnnnnnnctngcncagggagtanaaagaatgcagtaaacagcgcgccgctttttatgagggaaacaagggttgcagggtgtgcntgtgcgggtaaaaaaannnnnnnnnnnnnnnnnnnnnnnnnnnnnnnnnnnnnnnnnnnnnnnnnnnnnnnnnnnnnnnnnnnnnnnnnnnnnnnnnnnnnnnnnnnnnnnnnnnnnnnnnnnnnnnnnnnnnnnnnnnnnnnnnnnnnnnnnnnnnnnnnnnnnnnnnnnnnnnnnnnnnnnnnnnnnnnnnnnnnnnnnnnnnnnnnnnnnnnnnnnnnnnnnnnnnnnnnnnnnnnnnnnnnnnnnnnnnnnnnnnnnnnnnnnnnnnnnnnnnnnnnnnnnnnnnnnnnnnnnnnnnnnnnnnnnnnnnnnnnnnnnnnnnnnnnnnnnnnnnnnnnnnnnnnnnnnnnnnnnnnnnnnnnnnnnnnnnnnnnnnnnnnnnnnnnnnnnnnnnnnnnnnnnnnnnnnnnnnnnnnnnnnnnnnnnnnnnnnnnnnnnnnnnnnnagttgtgtgcggggagtactgcaccgatgagcaGGCCCAGTACGAGAGAGTCGCTCAGGGGCACATGTGGTGTGCGTAGTGTTTGAACGAGCGCGGCGCTCAGTGCGCCGAGTTCGGTACTGACGATGGAAAAAAGCGCGACCGTGTCGCnnnnnnnnnnnnnnnnnnnnnnnnnnnnnnnnnnnnnnnnnnnnnnnnnnnnnnnnnnnnGTCCAGCACTTAAGCCGGTGTaaaggaagggtgcgtgTTTGTACTCAGCGGTGCGCATTGGCAGGTGCCTCGCGGATAGTTTTCCCGACGCTTTGTGTGGAGTGGGGTTCTATTTTGGTGACTGGAGCACGGGAAAGATGCAGTGCGCGTTCTTGTGCATCGTGCAAAAGTTTTGCAAGAGGAATACGCGCAGTACAGGCGGCAGaACACAGACCGCATTGGTGGCAAATCCGCAGGGATTggagcacttcttcagtaaactgattacggtgtgcggcacgcgCAATTTTTATTGggtcaagatatactgggcaaatgcgcgcgcaatcaccacagttttgacagGAGGTACAGGACGCAGAgcttgaaagcgctttaccggtgacgtggagcgatttgatcccctttgagaaaggcaggtccaacgactctaannnnnnnnnnnnnnnnnnnnnnnnnnnnntgagatgcccggggcnngngcgaAACCCTCCACactcctcgatgagcgcgccaaggggggtgccgatacgcgcgcggagtacctgcggtgaTTTTAATCCAGCGCCTGTCAGAGCAATGTAGGAACTGATTTGCGGCTGACTGAGTACCACTGCCTCATACACGTGCACTGCAGTGAGTGCATCGATTGTGCAACCTGCACGATAGCGTGGGGCAGATAGATTGCTAAAAGGATAATGTCGTCTATACGCGTCGTGCATCTCGTTGAATGGAAAGACAGCGCGCATCCTTGCAAGACCGGGGGAGCGTTTTGGCAGCGTGTGGAGACACGCGGTATACGTAGCGTCCAGTATCCGTGCAATAATGGCGGTACCGGTAGCAAcaggttctggnaactcctgcgcaagGAACGAACTCAAAGGGCAGGTAGCGTCGTCGTCACAAAGGTAGAGCGTAAGtTTCCGTACACCCTTACGCacgtgcaGGTGGAGGTGCGTGGCGAGCGCATTGCCGCTTTCCCACGCCGCGCGCATGAGACGCAGCGTGTGCGCTTCAGCCCTTCTCTTCTGTCCGTAGTCTTTCGCGTACGTGTGCTGTGCACCCGCGTGCGTGCCCGTTGCGGTGCTCGTGATCTGGGCGTGCGGATGTAGGCGTGTGGCCACTACTCCTGCATCTGTAACTAGGCGCAGCAATTCTGAAGCGGTGCTGTGCTGCCACGGGCGATTTGGTTGGACCTtgnnnnnntgttcgaaggnnnnnnnnnnnnnnnnnnnnnnnnnnnnnnnnnnnnnnnnngcgagaaaattagcacttaccaagcgggtgacgacaccggggacggaGGCATGTGCGTGAGCAGCACCGGCGTGCGCGGCGCGTGCGATCAGCTGACCTTCTCGGACCGTATCCCCAACCTTTACCACAGCGACGGCGGCTGGCTCTCCAGcaTCAACGGAGAACGGAACGAGCGCGCGCGCAGGCAAAAAAGCATTCCCCCCAAAGCTGAGGGGGAAAATCnnnnnnnnnnnnnnnnnnnnnnnnnnnnnnnnnnnnnnnnnnnnnnnnnnnnnnnnnnnnnnnnnnnnnnnnnnnnnnnnnnnnnnnnnnnnnnnnnnnnnnnnnnnnnnnnnnnnnnnnnnnnnnnnnnnnnnnnnnnnnnnnnnnnnnnnnnnnnnnnnnnnnnnnnnnnnnnnnnnnnnnnnnnnnnnnnnnnnnnnnnnnnnnnnnnnnnnnnnnnnnnnnnnnnnnnnnnnnnnnnnnnnnnnnnnnnnnnnnnnnnnnnnnnnnnnnnnnnnnnnnnnnnnnnnnnnnnnnnnnnnnnnnnnnnnnnnnnnnnnnnnnnnnnnnnnnnnnnnnnnnnnnnnnnnnnnnnnnnnnnnnnnnnnnnnnnnnnnnnnnnnnnnnnnnnnnnnnnnnnnnnnnnnnnnnnnnnnnnnnnnnnnnnnnnnnnnnnnnnnnnnnnnnnnnnnnnnnnngagcgtgagcgccgacacaagagcagagtgactcgaaggcatgccgccggtgcgccaaacagcaaaatcaaaaaagccgtgcaccgaccGTATAGCCGaccttcggcatgcgatagccactttaatcacctgacttgcaagccaactACAGGTGGCAGAGAGGAAGACTTCACCACTGAACACCGCATGCAGCTGGCCAAGAAAATAGCCACCGTCCATCGCGGGGCAGTCTCCTATACTCTCTAAACCTTTGTCAAGGGAGCGTTGTGTGCGCTATACTGGGGCGGCTGTACCCGCCATGGGCTGTGGTGTCGTACTGGGAAAGACGGGGAGGACGTTTGGGAGGAGCAACTGGTATCCGGCACGGGGACGCACCGTGTATGAAAGTAGGTGTTCGTCGCGTGAAAAGGTGGGTCTCGTCTAACGTTCCCCACACTGTGCATATGGATGTCGTCCCTCCTGTTTCCACCGTGAGACCCCATGGTGGAGCGGCTGTTGAGTGTTTTCGCTGCACCAAAAACGATGCAGGTAGACGAGTAGATCGTGTtttgcgaatattgttgcctgcnnnnnnnnnnnnTGGGGTGTATGCCGCGCTGCGCCGCACCGCTATCCGGCTCAATGGTCGTCCGGTGCAGCCTGCAAAGCGGGTGCAGGTGGGGGATGTGCTTTCtttacctgaatccctgtgtagggcgcgtgcagcatccagtcgtctttcgAAGATGCCTnnnnnnnnnnnnnnnnnnnnnnnnnnnnnnnnnnnnnnnnnnnnncaagatttgcttttttttcataaacccgcagggttgtgtgtgcacggtccgcgcagcctggatgcctgggtgaggggacaagggcgtgcgcacgttcnnnnnnnnnnnnnnnnnnnnnnnnnnnnnnnnnnnnnnnnnnnnnGAGGGACAGagggactgattgctttttcgcgctcgttacgcggtgcCCAGTGGTTTTCTGCCGCGTTGCAGCAGCATACATTGCGCAAGTTTTATCTTGCGATCACCGCCGCCCCTGCGCGCACCGCTGCTGCGGCGCGCACGCTGGTGCGtccaggagaggtaacgcatgtgcaaacagtgttacatagctgtgatnnnnnnnnnnnnnnnnnnnnnnnnnnnnnnnTTACGGGGAAAAAGCATCAGATTCGCCGTTATTGCGCTGCACAGGGATTCCCCCTGGTAGGAGATCGCACGTACGGTGGCGCGAGTCAGCACGGGAATGCnnnnnnnnnnnnnnnnnnnnnnnnnnnnnnnnnnnnnnnnnnnnnnnnnnnnnnnnnnnnnnnnnnnnnnnnnnnGGTGTnGACAACACCGAGCCCTGCATTCATGCGGGTGTTAACTTCTTTTCCGCCGGACTCCCTcgcgcgcgcgcgtgcgtgcgtggacgctctagacggngcgttaggtacagacgcaacctgcccgcaggatacggcagtgtgtacacctnnnnnnnnnccggtgtgagcgtgtactgtcgaagttccaaacagnnnnnnnnnnnnnngtgtagacctgtccccgtaggggcactgttactgttcgtaacactgttgctccttgctcttgcccttgcgtactttggtgctcaggtggaaccattgacaccnnnnnnnnnnnnnnnnnnnnnnnnnnnnngtcaggcgtcggggtacgtgnnnnnnnnnnnnnnnnnnnnnnnnnnnnnnnnnnnnnnnnnnnnnnnnnnnnnnnnnnnnnnnnnnnnnnnnnnnnnnnnnnnnnnnnnnnnnnnnnnnnnnnnnnnnnnnnnnnnnnnnnnnnnnnnnnnnnnnnnnnnnnnnnnnnnnnnnnnnnnnnnnnnnnnnnnnnnnnnnnnnnnnnnnnnnnnnnnnnnnnnnnnnnnnnnnnnnnnnnnnnnnnnnnnnnnnnnnnnnnnnnnnnnnnnnnnnnnnnnnnnnnnnnnnnnnnnnnnnnnnnnnnnnnnnnnnnnnnnnnnnnnnnnnnnnnnnnnnnnnnnnnnnnnnnnnnnnnnnnnnnnnnnnnnnnnnnnnnnnnnnnnnnnnnnnnnnnnnnnnnnnnnnnnnnnnnnnnnnnnnnnnnnnnnnnnnnnnnnnnnnnnnnnnnnnnnnnnnnnnnnnnnnnnnnnnnnnnnnnnnnnnnnnnnnnnnnnnnnnnnnnnnnnaagcgcacgcgcgttgcaggaaattaatggtgacttgtttcgtgctcctcttcgcagtcggtactatgtttcttcgcggtatggatggcgtagtgatccgtttaccggtgccagaagttnnnnnnnnnnnnnnnnnnnnnnnnnnnnnnnnnnnnnnnnnnnnnnnnnnnnnnnnnnnnnnnnnnnnnnnnnnnnnnnnnnnnnnnnnnnnnnnnnnnnnnnnnnnnnnnnnnnnnnnnnnnnnGGGTATCAGACCCTGTATGGGCATCTGCAGACGGTGTTGGTTTCAGCAGGTACGCGCGTCACCAGCGCGACAAAAATCGGATTATTGGGAAAAACAGGACGCAGCACGGGACCACACCTGCATTTTACAATCTATAAGAATGGCTCCgcgatannnnnnnnnnnnnnnnnnnnnnnnnnnnnnnnnnnnnnnnnnnnnnnnnnnnggnnnnnnnnnnnnnnnnnnnnnnnnnnnnnnnnnnnnnnnnnnnnnnnnnnnnnnnnnnnnnnnnnnnnnnnnnnnnnnnnnnnnnnnnnnnnnnnnnnnnnnnnnnnnnnnnnnnnnnnnnnnnnnnnnnnnnnnnnnnnnnnnnnnnnnnnnnnnnnnnnnnnnnnnnnnnnnnnnnnnnnnnnnnnnnnnnnnnnnnnnnnnnnnnnnnnnnnnnnnnnnnnnnnnnnnnnnnnnnnnnnnnnnnnnnnnnnnnnnnnnnnnnnnnnnnnnnnnnnnnnnnnnnnnnnnnnnnnnnnnnnnnnnnnnnnnnnnnnnnnnnnnnnnnnnnnnnnnnnnnnnnnnnnnnnnnnnnnnnnnnnnnnnnnnnnnnnnnnnnnnnnnnnnnnnnnnnnnnnnnnnnnnnnnnnnnnnnnnnnnnnnnnnnnnnnnnnnnnnnnnnnnnncattgtgctcgnnnnnnnnnnnnnnnnnnnnnnnnnnnnnnnnnnnnnnnnnnnnnnnnttatcctctgacggtgccgccacgttgtgtgtgcggtgccagtccagcgctttttctcgaaaaagggcgcagtcgtctacagtgcgcgcttcnnnnnnnnnnnnnnnnnnnnnnnnnnnnnnnnnnnnnnnnnnnnnnnnnnnnnnnnnnnnnnnnnnnnnnnnnnnnnnnnnnnnnnnnnnnnnnnnnnnnnnnnnnnnnnnnnnnnnnnnnnnnnnnnnnnncacccctcgctttgtttccggtctcccgggtcggtagcgtgtccctgcaggaaacacgagaatcacctttcccgcgttgcgcagttcctctaaataCTTCATAGCTGCACGATTCAGTGCTCGGCTGCGCTTTGTCTCTCTTGCAAGACAGtgcgcatcagtgatgGTGGCCAAACTCCTACTGGGATATATCACCAGGTGATCATATCCTTCGGTGAGCATTGCCAGGATGCGATTTTCTTCACGAAGTTTAATACCGGCAATAGATACGATTTTTTCTGAAAGCATGCGCCCGGCACTACTTCCTTGTTCCATAAGGTAGAGCAGACAGGGTAAGTCAAAATTGCTGTAGTGTTCCGCAAGAATGAGTCCCCGTTTCCCTTCATTGGTTTTATGTAAAAAGGATGCGATTTGCTCATTTCCGCGCATTGTGGAGCCGGGGAGTAATATGCGGGCGACGAGGGACTCAACGATacgccggataGGTTCGTTTCTCTCGTGTAACAAATTATCCTCCCGCATGAAGCGGTCTGCGCGCGTATACtgctccaggaGCGGGACGATGTCGCTAAAAGTCTGCTGCAACGAATGTATACTCACTGATTGTCTCCTTGGGCGAGGCGTGCAAGGTGTGCgggcaattcataaattgtttttacgcgtgtcatgnnnnnnnnnnnnnnnnnnnnnnnnnnnnnnncacgtnnnnnnnnnnnnnnnnnnnnnnnnnnnnnnnnnnnnnnnnnnnnnnnnnnnnnnnnnnnnnnnnnnnnnnnnnnnnnnnnnnnnnnnGTTTTGTGTACCGCTTCAAGCGTACGTACAAAGCAGCTGTGGTGTGGTTTTCCCTTGCCTGCATGATACGTGATGTCAAAGACnnnnnnnnnnaggtctgacagatgaaaaaaatccaatacgcgttgtgcgtgcacgcgcggtgcgttagttaatgccgtcagtggcatgtgcagtgactgtaaaaaagggcggagcatcnnnnnnnnnnnnnnnnnnnnnnnnnnnnnnnnnnnnnnnnnnnnnnnnnnnnnnnnnnnnnnnnnnnnnnnnnnnnnnnnnnnnnnnnnnnnnnnnnnnnnnnnnnnnnnnnnnnnnnnnnnnnnnnnnnnnnnnnnnnnnnnnnnnnnnnnnnnnnnnnnnnnnnnnnnnnnnnnnnnnnnnnnnnnnnnnnnnnnnnnnnnnnnnnnnnngggatttgtacaggagtacagcgtgttgtctatgtcaaataatatgtgtctaatcacgggtatgctnnnnnnnnnnnnnnnnnnnnnnnnnnnnnnnnnnnnnnnnnnnnnnnngtcaaagtagcgcgcgtaacgctgcgctgtgcgctggnnnnnnnnnnnactaaGTATAGAGTAGTGCGTATCAGAGTGGCGGATGAATATCTTCCATGTCTTTTTAACTGTTTTTGACGGAAGGTCAAACACTGCATAGGAAAGCGTAAGACGCGCGTGAGTGTCATTGATATGGAGTTGTTCAAGAAtgttttttcccnnnnnnnnnnnnnnnnnnnnnnnnnnnnnnnnnnnnnnnnnnnnnnnnnnnnnnnnnnnnnnnnnnnnnnnnnnnnnnnnnnnnnnnnnnnnnnnnnnnnnnnnnnnnnnnnnnnnnnnnnnnnnnnnnnnnnnnnnnnnnnnnnnnnnnnnnnnnnnnnnnnnnnnnnnnnnnnnnnnnnnnnnnnnnnnnnnnnnnnnnnnnnnnnnnnnnnnnnnnnnnnnnnnnnnnnnnnnnnnnnnnnnnnnnnnnnnnnnnnnnnnnnnnnnnnnnnnnnnnnnnnnnnnnnnnnnnnnnnnnnnnnnnnnnnnnnnnnnnnnnnnnnnnnnnnnnnnnnnnnnnnnnnnnnnnnnnnnnnnnnnnnnnnnnnnnnnnnnnnnnnnnnnnnnnnnnnnnnnnnnnnnnnnnnnnnnnnnnnnnnnnnnnnnnnnnnnnnnnnnnnnnnnnnnnnnnnnnnnnnnnnnnnnnnnnnnnnnnnnnnnnnnnnnnnnnnnnnnnnnnnnnnnnnnnnnnnnnnnnnnnnnnnnnGGTGAGGTGATGCGTTGTCCGGTCAGCTCAAAGCGGCCGTGCTTTTCAGTACCCGTGAAGAGAATTGTGATCCTGCGGCTTTCTGCCGGAGTACACAGCAGCGCACCGTACGTGTGTGCGTCGTAGCACAAAAAGCCAACCCATGTGGGCGnnnnnnnnnnnnnnnnnnnnnACAATACGTAATCTCCAGAGACTGGCGCCGGTTCTGTGGCCAAAAGACACCGCGCTCCCATGCTGAAAAGACACACGAGCGCTACCTTTATGTACACCGTTTTTCCTTTTCTCCTTCGTCATTTTTTTCTAAACCACGGGTGCCGTACACTGTGCTATTAnnnnnnnnnnnnnnnnntgcatgcgctggatcccgctccggcattcccgttcttacgaactttccggggaagcgagttgttcGAGCAGGCGTGCAATGTGCTCGGACGCCTCTTCTTGCGTAAAAAAATACGTGTGCGCACCACACCGTGTTGTGCATTCAACGTGCGGTAGCGTTTTCGCACTCAGTACCACACGAAGGGGAATACCGATTAAATCAGCGTCTGCGAACTTTACTCCGGGTCGCTCTGCACGATCATCAAACAGTACCTCAACTCCCCGTGTCCGCAGCTGCACGTACAGTGCCTCTGCTGCTGCATAGGGGGCCTGCGTGTGAGGGATGGGTATGAGCACAACTGCATAGGGGCTGATCGCAAGCGGCCAGCTGATACCCCGCGTGTCATGGTGGTTTTCCACCACAGAGGCAAGCGTGCGATCAAGGCCAATGCCATAGCAGCCCATCAGGGGGAACTGTCGTACACCCTGCTCATCAGTAAAGGTAAGGTGCATGCTGCGCGTGTATTTGTCCCCTAATTTAAAGAGGTGACCTAGTTCGTTACCCTTTTTTTCGTATAGGGGCGCTCCACAGTGTATGCACCGGTCGCCGGCGCGCACGAGCATGAGATCTGCAACTGCGTCAGGAACAAAGTCTCGACCCGGTTCTACCTGCACAAGATGCGTGTCAACTTTCAACGCACCGGTGATAGCGTTGTGCATAGCCATCACGGACTGATCTGCAACGAGGGAAAAAAATGGAATGTCAGAAGTGACGGATGCAGGCTCAGCAGCGGAGGGGAAGAACGCAGGGGTGCGAGCGGCATACGCAGCTGCAGCACGCTGTGCAAGTCCTACCGGACCAATGAATCCTACGGgggtgccgctgagtgcatacaCCTCTTGTTCAGTTGCCAGTACTACAGATGGCACGCGCAGGAGCGCTTCGAGCTTTGACTCGTTGAGTTCTAGGTCGCCGCGGATGCACACTGCCACaaaatgcccagccgcctggggcaccgtgtcaatgcggtaaataagcgtcttgatnnnnnnnnnnnnnnnnnnnnnnnnnnnnnnnnnnnnnnnnnnnnnnnnnnnnnnnnnnnnnnnnnnnnnnnnnnnnnnnnnnnnnnnnnnnnnnnnnnnnnnnnnnnnnnnnnnnnnnnnnnnnnnnnnnnnnnnnnnnnnnnnnnnnnnnnnnnnnnnnnnnnnnnnnnnnnnnnnnnnnnnnnnnnnnnnnnnnnnnnnnnnnnnnnnnnnnnnnnnnnnnnnnnnnnnnnnnnnnnnnnnnnnnnnnnnnnnnnnnnnnnnnnnnnnnnnnnnnnnnnnnnnnnnngcaatgactgataggccgatgcgacggaaaatggcgcgatacgcgtgcgcaaacttttcgtacgtgcgcgcaaggcatgcgcagtctgtgtggaaagaataggcgnnnnnnnnnnnnnnnnnnnnnnnnnnnnnnnnnnnnnnnnnnnnnnnnnnnnnnnnnnnnnnnnnnnnnnnnnnnnnnnnnnnnnnnnnnnnnnnnnnnnnnnnnnnnnnnnnnnnnnnnnnnnnnnnnnnnnnnnnnnnnnnnnnnnnnnnnnnnnngaaaaggagctcgtgatcgagccgatttttggcgcgcaaaagctctgcgcccatgcggtaccagcgnnnnnnnnnnnnnnnnnnnnnnnnnnnnnnnnnnnnnnnnnnnnnnnnnnnnnnnnnnnnnnnnnnnnnnnnnnnnnnnnnnnnnnnnnnnnnnnnnnnnnnnnnnnnnnnnnnnnnnnnnnnnnnnnnnnnnnnnnnnnnnnnnnnnnnnnnnnngacgtaccctgcgcgcatgagcaGCTGGTGGCTTGCGATGGTtgcatcagcaGGTGCAGACCGCAGGGTTGGTGCAAAAAACGCAGACATCTTCACGAAACGTACCCCTCCCGCGCGTAGTATATGTCTTTTTCAGACTCTTGTGTCTTACCCGGTCTGnGCGGAGCTGCCGACAGCTCTGACAAGGtgtccgcatctttgctaaaGAAGCGTTCCTTGTTCTGGGGAGTGCGGGGTGAGCGTGCGTGCCTCGGGGACAGGGAGACCTAGGTGCGCATAGGCACGCGCGGTGGCCATGCGCCCGCGGGGGGTGCGCTGCATGAGCCCAATTTGGATAAGGTAGGGCTCGTAGTAATCCTCAAGTGTTTCCGGTGATTCCCCGAGGGAGatcgccagcgtttctgcgcccactggccctccgccgaaGTGCTCAATCATGACGCGCAGCAGCTGTATGTCGTGCAGTTCTAGCCCTAATTCGTCGATCTTTAGGTGGGCAAGCCCTGCGCGTACTATCGTCTCGCTGATGTGTGCAGACCCCGCAACTTGGGCAAAATCGCGTATACGGCGCAAAAGCCGGTTGGCCACCCGGGGTGTTCCtcgcgaacacnnnnnnnnnnnnnnnnnnnnnnnnnnnnnnnnnnnnnnATCTAGAAGCCGCGCTGAGCGTTGCACAATGGCAGCAAGCTCCTCAGGGGTATAGAACTCGAAGCGCTCTACGATTCCAAAGCGGCTAATCAGCGGGCTTGAAACCATACCCGCGCGAGTGGTTGCACCAATGAGGGTAAACGGGGGGAGTGGGATGCGCACCGTGCGCGCGGACGGTCCCTGACCGATAACCCAATCCAGTTCGTAGTCCTCCATGGCAATGTACAGCATCTCTTCTATGGCTGGTTTGAGGCGGTGGATTTCATCCACGAAGAAGACGCTTCGCTCACTCAGCGCAGTGAGGATACCCGCTAAATCTTTTGGTTTATCAAGCGCCGGTGCGCCTGTAACCTTGCACTCAACGCCCAGCTCGCATGCAGTGATATGCGCGAGCGTCGTTTTGCCGAGCCCCGGGGGGCCGATGAGGAACAGGTGATCTAAGCTTTCGTTGCGATCGCGCGCTGCCTGAATGAAAAGACGTAAGTTGCGTTTTGTTTTCTCCTGACCTAGAAAGTCTTTCAGGAGGCGCGGTCTGAGCGCACGCTCGCGATCGTCTGTAGGTTGTGCTTCAgggcgcacnnnnnnnnnnnnnnnnnnnnnnnnnnnnnnnnnnnnnnnnnnnnnnnnnnnnnnnnnnnnnnnnnnnnnnnnnnnnnnnnnnnnnnnnnnnnnnnnnnnnnnnnnnnnnnnnnnnnnnnnnnnnnnnnnnnnnnnnnnnnnnnnnnnnnnnnnnnnnnnnnnnnnnnnnnnnnnnnnnnnnnnnnnncccanggagtntcaCTTGATACGTGAAAGAATATGTGCCTGCGTGCTCGCGCTTGGCATGCTGACCGGTTTTACGCACGCATTCGGTAGCAAGGATGCCGCAGCGGACGGGAAACCCCTggttgtcaCCACCATTGGCATGATAGCGGATGCTGTCAAAAACATCgctcaaggtgatgtgcatctaaaggggttgatgggtcctggtgttgacccgcaCCTGTACACGGCTACTGCGGGGGATGTGGAATGGCTCGGGAATGCGGATCTCATCCTGTAcaaCGGGTTgcacctggaaaccaagatgggcgaggtGTTTTCCAAACTGCGCGGGAGCCGCTTGGTAGTTGCAGTTTCTGAGACTATTCCGgngtctcagcgtctttctcttgaggaagcagagttcgatccgcatgtgtggtttgatGTAAAGctgtggncttattcggtgaaggcagtgtacgaannnnnnnnnnnnnnnnnnnnnnnnnnnnnnnnnnnnnnnnnnnnnnnnnnnnnnnnnnnnnnnnnnnagttggataagcttgacgcgtacgttcggcgcaaggcgcagtcgcnnnnngctgaaaggcgtgtgttggtgaccgctcatgatgcgTTCGGCTATTTTAGCCGTGCGTATGGTTTTGAGGTGAAGGGGTTGCAAGGGGTGAGCACCGCTTCGGAAGCCAGTGCGCATGATATGCAGGAACTGGCAGCGTTTATTGCGCAGCGTAAACTCCCTGCTATCTTTATTGAGAGTTCTATTCCGCACAAAAACGTTGAAGCGTTAAGGnatGCGGTGCAGGCAAGAGGGCACGTAGTGCAGATTGGAGGCGAGTTGTTTTCTGATGCGATGGGGGATGCGGGTACGAGCGAGGGTACCTACGTAGGGATGGTAACACACAATATCGATACGATCGTTGCTGCGTTGGCTCGCTAGCGTGGAATGTAGAGTTGAGTGGGTAGAAGAGCCGTTGGTGGTCGGCTGTACGGCAGCGCCTGTTCACGACGTGGTGATTAGTGAAGGAGGTGGCGCGCTCACCGCTGACGGCTCTTACCCGATTGAGCGAAAAGAAGGGAGCAGTATATGGCAGAGATATCAGCAACAGCTTATGCTGTCCAGGTTGACGACCTGACGCTTGCGTATCGGCAGAAGCCGGTGCTTTGGGACGTGGATGTGCGTATTCCAGAGGGGGTTATCGAGGCCATTATCGGTCCTAATGGGGCGGGCAAGTCGACCCTATTGAAGGCGATCATGGGTCTTCTGCCTCTCGCTTCCGGAGAGGTGCGTGTCTTTGGGCGTCCTTTTTCaaaggagcggcggcgtgttgcgtatgtccnnnnnnnnnnnnnnnnnnnnnnnnnnnnnnnnnnnnnnnnnnnnnnnnnnnnnnnnnnnnnnnnnnnnnnnnnnnnnnnnnnnnnnnnnnnnnnnnnnnnnnnnnnnnnnnnnnnnnnnnnnnnnnnnnnnnnnnnnnnnnnnnnnnnnnnnnnnnnnnnnnnnnnnnnnnnnnnnnnnnnnnnnnnnnnnnnnnnnnnnnnnnnnnnnnnnnnnnnnnnnnnnnnnnnnnnnnnnnnnnnnnnnnnnnnnnnnnnnnnnnnnngtgtggatgcagctactgaacaagcaatcgttantcttttaaaaacgctgaaagggcgtgggaaaacgttgcttgttgtgcatcATGATTTGCAGACGGTGGCAGAGTATTTTGACCGCGTGCTGCTTTTAAATGTTCGCGTCATCGCTGAAGGGGCCGTCGTGTCTGCCTTCACCGAAGAATACGTTCAAAGAGCCTATGGCGGACGGATTAGTTCCACCCTTTTTCCGAGAGGAAATAAGGAGGATGTGCACGATGCACGCGCTCATGCGTCTGTTCtctGACTATACgctgcaaaATGTGGTGTTAGGCACGCTTTTTTTGGGTTTGGGTTCTGGGCTGGTCGGCAGTTTTGCGGTGCTGCGTCGACAAAGCCTTTTCGGTGACGCAGTTTCTCATGCAACCCTTCCGGGGATTGTTATCGCGTTTCTTTTAACCGGCACGAAGTCtactgagatacntttgctgggtgctgcCCTCAGTGGTTTAGTAGGAACTGTGGTGATGCTAATGGTGATGCGTACTACAAAAATTGATACCGATGGTGCGCAGGGCATTGTGTtgggtgtttttcttgggtttGGGTTTCTATTACTCACCCACGTGCAGAAGTCGCCCCAGGCGGCAaaggctggtctgaacaaattnnnnnnagggcaagcggccacgATTTTGCAGCGAGATGTCCTGCTCATCATTGCGATGGAGGTGGTGATCGGTTTGCTTGTACTGCTGTTTTGGAAAGAACTGAAGCTTTCTACCTTCGATCGAGACTTCTCTGCGGTGCAAGGTTTTTCTCCACAGCTTATGGAGTTCATGCTCACGGCACTCATCGTAGTTGCAGTTGTCGTAGGGGTTCAGGCAGTGGGGGttatctnnnnnnnnnnnnnnnnnnnnnnnnnnnnnnnnnnnnnnnnnnnnnnnnnnnnnnnnnnnnnnnnnnnnnnnnnnnnnnnnnnnnnnnnnnnnnnnnnnnnnnnnnnnnnnnnnnnnnnnnnnnnnnnnnnnnnnnnnnnnnnnnnnnnnnnnnnnnnnnnnnnnnnnnnnnnnnnnnnnnnnnnnnnnnnnnCTATTATGCTTGGTCCTCAGCGGGGTGTTTTGTATCAACTGTGGCGGAGAAGACGGGTTTCGCTTCTTCAAGAGGAGGGGTAGAATATGACCATGGAGGTTGTGCTtattgcagtggtcgtgtcggttgcgtgcgcgctgtgtggggtnnnnnnnnnnnnnnnnnnnnnnnnnnnnnnnnnnnnnnnnnnnnnnnnnnnnnnnnnnnnnnnnnnnnnnnnnnnnnnnnnnnnnnnnnnnnnnnnnnnnnnnnnnnnnnnnnnnnnnnnnnnnnnnnnnnnnnnnnnnnnnnnnnnnnnnnnnnnnnnnnnnnnnnnnnnnnnnnnnnnnnnnnnnnnnnnnnnnnnnnnnnnnnnnnnnnnnnnnnnnnnnnnnnnnnnnnnnnnnnnnnnnnnnnnnnnnnnnnnnnnnnnnnnnnnnnnnnnnnnnnnnnnnnnnnnnnnnnnnnnnnnnnnnnnnnnnnnnnnnnnnnnnnnnnnnnnnnnnnnnnnnnnnnnnnnnnnnnnnnnnnnnnnnnnnnnnnnnnnnnnnnnnnnnnnnnnnnnnnnnnnnnnnnnnnnnnnnnnnnnnnnnnnnnnnnnnnnnnnnnnnnnnnnnnnnnnnnnnnnnnnnnnnnnnnnnnnnnnnnnnnnnnnnnnnnnnnnnnnnnnnnnnnnnnGTATTACCTGTGTGGGAGCCTTCGATTCGGTGGGTGCAGTGTTGGTCATTGCATTGATGATTACACCGCCTGCAGCAGCGCTTTTGTTGACAGATAACTTGTTGTTgatgttggtccttgcttcattgctcgcctcttgtgnnnnnnnnnnnnnnnnnnnnnnnnnnnnnnnnnnnnnnnnnngcattgcaggagcaatggctaccatggcgggcgttctgttcgcgttggtgtaccttttctctccannnnnnnnnnnnnnnnnnnnnnnnnnnnnnnnnnnnnnnnnnnnnnnnnnnnnnnnnnnnnnGACACTTGCCGTGCATCTTGCAACacaccgttacacggtggagcgcagcgtggagnnnnnnnnnnnnnnnnnnnnnnnnnnnnnnnnnnggtctgcgcgcagggcggcccgcgtggtgcgtaccgcgctcaggcgagggatggtagagcgtcacggtgccttgctgctactcactgcgcagggtgtgtcgctcgcgcaggcgcgattggatgtatccgtgtagnnnnnnnnnnnnnnnnnnnnnnnnnnnnnnnnnnnnnnnnnnnnnnnnnnnnnnnnnnnnnnnnnnnnnnnnnnnnnnnnnnnnnnnnnnnnnnnnnnnnnnnnnnnnnnnnnnnnnnnACACGTGACGCCGGGGACTATCAGCACAATGGTGAAGCGCTTGGAAAAGGGTGGCtatgtgcaacgcacgcaTCGTCTTGGCTGTACGTTAACCAGAAAGGGGGCAGTTTTTGGATCTGCAGTGTTAAGGAAGCATCGCTTGTTGGAGAGTTTTCTTTCCCAGGTATTGTGTTTAGAAGCAGGGGTGGTGCACAAAGAAGCGGAAAnnnnnnnnnnnnnnnnnnnnnnnnnnnnnnnnnnnnnnnnnnnnnnnnnnnnnnnnnnnnnnnnnnnnnnnnnnnnnnnnnnnnnnntCCCAAGAAAGGATACGCTTTTGGATTTGTATGTTGAGGACGATGTGCCAGGTGTATGATCTTTTTGTATGGGGTgaggatgcgccttttgtcaGATAAAAGGGGATGTGCAAAACGTATTGTTGAGAGGAGAGGGCCATGAAGCTTGTGTTGATCCGTCATGGAGAAAGTGAATGGAACAGGCTGAAcctgttcactggttggacagatgttccgcttaccccacgtggggagtcggaagcccannnnnnnnnnnnnnnnnnnnnnnnnnnnnnnnnnnnnnnnnnCCTATGCTACACttctttcttgaaacgtgccattcgtacgctcaATTTTGTACTCCAGGCACTGGACCGTGAGTGGTTGCCGGTTCACAAAAGCTGGAAATTGAACGAGCGGCATTATGGGGATCTACAAGGTTTAAATAAGACAGAGACGGCGCAGAAGTATGGTGAGCAGCAGGTTAGGGTGTGGCgtcgctcCTTTGATGTGGCTCCTCCTCCGCTTACTGTAGGGGACGCACGTTGTCCGCATACTCAAGCCTCCTACCGGGGGGTATGCGCGTCTGGTCGGACGCCAGTACTTCCGTTTACGGAAAGTTTGAAAGATACCGTTGCGCGTGTGGTGCCGTATTTTGAAGAGGAAATCAAACCGCAGATGATTTCCGGACAGCGTGTGCTTATTGTGGCGCATGGTAACTCGTTGCGCGCACTGATGAAGCACATAGAGTCTTTGGATGAGACTCAGATAATGGAAGTAAATTTGCCTACCGGTGtaccgcttgtctatgagTTCGAGGCGGATTTTACCCTGTGTGGGAAGCGTTTTTTAGGTAATGAGGCGGATGTTGCAGCGAGGGCGCAGGCtgtggctgatcagggtaagagtaactaactaaacctgcgtgtcgtatcctctggtcgtatgtatgattgtaaggctcnnnnnnnnnnnnnnncggagcgtcctgctttgtggcttgacttcgggctctagtcgtggtacgcnnnnnnnnnnnnnnnnnnnnnnnnnnnnnnnnnnnnnnnnnnnnnnnnnnnnnnnnnnnnnnnnnnnnnnnnnnnnnnnctgctatattccgttcatttatcnnnnngccttgtggtcggggctttttcctgaccttcaagggacagcttttttccgtgcctgggttgcgagtgcgcgttttcgtgtttttcacggggagggtgcgtgacggtcggcgtttttgctgcacnnnnnnnnnnnnnnnnnnnnnnncgtgannnnnnnnnnnnnnnnnnnnnnnnnngggcagggctcacgttctatgttgtgtcggtgggtgcncttcaggtggtgtatgtgtgcggcggtgttgggaaagtgaatgcagcgctttgcactcaactactcatttcggagtttggtgnnnnnnnnnnnnnnnnnnnnnnnnnnnnnnnnnnnnnnnnnnnnnnnnnnnnnnnnnnnnnnnnnnnnnnnnnnnnnnnnnnnnnnnnnnnnnnnnnnnnnnnnnnnnnnnnnnnnnnnnnnnnnnnnnnnnnnnnnnnnnnnnnnnnnnnnnnnnnnnnnnnnnnnnnnnnnnnnnnnnnnnnnnnnnnnnnnnnctttgatctctgcacacgggatccggagtggaccgagggtgcttgtgcgctctctggctcgggggatccgccttctcgcgTttctcgtttaGTCGAAGGGCGCGTCGCCTCAGGGGATCTTTTCGTGTCAGATGCGCAAACACGTGCACGCATTATACGCGAATTTGGTGCGCATGGTGTGGAAATGGAGGGGGCAGCCTTTGCGCATGTAGCGTCAGTTAATGGCGTCCCctttgtTATTATACGCTGTATATCCGACGGTGCGGGTGCTGAGCAGGATGTCTCTATGAGttataaagagttttcgacgcgtgcggcgcggcgttctgcccttttgacgttgcGTGTTCTTGAGCGTCTCAGTGCTTTACGCACATCGGTAGTGGCTTCTCTTTTCCCGATGGTCGTGGTGTAATGGTCGGGAGTAAGACAGTGGGCTGAGCAGTCGGGCGATGCTGTGGGGnnnnnnnnnnGGTGTATACCCAGTTGTTATACTGCGGCACTCGGAGGTTTTATGGTGAAAAGAGGTGGCGCGTTCGCGCTGTGTCTTGCGGTGTTGCTTGGGGcgtgttcatttagttctatcccgaatggcacgtaccgggcgacgtatcaggattttgatgagaatggttgnnnnnnnnnnnnnnnnnnnnnnnnnnnnnnnnnnnnnnnnnnnnnnnnnnnnnnnnnnnnnnnnnnnnnnnnnnnnnnnnnnnnnnnnnnnnnnnnnnnnnnnnnnnnnnnnnnnnnnnnnnnnnnnnnnnnnnnnnnnnnnnnnnnnnnnnnnnnnnnnnnnnnnnnnnnnnnnnnnnnnnnnnnnnnnnnnnnnnnnnnnnnnnnnnnnnnnnnnnnnnnnnnnnnnnnnnnnnnnnnnnnnnnnnnnnnnnnnnnnnnnnnnnnnnnnnnnnnnnnnnnnnnnnnnnnnnnnnnnnnnaggtagaccggtccgggacaTCCCTATAAGCTGAAGACCGATACGGGCGCCCACACTGGTGGGGGGCCTCGAAAGCGATGATGGGGtgtgcggggtcttctcgcgccctCTTCACCGACGCGTGCGCCTCCTCAATTAAGTGGGCCGCGGTAGTTAGTCTGGACGTACGCGTCGCGATGCCGATAAGGACGCGTGCGCTCTCAATGGCGCCGCACagctccttgtgtatatccctcgcgagtACCATCGCCTCTTCGAGGCTTGCGTCTTGTACGATACCCGCGAAGCAGCAACTACCCTCGAATTCAAAGAGCATATCCCGAAATTTGAAGAACTCCACGAGCTTTTTCGCTGCGTCCGCAACnnnnnnnnnnnnnnnnnnnnnnnccacctgtactATCATTAGCGTTAGGTCTTGTTCGGAAGCGGTGGCGCGTCCTAATTCTGCCTCAAGCCTTTCCtccagataggcccgccaaccgaccccggttaagggattgaaaagaccataggtgctctttttttcatgaacgggaagcagcgtctgccnnnnnnnnnnnnnnnnnnnnnnnnnnnnnnnnnnnnnnnnnnnnnnnnnnngttcctggaacaccgagtcatgtatggaaaaaagctcctcgtcagaatggttgtacaaatgtaaaccttcgaggtcgctcacgctctccttagaagcaaggtcnnnnnnnnnnnnnnnnnnnnnnnnnnnnnnnnnnnnnnnnnnnnnnnnnnnnnnnnnnnnnnnnnnnnnnnnnnnnnnnnnnnnnnnnnnnnnnnnnnnnnnnnnnnnnnnnnnnnnnnnnnnnnnnnnnnnnnnnnnnnnnnnnnnnnnnnnnnnnnnnnnnnnnnnnnnnnnnnnnnnnnnnnnnnnnnnnnnnnnnnnnnnnnnnnnnnnnnnnnnnnnnnnnnnnnnnnnnnnnnnnnnnnnnnnnnnnnnnnnnnnnnnnnnnnnnnnnnnnnnnnnnnnnnnnnnnnnnnnnnnnnnnnnnnnnnnnnnnnnnnnnnnnnnnnnnnnnnnnnnnnnnnnnnnnnnnnnnnnnnnnnnnnnnnnnnnnnnnnnnnnnnnnnnnnnnnnnnnnnnnnnnnnnnnnnnnnnnnnnnnnnnnnnATTCAGCGCCATGATAAGGTCATCATTTTTACACAAATTCTGGATAAAATCCGCCTCTGCATCTGTCAACTGCCTTTTGGATGTCACGCGCGGTGCAATCCGCCTGATCACttgtgcaaagcgctgctgnnnnnnnnnnntatttctttgaatcgcttgtgaaatagagaaggcgaaccaagccaccgttaagaccatgatcgcgatagcaaaaacggcataggcgcgcnnnnnnnnnnnnnnnnnnnnnnnnnnnnnnnnnnnnnnnnnnnnnnnnnnnnnnnnnnnnnnnnnnnnnnnnnnnnnnnnnnnnnnnnnnnnnnnnnnnnnnnnnnnnnnnnnnnnnnnnnnnnnnnnnnnnnnnnnnnnnnnnnnnnnnnnnnnnnnnnnnnnnnnnnnnnnnnnnnnnnnnnnnnnnnnnnnnnnnnnnnnnnnnnnnnnnnnnnnnnnnnnnnnnnnnnnnnnnnnnnnnnnnnnnnnnnnnnnnnnnnnnnnnnnnnnnnnnnnnnnnnnnnnnnnnnnnnnnnnnnnnnnnnnnnnnnnnnnnnnnnnnnnnnnnnnnnnnnnnnnnnnnnnnnnnnnnnnnnnnnnnnnnnnnnnnnnnnnnnnnnnnnnnnnnnnnnnnnnnnnnnnnnnnnnnnnnnnnnnnnnnnnnnnnnnnnnnnnnnnnnnnnnnnnnnnnnnnnnnnnnnnnnnnnnnnnnnnnnnnnnnnnnnnnnnnnnnnnnnnnnnnnnnnnnnnnnnnnnnnnnnnnnnnnnnnnnnnnnnnnnnnnnnnnnnnnnnnnnnnnnnnnnnnnnnnnnnnnnnnnnnnnnnnnnnnnnnnnnnnnnnnnnnnnnnnnnnnnnnnnnnnnnnnnnnnnnnnnnnnnnnnnnnnnnnnnnnnnnnnnnnnnnnnnnnnnnnnnnnnnnnnnnnnnnnnnnnnnnnnnnnnnnnnnnnnnnnnnnnnnnnnnnnnnnnnnnnnnnnnnnnnnnnnnnnnnnnnnnnnnnnnnnnnnnnnnnnnnnnnnnnnnnnnnnnnnnnnnnnnncctatgtgaacgtattttttcaatacctttttcaagacttggccctgcacctaatatgatcgcctcagtttgtgtattgatgttcggtagctttggtgtgaatatctgtgcaTATCTTAGATTGAGTAATGCGTTTCTCATCCAAAGCtttccaaagtgcacttggactgaaaagtcggcacgaatgattttcattgcttggctggtaagctcagcgatcttttgttcgcttgtgggaaaaaatgccttccagggttgtagatnnnnnnnnnnnnnnnnnnnnnnnnnnnnnnnnnnnnnnnnnnnnnnnnnnnnnnnnnnnnnnnnnnnnnnnnnnnnnnnnnnnnnnnnnnnnnnnnnnnnnnnnnnnnnnnnnnnnnnnnnnnnnnnnnnctccaggagtgcacggaaactcgcgtaatcaaattcACATACCGCGCAGCATGCGTGnnnnnnnnnnnnnnnnnnnnnnnnnnnnnnnnnnnnnnnnnnnnnnnnnnnnnnnnnnnnnnnnnnnnnnnnnnnnnnnnnnnnnnnnnnnnnnnnnnnnCATGGGTTGTATCTTGAGTGGAGTGTCCGATTGTTCCTAAAGAAGGGAATGTTTTCTCCTGTGTGTGCAGTCAATGTGCCGGCGTATATTCCgttagaatgggtgcgctccagtgtcaaggcgatatcctgatagcgcattctgagcgctcggatatTTTTTTTAAACACATTAGTCATATGCTGAGCTTGTTCCTAAGTTGAACGATTTCATGGCGCACCTTGTGAATGAGAGtntgtgttccctgagaagggttttttctcaggtgaagtagctcagcgtatgcaagaaactcaaaagcctcttttnnnnnnnnnnnnnnnnnnnnnnnnnnnnnnnnnnnnnnnnnnnnnnnnnnnnnTGTGATGCGttcaaagtagtggcggagcactgcctttttgtggannnnnnnnnnnnnnnnnnnnnnnnnnnnnnnnnnnnnnnnnnnnnnnnnnnnnnnnngttgggagaaatcttcatacgtgatggtacgtacattggaaggtggtgcgtgttctgtatttagccgtgtaagtttctggattttctcttgtgggagactgtaaaaccagtgtgcgtatgtttctagtgnnnnnnnnnnnnnnnnnnnnnnnnnnnnnnnnnnnnnnnnnnnnnnnnnnnnnnnnnnnnnnnnnnnnnnnnnnnnnnnnnnnnnnnnnnnnnnnnnnnnnnnnnnnnnnnnnnnnnnnnnnnnnnnnnnnnnnnnnnnnnnnnnnnnnnnnnnnnnnnnnnnnnnnnnnnnnnnnnnnnnnnnnnnnnnnnnnnnnnnnnnnnnnnnnnnnnnnnnnnnnnnnnnnnnnnnnnnnnnnnnnnnnnnnnnnnnnnnnnnnnnnnnnnnnnnnnnnnnnnnnnnnnnnnnnnnnnnnnnnnnnnnnnnnnnnnnnnnnnnnnnnnnnnnnnnnnnnnnnnnnnnnnnnnnnnnnnnnnnnnnnnnnnnnnnnnnnnnnnnnnnnnnnnnnnnnnnnnnnnnnnnnnnnnnnnnnnnnnnnnnnnnnnnnnnnnnnnnnnnnnnnnnnnnnnnnnnnnnnnnnnnnnnnnnnnnnnnnnnnnnnnnnnnnnnnnnnnnnnnnnnnnnnnnnnnnnnnnnnnnnnnnnnnnnnnnnnnnnnnnnnnnnnnnnnnnnnnnnnnnnnnnnnnnnnnnnnnnnnnnnnnnnnnnnnnnnnnnnnnnnnnnnnnnnnnnnnnnnnnnnnnnnnnnnnnnnnnnnnnnnnnnnnnnnnnnnnnnnnnnnnnnnnnnnnnnnnnatattcggtttgctggCTCCCAGGGAACAAACGCGGTTTGCCCGAGCAATTCGTCAGGGAtatgattaattaagaaagaatgcaaactgccgcagtctggtctccagacnnnnnnnnnnnnnnnnnnnnnnnnnnnnnnnnnnnnnnnnnnnnnnnnnnnngcgataagctttgcatgtggaaagcgtgcccgcaaaaactccgcgGTGTACGACtcacccggctctgttattaccacaatgcggcgaccttctaggatcgcagcgacaaaacgctccgcctcccttcttgggttatattttgagtggaggttcagcgaCACGTCTCCCCGGTTTCGTACTGCAAGAAACGGAGGACGTCCGTCTGCGCGCGAGTAGTTCCACTGCTTGTGATGAGAAGTGAATTCTCCGCGTGGTTAACAAAGTACGCGCTGACGCCACGGGTAAGTTGGTTTAGGTACAGCTCAGGATCGACGGGAAAGGAAGAAAAGACCACAACGCGCAGCTCCCGGGAAGCGACGCATGAggcaaggttgTCGTTCCCTACGAGCGTTAATAGCTGGTnnnnnnnnnnnnnnnnnnnnnnnnnnnnnnnnnnnnnnnnnnnnnnnnnnnnnnnnnnnnnnnnnnnnnnnnnnnnnnnnnnnnnnnnnnnnnnnnnnnnnnnnnnnnnnnnnngggcgaACTcgagctgggagcagctcggtgccaactccggCTGTCGCGGATACACGGGCCTACTCGCCTCGATAAACGAGAAGCGCGCCTGTACGGCAAGGATAAAGGAACGCACGCGCTCCTCGCCTGATGCATCACCGTGAAGGTCTGCAGCGAGggcaccttcctcgacgtcggttcnggtatcaaagnnnnnnnnnnnnnnnnnnnnnnnnnnnnnnnnnnnnnnnnnnnnnnnnnnnnnnnnnnnnnnnnnnnnnnnnnnnnnnnnnnnnnnnnnnnnnnnnnnnnnnnnnnnntgcctgaacagggtcgtccactgctccgcaggcaggccgggcgaaaggagggaggtgggaacacgcatacggtaaaacgtggtggtgtcaatcccgaaggggaaacgcaggtcaaacatgcgctcttcagtgannnnnnnnnnnnnnnnnnnnnnnnnnnnnnnnnnnnnnnnnnnnnnnnnnnnnnnnnnnnnnnnnnnnnnnnnnnnnnnnnnnnnnnnnnnnnnnnnnnnnnnnnnnnnnnnnnnnncgataagacccattaggaaatccctagctcgtcaaagaggtgtttgtacgtgtggaaatactccgatcnnnnnnnnnnnnnnnnnnnnnnnnnnnnnnnnnnnnnnnnnnnnnnnnnnnnnnnnnnnnnnnnnnnnnnnnnnnnnnnnnnnnnnnnnnnnnnnnnnnnnnnnnnnnnnnnnnnnnnnnnnnnnngcgcaggacgggccgcttccgattggggcggggactcgattgccaccggcgtggcaacctccgaaGACTCAGAGGCGGCGGTTTCAATCGCGTGGGGAGCAGAAAACTCTAGCGAGGGAACGGAGACGTCCAACTCCTGCTCTTCTGGCagtgggaacgatgtatcttcgcgctgggtgtcttcgtcgtcgaagacattttcagtattgaggggctgctcggcaccggctgcatcgaggccggtggaggaaacgtgnnnnnnnnnnnnnnnnnnnnnnnnnnnnnnnnnnnnnnnnnnnnnnnnnnnnnnnnnnnnnnnnnnnnnnnnnnnnnnnnnnnnnnnnnnnncatcgaaaacttcgtttccaagagtagcacgcccctgttgtacccactgctcctgcgcgacgcgcgctgcagcgtggtcgtcctcctctAGGTAGCTGAAATCGCCTGCAGCGGACCCGATGTGAGAGGACTCAAAACCACTGAGTTCACCACCAAATGCGTCGGCCGCAGAGTCTTTCCCATCTTCCTCGGTAAACTCagaggtgatgaggatattgttcagctcatcgttggtaagcgcaatcgtctcgtctggatcatcgtcacagaaaaacccgGAGTAGGCAGCCTctgttccttccggagccggaGCGGTGGCGGGAGCCtgagcaggctcggcgccagcttgccgggagaaggttcccttcagctggtctagatcagcgcggatactgctgatttcctgtgcaattttcagaagcaaatcggnnnnnnnnnnnnnnnnnnnnnnnnnnnnnnnnnnnnnnnnnnnnnnnnnnnnnnnnnnnnnnnnnnnnnnnnnnnnnnnnnnnnnnnnnnnnnnnnnnnnnnnnnnnnnnnnnnnnnnnnnnnnnnnnnnnnnnnnnnnnnnnnnnnnnnnnnnnnnnnnnnnnnnnnnnnnnnnnnnnnnnnnnnnnnnnnnnnnnnnnnnnnnnnnnnnnnnnnnnnnnnnnnnnnnnnnnnnnnnnnnnnnnnnnnnnnnnnnnnnnnnnnnnnnnnnnnnnnnnnnnnnnnnnnnnnnnnnnnnnnnnnnnnnnnnnnnnnnnnnnnnnnnnnnnnnnnnnnnnnnnnnnaccaaatcgtcgaacgacggcacctccgagtaagcggcatctccggctGGCGCATCTGCCGCCTGGTCGGAGGAGGACTCGCCACCCGGAGCGTGGCTGCACCCGCGAGCGAGCACCTCGTCCTCcacggagaggtcancaaagccacgagaatcctctncagangactgcacaggaagcgcacgntacacatngtgccnnnnnnnnnnnnnnnnnnnnnnnnnnnnnnnnnnnnnnnnnnnnnnnnnnnnnnnnnnnnnnnnnnnnnnnnnnnnnnnnnnnnnnnnnnnnnnnnnnnnnnnnnnnnnnnnnnnnnnnnnnnnnnnnnnnnnnnnnnnnnnnnnnnnnnnnnnnnnnnnnnnnnnnnnnnnnnnnnnnnnnnnnnnnnnnnnnnnnnnnnnnnnnnnnnnnnnnnnnnnnnnnnnnnnnnnnnnnnnnnnnnnnnnnnnnnnnnnnnnnnnnnnnnnnnnnnnnnnnnnnnnnnnnnnnnnnnnnnnnnnnnnnnnnnnnnnnnnnnnnnnnnnnnnnnnnnnnnnnnnnnnnnnnnnnnnnnnnnnnnnnnnnnnnnnnnnnnnnnnnnnnnnnnnnnnnnnnnnnnnnnnnnnnnnnnnnnnnnnnnnnnnnnnnnnnnnnnnnnnnnnnnnnnnnnnnnnnnnnnnnnnnnnnnnnnnnnnnnnnnnnnnnnnnnnnnnnnnnnnnnnnnnnnnnnnnnnnnnnnnnnnnnnnnnnnnnnnnnnnnnnnnnnnnnnnnnnnnnnnnnnnnnnnnnnnnnnnnnnnnnnnnnnnnnnnnnnnnnnnnnnnnnnnnnnnnnnnnnnnnnnnnnnnnnnnnnnnnnnnnnnnnnnnnnnnnnnnnnnnnnnnnnnnnnnnnnnnnnnnnnnnnnnntcgGTGCGTATGCGCGTCAGCTAGGATATGTCCGCGCGGGGGATGTGTTAGTGAGGCCGGTAAACTTTACCGTTGCGCACATGCATACCCTTGATTCTGGGGATGCACGTCCGCTTGTTGCACCTGCATGTTTTAGCGACACGCGTTGCAAGGTGTACGCGCTGTGCGTCGGCTTCTTTGTCGTCCTGTTACAGCtgctGTGGGGTAGCGCGCGTGCGTATTTTAAAACATGAGGCGCAGTCTGCGCACGCTGCTGCGCGTGCGCTCAAGGCCGGTGCGCTCGTAGCGTTGCCGACAGATACGGTGTACGGTTTCTCTGGCCTTGTGCCACACGCTGTTCCGGATCTCATATGTCTGAAGGCGCGTGGGTGCACAGAGACGGAAGGGAACCGGAGAGAGGGCTATCCGTTCATTGCACTGCTTGCAGATCCACAGGACGTGGTTGTCTATACCGGGACGCGGCTTCCnnnnnnnnnnnnnnnnnnnnnnnnnnnnnnnnnnnnnnnnnnnnnnnnnnnnnnAGACGGCGCGACGCAGGCGTTCCGCTGTCCTGCTGACCTGTGGCTGCGCtcagtgatacgggcagttgggggagcgatcttttccacgagtgcaaatcnnnnnnnnnnnnnnnnnnnnnnnnnnnnnnnnnnnnnnnnnnnnnnnnnnnnnnnnnnnnnnnnnnnnnnnnnnnnnnnnnnnnnnnnnnnnnnnnnnnnnnnnnnnnnnnnnnnnnnnnnnnnnnnnnnnnnnnnnnnnnnnnnnnnnnnnnnnnnnnnnnnnnnnnnnnnnnnnnnnnnnnnnnnnnnnnnnnnnnnnnnnnnnnnnnnnnnnnnnnnnnnnnnnnnnnnnnnnnnnnnnnnnnnnnnnnnnnnnnnnnnnnnnnnnnnnnnnnnnnnnnnnnnnnnnnnnnnnnnnnGCACGACAGGGGCGGTTCTGACGCCtgaaagccggttgtttcctgagatctttgggaacctccactggagcggacgcgcctcctgcgcgagcttctgagcgatctcagggnnnnnnnnnnnnnnnnnnnnnnnnnnnnnnnnnnnnnnnnnnnnnnnnnnnnnnnnnnnnnnnnnnnnnnnnnnnnnnnnnnnnnnnnnnnnnnnnnnnnnnnnnnnnnnnnnnnnnnnnnnnnnnnnnnnnnnnnnnnnnnnnnnnnnnnnnnnnnnnnnnnnnnnnnnnnnnnnnnnnnnnnnnnnnnnnnnnnnnnnnnnnnnnnnnnnnnnnnnnnnnnnnnnnnnnnnnnnnnnnnnnnnnnnnnnnnnnnnnnnnnnnnnnnngtcaaacacatctCGGACCAGGTGGCGCGAGTCAAGCAGAATGCGCGCTGCCGTCTnnnnnnnnnnnnnnnnnnnnnnnnnnnnnnnnnnatctttacccttgagtgcaaCAGCCAGTCTAATCCCCTCAGGGGTGAGGTCCATCGCGCCGCAAAATATGTAGTCAAGATTCCCTTTTTCGGGAAAACGGTGCGGCACCGCTTGCTCTCTGCAATCTACAACGTGATACCCACGCAACTCCCGAATGAAAGAAAAGAGCGCGTCGTTGATGGTAGTTTCTGTGTGCGCAGGCACACCAGACACTTCTAGCCTGTAGACGCCAACACGAGGAGCTGCGTGAACAGCATGCGCGAACAGCAcgaacaGGATAAGCAGCCGAGAAGAACGGACACCTTTTTTCATGAGACTAGTGGTGTCGCTCACAGAGGCTGCGGGACAGCTCCCGTGCGTTGTCGCGAGCTTTGATCTGCGCGCGCTTGTCAAAAAGCTTCTTGCCCTTGCAGATTCCCAGCGCTACCTTCACCCGCCCTGCTTTTAGGTAAAACTCCAGGGGGACCAGAGTATAGCCTTTCTCTTCAACCTTGCGCTTCAAGCGCGCAATCTGGTCCCGATGTGCCAGTAACTTCCGCATCCGATCCGGATTGGGGGCAAAGGAGCAAGCATGCACGTACTCCGCAATATGCACATTCTTTAGCCACAGCTCGCCTCCGCGCATCTCTGCAAATGCGTCAGGAAAAGAAAGATGCCCCGCGCGCACAGACTTCACCTCCGTGCCTTCAAGngcgatgccacactctagacggtcttccacatggtaattgaaaaaaGCCTTGCGGTTCTTTGCAATGAGATGGGTTCCTGTGCCCCTCATGGCGCCGGATGCTACcggataggcacttcccttgtcaattcgattatcgccGTGTTAGGCTGCCGTGTCTGGGAGGGACGCCGTTTTATGTTTGCGCGGTGGAGAAGGTACTCATATTTGGCGCGGCGCGAAGCACGGCGGAATGCGACCGCAGTTTGTAGTGCtggggtgggcttctttctgttctatctttttatcactacgcaTGTGGTTGCAGcgtatcgcattcaggcggactcgatgcagcnnnnnnnnnnnnnnnnnnnnnnnnnnnnnnnnnnnnncctgtttcgctttgcccgcatcaagcggggggatttggtgcttgcaactccccttgagaaagaggatataggcnnnnnnnnnnnnnnnnnnnnnnnnnnnnnnnnnnnnnnnnnnnnnnnnnnnnnnnnnnnnnnnnnnnnnnnnnnnnnnnnnnnnnnnnnnnnnnnnnnnnnnnnnnnnnnnnnnnnnnnnnnnnnnnnnnnnnnnnnnnnnnnnnnnnnnnnnnnnnnnnnnnnnnnnnnnnnnnnnnnnnnnnnnnnnnnnnnnnnnnnnnnnnnnnntagctacgacgtgcgtaagggggtgcttcctgagcatTGGTCTGAACGGCTTCCCTTTTCTGGTTTCATGGAAGAGATGCAGTTGGACGAGCACTCCTACTTCGTGCTGTGCGATAATCgaattgtctccagtgattctcgtctgtggggtnnnnnnnnnnnnnnnnnnnnnnnnnnnnnnnnnnnnnnnnnnnnnnnnnnnnnnnnnngagcatttggtgtCTTGTAGTGTGTAGGCGCCGCATTTGTGGTGCGTGCTGCGCATCGTGCTGTTCCTTTTATCATGTCTTCTGAGGTCGGTGCGTCTTTGTACGTGCACATCCCCTTCTGTGCGCAACGCTGTGCTTACTGCGATTTTTACTCCCTGGTGCGTTCAACCTATTTTAGGCCTCATCAGCCTTGTCCGCATTTTATCGATCGGCTGCTACAGGATGTGGCATTGCAGCGGGAGTGCTTTGGGGTCCAGGGGTGGCAGACAGTGTATATGggtggAGGTACCCCTTCGCTATTGGCACCGCAGGACATTCGTCATTTTTGCGTAGCGTTACGCGCCGCGCAGCGGTATCCGATTCAGGAGTTCACTCTTGAGGTGAATCCTGAGGATGTGACCGAAGAGTTTTTGTGTGCGTGTGCAGAAGGCGGAGTAAACCGTTTATCCCTTGGGGTACAAAGtctgcgtgatgaggtgttgcgtgcggnnnnnngtgcagcctctgctgaatgtgctcgtaccgcgctccgcgtgatgacggcaaatgcgcgCTTTTTCTCTGGCGGGGTGCGTATTTCAGCAGATCTCATCGCTGGATTGCGCGGGCAAACGGCGCGAATGGTGCGTGAGGATATAGATGAGCTTTTGTCTTTTGGGCTGAGACACGTGTCGCTATATGGGTTGTGTGTACCGCATCCGACTGAAACGCAAGAGGAGCGAATTGCAGCGCTTTGGGCACACGGCAGCGCGTATCTGGTGCGTGCAGGATTTAACCGGTATGAGCTTTCGAATTTTGCACGTACTGCGGCGGACGAGAGCGCGCACAACAGAGCATATTGGCGGATGGCACCGCACGCAGGGGTGGGGCCTGGCGCAGTTGGCACGCGTTTTGTCAACCTTTCTTTATCAAAGGAGGGGGCGTGGGCGATCCGCAGCACGGTGCGGAAACATCTTGGCCAATACTTAGCAGAAGTGTGTCGGGGAAATGTGTATGAGCACGAATTCCTTACAGAACATATGnnnnnGCAAGAAGCATTGTTAATGGGATTACGTCTTGAACAGGGACTGGATGTGGTTACATTTCGTGCGCGGTTCGGGAAGGGAATTGAAGcgtacattggcaaaacaatcgcgcggtggcagtgtcatggccnnnnnnnnnnnnnnnnnnnnnnnnnnnnnnngagtgcgcaggCACGGGTATTTCTGGACAGTTTTTTGCGAGAGGCGTTTGCAGAACTTGCGCGCACGTGAGTGGTCGTGAGAATGGGTACGCCGTGTACTATTTgaattcgggtatatttcctttggaagagcttgncagcctgcgtttgtnnnnnnnnnnnnnnnnnnnnnnnnnnnnnnnnnnnnnnnnnnnnnnnnnnnnnnnnnnnnnnnnnnnnnnnnnnnnnnnnnnnnnnnnnnnnnnnnnnnnnnnnnnnnnnnnnnnnnnnnnnnnnnnnnnnnnnnnnnnnnnnnnnnnnnnnnnngtgcctgagaggcccgtttggttgtagcgctgtcGATCCTTCATTTCGCTTTTTTGTGCCTCCTTGTTGCGTGCGGGCGTGCGGGGTCCGCCGTATCTGTGCCACAGATCTTTTGAGGAGGATTTTCATGGCCAAGGAAAAGTTCGCGCGCACTAAAGTTCACATGAACGTGGGTACTATTGGGCACGTCGATCACGGGAAGACAACGCTCTCTGCGGCGATCACCTCGTACTGTGCAAAGAAGTTCGGTGATAAGCAACTAAAATACGACGAGATTGACAATGCGCCCGAAGAGAAAGCGCGCGGGATCACCATTAACACGCGTCATCTTGAGTATCAGTCCGATCGTCGTCATTACGCGCATATTGATTGTCCTGGGCACGCGGACTATGTGAAGAATATGATCACGGGTGCTGCGCAGATGGACGGTGGTATTCTCGTCGTGTCTGCGCCTGACGGCGTTATGCCACAGACGAAGGAGCATCTTCTGCTCGCCCGTCAGGTTGGTGTTCCCTCCATCATTGTTTTTTTGAACAAGGTTGATTTGGTTGATGATCCTGAGTTGCTAGAGCTGGTGGAAGAAGAGGTGCGTGAtgcgcttgctggatatgggttttcgcgtgagacgcctAtCGTCAAGGGGTCTGCGTTTAAAGCTCTGCAGGATGGCGCTTCCCCGGAGGATGCAGCTTGTATTGAGGAACTGCTTGCGGCCATGGATTCCTACTTTGAAGACCCAGTGCGTGACGACGCAAGACCTTTCTTGCTCTCTATCGAGGATGTGTACACTATTTCTGGGCGTGGTACCGTTGTCACGGGGCGCATCGAATGTGGGGTAATTAGTCTGAATGAAGAGGTCGAGATCGTCGGGATTAAGCCCACTAAGAAAACagTGGTTACTGGCATTGAGATGTTTAATAAGTTGCTTGAtcagggaATTGCAGGTGATAACGTGGGGCTGCTTTTGCGCGGGGTGGATAAAAAAGAGGTTGAGCGCGGTCAGGTGCTTTCTAAGCCCGGTTCTATTAAGCCACACACCAAGTTnnnnnnnnnnnnnnnnnnnnnnnnnnnnnnnnnnnnnnnnnnnnnnnnnnnnnnnnnnnnnnnnnnnnnnnnnnnnnnnnnnnnnnnnnnnnnnnnnnnnnnnnnnnnnnnnnnnnnnnnnnnnnnnnnnnncatggtgaagccggggGATAACACCAAGATTATAGGTGAGCTCATCCACCCGATAGCTATGGACAAGGGTCTGAAGCTTGCGATTCGTGAAGGGGGGCGCACTATTGCTTCTGGTCAGGTGACAGAGATTTTGTTGTAGGCGTTTGCGGCGCGGAGTGTGTTTGGAGTTATTTTGCAAGGTGGGTGCGGTTTTAGGCTGATGGAGGGGnTATGGCCAGGGAGAGAATTCGGGTAAAACTGTGCGGATTTGACGTGGAGCTAGTGGATCAAAGTTCGCGCGCGATCGTGCACGCGGTGCAGAAGGCGGGCGCTGAGGTGCTCGGACCTATTCCGCTTCCGACTAGGATGCACAAGTTTACGGTCTTGCGCTCCCCTCATGTGAACAAGAAGTCGAGGGAACAGTTTGAGATGCGTACGCACAAGCnnnnnnnnnnnnnnnnnnnnncttctcaggnnnnnnnnnnnnnnnnnnnnnnnnnnnnnnnnnnnnnnnnnnnnnnnnnnnnnnnnaagcagtgaggcgtgtgtgttttgtctgtgcgttgcgatacggaagAGGTAGGTGATGGTTGGTTTAATCGGCCAGAAAGTTGGTAtgacccagatttttgacgcacggggttgtgttacgccggtgacggtgattcgggnggagcacaacGTGGTGGTAGGACTGAAGGATGTGGAGCGCTTCGGTTACTCTGCAGtgatacttggcacagggtgcatgaagaaaagtcgtatctcaaagccatatgctggacagttcgctgagcgGATACCGCCGGTGAGGGTCATGAGGGagtttcggggctttacgttggacgtttcggtnnnnnnnnnnnnnnnnnnnnnnnnnnnnnnnnnnnnnnnnnnnnnnnnnnnnnnnnnnnnnnnnnnnnnnnnnnnnnnnnnnnnnnnnnnnnnnnnnnnnnnTTTCAGCGGAGGTCGCTCTTCTCACGGATCGAAGTTTCATCGTGAAGCGGGTTCCACCGGGCAGTGTACGAGTCCTGGCCGTACGTTTAAAAACGTAAAAATGCCGGGACGTATGGGGGCtgagcgggtgacggtgcagaatctgcGTATTGAACnGATTGATGTGGGTttgggtgtcgtgatggtgcgcggtgcggtgccaggtagaaacaaggccacggtgtttctgcggaccgcggtcaagcgtgaaagataggggtgtatacgcagtggaaaagacagtgtattcnnnnnnnnnnnnnnnnnnnnnnnnnnnnnnnnnnnnnnnnnnnnnnnnnnnnnnnnnnnnnnnnnnnnnnnnnnnnnnnnnnnnnATAAATAGTGAGTTGAGTAACAAGCGCTTGGGGACTGCGTGTACTAAGGGACGTTCCGAAGTGCATGGTTCGAATACCAAGCCCTATAAGCAGAAGGGTACGGGTCGTGCTCGCCGCGGAGATAAGAAGTCTCCACTTCTGGTGGGGGGTGGTACTATATTTGGTCCTAAGCCGCGTGATTTTCACTATGCTCTCCCGAAGAAGGTGAAGCGTTTGGCCATGAAGTCTCTCCTAAGTTTAAAGGCGCAGGGGGATGCnCTGACAGTGATTGAGGACtttacggtcgaaagtggaaaaactagggatctgatacaggtgttgcgtcattttgcacaaaaggagcgtaCCGTnnnnnnnnnnnnnnnnnnnnnnnnnnnnnnnnnnnnnnnnnnnnnnnnnnnnnnnnnnnnnnnnnnnnnnnnnnnnnnnnnnnnnnnnnnnnnnnnnnnnnnnnnnnnnnnnnnnnnnnnnnnnnnnnnnnnnnnnnnacataagatnnnnnnnnnnnnnnnnnnnAAGGATGCTGCACAAGATGGAACATACTGATGTAGTGATTGCTCCGGTGCTTACGGAGAAGTCGAATGCGCTGCGGCAACAGGGTAAGTACGTGTTCCGTGTTGCAGCTCGTGCGACAAAGATTCAGATTAAGCAGGCGGTGACGCAGCTTTTTGGAGTAACGGTTAGGCGGTGTACGGTAATGAATGTCTTTGGGAAGAAGAGGCGTGTTCGTCATCGGACCGGTAGGACGTCTGGGTGGAAGAAGGCGATCGTGCACGTTGCAGCAGGACAGTCAATTGGTGTTCTTGAGCGTGCATAGCGGTAAGCTGCGGTAGCTGCGTAAGGTGCCAGAGCGGTGACCGAAGGAGACGGGGATGGCGTTGAAGATGTATAGGCCTATGAcggcgggcttgcGGGGGCGTGTTGATCTGTGTCGTGCGGAGCTTACCGCGCGCACGCCCGAAAAGAGTCTTACACGCGGTAAGCCTGCCAAGGCGGGCAGGGGTGCTGGGGGTAGGATTTCGGTGCGTCATCGTGGGGGTGGnCATAAGCGGAGGTACCGTGATATCGATTTTAAACGTGATTTGCACGACATACCTGGCACGGTAAAGACTATCGAGTATGACCCGAATCGAAGTGTGAACATCGCGCTTGTGTTTTACGCGAATGGTCAGAAGCGCTATATACTCGCACCCAAGGGTTTGAAGGTGGGACAGCAGGTCGTTAGCGGAGAGAAGGTCCCTTTAGAGCCCGCGAACGCGCTGCCACTCGGGGTAATTCCAGTTGGTTTTACGGTGCATAACgttgaGCTTACGATCGGTAAGGGTGGTCAGATCGCGCGTTCTGCAGGCACCAGGGCGGTGATTGCGGCAAAGGACGGTGGCTATGTGATGCTTCGTTTGCCCTCTGGGGAGGCGCGTCTGGTGCATCGCAGGTGCTATGCCACTATTGGTGAATTAGGTAATGAGGATCATATGAACACGGCTTTGGGGAAGGCAGGTCGTGCGCGTTGGCGTGGGGTGCGGCCGACAGTTCGTGGTATGGCTATGAATCCTGTGGATCACCCGTTAGGTGGTGGTGAAGGGCGTGGTAAGGGACGTAACCCAGTAACTCCCTGGGGGCAGCCGTGTCGAGGATACAAGACGCGCAAGAAGCGCAGGGTATCCGATcgctttatcgtgtcaaagagnnnnnnnnnnnnnnnnnnnnnnnnnnnnnnnnnnnnnnnnnnnnnnnnnnnnnnaaaagCTGTATAAGCGAGTTGTCGAGATGAACAAAGCGGCTAATCAGAGAAATAAAAAGGTGATCAAGTCGTATTCGCGTTGTTCCACCATTATCCCTGAAATGGTGGGCTTCACTATCTCGGTGCACAATGGCAAGTCGTGGATCCCAGTGTACATTACGGnnnnnnnnnnnnnnnnnnnnnnnnnnnnnnnnnnnnnnnnnnnnnnnnnnnnnnnnnnnnnnnnnnnnnnnnnnnnnnnnnnnnnnnnnnnnnnnnnnnnnnnnnnnnnntcacgtatcgagcgaagacaaaatttttggttgcgtctccgacaaaggtgcgtccggttgcgaatgtggtgaagtgcaagccgtatgtgcgcgcgatggcgcttttgggacacnnnnnnnnnnnnnnnnnnnnnnnnnnnnnnnnnnnnnnnnnnnnnnnnnnnnnnnnnncaattgatcgggacaagcgtcttgatgaagagcgcttgttcgtgcgtgacattcagatagatgaggggcctcgtttgaagcgtctgtggtgccggggnnnnnnnnnnnnnnnnnnnnnnnnnnnnnnnnnnnnnnnnnnnnnnnnnnnnnnnnnnnnnnnnnnnnngacgnaagatgggtcaaaaggttagtccaatcggtctgagactggggatcaataaagtatGGTCTTCTAGGTGGTATGCAGGTCCTCGGGAGTACGCGGCGTTGCTGCATGAGGATTTAAGGATTCGTAGCATGATTCGCTCCTTTCCTGAGTGCAAAAATGCGGATATTGCCGAGGTGGAGATTGTCCGTCATCCCCAGCGAGTGACGGTAGTGATGCACACCGCGCGCCCTGGAGTAGTTATtggagCAAAGGGTGTAAATATAGAAAAGATTGGCGCTGAGGTTCAAAAGCGTTTGAATAAGAAGGTTCAAATCAAGGTAAAAGAGATCAAGCGCATGGAGTTAAATGCTTACTTGGTTGCGCAGAATGTTGCTCGCCAACTCACGGCGCGTGTTTCTTTTCGTAAGTGTTTGCGGCAGGCCTGTGCGGGGACGATGAAGTCTGGTGCTCAAGGGGTAAAAATTCGAGTTTCGGGGCGTTTGGGTGGTGCTGAGATGTCTCGCACTGAGGAGATAAAAGAGGGGCGTACGCCTCTGCACACGCTGCGCGCAGATATTGATTATGGTTTTGCCGAGGCACATACGACTTATGGGAGTATCGGGGTAAAGGTGTGGCTATACTCAGGGATGATGTACGGGAAtgagtgtcgcaaagATGTAGGCTCTCTGTTGCGGCGATCGCGCAGGGAGAGTGGCCAAAAGTCTGACGAGTTGGTGCGCGACGAGCGTACGCATGCGGAGAGAGGTTGAGGTATGGCGCTTAGTCCCAAGCGGGTAAAGTACCGAAAGGTACAGCGGGGGAGGGTnAAGGGGGATGCCACTCGGTGCAATGCGGTTGATTTTGGTGCGTACGCGCTGGTGTGTCTTGAGCCGTTTTGGTTgacgagccgacaaatcgaagcggctcgtgtagcgtnnnnnnnnnnnnnnnnnnnnnGGGGTAAGTTGTGGATTCGTGTTTTTCCCGATAAGCCATACAGCAAGAAGCCTGCAGAGACGCgtnnnnnnnnnnnnnnnnnnnnnnnnnnnnnnnnnnnnnnnnnnnnnnnnnnnnnnnnnnnnnnnnnnnnnnnnnnnnnnnnnnnnnnnnnnnnnnnnnnnnnnnnnnnnnnnnnnnnnnnnnnnnnnnnnnnnnnnnnnnnnnnnnnnnnnnnnnnnnnnnnnnnnnnnnnnnnnnnnnnnnnnnnnnnnnnnnGGGTGGGTGTGCGCAATTATCATATTctgagcttcTTTCGAGGCGTCGTGAGCTTGAGAGAAAATACTTGGATCTGCGCTTTCAGCTTGTTGTTGAGCATGTTGACAACAAGCTTATGAAAAGGATTCTCCGTCGTCAAATTGCGGTGGTTAATACTTTTTTGCGACATAAAGAGTTGACTGAACTagaaaagagaggggttcgggagtgatggagnnnnnnnnnnnnnnnnnnnnnnnnnnnnnnnnnnnnnnnnnnnnnnnnnnnnnnnnnnnnnnnnnnnnnnnnnnnnnngttcggattacgacaaagaagttgcacgcgttGTATAAGaagtacgtgtcgCGGAGCAAAAAGTATCAGGCTCATGATGAGGAAAATACCGCGCGGGCAGGGGATGTGGTGCGTATTGCCGAGAGTCGTCCTTTGAGTAGGCGTAAGCGCTGGCGGTTGGTAGAGATTGTTGAACGAGCGAAGTAAGGGATTTGTGTCATGATTCAGGTGCAGTCGCGGTTGAACGTCGCGGATAATTCTGGAGCCAGGTTGGTGCnnnnnnnnnnnnnnnnnnnnnnnnnnnnnnnnnnnnnnnnnnnnnnnnnnnnnnnnnnnnnnnnnnnnnnnnnnnnnnnnnnnnnnnnnnnnnnnnnnnnnnnnnnnnnnnnnnnnnnnnccgtcattgtacgagtttctaaggaatatcgtcgcgtagacggtacttatattcgatTTGACGACAATGCCTGTGTTGTTATCGATGCTAATGGAAATCCTAAGGGGAAGCGTATTTTtggtcctgttgcgcgggagctgcgggatatggannnnnnnnnnnnnnnnnnnnnnnnnnnnnnnnnnnnnnnnnnnnnnnnnnnnnggggnnnnnnnnnnnnnnnnnnnnnnnnnnnnnnnnnnnnnnnnnnnnnnnnnnnnnnnnnnnnnnnnnnnnnnnnnnnnnnnnnnnnnnnnnnnnnnnnnnnnnnnnnnnnnnnnnnnnnnnnnnnnnnnnnnnnnnnnnnnnnnnnnnnnnnnnnncTCAGGATGAGGGGGGTATCATGGAGGTTGAAGCTCCTATTCATATTTCCAACGTTATGATTATGGGCAAGAAGGGGCCTACGCGCGTGGGGTATCGGATGGAAAACGGTAAGAAAGTGAGGGTATGTCGTAAAACAGGAGAGGTGCTATGACCGATCATTCTTGCATACCTGAACTGAAAGTCCGGTATGTGCAGCAGATTGTTCCGGATATGATGCGGGATTTTGGTTACTCGACGGTGATGCAGGTTCCTAAGCTGTTGAAGATAGTGTTGAGTATGGGTCTCGGGGAAGCGCTCGCTAATCGGAAGCTTTTGGACGCGTCAGTAGCAGATTTGGGTGTTATTAGTGGCCAGCATGCAGTAAAGnnnagggcgcgcaagagtattgcgaattttaagctgcgtgaaggcaatgagattggggtgatggtgactctgcgccgtagtaggatgtatgagtttctccaccggctcatcaatgTTGCTCTGCCTCGTGTAAAGGATTTTCGTGGGGTAAGTCCTCGTGGGTTTGATGGACATGGTAATTACTCGATGGGTATTACGGAACAGATTATTTTTCCTGAAATTGACTTTnACAAAATCGAGCGAATTAGCGGTTTGAACGTCAATGTAGtgannnnnnnnnnnnnnnnnnnnnnnnnnnnnnnnnnnnnnnnnnnnnnnnnnnnnnnnnnnnnnnnnnnnnnnnnnnnnnnnnnnnnnnnnnnnnnnnnnnnnnnnnnnnnnnnnnnnnnnnnnnnnnnnnnnnnnnnnnntacaaccgctGTGGGGTGTGTGGGCGACCCCGCGGGTACATGAGGAGATTTCAATTGTGCCGCCTGTGTTTTAGAAAGCTGGCGAGCGAGGGTCAAATCCCTGGGGTAACGAAGTCGAGTTGGTAGnnnnnnnnnnnnnnnnnnnnnnnnnnnnnnnnnnnnnnnnnnnnnnnnnnnnnnnnnnnnnnnnnnnnnnnnnnnggacatgaannnnnnnnnnnnnnnnnnnnnnnnnnnnnnnnnnnnnnnnnnnnnntactgaaaacggaaggatatatcaggaacttcaggaaagtagaggaggatggttccggttgtattcgtgtgttTCTTAAGTATGACGATAACGAAACGTCGGTTATTCACGGTATCGAGCGGATTTCTACTCCGGGCCGCCGTGTGTACTCGGGGTACAAGACGCTTCGTCGTGTGTATAACGGGTACGGCACTTTGATTGTTTCTACCTCTCTAGGGGTGACCACTGGCAGGCATGCAAGGGAGCAGCGTGtnnnnnnnnnnnnnnnnnnnnnnGTTTGGTAGGGGGCTGTAGTGTCAAGAATTGGTAAAGTTCCTGTGTCTGTTCCTGGCGGTGTGCACGTGCGAGTCTCTTCTGGGGTGGTTGAGGTCGAGGGTCCAAAGGGGGTGCTTTCGTGTGCGTTTCTCCCAGTGGTTACGGTTCGTGTTGAGCAGGAATACGTAATTGTTGCCCGGTGTGATGATTCCAAGCGCGCGCGTGCATGTCATGGGCTGTATCGCAAGCTTTTGAGCAATATGGTAGTTGGGGTAAGCGAAGGGTTTTCTAAGACATTGGTAATTACGGGTATCGGGTACCGCGCTGAGGTTCAAGGGCGGGTGCTGGTGATGGCATTGGGTTACTCCAATGACTTTACAGTGCTCATTCCCTCTGGTATTGAGGTGCGGGTTGAGTCTTCCACGAGGGTTATTGTTTCCGGTGTAAGTAAGGAAAGAGTGGGGGAGTTCGCAGCGCAACTTCGTAGGCTGCGGTTGCCTGAGGnnnnnnnnnnnnnnnnnnnnnnnnnnnnnnnnnnnnnnnnnnnnnnnnnnnnnnnnnnnnnnnnnnnnnnnnnnnnnnnnnnntgctaaggaagtgcagtgataaacagcgaaagaggatgaagcgtaaggttcatattaggaagagggtgtatggcacggcggttcgccctcggatgacggtgttccgaagtaatcgGAACATTTCGGTGCAGGTCATTGACGACGACGCGCGtagcacgcttgcgtcagtttctACTCTTGAGAAGGATTTTGTTCTGCTTAGGGCAAATGTTTCTTCTGGTTTGCAGATAGGAGAAGAGATCGGCAGGCGCCTTTTAGAGAAACACATTGACACGGTTATCTTTGACCGAAATGGGTACTTGTACCACGGGGTAGTGGCGGCCGTCGCAGATGGTGCGCGTAAGGCAGGAGTTAAGTTCTAGGAGAGCGTATGGATCGTCACAGGGATTTTGGCAAAGACAGACTTCGAGACAAAGAGTTTACCGAGAAATTAATCAAGCTGAACCGCACGGCAAAGGTAGTAAAGggcggacgtcggttttccttttcggcactCACGGTAGTTGGTGATCAAAAGGGCCGCGTGGGGTTTGGTTTTGGTAAAGCCGGGGATGTGAGCGAGGCAATTAGGAAGAGTGTTGAAAGGGCGAAGCGGAGTATGGTGCTCTTTCCGCTCAAGGATGGTACCATCCCGCATGAAGTACAAGCTaagtttaagggctctctggtgttactgnnnnnnnnnnnnnnnGGTACGGGTATTATTGCTGGTGGAACCGTGCGTGCTATCATGGAGGTTGCAGGTGCAACCGATGTGCTGTCTAAGTCTTTGGGTTCGAATTCTGCTATCAACGTGGTTCGTGCAACGTTTGGGGCGGTTGCGCAGTTGATGGATGCAAGAAnnnnnnnnnnnnnnnnnnnnnnnnnnnnnnnnnnnnnnnnnnnnnnnnnnnnnnnnnnnnnnnnnnnnnnnnnnnnnngaggagtacgatcggccagagggagccggtgcgtcGGACGGTTCGGTCTTTGGGTTTGAGGAAGTTGCATTCAATGGTGGAGAAAGACGGGAGTCCTGCCGTCTTGGGGATGGTGCGAGCTGTTTCGCACCTggtgcgggtggaggagttannnnnnnnnnnnnnnnnnnTTTGATTGCTCCGAAGGGGGCTAATAGGGCGCGTCGTATCGTGGGnCGTGGGTCCTCCTCTGGGCGGGGTACCACGTCTGGGCGGGGTACTAAGGGACAGCAGGCCCGTGCGGGGCATAAGGCTTATGTAGGTTTTGAGGGTGGGCAGATGCCGCTATATCGGCGTGTGCCGCGGCGGGGTTTTTCTAACTGTGCTTTCAAAAAGGAATACGCGGTAGTTAATGTGGGCGCGCTTGAGTTTGTCTATGCTCCAGGGGAGACGGTCaacagacaGACTCTCATTGAGAAGGGATTGGTAAAGGGGCGGGTCCCCTTCATCAAAATCTTGGCAGACGGAGAGCTGACAAAGTCTATTGTGGTGCGGGTGGACCGGGTTTCTGCTCGTGCACAGGAGAAGATTCAGCAGGCGGGCGGTTCAGTGGAGtgtattgaagcgcaggaacgatgnnnnnnnnnnnnnnnnnnnnnnnnnnnnnnnnnnnnnnnnnnnnnnnnnnnnnnnnnnnnnnnnnnnnnnnnnnnnnnnnnnnnnnnnnnnnnnnnnnnnnnnnnnnnnnnnnnnnnnnnnnnnnnnnnnnnnnnnnnnnctttctgcttatttccgatctcaggttcggggaaatgcttttgcagactacatggatttttttgtaggcggggcgttctcgaatttttcagtgtttatgctgggnnnnnnnnnnnnnnnnnnnnnnnnnnnnnnnnnnnnnnnnnnnnnnnnnnnnnnnnnnngtcttaagaaggttgtagaagatgtaggggggagacgtcgcgttcagttttggacacgtgttgcaacggtttttgtgtGTCTTATACnnnnnnnnnnnnnnnnnnnnnnnnnnnnnnnnnnnnnnnnnnnnnnnnnnnnnnnnnnnnnnnnnnnnnnnnnnnnnnnnnnnnnnnnnnnnnnnnnnnnnnnnnnnnnnnnnnnnnnnnnGCTTGGGGAACAGATCACAGCgcgaggcattggtaacggtgtgtcaatgattattttTTCGGGTATTGTCGCGCGTTTGCCTCATGCGCTTGCAGAGATGTGGAGGCTGCAGCGTCTTGGCGAGTTGAATATGGTGTTTGTGATCGTTGCGtttgtgatgtttgtaggaattattgtgctggtggtgtatgagcagcaggggcaacgannnnnnnnnattcattatgcgcggcgtgtggtcgggcggaaaatgtacggtnnnnnnnnnnnnnnnnnnnnnnnnnnnnnnnnnnnnnnnnnnnnnnnnnnnnnnnnnnnnnnnnnnnnnnnnnnnnnnnnnnnnnnnnnnnnnnncagcaGTATTGGACCGAACGTGCGCTTTCTGCATCAGCTTGCGCAGTTCTTACGACCGAACAGTTGGTGGTACAACGCGTTCTATGTnnnnttgattgtgttttttgcgtacttctacacgcaagtcacccttaacccgactgagatagcannnnnnnnnnnnnnnnnnnnnnnnnnnnnnnnnnnnnnnnnnnnnnnnnnnnnnnnnnnnnnnnnnnnnnnnnnnnnnnnnnnnnnnnnnnnnnnnnnnnnnnnnnnnnnnnnnnnnnnnnnnnnnnnnnnnnnnnnnnnnnnnnnnnnnnnnnnnnnnnnnnnnnnnnnnnnnnnnnnnnnnnnnnnnnnnnnnnnnnnnnnnnnnnnnnnnnnnnnnnnnnnnnnnnnnnnnnnnnnnnnnnnnnnnnnnnnnnnnnnnnnnnnnnnnnnnnnnnnnnnnnnnnnnnnnnnnnnnnnnnnnnnnnnnnnnnnnnnnnnnnnnnnnnnnnnnnnnnnnnnnnnnnnnnnnnnnnnnnnnnnnnnnnnnnnnnnnnnnnnnnnnnnnnnnnnnnnnnnnnnnnnnnnnnnnnnnnnnnnnnnnnnnnnnnnnnnnnnnnnnnnnnnnnnnnnnnnnnnnnnnnnnnnnnnnnnnnnnnnnnnnnnnnnnnnnnnnnnnnnnnnnnnnnnnnnnnnnnnnnnnnnnnnnnnnnnnnnnnnnnnnnnnnnnnnnnnnnnnnnnnnnnnnnnnnnnnnnnnnnnnnnnnnnnnnnnnnnnnnnnnnnnnnnnnnnnnnnnnnnnnnnnnnnnnnnnnnnnnnnnnnnnnnnnnnnnnnnnnnnnngaatacaaagtggaaggtcgtctgagaactgaggttgccttaaatatcaagaggttgatggatattgggtgttaccgagggctaagacatagaaaggggctgccnnnnnnnnnnnnnnnnnnnnnnnnnnnnnnnnnnnnnnnnnnnnnnnnnnnnnnnnnnnnnnnnnnnnnnnnnnnnnnnnnnnnnnnnnnnnnnnnnnnnnnnnnnnnnnnnnnnnnnnnnnnnnnnnnnnnnnnnnnnnnnnnnnnnnnnnnnnnnnnnnnnnnnnnnnnnnnnnnnnnnnnnnnnnnnnnnnnnnnnnnnnnnnnnnnnnnnnnnnnnnnnnnnnnnnnnnnnnnnnGGTTTAATGGGGCAAAGAAATCTACTCCTTTTGCAGCACAGACGGTCGCGGAAGCTGCGGTACAGAAAGCGCAGCAGTGCGGACTGCGTGAAGTACATGTGTTTGTCAAAGGGCCGGGTATTGGGcgtgagtcagcaattagaatgcttggtaccatgggactgagggtgcgttcgattcgcgacatcacacccattccacataacggctgtcgnnnnnnnnnnnnnnnnnnnnnnnnnnnnnnnnnnnnnnnnnnnnnnnnnnnnnnnnnnnnnnnnnnnnnnnnnnnnnnnnnnnnnnnnnnnnnnnnnnnnnnnnnnnnnnnnnnnnnnnnnnnnnnnnnnnnnnnnnnnnnnnnnnnnnnnnnnnnnnnnnnnnnnnnnnnnnnnnnnnnnnnnnnnnnnnnnnnnnnnnnnnnnnnnnnnnnnnnnnnnnnnnnnnnnnnnnnnnnnnnnnnnnnnnnnnnnnnnnnnnnnnnnnnnnnnnnnnnnnnnnnnnnnnnnnnnnnnnnnnnnnnnnnnnnnnnnnnnnnnnnnnnnnnnnnnnnnnnnnnnnnnnnnnnnnnnnnnnncngggggcagagtctagtacgttcacgtatgagtttcgcggcgcggtnnnnnnnnnnnnnnnnnnnnnnnnnnnnnnnnnnnnnnnnnnnnnnnnnnnnnnnnnnnnnnnnnnnnnnnnnnnnnnnnnnnnnnnnnnnnnnnnnnnnnnnnnnnncgtcgaattcgggcgtgggtatgtacctgctgaatcgcacgatcggtatgccgatttagttggggttatccctgttgacgcaatttttagtcccgtgttgagagtccgctatgatattcagtcttgccgtgtaggtcnnnnnnnnnnnnnnnnnnnnnnnnnnnnnnnnnnnnnnnnnnnnnnnnnnnnnnnnnnnnnnnnnnnnnnnnnnnnnnnnnnnnnnnnnnnnnnnnnnnnnnnnnnnnnnnnnnnnnnnnnnnnnnnnnnnnnnnnnnnnnnnnnnnnnnnnnnnnnnnnnnnnnnnnnnnnnnnnnnnnnnnnnnnnnnnnnnnnnnnnnnnnnnnnnnnnnnnnnnnnnnnnnnnnnnnnnnnnnnnnnnnnnnnnnnnnnnnnnnnnnnnnnnnnnnnnnnnnnnnnnnnnnnnnnnnnnnnnnnnnnnnnnnnnnnnnnnnnnnnnnnnnnnnnnnnnnnnnnnnnnnnnnnnnnnnnnnnnnnnnnnnnnnnnnnnnnnnnnnnnnnnnnnnnnnnnnnnnnnnnnnnnnnnnnnnnnnnnnnnnnnnnnnnnnnnnnnnnnnnnnnnnnnnnnnnnnnnnnnnnnnnnnnnnnnnnnnnnnnnnnnnnnnnnnnnnnnnnnnnnnnnnnnnnnnnnnnnnnnnnnnnnnnnnnnnnnnnnnnnnnnnnnnnnnnnnnnnnnnnnnnnnnnnnnnnnnnnnnnnnnnnnnnnnnnnnnnnnnnnnnnnnnnnnnnnnnnnnnnnnnnnnnnnnnnnnnnnnnnnnnnnnnnnnnnnnnnnnnnnnnnnnnnnnnnnnnnnnnnnnnnnnnnnnnnnnnnnnnnnnnnnnnnnnnnnnnnnnnnnnnnnnnnnnnnnnnnnnnnnnnnnnnnnnnnnnnnnnnnnnnnnnnnnnnnnnnnnnnnnnnnnnnnnnnnnnnnnnnnnnnnnnnnnnnnnnnnnnnnnnnnnnnnnnnnnnnnnnnnnnnnnnnnnnnnnnnnnnnnnnnnnnnnnnnnnnnnnnnnnnnnnnnnnnnnnnnnnnnnnnnnnnnnnnnnnnnnnnnnnnnnnnnnnnnnnnnnnnnnnnnnnnnnnnnnnnnnnnnnnnnnnnnnnnnnnnnnnnnnnnnnnnnnnnnnnnnnnnnnnnnnnnnnnnnnnnnnnnnnnnnnnnnnnnnnnnnnnnnnnnnnnnnnnngtatgagtgcgagattgatggtaagaaggtcaaggtttccaaggttgggcgcgcgactctccaGAATAGGAAGAGACGTTTGGATGCGCAGCCTGGAGCTTGATCGCGCATCCTCGTGATATGAGGTTCCGTCCCAAGGACGTTAGGTGGTtgtccgtttctgtgcttggcagttaccattgggatgcnnnnnnnnnnnnnnnnnnnnnagtcagacgGTAAATAGGTGTTTTCTTGACCGAGGGCGGCGTCTCTCGTTACTTTTACGGCATTACCGCGAGGGTGTTATGGCAAAAaaggagaagaaagtgtgcggcggcgacgttcaggggcagggagttgcctcaGGTTGTGACGAGGCCTTGGAGCGGGCAGATAGCCTTCGCGCGTCTGATCCTGTACCGGTTGAATCGGGGGAGGGTTCTGTTCCTGGGGAGCATAGTCAGGAGTTGGAGACAGGTGCCTCTGAAGAGACCCTGCGCGAGCGCGTGAATGTTTTGCAGGAGCAGTACCTGCGCAAGGCTGCCGACCTCGAAAACTACCGGAAGCGTGCGTTGCGGGAAAGnCAGGAGGCGGTGGAACACGCGTACGCGGCGCTGCTTGCCGACATCGTCGCTGTCTTGGATGACTTTGACCGTGCTATTGAAGCGGCGGATCACGCGTCGAGTACAGAGGTGGAGGCTTCATCTGCCTTCCGAGAGGGTGTTCTTATGATCCGCAAGCAGCTCTCCTCAGTGCTTGAGACAAAGTATGGTCTTGAGTATTACCCGGTGCTCGGGGAGCGCTTCGATCCAAATCTCCATGAGGCTTTGAGTATGAGTCCTTCCGCTTCTGTGCATGAGAAGATAGTAGGGGCAGAGCTACAAnnnnnnnnnnnnnnnnnnnnnnnnnnnnnnnnnnnnnnnnnnnnnnnnnnnnnnnnnnnnnnnnnnnnnnnnnnnnnnnnnnnnnnnnnnnnnnnnnnnnnnnnnnnnnnnnnnnnnnnnnnnnnnnnnnnnnnnnnnnnnnnnnnnnnnnnnnnnnnnnnnnnnnnnnnnnnnnnnnnnnnnnnnnnnnnnnnnatcctatacagaggagttgagggttatggggaagattattggcattgacttgggaacgacaaattcgtgtgttgcgatcatggaggggggggagcccgttgtcattcaaaatgccgaagggggaaggactacgccCTCCATTATCGGTTTCACCTCTGATGGTGGACGCGTCGTCGGTCAGCCAGCAAAAAACCAAATGGTTACTAATCCGGAACATACTATCTATTCGATAAAGCGCTTTATCGGCAGTCGTTTCAATGAACTGACCGGTGAAGCAAAAAAGGTGCCCTACAAAATTGTTCCACAGGGAGACGACGTGCGCGTTGAGGTGGAGGGTAAGCTTTACTCTACGCAGGAGATCTCCGCGTTCATTTTGCAAAAAATGAAGAAGACAGCTGAGGATTATTTGGGCGAGGCAGTCACAGAGGCAGTCATTACCGTTCCGGCTTACTTTAACGATGCACAGCGTCAGGCAACCAAGGATGCGGGGAAGATAGCAGGGCTCGATGTGAAGCGTATTATTAATGAGCCGACTGCTGCGTCGCTTGCCTTTGGTTTTAACAAAGACTCTAAGAGAGAGAAGATTATTGCTGTGTATGATCTTGGGGGGGGTACCTTTGACATATCCATCTTGGAACTCGGTGACGGTGTTTTTGAAGTCAAGTCAACGAATGGGGACACTCACCTGGggggcgatgnnnnnnnnnnACGTATCGTGCAATGGCTGGAGCAGGGCTTCAAGAGTGACACGGGTATCGACTTGGGCAACGACCGCATGGCGTTGCAGCGGCTGAGAGAAGCGGCGGAGAAAGCAAAGATAGCGCTTTCTTCCTCTGCGAGTACCGAGATTAATTTGCCCTTCATTACTGCAGATGCCAATGGGCCAAAGCATCTCCAGAGGACTCTCTCTCGATCTGAGTTTGAGAAGATGACTGATGATCTTTTTGAGCGGACCAAAGAGCCTTGCCGCAAGGCGCTCAAAGACGCCGGAATTAGTGCGGACAGGATCGATGAGATTCTCTTAGTTGGTGGTTCCACGCGCATGCCCAAAGTAGCGCACGTGATCAAAGATGTCTTTGGGAAAGAAGGATCGAAGGGAGTCAATCCTGACGAGGCTGTCGCAATTGGCGCTGCAATTCAAGGAGGTATCCTCGGGGGGGACGTGAAGGATGTACTTCTCTTAGACGTTACGCCTCTTTCTCTAGGAATTGAAACAATGGGCGGGGTGTTCACTCCGCTTATCAGTCGTAATACCACnnnnnnnnnnnnnnnnnnnnaggtgttttccaccgcagctgatgggcagacggcagtttccattcacgtgctgcagggggagcgtggcatGGCGAACCAAAACCGGACGCTCGGTAATTTTGATCTAGTAGGAATTCCCCCTGCTCCGCGGGGAGTGCCGCAAATTGAAGTgacgtttgacattgatgcgaatggtatnnnnnnnnnnnnnnnnnnnnnnnnnnnnnnnnnnnnnnnnnnnnnnnnnnnnnnnnnnnnnnnnnnnnnnnnnnnnnnnnnnnnnnnnnnnnnnnnnnnnnnnnnnnnnnnnnnnnnnnnnnnnnnnnnnnnnnnnnnnnnnnnnnnnnnnnnnnnnnnnnnnnnnnnnnnnnnnnnnnnnnnnnnnnnnnnnnnnnnnnnnnnnnnnnnnnnnnnnnnnnnnnnnnnnnnnGACCGCGCGCGCATAGACGAGGCGATCGCAGAGTTGAAGACGGTGCTCTCAGGCGACGACGTCGCATCGATCAAAGCGAAGACTGAGATCTTGCAGCAAGCTTCCTACAAAATTGCGGAGGAAATGTATAAACGTCaAGCAGCAGCGGGTGccgctgcaggtaagaagagtgATGCACCCTCTGGCAATGAGGCAGAAGGtggtGACGTTGATTACGAGGTAGTGAAGGACGAAGATTCAAAGTAGACATCTGGTGTTGCGGGGAGGGAATAGCCTGCGTGTAGGAGCTGTGTGATCTGACTTCCCCCAGGCCTTTTGTGATCCGGGTGTTCGCCTGATCGCCCGGGTCTTTCGGCTGTCTAGTGGGTGTTTGGATGTAGCCTGCGTAGGCGGTGCTTCAGGCGTCCTGCTTnnnnnnnnnnnnnnnnnnnnnnccctgtttttctgtgtgtgcgcgcaaatgtagacaaagattctctagacggggtgatcgTGGCAAAGAAGGATTATTACGAGGTTCTCGGTATCTCAAAGACCGCGAGTGGAGAAGAAATCAAAAAGGCGTACCGGCGGCTGGCTATTCAGTTTCATCCTGACCGTAATCAGGGAAATAAAGAGGCGGAGGAACGCTTCAAGGAGGCTACCGAAGCCTATGAGGTGCTCATTGATGCACAGAAGCGTGCCGCGTACGATCGGTATGGCTTTGATGGCCTGAAGGATATGCACGGTGCGCATGGCTTTAACTCTTCGGCCTTTCAGGGGTTCGAAGATATTTTTGGGGGTGGCTTTTCTGATATCTTTGAAAATATTTTTGGGACTTCGTCTCGCCGCGGCGGTTCAGGGAACGACGGCTCGGGTGGCTCCGGGCGTGGGGCAAACTTGCGTTATGATTTGCAAATCTCTTTTGAAGAAGCAGTGTACGGGAAAAAGAGTGAGCTGCACTATGTGCGCGACGAAACGTGTATTACCTGCAAGGGTGCCGGCTCGGCCAGCGGTGGGCGTAAGATGTGTCCAGATTGCAAGGGTACGGGGCAGATTCGGCGTAGTACAGGTTTTTTCTCTATTGCGCAAAGTTGTGCGCGCTGTGGTGGTGAGGGGACGATTATCGAAAGtccctgtgcacgGTGTGCGGGTAGTGGCATTGAGCGTAAAAAGCAAAAAATTATCGTCAGTATTCCGGCAGGTGTAGAAGAAGGGCGGCGCATTACTATTCCCCGTCAGGGAAACGCCGGTCGCGCAGGCGGTGCCTACGGGGACCTGTACGTGTTTGTGTTTGTTCGTGCGCATGAGTATTTCGAACGTGAAGGTGCTGACCTGTACTGTGCAACTTCGATATCGGTAACCCAAGCGATTTTGGGCGCGCAGGTGACGGTGCGGGCATTAGATGGATCTGCGCAGCAGgtgcgggttcnnnnnnnnnnnnnnnnnnnnnnnnnnnnnnnnnnnnnnnnnnnnnnnnnnnnnnnnnnnnnnnnnnnnnnnnnnnnnnnnnnnnnngtattggtgcgTATTCCAACTACGCTTTCTGCACGGTCGCGTGCGCTCTTAGCGGAGATTTCTCAAGAGGAAGGGGAAAACGCCCATCCGCCGTTGCTtgaactttcaagtctcaagtaggCTACAGAAAGGGGCGCGTGGGGTAAAAGGATTATTCTCGCGTGCGTGGTGTTTCTTTCTCGTGTGTCGCAGGATGAGTTGGCTTCATCGTGATGGGTGCGTGTGCTATCTGAGTTTTCTTCCCACAGTTTAAAAGACAACGTGTTTTTGAAGcAGCCATACAAAGGGAACGGTAGGTGATTCGCAGAAGGCTCGCAATTGTAAAGGCAGGTTCATTCGcactcctggcgctttttttttcaatatttttgcgctttctcagtccgcggtattcgtttctcggtcgtttcgtttctGCGCGCGATatggcgctgttgatttctcggtatgagcatttgcctgagctttcttcgcgtgatcgagccttgCTGGTAGGTTTCGTTTTCATGATTTTTGCGCTTGCGCTTACAGAAATCCAACGCTATGCGCACGGGCGCATCCGTCTTGTTGTCTATCGACAAACTGAAACGCGTCTTTTTGAACATTTTATTACACnnnnnnnnnnnnnnnaccgtaatgaacaacttgttgctgccttccaggaggtgcttgaatatcgcgccgattgcgcagtgcnnnnnnnnnnnnnnnnnnnnnnnnnnnnnnnnnnnnnnnnnnnnnnnnnnnnnnnnnnnnnnnnnnnnnnnnnnnnnnnnnnnnnnnnnnnnnnnnnnnnnnnnnnnnnnnnnnnnnnnnnnnnnnnnnnnnnnnnnnnnnnnnnnnnnnnnnnnnnnnnnnnnnnnnnnnnnnnnnnnnnnnnnnnnnnnnnnnnnnnnnnnnnnnnnnnTCTTTTTATAGTATGTCGTTTTATCCGTGCTGTTGAGCGCGAAGTTATATCGCTCTTGTTCAAAGAGTTTGAAGAGTATTTAAATCGAAAGAGGAACATGAGCACTTTGTTGTTGTACTCCCATGTATCCCAGGAATGGGCAATGATGGCGCGTGTACAGCGCGCACTTTTGCCTAAGGCGCTCCCACATACCAAGGAAATATGCGTCGGGGCATTCTATCAGCCACTGGTTAATGTATCTGGGGATTATTACGACGTTATTTCAATTGATGAACACCTGTTTTTATTTGtaattggagatgtttctggcaaAGGTTTGGCCGCGTCGTTAGTTATGGGTGTTGTGCTCAGTACGATTCgcatcgtggaagacaaaaagaatcttcctgagattttgtatgcggtggaccgggcagtaaagcgtatgcatctccacgataagtacacgaccttattcttgggattAATTGACACAGCGGGTATGAATattCGCTACATTAACGCATCGATGGAAAGTCCCCTTGTCTTTACCCAGGCGGGAGAGGCGCACGAAGTGTATCATCTTCATTCnnnnnnnnnnnnnnnnnnnnnnnnnnnnnnnnnnnnnnnnnnnnnnnnnnnnnnnnnnnnnnnnnnnnnnnnnnnnnnnnnnnnngtatcagacggggtagttgaagtagtcaatgcccaagGnnnnnnnnnnnnnnnnnnnnnnnnnnnnnnnnnnnnnnnnnnnnnnnnnnnnnnnnnnnnncctgcgcagatggctcaaagtattgcagctctagcattgaatttcagtgcggatcgcactacccatgacGACATGACCATTGTGACTGTGCAGGTGAAGAGGTAGGTAATGGGGTTGCTATTTTTTTCTTACCTCTTTGTCGCGGTTTTTGTTTTCGACGTATTCTGTTTAAAGTTTTACACAAGGCGCGCGTATGCACTGCTGGCAAATATAGCCTTGGTATCAGGnnnnnnnnnnnnnnnnnnnnnnnnnnnnnnnnnnnnnnnnnnnnnnnnnnnnnnnnnnnnnnnnnnnnnnnnnnnnnnnnnnnnnnnnnnnnnnnnnnnnnnnnnnnnnnnnnnnnnnnnnnnnnnnnnnnnnnnnnnnnnnnnnnnnnnnnnnnnnnnnnnnnnnnnnnnnnnnnnnnnnnnnnnnnnnnnnnnnnnnnnnnnnnnnnnnnnnnnnnnnnnnnnnnnnnnnnnnnnnnnnnnnnnnnnnnnnnnnnnnnnnnnnnnnnnnnnnnnnnnnnnnnnnnnnnnnnnnnnnnnnnnnnnnnnnnnnnnnnnnnnnnnnnnnnnnnnnnnnnnnnnnnnnnnnnnnnnnnnnnnnnnnnnnnnnnnnnnnnnnnnnnnnnnnnnnnnnnnnnnnnnnnnnnnnnnnnnnnnnnnnnnnnnnnnnnnnnnnnnnnnnnnnnnnnnnnnnnnnnnnnnnnnnnnnnnnnnnnnnnnnnnnnnnnnnnnnnnnnnnnnnnnnnnnnnnnnnnnnnnnnnnnnnnnnnnnnnnnnnnnnnnnnnnnnnnnnnnnnnnnnnnnnnnnnnnnnnnnnnnnnnnnnnnnnnnnnnnnnnnnnnnnnnnnnnnnnnnnnnnnnnnnnnnnnnnnnnnnnnnnnnnnnnnnnnnnnnnnnnnnnnnnnnnnnnnnnnnnnnnnnnnnnnnnnnnnnnnnnnnnnnnnnnnnnnnnnnnnnnnnnnnnnnnnnnnnnnnnnnnnnnnnnnnnnnnnnnnnnnnnnnnnnnnnnnnnnnnnnnnnnnnnnnnnnnnnnnnnnnnnnnnnnnnnnnnnnnnnnnnnnnnnnnnnnnnnnnnnnnnnnnnnnnnnnnnnnnnnnnnnntatgcgcatcgcgtgtagaggattgacatttttacttgctgacgaggcaggcgtattcaagacggtgtattctgattttgaagagatggcaacgcatgagatggaacatcagtcgttacaGTATTTTTTCAATCAAGATGTTCTTATGATCagaaaatcggatccagttattgaccatgcacgtcaggcaattcggacagatatcctgcatttttttgatataacgcnnnnnnnnnnnnnnnnnnnnnnnnnnnnnnnnnnnnnnnnnnnnnnnnnnnnnnnnnnnnnnnnnnnnnnnnnnnnnnnnnnnnnnnnnnnnnnnnnnnagcatgttttagaaaagttgtattcacactttgtattggtttcctattacttacagaatattgcaaagcaagacgtggttatcacggtggacaaagaacttaaaatgtcccatcagattattgagtcaatacaacggaaaagggannnnnnnnnnnnnnnnnnnnnnnnnnnnnnnnnnnnnnnnnnnnnnnnnnnnnnnnnnnnnnnnnnnnnnnnnnnnnnnnnnnnnnnnnnnnnnnnnnnnnnnnnnnnnnnnnnnnnnnnnnnnnnnnnnnnnggtctggcagcgagtatgtcaatggtgattttgaagtctgtactgagtacctttctgcggggactgtgcctggaagaaacggcagtatttacaacctttattgnnnnnnnnnnnnnnnnnnnnnnnnnnnnnnnnnnnnnnnnnnnnnnnnnnnnnngtgtattctgtattcttgacctggcaacccATACGCTCTACTATGCGAACTGTGGCATACCGCTCATGTCGATGTACGTCGCTTCATACAAGAACGTGGTGGAGATACAAGGCGAGGGGCGCGTGCTGGGTTTTGTTAAAGATGTTATGCCCTTTTTGCGGGTGAGGAAAGTTCAACTCGGTCAGGGGGacgtggnnnnnnnnnnnnnnnnnnnnnnnnnnnnnnnnnnnnnnnnnnnnnnnnnnnnnnnnggtaannnnnnnnnnnnnnnnnnnnnnnnnnnnnnnnnnnnnnnnnnnnnttctcaaattacccgtactatttatgctcggctgtgtgagtttatggcgcgagatatgcaggatgatgtaactgttctggcaataaagtgccttgggcctcnnnnnnnnnnnnnnnnnnnnnnnnnnnnnnnnnnnnnnnnnnnnnnnnnnnnnnnnnnnnnnnnnnnnnnnnnnnnnnnnnnnnacggtgactggagcggtcagctcctatacttacggggagtttgagtcgcgtgtgcatggggcgctcaaagagaatcacgttgttttggatctctccggcgtgacggctatgtcttcttcgGGATTGGGGGnnnnnnnnnntgcatacgATGAGGGACTGAAGTACCAGCGTCGTCTGTGCATTCTTAATCCTTCTGAGAGCGTACGCAGAGCGATAGAGCTGACCGGCTTCTCTGAAATGTTCACGGTAATTAAATCATTAGATGAGTTGGACTAAGTGGCGCGTTGGAACtgcgtgtgcgtatgcagggtaCGTGTTAGTTGCTCTGGGAAATGCGGTCTCGCCGCAGGGCGTGGATCGGGTGGATCTGCATCGTGTGCGTGCTGCTGCGAAGCGCGCGGCGCTTCCTGCGCGCCTTGCTACGCGTTTGTGTGCACAGGGGAGGGAGTTTTTGCAAGAGCTCGACGcggtacnnnnnnnnnnnnnnnnnnnnnnnnnnnnnnnnnnnnnnnnnnnnnnnnnnnnnnnnnnnnnnnnnnnnnnnnnnnnnnnnnnnnnnnnnnnnnnnnnnnnnnnnnnnnnnnnnnnnnnnnnnnnCCTTTTAACTAAGATTGCGTACGAAGCGTTACATCAGCTGATTCAGGCGGCTGCGCATGACGGGGTGGCACTGTCCGTTGGTTCTGCCTATCGCTCTTTTGCGTATCAGAAAAAGTTGTTCTCCTGGTATGCGCAGGAGCATGGCATGCAggaggctatgcgtttttccgcgcgagaaggaacaagtcagcaccagttggggacggtagtggatttcggttctatcacgccnnnnnnnnnnnnnnnnnnnnnnnnnnggtggacgcagcgcaacgcccatcgttttggatggtcgctgtcttttccccctggttacgagcaggtgacggggtatgtatgggagcnnnnnnnnnnnnnnnnnnnnnnnnnnnnnnnnnnnnnnnnnnnnnnnnnnnnnnnnnnnnnnnnnnngcaatacatgcttggatttattcatgaatggaaggtgaacgcatcgtcgtgaggagaccgaatgtggaagcggggtgtgggcgcaCTATTGTGGGGTATGGtgttgttgcgctcttttgtggcatatgcggattcgcaggcggctactggtnnnnnnnnnnnnnnnnnnnnnnnnnnnnnnnnnnnnnnnnnnnnnnnnnnnnnnnnnnnnnnnnnnnnnnnnnnnnnnnnnnnnnnnnnnnnnnnnnnnnnnnnnnnnnnnnnnnagacgctcgacgtgaaattggagaaggccgccaaggccttggaggaatcggagcacgcattgagcgaaTCGAAAGAGTTGATAGAGACATTGAGAAGCGAATTGGAGATATTGAGGCAGCGTGTGAACGCATTGAACATGCGGTTGAGGCGtttagagatcacgaataatgttCTTATCGGGGTTGCAGTTGTGTGCGGTGTAGCCGCTATTGGTGCAGGGATATATGCTGCGGTGAAGTGACGGTGtttgtgtaaccatttgccgcaggcgagctcatccgtgtggctgcgcggacttgtgtgctctgaagtcgggctgtcgannnnnnnnnnnnnnnnnnnnnnnnnnnnnnnnnnnnnTGTCTACACtgtgagacatggacctctctgaaggcgcgcgtgcgttaaacgctgctcttgaaggaacggtggtgcacgcgctgttatctcctcttggtttgcgcgcgtattttccccacgGGATTGTTGcacagaatgcaGAGGCGCGGATGCGTGCGCCTGATATGAATGGGGCGGTTGGCATGGTTCTGCGTCATGGGGTGCCGGTGCTTCGTGATACGTTGCATGCGCTTGTTCCTGATCTTTCTTCTTCTGAAATAGTTTCCTAcgcgccgactgcagGTATTCCTGCGCTCCGCGAGCGCTGGGCGCGGCGTTTGTGTGCGCGTGATCCTGTGTTGTGTCCGGATGAACCGGACGTTGCAGCAATGCAGAGTGCAGCAGagcagggagagcattcttgtgcggcannnnnnnnnnnnnnnnnnnnnnnnnnnnnnnnnnnnnnnnnnnnnnnnnnnnnnnnnnnnnnnnnnnnnnnnnnnnnnnnnnnnnnnnnnnnnnnnnnnnnnnnnnnnnnnnnnnnnnnnnnnnnnnnnnnnnnnnnnnnagtGCGTTGTGCGGCAACGCCGGTTCCCTTTTCTCTTTTTCGAGCGGGGCGCTTTGATTTGAGCGCGTGCAAGGAAGCGCTGGACGCACAGGCTTCGGATGGGGTGATAAGGTTAATTTTGAATTTCCCCCATAATCCGTCAGGTTATACCCCCACGGAAGAGGAGGCACAGCAATTATACGAGGTGGTGTATGCTtatgctgacgcagggtgtgcggtactggtgatttgtgacgatgcgtatagcgggttcgagtatgaggnnnnnnnnnnnnnnnnnnnnnnnnnnnnnnnnnnnnnnnnnnnnnnnnnnnnnnnnnnnncgttaaagatagatgnnnnnnnnnnnnnnnnnnnnnnnnnnnnnnnnnnnnnnnnnnnnnnnnnnnnnnnnnnnnnnnnnnnnnnnnnnnnnnnnnnnnnnnnnnnnnnnnnnnnnnnnnnnGGGATGATCCGTGCATCACTTTCATGTACTGCCACGCTTACGCAGACGCTTGCGCTGCGTCTGTTGGCTGAAGAGGATGAGCCTGCTGGGTTGGGTTCAGCCACGGCACGTGAGAGacaccaattttttcagttgatgtgtgcACGCTACCGAGAGGTGCAGCGCGTGGTACGTGATCTTACCTGGAGCACAACGGAGTGTGGGGCTGCGGTACCGCTGCGTGTTGAGTTTTTGCCGTGTAATTCTGGTTATTTTACCTGTATCCGTTGCATAGGTTTTTCTGCAGAAACACTGCGCCAATATCTGCTCGAACAGTGCGGAATAGGAACTGTGGCAATTGATGAGCAGCATCTTCGAGTGGCTTTCAGCGCACTTGAGTTACCTGCTATCGAGCGGGTGCTGCGCGCAGTTGTACATACTGCTGTGCAACTGTCTTGCGTTGGTGAGGGCTCACGGTCTTAGTTTtaccgnnnnnnnnnnnnnnnnnnnnnnnnnnnnnnnnnnnnnnnnnnnnnnnnnnnnnnnnnnnnnnnnnnnnnnnnnnnnnnnnnnnnnnnnnnnnnnnnnnnnnnnnnnnnnnnnnnnnnnnnnnnnnnnnnnnnnnnnnnnnnnnnnnnnnnnnnnnnnnnnnnnnaggagaacgaaataatgccaaaacaagtagtcgtttttgctatgtggtgtatcggtggcgtactggtatcgcatcctgtctctgcgaagcagaatgctgtctctcgtgcggaggtgaaagnnnnnnnnnnnnnnnnnnnnnnnnnnnnnnnnnnnnnnnnnnnnnnnnnnnnnnnnnnnnnnnnnnnnnnnnnnnnnnnnnnnnnnnnnnnnnnnnnnnnnnngattgatgggctgccggttgtcgaggtggctaccaaggcgttttatgggtgcgttTCGCTGGTGcgggtgtcgttgcctagttcggtgcgcatgattgggcagcatgcatttgnnnnnnnnnnnnnnnnnnnnnnnnnnnnnnnnnnnnnnnnnnnnnnnnnnnnnnnnnnnnnnnnnnnnnnnnnnnnnnnnnnnnnnnnnnnnnnnnnnnnnnnnnnnnnnnnnnnnnnnnnnnnnnnnnnnnnnnnnnnnnnnnnnnnnnnnnnnnnnnnnnnnnnnnnnnnnnnnnnnnnnnnnnnnnnnnnnnnnnnnnnnnnnnnnnnnnnnnnnnnnnnnnnnnnnnnnnnnnnnnnnnnnnnnnnnnnnnnnnnnnnnnnnnnnnnnnnnnnnnnnnnnnnnnnnnnnnnnnnnnnnnnnnnnnnnnnnnnnnnnnnnnnnnnnnnnnnnnnnnnnnnnnnnnnnnnnnnnnnnnnnnnnnnnnnnnnnnnnnnnnnnnnnnnnnnnnnnnnnnnnnnnnnnnnnnnnnnnnnnnnnnnnnnnnnnnnnnnnnnnnnnnnnnnnnnnnnnnnnnnnnnnnnnnnnnnnnnnnnnnnnnnnnnnnnnnnnnnnnnnnnnnnnnnnnnnnnnnnnnnnnnnnnnnnnnnnnnnnnnnnnnnnnnnnnnnnnnnnnnnnnnnnnnnnnnnnnnnnnnnnnnnnnnnnnnnnnnnnnnnnnnnnnnnnnnnnnnnnnnnnnnnnnnnnnnnnnnnnnnnnnnnnnnnnnnnnnnnnnnnnnnnnnnnnnnnnnnnnnnnnnnnnnnnnnnnnnnnnnnnnnnnnnnnnnnnnnnnnnnnnnnnnnnnnnnnnnnnnnnnnnnnnnnnnnnnnnnnnnnnnnnnnnnnnnnnnnnnnnnnnnnnnnnnnnnnnnnnnnnnnnnnnnnnnnnnnnnnnnnnnnnnnnnnnnnnnnnnnnnnnnnnnnnnnnnnnnnnnnnnnnnnnnnnnnnnnnnnnnnnnnnnnnnnnnnnnnnnnnnnnnnnnnnnnnnnnnnnnnnnnnnnnnnnnnnnnnnnnnnnnnnnnnnnnnnnnnnnnnnnnnnnnnnnnnnnnnnnnnnnnnnnnnnnnnnnnnnnnnnnnnnnnnnnnnnnnnnnnnnnnnnnnnnnnnnnnnnnnnnnnnnnnnnnnnnnnnnnnnnnnnnnnnnnnnnnnnnnnnnnnnnnnnnnnnnnnnnnnnnnnnnnnnnnnnnnnnnnnnnnnnnnnnnnnnnnnnnnnnnnnnnnnnnnnnnnnnnnnnnnnnnnnnnnnnnnnnnnnnnnnnnnnnnnnnnnnnnnnnnnnnnnnnnnnnnnnnnnnnnnnnnnnnnnnnnnnnnnnnnnnnnnnnnnnnnnnnnnnnnnnnnnnnnnnnnnnnnnnnnnnnnnnnnnnnnnnnnnnnnnnnnnnnnnnnnnnnnnnnnnnnnnnnnnnnnnnnnnnnnnnnnnnnnnnnnnnnnnnnnnnnnnnnnnnnnnnnnnnnnnnnnnnnnnnnnnnnnnnnnnnnnnnnnnnnnnnnnnnnnnnnnnnnnnnnnnnnnnnnnnnnnnnnnnnnnnnnnnnnnnnnnnnnnnnnnnnnnnnnnnnnnnnnnnnnnnnnnnnnnnnnnnnnnnnnnnnnnnnnnnnnnnnnnnnnnnnnnnnnnnnnnnnnnnnnnnnnnnnnnnnnnnnnnnnnnnnnnnnnnnnnnnnnnnnnnnnnnnnnnnnnnnnnnnnnnnnnnnnnnnnnnnnnnnnnnnnnnnnnnnnnnnnnnnnnnnnnnnnnnnnnnnnnnnnnnnnnnnnnnnnnnnnnnnnnnnnnnnnnnnnnnnnnnnnnnnnnnnnnnnnnnnnnnnnnnnnnnnnnnnnnnnnnnnnnnnnnnnnnnnnnnnnnnnnnnnnnnnnnnnnnnnnnnnnnnnnnnnnnnnnnnnnnnnnnnnnnnnnnnnnnnnnnnnnnnnnnnnnnnnnnnnnnnnnnnnnnnnnnnnnnnnnnnnnnnnnnnnnnnnnnnnnnnnnnnnnnnnnnnnnnnnnnnnnnnnnnnnnnnnnnnnnnnnnnnnnnnnnnnnnnnnnnnnnnnnnnnnnnnnnnnnnnnnnnnnnnnnnnnnnnnnnnnnnnnnnnnnnnnnnnnnnnnnnnnnnnnnnnnnnnnnnnnnnnnnnnnnnnnnnnnnnnnnnnnnnnnnnnnnnnnnnnnnnnnnnnnnnnnnnnnnnnnnnnnnnnnnnnnnnnnnnnnnnnnnnnnnnnnnnnnnnnnnnnnnnnnnnnnnnnnnnnnnnnnnnnnnnnnnnnnnnnnnnnnnnnnnGTGCCGTGTTCGCCGTGTGGCGGAAGCCACACGGTAGGTTTTTCTGCTCCTGCACGGCAGTCTCTCCCCTTCCCTTTTGAAAAGGGGGTATAGCTCAACTGGCTAGAGCGACGGCTTTGCAGGTCGTAGGTCAGGGGTTCGAGTCCCCTTATCTCCAGGGAAAGCCCACTATTATTCTTTATGTCCCTTTGTTTTGTTTATGGGGTAAGGAGTAGGTGGTAGGTGATTTTTGAGAGTATTAGGGTGGGGTGnnnnnnnnnnnnnnnnnnnnnnnnnnnnnnnnnnnnnnnnnnnnnnnnnnnnnnnnnnnnnnnnnnnnnnnnnnnnnnnnnnnnnnnnnnnnnnnnnnnnnnnnnnnnnnnnnnnnnnnnnnnnnnnnnnnnnnnnnnnnnnnnnnnnnnnnnnnnnnnnnnnnnnnnnnnnnnnnnnnnnnnnnnnnnnnnnnnnnnnnnnnnnnnnnnnnnnnnnnnnnnnnnnnnnnnnnnnnnnnnnnnnnnnnnnnnnnnnnnnnnnnnnnnnnnnnnnnnnnnnnnnnnnnnnnnnnnnnnnnnnnnnnnnnnnnnnnnnnnnnnnnnnnnnnnnnnnnnnnnnnnnnnnnnnnnnnnnnnnnnnnnnnnnnnnnnnnnnnnnnnnnnnnnnnnnnnnnnnnnnnnnnnnnnnnnnnnnnnnnnnnnnnnnnnnnnnnnnnnnnnnnnnnnnnnnnnnnnnnnnnnnnnnnnnnnnnnnnnnnnnnnnnnnnnnnnnnnnnnnnnnnnnnnnnnnnnnnnnnnnnnnnnnnnnnnnnnnnnnnnnnnnnnnnnnnnnnnnnnnnnnnnnnnnnnnnnnnnnnnnnnnnnnnnnnnnnnnnnnnnnnnnnnnnnnnnnnnnnnnnnnnnnnnnnnnnnnnnnnnnnnnnnnnnnnnnnnnnnnnnnnnnnnnnnnnnnnnnnnnnnnnnnnnnnnnnnnnnnnnnnnnnnnnnnnnnnnnnnnnnnnnnnnnnnnnnnnnnnnnnnnnnnnnnnnnnnnnnnnnnnnnnnnnnnnnnnnnnnnnnnnnnnnnnnnnnnnnnnnnnnnnnnnnnnnnnnnnnnnnnnnnnnnnnnnnnnnnnnnnnnnnnnnnnnnnnnnnnnnnnnnnnnnnnnnnnnnnnnnnnnnnnnnnnnnnnnnnnnnnnnnnnnnnnnnnnnnnnnnnnnnnnnnnnnnnnnnnnnnnnnnnnnnnnnnnnnnnnnnnnnnnnnnnnnnnnnnnnnnnnnnnnnnnnnnnnnnnnnnnnnnnnnnnnnnnnnnnnnnnnnnnnnnnnnnnnnnnnnnnnnnnnnnnnnnnnnnnnnnnnnnnnnnnnnnnnnnnnnnnnnnnnnnnnnnnnnnnnnnnnnnnnnnnnnnnnnnnnnnnnnnnnnnnnnnnnnnnnnnnnnnnnnnnnnnnnnnnnnnnnnnnnnnnnnnnnnnnnnnnnnnnnnnnnnnnnnnnnnnnnnnnnnnnnnnnnnnnnnnnnnnnnnnnnnnnnnnnnnnnnnnnnnnnnnnnnnnnnnnnnnnnnnnnnnnnnnnnnnnnnnnnnnnnnnnnnnnnnnnnnnnnnnnnnnnnnnnnnnnnnnnnnnnnnnnnnnnnnnnnnnnnnnnnnnnnnnnnnnnnnnnnnnnnnnnnnnnnnnnnnnnnnnnnnnnnnnnnnnnnnnnnnnnnnnnnnnnnnnnnnnnnnnnnnnnnnnnnnnnnnnnnnnnnnnnnnnnnnnnnnnnnnnnnnnnnnnnnnnnnnnnnnnnnnnnnnnnnnnnnnnnnnnnnnnnnnnnnnnnnnnnnnnnnnnnnnnnnnnnnnnnnnnnnnnnnnnnnnnnnnnnnnnnnnnnnnnnnnnnnnnnnnnnnnnnnnnnnnnnnnnnnnnnnnnnnnnnnnnnnnnnnnnnnnnnnnnnnnnnnnnnnnnnnnnnnnnnnnnnnnnnnnnnnnnnnnnnnnnnnnnnnnnnnnnnnnnnnnnnnnnnnnnnnnnnnnnnnnnnnnnnnnnnnnnnnnnnnnnnnnnnnnnnnnnnnnnnnnnnnnnnnnnnnnnnnnnnnnnnnnnnnnnnnnnnnnnnnnnnnnnnnnnnnnnnnnnnnnnnnnnnnnnnnnnnnnnnnnnnnnnnnnnnnnnnnnnnnnnnnnnnnnnnnnnnnnnnnnnnnnnnnnnnnnnnnnnnnnnnnnnnnnnnnnnnnnnnnnnnnnnnnnnnnnnnnnnnnnnnnnnnnnnnnnnnnnnnnnnnnnnnnnnnnnnnnnnnnnnnnnnnnnnnnnnnnnnnnnnnnnnnnnnnnnnnnnnnnnnnnnnnnnnnnnnnnnnnnnnnnnnnnnnnnnnnnnnnnnnnnnnnnnnnnnnnnnnnnnnnnnnnnnnnnnnnnnnnnnnnnnnnnnnnnnnnnnnnnnnnnnnnnnnnnnnnnnnnnnnnnnnnnnnnnnnnnnnnnnnnnnnnnnnnnnnnnnnnnnnnnnnnnnnnnnnnnnnnnnnnnnnnnnnnnnnnnnnnnnnnnnnnnnnnnnnnnnnnnnnnnnnnnnnnnnnnnnnnnnnnnnnnnnnnnnnnnnnnnnnnnnnnnnnnnnnnnnnnnnnnnnnnnnnnnnnnnnnnnnnnnnnnnnnnnnnnnnnnnnnnnnnnnnnnnnnnnnnnnnnnnnnnnnnnnnnnnnnnnnnnnnnnnnnnnnnnnnnnnnnnnnnnnnnnnnnnnnnnnnnnnnnnnnnnnnnnnnnnnnnnnnnnnnnnnnnnnnnnnnnnnnnnnnnnnnnnnnnnnnnnnnnnnnnnnnnnnnnnnnnnnnnnnnnnnnnnnnnnnnnnnnnnnnnnnnnnnnnnnnnnnnnnnnnnnnnnnnnnnnnnnnnnnnnnnnnnnnnnnnnnnnnnnnnnnnnnnnnnnnnnnnnnnnnnnnnnnnnnnnnnnnnnnnnnnnnnnnnnnnnnnnnnnnnnnnnnnnnnnnnnnnnnnnnnnnnnnnnnnnnnnnnnnnnnnnnnnnnnnnnnnnnnnnnnnnnnnnnnnnnnnnnnnnnnnnnnnnnnnnnnnnnnnnnnnnnnnnnnnnnnnnnnnnnnnnnnnnnnnnnnnnnnnnnnnnnnnnnnnnnnnnnnnnnnnnnnnnnnnnnnnnnnnnnnnnnnnnnnnnnnnnnnnnnnnnnnnnnnnnnnnnnnnnnnnnnnnnnnnnnnnnnnnnnnnnnnnnnnnnnnnnnnnnnnnnnnnnnnnnnnnnnnnnnnnnnnnnnnnnnnnnnnnnnnnnnnnnnnnnnnnnnnnnnnnnnnnnnnnnnnnnnnnnnnnnnnnnnnnnnnnnnnnnnnnnnnnnnnnnnnnnnnnnnnnnnnnnnnnnnnnnnnnnnnnnnnnnnnnnnnnnnnnnnnnnnnnnnnnnnnnnnnnnnnnnnnnnnnnnnnnnnnnnnnnnnnnnnnnnnnnnnnnnnnnnnnnnnnnnnnnnnnnnnnnnnnnnnnnnnnnnnnnnnnnnnnnnnnnnnnnnnnnnnnnnnnnnnnnnnnnnnnnnnnnnnnnnnnnnnnnnnnnnnnnnnnnnnnnnnnnnnnnnnnnnnnnnnnnnnnnnnnnnnnnnnnnnnnnnnnnnnnnnnnnnnnnnngcgcgcgtccgtgctctccacacgaggcggagctccgtccactcacgaaagatatcgtgtccgaagtgcaatACGAGCATCATAACGTAAACTGCACAGGGTGTTGTGTACGCTGTGCTGCGCACATGTAGCACnnnnnnnnnnnnnnnnnnnnnnnnnnnnnnnnnnnnnnnnnnnnnnnnnnnnnnnnnnnnnnnnnnnnnnnnnnnnnnnnnnnnnnnnnnnnnnnnnnnnnnnnnnnnnnnnnnnnnnnnnnnnnnnnnnnnnnnnnnnnnnnnnnnnnnnnnnnnnnnnnnnnnnnnnnngatcttcagagaaaagaaaaaagcgaggtagatactaactacacgtatgtgtgcagccacgcatttcagagaacgtgtgnnnnnnnnnnnnnnnnnnnnnnnnnnnnnnnnnnnnnnnnnnnnnnnnnnnnnnnnnnnnnnnnnnnnnnnnnnnnnnnnnnnnnnnnnnnnnnnnnnnnnnnnnnnnnnnnnnnnnnnnnnnnnnnnnnnnnnnnnnnnnnnnnnnnnnnnnnnnnnnnnnnnnnnnnnnnnnnnnnnnnnnnnnnnnnnnnnnnnnnnnnnnnnnnnnnnnnnnnnnnnnnnnnnnnnnnnnnnnnnnnnnnnnnnnnnnnnnnnnnnnnnnnnnnnnnnnnnnnnnnnnnnnnnnnnnnnnnnnnnnnnnnnnnnnnnnnnnnnnnnnnnnnnnnnnnnnnnnnnnnnnnnnnnnnnnnnnnnnnnnnnnnnnnnnnnnnnnnnnnnnnnnnnnnnnnnnnnnnnnnnnnnnnnnnnnnnnnnnnnnnnnnnnnnnnnnnnnnnnnnnnnnnnnnnnnnnnnnnnnnnnnnnnnnnnnnnnnnnnnnnnnnnnnnnnnnnnnnnnnnnnnnnnnnnnnnnnnnnnnnnnnnnnnnnnnnnnnnnnnnnnnnnnnnnnnnnnnnnnnnnnnnnnnnnnnnnnnnnnnnnnnnnnnnnnnnnnnnnnnnnnnnnnnnnnnnnnnnnnnnnnnnnnnnnnnnnnnnnnnnnnnnnnnnnnnnnnnnnnnnnnnnnnnnnnnnnnnnnnnnnnnnnnnnnnnnnnnnnnnnnnnnnnnnnnnnnnnnnnnnnnnnnnnnnnnnnnnnnnnnnnnnnnnnnnnnnnnnnnnnnnnnnnnnnnnnnnnnnnnnnnnnnnnnnnnnnnnnnnnnnnnnnnnnnnnnnnnnnnnnnnnnnnnnnnnnnnnnnnnnnnnnnnnnnnnnnnnnnnnnnnnnnnnnnnnnnnnnnnnnnnnnnnnnnnnnnnnnnnnnnnnnntacttacattgaccagttcaagcagcggtcctgcgcacgactccacagccgtgtctgccgcgacatctgcgctcatgcgtccacagaagatgcACCGTGCCCTGTGGCGCGTACACACCCGCGACACGCGAATACnnnnnnnnnnnnnnnnnnnnnnnnnnnnnnnnnnnnnnnnnnnnnnnnnnnnnnnnnnnnnnnnnnnnnnnnnnnnnnnnnnnnnnnnnnnnnnnnnnnnAAAGGGAAGgaagactcctagcatgagctcccCGGTCCTGnnnnnnnnnnnnnnnnnnnnnnnnnnnnnnnnnnnnnnnnnnnnnnnnnnnnnnnnnnnnnnnnnnnnnnnnnnnnnnnnnnnnnnnnnnnnnnnnnnnnnnnnnnnnnnnnnnnnnnnnnnnnnnnnnnnnnnnnnnnnnnnnnnnnnnnnnnnnnnnnnnnnnnnnnnnnnnnnnnatccaaggaggaatcctcccgtaggggcaaaaagcgcggtgtatccgccccgacctcctgaaaaaaccggcagaccaaggagtcctgccccgaggaagctgagaacggcgagtgcaccgtctcgcggTCCCAACAATAAACCggtgagaacggccgctgcattctgcagtacaagcggaacaggcttgagaggaatgctaacgagcgcactcgagctaatgagtgcggcaaaaagcgcaacaaaagccaaagacttactacggtgcatgGTACAGTACnnnnnnnnnnnnnnnnnnnnnngtataacacggaaggaggaatctttcaaTATCTTGGGTGGTGCCACAGGTATAGTTTTTAACAGACTTACCCGAACGGCTGCCAGGTGCGTACGCGTCGGTTCGTTCCCACTGTGCGCGGnCAGAGCTCGTGTAGTGTCTATTGACAGATGCAAGGATCGGGTACCGTCATGnnnnnnnnnnnnnnnnnnnnnnnnnnnnnnnncgctGGGGACAGAGAGAGTATCGCACGCTTCGTGCGTGTGGTGCGCGATTGTCTGGATTTGTTTCGCACCGAGGGTATTGGGCCCCGTCCTAGGAATGATTCGGTAATTTTACCGAATGCTGCGTGTTCACCGCGTAATCATGCAGGAAAGCGTGCGCAGAGCACTGCCGATGCGTGTGTGAGAAGCAGTGACGGGTCTGTATACACGGACGAAACCTTGCGCGAGGAAATTTTTGCATGCCGTGCGTGTGAATTGTATCAACGGCGTACACATGCGGTGGTGGGAGAGGGTGTTGCAGACGCAGACGTGCTCGTCGTTGGGGAGGCCCCTGGAGCGGAAGAAGATcgaagcggtCGTCCGTTCGTAGGACGGTCAGGTAAATTGCTGGACGCAATGCTTGCGGCGATTGGACTTTCGCGTCAGCAAAATTGTTATATCACCAATGTGGTTAAGTGCCGGCCGCCAAGGAACCGCACACCAACACCCCACGAGACTGCGTGTTGTGCACGGTTCCTCCATGCGCATCTTACGCTGCATCGCCCGTGTGCTATTTTGGTGCTCGGCCGCTGCGCCGCACAGCACATGCTCCAAAnnnnnnnnnnnnnnnnnnnnnnnnnnnnnnncttttttaccnnnnaggggattccccttctggctacgtaccatccgagtgcgttgttacgggatgaagcgctgaaacgtccggcgngggaggatctcaaaacgtttcgtgcacggttgctgcagttgaagcaggacgcacacatgccaatannnnnnnnnngcgcnnnnnnnnnnnnnnnnnnnnnnnnnnnnnnnnnnnnnnnnnnnnnnnnnnnnTGCGTGTGCTGCCCACGCGGGTGAGGCACTCGTGGGTAGACGGGTTCTTGCTCCCTTTGGGGCGCGTACACTCATTGGATTTGTGATAAGTGAATCACATTCTTCGCCTGCTGATTGCGGTGGTGCAGTTGGCACGTTCAAGGAGATCATCCGCGTCATTGACAGGGAAGCGCTTTTTGACCAAACGCATCTTGCGTGTGCGCGTTGGATGGCGCATTTCTACCTGTGTGCCTTAGGTCAGGCGCTGTGTGCGGTGGTTCCGTCTnGGAAACGAGAACGGACATTGTCTTCTTTTGCTTCTTGTGCGGGTGTTCGGCGCACTGACACCTATGCGCTTTCGGGCGAACAGCGCAAGGCGATTGATGCGATTACCGcgagcaccggtgcgcgcagtttttatgtgcacggggtgacagggtcggggaagacgGAAGTGTTCTTGCGCGCAGCCGAGGCagtccttgcgcgtggcaagtcGGTTATCTATCTTGTTCCTGAGATAGCGCTCACTCACCAGGTGCTCCAGGAGGTATATGTGCGCTTTGGCAGTCAGGCGGCGGTGTTGCACTCAGCGCTCAGTGGCAGTCAGCGCCTAGGTGAGTGGCGGCGCATACAGCGCATGCGTCACTGTGTAGTGAttggagctcggagtgcaatttttgctccgttgaagcggctgggccttgtgataatggatgaagaacatgnnnnnnnnnnnnnnnnnnnnnnnnnnnnnnnnnnnnnnnnnnnnnnnnnnnnnnnnnnnnnnnnnnnnnnnnnnnnnnnnnnnnnnnnnnnnnnnnnnnnnnnnnnnnnnnnnnnnnnnnnnnnnnnnnnnnnnnnnnnnnnnnnnnnnnnnnnnnnnnnnnnnnnnnnnnnnnnnnnnnnnnnnnnnnnnnnnnnnnnnnnnnnnnnnnnnnnnnnnnnnnnnnnnnnnnnnnnnnnnngtctggtggatgaaataCGCAAGACGAAGGAGGCAGGATATCAATCGATGCTCTTTTTGAATCGTCGAGGATTTTCCTATTCGTTTCAGTGTCGCAGCTGTGGATACACGCTGTGTTGCACGCAGTGTGCAGTTCCCTTGACGTGGCACAaacgtgtgggggcaatgcnnnnnnnnnnnnnnnnnnnnnnnnnnnnnnnnnnnnnnnnnnnnnnnnnnnnnnnnnnnnnnnnnnnnnnnnnnnnnnnnnnnnnnnnnnnnnnnnnnnnnnnnnnnnnnnnnnnnnnnnnnnnnnnnnnnnntattgcacgGGTGGACACCGATGCGCTGCGCTCAGGGCACGTGCAGCAGACGATGGAGCAGTTTCGCGCGGGGAAAATCGATGTACTGTTGGGTACGCAAATGATAGCAAAGGGATTTAATTTCCCTACGCTGCGTTTAGTGGGTATTGCCnnnnnnnnnnnnnnnnnnnnnnnnnnnnnnnnnnnnnnnnnnnnnnnnnnnnnnnnnnnnnnnnnnnnnnnnnnnnnnnnnnnnnnnnnnnnnnnnnnnnnnnnnnnnnnnnnnnnnnnnnnnnnnnnnnnnnnnnnnnnnnnnnnnnnnnnnnnnnnnnnnnnnnnnnnnnnnnnnnnnnnnnnnnnnnAACTTGCGCAGCGGGAGGCGCTGTGTTTTCCGCCCTTTGTGCGCCTTATTCGGTTTGTTTTTCGCAGCAAGACGCGGCGCAAGGCTAAAGnnnnnnnnnnnnnnnnnnnnnnnnnnnnnnnnnnnnnnnnGCCTCTGGGtgcggatgtactgggacctgcagcgtgtgtggtggcgcaggtggcaggcagnnnnnnnnnnCAAATACTGCTGCGTGCCCCATCATTCCCAGTGGTGCAGCAGGTGGCGCGCAGCTTTTTAGATGAATTTCGAGCTCCGGCGGGGGTGTACGTAGAATCTGACGTAGATCCTGTAAATGTACTGTAGGGCGAGTAGATGTACTCCGTGTTATCCTGCTGTTTGCGTGTTTGGTTGACCGGTAGTATGCGGTGCCTGGTATAGGTGCGGGACGGAAAGGAGAGAGGATGTGGCACTGCCGATTATTTTTCAGGACGCAGCGGTGGnnnnnnnnnnnnnnnnnnnnggnnnnnnnnnnnnnnnnnnnnnnnnnnnnnnggtgtgcgtagttgacgtattacagaaacagcttggggtgcgtctgtttcctctgcatCGTTTGGACAAGGACACCGCGGGCGTGCTGCTGTTTGCAAAACATGCACGGGCAGCTGCTCTGTACCAGGGGATTTTAGGCAGCATGCGTGTGattaagcgctatcgcgcactttgttttgggcgacctccccgaGAGTGTGGTGATATTCGCGTTCCTATCCGTACCGGTACGGCAGCAAGGCGGCGTCAGGTnnnnnnnnnnnnnnnnnnnnnnnnnnnnnnnnnnnnnnnnnnnnnnnnnnnnnnnnnnnnnnnnnnnnnnnnnnnnnnnnnnnnnnnnnnnnnnnnnnnnnnnnnnnnnnnnnnnnnnnnnnnnnnnnnnnnnnnnnnnnnnnnnnnnnnnnnnnnnnnnnnnnnnnnnnnnnnnnnnnnnnnnnnnnnnnnnnnnnnnnnnnnnnnnnnnnnnnnnnnnnnnnnnnnnnnnnnnnnnnnnnnnnnnnnnnnnnnnnnnnnnnnnntgcctgtacacttcctgcgtgctcttgatgccgttgcgctatgattgcctgtagcaGGGCATTCTGGTAGGCGGTGTGTGGTTTTGAGTTCTGCCGGTAACAGaaagagtgtcgtgtgaatttcaatagttttTCTCTAGGGTGTGTACTGCACTCGTTGTGTTTTTGCAGGCGCGAGGGGAGGGGAGCGGTCCCCTGCTGCTGTACTGTCTGTAGGAAAGATACCGGCGCCTATTGTTATATcgggctatttgtgctagagtgcgcgaaaccgctagtggggatgGCCTATGGGTACTGTTGttccgggattcgatgacgagaaagacgaaagtcttaagatgaatcnnnnnnnnnnnnnnnnnnnnnnnnnnnnnnnnnnnnnnnnnnnnnnnnnnnnnnnnnnnnnnnnnnnnnnnnnnnnnnnnCAAAAGAGGattgcgaaggttatcgatgcaggctacacgcgtattgtatttaactgcgcctctttgaattatgtctcctccactggaattggttcttttacggcgtttctaaaaacggtcaagcctaaagnnnnnnnnnnnnnnnnnnnnnnnnnnnnnnnnnnnnnnnnnnnnnnnnnnnnnnnnnnnnnnnnnnnnnnnnnnnnnnnnnnnnnnnnnnnnnnnnnnnnnnnnnnnnnnnnnnnnnnnnnnnnnnnnnnnnnnnnnnnnnnnnnnnnnnnnnnnnnnnnnnnnnnnnnnnnnnnnngctctaagaagttaaaggcgactAAGCAGGGGCGTTTTCGTTGTTCCGAATGTAAGACGATTCTCGCCCTTGACGCGAGCGCACACGTGTCTctcggnnnnnnnnnnnnnnnnnnnnnnnnnnnnnnnnnnnnnnnnnnnnnnnnnnnnnnnnnnnnnnnnnnnnnnnnnnnnnnnnnnnnnnnnnnnnnnnnnnnnnnnnnnnnnnnnnnnnnnnnnnnnnnnnTGTCTGTGTTGCTTCTGTAGCTCAGTTGGCAGAGCGCAACCATGGTAAGGTTGAGGTCAGCGGTTCAATCCCGCTCGGAAGCTTCCGTCTGTGGATGTGAGGAGGGGTGGTATGGCAAAGAGGACGGCGGTGGAGCTTATTGCGCTTCAGTGCACTGGATGCAAGCGGCGTAATTACACCACTTCAAGAAACCGACGTAACGTTCAGGAAAAGCTCGAGCTCAGGAAGTATTGTCCTTTTGAGCGTAGACGTGTGCTGCATAGAGAGGCGAAGATAAAGTAGGCTGTCGTCAnnnnnnnnnnnnnnnnnnnnnnnnnnnnnnnnnnnnnnnnnnnnnnnnnnnnnnnnnnnnnnnnnnnnnnnnnnnnnnnnnnnnnnnnnnnnnnnnnnnnnnnnnnnnnnnnnnnnnnnnnnnnnnnnnnnnnnnnnnnnnnnnnnnnnnnnnnnnnnnnnnnnnnnnnnnnnnnnnnnnnnnnnnnnnnnnnnnnnnnnnnnnnnnnnnnnnnnnnnnnnnnnnnnnnnnnnnnnnnnnnnnnnnnnnnnnnnnnnnnnnnnnnnnnnnnnnnnnnnnnnnnnnnnnnnnnnnnnnnnnnnnnnnnnnnnnnnnnnnnnnnnnnnnnnnnnnnnnnnnnnnnnnnnnnnnnnnnnnnnnnnnnnnnnnnnnnnnnnnnnnnnnnnnnnnnnnnnnnnnnnnnnnnnnnnnnnnnnnnnnnnnnnnnnnnnnnnnnnnnnnnnnnnnnnnnnnnnnnnnnnnnnnnnnnnnnnnnnnnnnnnnnnnnnnnnnnnnnnnnnnnnnnnnnnnnnnnnnnnnnnnnnnnnnnnnnnnnnnnnnnnnnnnnnnnnnnnnnnnnnnnnnnnnnnnnnnnnnnnnnnnnnnnnnnnnnnnnnnnnnnnnnnnnnnnnnnnnnnnnnnnnnnnnnnnnnnnnnnnnnnnnnnnnnnnnnnnnnnnnnnnnnnnnnnnnnnnnnnnnnnnnnnnnnnnnnnnnnnnnnnnnnnnnnnnnnnnnnnnnnnnnnnnnnnnnnnnnnnnnnnnnnnnnnnnnnnnnnnnnnnnnnnnnnnnnnnnnnnnnnnnnnnnnnnnnnnnnnnnnnnnnnnnnnnnnnnnnnnnnnnnnnnnnnnnnnnnnnnnnnnnnnnnnnnnnnnnnnnnnnngctagtccaggtggaggcgctctgattttcttcttccagggtggagagtgttgcaatgcgcatgattgcctgccgcttacgcgttggtttcgggtgttttgttgttttttacgtcataaggagaggccagtatggcagcgaagaagaaagnnnnnnnnnnnnnnnnnnnnnnnnnnnnnnnnnnnnnnnnnnnnnnnnnnnnnnnnnnnnnnnnnnnnnnnnnnnnnnnnnnnnnnnnnnnnnnnnnnnnnnnnnnnnnnnnnnnnnnnnnnnnnnnnnnnnnnnnnnnnnnnnnnnnnnnnnnnnnnnnnnnnnnnnnnnnnnnnnnnnnnnnnnnnnnnnnnnnnnnnnnnnnnnnnnnnnnnnnnnnnnnnnnnnnnnnnnnnnnnnnnnnnnnnnnnnnnnnnnnnnnnnnnnnnnnnnnnnnnnnnnnnnnnnnnnnnnnnnnnnnnnnnnnnnnnnnnnnnnnnnnnnnnnnnnnnnnnnnnnnnnnnnnnnnnnnnnnnnnnnnnnnnnnnnnnnnnnnnnnnnnnnnnnnnnnnnnnnnnnnnnnnnnnnnnnnnnnnnnnnnnnnnnnnnnnnnnnnnnnnnnnnnnnnnnnnnnnnnnnnnnnnnnnnnnnnnnnnnnnnnnnnnnnnnnnnnnnnnnnnnnnnnnnnnnnnnnnnnnnnnnnnnnnnnnnnnnnnnnnnnnnnnnnnnnnnnnnnnnnnnnnnnnnnnnnnnnnnnnnnnnnnnnnnnnnnnnnnnnnnnnnnnnnnnnnnnnnnnnnnnnnnnnnnnnnnnnnnnnnnnnnnnnnnnnnnnnnnnnnnnnnnnnnnnnnnnnnnnnnnnnnnnnnnnnnnnnnnnnnnnnnnnnnnnnnnnnnnnnnnnnnnnnnnnnnnnnnnnnnnnnnnnnnnnnnnnnnnnnnnnnnnnnnnnnnnnnnnnnnnnnnnnnnnnnnnnnnnnnnnnnnnnnnnnnnnnnnnnnnnnnnnnnnnnnnnnnnnnnnnnnnnnnGGACTTGGGGGCTGCTGTCTGTGAGTTGAAAAAGGGGCGTGTCGAGTTTcgcgcggataagacaggtgtggtccatctagcagtagggaaaacgacgatggactctgcgcagattgtagagaatgttgacgtGTTTCTGTcggagatggatcgCAAGAAGCCCGTTGACGTAAAAGCTGGTTTTGTCCGTTCGATTTcgctcaGCTCCAGTATGGGGCCTGGGATTTGGGTTGTCCATAAGTCAGAGGAGTAGTATGGCAGTACGCGCACGAAGGCtgcagccggcaaaggnnnnnnnnnnnnnnnnnnnnnnnnnnnnnnnnnnnnnnnnnnnnnnnnnnnnnnnnnnnnnnnnnnnnnnnnnnnnnnnnnnnnnnnnnnnnnnnnnnnnnnnnnnnnnnnnnnnnnnnnnnnnnnnnnnnnnnnnnnnnnnnnnnnnnnnnncgaatatcgcctttacgtccctaaacatgacggtgggagagtATCTGGTGGGGCCCACGGCCATCGCCCTAGTGGACACGGAGCATGCGAATGGCGTCGCGCGTGTGCnnnnnnnnnnnnnnnnnnnnnnnnnnnnnnnnnnnnnnnnnnnnnnnnnnnnnnnnnnnnnnnnnnnnnnnnnnnnnnnnnnnnnnnnnnnnnnnnnnnnnnnnnnnnnnnnnnnnnnnnnnnnnnnnnnnnnnnnnnnnnnnnnnnnnnnnnnnnnnnnnnnnnnnnncgtattacaggctgtgatggacaaaagggatgagggtgtagaagtttccgtggtgTCGGGAGGTGATTCGTCCtaggcggttgttgtaaCTTAGTTACGGGGTATGTGTTAGGCGGTCAGGCTTCTGGGGTGCTGTCTTCCTGTCCGTTTATAGGGGTTATTTCGCATACAAGGAGAAGATAATATGGCGGCGTTGAGTAATGAACAGATTATTGAGGCGATTCGGGGCAAGACCATCCTGGAGCTTTCTGAGCTTATCaaggcnnnnnnnnnnnnnnnnnnnnnnnnnnnnnnnnnnnnnnnngcgccggtagcggaaggtggcgggnnnnnnnnnnnnnnnnnnnnnnnnnnnnnnnnnnnnnnnnnnnnnnnnnnnnnnnnnnnnnnnnnnnnnnnnnnnnnnnnnnnnnnnnnnnnnnnnnnnnnnnnnnnnnnnnnnnnnnnnnnnnnnnnnnnnnnnnnnnnnnnnnnnnnnnnnnnnnnnnnnnnnnnnnnnnnnnnnnnnnnnnnnnnnnnnnnnnnnnnnnnnnnnnnnnnnnnnnnnnnnnnnnnnnnnnnnnnnnnnnnnnnnnnnnnnnnnnnnnnnnnnnnnnnnnnnnnnnnnnnnnnnnnnnnnnnnnnnnnnnnnnnnnnnnnnnnnnnnnnnnnnnnnnnnnnnnnnnnnnnnnnnnnnnnnnnnnnnnnnnnnnnnnnnnnnnnnnnnnnnnnnnnnnnnnnnnnnnnnnnnnnnnnnnnnnnnnnnnnnnnnnnnnnnnnnnnnnnnnnnnnnnnnnnnnnnnnnnnnnnnnnnnnnnnnnnnnnnnnnnnnnnnnnnagcttgaggggggagtgcatgtcagcacgagtttgcaaaacacacagagtgtacgtgggaaaggatgtcaggaattttatggacatcccggatctcatcgaaatccagcttcgatcttacgnnnnnnnnnnnnnnnnnnnnnGGAATACACCGTCCGGCGCCGACACCCTTATCTCCGGTACTAGAGAGGAGCTCGGCCTCGAAGACGTGTTCAAGACTACCTTTCCTATCGAGAGCTCTACGGGGGACATGACGCTCGAGTACCAATCATACTCCCTTGATGAGAAAAACATCAAGTTCTCCGaggcggagtgtaaacaaaagggtttgacgtaCGCCATTCCGCTGAAGGCGCTTGTTGATTnACGTTTCAATAATACGGGGGAGATTAGGCGCAAAGACATTTATATgggagatatccccaagatgactgaacgcggcacctttatcatcaacggtgcggaGCGTGTGGTGGTATCCCAGATCnnnnnnnnnnnnnnnnnngtcttttctcatgagaaggacaaggaaggncgggaggtattctccagccgcattattccgtaccggggaagctggcttgaatttgaaattgntcagaaaaaagatctcatcnnnnnnnnnnnnnnnnnnnnnnnnnnnnnnnnnnnnnnnnnnnnnnnnnnnnnnnnnnnnnACGAAACGCGTGAGCAGATCATCGAGGCCTTTTACGCCATAGAAAAGACGCCTGTTTGTCAGGATCGTGTGGAGTACnnnnnnnnnnnnGGTAAGATCCTAGCACGATCGGTGACGGTGGAAAATGAGCAGGGTGAAACCCGGGtgttgtacaaagcaggagagaaaatccatcccnnnnnnnnnnnnnnnnnnnnnnnnnnnnnnnnnnnnnnnnnnnnnnnnnnnnnnnnnnnnnnnnnnnnnnnnnnnnnnnnnnnnnnnnnnnnnnnnnnnnnnnnnnnnnnngaaATGAAGTTCTCTAAGTCGGGTGCACAGGACGAGCTTTCGCGTGAAGAGGCACTGTGTATTGTATACTCAGCGCTAAGACCAAGCGATCCTATGACCATGGACGCGGCGGAAAAAGATTTGCAGACAATGTTTTTCTCCCCACGTCGCTATGATTTAGGGCGGGTGGGGCGTTACAAGCTGAACAAGAAATTTCGCTCTGACTCGCCGACTACTGAGTGCACGCTCACCCTCGATGATATCGTAAATACnnnnnaatttctcatcagaatgtatagcggtgatgcacaggaagatgatatcgatcacctgggcaaccgtcgtnnnnnnnnnnnnnnnnnnnnnnnnnnnnnnnnnnnnnnnnnnnnnnnnnnnnnnnnnnnnngtattgcgaaggagCGTATGAGttctaaggaaacgGAAACGATCAAGCCGCAGGATCTCATTTCCATAAAACCTATCATGGCTGCGATTAAGGAGTTCTTTGGTGCAAGTCAGCTTTCTCAGTTCATGGATCAGGTCAATCCGCTGGCGGAGTTGACACACaagcnnnnnnnnnnnnnACTTGGTCctggtggactttcaagggagcgtgctgggtttgaggtacgcgatgtgcactacacgcactacggtcggatgnnnnnnnnnnnnnnnnnnnnnnnnnnnnnnnnnnnnnnnnnnnnnnnnnnnnnnnnnnnnnnnnnnnnnnnnnnnnnnnnnnnnnnnnnnnnnnnnnnnnnnnnnnnnnnnnnnnnnnnnnnnnnnnnnnnnnnnnnnnnnnnnnnnnnnnnnnnnnnnnnnnnnnnnnnnnnnnnnnnnnnnnnnnnnnnnnnnnnnnnnnnnnnggtcatccgtgtagatcatgtctcttgtcggcaccggggggattnnnnnnnnnnnnnnnnnnnnnnnnnnnnnnnnnnnnnnnnnnnnnnnnnnnnnnnnnnnnnnnnnnnnnnnnctctcataccgtttcttgagcatgatgatgctaacnnnnnnnnnnnnnnnnnnnnnnnnnnnnnnnAGGGAGTGCCGCTTATTTTTCCTGAACCCCCGCGCGTGGGTACAGGCATGGAAGAGAAGTGTGCATATGACTCTGGAGTGCTGGTGAAGGCAAAGCAAGACGGAACGGTTGcctacgtttcctcagagaagatagtggtttgttccgccgcggcgtctggggaagagcagnnnnnnnnnnnnnnnnnnnnnnnnnnnnnnnnnnnnnnnnnggatacctgttaccaccagcggccaatagtgcacgtgggagatcgggtacaggtagGAnnnnnnnnnnnnnnnnnnnnnnnnnnnnatcgaggggagcttgcgcttggcagaaacattctagttggttttgtgccgtggaacGGTTACAACTACGAGGATGCCATTTTGATTTCTCACCGGGTGGTAAAGGAGGATATGTTCACCTCGGTTCACATCAAAGAATTTTCTACTGAGGTGCGTGAAACCAAGCTGGGTTCTGAACGAATGACGAATGATATCCCGAATAAGTCTGAGAAGAATCTGGATAATTTGGATGCAGAGGGGATCATTCGTATTGGGTCAAAGGTGCGTGCGGGAGACGTGCTTATCGGAAAGATTACGCCAAAAAGCGAGTCTGAGACGACGCCAGAGTTTAGGCTGCTGAATTCTATTTTTGGGGAGAAGGCGAAGGAAGTGCGTGATTCTTCTCTACGTGTGCCGCATGGAGTTGAGGGTACAGTCATTGACGTGCAGCGACTCAGGCGTTCGGAGGGAGATGATTTAAACCCCGGGGTGTCAGAGGTGGTGAAGGTTCTTATCGCTACCAAGCGTAAGCTGCGTGAAGGGGATAAAATGGCCGGTCGCCACGGTAACAAGGGTATCGTTGCGCGCATCCTTCCTGAAGAAGACATGCCGTATCTGGATGATGGTACCCCGCTTGATGTCTGTTTGAACCCGCTCGGTGTACCTTCTCGTATGAACnnnnnnnnnnnnnnnnnnnnnnnnnnnnnnnnnnnnnnnnnnnnnnnnnnnnaatggtatgagtctcctgtctttcnnnnnnnnnnnnnnnnnnnnnnnnnnnnnnnnnnnnnnnnnnnnnnnnnnnnnnnnnATTCAAAAGtGATGCTGCGTGACGGACGCACGGGGGATTATTTTCAAAACCCTGTATTTGTGGGGGTTATTTACTTTATGAAGCTTGCGCATCTAGTGGAtgacaaaatgcacgcccgctctacaggtccatattcgcttgtgacgcagcaacccttagggggtaaagcgcagtttggagggcagcgtctcggggaaatggaggtgtgggcgcnnnnnnnnnnnnnnnnnnngaataccctgcaggagttgctaacgattaaatcggatgatatgcacgggcGTTCTAAAATTTATGAGGCAATTGTAAAAGGGGAGGCTTCGTCTCCTACCGGTATTCCTGAATCTTTTAACGTGTTGGTGCAGGAGCTGCGGGGACTTGCGctcgactttncgatttacgatgcgaagggcaagcagattccgctcactgagcgcgatgaagaaannnnnnnnnnnnnnnnnnnnnnnnnnnnnnnnnnnnnnnnnnnnnnnnnnnnnnnnnnnnnnnnnnnnnnnnnnnnnnnnnnnnnnnnnnnnnnnnnnnnnnnnnnnnnnnnnnnnnnnnnnnnnnnnnnnnnnnnnnnnnnnnnnnnnnnnnnnnnnnnnnnnnnnnnnnnnnnnnnnnnnnnnnnnnnnnnnnnnnnnnnnnnnnnnnnnnnnnnnnnnnnnnnnnnnnnnnnnnnnnnnnnnnnnnnnnnnnnnnnnnnnnnnnnnnnnnnnnnnnnnnnnnnnnnnnnnnnnnnnnnnnnnnnngagcgcatggggcatattgagcttgcaacgcctgtttctcatatttggtactaccgttgtgtaccannnnnnnnnnnnnnGTTACTCGATCTACAGGTGATCGCACTGCGTTCTGTTTTGTACTATGAGAAGTACATAGTTATAGAGCCGGGCGACACCGATTTAAAAAAGAATCAGTTGCTCACTGAAACTGAGTACAATgacgcgcaggaacgctacggtggcggctttacggcgggaatgggnnnnnnnnnnnnnnnnnnnnnnnnnnnnnnnnnnnnnnnnnnnnnnnnnnnnnnnnnnnnnnnnnnnnnnnnnnnnnnnnnnnnnnnnnnnnnnnnnnnnnnnnnnnnnnnnnnnnnnnnnnnnnnnnnnnnnnnnnnnnnnnnnnnnnnnnnnnnnnnnnnnnnnnnnnnnnnnnnnnnnnnnnnnnnnnnnnnnnnnnnnnnnnnnnnnnnnnnnnnnnnnnnnnnnnnnnnnnnnnnnnnnnnnnnnnnnnnnnnnnnnnnnnnnnnnnnnnnnnnnnnnnnnnnnnnnnnnnnnnnnnnnnnnnnnnnnnnnnnnnnnnnnnnnnnnnnnnnnnnnnnnnnnnnnnnnnnnnnnnnnnnnnnnnnnnnnnnnnnnnnnnnnnnnnnnnnnnnnnnnnnnnnnnnnnnnnnnnnnnnnnnnnnnnnnnnnnnnnnnnnnnnnnnnnnnnnnnnnnnnnnnnnnnnnnnnnnnnnnnnnnnnnnnnnnnnnnnnnnnnattccgggcgttcggttatcgtagtggggcctgaacttaagttgtggcagtgcgggttgcctACAAAAATGGCGCTTGAGCTGTTTAAGCCctttattatgaaaaagctggttgagaaagaaattgtctcgaacatcaaaaaggcaaagatgctcgtggnnnnnnnnnnnnnnnnnnnnnnnnnnnnnnnnnnnnnnnnnnnnnnnnnnnnnnCAGTTATGCTTAATCGGGCGCCGACATTGCATCGATTGGGCATTCAGGCTTTTGAGCCGGTGTTGGTGGAGGGGAAGGCGATTCGTCTTCATCCGCTTGTGTGTAAACCTTTTAATGCTGATTTTGATGGGGATCAAATGGCGGTGCATGTGCCGCTGACGCAGGCGGCACAGATGGAGTGTTGGACnnnnnnnnnnnnnnnnnnnnnnnnnnnnnnnnnnnnnnnnnnnnnnnnnnnnnnnnnnnnnnnnnnnnnnnnnnnnnnnnnnnnnnnnnnnnnnnnnnnnnnnnnnnnnnnnnnnnnnngtgctcgtcctcgccgtttttcctcggtggaggaggtaatgatggnnnnnnnnnnnnnnnnnnnnnnnnnnnnnnnnnnnnnnnnnnnnnnnnnnnnnnnnnnnnnnnnnnnnnnnnnnnnnnnnnnnnnnnnnnnnnnnnnnnnnnnnnnnnnnnnnnnnnnnnnnnnnnnnnnnnnnnnnnnnnnnnnnnnnnnnnnnnnnnnnnnnnnnnnnnnnnnnnnnnnnnnnnnnnnnnnnnnnnnnnnnnnnnnnnnnnnnnnnnnnnnnnnnnnnnnnnnnnnnnnnnnnnnnnnnnnnnnnnnnnnnnnnnnnnnnnnnnnnnnnnnnnnnnnnnTCGTGCCTGAGCAGAAGGTGCAGATGCTCGAAAAGGCGAACAAGGAAGTGCTAGCGATTGCGAGTCAATACCGCGGGGGGCACATCAnnnnnnnnnnnnnnnnnnnnnnnnnnnnnGAGGTGTGGTCTAAAACAAGTGAGGAGCTCACTTCGCTCATGATGGAAACACTTGAGCGCGACAAGGATGGATTTAATACCATTTACATGATGGCTACCTCAGGTGnnnnnnnnnnnnnnnnnnaaatccgccaactggcgggaatgcgtggcttaatggcaaagccgagtggggatatcannnnnnnnnnnnnnnnnnnnnnnnnnnnnnnnnnnnnnnnnnnnnnnnnnnnnnnnnnnnnnnnnnnnnnnnnnnnnnnnnnnnnnnnnnnnnnnnnnnnnnnnnnnnnnnnnnnnnnnnnnnnnnnnnntcgtctggttgatatcgcgcaagatgtggtggtgaacgaggaggactgtggtaccatcaatggcattgaatatcgcgcggtgaagtccggcgatgagattattgaatcgcttgctgagcgcatcgtaggaaagtatacnnnnnnnnnnnnnnnnnnnnnnnnnnnnnnnnnnnnnnnnnnnnnnnnnnnnnnnnnnnnnnnnnnnnnnnnnnnnnnnnnnnnnnnnnnnnnnnnnnnnnnnnnnnnnnnnnnnnnnnnnnnnnnnnnnnnnnnnnnnnnnnnnnnnnnnnnnnnnnnnnnnnnnnnnnnnnnnnnnnnnnnnnnnnnnnnnnnnnnnnnnnnnnnnnnnnnnnnnnnnnnnnnnnnnnnnnnnnnnnnnnnnnnnnnnnnnnnnnnnnnnnnnnnnnnnnnnnnnnnnnnnnnnnnnnnnnnnnnnactacggaagagaaccgcatcacgtttaagtatcccatactggtaaagagtattgagggggtgcatgtgaAAATGGAGGATGGCTCTCAGCTGTTCACGCGTCGGGGGACGCTCTTTTTTCACAAAACTCTGGCAGAGTATCAGCTTCAAGagggtgacagcgtgcaggtgcgtgaccgcgcgcgggtgctaaaggatgaggttctctaccacnnnnnnnnnnnnnnnnnnnnnnnnnnnnnnnnnnnnnnnnnnnnnnnnnnnnnnnnnnnnnnnnnnnnnnnnnnnnnnnnnnnnnnnnnnnnnnnnnnnnnnnnnnnnnnnnnngtagtaatcaaggcagacgagtatgtgccgcccggaaagaccgtggctacgtttgatcnnnnnnnnnnnnnnnnnnnnnnngagcaggatggctttgtgcggtacgaagatattattttgggctctacgctcatcgaagaggtaaatactgaaacggggatggtggagcgcaggattacgacgttgaaaacaggaatacagcttcaaccgnnnnnnnnnnnnnnnnnnnnnnnnnnnnnnnnnnnnnnnnnnnnnnnnnnnnnnnnnnnnnnnnnnnnnnnnnnnnnnnnnnnnnnnnnnnnnnnnnnnnnnnnnnnnnnnnnnnnnnnnnnnnnnnnnnnnnnnnnnnnnnnggatattncgggggggctgccgcgtgtttctgaattatttgaagcgcggcgccCTAAGAATGCGGCTGTCTTGGCACAGATTTCTGGGGTTGTGTCGTTCAAAGGACTGTTTAAGGGTAAGCGTATTGTCGTGGTGCGTGACCATTACGGGAAGGAATATAAGCACCTCGTGTCCATGTCGCGTCAGCTTTTAGTACGTGATGGAGATACGGTTGAGGCAGGCGAACGCTTGTGTGATGGTTGCTTTGATCCCCATGATATCCTGGCAATTCTGGGTGAAAATGCTTTGCAAAACTATTTGATGAATGAGATCCGTGACGTGTATCGTGTGCAGGGTGTTTCAATnnnnnnnnnnnnnnnnnnnnnnnnnnnnnnnnnnnnnnnnnnnnnnnnnnnnnnnnnctcggttggggacacgcgttttatctacgggCAACAGGTGGATAAGTACCGTTTTCACGAAGAGAACCGTCGGGTTGAAGCGGAaggggggcagcctGCGGTTGCGCGCCCAATGTTCCAGGGTATAACGAAGGCGGCGTTGAACATAGACTCTTTCATATCTGCGGCATCTTTCCAAGAAACGAACAAGGTGCTCACCAATGCGGCGATTGCAGGCTCTGTTGATGACTTGTGTGGGTTGAAGGAGAACGTCATTATAGGGCACTTAATTCCCGCAGGTACGGGGATGCGGCGTTATCGTCAGGTGAAGCTGTTTGACAAGAACAAGCGGGATCTTGATGTGCAGATGGAGGAAGTTATCAGGCGTAGAAAACTTGAAGAGGAGGCGCTTGCCCAGGCAGTTGCGGGTATGGAAGGGGAACCTGAAGGCGAAGCGnnnnnnnnnnnnnnnnnnnnnnnnnnnnnnnnnnnnnnnnnnnnnnnnnnnnnnnnnnnnnnnnnnnnnnnnnnnnnnnnnnnnnnnnnnnnnnnnnnnnnnnnnnnnnnnnnnnnnnnnnnnnnnnnnnnnnnnnnnnnnnnnnnnnnnnnnnnnnnnnnnnnnnnnnnnnnnnnnnnnnnnnnnnnnnnnnnnnnnnnnnnnnnnnnnnnnnnnnnnnnnnnnnnnnnnnnnnnnnnnnnnnnnnnnnnnnnnnnnnnnnnnnnnnnnnnnnnnnnnnnnnnnnnnnnnnnnnnnnnnnnnnnnnnnnnnnnnnnnnnnnnnnnnnnnnnnnnnnnnnnngtgctgattcgcggtggacgtgtgaaagatttacctggagtacgttatcatattatccggggggccaaggacactcttggcgtggtggatcgtaagcgcggtcgttcAAAGTACGGGGCTAAGCgcncncgggcgtaggggcnggggagagnnnnnnnnnnnnnnnnnnnnnnnnnnnnnnnnnnnnnnnnnnnnnnnnnnnnnnnnnnnnnnnnnAGTGTGGTGTTGGCGAAGTTTATTTGTCGAATGATGCTGGCGGGTAAGAAGGCAACTGCGGTGGGTATTATGTacgattgtcttgaacgtaTTCAGCAAAGGACTGGTGAGGAGCCTCTTCCGGTGTTCACAAAAGCGTTAGAGAACGTAAAGCCTGCAGTGGAGGTTAAATCGCGGCGGGTTGGTGGTTCTACCTATCAGGTGCCGATGGAAATTCGGGAAACGAGGCGTGAGGCTTTAGGTATGCGCTGGATTATCGGTGCAGCACGCAGGCGCAGCGGGCGTGGCATGTCGGAGCGACTTGCAGCAGAGATCCTTGATGCGTACCACAGCACGGGAACTGCCTTTAAACGTAAAGAGGATACGCACCGCATGGCAGAGGCCAATAAGGCTTTTTCGCACTATCGCTGGTAGATACGCGTCTCTTCCTGGGGnnnnnnnnnnnnnnnnnnnGTCTGCCCTTGGCAGGGGGTGTTTTTGCCCTCGTCCTTTCTCTTGATTCATCTGGACGTCGGTTTTGGGTGGCGTGCTCTTGTGCGCCTTATCAGCATAAACGGAGGGTCCATACGGTGGGGGGGCTACTCTCGGATCCACATAATTTTGCGCGCGCGTGTGCCCTCTTTCGTGAATTTTCCGCAAGGGAAGAGCGCTCGGGGGTGGTTCGCGCAGAGCTCAAGTGCCCTGTTGACGTTCGTGATTCGTTCTTGCGTGCGTCTAGGGagatagatcCCCGCGCTGCGCATAGCCTTCAGCAAGAGGAAGTGCTCGGCGATGGGCGCGCCGCGCTGTGCAGTGCAGATTGCCTGTTGTTACAGGCATTTACGCACTTCCATAATACGGAACTTGCACGGTATCGGGCACGTTTGGCAGATATTGACGCGCGTATTGTGTATGAGCGCTTTTTGCGCGACTGGGAGTGTGCAGTCGCCTGTGCCCACTTCGGTATCCATAGGcggagtacggtttttaccnnnnnnnnnnnnnnnnnnnnnnnnnnnnnnnnnnnnnnnnnnnnnnnnnnnnnnnnnnnnnnnnnnnnnnnnnnnnnnnnnnnnnnnnctGCTCAATGGCTTGGGGAAGTTGAACGCGTCAGCTCGGGGGTGTTTGCGCAAACCCCCTCTGTTAGTGCTGCACTTGAGAGGATAGCGACAAGCCTTGCCCGCGGGGATGCGGTGCTACTCCGCGGGCATCTCGGCACGGGAAAGACTGAnnnnnnnnnnnnnnnnnnnnnnnnnnnnnnnnnnnnnnnnnnnnnnnnnnnnnnnnnnnnnnnnnnnnnnnnngagtggtgcactgctgcaaccggtggctcgtgtgcggagattcggggcagcgagcgtggcgtgtcagatgccttacaaaactCCCAGGAAGCGCGTACGCAGTTTCAAcgcCTTTTTTCTCAGCGCGCGGCATATTATGAACAGGCGCTACgtctcggtgacgagcggcttttgcagcgcatcaaacccctatttatttcggggtcaaagcacatcgacacagaggatcTGTTtcttgaaaaGACGCTTGTGTTAAAAAACAgtctggagggaaaaacacccgaacactgttaccaggatgtgcatgCCTTTACTGAGCGTGGTTACGCATTTGATCCAATGCTGGATATCTATCTTGCAAAGTATGGCAATTTTGGCACTGAGGTACAAAAGattgagcgtgaggtgctgcgcgcagtaaaagagggacgacctcttgttatcgatgaaatcaacgcnnnnnnnnnnnnnnnnnnnnnnnnnnnnnnnnnnnnnnnnnnnnnnnnnnnnnnnnnnnnnnnnnnnnnnnnnnnnnnnnnnnnnnnnnnnnnnnnnnnnnnnnnnnnnnnnnnnnnnnnnnnnnnnnnnnnnnnnnnnnnnnnnnnnnnnnnnnnnnnnGGGGCGCTCAACCCCGCGTTTGCTTCGCGCTTTAACACGTTCGAATATGATTACCCTCCGCAGTCCACAGAAGGTCTTTATACGAATCAGGCGCATCCTGAGAAAAATGAACTTTTTCGCATCATTCTCGCGcgtcttgcctcttctggaggatcactttgcctgccggatattgatggttcattggacaagctgtttaaacttnnnnnnnnnnnnnnnnnnnnnnnnnnnnnnnnnnnnnnnnnnnnnnnnnnnnnnnnnnnnnnnnnnnnnnnnnnnnnnnnnnnnnnnnnnnnnnnnnnnnnnnnnnnnnnnnnnnnnnnnnnnnnnnnnnnnnnnnnnnnnnngcttgatgagtggaatcggggcgaagagaaggatgtggatcaggcgctgtgggacggctttatcggtggtgttttgaatgcagacgatcaaaattatattttatcgcaagcagtccgatttggtttttttcannnnnnnnnnnnnnnnnnnnnnnnnnnnnnnnnnnnnnnnnnnnnnnnnnnnnnnnnnnnnnnnnnnnntacccgtccttacgaatacacgcgtctgccggatggggtaaaaactctcgctgaggtggtggtattgctctttggacacggacctacgtnnnnnnnnnnnnnnnnnnnnnnngtgccggatgtggtagatGCCGGTGCGATGTTGGTTCTGCACGATCGGATACACGAGTTGGTACGCGTGTGTGCAGCATTCAGACTTTTGCAGGAGAAATCATACGCACGTGAGTGTGCGGATCGGATTGCCCATCTGCAGTGTTCGTGTGACCAACTGGTGTCTGAGCACGCGCGATTGCGCAGTGAGGGAGGAACTCCGCGCGCACTGTGGGatgannnnnnnnnnnnnnnnnnnnnnnnnnnnnnnnnnnnnnnnnnnnnnnnnnnnnnnnnnnnnnnntgctttcccgaTATCTGTGGAGATGAGTGCACGTACAGTGACGTGTTTTTTGAATTTGGAAAAAGCATCACCGCAGCGATTCCTTTGGCGGAAGATTCTGTCCTTATCGCAGGTGGCACAGATGATATACGGCTTTTATACcGGGTGCACGatcaatggcttgttGCGGAATCCACGCATAAGAGCGTGAAAACGGATTGGCGTATTTGTGCGCTTATCTCTATGGGTGATGGAGAAGTAGTTGTCGTTACCGAAGGTGGTGAAACGATCGTGCTTTCTATTCAGGAACGATCgaagaaggcagagagaagaagggnnnnnnnnnnnnnnnnnnnnnnnnnnnnnnnnnnnnnnnnnnnnnnnnnnnnnnnnnnnnnnnnnnnnnnnnnnnnnnnnnnnnnnnnnnnnnnnnnnnnnnnnCTCTTCATTTTAGAAAAGAGTGCACACGGATATGAGTATCGTGGGCCGATTCTTGGTTTAGGTGGGGGAATACGGGTAGTACGGGCTCTTAATACTGCGCAGCTTGTGGTnnnnnnnnnnnnnnnnnnnnnnnnnnnnnnnnnnnatgaagnnnnnnnnnnnnnnnnnnnnnnnnnnnnnnnTCCATACACTGAAGCGGTCGTGCTTTCTGAGGATCGAGTGGTAGTCTTTGGCGATGACGGGGAAAGTCGCGTGCTTCGGCACGAGCATGGCATGGCTGCGGGGGACTATGCGTATGGCGAATGGATTACAGGTTTCAAAAGTGTAGTGTTATGCGCGTTGGCGCTCTCTTCACGCATGGTGCTTGTTTTCGGCCGGGGTGGGGAGGCGCGTTTGCTGTCATTCGATGCTACGgnnnnnnnnnnnnnnnnnnnctcatttggaaacacaacaggagcggtcgtttctgcattcttattgcagagtgaacacccgtcgGTATTCAACGTGTTTATTGCGGCAGAAAATGGTACGTGCAGGCTTTTGACGGTGCACGAATTTTGTGAACCTTCTATGTTTAAAGAGAGACtgagtaagnnnnnnnnnnnnnnnnnnnnnnnnnnnnnnnnnnnnnnnnnnnnnnnnnnnnnnnnnnnnnnnnnnnnnnnnnnnnnnnnnnnnnnnnnnnnnnnnnnnnnnnnnnnnnnnnnnnnnnnnnnnnnnnnnnnnnnnnnnnnnnnnnnnnnnnnnnnnnnnnnnnnnnnnnnnnnnnnnnnnnnnnnnnnnnnnnnnnnnnnnnnnnnnnnnnnnnnnnnnnnnnnnnnnnnnnnnnnnnnnnnnnnnnnnnnnnnnnnnnnnnnnnnnnnnnnnnnnnnnnnnnnnnnnnnnnnnnnnnnnnnnnnnnnnnnnnnnnnnnnnnnnnnnnnnnnnnnnnnnnnnnnnnnnnnnnnnnnnnnnnnnnnnnnnnnnnnnnnnnnnnnnnnnnnnnnnnnnnnnnnnnnnnnnnnnnnnnnnnnnnnnnnnnnnnnnnnnnnnnnnnnnnnnnnnnnnnnnnnnnnnnnnnnnnnnnnnnnnnnnnnnnnnnnnnnnnnnnnnnnnnnnnnnnnnnnnnnnnnnnnnnnnnnnnnnnnnnnnnnnnnnnnnnnnnnnnnnnnnnnnnnnnnnnnnnnnnnnnnnnnnnnnnnnnnnnnnnnnnnnnnnnncgcttgcagagcgcgtgcTGTTCCATGATATGCCAAAGGGTTGCAATCGCGCATGCATCGCACGGGATCCTGTAGTACAGGAGGTGCTACGCACTTTAGTGCTTGGTACTGAGATGAGTAATTTTATTCACTCAGAATTTTTCCCCCTTTCTGGCGAGGTAGTCCCAACCAAGCACCGGGACCGTATTGTCTCTGTGTTTCTCTTAGAGCATTTTGTGCGGCtttggcatctgagtgtggcaaatatcacattcnnnnnnnnnnnnnnnnnnnnnnnnnnnnnnnnnnnnnnnnnnnnnnnnnnnnnnnnnnnnnnnnnnnnnnnnnnnnnnnnnnnnnnnnnnnnnnnnnnnnnnnnnnnnnnnnnnnnnnnnnnnnnnnnnnnnnnnnnnnnnnnnnnnnnnnnnnnnnnnnnnnnnnnnnnnnnnnnnnnnnnnnnnnnnnnnnnnnnnnnnnnnnnnnnnnnnnnnnnnnnnnnnnnnnnnnnnnnnnnnnnnnnnnnnnnnnnnnnnnnnnnnnnnnnnnnnnnnnnnnnnnnnnnnnnnnnnnnnnnnnnnnnnnnnnnnnnnnnnnnnnnnnnnnnnnnnnnnnnnnnnnnnnnnnnnnnnnnnnnnnnnnnnnnnnnnnnnnnnnnnnnnnnnnnnnnnnnnnnnnnnnnnnnnnnnnnnnnnnnnnnnnnnnnnnnnnnnnnnnnnnnnnnnnnnnnnnnnnnnnnnnnnnnnnnnnnnnnnnnnnnnnnnnnnnnnnnnnnnnnnnnnnnnnnnnnnnnnnnnnnnnnnnnnnnnnnnnnnnnnnnnnnnnnnnnnnnnnnnnnnnnnnnnnnnnnnnnnnnnnnnnnnnnnnnnnnnnnnnnnnnnnnnnnnnnnnnnnnnnnnnnnnnnnnnnnnnnnnnnnnnnnnnnnnnnnnnnnnnnnnnnnnnnnnnnnnnnnnnnnnnnnnnnnnnnnnnnnnnnnnnnnnnnnnnnnnnnnnnnnnnnnnnnnnnnnnnnnnnnnnnnnnnnnnnnnnnnnnnnnnnnnnnnnnnnnnnnnnnnnnGGCGTGCACGTGTGTCGTCTGATACATCTTTTGTTAAGGTTGTGTTGGTAATTACTGACGGATgCTCCTCATACCCTCATGAGAGTCGACGAACGATAGAGGAACTGCGTCGGCGTGGAGTGATGATTTTTGGGTTCCAAATTGGATTGAtnnnnnnnnnnnnnnnnnnnnnnnnnnnnnnnnnnnnGAATAGCACTGAGGAGTTAGGACTATTGTTAGGGGAGCGGTTGGAATATCTACCGCGTGAGCTTTTGCGTACnnnnnnnnnnnnnnnnnnnnnnnnnCACCGGTTGTCATCTGGTGAGAGATTGCATGCGCAAAGGGTATGGTagtgtggtgcgtggacgtatgtgcttannnnnnnnnnnnnnnnnnnnnnnnnnnnnnnnnnnnnnnnnnnnnnnnnnnnnnnnnnnnnnnnnnnnnnnngtttttgcgnnnnnnnnnnnnnnnnnnnnnnnnnnnnnnngcacTGTGTCTTTCTGCTGATGAAGCGTTTGTGTCTGCGATGAAGCTTGCGCGTACGTTGAATGTGTCGGTTAGTTGGAATCCGCTTTCGCAAACAATTTTATTTTCCAAAggtgagcgacgagtacaatgtcgggttgggcAAGTGCTTGcactattggatggtagtgaggtgctgatgattgatccgccggtggtaaaggacgcggtggtgtatgtaagtggaGGGTTTGCAGAGCGGCTAAGGAAAGTATTTGGGTATTCTTCACATCAACCTGAGCATCGTGTGGGTGCGGTGCTCATAGACCCAGGACATGGAGGAAAGGATTGGGGGACGAAGGGCTCGTACCGTGAGCAGGGTAAAACGgtggtggtgaaaGAAAAAGACATTGCGTTGCGTGCATCTCaaaatatctatgnnnnnnnnnnnnnnnnnnnnnnnnnnnnnnnnnnnnnnnnnnnnnnnnnnnnnnnnnnnnnnnnnnnnCTTGAGGAGCGTGTGGCGATGGCAAATGGGGTAAAGCTAGGGAagtacgaagcgatcctgtacgtgtcnnnnnnnnnnnnnnnnnnnnnnnnnnnnnnnnnnnnnnnnnnnnnnnnnnnnnnnnnnnnnnnnnnnnnnnnnnnnnnnnnnnnnnnnnnnnnnnnnnnnnnnnnnnnnnnnnnnnnnnnnnnnnnnnnnnnnnnnnnnnnnnnnnnnnnnnnnnnnnnnnnnnnnnnnnnnnnnnnnnnnnnnnnnnnnnnnnnnnnnnnnnnnnnnnnnnnnnnnnnnnnnnnnnnnnnnnnnnnnnnnnnnnnnnnnnnnnnnnnnnnnnnnnnnnnnnnnnnnnnnnnnnnnnnnnnnnnnnnnnnnnnnnnnnnnnnnnnnnnnnnnnnnnnnnnnnnnnnnnnnnnnnnnnnnnnnnnnnnnnnnnnnnnnnnnnnnnnnnnnnnnnnnnnnnnnnnnnnnnnnnnnnnnnnnnnnnnnnnnnnnngaCCCTTATGATAGGggatggggcagcattGTACGGTGTAGTGAGGGCGGTGTGTTGGACTCTCTTGCTAATAATTGGTGTGGCACTTGCGGTGCAGCAATTGGCGGGGGAGCGGTGTATTCGGTAnGTGCTTTTCTTTGAGCGGATGCGTGATGGGACAGTGGTGTGTGAGCCGCGCTATGTGCGTGGGCACACGCAAGGGGGTGCTGTGCGCGAGGTAGTGCGCGAATTGTTGTTAGGGCCGCAGCATCATGGTTACGCACGGTTGGTGGAtccagcggtgagaccgctgagttgtttttctcgtggcGATACGTTGTATATAGATTTGCCTGTAGGGGTGCTGTCACCGAAGTATCGGACGTGTAGtctgcacnnnnnnnnnnnnnnnnnnnnnnnnnnnnnnnnnnnnnnnnnnnnnnnnnnnnnnnnnnnnnnnnnnnnnnnnnnnnnncgcgcggggtttgaaagtgatgggtagatgcaggggtgttgacaaaataggcggtgagcgtatactcgaagagtggttatcttattgtgcgaaaggagcgtttgaatgaagaaagcggttgngttgagcgcggtggcgctgctctcgggnnnnnnnnnnnnnnnnnnnnnnnnnnnnnnnnnnnnnnnnnnnnnnnnnnnnnnnnnnnnnnnnnnnnnnnnnnnnnnnnnnnnnnnnnnnnnnnggcgtaccgttctggactatgcttctctggcggatacctcgtacactgacgagcagnnnnnnnnnnnnnnnnnnnnnnnnnnnnnnnnnnnnnnnnnnnnnnnnnnnnnnnnnnnnnnnnnnnnnnnnnnnnnnnnnnnnnnnnnnnnnnnnnnnnnnnnnnnnnnnnnnnnnnnnnnnnnnnnnnnnnnnnnnnnnnnnnnnnnnnnnnnnnnnnnnnnGTTCCCCACGTGGGACAGTAACGCAAACGCAATGATAAAGCCGGCGTTCGTAATTCCTGCGTACGAGGTGATggctcaggtggacgatcagggtaatGTACAGGCCCCCACAGAGGAGGAGAAGGCTTCTGGAAAGGGGCGTTTTGAAGATGGGTACGGAGTGGTAAAGaatnnnnnnnnnnnnnnnnnnnnnnnnnnnnnnnnnnnnnnnnnnnnttatcctcatggtttgtacgtgatgatgcgggatcaggatggtgaggtgcatcgctacttcATGGGGTATCTCCTGTTCGACTCCTGGAAGGAGTtggtgtggaacaatccttcgtatatctctgatgttcggtcgcgggaggtgcgcttgnnnnnnnnnnnnnnnnnnnnnnnnnnnnnngtcgtgttngaaggctttatggttactagggnnnnnnnnnnnnnnnnnnnnnnnnnnnnnnnnnnnnnnnnnnnnnnnnnnnnnnnnnnnnnnnnnnnnnnnnnnnnnnnnnnnnnnnnnnnnnnnnnnnnnnnnnnnnnnnnnnnnnnnnnnnnnnnnnnnnnnnnnnnnnnnnnnnnnnnnnnnnnnnnnnnnnnnnnnnnnnnnnnnnnnnnnnnnnnnnnnnnnnnnnnnnttgctacagaggttggttttacaccctctgggggtGCTCAGCGGCAGGAAGAGCAGCAGTagtgcagtagtcttcctagggagagggggcggtggggntctaggcgcggggcgtgtcttttccctctcttcttttcttgggttttagcggnnnnnnnnnnnnnnnnnnnnnnnnnnnnnnnnnnnnnnnnnnnnnnnnnnnnnnnnnnnnnnnnnnnnnnnnnnnnnnnnnnnnnnnnnnnnnnnnnnntctgcggcgcgtacttgtgcgcggaggtatttggctgctgttacatccggggatagggagagttctctgcctctacttaggagcttggtgaagcgacttgacaccgctgcccggaaaggtgttttcgctagaaaggctgtggctcgccannnnnnnnnnnnnnnnnnnnnnnnnnnnnnnnnnnnnnnnnnnnnnnnnnnnnnnnnnnnnnnnnnnnnnnnnnnnnnnnnnnnnnnnnnnnnnnnnnnnnnnnnnnnnnnnnnnnnnnnnnnnnnnnnnnnnnnnnnnnnnnnnnnnnnnngatttgagnnnnnnnnnnnnnnnnnnnnnnnnnnnnnnnnnnGTCTCTGTGGTAACCGCTGCATTGGAACGGGGGGAGACAGTCGAGCTGAGGGATTTTGGGGTGTTTGAGTCTCGCGTGCGTAAGGCTTCCGTCGGGAAGAGCATAAAGAnnnnnnnnnnnnnnnnnnnnnnnnnnnnnnnnnnnnnnnnnnnnnnnnnnnnnnnnnnnnnnnnnnnnnnnnnnnnnnnnnnnnnnnnnnnnnnnnnnnnnnnnnnnnnnnnnnnnnnnnnnnnnnnnnnnnnnnnnnnnnnnnnnnnnnnnnnnnnnnnnnnnnnnnnnnnnnnnnnnnnnnnnnnnnnnnnnnnnnnnnnnnnnnnnnnnnnnnnnnnnnnnnnnnnnnnnnnnnnnnnnnnnnnnnnnnnnnnnnnnnnnnnnnnnnnnnnnnnnnnnnnnnnnnnnnnnnnnnnnnnnnnnnnnnnnnnnnnnnnnnnnnnnnnnnnnnnnnnnnnnnnnnnnnnnnnnnnnnnnnnnnnnnnnnnnnnnnnnnnnnnnnnnnnnnnnnnnnnnnnnnnnnnnnnnnnnnnnnnnnnnnnnnnnnnnnnnnnnnnnnnnnnnnnnnnnnnnnnnnnnnnnnnnnnnnnnnnnnnnnnnnnnnnnnnnnnnnnnnnnnnnnnnnnnnnnnnnnnnnnnnnnnnnnnnnnnnnnnnnnnnnnnnnnnnnnnnnnnnnnnnnnnnnnnnnnnnnnnnnnnnnnnnnnnnnnnnnnnnnnnnnnnnnnnnnnnnnnnnnnnnnnnnnnnnnnnnnnnnnnnnnnnnnnnnnnnnnnnnnnnnnnnnnnnnnnngggcgtttttgtctgccgcgtatagccactgggtttcggcgcnnnnnnnnnnnnnnnnnnnnnnnnnnnnnnnnnnnnnnnnnnnnnnnnnnnnnnnnnnnnnnnnnnnnnnnnnnnnnnnnnnnnnnnnnnnnnnnnnnnngcctaatagnnnnnnnnnnnnnnnnnnnnnnnnnnnnnnnnnnnnnnnnnnnnnnnnnnnnnnnnnnnnnnnnnnnnnnnnnnnnnnnnnnnnnnnnnnnnnnnnnnnnnnnnnnnnnnnnnnnnnnnnnnnnnnnnnnnnnnnnnnnnngnnnnnnnnnnnnnnnnnnnnnnnnnnnnnnnnnnnnnnnnnnnnnnnnnnnnnnnnnnnnnnnnnnnnnnnnnnnnnnnnnnnnnntatcggtagtgacgacgcatataagaagcgcacgaaggaggggaattgggaacgtgttgattacnnnnnnnnnnnnnnnnnnnnnnnnnnnnnnnnnnnnnnnnnnnnnnnnnnnnnnnnnnnnnnnnnnnnnnnnnnnnnnnnnnnnnnnnnnnnnnnnnnnnnnnnnnnnnnnnnnnnnnnnnnnnnnnnnnnnnnnnnnnnnnnnnnnnnnnnnnnnnnnnnnnnnnnnnnnnnnnnnnnnnnnnnnnnnnnnnnnnnnnnnnnnnnnnnnnnnnnnnnnnnnnnnnnnnnnnnnnnnnnnnnnnnnnnnnnnnnnnnnnnnnnnnnnnnnnnnnnnnnnnnnnnnnnnnnnnnnnnnnnnnnnnnnnnnnnnnnnnnnnnnnnnnnnnnnnnnnnnnnnnnnnnnnnnnnnnnnnnnnnnnnnnnnnnnnnnnnnnnnnnnnnnnnnnnnnnnnnnnnnnnnnnnnnnnnnnnnnnnnnnnnnnnnnnnnnnnnnnnnnnnnnnnnnnnnnnnnnnnnnnnnnnnnnnnnnnnnnnnnnnnnnnnnnnnnnnnnnnnnnnnnnnnnnnnnnnnnnnnnnnnnnnnnnnnnnnnnnnnnnnnnnnnnnnnnnnnnnnnnnnnnnnnnnnnnnnnnnnnnnnnnnnnnnnnnnnnnnnnnnnnnnnnnnnnnnnnnnnnnnnnnnnnnnnnnnnnnnnnnnnnnnnnnnnnnnnnnnnnnnnnnnnnnnnnnnnnnnnnnnnnnnnnnnnnnnnnnnnnnnnnnnnnnnnnnnnnnnnnnnnnnnnnnnnnnnnnnnnnnnnnnnnnnnnnnnnnnnnnnnnnnnnnnnnnnnnnnnnnnnnnnnnnnnnnnnnnnnnnnnnnnnnnnnnnnnnnnnnnnnnnnnnnnnnnnnnnnnnnnnnnnnnnnntgatcgtgtggagatgagggatggaagccgagtgtttggggatgtcactgcttctagaattaaaatctgtgatggagttacnTTTGAGGGGTCTGTTTGCATGACTCGGGAAGGGAATGTTTCGAAGCGGGATCTATTTTCTGTCCAGTCTgagcaattgaaggagcatctgcgtcgttagcgtagatatggttgggtcttgactgaatgcctaaaagaggcgcacagttcctgtannnnnnnnnnnnnnnnnnnnnnnnnnnnnnnnnnnnnnnnnnnnnnnnnnnnnnnnnnnnnnnnnnnnnnnnnnnnnnnnnnnnnnnnnnnnnnnnnnnnnnnnnnnnnnnnnnnnnnnnnnnnnnnnnnnnnnnnnnnnnnnnnnnnnnnnnnnnnnnnnnnnnnnnnnnnnnnnnnnnnnnnnnnnnnnnnnnnnnnnnnnnnnnnnnnnnnnnnnnnnnnnnnnnnnnnnnnnnnnnnnnnnnnnnnnnnnnnnnnnnnnnnnnnnnnnnnnnnnnnnnnnnnnnnnnnnnnnnnnnnnnnnnnnnnnnnnnnnnnnnnnnnnnnnnnnnnnnnnnnnnnnnnnnnnnnnnnnnnnnnnnnnnnnnnnnnnnnnnnnnnnnnnnnnnnnnnnnnnnnnnnnnnnnnnnnnnnnnnnnnnnnnnnnnnnnnnnnnnnnnnnnnnnnnnnnnnnnnnnnnnnnnnnnnnnnnnnnnnnnnnnnnnnnnnnnnnnnnnnnnnnnnnnnnnnnnnnnnnnnnnnnnnnnnnnnnnnnnnnnnnnnnnnnnnnnnnnnnnnnnnnnnnnnnnnnnnnnnnnnnnnnnnnnnnnnnnnnnnnnnnnnnnnnnnnnnnnnnnnnnnnnnnnnnnnnnnnnnnnnnnnnnnnnnnnnnnnnnnnnnnnnnnnnnnnnnnnnnnnnnnnnnnnnnnnnnnnnnnnnnnnnnnnnnnnnnnnnnnnnnnnnnnnnnnnnnnnnnnnnnnnnnnnnnnnnnnnnnnnnnnnnnnnnnnnnnnnnnnnnnnnnnnnnnnnnnnnnnnnnnnnnnnnnnnnnnnnnnnnnnnnnnnnnnnnnnnnnnnnnnnnnnnnnnnnnnnnnnnnnnnnnnnnnnnnnnnnnnnnnnnnnnnnnnnnnnnnnnnnnnnnnnnnnnnnnnnnnnnnnnnnnnnnnnnnnnnnnnnnnnnnnnnnnnnnnnnnnnnnnnnnnnnnnnnnnnnnnnnnnnnnnnnnnnnnnnnnnnnnnnnnnnnnnnnnnnnnnnnnnnnnnnnnnnnnnnnnnnnnnnnnnnnnnnnnnnnnnnnnnnnnnnnnnnnnnnnnnnnnnnnnnnnnnnnnnnntcgatgagcgtcctgaagaggtaaccGACATGGAGCGTACGGTnnnnnnnnnnnnnnnnnnnnnnnnnnnnnnnnnnnnnnnnnnnngccatgtacagattgcagagatagtgcttgagcgcgcaaagcgattggtagagcaccggaaggacgtggtgattttgcttgactcgattannnnnnnnnnnnnnnnnnnnnnnnnnnnnnnnnnnnnnnnnnnnnnnnnGTTGTCGGGGGGagtggaTTCAAATGCTcTGCACAAACCGAAACGCTTCTTTGGTGCAGCTCGCAATGTGGAGGAAGGAGGAAGTCTGACAATCATTGCCACTGCGCTTATTGAAACAGGCAGTAGGATGGATGAGGTTATTTTTGAGGAGTTTAAAGGTACGGGGAATATGGAGATAAATCTCGATCGTCGCCTTTCGGATCGGCGCCTATTTCCCGCGGTGAGTATTAAAAAGTCTGGTACGCGTAAGGAAGAGTTGCTCCTGACGGAGGAAGAAATGCAGCGTATTTGGGTGTTGCGTAAGGTAATCAATCCtatggacgatgcagaaattgtggaattgttannnnnnnnnnnnnnnnnnnnnnnnnnnnnnnnnnnnnnnnnnnnnnnnnnnnnnnnnnnnnnnnnnnnnnnnnnnnnnnnnnnnnnnnnnnnnnnnnnnnnnnnnnnnnnnnnnnnnnnnnnnnnnnnnnnnnnnnnnnnnnnnnnnnnnnnnnnnnnnnnnnnnnnnnnnnnnnnnnnnnnnnnnnnnnnnnnnnnnnnnnnnnnnnnnnnnnnnnnnnnnnnnnnnnnnnnnnnnnnnnnnnnnnnnnnnnnnnnnnnnnnnnnnnnnnnnnnnnnnnnnnnnnnnnnnnnnnnnnnnnnnnnnnnnnnnnnnnnnnnnnnnnnnnnnnnnnnnnnnnnnnnnnnnnnnnnnnnnnnnnnnnnnnnnnnnnnnnnnnnnnnnnnnnnnnnnnnnnnnnnnnnnnnnnnnnnnnnnnnnnnnnnnnnnnnnnnnnnnnnnnnnnnnnnnnnnnnnnnnnnnnnnnnnnnnnnnnnnnnnnnnnnnnnnnnnnnnnnnnnnnnnnnnnnnnnnnnnnnnnnnnnnnnnnnnnnnnnnnnnnnnnnnnnnnnnnnnnnnnnnnnnnnnnnnnnnnnnnnnnnnnnnnnnnnnnnnnnnnnnnnnnnnnnnnnnnnnnnnnnnnnnnnnnnnnnnnnnnnnnnnnnnnnnnnnnnnnnnnnnnnnnnnnnnnnnnnnnnnnnnnnnnnnnnnnnnnnnnnnnnnnnnnnnnnnnnnnnnnnnnnnnnnnnnnnnnnnnnnnnnnnnnnnnnnnnnnnnnnnnnnnnnnnnnnnnnnnnnnnnnnnnnnnnnnnnnnnnnnnnnnnnnnnnnnnnnnnnnnnnnnnnnnnnnnnnnnnnnnnnnnnnnnnnnnnnnnnnnnnnnnnnnnnnnnnnnnnnnnnnnnnnnnnnnnnnnnnnnnnnnnnnnnnnnnnnnnnnnnnnnnnnnnnnnnnnnnnnnnnnnnnnnnnnnnnnnnnnnnnnnnnnnnnnnnnnnnnnnnnnnnnnnnnnnnnnnnnnnnnnnnnnnnnnnnnnnnnnnnnnnnnnnnnnnnnnnnnnnnnnnnnnnnnnnnnnnnnnnnnnnnnnnnnnnnnnnatgtcgtgaatgggattaaaactgaaggtagnatcagcgctcagtggagatatgcggcgtgctactatgcacggtgacgatctgtgcattctataacaggggaggagagaagtttatgcggggaacatattgtgtgacgcnnnnnnnnnnnnnnnnnnnnnnnnnnnnnnnnnnnnnnnnnnnnnnnnnnnnnnnnnnnnnnnnnnnnnnnnnnnnnnnnnnnnnnnnnnnnnnnnnnnnnnnnnnnnnnnnnnnnnnnnnnnnnnnnnnnnnnnnnnnnnnnnnnnnnnnnnnnnnnnnnnnnnnnnnnnnnnnnnnnnnnnnnnnnnnnnnnnnnnnnnnnnnnnnnnnnnnnnnnnnnnnnnnnnnnnnnnnnnnnnnnnnnnnnnnnnnnnnnnnnnnnnnnnnnnnnnnnnnnnnnnnnnnnnnnnnnnnnnnnnnnnnnnnnnnnnnnnnnnnnnnnnnnnnnnnnnnnnnnnnnnnnnnnnnnnnnnnnnnnnnnnnnnnnnnnnnnnnnnnnnnnnnnnnnnnnnnnnnnnnnnnnnnnnnnnnnnnnnnnnnnnnnnnnnnnnnnnnnnnnnnnnnnnnnnnnnnnnnnnnnnnnnnnnnnnnnnnnnnnnnnnnnnnnnnnnnnnnnnnnnnnnnnnnnnnnnnnnnnnnnnccctcgctctgttgaAAAAATACGGTTACCAGAGTCGATCGGATCTAGTGTATGTGCAAACGTATGATTTTAACGAGCTGAAGCGTATCAAACGAGAACTTTTACCAaagtacgaaatgaacgtgaagctgattcagcgtgtTGCTTACACAGATCAACGTGAAACacaggagaaggactcgcgtgGGAAATGGATAAACTACAATTACAATTGGATGTTTGAGCCCGGTGGTATGCAGAAAATAGCAAAATATGCAGACGGCGTGGGTCCTGACTGGAGGATGCTCATAGAGAATGAATGGTCGAAGGTGGGCGCTGTTCGCCTGAGTCCGATGGTTTCTGCAATCCAAGATGCGAAATTGGAATGTCATGTGCACACGGTACGGAAAGAAACACTGCCTAGCTACGCGCGCACCATGGACGAGATGTTTTCCATTTTGTTCaaacagnnnnnnnnnnnnnnnnnnnnnnnngATTTTCCtgatcttggggtAAAGTTTCTGGGCAAACCCGCCCGCTATTGACCGGCTTCTGTGTAGTCCAGGCGATGTAAGTGTTCAGTGTCGCCTGGTACCGGTGGAGTTCGTTCTCAGTAGGTGTCGTTCATCTCCATGAAGCGAGCACGCACCGCCTCGAGACGTGCGagctcgcgcgatatggtannnnnnnnnnnnnnnnnnnnnnnnnnnnnnnnnnnnnnnnnnnnnnnnnnnnnnnnnnnnnnnnnnnnnnnnnnnnnnnnnnnnnnnnnnnnnnnnnnnnnnnnnnnnnnnnnnnnnnnnnnnnnnnnnnnnnnnnnnnnnnnnnnnnnnnnnnnnnnnnnnnnnnnnnnnnnnnnnnnnnnnnnnnnnnnnnnnnnnnnnnnnnnnnnnnnnnnnnnnnnnnnnnnnnnnnnnnnnnnnnnnnnnnnnnnnnnnnnnnnnnnnnnnnnnnnnnnnnnnnnnnnnnnnnnnnnnnnnnnnnnnnnnnnnnnnnnnnnnnnnnnnnnnnnnnnnnnnnnnnnnnnnnnnnnnnnnnnnnnnnnnnnnnnnnnnnnnnnnnnnnnnnnnnnnnnnnnnnnnnnnnnnnnnnnnnnnnnnnnnnnnnnnnnnnnnnnnnnnnnnnnnnnnnnnnnnnnnnnnnnnnnnagataggcatgcgctgggtcgccgcacagcgcacagacagtggtgagaagagtaaaaacggcggaacgaaagctacgcgcacccacgtGCGTCACTGTTTCGGTTGATTGTGTGCGTATCCTTAATGAATTGCAGTGCCATCCTTTCCGATgaaatggacctaaagagagcacgctgctcccggcagaggtgggaaggacggtgtgtgcgcttgctaagagtatgccgtatgattttccnnnnnnnnnnnnnnnnnnnnnnnnnnnnnnnnnnnnnnnnnnnnnnnnnnnnnnnnnnnnnnnnnnnnnnnnnnnnnnnnnnnnnnnnaggagacgatgangaggaagcttagtacgggcctgttgctgtggattgcgtttatctcgggtgttacgtcctgcaagtctgcgcctccggcggaggagctcgtggaagttgcgccgcctgtggaggagcagGAagaagagcccatgacgccnnnnnnnnnnnnnnnnnnnnnnnnnnnnnnnnnnnnnnnnnnnnnGGTGTCGCGTGCTGAGTACGTGGTCAAAAACGAAGACACCCTTTCTCAAATTGCTAAAAAGTTTTACGGCTcgcgcatccgcggatnnnnnnnnnnnnnnnnnnnngcctgtagtgagggcgtggtaacgcatccagaccgcattaggccaggtatgaagttggttattccgaactttgacgagtttatggctgatccggatcatgtccgcagnnnnnnnnnnnnnnnnnnnnnnnnnnnnnnnnnntatcgttctgnnnnnnnnnnnnnnnnAGCTGAGTTTATGAACAAACTnGGGGAAAAAATCGGCAAGACCGATCCTCGGGACATGCCGCGCTAGGAGTCACTGCGCGCCAGTTTGCTCAAATCCGCGCCGGGGTGGCAGCTnnnnnnnnnnnnnnnnnnnnnnnnnnnnnnnnnnnnnnnnnnnnnnnnnnnnnnnnnnnnnnnnnnnnnnnnnnnnnnnnnnnnnnnnnnnnnnnnnnnnnnnnnnnnnnnnnnnnnnnnnnnnnnnnnnnnnnnnnnnnnnnnnnnnnnnnnnnnnnnnnnnnnnnnnnnnnnnnnnnnnnnnnnnnnnnnnnnnnnnnnnnnccgggggtgcgtaagtacaggaagtgcacccattcctgcgcgctgtcaaagtcgaaggacagcaccccttcatactcgcttttcagcgattGATCTATGAAGCACCGCAGTGCTACACGCCCCGTGCTCCCTGCCCCTGCGGGGATAAGCGCCGGGGACAGCGCGCGCGCCCCTGTCCAGGTGAAGACGTTGCGTTCTGTAAAAGACnnnnnnnnnnnnnnnnnnnnnnnnnnnnnnnnnnnnnnnnnnnnnnnnnnnnnnnnnnnnnnnnnnnnnnnnnnnnnnnnnnnnnnnnnnnnnnnnnnnnnnnnnnnnnnnnnnnnnnnnnnnnnnnnnnnnnnnnnnnnnnnnnnnnnnnnnnnnnnnnnnnnnnnnnnnnnnnnnnnnnnnnnnnnnnnnnnnnnnnnnnnnnnnnnnnnnnnnnnnnnnnnnnnnnnnnnnnnnnnnnnnnnnnnnnnnnnnnnnnnnnnnnnnnnnnnnnnnnnnnnnnnnnnnnnnnnnnnnnnnnnnnnnnnnnnnnnnnnnnnnnnnnnnnnnnnnnnnnnnnnnnnnnnnnnnnnnnnnnnnnnnnnnnnnnnnnnnnnnnnnnnnnnnnnnnnnnnnnnnnnnnnnnnnnnnnnnnnnnnnnnnnnnnnnnnnnnnnnnnnnnnnnnnnnnnnnnnnnnnnnnnnnnnnnnnnnnnnnnnnnnnnGGGATAGACCGTGAGAAAAACACCATCCGGTGGTACCGTcttcggtcatgaccttgaacnnnnnnnnnnnnnnnnnnnnnnnnnnnnnnnnnnnnnnnnnnnnnnnnnnnnnnnnnnnnnnnnnnnnnnnnnnnnnnnnnnnnnnnnnnnnnnnnnnnnnnnnnnnnnnnnnnnnnnnnnnnnnnnnnnnnnnnnnnnnnnnnnnnnnnnnnnnnnnnnnnnnnnnnnnnnnnnnnnnnnnnnnnnnnnnnnnnnnnnnnnnnnnnnnnnnnnnnnnnnnnnnnnnnnnnnnnnnnnnnnnnnnnnnnnnnnnnnnnnnnnnnnnnnnnnnnnnnnnnnnnnnnnnnnnnnagcgcacgtacaccnnnnnnnnnnnnnnnnnnnnnnnnnnnnnnnnnnnnnnnnnccactggaggacgccgtaGCCAACCGTGTTGGTGCAAGAAAACAGCAGAAGCAGAAAGGGCACCAGCAGGCCGAGCGCACAGAAGCGCCTCCAGATagagtgaacttgcacataccgatantagcacacccacctntcctggaaaaggcnnnnnnnnnnnnnnnnnnnnnnnnnnnnnnnnnnnnnnnnnnnnnnnnnnnnnnnnnnnnnnnnnnnnnnnnnnnnnnnnnnnnnnnnnnnnnnnnnnnnnnnnnnnnnnnnnnnnnnnnnnnnnnnnnnnnnnnnnnnnnnnnnnnnnnnnnnnnnnnnnnnnnnnnnnnnnnnnnnnnnnnnnnnnnnnnnnATCTTGACGATCGTGTCATGATCCTGAATAAGGGGCACGTGGCGCTTACCTTTGCGGATGTCGAAACAGGGTCGCAGGTGACTGAGTACATTAAAACTGGTGAGTTCTTTGGGGttaagtctgcgcnnnnnnnnnnnnnnnnnnnnnnnnnnnnnnnnnnnnnnnnnnnnnnnnnnnnnnnnnnnnnnnnnnnnnnnnnnnnnnnnnnnnnnnnnnnnnnnnnnnnnnnnnnnnnnnnnnnnnnnnnnnnnnnnnnnnnnnnnnnnnnnnnnnnnnnnnnnnnnnnnnnnnnnnnnnnnnnnnnnnnnnnnnnnnnnnnnnnnnnnnnnnnnnnnnnnnnnnnnnnnnnnnnnnnnnnnnnnnnnnnnnnnnnnnnnnnnnnnnnnnnnnnnnnnnnnnnnnnnnnnnnnnnnnnnnnnnnnnnnnnnnnnnnnnnnnnnnnnnnnnnnnnnnnnnnnnnnnnnnnnnnnnnnnnnnnnnnnnnnnnnnnnnnnnnnnnnnnnnnnnnnnnnnnnnnnnnnngttttgaggtgccttctgcttttGCTGAGTCAGCTGTCGATTCTGAAGTGGGGGATCTGCGTGCCGCAGAGCTTCTTGAGCAACAGGGAAAGTGGGGTGAGGCTTATGAGCATTACCGGTTAGGGTGCGCGTCGAACGGGGGAGtgggcgcggaggctgcgtaTCTGGGTGCGGGGCGGTGTCTTTTTGAGCAGAGGGagtttgtgcggagcattcagacgttcactgagtgcattannnnnnnnnnnnnnnnnnnnnnnnnnnnnnnnnnnnnnnnnnnnnnnnnnnngtgctatcagggtatggggaggccggataaGGCGATTTCnnnnnnnnnnnnnnnnnnnnnnnnnnnnnnnnnnnnnnnnnnnnnnnnnnnnnnnnnnnnnnnnnnnnnnnnnnnnnnnnnnnnnnnnnnnnnnnnnnnnnnnnnnnnnnnnnnnnnnnnnnnnnnnnnnnnnnnnnnnnnnnnnnnnnnnnnnnnnnnnnnnnnnnnnnnnnnnnnnnnnnnnnnnnnnnnnnnnnnnnnnnnnnnnnnnnnnnnnnnnnnnnnnnnnnnnnnnnnnnnnnnnnnnnnnnnnnnnnnnnnnnnnnnnnnnnnnnnnnnnnnnnnnnnnnnnnnnnnnnnnnnnnnnnnnnnnnnnnnnnnnnnnnnnnnTTGTGGCGCTGGAGTTCAATCGGGAGAATTTCGAAGTGCTCATGATGGGTAATCCTGCTATTGCAATGCGGCTTTTGAAAACGTTTGTGCGGCGTATTTACACCCAGAAGCGGCGTTTTATGATTTTAACGATTCAAGATAGTACTGCGCGTGTGGGGGATGTTTTCTTGATGTTGGATGAAACGCAACCGCACGTGGATcgcagcagcgacgcgcgttcttttgatatcagcatcgaagagnnnnnnnnnnnnnnnnnnnnnnnnnnnnnnnnnnnnnnngacgcgctgcgcaggctttctgatcagcggaaggttgagtgtCAAGAGCGCCGGATAGTAGTTAAAAATAtgacggatttgaaccgctacgtgACCATGCGCCGGGGTAAGGGTGATGGTTTGCCCTTAGGCTGAACCGTTGTAGCGGAGTGCCCATTCGTTTTCGCTGTCTGCTGAGGAGACGCTCTTTTTCCCTGGGGTGTACCGTGCGCGTACATACAAAGGCGCTGCGCGGGCTTTGCGTGCAGCTCTGGTTCTGTACGGTGTGGGAGCTGTGTGAGCGCTCATTATTGATTATTCGTGTGGAAAGGCCGATACTCGGCGGCGATGGGTGCGCTTTTTGCGACAATATTCGCCcttgttggggtnnnnnnnnnnnnnnnnnnnnnnnnnnnnnntgctgcgggtgtgctgtatGCAAAGGAAGCGCAGCTGCTGCTCGGGGGTGCGCnggggtggcagtcgcttacacgggtaaaaaacctgCGTACGCGTGTGGGGCGAGCGGGCCATCCGGCGCTTGAACTTTCTTCTGCTGCGCGTACCCGCAGTGTTCGGGGTGACTTGTATCTTAGTTTTGATGATCCGTTGGTCAGCGATTTGTACGGTCGTTATCGTGTGCAGTCCTCCTCTGCGCAGCATGtgggcgaagagcgtgcgcatcgcggnnnnnnnnnnnnnnnnnnnnnnnnnnnnnnnnnnnnnnnnnnnnnnnnnnnnnnnnnnnnnnnnnnnnnnnnnnnnnnnnnnnnnnnnnnnnnnnnnnnnnnnnnnnnnnnnnnnnnnnnnnnnnnnnnnnnnnnnnnnnnnnnnnccattgagttttggctgtatcctgcggtgagtgagaacggggcgGTGTTGTTCCGGTGGCGTTCTTCTCTTTCTGATCGCGGACGTTCTTTTTATCAGCATATTGTGGCGCACATTTTACAGCACCGGCTTGAGTGGCGCGCGGAGGGGTTGTGGAATGACGTGCGCGGACAGGCAGTCTCTTTGCGTCTCCGTTCGCGTACGCACGTGCTGCCAGAGCGGTGGAGCCACCATATGCTCACGTACGACGAAACGCGCGGTGTGCTTGAGTATCGCATGAATGGGCGTACAGAATGTCTTGAGTACCTCACGGATTCGCAGGATGAAACTGGCCAGGTGTGGCAnnnnnnnnnnnnnnnnnnnnnnnnnnnnnnnnnnnnnnnnnnnnaCTCTGGTCTGATCGATGAGGTGGtgattactgaAGAATTTGTTCCGCCGTCTGAAGCAGGAGAGGTGCTGCGCCGTTTTAACCGTCTTGCGCGTTTTGACCAGGCAGGTGGCCGCTTTGAGTCGGAGATCACGGATGCAGGAGGACTGCAggcggtggtccggcgtgtgaatgcaGATGTGGATATTCCAGAGCAGGCAGATGTGGCGTTCTTCGTGCGAGTAGGACAAACGAAAGAGGACTGGACGTTGGAGTACCCGTTGTGGCAGCCGGTGGTGGCAGGCCAGCCGCTCAGCGGATTGGAAGGACGATACTTCCAGGTGGCTGTGCAATTGTATCCAGATGGTGCAGgtcgtaagacnnnnnnnnnnnnnnnnnnnnnnnnnnnnnnnnnnnnnnnnnnnnnnnnnnnnnnnnnnnnnnnnntttTTGCGCAGGCGGCTGACGGGTCGGTGATACTCACGTGGACACCTTCGGTTGACTTTGACGTAGAGGGGTATGTGGTGTATGTGGGCGACACGTCAGGGATGTATTTTGTGGCGGGATCTCCTATTCGGGTGGGTAAGCGTTTGTCATAtacnnnnnnnnnnnnnnnnnnnnnnnnnnnnnnnnnnnnnnnnnnnnnnnnnnnnnnnnnnnnnnnnnnnnnnnnnnnnGGCCAATTGTCCGCGGAGGCGTGGGCGCGTCCGCTTCCCCATCTAGGTacggctgacattccgtcccagggaggatagtgtcatggaactcaatGCATATAttgaccacacgctcttaaggccctctgcgtctgaggccgaagtgttgcgtctgtgccaagaggcgcagcactatcgcttcgcgtcggtatnnnnnnnnnnnnnnnnnnnnnnnnnnnnnnnnnnnnnnnnnnnnnnnnnnnnnnnnnnnnnnnnnnnnnnnnnnnnnnnnnnnnnnnnnnnnnnnnnnnnnnnnnnnnnnnnnnnnnnnnnnnnnnnnnnnnnnnnnnnnnnnnnnnnnnnnnnnnnnnnnnnnnnnnnnnnnnnnnnnnnnnnnnnnnnnnnnnnnnnnnnnnnnnnnnnnnnnnnnnnnnnnnnnnnnnnnnnnnnnnnnnnnnnnnnnnnnnnnnnnnnnnnnnnnnnnnnnnnnnnnnnnnnnnnnnnnnnnnnnnnnnnnnnnnnnnnnnnnnnnnnnnnnnnnnnnnnnnnnnnnnnnnnnnnnnnnnnnnnnnnnnnnnnnnnnnnnnnnnnnnnnnnnnnnnnnnnnnnnnnnnnnnnnnnnnnnnnnnnnnnnnnnnnnnnnnnnnnnnnnnnnnnnnnnnnnnnnnnnnnnnnnnnnnnnnnnnnnnnnnnnnnnnnnnnnnnnnnnnnnnnnnnnnnnnnnnnnnnnnnnnnnnnnnnnnnnnnnnnnnnnnnnnnnnnnnnnnnnnnnnnnnnnnnnnnnnnnnnnnnnnnnnnnnnnnnnnnnnnnnnnnnnnnnnnnnnnnnnnnnnnnnnnnnnnnnnnnnnnnnnnnnnnnnnnnnnnnnnnnnnnnnnnnnnnnnnnnnnnnnnnnnnnnnnnnnnnnnnnnnnnnnnnnnnnnnnnnnnnnnnnnnnnnnnnnnnnnnnnnnnnnnnnnnnnnnnnnnnnnnnnnnnnnnnnnnnnnnnnnnnnnnnnnnnnnnnnnnnnnnnnnnnnnnnnnnnnnnnnnnnnnnnnnnnnnnnnnnnnnnnnnnnnnnnnnnnnnnnnnnnnnnnnnnnnnnnnnnnnnnnnnnnnnnnnnnnnnnnnnnnnnnnnnnnnnnnnngcactcgctctttaggacgggggttcgattccccccatctccacaagatggtgagatccagtggtcagttatccgtgaggggtagcgtaccgcgaggcgtgcatgttttttcacgtaagcgtgcatgcgcaGTACTAAGTataagaactgccgcgctggggataagccagcagagctGTTCCTGTGCGAGTGGAAGGGTATGTATGAGTGTATTCAACACGTGTCCTATCCGGGTGTGCGCCACAAGCGGCGTACCGACTGCACAGGTGCTGAGTGTTCCTGCGCTCCATACTGTCAGGCGGTAGGCGGCGCGTGGTATCCGTATGAGtcgctcgaggtgtgatatgnnnnnnnnnnnnnnnnnnnnnnnnnnnnggatcatgagtaggggnnnnnnnnnnnnnnnnnnnnnnnnnnGTCCTGTATTTGCGAGCGCAAAGCCGGATATGGTGAACAGGCGTATCCAGCACAGGTAAGACACACGGGGTGCGCGTTTGTGGAAGTATTCGCTGACGCAAACAAGCAGtccggcgcaggtagttaggcaggcaattgtaannnnnnnnnnnnnnnnnnnnnnnnnnnnnnnnnnnnnnnnnnnnnnnnnnnnnnnnnnnnnnnnnnnnnnnnnnnnnnnnnnnnnnnnnnnnnnnnnnnnnnnnnnnnnnnnnnnnnnnnnnnnnnnnnnnnnnnnnnnnnnnnnnnnnnnccctgctatccaggcgacggtgcgcatatctgcgagggtgtttgctggagtgggagggaaaggggcggttgtgtagcgggtccgatgcaaataggtaaggatcatattgccgaacataagggaggcnnnnnnnnnnnnnnnnnnnnnnnnnnnnnnnnnnnnnnnnnnnnnnnnnnnnnnnnnnnnnnnnnnnnnnnnnnnnnnnnngtagggaccgagtgncggagtgaaggaggcaaggaagaggaggagaacgagtactaggagcgcgggggtaagtactttgccgagagtgttgcttaaggtgtgtgggcgcatgcagagccagtaggaaacaatgaagaagcannnnnnnnnnnnnnnnnnnnnnnnnnnnnnnnnnnnnnnnnnnnnnnnnnnnnnnnnnnnnnnnnnnnnnnnnnnnnnnnnnnnnnnnnnnnnnnnnnnnnnnnnnnnnnnnnnnnnnnnnnnnnnnnnnnnnnnnnnnnnnnnnnnnnnnnnnnnnnnnnnnnnnnnnnnnnnnnnnnnnnnnnnnnnnnnnnnnnnnnnnnnnnnnnnnnnnnnnnnnnnnnnnnnnnnnnnnnnnnnnnnnnnnnnnnnnnnnnnnnnnnnnnnnnnnnnnnnnnnnnnnnnnnnnnnnnnnnnnnnnnnnnnnnnnnnnnnnnnnnnnnnnnnnnnnnnnnnnnnnnnnnnnnnnnnnnnnnnnnnnnnnnnnnnnnnnnnnnnnnnnnnnnnnnnnnnnnnnnnnnnnnnnnnnnnnnnnnnnnnnnnnnnnnnnnnnnnnnnnnnnnnnnnnnnnnnnnnnnnnnnnnnnnnnnnnnnnnnnnnnnnnnnnnnnnnnnnnnnnnnnnnnnnnnnnnnnnnnnnnnnnnnnnnnnnnnnnnnnnnnnnnnnnnnnnnnnnnnnnnnnnnnnnnnnnnnnnnnnnnnnnnnnnnnnnnnnnnnnnnnnnnnnnnnnnnnnnnnnnnnnnnnnnnnnnnnnnnnnnnnnnnnnnnnnnnnnnnnnnnnnnnnnnnnnnnnnnnnnnnnnnnnnnnnnnnnnnnnnnnnnnnnnnnnnnnnnnnnnnnnnnnnnnnnnnnnnnnnnnnnnnnnnnnnnnnnnnnnnnnnnnnnnnnnnnnnnnnnnnnnnnnnnnnnnnnnnnnnnnnnnnnnnnnnnnnnnnnnnnnnnnnnnnnnnnnnnnnnnnnnnnnnnnnnnnnnnnnnnnnnnnnnnnnnnnnnnnnnnnnnnnnnnnnnnnnnnnnnnnnnnnnnnnnnnnnnnnnnnnnnnnnnnnnnnnnnnnnnnnnnnnnnnnnnnnnnnnnnnnnnnnnnnnnnnnnnnnnnnnnnnnnnnnnnnnnnnnnnnnnnnnnnnnnnnnnnnnnnnnnnnnnnnnnnnnnnnnnnnnnnnnnnnnnnnnnnnnnnnnnnnnnnnnnnnnnnnnnnnnnnnnnnnnnnnnnnnnnnnnnnnnnnnnnnnnnnnnnnnnnnnnnnnnnnnnnnnnnnnnnnnnnnnnnnnnnnnnnnnnnnnnnnnnnnnnnnnnnnnnnnnnnnnnnnnnnnnnnnnnnnnnnnnnnnnnnnnnnnnnnnnnnnnnnnnnnnnnnnnnnnnnnnnnnnnnnnnnnnnnnnnnnnnnnnnnnnnnnnnnnnnnnnnnnnnnnnnnnnnnnnnnnnnnnnnnnnnnnnnnnnnnnnnnnnnnnnnnnnnnnnnnnnnnnnnnnnnnnnnnnnnnnnnnnnnnnnnnnnnnnnnnnnnnnnnnnnnnnnnnnnnnnnnnnnnnnnnnnnnnnnnnnnnnnnnnnnnnnnnnnnnnnnnnnnnnnnnnnnnnnnnnnnnnnnnnnnnnnnnnnnnnnnnnnnnnnnnnnnnnnnnnnnnnnnnnnnnnnnnnnnnnnnnnnnnnnnnnnnnnnnnnnnnnnnnnnnnnnnnnnnnnnnnnnnnnnnnnnnnnnnnnnnnnnnnnnnnnnnnnnnnnnnnnnnnnnnnnnnnnnnnnnnnnnnnnnnnnnnnnnnnnnnnnnnnnnnnnnnnnnnnnnnnnnnnnnnnnnnnnnnnnnnnnnnnnnnnnnnnnnnnnnnnnnnnnnnnnnnnnnnnnnnnnnnnnnnnnnnnnnnnnnnnnnnnnnnnnnnnnnnnnnnnnnnnnnnnnnnnnnnnnnnnnnnnnnnnnnnnnnnnnnnnnnnnnnnnnnnnnnnnnnnnnnnnnnnnnnnnnnnnnnnnnnnnnnnnnnnnnnnnnnnnnnnnnnnnnnnnnnnnnnnnnnnnnnnnnnnnnnnnnnnnnnnnnnnnnnnnnnnnnnnnnnnnnnnnnnnnnnnnnnnnnnnnnnnnnnnnnnnnnnnnnnnnnnnnnnnnnnnnnnnnnnnnnnnnnnnnnnnnnnnnnnnnnnnnnnnnnnnnnnnnnnnnnnnnnnnnnnnnnnnnnnnnnnnnnnnnnnnnnnnnnnnnnnnnnnnnnnnnnnnnnnnnnnnnnnnnnnnnnnnnnnnnnnnnnnnnnnnnnnnnnnnnnnnnnnnnnnnnnnnnnnnnnnnnnnnnnnnnnnnnnnnnnnnnnnnnnnnnnnnnnnnnnnnnnnnnnnnnnnnnnnnnnnnnnnnnnnnnnnnnnnnnnnnnnnnnnnnnnnnnnnnnnnnnnnnnnnnnnnnnnnnnnnnnnnnnnnnnnnnnnnnnnnnnnnnnnnnnnnnnnnnnnnnnnnnnnnnnnnnnnnnnnnnnnnnnnnnnnnnnnnnnnnnnnnnnnnnnnnnnnnnnnnnnnnnnnnnnnnnnnnnnnnnnnnnnnnnnnnnnnnnnnnnnnnnnnnnnnnnnnnnnnnnnnnnnnnnnnnnnnnnnnnnnnnnnnnnnnnnnnnnnnnnnnnnnnnnnnnnnnnnnnnnnnnnnnnnnnnnnnnnnnnnnnnnnnnnnnnnnnnnnnnnnnnnnnnnnnnnnnnnnnnnnnnnnnnnnnnnnnnnnnnnnnnnnnnnnnnnnnnnnnnnnnnnnnnnnnnnnnnnnnnnnnnnnnnnnnnnnnnnnnnnnnnnnnnnnnnnnnnnnnnnnnnnnnnnnnnnnnnnnnnnnnnnnnnnnnnnnnnnnnnnnnnnnnnnnnnnnnnnnnnnnnnnnnnnnnnnnnnnnnnnnnnnnnnnnnnnnnnnnnnnnnnnnnnnnnnnnnnnnnnnnnnnnnnnnnnnnnnnnnnnnnnnnnnnnnnnnnnnnnnnnnnnnnnnnnnnnnnnnnnnnnnnnnnnnnnnnnnnnnnnnnnnnnnnnnnnnnnnnnnnnnnnnnnnnnnnnnnnnnnnnnnnnnnnnnnnnnnnnnnnnnnnnnnnnnnnnnnnnnnnnnnnnnnnnnnnnnnnnnnnnnnnnnnnnnnnnnnnnnnnnnnnnnnnnnnnnnnnnnnnnnnnnnnnnnnnnnnnnnnnnnnnnnnnnnnnnnnnnnnnnnnnnnnnnnnnnnnnnnnnnnnnnnnnnnnnnnnnnnnnnnnnnnnnnnnnnnnnnnnnnnnnnnnnnnnnnnnnnnnnnnnnnnnnnnnnnnnnnnnnnnnnnnnnnnnnnnnnnnnnnnnnnnnnnnnnnnnnnnnnnnnnnnnnnnnnnnnnnnnnnnnnnnnnnnnnnnnnnnnnnnnnnnnnnnnnnnnnnnnnnnnnnnnnnnnnnnnnnnnnnnnnnnnnnnnnnnnnnnnnnnnnnnnnnnnnnnnnnnnnnnnnnnnnnnnnnnnnnnnnnnnnnnnnnnnnnnnnnnnnnnnnnnnnnnnnnnnnnnnnnnnnnnnnnnnnnnnnnnnnnnnnnnnnnnnnnnnnnnnnnnnnnnnnnnnnnnnnnnnnnnnnnnnnnnnnnnnnnnnnnnnnnnnnnnnnnnnnnnnnnnnnnnnnnnnnnnnnnnnnnnnnnnnnnnnnnnnnnnnnnnnnnnnnnnnnnnnnnnnnnnnnnnnnnnnnnnnnnnnnnnnnnnnnnnnnnnnnnnnnnnnnnnnnnnnnnnnnnnnnnnnnnnnnnnnnnnnnnnnnnnnnnnnnnnnnnnnnnnnnnnnnnnnnnnnnnnnnnnnnnnnnnnnnnnnnnnnnnnnnnnnnnnnnnnnnnnnnnnnnnnnnnnnnnnnnnnnnnnnnnnnnnnnnnnnnnnnnnnnnnnnnnnnnnnnnnnnnnnnnnnnnnnnnnnnnnnnnnnnnnnnnnnnnnnnnnnnnnnnnnnnnnnnnnnnnnnnnnnnnnnnnnnnnnnnnnnnnnnnnnnnnnnnnnnnnnnnnnnnnnnnnnnnnnnnnnnnnnnnnnnnnnnnnnnnnnnnnnnnnnnnnnnnnnnnnnnnnnnnnnnnnnnnnnnnnnnnnnnnnnnnnnnnnnnnnnnnnnnnnnnnnnnnnnnnnnnnnnnnnnnnnnnnnnnnnnnnnnnnnnnnnnnnnnnnnnnnnnnnnnnnnnnnnnnnnnnnnnnnnnnnnnnnnnnnnnnnnnnnnnnnnnnnnnnnnnnnnnnnnnnnnnnnnnnnnnnnnnnnnnnnnnnnnnnnnnnnnnnnnnnnnnnnnnnnnnnnnnnnnnnnnnnnnnnnnnnnnnnnnnnnnnnnnnnnnnnnnnnnnnnnnnnnnnnnnnnnnnnnnnnnnnnnnnnnnnnnnnnnnnnnnnnnnnnnnnnnnnnnnnnnnnnnnnnnnnnnnnnnnnnnnnnnnnnnnnnnnnnnnnnnnnnnnnnnnnnnnnnnnnnnnnnnnnnnnnnnnnnnnnnnnnnnnnnnnnnnnnnnnnnnnnnnnnnnnnnnnnnnnnnnnnnnnnnnnnnnnnnnnnnnnnnnnnnnnnnnnnnnnnnnnnnnnnnnnnnnnnnnnnnnnnnnnnnnnnnnnnnnnnnnnnnnnnnnnnnnnnnnnnnnnnnnnnnnnnnnnnnnnnnnnnnnnnnnnnnnnnnnnnnnnnnnnnnnnnnnnnnnnnnnnnnnnnnnnnnnnnnnnnnnnnnnnnnnnnnnnnnnnnnnnnnnnnnnnnnnnnnnnnnnnnnnnnnnnnnnnnnnnnnnnnnnnnnnnnnnnnnnnnnnnnnnnnnnnnnnnnnnnnnnnnnnnnnnnnnnnnnnnnnnnnnnnnnnnnnnnnnnnnnnnnnnnnnnnnnnnnnnnnnnnnnnnnnnnnnnnnnnnnnnnnnnnnnnnnnnnnnnnnnnnnnnnnnnnnnnnnnnnnnnnnnnnnnnnnnnnnnnnnnnnnnnnnnnnnnnnnnnnnnnnnnnnnnnnnnnnnnnnnnnnnnnnnnnnnnnnnnnnnnnnnnnnnnnnnnnnnnnnnnnnnnnnnnnnnnnnnnnnnnnnnnnnnnnnnnnnnnnnnnnnnnnnnnnnnnnnnnnnnnnnnnnnnnnnnnnnnnnnnnnnnnnnnnnnnnnnnnnnnnnnnnnnnnnnnnnnnnnnnnnnnnnnnnnnnnnnnnnnnnnnnnnnnnnnnnnnnnnnnnnnnnnnnnnnnnnnnnnnnnnnnnnnnnnnnnnnnnnnnnnnnnnnnnnnnnnnnnnnnnnnnnnnnnnnnnnnnnnnnnnnnnnnnnnnnnnnnnnnnnnnnnnnnnnnnnnnnnnnnnnnnnnnnnnnnnnnnnnnnnnnnnnnnnnnnnnnnnnnnnnnnnnnnnnnnnnnnnnnnnnnnnnnnnnnnnnnnnnnnnnnnnnnnnnnnnnnnnnnnnnnnnnnnnnnnnnnnnnnnnnnnnnnnnnnnnnnnnnnnnnnnnnnnnnnnnnnnnnnnnnnnnnnnnnnnnnnnnnnnnnnnnnnnnnnnnnnnnnnnnnnnnnnnnnnnnnnnnnnnnnnnnnnnnnnnnnnnnnnnnnnnnnnnnnnnnnnnnnnnnnnnnnnnnnnnnnnnnnnnnnnnnnnnnnnnnnnnnnnnnnnnnnnnnnnnnnnnnnnnnnnnnnnnnnnnnnnnnnnnnnnnnnnnnnnnnnnnnnnnnnnnnnnnnnnnnnnnnnnnnnnnnnnnnnnnnnnnnnnnnnnnnnnnnnnnnnnnnnnnnnnnnnnnnnnnnnnnnnnnnnnnnnnnnnnnnnnnnnnnnnnnnnnnnnnnnnnnnnnnnnnnnnnnnnnnnnnnnnnnnnnnnnnnnnnnnnnnnnnnnnnnnnnnnnnnnnnnnnnnnnnnnnnnnnnnnnnnnnnnnnnnnnnnnnnnnnnnnnnnnnnnnnnnnnnnnnnnnnnnnnnnnngcccttcatggtacgtggtgtttttgaagggagcatcgagtcctcgggtgaTCTTATGGTTGAGGAGCAGGCGCGTGTTCGCGCTGAGATAGTTGCAGATCGTGTTGTCATTAAGGGAGAAGTAATCGGGAACGTCACGGCTATGTCTGTTGTTCGCGTCTTCCCATGTGGGAGGTTGATAGGGGATGnnnnnnnnnnnnnnnnnnnnnnnnnnnnnnnnnnnnntttcagtggcgtgtgTAGTATGCCCGAATCGGGTTGATCGGTTTCCCTTGCAGAGGGGTAGCTCTCTTGGTCGCTAGGTTTCTTTTGCCCGTTGTGTTCTGTTTCGTGTGTAGGGGGCTCTGTTCGCAGCAGTCTGGTCTTGCTTTCTACnnnnnnnnnnnnnnnnnnnnnnnnnnnnnnnnnnnnnnnnnnnnnnnnnnnnnnnnnnnnnnnnnnnnnnnnnnnnnnnnnnnnnnnnnnnnnnnnnnnnnnnnnnnnnnnnnnnnnnnnnnnnnnnnnnnnnnnnnnnnnnnnnnnnnnnnnnnnnnnnnnnnnnnnnnnnnnnnnnnnnnnnnnnnnnnnnnnnnnnnnnnnnnnnnnnnnnnnnnnnnnnnnnnnnnnnnnnnnnnnnnnnnnnnnnnnnnnnnnnnnnnnnnnnnnnnnnnnnnnnnnnnnnnnnnnnnnnnnnnnnnnnnnnnnnnnnnnnnnnnnnnnnnnnnnnnnnnnnnnnnnnnnnnnnnnnnnnnnnnnnnnnnnnnnnnnnnnnnnnnnnnnnnnnnnnnnnnnnnnnnnnnnnnnnnnnnnnnnnnnnnnnnnnnnnnnnnnnnnnnnnnnnnnnnnnnnnnnnnnnnnnnnnnnnnnnnnnnnnnnnnnnnnnnnnnnnnnnnnnnnnnnnnnnnnnnnnnnnnnnnnnnnnnnnnnnnnnnnnnnnnnnnnnnnnnnnnnnnnnnnnnnnnnnnnnnnnnnnnnnnnnnnnnnnnnnnnnnnnnnnnnnnnnnnnnnnnnnnnnnnnnnnnnnnnnnnnnnnnnnnnnnnnnnnnnnnnnnnnnnnnnnnnnnnnnnnnnnnnnnnnnnnnnnnnnnnnnnnnnnnnnnnnnnnnnnnnnnnnnnnnnnnnnnnnnnnnnnnnnnnnnnnnnnnnnnnnnnnnnnnnnnnnnnnnnnnnnnnnnnnnnnnnnnnnatgcgcagcttgagcctgcttctcttgaagccatggatgATCGTGTCCTTGCTTTTCCAGGAAAACAAAAAGATGCCCTCAGCCTTCTGCCCTCGTGTCTCCGTGCGTTACTTGTGCAGCGTGGTCCTGCGCCGATAGATCAGTATGTATGnnnnnnnnnnnnnnnnnnnnnnnnnnnnnnnnnnnnnnnnnnnnnnnnnnnnnnnnnnnnnnnnnnnnnnnnnnnnnnnnnnnnnnnnnnnnnnnnnnnnnnnnnnnnnnnnnnnnnnnnnnnnnnnnnnnnnnnnnnnnnnnnnnnnnnnnnnnnnnnnnnnnnnnnnnnnnnnnnnnnnnnnnnnnnnnnnnnnnnnnnnnnnnnnnnnnnnnnnnnnnnnnnnnnnnnnnnnnnnnnnnnnnnnnnnnnnnnnnnnnnnnnnnnnnnnnnnnnnnnnnnnnnnnnnnnnnnnnnnnnnnnnnnnnnnnnnnnnnnnnnnnnnnnnnnnnnnnnnnnnnnnnnnnnnnnnnnnnnnnnnnnnnnnnnnnnnnnnnnnnnnnnnnnnnnnnnnnnnnnnnnnnnnnnnnnnnnnnnnnnnnnnnnnnnnnnnnnnnnnnnnnnnnnnnnnattcatattagaatttcgagtttgtacccagaaagcgtaacatctgcttttttgnnnnnnnnnnnnnnnnnnnnnnnnnnnnnnnnnnnnnnnnnnnnnnnnnnnnnnnnnnnnnnnnnnnnnnnnnnnnnnnnnnnnnnnnnnnnnnnnnnnnnnnnnnnnnnnnnnnnnnnnnnnnnnnnnnnnnnnnnnnnnnnnnnnnnnnnnnnnnnnnnnnnnnnnnnnnnnnnnnnnnnnnnnnnnnnnnnnnnnnnnnnnnnnnnnnnnnnnnnnnnnnnnnnnnnnnnnnnnnnnnnnnnnnnnnnnnnnnnnnnnnnnnnnnnnnnnnnnnnnnnnnnnnnnnnnnnnnnnnnnnnnnnnnnnnnnnnnnnnnnnnnnnnnnnnnnnnnnnnnnnnnnnnnnnnnnnnnnnnnnnnnnnnnnnnnnnnnnnnnnnnnnnnnnnnnnnnnnnnnnnnnnnnnnnnnnnnnnnnnnnnnnnnnnnnnnnnnnnnnnnnnnnnnnnnnnnnnnnnnnnnnnnnnnnnnnnnnnnnnnnnnnnnnnnnnnnnnnnnnnnnnnnnnnnnnnnnnnnnnnnnnnnnnnnnnnnnnnnnnnnnnnnnnnnnnnnnnnnnnnnnnnnnnnnnnnnnnnnnnnnnnnnnnnnnnnnnnnnnnnnnnnnnnnnnnnnnnnnnnnnnnnnnnnnnnnnnnnnnnnnnnnnnnnnnnnnnnnnnnnnnnnnnnnnnnnnnnnnnnnnnnnnnnnnnnnnnnnnnnnnnnnnnnnnnnnnnnnnnnnnnnnnnnnnnnnnnnnnnnnnnnnnnnnnnnnnnnnnnnnnnnnnnnnnnnnnnnnnnnnnnnnnnnnnnnnnnnnnnnnnnnnnnnnnnnnnnnnnnnnnnnnnnnnnnnnnnnnnnnnnnnnnnnnnnnnnnnnnnnnnnnnnnnnnnnnnnnnnnnnnnnnnnnnnnnnnnnnnnnnnnnnnnnnnnnnnnnnnnnnnnnnnnnnnnnnnnnnnnnnnnnnnnnnnnnnnnnnnnnnnnnnnnnnnnnnnnnnnnnnnnnnnnnnnnnnnnnnnnnnnnnnnnnnnnnnnnnnnnnnnnnnnnnnnnnnnnnnnnnnnnnnnnnnnnnnnnnnnnnnnnnnnnnnnnnnnnnnnnnnnnnnnnnnnnnnnnnnnnnnnnnnnnnnnnnnnnnnnnnnnnnnnnnnnnnnnnnnnnnnnnnnnnnnnnnnnnnnnnnnnnnnnnnnnnnnnnnnnnnnnnnnnnnnnnnnnnnnnnnnnnnnnnnnnnnnnnnnnnnnnnnnnnnnnnnnnnnnnnnnnnnnnnnnnnnnnnnnnnnnnnnnnnnnnnnnnnnnnnnnnnnnnnnnnnnnnnnnnnnnnnnnnnnnnnnnnnnnnnnnnnnnnnnnnnnnnnnnnnnnnnnnngagaaggagacgcaggacagtctgcaggtttcaccgcagcaacgacgtgacgctcgcacctttgctgcgtgcgcgtttgctgcgtgcgatcgggtgcctgcnnnnnnnnnnnnnnnnnnnnnnnnnnnnnnnnnnnnnnnnnnnnnnnnnnnnnnnnnnnnnnnnnnnnnnnnnnnnnnnnnnnnnnnnnnnnnnnnnnnnnnnnnnnnnnnnnnnnnnnnnnnnnnnnnnnnnnnnnnnnnnnnnnnnnnnnnnnnnnnnnnnnnnnnnnnnnnnnnnnnnnnnnnnnnnnnnnnnnnnnnnnnnnnnnnnnnnnnnnnnnnnnnnnnnnnnnnnnnnnnnnnnnnnnnnnnnnnnnnnnnnnnnnnnnnnnnnnnnnnnnnnnnnnnnnnnnnnnnnnnnnnnnnnnnccgcatatctgctcagcctggagtgtcctgcgagaatgcgcgggaagGAAAAAAAATGCAGGAACAGTTTGAAATATTCGTAACACGCGTGCGCAGGGTCGCCGCACAATTGCCTGTGCacggcatacgcgaccttgcggtgaacggacgcgACTTGATAGnnnnnnnnnnnnnnnnnnnnnnnnnnnnnnnnnnnnnnnnnnnnnnnnnnnnnnnnnnnnnnnnnnnnnnnnnnnnnnnnnnnnnnnnnnnnnnnnnnnnnnnnnnnnnnnnnnnnnnnnnnnnnnnnnnnnnnnnnnnnnnnnnnnnnnnnnnnnnnnnnnnnnnnnnnnnnnnnnnnnnnnnnnnnnnnnnnnnnnnnnnnnnnnnnnnnnnnnnnnnnnnnnnnnnnnnnnnnnnnnnnnnnnnnnnnnnnnnnnnnnnnnnnnnnnnnnnnnnnnnnnnnnnnnnnnnnnnnnnnnnnnnnnnnnnnnnnnnnnnnGTCGATGGGGAAGAAGGAGGAAGGCGGGTGATGTCTGTAGCAGAACCGCCGGGTGAAGGGGAAGACGCCGCCGTGTGCGCGCGAGCTCCAGCGTAATCGTGCAAACTTCCGCCACGATTAAGACATGCAACGCACTCCTCTGCGGCACGTACAGACAACGCATGGGTAACTACATACTGAGCAACGCTTACACACAACTGCATGTCGGTGAGTGACAGAAGTGCGCGCGCATGCCCTGCGCTCAGCGTTCGAGACGaaagcgactgctgaacttcaggaggcAGTTTTAAAAGACGCAGCGCATTGGTAATGGTACTGCGGTTTTTTCCAACCCGCTgtgnnnnnnnTTCATGACTTAAATTACCCAGATCCATGATATGTTGATAGGCGCGCGCCTCTTCCAGGGGATTCAGGTTTTCTCGCTGAACATTTTcaatgagcgcgatggcaagctttttttcatgatcgcaggtgcgcacgataacaggtatccgattnnnnnnnnnnnnnnnnnnnnnnnnnnnnnnnnnnnnnnnnnnnnnnnnnnnnnnncttccgtcctggtttttttccgcaaggactggctggattaccccgtgctcacgaatagacgcggcaagctcctcgagagattcctgcgcaaaggtgcgacgcgcctgatgnggattcgcctnnnnnnnnnnnnnnnnnnnnnAGTGTACAGtctgcacaccgcctgaatcacgaacatcgtatcgatctgagctttcttgcagcagcgcgtcaatgcctttgcctAATTTATCnnnnnnnnnnnnnnnnnnnnnnnnnnnnnnnnnnnnnnnnnnnnnnnnnnnnnnnnnnnnnnnnnnnnnnnnnnnnnnnnnnnnnnnnnnnnnnnnnnnnnnnnnnnnnnnnnnnnnnnnnnnnnnnnnnnnnnnnnnnnnnnnnnnnnnnnnnnnnnnnnnnnnnnnnnnnnnnnnnnnnnnnnnnnnnnnnnnnnnnnnnnnnnnnnnnnnnnnnnnnnnnnnnnnnnnnnnnnnnnnnnnnnnnnnnnnnnnnnnnnnnnnnnnnnnnnnnnnnnnnnnnnnnnnnnnnnnnnnnnnnnnnnnnnnnnnnnnnnnnnnnnnnnnnnnnnnnnnnnnnnnnnnnnnnnnnnnnnnnnnnnnatgtgaggatacccagcgagggcggacaatcaatcaggataaagtcgtacgtgtcttttacttctgccaatatctttttaaggtnnnnnnnnnnnnnnnnnnnnnnnnnnnnnnnnnnnnnnnnnnnnnnnnnnnnnnnnnnnnnnnnnnnnnnnnnnnnnnnnnnnnnnnnnnnnnnnnnnnnnnnnnnnnnnnnnnnnnnnnnnnnnnnnnnnnnnnnnnnnnnnnnnnnnnnnnnnnnnnnnnnnnnnnnnnnnnnnnnnnnnnnnnnnnnnnnnnnnnnnnnnnnnnnnnnnnnnnnnnnnnnnngcgctaaatacgcacccaagttaatggcagnnnnnnnnnnnnnnnnnnnnnnnnnnnnnnnnnnnnnnnnnnnnnnnnnnnnnnnnnnnnnggaGTGTACTCCACTTTCTAAATAAATAAAAGGACTTCGAAAGCGCGTCTGAATGGGAAGCTATGCGATGGGGTAAATCCTGGTAAGGAGATACGGTGTGCAAAAGCTAGAGTGATACGATGAACTGTGCTACCTTACGCGGAGGTGATCCTTTTTCTTATAAAAAAACATTGCATTGattgtggtgaagcgTTTTTCGGTATTGTAGTGCTCAATGCGTTGTGTCTTGCAGGGGTAgggtacagtctgttatggcaccaagggcctggtcggagcgtgttgtttgtcttagtactggctacgctgtacgcatgtctgtgcgcgttctgtgtcgttcgcggagnnnnnnnnnGTGATACTCTGGCAGATACGAATCTACGCGTCTTCACACACGCnnnnnnnnnnnnnnnnnnnnnnnnnnnnnnnnnnnnnnnnnnnnnnnnnnnnnnnnnnnnnnnnnnnnnnnnnnnnnnnnnnnnnnnnnnnnnnnnnnnnnnnnnnnnnnnnnnnnnnnnnnnnnnnnnnnnnnnnnnnnnnnnnnnnnnnnnnnnnnnnnnnctcctgtGGTTTGTGCCGGTGCGTGCACGCTACAGAACGCATTTTCGCCAATGCGTATATCTCTCGGCACGAGTGTTTTTTGAGCACCCGTGTATTACCTTCCTTATGGTTTTGTACAGCATGGGTGTGCTCGCGCTGAGTGTACCAATGGnnnnnnnnnnnnnnnnnnnnnnnnnnnnnnnnnnnnnnnnnnnnnnnnnnnnnnnnnnnnnnnnnnnnnnnnnnnnnnnnnnnnnnnnnnnnnnnnnnnnnnnnnnnnnnnnnnnnnnnnnnnnnnnnnnnnnnnnnnncatgtgccagatgcgtgcacaATCCCGCGCGCACACCGTCGGCGAGCTCTTCTCTCCGTGGAAATCGTAATGTTCATTGTGTTCGAGAGAAATAACAACCCCGCAGTCTATGAGGGGGTGCCGTACTCAGCAGGATTTTGGTATATGTGCTCAAGCGCGATCTGTGCTTGTGcaaacagcatgatactttctgtcgcaGTCTGATAACGCTTCTCAGCAACGACACGCACAATGCCGCAGTTAATGATGAGCATTGCACAGGTGCGGCACGGTGTCATGGTACAGTAGAGTGTTGCGCCCTCTAGACCGATGCCCAAACGCGCTGCCTGGCAGAGGGCGTTTTGCTCnnnnnnnnnnnnnnnnnnnnnnnnnnnnnnnnnnnnnnnnnnnnnnnnnnnnnnnnnnnnnnnnnnnnnnCCatgttcGTCACAATGCAGGAGACCTCGCGGTGCGCCTGCATACCCAGTTACAAGCAGATGGTGATCGCGCGCTATGACGCAGCCTGCGCGTCCGCGATCGCAGGTGGCACGCTTGGCAATTGCATGACACACTTCCATAAAATACTCATCCCAACTcnnnnnnnnnnnnnnnnnnnnnnnnnnnnnnnnnnnnnnnnnnnnnnnnnnnnnnnnnnnnnnnnnnnnnnnnnnnnnnnnnnnnnnnnnnnnnnnnnnnnnnnnnnnnnnnnnnnnnnnnnnnnnnnnnnnnnnnnnnnnnnnnnnnnnnnnnnnnnnnnnnnnnnnnnnnnnnnnnnnnnctcctgtgccagcgcaatggatgcagtgtttgtccccagggactgcgtccgtgtaacagttcttttgcgcgtggtcgtttgcgccgttttgagaaaatgtcgataacnnnnnnnnnnnnnnnnnnnnnnnnnnnnnnnnnnnnnnnnnnnnnnnnnnnnnnnnnnnnnnnnnnnnnnnnnnnnnnnnnnnnnnnnnnnnnnnnnnnnnnnnnnnnnnnnnnnnnnnnnnnnnnnnnnnnnnnnnnnnnnnnnnnnnnnnnnnnnnnnnnnnnnnnnnnnnnnnnnnnnnnnnnnnnnnnnnnnnnnnnnnnnnnnnnnnnnnnnnnnnnnnnnnnnnnnnnnnnnnnnnnnnnnnnnnnnnnnnnnnnnnnnnnnnnnnnnnnnnnnnnnnnnnnnnnnnnnnnnnnnnnnnnnnnnnnnnnnnnnnnnnnnnnnnnnnnnnnnnnnnnnnnnnnnnnnnnnnnnnnnnnnnnnnnnnnnnnnnnnnnnnnnnnnnnnnnnnnnnnnnnnnnnnnnnnnnnnnnnnnnnnnnnnnnnnnnnnnnnnnnnnnnnnnnnnnnnnnnnnnnnnnnnnnnnnnnnnnnnnnnnnnnnnnnnnnnnnnnnnnnnnnnnnnnnnnnnnnnnnnnnnnnnnnnnnnnnnnnnnnnnnnnnnnnnnnnnnnnnnnnnnnnnnnnnnnnnnnnnnnnnnnnnnnnnnnnnnnnnnnnnnnnnnnnnnnnnnnnnnnnnnnnnnnnnnnnnnnnnnnnnnnnnnnnnnnnnnnnnnnnnnnnnnnnnnnnnnnnnnnnnnnnnnnnnnnnnnnnnnnnnnnnnnnnnnnnnnnnnnnnnnnnnnnnnnnnnnnnnnnnnnnnnnnnnnnnnnnnnnnnnnnnnnnnnnnnnnnnnnnnnnnnnnnnnnnnnnnnnnnnnnnnnnnnnnnnnnnnnnnnnnnnnnnnnnnnnnnnnnnnnnnnnnnnnnnnnnnnnnnnnnnnnnnnnnnnnnnnnnnnnnnnnnnnnnnnnnnnnnnnnnnnnnnnnnnnnnnnnnnnnnnnnnnnnnnnnnnnnnnnnnnnnnnnnnnnnnnnnnnnnnnnnnnnnnnnnnnnnnnnnnnnnnnnnnnnnnnnnnnnnnnnnnnnnnnnnnnnnnnnnnnnnnnnnnnnnnnnnnnnnnnnnnnnnnnnnnnnnnnnnnnnnnnnnnnnnnnnnnnnnnnnnnnnnnnnnnnnnnnnnnnnnnnnnnnnnnnnnnnnnnnnnnnnnnnnnnnnnnnnnnnnnnnnnnnnnnnnnnnnnnnnnnnnnnnnnnnnnnnnnnnnnnnnnnnnnnnnnnnnnnnnnnnnnnnnnnnnnnnnnnnnnnnnnnnnnnnnnnnnnnnnnnnnnnnnnnnGTACTGCGCACCGGCCTGCGTACGCAGGGTGAATGTATCCCCCACGCGTGCGCGTCGAACACGCACCATGTAATGAAAATCnnnnnnnnnnnnnnnnnnnnnnnnnnnnnnnnnnnnnnnnnnnnnnnnnnnnnnnnnnnnnnnnnnnnnnnnnnnnnnnnnnnnnnnnnnnnnnnnnnnnnnnnnnnnnnnnnnnnnnnnnnnnnnnnnnnnnnnnnnnnnnnnnnnnnnnnnnnnnnnnnnnnnnnnnnnnnnnnnnnnnnnnnnnnnnnnnnnnnnnnnnnnnnnnnnnnnnnnnnnnnnnnnnnnnnnnnnnnnnnnnnggggtataaCAAGGGCGCACGCGTAGCGAACTGGACGTTCAAGATACCTTCTTTTTTTCCTGTATTCCCTCTTGCAATTCTTTGACCGTTTTAGTACGCGCCACCAGCGCGTGAATATCCTGTGTGCGTATCAACTCCACCGCCTTTCGCAgctgttcatnnnnnnnnnnnnnnnnnnnnnnnnnnnnnnnnnnnnnnnnnnnnnnnnnnnnnnnnnnnnnnnnnnnnnnnnnnnnnnnnnnnnnnnnnnnnnnnnnnnnnnnnnnnnnnnnnnnnnnnnnnnnnnnnnnnnnnnnnnnnnnnnnnnnnnnnnnnnnnnnnnnnnnnnnnnnnnnnnnnnnnnnnnnnnnnnnnnnnnnnnnnnnnnnnnnnnnnnnnnnnnnnnnnnnnnnnnnnnnnnnnnnnnnnnnnnnnnnnnnnnnnnnnnnnnnnnnnnnnnnnnnnnnnnnnnnnnnnnnnnnnnnnnnnnnnnnnnnnnnnncattgaggtcaaagacttgctgcacgactccctttccgtatgtggtttggcnnnnnnnnnnnnnnnnnnnnnnnnnnnnnnnnnnnnnnnnnnnnnnnnnnnnnnnnnnnnnnnnnnnnnnnnnnnnnnnnnnnnnnnnnnnnnnnnnnnnnnnnnnnnnnnnnnnnnnnnnnnnnnnnnnnnnnnnnnnnnnnnnnnnnnnnnnnnnnnnnnnnnnnnnnnnnnnnnnnnnnnnnnnnnnnnnnnnnnnnnnnnnnnnnnnnnnnnnnnnnnnnnnnnnnnnnnnnnnnnnnnnnnnnnnnnnnnnnnnnnnnnnnnnnnnnnnnnnnnnnnnnnnnnnnnnnnnnnnnnnnnnnnnnnnnnnnnnnnnnnnnnnnnnnnnnnnnnnnnnnnnnnnnnnnnnnnnnnnnnnnnnnnnnnnnnnnnnnnnnnnnnnnnnnnnnnnnnnnnnnnnnnnnnnnnnnnnnnnnnnnnnnnnnnnnnnnnnnnnnnnnnnnnnnnnnnnnnnnnnnnnnnnnnnnnnnnnnnnnnnnnnnnnnnnnnnnnnnnnnnnnnnnnnnnnnnnnnnnnnnnnnnnnnnnnnnnnnnnnnnnnnnnnnnnnnnnnnnnnnnnnnnnnnnnnnnnnnnnnnnnnnnnnnnnnnnnnnnnnnCTTCCACGTAGGCGGGTTTGCCCGGAGTGGATGTTCTGGACTTAGAAATGACAACGCCGATGCCGCCAAAGAAACCCTTGGTAGTGTCTTGGAGATTAACGCCGCTGATGCTGTCGCTTTCGACGAAGGTTGTGTAAGGGTCCTGAAGAGAGTTAATCATGCCTTCTAGTGCCCCCTTGTAGAGGATGTGGGGGTCTACCTCGTCGACGTAGTATTTGCGGAGGAATTCGTAGACATCCTGCACCGTCTGCATGCGCTCGTCTTCCTCGGAAGACTGTGGCAAATAAGCGGCAGTCCATGTGGGGAagggagctgcgctGGTGATCAGACAACAGacgaggagcgcaancagTCTCAGAACACTCATGGGCGGATGATGGAATGCGTGCCCGTCTGTGTCAAGAATCTGCGTTGGACAGTTTCCTTTCCATGCGACTAGACGGAGGAGGGCACTGGGGGAGGTGCAGGCGCGCCCCAGACCCGCTCTGGGAGGAGCAGGATATAGCGTGACTTCCGTTTnnnnnnnnnnnnnnnnnnncttcgtttttcacccctgtgcatgGGGGGGGGgaGAGAGAGAGAGAAGTTTCGTTCATCCTCGCACCCGTTGCGTTGCGGTGCGTGCAAGGACTGTGCTACAGTGCCGGCCGATGGGGACCGTGATCATCGCTCTTGATGGACCTGCAGGCTCTGGGAAGAGCAGCGTCTGTCGTCtgctcgcgtctcgCCTTGGCGcgcaATGTTTGAACACGGGTTCTTTCTACCGTGCATTTACCCTCGCCGCATTGCGTAGGGTATCGGAGTTGGCCGTGCAAGCGTGCTCTCCTTCTCCGGaccCTGATGCGGCGGTCGGGTGCGCGGCTGTTCCACACGCAACAAATCTGGACACATCATATGCTCCTCTGACGGCCCAGAAGAAGGTTGCACTTTTTGATGAAGCGTATTGGGTTTCGTTTGCGCGCACAGTTGCGCTTTCTTATCGTGCGGGTGTGATGTACGTGGGCGAAGAGAACGTGGAGTCACTGCTGnnnnnnnnnnnnnnnnnnnnnnnnnnnnnnnnnnnnnnnnnnnnnnnnnnnnnnnnnnnnnnnnnnnnnnnnnnnnnnnnnnnggccgtttgtggtgcgcgggtaGTTTGTGAAGGGCGTGATCTAACGacggttgtgtttgtggatgcggacttgaagtgctaCCTTGACGCTTCTATTGAGGCGCGTGTGGCGCGTCGTTGGGCGCAGGGAACGAGCCGGTTATCGAAGCAGGAACTCGAGCAGCGCATGCGCGCGCGTGACGCACACGACAGGGCGCGCACCGTGGGGGGGCTCAGGTGTGCGCCTGATGCGCTGTACGTGGATACTTCTTGCTTGACCATTGAGGAGGnTTGTGAAAGAATCGCGCGCGAGGCGCACCGCAGAGCTTTGTGGggaggagagcgttcagtggaaaatcaggaggggaagggtnnnnnnnnnnnnnnnnnnnnnnnnnnnnnnnnnnnnnnnnnnnnnnnnnnnnnnnnnnnnnnnnnnnnnnnnnnnnnnnnnnnnnnnnnnnnnnnnnnnnnnnnnnnnnnnnnnnnnnnnnnnnnnnnnnnnnnnnnnnnnnnnnnnnnnnnnnnnnnnnnnnnnnnnnnnnnnnnnnnnnnnnnnnnnnnnnnnnnnnnnnnnnnnnnnnnnnnnnnnnnnnnnnnnnnnnnnnnnnnnnnnnnnnnnnnnnnnnnnnnnnnnnnnnnnnnnnnnnnnnnnnnnnnnnnnnnnnnnnnnnnnnnnnnnnnnnnnnnnnnnnnnnnnnnnnnnnnnnnnnnnnnnnnnnnnnnnnnnnnnnnnnnnnnnnnnnnnnnnnnnnnnnnnnnnnnnnnnnnnnnnnnnnnnnnnnnnnnnnnnnnnnnnnnnnnnnnnnnnnnnnnnnnnnnnnnnnnnnnnnnnnnnnnnnnnnnnnnnnnnnnnnnnnnnnnnnnnnnnnnnnnnnnnnnnnnnnnnnnnnnnnnnnnnnnnnnnnnnnnnnnnnnnnnnnnnnnnnnnnnnnnnnnnnnnnnnnnnnnnnnnnnnnnnnnnnnnnnnnnnnnnnnnnnnnnnnnnnnnnnnnnnnnnnnnnnnnnnnnnnnnnnnnnnnnnnnnnnnnnnnnnnnnnnnnnnnnnnnnnnnnnnnnnnnnnnnnnnnnnnnnnnnnnnnnnnnnnnnnnnnnnnnnnnnnnnnnnnnnnnnnnnnnnnnnnnnnnnnnnnnnnnnnnnnnnnnnnnnnnnnnnnnnnnnnnnnnnnnnnnnnnnnnnnnnnnnnnnnagttnggcgtgaatgATGTGGTCAAGGGGCGTGTAACGAAAATCGCTGATTTCGGTGCGTTCATTGAACTTGCTGAGGGTATCGAGGggcttgcgcatattagtgagttcagctgggtaaagaaaacgagtaaacccagtgacatggttaaaattggggatgaagtnnnnnnnnnnnnnnnnnnnnnnnnnnnnnnnnnnnnnnnnnnnnnnnnnnnnnnnnnnnnnnnnnnnnnnnnnnnnnnnnnnnnnnnnnnnnnnnnnnnnnnnnnnnnnnnnnnnnnnnnnnnnnnnnnnnnnnnnnnnnnnnnnnnnnnnnnnnnnnnnnnnnnnnnnnnnnnnnnnnnnnnnnnnnnnnnnnnCGATGATCTGTCCTGGGTTAAGCGGACGCGTCCTGCAGACCATGAGCTTGAGGTAGGTAAAGAAATCGAGTGTATGGTTATCGAGTGTGATCCGCAAGCGCGCCGTATTCGGcttggtgttaagcagttgagcgacaatccgtggcaggtgnnnnnnnnnnnnnnnnnnnnnnnnnntaccgtggaggggGAAGTATCTTCCGTGACGGATTTTGGGATTTTCGTGCGTGTTCCCGGTGGCGTTGAGGGGCTTGTTCGCAAGCAGCATCTGGTTGAGAATCGGGATGGGGATCCGGGTGAAGCGCTAAGGAAATACGCAGTGGGGGACCGGGTCAAGGCAGTGATCGTAGATATGAACGTGAAGGACAGGAAGgttgctttttctgttagagactatcaaagnnnnnnnnnnnnnnnnnnnnnnnnnnnnnnnnnnnnnnnnnnnnnnnnnnnnnnnnnnnnnnnnnnnnnnnnnnnnnnnnnnnngaggcaaacgtctGGAAAGTGAGTGGGTCGGTGTGTTGCGCGTGTGATACGCGGTTCTTTCGGGCGCAGTGGGTTGGCTGCGGTTTTGGTGGCTCCTCCtcggttgttgtgggtgcttctccgcaaggtcttttgcgtgtgaggtaggtgnnnnnnnnnnnnnnnnnnnnnnnnnnnnnnnnnnnnnnnnnnnnnnnnnnnnnnnnnnnnnnnnnnnnnnnnnnnnnnnnnnnnnnnnnnnnnnnnnnnnnnnnnnnnnnnnnnnnnnnnnnnnnnnnnnnnnnnnnnnnnnnnnnnnnnnnnnnnnnnnnnnnnnnnnnnnnnnnnnnnnnnnnnnnnnnnnnnnnnnnnnnnnnnnnnnnnnnnctatcctcggggggctagggctaagtgcagggtgtttgctggttatgacgcgtttacgcgcgcgtgcttcagcnnnnnnnnnnnnnnnnnnnnnnnnnnnnnnnnnnnnnnnnnnnnnnnnnnnnnnnnnnnnnnnnnnnnnnnnnnnnnnnnnnnnnnnnnnnnnnnnnnnnnnnnnnnnnnnnnnnnnnnnnnnnnnnnnnnnnnnnnnnnnnnnnnnnnnnnnnnnnnnnnnnnnnnnnnnnnnnnnnnnnnnnnnnnnnnnnnnnnnnnnnnnnnnnnnnnnnnngnnnnnnnnnnnnnnnnnnnnnnnnnnnnnnnnnnnnnnnnnnnnnnnnnnnnnnnnnnnnnnnnnnnnnnnnnnnnnnnnnnnnnnnnnnnnnnnnnnnnGCGCGGATGAGCGTGGTCGCTTCGAGGAGGCGCGTGAGCTGTATCAGAGGAGTGCACGCGTGCAGGATTTTCCACTCGTTCCCCGTGnnnnnnnnnnnnnnggtcgcatggaagaggcgttagggcgcgcggctgctgctacagctgcctacatgcagctgtatgagcgctttccgctcaatggatgggcgnnnnnnnnnnnnnnnnnnnnnnnnnnnnnnnnnnnnnnnnnnnnnnnnnnnnnnnnnnnnnnnnnnnnnnnnnnnnnnnnnnnnnnnnnnnnnnnnnnnnnnnnnnnnnnnnnnnnnnnnnnnnnnnnnnnnnnnnnnnnnnnnnnnnnnnnnnnnnnnnnnnnnnnnnnnnnnnnnnnnnnnnnnnnnnnnnnnnnnnnnnnnnnnnnnnnnnnnnnnnnnnnnnnnncgtgcgtgaaggcgattttcgccggttcttttgatccgcctacctttgggcatttagatcttgttctgcgcgcgcgtnnnnnnnnnnnnnnnnnnnnnnnnnnnnnnnnnnnnnnnnnnnnnnnnnnnnnnnnnnnnnnnnnnnnnnnnnnnnnnnnnnnnnnnnnnnnnnnnnnnnnnnnnnnnnnnnnnnnnnnCGTCTTTCCTTGGCGTTCGCTTGTGGTTACGTACGCTCGAGATGTTGGTGCGCGTGTGCTGGTTCGCGGTGTGAGAAATGCTACTGATTTCTGTCAGGAGTTTGATCTTGCATGGGTTCATCGnnnnnnnnnnnnnnnnnnnnnnnnnnnnnnnnnnnnnnnnnnnnnnnnnnnnnnnnnnnnnnnnnnnnnnnnnnnnnnnnnnnnnnnnnnnnnnnnnnnnnnnnnnnnnnnnnnnnnnnnnnnnnnnnnnnnnnnnnnnnnnnnnnnnnnnnnnnnnnnnnnnnnnnnnnnnnnnnnnnnnnnnnnnnnnnnnnnnnnnnnnnnnnnnnnnnnnnnnnnnnnnnnnnnnnnnnnnnnnnnnnnnnnnnnnnnnnnnnnnnGTTCTCCTGCATGCGTGCCGATAAAGGGGCGGTGGGAACGGTTGCTGTGGTCTGTGCCTTTGATCTGAGCGATTATGCCTTTGTGCCCTTGTGTGGCACGCGTGAGCAGTCGTGTATGAAAGCAGTGCCGGGGTTCGTGCCGTGCGTGGAGGTGAGGGGCTGTGAGAGTACAGCCGGGGTGGGGCGGCGTTGCGCTTTCGAGCGTGCTGTTACAGCTGCTTACGCGTTGCCTGGTTGCCATCAGGTGTGTGTGTACGCGGATGCGGCGAGCGCCGCGAAGGTTGCGCGTTTTATGCAGTGCGCTACGCCTATTTTTCCGTCGCTGCGCGTGAACGTTCTGGATGACATGCGAGTGAGCGCCTTCTTCGCGCATGTTGCACGCGTATGTGCGGAATACGCGCAATTGGGTGAAGAACCTGAGGCTGTCTTTGTTCTGCGCGCAGAcgcgccctttattgannnnnnnnnnnnnnnnnnnnnnnnnnnnnnnnnnnnnnnnnnnnnnnnnnnnnnnnnntttgcagannnnnnnnnnnnnnnnnnntttgctgcagntgttgcgccaggacttttccccatccttgctactttgactcaggatgcacatatttgttttgacacttcgttcatttttgagagcattaaaacagacattaattcttttgatcnnnnnnnnnnnnnnnnnnnnnnnnnnnnnnnnnnnnnnnnnnnnnnnnnnnnnnnnnnnnnnnnnnnnnnnnnnnnnnnnnnnnnnnnnnnnnnnnnnnnnnnnnnnnnnnnnnnnnnnnnnnnnnnnnnnnnnnnnnnnnnnnnnnnnnnnnnnnnnnnnnnnnnnnnnnnnnnnnnnnnnnnnnnnnnnnnnnnnnnnnnnnnnnnnnnnnnnnnnGTATCTTTTCCTGCGCAGGTGGAGAATGCTTCACTGATGCCAAAAGAGGAAGCGTGTGCGTTGATCCGGCGCATTGCAGACTTTTCAGAAcgtgcgGTCATTTCGCnnnnnnnnnnnnGGGATCCGGTGTTGTATCCTGCACTGTGTGACGTCGTACGTGAGATTCTCAAGTATCCGGGTTTGnnngtgcTCATTGAGACAAGTGGGTTGGGGTGGCAGGAGTCTGTAGTGCGTGATTTGTGCGAATgtgcgcgcaacagtgcgcgcactccttttgccattggctggattGTGTTTTTAGATGCGGTGAGTTCAGGCATGTACAGnCAGGTACACCGTGTGTCGCTGTCAGAAGCGGAATTTTTTCTAAAAGAAGCGACGGAATTTGCAATGCAGGTACATGCGCAATGTCCGGGCGTGCTGTGGCCACAGATTTTCCGTATGAATGAAAACGAAAAAGAGCTCGAGCCCTTTTACcgcacgtggaaggnnnnnnnnnnnnnnnnnnnnnnnnnnnnnnnnnnnnnnnnnnnnnnnnnnnnnnnnnnnnnnnnnnnnnnnnnnnnnnnnnnnttggagaggtatccgtgttggcatctaaaacgagatatgattattttcactgatggtcgagttccgctGTGCAAAGAAGATGTGCACTGTCGCCACGCGCTGGGCAATGCTTTccagcaggatcttgcacagatttggcggcggggacaggatgtgtatcttcagcacgttcgtgcaGTACATGAAggattatgtgggcagtgtgatgagtactatacctataatttttaacgagaaaaatgttgcacacacgGTGGTGGGTGGTCAACTCtgtccggtggcgtctgctTTTTTAGGTGCAGTAGTACTCAATCGGGGAGTGCGGTGGAATCGGGCAGAGTTTTTTGCGCAACTGACTACccttggcatcgcgcccattgtatcggtggagcggagtgcagcagCTCCAGATGTTACCGGTCTTGCTGAGCGTTTCCCCTTCGTTAAGTTTATCACACCGCTTGAGTGTATTTCGGTTGGAGAAATGATCAATTTAGGTGTTGCAGAACttgatgtcatgtatgTTTTAGTTCTGTGGAGCGACATGCGCATAGATCCGCAGGGTATGAGCCAGCACATACAGGATATCTTGCGCACGAATACGCACATGTGTATTGCGCCGCAATTGTGTTCTTCCCACAGTACTGTACnnnnnnnnnnnnnnnnnnnnnnnnnnnnnnnnnnnnnnnnnnnnnnnnnnnnnnnggaatgtacgactaaccatacgcctacgatataCCCGTATGATTTTGTTGGAATATACCATCGGGACCGGTTTATCCAATGCGGAGGTTTTGATTTTACCATCGCGAATGCATATTGGCAAAATCTTGATTTTGGATTGCGTTCTTAnnnnnnnnnnnngtgcatagaggttaatacgcaaataagactgttttatgaggctcttcccccgcaannnnnnncaagtgcagatagttcgtaccgcatcttttatcTGAAAAACCTTGCGCCACTCCTAGAGAAGGATAGGGCCTTTATTTCGGGCACGCAGtttttttcattttttagaaattctggtctgcatctaatagaggcgtaccgtctctttcgggannnnnnnnnnnnnnnnnnnnnnnnnnnnnnnnnnnnnnnnnnnnnnnnnnnnnnnnnnnnnnnnnnnnnnnnnnnnnnnnnnnnnnnnnnnnnnnnnnnnnnnnnnnnnnnnnnnnnnnnnnnnnnnnnnnnnnnnnnnnnnnnnnnnnnnnnnnnnnnnnnnnnnnnnnnnnnnnnnnnnnnnnnnnnnnnnnnnnnnnnnnnnnnnnnnnnnnnnnnnnnnnnnnnnnnnnnnnnnnnnnnGATGAACATTCTAAAAAAGATTTTGAACCCGTTGCGCGCGCGCATGGGTTCTACTGTATTTCAGGATCGGCAGAAGannnnnnnnnnnnnnnnnnnnnnnnnnnnnnnnnnnnnnnnnnTTCGTTCCCAATTCGAACCGTCGTGCGGGTTACCGGAGATAACCCTTTTTTGTTTCACGAAGCAGCTGCTGCAGCGCTCCTGCGTTATGCGGAACTGAACGAGCCAGATTATTTTACCTTCnnnnnnnnnccctacggcagcggagtggaaatattaaaggcgcgctcgttGCTTTTAGCAGATCGTCTTCCTTTGGAGGCGTACGACCGTGAGCATGTAGGACCTGCCTTACATCGCCGCCcgggtattttcgtgtgtgtgcgtgaacctgccgctgccgnnnnnnnnnACCCTGATGTGCGAGTAACGGTGGACACGCAGGAAGACTTTCAGCGAGCGCAGCATATGAnnnnnnnnnnnnnnnnnnnnnnnnnnnnnnnnnnnnnnnnnnnnnnnnnnnnnnnnnnnnnnnnnnnnnnnnnnnnnnnnnnnnnnnnntttgtaccctcagtgaaaataggacagggagcggggcatttatatcgggcggcgtatttagtcttgcgactgcaaggannnnnnnnnnnnnnnnnnnnnnnnnnnnnnnnnnnnnnnnnnnnnnnnnnnnnnnnnnnnnnnnnnnnnnnnnnnnnnnnnnnnnnnnnnnnnnnnnnnnnnnnnnnnnnnnnnnnnnnnnnnnnnnnnnnnnnnnnnnnnnnnnnnnnnnnnnnnnnnnnnnnnnnnnnnnnnnnnnnnnnGGATGAGGGGGGATCTGGACGTCTGAATGCGGATTACTTGATAGATGTATTTCCCGTCTTGCAAAGTCCGGGTCGAAGGTnnnnnnnnnnnnnnnnnnnnnnnnnnnnnnnnnnnnnnnnnnnnnnnnnnnnnnnnnnnnnnnnnnnnnnnnnnnnnnnnnnnnnnnnnnnnnnnnnnnnnnnnnnnnnnnnnnnnnnnnnnnnnnnnnnnnnnnnnnnnnnnnnnnnnnacggccgcgcGCGTTCGTGTGCTGAGCGGATAGCTGCATTAGGTTTTGACACCTCAGTGGTAGTTCCTGGTGAAGCGTCTGAACGGACGGACGGTAAGGTGCACATATACCCTTCTTTGCCTCATTTGAAAGAGCATCTCCACCGCTGGGACGTGGTAGTTacccattttggttttaccgcatttgaggctgcagctgcaggggcggcggtgttgcttgtttcaccaacgccctatcattttttactctnnnnnnnnnnnnnnnnnnnnnnnnnnnnnnnnnnnnnnnnnnnnnnnnnnnnnnnnnnnnnnnnnnnnnnnnnnnnnnnnnnnnnnnnnnnnnnnnnnnnnnnnnnntcgcagtgtcgtgatctggcagatgaaatcatgcgcgttgcgcgggggaaaaaacactgctgtcctttttgtggagaaatggtagcggtggatgttgtcggtcgtgcattacataaaaccnnnnnnnnnnnnnnnnnnnnnnnnnnnnnnnnnnnnnnnnnnnnnnnnnnnnnnnnnnnnnnnnnnnnnnnnnnnnnnnnnnnnnnnnnnnnnnnnnnnnnnnnnnnnnnnnnnnnnnnnnnnnnnnnnnnnnnnnnnnnnnnnnnnnnnnnnnnnnnnnnnnnnnnnnnnnnnnnnnnnnnnnnnnnnnnnnnnnnnnnnnnnnnnnnnnnnnnnnnnnnnnnnnnnnnnnnnnnnnnnnnnnnnnnnnnnnnnnnnnnnnnnnnnnnnnnnnnnnnnnnnnnnnnnnnnnnnnnnnnnnnnnnnnnnnnnnnnnnnnnnnnnnnnnnnnnnnnnnnnnnnnnnnnnntctgcattcctgcttgtgttgcaacgtttccattacttcctgagtgttttgactttgtgattcggcacagctttgccacgaatcgtccccaacaagtatctgtttacattcgagagtgtacgttctccgccgttaccctgtggtttgtgannnnnnnnnnnnnnnnnnnnnnnnnnnnnnnnnnnnnnnnnnnnnnnnnnnnnnnnnnnnnnnnnnnnnnnnnnnnnnnnnnnnnnnnnnnnnnnnnnnnnnnnnnnnnnnnnnnnnnnnnnnnnnnnnnnnnnnnnnnnnnnnnnnnnnnnnnnnnnnnnnnnnnnnnnnnnnnnnnnnnnnnnnnnnnnnnnnnnnnnnnnnnnnnnnnnnnnnnnnnnnnnnnnnnnnnnnnnnnnnnnnnnnnnnnnnnnnnnnnnnnnnnnnnnnnnnnnnnnnnnnnnnnnnnnnnnnnnnnnnnngcattgagcaaatggttccaattaggagacactatggaggtgtacgcgataaagcaaggannnnnnnnnnnnnnnnnnnnnnnnnnnnnnnnnnnnnnnnnnnnnnnnnnnnnnnnnnnnnnnnnnnnnnnnnnnnnnnnnnnnnnnnnnnnnnnnnnnnnnnnnnnnnnnnnnnnnnnnnnnnnnnnnnnnnnnnnnnnnnnnnnnnnnnnnnnnnnnnnnnnnnnnnnnnnnnnnnnnnnnnnnnnnnnnnnnnnnnnnnnnnnnnnnnnnnnnnnnnnnnnnnnnnnnnnnnnnnnnnnnnnnnnnnnnnnnnnnnnnnnnnnnnnnnnnnnnnnnnnnnnnnnnnnnnnnnnnnnnnnnnnnnnnnnnnnnnnnnnnnnnnnnnnnnnnnnnnnnnnnnnnnnnnnnnnnnnnnnnGCTCGTGTTTTGGAATACGGCATGGCGCATCGCCTAGATTACCTAGnnnnnnnnnnnnnnnnnGTATATGCACcggactacagtgtccgtatacgggcgttccatgaatacaatcgtttggcccgtcgtggggggagcgcgcttatccctatctttcccatagaagcactatcaggcgatcggcagcgacaggttatgaaaatcctnnnnnnnnnnnnnnnnnnnnnnnnnnnnnnnnnnnnnnnnnnnnnnnnnnnnnnnnnnnnnggagtacactgtgaacactccatgccgcacctgattgatGTAATGCCCGAGGGAGTGGATAAAGGGAATGCCCTGCATATATTGTGCCAGTACTACGGCATTTCTTTGGAACAAGTATGCGTGTTTGGTGATCAGAGGAATGATATCGTCATGTTCAAAGCAGCGGGCGTTTCCTTTTGTATGCGTAATGGTCATGAGGAAGCAAAAAAACACGCAAGGTATATTGCCGAatctaatgatgatgcaggttttgcacaGGCTTTGAAGCGCTATGGGGTGGTAGAGTAAAGAGGGCagtgcttttttagggaaaaggaagaTCGCATGGTGCGTTCTGCGCGTTCAGAGTACAAGTGTCGCCTGTGTGCCcgttgtgatggatacgggtgtcgtggtcagttaccagggatggggggtgtttttaacagtgcgAATTTTATCGCGAACTGCCTTGCATGGAAAAAATATGTTCCGCAAAGGCATGAACTACCGGTCGTGCGTCTTGCGCCTATGACGGGTGCAGTTGAAAACGTCGGATATCCTGACGnnntctcgttttattatcggctcatcgaagcggtgtccggaacgggtgtcctgttgagtgtgggagatggttgtccggacatcaaacnnnnnnnnnnnnnnnnnnnnnnnnnnnnnnnnnnnnnnnnnnnnnnnnnnnnnnnnnnaccgtatgtnnnnnnnnnnnnnnnnnnnnnnnnnnnnnnnnnnnnnnnnnnnnnnnnnnnnnnnnnnnnngatatcgatgcgtaccaTATTGTGACCATGAGAGATAAAGTGCAGTTAGAGAAAAAGACTCCTACGCACCTTCGTGCAGTCAGACGTTTTGCAAAACTTCCTATCGTGGTGAAAGGAATTTTTGCGCCGCGCGATGTCGAATTAGTCAGAGAGTTGAAACCGGATGTCGCTATCGTATCAAATCATGGAGGCAGGGTTGAAACTGCGCGCGGAAGCACAGCGGACTTTCTTTTTGAATACGGTGGAGAGTTAGCGCGCTGTGCCGGTGAAGTGTGGGTAGACGGTGGTATTCGCngttatgcgcaTTtGTGCGCAGCACGGGAGTTGGGTGCGCAGCAGGTGCTGATAGGGCGTCCTTTTATCACAGCTCTTCTCAAAGGAGGAAAGGGTGGGGTGCAACTTCTCGTGCGGAATATGACGGATAGGTCCGTTCGGTGTGTATGATGGGAAGTGTGTTTTCTAGGAAGGGaatgctGtgTGTTCTTGCAGTCTGGTGCGCGGCAAGTGCGGCGTACCCTCTGCGCTTGCGCTACAAGTTTCGTAAGGGGGATACGCACCGTATCAACTCGCTGATACGCGAGGATGTGTTTGTGAACGATACGCTTGCTCACACGGCGGAGATTACGAATCGAATCACGGTGCATGTTTCCGAAGTACGTGTTGCACACGGGTCGGCGCCTGATGCGGCGCGGTATGTGTGTCACTTCATGACATCCGAGAAAAGTCCCAATAATACGTTTCGTTGGGGGAGGCACTACGAAAGTATTTTCTGGCGCGACGCATTTGGTGtgtatgannnnnnnnnnnnnnnnnnnnnnnnnnnnnnnnnnnnnnnnnnnnnnnnnnnnnnnTATGATATCGAagttggagATACCTGGGAGCATGAAGCAGAAGAAGCGCATGATCTGCGTGACGGCTTTGGTATCCAAACGCCCTTCATTGTGCCGTTTACCGTGTCGTATACCTACCGTGGTGAAGTTCAGCGTGGTAGTCGGCGCTATCATCACATTACTGCGGCGTATTCTATGagttatgagncgcccaaacggacacatggcgtgcagcgcaatgcaaaagaaggaatgtatccggtgcgtaCTACgggcgtttctaagcaaaatttgtattgggataacgaacttgggaacatagctgagtacgacgannnnnnnnnnnnnnnnnnnnnnnnnnnnnnnnnnnnnnnnnnnnnnnnnnnnnnnnnnnnnnnnnnnnnnnnnnnnnnnnnnnnnnnnnnnnnnnnnnnnnnnnnnnnnnnnnnnnnnnnnnnnnnnnnnnnnnnnnnnnnnnnnnnnnnnnnnnnnnnnnnnnnnnnnnnnnnnnnnnnnnnnnnnnGTATAGAAAATGTTCAATTTGACGCAGACTCTGCATCCCTTGCACCTTCTGAGTACGAGAAGCTGCGCAAAATAGCAGAACTTTTGCGCGCGTTTCCCGATCGAGAGTTGTTGGTGTCTGGACATGCGGCACGACGGGGGTCTGTGCAGGATCAACAGAGGATTTCTGAAGAGCGTGCTGATGTGGttgcacgttacctgcagnnnnnnnnnnnnnnnnnnnnnnnnnnnnnnnnnnnnnnnngttgtggtgnnnnnnnnnnnnnnnnnnnnnntgattcagaggatggtcgtagaaaaAACCGACGGGTAGAAATCACTATCATAAGTAAGTAAAGTGTTGCCCTGTGCGCATGACGCGTGTGCGTTCGCTGTGCCTTGCGTATGCAAGGTGTgttcgcttgggtgtgtttcgttGTATTAGCCTGCGCGGATGGGCTCCCTATCGTGCggttgagacggtgcttgtccagtccgcgttgacaagtgtggagagattgcatgagatgttcaGGGTTTACTGATTTGGCGATCGTCGCCTGTCCnnnnnnnnnncacttcgcggacgaaaCTATCAAACATTTGACGCGTGTGTGTGAACGCAAGTTTCACCAGAGAATGGACCGTTTGACGGACCGCTACGGTCTTGACTCGGGGTCGGTGATACGAGATGCGAACTTTTACCGGGACCTTTTTTCCACAGAGTnnnnnnnnnnnnnnnnnnnnnnnngttttaatccaccgcattttaaggtggatgcacaGTTTATCTGttttctaaatggtgagttaaaaacgcaaattaatgaatgtattcnnnnnnnnnnnnnnnnnnnnnnnnnnnnnnnnnnnnnnnnnnnnnnnntcctggtgaataacggaaaaagtaaaaaaatattctccgtgaatgatcacgtgATGATGCTCATTGTGACCATAGATGCAGTTCGGCACGCGGGAGCTGGGCGCGTTACCTTGGTTTTACCcacttatccgtatagccgtcagcacaaaannnnnnnnnnnnnnnnnnnnnnnnnnnnnnnnnnnnnnnnnnnnnnnnnnnnnnnnnnnnngaGTCACATTGTCACTCTTGATTTGCATTCACGTGAGATTGAAAATGCCTTTCATCGCACGCGGCTTGAAAATTTACACGCCAGTTATCAGATTATCCGGGAGTTGGCAAAGATTGAAAATCTTTCTGACCCCGATATCCCTTTTGTAGTAGTnnnnnnnnnnnnnnnnnnnnnnnnnnnnnnnnnnnnnnnnnnnnnnnnnnnnnnnnnnnnnnnnnnnnnnnnttataannnnnnnnnnnnnnnnnnnnnnnnnnnnnnnnnnnnnnnnnnnnnnnnnnnnnnnnnnnnnnnnnnnnnnnnnnnnnnGGGGAAGACGGCGTTTATTGCTGACGATATGCTTGGCAGCGGAGGTACGATGCTAAAGnnnnnnnnnnnnnnnnnnnnnnnnnnnnnnnnnnnnnnnnnnnnnnnnnnnnGTTTACCTTTCTTtACGGGAAATGCACTTGAGTTATTTGATGAAGCGTACGAGAAACGGTATTTCTCTCGGATTATTGGCACAAATGCGGTGTTCCACACACAGCTTTCGCACAAGCAGTGGTATACTGAAACCGATGTGTCAGGGTTGTTcgcgcgcgTCATCGCGCGCATTCATCATAATCAATCGTTGAGCAGTCTCTTGGATGATCGCAGTATCATCGAGCGACTCCTACACGCTCGCTTGTCCGTTGCGGGGACACCGCGCGCATAGGGTAGCGTCGCAACGGACATGGGTTCGGGGATCTTTGTCGCGGACATCGGTACnnnnnnnnnnnnnnnnnnnnnnnnnnnnnnnnnnnnnnnnnnnnnnnnnnnnnnnnnnnnnnnnnnnnnnnnnnnnnnnnnnnnnnnnnnnnnnnnnnnnnnnnnnnnnnnnnnnnnnnnnnngttgcgtgccgtgcatcacgttattgccattactatttcgggcaatggaccgagcgtcgttgccGTGCACAAGAAGAGTCATGCnnnnnnnnnnnnnnnnnnnnnnnnnnnnnnnnnnnnnnnnnnnnnnnnnnnnngtgtttctctttttttgccaaaggttttgctccttttgcagcgattgcatttttgtgcgcgtgatgtacagttttttcnnnnnnnnnnnnnnnnnnnnnnnnnnnnnnnnnnnnnnnnnnnnnnnnnnnnnnnnnnnnnnnnnnnnnnnnnnnnnnnnnnnnnnnnnnnnnnnnnnnnnnnnnnnnnngCGTTACCTGAAACGCTCTTTGCGCCGTTCGTGGCTCCTGGCTCAATTGTTGCCTCCTATCGGGGAATACCGGTAGTCTGCGGCGCGCCGGACTTTGCGGCAGCACTCATTGGAACAAATACGTTACATGCagggagcggatgcgatcgggnnnnnnnnnnnnnnnnnnnnnnnnnnnnnnnnnnnnnnnnnnnnnnnnnnnnnnnnnnnnnnnnnnnnnnnnnnnnnnnnnnnnnnnnnnnnnnnnnnnnnnnnnnnnnnnnnnnnnnnnnnnnnnnnnnnnnnnnnnnnnnnnnnnnnnnnnnnnnnnnnnnnnnnnnnnnnnnnnnnnnnnaacggatgggtcaaattatggcgttaccttttcagctgcatgatgcgtatccccccaccgtggtgggagaagggagacagctggtagaggatcttgcctttgaggtGTGTGCAGGTCTGGAGTATCTGGAGTCTGTGACCCAGTTGCAACCggtatacacnnnnnnnnnnnnnnnnnnnnnnnnnnnnnnnnnnnnnnnnnnnnnnnnnnnnnnnnnnnnnnnnngttttgtgttaccagaaattcatgacgcagaactgacaggaaacgcggcgctcgcgtgtgttgccttggattttgatgcggatatgcaaacagcagcgcaacggctgtgTCGTCTCAAGCGTGAGTTTATTCCCAACCGTGCCAGACACGAACAGTATGCACAGAAGCGTCTTTTGCGTGCTGCGCGTGAGGCACAGGGGnagtcagcgcaagatacgcaggaatatcgggtgttcagcacattctgaacaCTTGAGGACGGTGCGTGTTTGACGTTACGCTCTCGCTGTGCGCGAGTTTTGTCCACTAATTGGTGTCATCGGTCGAAGTGGTGCGGGGAAGAATGTGGTCTCGCGCCTGCTTGCAGAACGGGGATGTTACTGTATTGACGCGGATGCGCGCACGCGTGAACTTTTGGAATCTTACGGCGACTCTATAGTAGAGCGGTTTCAAGTGGCTGCGGCTGCGCGCGGCCTTTCGTTACGGCGTAAAGATGGCGGCCTGCACTCTGCGCATTTGGGTGTGTTACnnnnnnnnnnnnnnnnnnnnnnnnnnnnnnnnnnnnnnnnnttgttaccgaaagttacccgtcttttgtgtgaagacatagcgcgcgcgcaggctgcgcgtccaaaagcgatagtactgaatgcgccgacattgcataaaacagaactgttacaagcctgtagttttgtttnnnnnnnnnnnnnnnnnnnnnnnnnnnnnnnnnnnnnnnnnnnnnnnnnnnnnnnnnnnnnnnnnnnnnnnnnnnnnnnnnnnnnnnnnnnnnnnnnnnnnnnnnnnnnnnnnnnnnnnnnnnnnnnnnnnnnnnnnnnnnnnnnnnnnnnnnnnnnnnnnnnnnnnnnnnnnnnnnnnnnnnnnnnnnnnnnnnnnnnnnnnnnnnnnnnnnnnnnnnnnnnnnnnnnnnnnnnnnnnnnnnnnnnnnnnnnnnnnnnnnnnnnnnnnnnnnnnnnnnnnnnnnnnnnnnnnnnnnnnnnnnnnnnnnnnnnnnnnnnnnnnnnnnnnnnnnnnnnnnnnnnnnnnnnnnnnnnnnnnnnnnnnnnnnnnnnnnnGTACCTGAGCACCCGTCAGTGGATCCGAGCTTATGGCTAAAAAATCCTGAAAAAATTCCTGAGCTGGGGAAGACTCCTGCGCCAACACAGAGGGCAATTCCTGATCTCACGGTGGTAAAGGATGTCCCTACCGCTCGTGCGAAAAGTCTTGATGTTTCTCCTCCGGCGCGTGCnnnnnnnnnagacnnnnnnnnnnnnnnnnnnnnnnnnnnnnnnnnnnnnnnnnnnnnnnnnnnnnnnnnnnnnnnnnnnnnnnnnnnnnnnnnnnnnnnnnnnnnnnnnnnnnnnnnnnnnnnnnnnnctcgtacccgtgctccgcttgttgcacagcggnnnnnnnnnnnnnnnnnnnnnnnnnnnnnnnnnnnnnnnnnnnnnnnnnnnnnnnnnGTGCAAGCCGCTTCGCTCTCAAGTAAGCTCAACGCCGAGCGTGCTCGGGGTGTGCTnnnnnnnnnnnnnnnnnnnnnnnnnnnnnnnnnnnnnnnnnnnnnnnnnGGCTTGCGTCACCGTGTGCGTGTCGGTCCTTTTACTAATCGTACAGAAGCGGTATATTGGATGAAAAGCATCCGTGAGATCGCAGAGTTTAGCGGATGCTACGTTTTTCAAGAACGCGTGAAAAACTAGTCTTTCTCCTTCTCTCTGGGTGgtgcaatgcgtttnnnnnnnnnnnnnnnnnnnngtgctctggnnnnnnnnnnnnnngggtgccggtagggtgcaccctACGCGAGAGAGGGATTTTTGGGGGGAGGTGTTATGAACGGTGCGGTGTGTGTTCTCAGTGCGCTCATTGCAGTGTTTACTTGCTTTTCGTGTAGGCCTGCGGTGCAAGATGAGCGCGCGGTGCGTATTGCCGTTTTTGTCCCAGGTTTTCGtcacgacagtcctgtgtatgcaatGTTGTGTGACGGTGTTGAGCGTGCAGTTACGCAGGAAcgcgcnnnnnnnnnnnnnnnnnnnnnnnnnnnnnnnnnnnnnnnnnnnnnnnnnnnnnnnnngcgcgaaaagttggcgcatcttgctgcagaacagcgctatcgtttgattgtgnnnnnnnnnnnnnnnnnnnnnnnnnnnnnnnAGCCTATTTTGCGTCAATTTCCCCTGCAGCGGTTTTTGGTTCTAGATGCCTACGCGCCGCAGGAGCACtcgctgattacctttcgctataaccagtgggagcaagcctaccttgcaggacaCCTTTCCGCnnnnnnnnnnnnnnnnnnnnnnnnnnnnnnnnnnnnnnnnnnnnnnnnnnnnnnnnnnnnnnnnnnnnnnnnnnnnnnnnnnnnnnnnnnnnnnnnnnnnnnnnnnnnnnnnnnnnnnnnnnnnnnnnnnnnnnnnnnnnnnnnnnnnnnnnnnnnnnnnnnnnnnnnnnnnnnnnnnnnnnnnnnnnnnnnnnnnnnnnnnnnnnnnnnnnnnnnnnnnnnnnnnnnnnnnnnnnnnnnnnnnnnnnnnnnnnnnnnnnnnnnnnnnnnnnnnnnnnnnnnnnnnnnnnnnnnnnnnnnnnnnnnnnnnnnnnnnnnnnnnnnnnnnnnnnnnnnnnnnGTAGTTGGCAGTTCCGTTATGGAACAGGAGCGTCTTGCGTATGAGCAGACGCTGCGCTGCATTCGCGGTGAACTGCCATCTGCAGGAGCCTGGACATTGGGGGTGAAAGACGGGTACGTACGTTTCATTGAAGAGGATCCCTTGTACCTGCAAACGGTACCCGAACCGATTCGTGTGCGGCAGTCTGCGTTGCTCAGGCGTATTCAAAGCGGTGAGCTTACGTTGCCGGTGCGTTGATGATAGCTGAGCGCGGCGTTAGGGCATCTGCGCGCGGTGTGCTCTCGCTTCACCATATTGGGAAAACCTACCCGCGCGTGATGCCGCGTTCAAAGCGCGGGGTCTGGGGTATGTTCGGTCACCCTGGCAGGCGCGCAGTCGACGGTGCGCACACAGCGCATGGCCCGTGTTCGGGCGCGCGTGAGACGGACGCCnCTGAGCACAGCGTGCTCAGCGATGTGAATCTTTCCTTTTTTACCGGTGAAATTCACGCGTTGTTGGGAAAAAATGGTGCAGGAAAATCCACGCTTGCGCATATTCtttcgggcttttgcgtgccgacgcacgggcagctccgtctggatgGAAAGGAGCAGCGCTTCTCCGTACCCttcgatgcgctccgtgcAGGAATTGGCATAGTGCACCAGCAACCGGTATTtgcagaacgcgcaacagtatttgaaaatgtagtgatgggtagtgctgcactcnccggcgtgcgGTGGGTGCGCCGTGCACAGGTGAGAGAACGCATAGATCGCATTATCGCCCAGTGGCGTATGCCCTTGAAGAAGGAAGAATACGTTGCATGTTTGAGTGCAGACAAGCGGTTTTTTGTATCGTTGCTCTGTGTATtgTTTCGCAATCCGCGTTTCATCATCCTGGATGAGCCGCGGTGTGCTCCGGCGCAATCGCGTGcggttttcttttctcatcttgaagagttttttgtgcgttcttcgcacgcgccccgGTGTGGTGGCGGAGTGATAGTAGTGACgcaccgatnnnnnnatgcattgcgatgggcacagcgtatttctttgatagaagggggaaaagcgtgcagttttctgcgtacggatcttttagacgaatactgttctgcgcatcaggtgaatgaannnnnnnnnnnnnnnnnnnnnnnnnnnnnnnnnnnnnnnnnnnnnnnnnnnnnnnnnnnnnnnnnnnnnnnnnnnnnnnnnnnnnnnnagtcgtgcgcaactgtgccgcgcaCATCTTCTGCTCGTCCTTGGGTGTTGCGCGTTGAGTCGCTGCAGGTGAGTAAACACGCAGACGTTCCGCTCACAGATATTTCGTTTTCtgtggccgcatctgctATCATAGGGATAGTCGGTACGCCAGAAGATGGTGTGCACGTATTGGAAGATATACTGTGTGGTATGCACGCTGGGGCGTCGCGTACGCACTGCACCGGGAATATTCTTTTGCAGGAGCACGATCAGGTGTGGTGTCTCCCCCTCCAGCGCAATACGCCCTCGCTGCTGCGGGCACACGGGGTTGCGTGTGTGCCGTCTAATTGCATACAGCGCGGTGCCTCAATGCAGTTAACTCTTTTTGATTTGCTCGTTCCCTACACGTTGCGTACGTGGCGCACGCGTGTGCGCGCCCAGATGCGCTTTGTTGCTCGTCTTCTTGCAGAAGAAGAAATTTACTGCGATCCGTTGCAGCCTGCCTGTACGCTTTCAGGCGGACAGTTGCAGCGCGTTATTTTAGCGCGTGAGCTTGCAACGCGTCCACGTCTTCTCATTTTGGCAGAACCTGCAGAAGGATTGGATAGCGCCAGTGAACAGCGCTTGCTTGCACGTCTGCGCCAGGTAGCACAGGCAGGAACTGCTCTTGTGCTCCTTGCGCGAGAACAGCATCAGGCACAGTGGCGTGCGTTGTGCACAGAACGTTTCTTGCTACGTGCAGGTACGTTGTGTGCTGAAGTGTCAGGCACTCCGTCTCCGTCTCAGGATTCTCATACGTGAAACGGGTGATCAATTCGTGTATCGCAGTGTTGCTCGGTGTTGCAGTTATGAGTGCtgtgattgttctgtgctcggaaaatccatccgtatctttggcagcgttttttctnnnnnnnnnnnnnnnnnnnnnnnnnnnnnnnnnnnnnnnnnnnnnnnnnnnnnnnnnnnnnnnnnnnnnnnngggggcgagcnnnnnnnnnnnnnnnnnnnnnnnnnnnnnnnnnnnnnnnGGCCAGATCTATGCTGCAGGTTTTGTCACCGCATTGTTGCTCCGTGAGTACTGGGGTGTGGGCTTTTTGTTGCAATGGAGTGTTGCGCTCCTCTGTGCGCTGTCGGTCGCGGGGATATTAGCCTGTGTTTCTGGGATACTAAAAGCATGGCTGGCAACTAGCGAAATGATTACTTCCTTTTTGTTATCCACCGCAnnnnnnnnnnnnnnnnnnnnnnnnnnnnnnnnnnnnnnnnnnnnnnnnnnnnnnnnnnnnnnnnnnnnnnnnnnnnnnnnnnnnnnnnnnnnnnnnnnnnnnnnnnnntctttgttcggtGTGCCGGCCGTATTGACCTATGCGTCCTTAGTAGCACTCGCAGTTGGGTGTTTTTTTTCGTACACCCGCGTAGGATATCAGTTTCGCATATGCGGAAAGGCCCCCGAATTTGGGCGTTTTGTAGGGTTTCCCGTATGGGCCACCTATGTGTGGGGAATGGTGCTTTcaggggcgctgtttgggcttaccggatnnnnnnnnnnnnnnnnnnnnnnnnnnnnnnnnnnnnnnnnnnnnnnnnnnnnnnnnnnnnnnnnnnnnnnnnnnnnnnnnnnnnnnnnnnnnnnnnnnnnnnnnnnnnnnnnnnnnnnnnnnnnnnnnnnnnnnnnnnnnnnnnnnnnnnnnnnnnnnnnnnnnnnnnnnnnnnnnnnnnnnnnnnnnnnnnnnnnnnnnnnnnnnnnnnnnnnnnnnnnnnntatttctgcacaatggtccgcaccgtggnntgcagtgcggcgagGCGCACGCCGGGTATATCGATTCCTTGTCACCGTTTTTTGTTTTCGTGGGGAAAAGCACAGAACTCGCAGACGTCATGCGCTTTCTGTACATGATACACATCATAGGCGGTCTCGATGGGAGTGATAGGCACCACAGTGATAGCAATATTACATCGCGCAGCACCGCTTGCATGCGCTGCAGCAGGAGCGCTCGCAACAGAATACGCGGGAGTGTTGGGAATTTTTATGGAAGGGGTGATTACCTTCTCGTCCTTTTGCATAGCGTTTTTCGCACTGGTATGGGGAAGTTATTGGGGTGGACTGGGTATTACCGTGTGTGTGGTGCCGCTGTGTTTATTTTTTGTGGCCGTGGGTACGGagcgtatgcgggcaaatccGTTTTTAACGGGCATAGCAGTGCACTTTTCTGCAATGGGGATGAGCGCATTTGGTGCGTCCAGCATGTTTGCACGCGCTGCAGCTTCTGCGATGCAGATGGACACGGCAGCGCATGGTGTCTCTTTCACTCATGTTTCTCTTGCACATACCCGCGTTTTACCTCACCCCCTGTGGGGGACtgcggTGGCCTTTGCACTGGTGTGGGTTTTTCACCTGTATCTGTACTCTACAAACGTGGGTATCAATTTTATGCACTCTGGGGAAGGTGCGTTAGCCTTGCAGGTGCGGGGAACTGACGCGGCGCGGTATCGGATGGTGTCGTGGGCGGTAGCAGGCGTGTGTGCGGTGTGTGCGGGGGGACTGTTAGnattgcgtgtcggtacgtacaccccgcagatggctgcaggcagaggatggacggcacttgcaattgtttttcttgcacgcaagcggatgatGTGGTGTGTGCCGGCGGCGAtttttttctccggtattgaacacatgtgtgacgtattgcagnnnnnnnnnnnnnnnnnnnnnnnnnnnnnnnnnnnnnnnnnnnnnnnnnnnnnnnnnnnnnnnnnnnnnnnnnnnnnnnnnnnnnnnnnnnnnnnnnnnnnnnnnnnnnnnnnnnnnnnnnnnnnnnnnnnnnnnnnnnnnnnnnnnnnnnnnnnnnnnnnnnnnnnnnnnnnnnnnnnnnnnnnnnnnnnnnnnnnnnnnnnnnnnnnnnnnnnnnnnnnnnnnnnnnnnnnnnnnnnnnnnnnnnnnnnnnnnnnnnnnnnnnnnnnnnnnnnnnnnnnnnnnnnnnnnnnnnnnnnnnnnnnnnnnnnnnnnnnnnnnnnnnnnnnnnnnnnnnnnnnnnnnnnnnnnnnnnnnnnnnnnnnnnnnnnnnnnnnnnnnnnnnnnnnnnnnnnnnnnnnnnnnnnnnnnnnnnnnnnnnnnnnnnnnnnnnnnnnnnnnnnnnnnnnnnnnnnnnnnnnnnnnnnnnnnnnnnnnnnnnnnnnnnnnnnnnnnnnnnnnnnnnnnnnnnnnnnnnnnnnnnnnnnnnnnnnnnnnnnnnnnnnnnnnnnnnnnnnnnnnnnnnnnnnnnnnnnnnnnnnnnnnnnnnnnnnnnnnnnnnnnnnnnnnnnnnnnnnnnnnnnnnnnnnnnnnngcGTTCTACGGCGATGAATGTTCCTGCTACCTGTCCGAGTACtTTGAATGCACCTGTAtgggnatggttgtgcacaggcgcatctccttcggcagcgggggcTGGGTGAGTGCAATCGCGCGCGTGTTCGCACGAGATGTGAGGAGGTGACGGaggaagcaggtgcgcatgctgcacgggtagagggaacagggattcgtaccggggtgtgtgcgcgcagggagcggcgggttcttctcgtacgccggtgcagnnnnnnnnnnnnnnnnnnnnnnnnnnnnnnnnnnnnnnnnnnnnnnnnnnnnnnnnnnnnnnnnnnnnnnnnnnnnnnnnnnnnnnnnnnnnnnnnnnnnnnnnnnnnnnnnnnnnnnnnnnnnnnnnnnnnnnnnnnnnnnnnnnnnnnnnnnnnnnnnnnnnnnnnnnnnnnnnnnnnnnnnnnnnnnnnnnnnnnnnnnnnnnnnnnnnnnnnnnnnnnnnnnnnnnnnnnnnnnnnnnnnnnnnnnnnnnnnnnnnnnnnnnnnnnnnnnnnnnnnnnnnnnnnnnnnnnnnnnnnnnnnnnnnnnnnnnnnnnnnnnnnnnnnnnnnnnnnnnnnnnnnnnnnnnnnnnnnnnnnnnnnnnnnnnnnnnnnnnnnnnnnnnnnnnnCTTCTATGGTGTGGAAGAGCGCAGGATCTCCTTTGAGCATTTGCGTTTCAAGACACCGATCCTTCCGGGTTTGCTGACTGAGCAGGGTGAGCTTGTGCGTTCCGTCGACGGTGAAGCnnnnnnnnnnnnnnnnnnnnnnnnnnnnnnnnnnnnnnnnnnnnnnnnnnnnnnnnnnnnnnnnnnnnnnnnnnnnnnnnnnnnnnnnnnnnnnnnnnnnnnnnnnnnnnnnnnnnnnnnnnnnnnnnnnnnnnnnnnnnnnnnnnnnnnnnnnnnnnnnnnnnnnnnnnnnnnnnnnnnnnnnnnnnnnnnnnnnnnnnnnnnnnnnnnnnnnnnnnnnnnttggctGGTAGTGCCACGCGAGGGGCCCTGATCGGGTGCTCGTAAGGTGCAGGCGGCTGACTGCGGCGATGGAGGCGAGTGCTTCTCCCCGGAAGCCTAACGTGCGCAGCTGCAATAAGTCGTCTGCAGAcgatattttgctcgtggtgtgnnnnnnnnnnnnnnnnnnnnnnnnnnnnnnnnnnnnnnnnnnnnnnnnnnnnnnnnnnnnnnnnnnnnnnnnnnnnnnnnnnnnnnnnnnnnnnnnnnnnnnnnnnnnnnnnnnnnnnnnnnnnntctcgagcaattcgcgcacgacggaggcgggccgctcgatgacttctcctgcggcgatttttttagcggtgtcaggggacagccggtggataggtttgtaggacgtttcatgcatagaaaaannnnnnnnnnnnnnnnnnnnnnnnnnnnnnnnnnnnnnnnnnnnnnnnnnnnnnnnnnnnnnnnnnnnnnnnnnnnnnnnnnnnnnnnnnnnnnnnnnnnnnnnnnnnnnnnnnnnnnnnnnnnnnnnnnnnnnnnnnnnnnnnnnnnnnnnnnnnnnnnnnnnnnnnnnnnnnnnnnnnnnnnnnnnnnnnnnnnnnnnnnnnnnnnnnnnnnnnnnnnnnnnnnnnnnnnnnnnnnnnnnnnnnnnnnnnnnnnnnnnnnnnnnnnnnnnnnnnnnnnnnnnnnnnnnnnnnnnnnnnnnnnnnnnnnnnnnnnnnnnnnnnnnnnnnnnnnnnnnnnnnnnnnnnnnnnnnnnnnnnnnnnnnnnnnnnnnnnnnnnnnnnnnnnnnnnnnnnnnnnnnnnnnnnnnnnnnnnnnnnnnnnnnnnnnnnnnnnnnnnnnnnnnnnnnnnnnnnnnnnnnnnnnnnnnnnnnnnnnnnnnnnnnnnnnnnnnnnnnnnnnnnnnnnnnnnnnnnnnnnnnnnnnnnnnnnnnnnnnnnnnnnnnnnnnnnnnnnnnnnnnnnnngaatgaccctttcgtttgcacgagcaccgcgtcttttgnnnnnnnnnnnnnnnnnnnnnnnnnnnnnnnnnnnnnnnnnnnnnnnnnnnnnnnnnnnnnnnnnnnnnnnnnnnnnnnnnnnnnnnnnnnnnnnnnnnnnnnnnnnnnnnnnnnnnnnnnnnnnnnnnnnnnnnnnnnnnnnnnnnnnnnnnngtaataatgtggtccgtttgcgccctgtccattgcaggcgcagcactgctgccccccgttgttgccgcagcaattcCCGTTACCCCCGCCGTTGGCGCACAGGCAGGAGTnnnnnnnnnnnnccacggcagttcgcacaaaaaacgcgtttcggtcatcctgcataggaaagtttgtcagcgtttcaaaggccccgccgagcttagtgtaccggaaccctccggtgtnnnnnnnnnnnnnnnnnnnnnnnnnnnnnnnnnnnnnnnnnnnnnnnnnnnnnnnnnnnnnnnnnnnnnnnnnnnnnnnnnnnnnnnnnnnnnnnnnnnnnnnnnnnnnnnnnnnnnnnnnnnnnnnnnnnnnnnnnnnnnnnnnnnnnnnnnnnnnnnnnnnnnnnnnnnnnnnnnnnnnnnnnnnnnnnnnnnnnnnnnnnnnnnnnnnnnnnnnnnnnnnnnnnnnnnnnnnnnnnnnnnnnnnnnnnnnnnnnnnnnnnnnnnnnnnnnnnnnnnnnnnnnnnnnnnnnnnnnnnnnnnnnnnnnnnnnnnnnnnnnnnnnnnnnnnnnnnnnnnnnnnnnnnnnnnnnnnnnnnnnnnnnnnnnnncgctcgcgcagggcgcggtcatcccgctggtattggctcatgtgcatgcggtgtctgccggtgtaggccacacgcacaaccggctnnnnnnnnnnnnnnnnnnnnnnnnnnnnnnnnnnnnnnnnnnnnnnnnnnnnnnnnnnnnnnnnnnnnnnnnnnnnnnnnnnnnnnnnnnnnnnnnnnnnnnnnnnnnnnnnnnnnnnnnnnnnnnnnnnnnnnnnnnnnnnnnnnnnnnnnnnnnnnnnnnnnnnnnnnnnnnnnnnnnnnnnnnnnnnnnnnnnnnnnnnnnnnnnnnnnnnnnnnnnnnnnnnnnnnnnnnnnnnnnnnnnnnnnnnnnnnnnnnnnnnnnnnnnnnnnnnnnnnnnnnnnnnnnnnnnnnnnnnnnnnnnnnnnnnnnnnnnnnnnnnnnnnnnnnnnnnnnnnnnnnnnnnnnnnnnnnnnnnnnnnnnnnnnnnnnnnnnnnnnnnnnnnnnnnnnnnnnnnnnnnnnnnnnnggtagggtctgtgcggtccttgagcgtgaggcgcgagtattgCCCCTGCACccggacgaatactattttctgcccGTTCCCCACAAAGCAAGCTTCGGACGCGCCAGTGTACACCTCCGGCGAGAACGAGCCTGAGTGTGTGTCAAAAACGCGTACTCGGTGCGTGCGCTCGCTCCACGTGTCAATGGTGTCTGATACTGCCAAATAGCGTCCGTCCTCGGAGAAGTTCAGATGATGCAGCGTGTTGTCATGAGAGAacagcgtgcgcaccccacctgctttgtcacggaaacgcaccgcgtgannnnnnnnnnnnnnnnnnnnnnnnnnnnnnnnnnnnnnnnnnnnnnnnnnnnnnnnnnnnnnnnnnnnnnnnnnnnnnnnnnnnnnnnnnnnnnnnnnnnnnnnnnnnnnnnnnnnnnnnnnnnnnnnnnnnnnnnnnnnnnnnnnnnnnnnnnnnnnnnnnnnnnnnnnnnnnnnnnnnnnnnnnnnnnnnnnnnnnnnnnnnnnnnnnnnnnnnnnnnnnnnnnnnnnnnnnnnnnnnnnnnnnnnnnnnnnnnnnnnnnnnnnnnnnnnnnnnnnnnnnnnnnnnnnnnnnnnnnnnnnnnnnnnnnnnnnnnnnnnnnnnnnnnnnnnnnnnnnnnnnnnnnnnnnnnnnnnnnnnnnnnnnnnnnnnnnnnnnnnnnnnnnnnnnnnnnnnnnnnnnnnnnnnnnnnnnnnnnnnnnnnnnnnnnnnnnnnnnnnnnnnnnnnnnnnnnnnnnnnnnnnnnnnnnnnnnnnnnnnnnnnnnnnnnnnnnnnnnnnnnnnnnnnnnnnnnnnnnnnnnnnnnnnnnnnnnnnnnnnnnnnnnnnnnnnnnnnnnnnnnnnnnnnnnnnnnnnnnnnnnnnnnnnnnnnnnnnnnnnnnnnngcctgcatcctgtgtgtgggcatacaggacgacnnnnnnnnnnnnnnnnnnnnnnnnnnnnnnnnnnnnnnnnnnnnnnnnnnnnnnnnnnnnnnnnnnnnnnnnnnnnnnnnnnnnnnnnnnnnnnnnnnnnnnnnnnnnnnnnnnnnnnnnnnnnnnnnnnnnnnnnnnnnnnnnnnnnnnnnCTTCTGCGCACAACCTCTGGAAAGTGAAGACCATGGCAAGAGCGAGGGCAGCGCGCGCGTTCATGGGGCCCATGGTnnnnnnnnnnnnnnnnnnnnnnnnnnnnnnnnnnnnnnnnnnnnnnnnnnnnnnnnnnngaggcagggtgctcccttttaaaaaacgcatggacacgctacactcgtgccgtatggatccggcttttatctttattacaggcggtgtagtttcctcgctgggaaagggtattgccgcaggtgccatcggacttttgcttnnnnnnnnnnnnnnnnnnnnnnnnnnnnnnnnnnnnnnnnnnnnnnnnnnnnnnnnnnnnnnnnnnnnnnnnnnnnnnnnnnnnnnnnnnnnnnnnnnnnnnnnAGATGGCGGCGAAACGGACCTGGATCTGGGCCATTACGAGCGCTTCACGGACGTGCCTTCAAGTAGATTCAATAGCACGACTGCGGGGAGCGTGTACCGGGCTATTTTGGACCGTGAACGCGCGGGAGGATACGGAGGTGCTACCGTACaggttnnnnnnnnnnnnnnnnnnnnnnnnnnnnnnnnnnnnnnnnnnnnnnnnnnnnnnnnnnnnnnnnnnnnnnnnnnnnnnnnnnnnnnnnnnnnnnnnnnnnnnnnnnnnnnnnnnnnnnnnnnnnnnnnnnnnnnnnnnnnnnnnnnnnnnnnnnnnnnnnnnnnnnnnnnnnnnnnnnnnnnnnnnnnnnnnnnnnnnnnnnnnnnnnnnnnnnnnnnnnnnnnnnnnnnnnnnnnnnnnnnnnnnnnnnnnnnnnnnnnnnnnnnnnnnnnnnnnnnnnnnnnnnnnnnnnnnnnnnnnnnnnnnnnnnnnnnnnnnnnnnnnnnnnnnnnnnnnnnnnnnnnnnatgttgagcggcgtgctattgtcgagaacgtgacggctcgttctatctatgaggtgccgctcnnnnnnnnnnnnnnnnnnnnnnnnnnnnnnnnnnnnnnnnnnnnnnnnnnnnnnnnnnnnnnnnnnnnnnnnnnnnnnnnnnnnnnnnnnnnnnnnnnnnnnnnnnnnnnnnnnnnnnnnnnnnnnnnnnnnnnnnnnnnnnnnnnnnnnnnnnnnnnnnnnnnnnnnnnnnnnnnnnnnnnnnnnnnnnnnnnnnnnnnnnnnnnnnnnnnnnnnnnnnnnnnnnnnnnnnnnnnnnnnnnnnnnnnnnnnnnnnnnnnnnnnnnnnnnnnnnnnnnnnnnnnnnnnnnnnnnnnnnnnnnnnnnnnnnnnnnnnnnnnnnnnnnnnnnnnnnnnnnnnnnnnnnnnnnnnnnnnnnnnnnnnnnnnnnnnnnnnnnnnnnnnnnnnnnnnnnnnnnnnnnnnnnnnnnnnnnnnnnnnnnnnnnnnnnnnnnnnnnnnnnnnnnnnnnnnnnnnnnnnnnnnnnnnnnnnnnnnnnnnnnnnnnnnnnnnnnnnnnnnnnnnnnnnnnnnnnnnnnnnnnnnnnnnnnnnnnnnnnnnnnnnnnnnnnnnnnnnnnnnnnnnnnnnnnnnnnnnnnnnnnnnnnnnnnnnnnnnnnnnnnnnnnnnnnnnnnnnnnnnnnnnnnnnnnnnnnnnnnnnnnnnnnnnnnnnnnnnnnnnnnnnnnnnnnnnnnnnnnnnnnnnnnnnnnnnnnnnnnnnnnnnnnnnnnnnnnnnnnnnnnnnnnnnnnnnnnnnnnnnnnnnnnnnnnnnnnnnnnnnnnnnnnnnnnnnnnnnnnnnnnnnnnnnnnnnnnnnnnnnnnnnnnnnnnnnnnnnnnnnnnnnnnnnnnnnnnnnnnnnnnnnnnnnnnnnnnnnnnnnnnnnnnnnnnnnnnnnnnnnnnnnnnnnnnnnnnnnnnnnnnnnnnnnnnnnnnnnnnnnnnnnnnnnnnnnnnnnnnnnnnnnnnnnnnnnnnnnnnnnnnnnnnnnnnnnnnnnnnnnnnnnnnnnnggtattttttctagactcctctgtccttctcagccggttgaaagagctttgtgtgggcgtgcgggttttgtttgcgcgcgctccgatgnnnnnnnnnnnnnnnnnnnnnnnnnnnnnnnnnnnnnnnnnnnnnnnnnnnnnnnnnnnnnnnnnnnnnnnnnnnnnnnnnnnnnnnnnnnnnnnnnnnnnnnnnnnnnnnnnnnnnnnnnnnnnnnnnnnnnnnnnnnnnnnnnnnnnnnnnnnnnnnnnnnnnnnnnnnnnnnnnnnnnnnnnnnnnnnnnnnnnnnnnnnnnnnnnnnnnnnnnnnnnnnnnnnnnnnnnnnnnnnnnnnnnnnnnnnnnnnnnnnnnnnnnnnnnnnnnnnnnnnnnnnnnnnnnnnnnnnnnnnnnnnnnnnnnnnnnnnnnnnnnnnnnnnnnnnnnnnnnnnnnnnnnnnnnnnnnnnnnnnnnnnnnnnnnnnnnnnnnnnnnnnnnnnnnnnnnnnnnnnnnnnnnnnnnnnnnnnnnnnnnnnnnnnnnnnnnnnnnnnnaatgggaagacggccaatatcccttccatgcgcattagcgcgcatgatgtcatcactactaagaaccggaaaggtattcatnnnnnnnnnnnnnnnnnnnnnnnnnnnnnnnnnnnnnnnnnnnnnnnnnnnnnnnnnnnnnnnnnnnnnnnnnnnnnnnnnnnnnnnnnnnnnnnnnnnnnnnnnnnnnnnnnnnnnnnnnnnnnnnnnnnnnnnnnnnnnnnnnnnnnnnnnnnnnnnnnnnnnnnnnnnnnnnnnnnnnnnnnnnnnnnnnnnnnnnnnnnnnnnnnnnnnnnnnnnnnnnnnnnnnnnnnnnnnnnnnnnnnnnnnnnnnnnnnnnnnnnnnnnnnnnnnnnnnnnnnnnnnnnnnnnnnnnnnnnnnnnnnnnnnnnnnnnnnnnnnnnnnnnnnnnnnnnnnnnnnnnnnnnnnnnnnnnnnnnnnnnnnnnnnnnnnnnnnnnnnnnnnnnnnnnnnnnnnnnnnnnnnnnnnnnnnnnnnnnnnnnnnnnnnnnnnnnnnnnnnnnnnnnngttttctacccgtgagaagcctggtgttgttcttgaacagaacccacctgcgggggccatcgtcaaggctGGGCGCTACgtggacctcgtagtgagccaacaagcagtgactacgcacgttgaggactatcggggattgcaggttgaagaannnnnnnnnnnnnnnnnnnnnnnnnnnnnnnnnnnnnnnnnnnnnnnnnnnnnnnnnnnnnnnnnnnnnnnnnnnnnnnnnnnnnnnnnnnnnnnnnnnnnnnnnnnnnnnnnnnnnnnnnnnnnnnnnnnnnnnnnnnnnnnnnnnnnnnnnnnnnnnnnnnnnnnnnnnnnnnnnnnnnnnnnnnnnnnnnnnnnnnnnnnnnnnnnnnnnnnnnnnnnnnnnnnnnnnnnnnnnnnnnnnnnnnnnnnnnnnnnnnnnnnnnnnnnnnnnnnnnnnnnnncttctggggagagaaaagacggagaagcacgTGGAAGAACGCGTGCCAATGCGCAGGACTACGCGCGGGTnnnnnnnnnnnnnnnnnnnnnnnnnnnnnnnnnnnnnnnnnnnnnnnnnnnnnnnnnnCAGGTGCTCTTTCCAGAGCGTGGAGAGGCTCACGAAATATACGGTATCTTAGnnnnnnnnnnnnnnnnnnnnnnnnnnnnnnnnnnnnnnnnnnnnnnnnnnnnnnnnnnnnnnnnnnnnnnnnnnnnnnnnnnnnnnnnnnnnnnnnnnnnnnnnnnnnnnnnnnnnnnnnnnnnnnnnnnnnnnnnnnnnnnnnnnnnnnnnnnnnnnnnnnnnnnnnnnnnnnnnnnnnnnnnnnnnnnnnnnnnnnnnnnnnnnnnnnnnnnnnnnnnnnnnnnnnnnnnnnnnnnnnnnnnnnnnnnnnnnnnnnnnnnnnnnnnnnnnnnnnnnnnnnnnnnnnnnnnnnnnnnnnnnnnnnnnnnnnnnnnnnnnnnnnnnnnnnnnnnnnnnnnnnnnnnnnnnnnnnnnnnnnnnnnnnnnnnnnnnnnnnnnnnnnnnnnnnnnnnnnnnnnnnnnnnnnnnnnnnnnnnnnnnnnnnnnnnnnnnnnnnnnnnnnnnnnnnnnnnnnnnnnnnnnnnnnnnnnnnnnnnnnnnnnnnnnnnnnnnnnnnnnnnnnnnnnnnnnnnnnnnnnnnnnnnnnnnnnnnnnnnnnnnnnnnnnnnnnnnnnnnnnnnnnnnnnnnnnnnnnnnnnnnnnnnnnnnnnnnnnnnnnnnnnnnnnnnnnnnnnnnnnnnngccatatctgcgtactgcgcaggtacnnnnnnnnnnnnnnnnnnnnnnnnnnnnnnnnnnnnnnnnnnnnnnnnnnnnnnnnnnnnnnnnnnnnnnnnnngtttcggctgtgcatacgcgcgGTGATATTTTGCCTATGCGCTCGTCGCATAGACAGGnnnnnnnnnnnnnnnnnnnnnnnnnnnnngtaccgggaaatgagaagtGGCAGTTTGGATTTGAACCACACGGGAAGGTTGTGTGGTACCGACACCGGAGTGCCATGCTTGAGGCGCTGCGCACCCGGGCGGTGGACGTGGCACTTGTGGATCTGGTTGAGGCTCATGACGCAGTGCATCGTCAGGGTGCGCCTCTGAGGGTGATGCGGGTACCGCTTGGGTTGAGCCAGTATGCGGTTGCATTTCGGCGTGAGGATCGTGCGTTGCGTGACGAAATTCAGCGAATCTTGTATCGTATTGCTGCCTCCGGTGAGGCATACCGTATTGCAGAAAAATGGTTTGGTGTTGGTCAGTCGGTTATTGGGATagaataaaggtgcaaggcnnnnnnnnnnnnnnnnnnnnnnnnnnnnnnnnnnnnnnnnnnnnnnnnnnnnnnnnnnnnnnnnnnnnnnnnnnnnnnnnnnnnnnnnnnnnnnnnnnnnnnnnnnnnnnnnnnnnnnnnnnnnnnnnnnnnnnnnnnnnnnnnnnnnnnnnnnnnnnnnnnnnnnnnnnnnnnnnnnnnnnnnnnnnnnnnnnnnnnnnnnnnnnnnnnnnnnnnnnnnnnnnnnnnnnnnnnnnnnnnnnnnnnnnnnnnnnnnnnnnnnnnnnnnnnnnnnnnnnnnnnnnnnnnnnnnnnnnnnnnnnnnnnnnnnnnnnnnnnnnnnnnnnnnnnnnnnnnnnnnnnnnnnnnnnnnnnnnnnnnnnnnnnnnnnnnnnnnnnnnnnnnnnnnnnnnnnnnnnnnnnnnnnnncacgcgcgcagatttgcactgacgcAGCCGTACGTACGCGATGTGCGCGTCTTTGCGGTGTTGCGCCAAGCCCCGTACGCAACGGTTGCAGACCTGCATggaaagcggctcggggtccacgcagtgaccgatgtggaagaaaatgatgcatnnnnnnnnnnnnnnnnnnnnnnnnnnnnnnnnnnnnnnnnnnnnnnnnnnnnnnnnnnnnnnnnnnnnnnnnnnnnnnnnnnnnnnnnnnnnnnnnTGGTGACGCTCTGCGCAGTGACGCCGCACCTGCGGCGCTTGTATCGAATTTTGGATGAACCGATAGACACGTGTGAATACGTGTTTGCGTTTCGTGCGGAtgcgcgtnnnnnnnnnnnnnnnnnnnnnnnnnnnnnnnnnnnnnnnnnGCGAGAGGGTTTTGTgtcagcgctctcaaagcggtggtttggcagcgatatgtccatcatcgaccgctaaggcgggtggagggggaatacnnnnnnnnnnnnnnnnnnnnnnnnnnnnnnnnnnnnnnnnnnnnnnnnnnnnnnnnnnnnnnnnnnnnnnnnnnnnnnnnnnnnnnnnnnnnnnnnnnnnnnnnnnnnnnnnnnnnnnnnnnnnnnnnnnnnnnnnnnnnnnnnnnnnnnnnnnnnnnnnnnnnnnnnnnnnnnnnnnnnnnnnnnnnnnnnnnnnnnnnnnnnnnnnnnnnnnnnnnnnnnnnnnnnnnnnnnnnnnnnnnnnnnnnnnnnnnnnnnnnnnnnnnnnnnnnnnnnnnnnggtaaagattgncgcngagcatgtagagtttcagactccttttgtatggtgagggtgtgtnGCGCATGCGATGGACGCGGTTGAACTGAGAGAAGTGCCCGGATGAAGGGGAGGGTGCGTTTTTTCGCAGAAAGAAGACCAGCAGGGGAGGCGGAGCATGAGTTCACCGGTCATGCACGTGTGGATnnnnnnnnnnnnnnnnnnnnnnnnngtggtacCGGGTCTTTCGGGGGGTACGGTTGCGCTGCtctgtggcacgtgggaaTTACTGATTGCAGCAATAGCATTGGATAGAGCGCATCTGTGCCGTCAGTGGCGACGCTTGCTTGCCCTTGCGGGGGGAATAGTGGTGGGGATAGGGGTGTGCGCacgctttatgcgtgcgttgtacgaagcatttcctcatcttacgaatgnnnnnnnnnnnnnnnnnnnnnnnnnnnnnnnnnnnnnnnnnnnnnnnnnnnnnnnnnnnnnnnnnnnnnnnnnnnnnnnnnnnnnnnnnnnnnnnnnnnnnnnnnnnnnnnnnnnnnnnnnnnnnnnnnnnnnnnnnnnnnnnnnnnnnnnnnnnnnnnnnnnnnnnnnnnnnnnnnnnnnnnnnnnnnnnnnnnnnnnnnnnnnnnnnnnnnnnnnnnnnnnnnnnnnnnnnnnnnnnnnnnnnnnnnnnnngactacggcggtccttactactgcacacacgcgcggatttgtggcgaccgtgtgcgcaggggcgctannnnnnnnnnnnnnnnnnnnnnnnnnnnnnnnnnnnnnnnnnnnnnnnnnnnnnnnnnnnnnnnnnnnnnnnnnnnnnnnnnnnnnnnnnnnnnnnnnnnnnnnnnnnnnnnnnnnnnnnnnnnnnnnnnnnnnnnnnnnnnnnnnnnnnnnnnnnnnnnnnnnnnnnnnnnnnnnnnnnnnnnnnnnnnnnnnnnnnnnnnnnnnnnnnnnnnnnnnnnnnnnnnnnnnnnnnnnnnnnnnnnnnnnnnnnnnnnnnnnnnnnnnnnnnnnnnnnnnncncgtgctgggatcgagcgtgacgttgttcccacggggaactgcagccgggatgcaggatcagtttacgctttttatgggatgtgtggctgggtgtgtcggtgcgtttgcgctcgcaaaggtctgcgcctggggtgcgcgnnnnnnnnnnnnnnnnnnnnnnnnnnnnnnnnnnnnnnnnnnnnnnnnnnnnnnnnnnnnnnnnnnnnnnnnnnnnnnnnnnnnnnnnnnnnnnnnnnnnnnnnnnnnnnnnnnnnnnnnnnnnnnnnnnnnnnnnnnnnnnnnnnnnnnnnnnnnnnnnnnnnnnnnnnnnnnnnnnnnnnnnnnnnnnnnnnnnnnnnnnnnnnnnnnnnnnnnnnnnnnnnnnnnnnnnnnnnnnnnnnnnnnnnnnnnnnnnnnnnnnnnnnnnnnnnnnnnnnnnnnnnnnnnnnnnnnnnnnnnnnnnnnnnnnnnnnnnnnnnnnnnnnnnnnnnnnnnnnnnnnnnnnnnnnnnnnnnnnnnnnnnnnnnnnnnnnnnnnnnnnnnnnnnnnnnnnnnnnnnnnnnnnnnnnnnnnnnnnnnnnnnnnnnnnnnnnnnnnnnnnnnnnnnnnnnnnnnnnnnnnnnnnnnnnnnnnnnnnnnnnnnnnnnnnnnnnnnnnnnnnnnnnnnnnnnnnnnnnnnnnnnnnnnnnnnnnnnnnnnnnnnnnnnnnnnnnnnnnnnnnnnnnnnnnnnnnnnnnnnnnnnnnnnnnnnnnnnnnnnnnnnnnnnnnnnnnnnnnnnnnnnnnnnnnnnnnnnnnnnnnnnnnnnnnnnnnnnnnnnnnnnnnnnnnnnnnnnnnnnnnnnnnnnnnnnnnnnnnnnnnnnnnnnnnnnnnnnnnnnnnnnnnnnnnnnnnnnnnnnnnnnnnnnnnnnnnnnnnnnnnnnnnnnnnnnnnnnnnnnnnnnnnnnnnnnnnnnnnnnnnnnnnnnnnnnnngccagcaatgttacgctagaagacggaTACCTCATCGGTGCACAGAAGGACGCAAACAATCAGAACAAGGATAAACTGCTGTGGAATGTAGGGGGCCGACTCACCCTCGAACCAGGCGCCGGCTTCCGCTTCTCCTTCGCCCTCGACGCCggtaaccaacaccagagtgaggctaccgnnnnnnnnnnnnnnnnnnnnnnnnnnnnnnnnnnnnnnnnnnnnnnnnnnnnnnnnnnnnnnnncgctgcgcaggaacaggctaaacaggcggctgatacggttggtagcaccatagataactcggtgcaggtggcaagatcagttattactcnnnnnnnnnnnnnnnnnnnnnnnnnnnnnnnnnnnnnnnnnnnnnnnnnnnnnnnnnnnnnnnnnnnnnnnnnnnnnnnnnnnnnnnnnnnnnnnnnnnnnnnnnnnnnnnnnnnnnnnnnnnnnnnnnnnnnnnnnnnnnnnnnnnnnnnnnnnnnnnnnnnnnnnnnnnnnnnnnnnnnnnnnnnnnnnnnnnnnnnnnnnnnnnnnnnnnnnnnnnnnnnnnnnnnnnnnnnnnnnnnnnnngttatcacccagcaggtacaggcccgggttgcgcagtcgaccgcggttgcaatccagcaagtttctgtgttcaaccagcaaacnnnnnnnnnnnnnnnnnnnnnnnnnnnnnnnnnnnnnnnnnnnnnnnnnnnnnnnnnnnnnnnnnnnnnnnnnnnnnnnnnnnnnnnnnnnnnnnnnnnnnnnnnnnnnnnnnnnnnnnnnnnnnnnnnnnnnnnnnnnnnnnnnnnnnnnnnnnnnnnnnnnnnnnnnnnnnnnnnnnnnnnnnnnnnnnnnnnnnnnnnnnnnnnnnnnnnnnnnnnnnnnnnnnnnnnnnnnnnnnnnnnnnnnnnnnnnnnnnnnnnnnnnnnnnnnnnnnnnnnnnnnnnnnnnnnnnnnnnnnnnnnnnnnnnnnnnnnnnnnnnnnnnnnnnnnnnnnnnnnnnnnnnnnnnnnnnnnnnnnnnnnnnnnnnnnnnnnnnnnnnnnnnnnnnnnnnnnnnnnnnnnnnnnnnnnnnnnnnnnnnnnnnnnnnnnnnnnnnnnnnnnnnnnnnnnnnnnnnnnnnnnnnnnnnnnnnnnnnnnnnnnnnnnnnnnnnnnnnnnnnnnnnnnnnnnnnnnnnnnnnnnnnnnnnnnnnnnnnATACAAAAATAACGGGGGGACGGCTGTGCGTGGCATTCAGGAAAAGGAGTATATCCGTCnnnnnnnnnnnnnnnnnnnnnnnnnnnnnnnnnnnnnnnnnnnnnnnnnnnnnnnnnnnnnnnnnnnnnnnnnnnnnnnnnnnnnnnnnnnnnnnnnnnnnnnnnnnnnnnnnnnnnnnnnnnnnnnnnnnnnnnnnnnnnnnnnnnnnnnnnnnnnnnnnnnnnnnnnnnnnnnnnnnnnnnnnnnnnnnnnnnnnnnnnnnnnnnnnnnnnnnnnnnnnnnnnnnnnnnnnnnnnnnnnnnnnnnnnnnnnnnnnnnnnnnnnnnnnnnnnnnnnnnnnnnnnnnnnnnnnnnnnnnnnnnnnnnnnnnnnnnnnnnnnnnnnnnnnnnnnnnnnnnnnnnnnnnnnnnnnnnnnnnnnnnnnnnnnnnnnnnnnnnnnnnnnnnnnnnnnnnnnnnnnnnnnnnnnnnnnnnnnnnnnnnnnnnnnnnnnnnnnnnnnnnnnnnnnnnnnnnnnnnnnnnnnnnnnnnnnnnnnnnnnnnnnnnnnnnnnnnnnnnnnnnnnnnnnnnnnnnnnnnnnnnnnnnnnnnnnnnnnnnnnnnnnnnnnnnnnnnnnnnnnnnnnnnnnnnnnnnnnnnnnnnnnnnnnnnnnnnnnnnnnnnnnnnnnnnnnnnnnnnnnnnnnnnnnnnnnnnnnnnnnnnnnnnnnnnnnnnnnnnnnnnnnnnnnnnnnnnnnnnnnnnnnnnnnnnnnnnnnnnnnnnnnnnnnnnnnnnnnnnnnnnnnnnnnnnnnnnnnnnnnnnnnnnnnnnnnnnnnnnnnnnnnnnnnnnnnnnnnnnnnnnnnnnnnnnnnnnnnnnnnnnnnnnnnnnnnnnnnnnnnnnnnnnnnnnnnnnnnnnnnnnnnnnnnnnnnnnnnnnnnnnnnnnnnnnnnnnnnnnnnnnnnnnnnnnnnnnnnnnnnnnnnnnnnnnnnnnnnnnnnnnnnnnnnnnnnnnnnnnnnnnnnnnnnnnnnnnnnnnnnnnnnnnnnnnnnnnnnnnnnnnnnnnnnnnnnnnnnnnnnnnnnnnnnnnnnnnnnnnnnnnnnnnnnnnnnnnnnnnnnnnnnnnnnnnnnnnnnnnnnnnnnnnnnnnnnnnnnnnnnnnnnnnnnnnnnnnnnnnnnnnnnnnnnnnnnnnnnnnnnnnnnnnnnnnnnnnnnnnnnnnnnnnnnnnnnnnnnnnnnnnnnnnnnnnnnnnnnnnnnnnnnnnnnnnnnnnnnnnnnnnnnnnnnnnnnnnnnnnnnnnnnnnnnnnnnnnnnnnnnnnnnnnnnnnnnnnnnnnnnnnnnnnnnnnnnnnnnnnnnnnnnnnnnnnnnnnnnnnnnnnnnnnnnnnnnnnnnnnnnnnnnnnnnnnnnnnnnnnnnnnnnnnnnnnnnnnnnnnnnnnnnnnnnnnnnnnnnnnnnnnnnnnnnnnnnnnnnnnnnnnnnnnnnnnnnnnnnnnnnnnnnnnnnnnnnnnnnnnnnnnnnnnnnnnnnnnnnnnnnnnnnnnnnnnnnnnnnnnnnnnnnnnnnnnnnnnnnnnnnnnnnnnnnnnnnnnnnnnnnnnnnnnnnnnnnnnnnnnnnnnnnnnnnnnnnnnnnnnnnnnnnnnnnnnnnnnnnnnnnnnnnnnnnnnnnnnnnnnnnnnnnnnnnnnnnnnnnnnnnnnnnnnnnnnnnnnnnnnnnnnnnnnnnnnnnnnnnnnnnnnnnnnnnnnnnnnnnnnnnnnnnnnnnnnnnnnnnnnnnnnnnnnnnnnnnnnnnnnnnnnnnnnnnnnnnnnnnnnnnnnnnnnnnnnnnnnnnnnnnnnnnnnnnnnnnnnnnnnnnnnnnnnnnnnnnnnnnnnnnnnnnnnnnnnnnnnnnnnnnnnnnnnnnnnnnnnnnnnnnnnnnnnnnnnnnnnnnnnnnnnnnnnnnnnnnnnnnnnnnnnnnnnnnnnnnnnnnnnnnnnnnnnnnnnnnnnnnnnnnnnnnnnnnnnnnnnnnnnnnnnnnnnnnnnnnnnnnnnnnnnnnnnnnnnnnnnnnnnnnnnnnnnnnnnnnnnnnnnnnnnnnnnnnnnnnnnnnnnnnnnnnnnnnnnnnnnnnnnnnnnnnnnnnnnnnnnnnnnnnnnnnnnnnnnnnnnnnnnnnnnnnnnnnnnnnnnnnnnnnnnnnnnnnnnnnnnnnnnnnnnnnnnnnnnnnnnnnnnnnnnnnnnnnnnnnnnnnnnnnnnnnnnnnnnnnnnnnnnnnnnnnnnnnnnnnnnnnnnnnnnnnnnnnnnnnnnnnnnnnnnnnnnnnnnnnnnnnnnnnnnnnnnnnnnnnnnnnnnnnnnnnnnnnnnnnnnnnnnnnnnnnnnnnnnnnnnnnnnnnnnnnnnnnnnnnnnnnnnnnnnnnnnnnnnnnnnnnnnnnnnnnnnnnnnnnnnnnnnnnnnnnnnnnnnnnnnnnnnnnnnnnnnnnnnnnnnnnnnnnnnnnnnnnnnnnnnnnnnnnnnnnnnnnnnnnnnnnnnnnnnnnnnnnnnnnnnnnnnnnnnnnnnnnnnnnnnnnnnnnnnnnnnnnnnnnnnnnnnnnnnnnnnnnnnnnnnnnnnnnnnnnnnnnnnnnnnnnnnnnnnnnnnnnnnnnnnnnnnnnnnnnnnnnnnnnnnnnnnnnnnnnnnnnnnnnnnnnnnnnnnnnnnnnnnnnnnnnnnnnnnnnnnnnnnnnnnnnnnnnnnnnnnnnnnnnnnnnnnnnnnnnnnnnnnnnnnnnnnnnnnnnnnnnnnnnnnnnnnnnnnnnnnnnnnnnnnnnnnnnnnnnnnnnnnnnnnnnnnnnnnnnnnnnnnnnnnnnnnnnnnnnnnnnnnnnnnnnnnnnnnnnnnnnnnnnnnnnnnnnnnnnnnnnnnnnnnnnnnnnnnnnnnnnnnnnnnnnnnnnnnnnnnnnnnnnnnnnnnnnnnnnnnnnnnnnnnnnnnnnnnnnnnnnnnnnnnnnnnnnnnnnnnnnnnnnnnnnnnnnnnnnnnnnnnnnnnnnnnnnnnnnnnnnnnnnnnnnnnnnnnnnnnnnnnnnnnnnnnnnnnnnnnnnnnnnnnnnnnnnnnnnnnnnnnnnnnnnnnnnnnnnnnnnnnnnnnnnnnnnnnnnnnnnnnnnnnnnnnnnnnnnnnngccctgtctagtagccccctcaccctgcctcctgcacacacgcaaaaactcaccactccttgcacctccttgtccccttgggttacactgtgnccccttatttnnnnnnnnnnnnnnnnnnnnnnnnngaggattcttgtgagggagaagtgggtacgcgcgtttgcgggcgttttttgcgccatgctgctcatcggctgctctaagagcgacaggccgcagatgggaaacgcnnnnnnnnnnnnnnnnnnngacttcgtcgttGGAATGGTAACCGATTCAGGGGACATCGATGACAAGTCCTTTAACCAGCAGGTGTGGGAaggtatttcgcgcttcgcacaggagaacaacgcgaagtgcaagtatgtgactgctcgcactgacgctgagtacgtgnnnnnnnnnnnngcgtttgcagatgagaatatggagctcgtggtagcatgcggcnnnnnnnnnnnnnnnnnnnnnnnnnnnnnnnnnnnnnnnnnnnnnnnnnnnnnnnnnnnnnnnnnnnnnnnnnnnnnnnnngaccgggataacgttgtttctgcagtgtttggncagaatgaggggtcgttccttgtcggcgttgcagcggcgctgaaggcgaaagaggcgggaaaaagcgccgtcggtttcatcgttggcatgnnnnnnnnnnnnnnnnnnnnnnnnnnnnnnnnnnnnnnnnnnnnnnnnnnnnnnnnnnnnnnnnnnnnnnnnnnnnnnnnnnnnnnnnnnnnnnnnnnnnnnnnnnnnnnnnnnnnnnnnnnnnnnnnnnnnnnnnnnnnnnactcgggcgtgaatgtcatttttcaagtagcggggggcacaggaaacggcgttatcaaagaggcgcgcgatcgtcgtcnnnnnnnnnnnnnnnnnnnnnnnnttggcgtagatcgtgaccagtacatggatgGGGTGTACGATGGGTCGAAGTCTGTGGTGCTTACCTCCATGGTCAAGCGTGCGGATGTCGCTGCGGAGCGGATCTCAAAGATGGCGTACGATGGCTCTTTTCCCGGGGGGCAGTCCATTATGTTCGGGCTTGAAGACAAGGCAGTGGGGATTCCTGAGGAAAATCCCAATTTGagcagtgcggttatggagaaaattcggagttttgaggagaagattgtctcgaaggagatannnnnnnnnnnnnnnnnnnnnnnnnnnnnnnnnnnnnnnnnnnnnnnnnnnnnnnnnnnnnnnnnnnnnnnnnnnnnnnnnnnnnnnnnnnnnnnnnnnnnnnnnnnggccgtcgtgaatggcttctgtGTGCAGGACATTCCCTACGGGTCACGGGTTGTTTTGCCGGGGCGTATGCGTTCTTCTTCTGCGGGTGCGTAGAGTGGGGCGTGTGTCTCGACCCGCCCGTGGTCAGTGGGTATGGGGACGTCCAGTAAtgaacttgagggaggggctatgcnATACGCGGTGGAAATGCGCGATGTAACTGTCCGGTTCCCAGGCGTTGTTGCCAATGACTGTGTTTCTTTCGGTGTGCAGACCGCGGAGGTGCATGCCTTGCTGGGAGAGAATGGTGCAGGCAAGTCTACGCTCATGGGAGTCCTTTTTGGTACGTGTCCGAAGCAATCTGGAGAGCTGTTTGTAGATGGCAGGAGTGTGTGCATCCGtagtccgcgcgatgcggcgcgccatggcattggcatggtgcaccagcactttaatctggttcacaatctaaccGttagtgagaatatcgttcttggcgtcgagcctcgtgcgcgcnnnnnnnnnnnnnnnnnnnnnnnnnnnnnnnnnnnnnnnnnnnnnnnnnnnnnnnnnnnnnnnnnnnnnnnnnnnnnnnnnnnnnnnnnnnnnnnnnnnnCTGTTGGCAtgcagcagcgtgttgagattctcaaaatgctttaccgcgatgctcgggtgctcatttttgatgaacccaccgcagttctcgctccacaagaagtgcaGCAGCTGATGCAGGTGATCAGACGTCTTGCTCGTGAGGGTaaggcggtggtgcttatcaCACACAAACTGAGTGAAATTAAGGCAATCGCCGATCGCTGTACGGTACTGCGCAGGGGGGCGTGTATCGGTACGGTTTCTGTGGCTGAGGTGGGAGAAGAACGGTTGGTAGAAATGATGGTGGGCCATGCGGTGGACTACGCGCTGCCTCGCGCTTCAAGGAAGGATGGGGCGTGTGTAttagaggnnnnnnnnnnnnnnnnnnnnnnnnnnnnnnnnnnnnnnnnnnnnnnnnnnnnnnnnnnnnnnnnnnnnnnnnnnnnnnnnnnnnnnnnnnnnnnnnnnnnnnnnnnnnnnnnnnnnnnnnnnnnnnnnnnnnnnnnnnnnnnnnnnnnnnnnnnnnnnnnnnnnnnnnnnnnnnnnnnnnnnnnnnnnnnnnnnnnnnnnnnnnnnnnnnnnnnnnnnnnnnnnnnnnnnnnnnnnnnnnnnnnnnnnnnnnnnnnnnnnnnnnnnnnnnnnnnnnnnnnnnnnnnnnnnnnnnnnnnnnnnnnnnnnnnnnnnnnnnnnnnnnnnnnnnnnnnnnnnnnnnnnnnnnnnnnnnnnnnnnnnnnnnnnnnnnnnnnnnnnnnnnnnnnnnnnnnnnnnnnnnnnnnnnnnnnnnnnnnnnnnnnnnnnnnnnnnnnnnnnnnnnnnnnnnnnnnnnnnnnnngtgcggttcgtgCGCGTACCCTTTCAGGAGGTAATCAGCAAAAGGTTATCATTGCGCGCGAGTTGCACCGTGCACCGCGTCTTTTGATTGCCGCGCAGCCGACGCGCGGACTTGATTTGGGTGCGGTTCAGTATGTTCATCGCGCTATTGTCGCCGAACGTAATCGGGGGGGTGCAGTGCTCCTCTTTTCCCTTGATATGGATGAAGTGCTTGCACTGGCAGATTCTATTGCAGTTATGTACGAGGGAGAGATAGTGGGGACCGTGCACGCGTGCGACGCAACAGAGCAAGAGCTCGGGCGTCTCATGAGTGGGATGCGGAAAAAAGAGACTGCGGGCAAAAAAACCGGGGTACAGGGGTGATTGCGCGTCTCCGGGGGTGCCTGGTTCACCCCAAATACCACGCGCTGCTTATTCCCTGCTTGGCGGTGATCTTGGGGTTTGCCGTAGGtgcggtggtaatggcggtgtcaggtttgcacnnnnnnnnnnnnnnnnnnnnnnnnnnnnnnnnnntgtttggcgtGAATGTACAGGCTTTTGGCACCGGCAGGTCCGTGTGGAACTTCAGGTATATGGGCGAAGGAGTGGTGACGTGTCTGCCGCTGATACTCACAGGACTTGCGGTGGCATTTACGTCCCATATGGGATTGTTCAATATCGGGGCAGAAGGGCAGCTCGTAGTCGGTAGCGTGTGCGCAGTGTGTGTCGGTGTCCTTTGGCATGAGCACCTTTCTTTCTTTACCATTCCTGCGGCGGTTCTTGCAGGAATGGTAGGGGGAGGACTGTGGGGTTtgataccaggGGTGTTGCGCGCAGTGTGCGGGATCAGTGAGGTGGTGGTTACCATTAtgctcaactannnnnnnnngtatggggcgaattttgtagtcaccgctttgcctgggagcgacttgatgnnnnnnnnnnnnnnnnnnnnnnnnnnnnnnnnnnnnnnnnnnnnnnnCTCGCGTGTAAGCAATGGGTCGCGTCTGCATTGGGGCTTTTTGCTTGTGATAGCTGCACTGGTTAGCTTTAAGTTTCTCATTGAGAAAACAACGTTCGGCTATGAGCTCCGTGTCGTTGGTGCTAGTGCCGAAGCGGCCCGCTATGCGGGGattcacatnnnnnnnnnnnnnnnnnnnnnnncgagcatttcgggtatgtatgcagggctcnnnnnnnnnnnnnnnnnnnnnnnnnnnnnnnnnnnnnnnnnnnnnnnnnnnnnnnnnnnnnnnnnnnnnnnnnnnnnnnnnnnnnnnnnnnnnnnnnnnnnnnnnnCGGCGTGGGGGTGTGTgttcggcggttcgctgctcggttcgttgcnnnnnnnnnnnnnnnnnnnnnnnnnnnnnnnnnnnnnnnnnnnnnnnnnnnnnnnnnnnnnnnnnnnnnnnnnnnnnnnnnnnnnnnnnnnnnnnnnnatccgggcgatgctcgtcaggtgGGGGAGGCAGGGTGCGCACGTATGAACACGTTTTATTCGATGGTGGCGCTGACGCTTGTGTTTTCAACCCCTATTTTGATTACTGCGTTGGGGGGGTTGTTTTCCGAGCGGAGCGGGGTGATAAATATTGCCCTTGAAGGGTTGATGATGTTTGGTGCTTTTTCCACTGCTACGGTGACGGTCCTGTGCGAGCCGTATACGATagctgctccgtggatTGCACTGGGAGTTGGCATGGCAGTTGCCGCGTCGGTGGCGTTGTTTTACGCatatnnnnnnnnnnnnnnnnnnnnnnnnnnnnnnnnnnnnnnnnnnnnnnnnnnnnnnnnnnnnnnnnnnnnnnnnnnnnnnnnnnnnnnnnnnnnnnnnnnnnnnnnnnnnnnnnnnnnnnnnnnnnnnnnnnnnnnnnnnnnnaGTTACGGTTTTTTCAGTCGCATTCCGGTGCTTGGCCCGATGGTTTTCACCCATACGTACCCGACAGTATATCTAGGTTTTGTGCTAGTAGCATTGGCGTGGTACGTACTGTATCGCACGCCTTTCGGTGTGCACGTGCGTGCCACAGGGGATCAGCCGTATGCAGTAGACGGTGCGGGCTTGAGnnnnnnnnnnnnnnnnnnnnnnnnnnnnnnnnnnnnnnnnnnnnnnnnnnnnnnnnnnnnnnGGGTGCTGATACTGACGCAGGATATCCAATACACCGTCTACAGCACGCATGGGACGGGGTTTATCGCACTTGCAGCCTTGATTTCAGGACGGTGGCATCCTTTCGGGGTACTGGTGACAagcgttctttttggcttttcacagattttgaannnnnnnnnnnnnnnnnnnnnnnnnnnnnnnnnnnnnnnnnnnnnnnnnnnnnnnnnnnnnnnnnnnnnnnnnnnnnnnnnnnnnnnnnnnnnnnnnnnnnnnnnnnnnnnnnnnnnnnnnnnnnnnnnnnnnnnnnnnnnnnnnnnnnnnnnnnnnnnnnnnnnnnnnnnnnnnnnnnnnnnnnnnnnnnnnnnnnnnnnnnnnnnnnnnnnnnnnnnnnnnnnnnnnnnnnnnnnnnnnnnnnnnnnnnnnnnnnnnnnnnnnnnnnnnnnnnnnnnnnnnnnnnnnnnnnnnnnnnnnnnnnnnnnnnnnnnnnnnnnnnnnnnnnnnnnnnnnnnnnnnnnnnnnnnnnnnnnnnnnnnnnnnnnnnnnnnnnnnnnnnnnnnnnnnnnnnnnnnnnnnnnnnnnnnnnnnnnnnnnnnnnnnnnnnnnnnnnnnnnnnnnnnnnnnnnnnnnnnnccgcgcagttacgctgagaaatgttcgtgttcgggaagcagttcgcggtgagcaggttnnnnnnnnnnnnnnnnnnnnnnnnnnnnnnnnnnnnnnnnnnnnnnnnnnnnnnnnnnnnnnnnnnnnnnnnnnnnnnnnnnnnnnnnnnnnnnnnnnnnnnnnnnnnnnnnnnnnnnnnnnnnnnnnnnnnnnnnnnnnnnnnnnnnnnnnnnnnnnnnnnnnnnnnnnnnnnnnnnnnnnnnnnnnnnnnnnnnnnnnnnnnnnnnnnnnnnnnnnnnnnnnnnnnnnnnnggtgcgcagcgattccagacgcgcgcacctttctgtgccgcagggtaggttttcttttgcggaaactggCGCCTCGTTCGCTCTTTCTTGCGAAGCTGAGTATGTCGACACCCGTTCctcttcctggggaccgctgtacacacacctggacnnnnnnnnnnnnnntgaaacgtcgtttacgtcaggttccgccaccctcgAGCTTGCACCCCCGAGCGGCtcttttttcagtgtgccgacgcttactctcgtggcaatttacgcagatgacCTGTTTAAGTTTCACACGGCGCGGGGCATCTACCCTATGGAAGTTTCTGGGCAATGGAATACTGCAACCGGCGCTTGTGAAGCTTCCGTGCGCTGTGAAAATTTTCGTCCCCTTAAGTGGGCGCGGCTCCGCGACACCCACGTGCCAGCACAGGGTATGCAGGAATTGTCTGCGAGCGGGAACGTTCAGGTTGGGTATACCCCCATAGAACAGTGGCGGTGGAGTGCGGATGTGCACGCGCACACCCCGTATGTagtgcttgcgccggggtatcagctggaagacgttgtcgcaacgttacnnnnnnnnnnnnnnnnnnnnnnnnnnnnnnnnnnnnnnnnnnnnnnnnnnnnnnnnnnnnnnnnnnnnnnnnnnnnnnnnnnnnnnnnnnnnnnCCGCTGGATCCCTTCAGGGGTGCTTACGGTGCACAGGCTGCCGCTTCTTTCGGGGGCATACCTTTCAGCGCAGGTGCGTTTTCGCCCACAGGGGGTTGGTTTTGTGTGCACCGTCCCGCGGATACAGGnGGGGGAAGCGTTTCTGGAGGACGTGGCGCTCTCAGTACGTGTGGATCCGGCAAAAACGGATTTCCGCCTGGTGGCTGCAGacagcacggggcgctacgagtgtgacggatCATACCTTGCCGCGAATGCGGGGCAGTCTCGCTTTCTTGAGGCACACGTGGCGTTTGAATCGGTGAATGTCGGTGCGCTGTACCAAATGGTTGCTGCCTGTACGTCnnnnnnnnnnnnnnnnnnnnnnnnnnnnnnnnnnnnnnnnnnnnnnnnnnnnnnnnnnnnnnnnnnnnnnnnnnnnnnnnnnnnnnnnnnnnnnnnnnnnnnnnnnnnnnnnnnnnnnnnnnnnnnnnnnnnnnnnnnnnnnnnnnnnnnnnnnnnnnnnnnnnnnnnnnnnnnnnnnnnnnnnnnnnnnnnnnnnnnnnnnnnnnnnnnnnnnnnnnnnnnnnnnnnnnnnnnnnnnnnnnnnnnnnnnnnnnnnnnnnnnnnnnnnnnnnnnnnnnnnctatccatcaattcggttccgtatcgtaccaggggagtatatgccgaccgtacgctgacggtgtatggcGACTATGATTTTTCGGTGGTGGCATCGTTTGACGAGCGCGCAGGGGTTACCGGCACGTTTCAGGTGCAGAATCTGCCGGTTCctctctctcagagtctttttgattgtgacagttcttttgnnnnnnnnnnnnnnnnnnnnnnnnnnnnnnnnnnnnnnnnnnnnnnnnnnnnnnnnnnnnnnnnnnnnnnnnnnnnnnnnnnnnnnnnnnnnnnnnnnnnnnnnnnnnnnnnnnnnnnnnnnnnnnnnnnnnnnnnnnnnnnnnnnnnnnnttttggttctcgcgatcggtacttggctggaacggcgagctttgccgttgtgccgagaacagggcagcacgagcaggcgcggtatgaaacggccgtgcgccttgCATCTGAAGATGCGCAGGAGCAGGTgcagcttaacGCGCAGGTAACCGTGGGGGAACACGTCTATGTGGATAGCTCAGGGCGAATAGATAACGTAGACGTGGGGCGTTTTGTTGCAGGGCAGGGGGAGCGCAGTCGCGTCACCGGGTCGTGGACTGTGCTGGGTACGATGCAGGATATGTCTGGACAGGTGCAGGTAGATTCACTCGAGCTGATCGccaagggagTGCCCTTTCACCTGCGGGGAGGATGTGCACTTGATGACGGTACGCTTGCGCTTTTGCCCACCCAGGTGAcgtgggggtcacatcagtttgctgaccttgcaggagaatgggtgccgggtcaggcgcgtgcgtgGGTGCGCACCACGTACTCAGGCGCGTTTGAAGGGCAGCCGACACATGCCACCTGTACGCTCACCCTTGCCGGATCCcctgtggattcgggtaaGGCGACATCTGCACTGCGCACGTCGTTTCTCACGCCATTTTTGCAGACGCACAGTCAATACAcgatttctgcggagtttgagcactggcgcatcgTCACATACGAGGGTGAAAAGAACCGCATACTGGTAGTGCGCGATCCGGGCGTATGGgcgctgtacgccggtgagcacgacGAAATTACCGGATTTATGCTGGATGATGGTTCAGTGTCGTTGCAGGTGGCGCAGAGTTTGCCTGTTCATTTtttcttgaacgggtcGTTGAGTGCACAGCAGGTAGACGTGCAGATTCAGGATATCTTTGTTGATTTGGCGCGCGTATGGGcgtttacgggcatacggcatgnnnnnnnnnnnnnnnnnnnnnnnnnnnnnnnnnnnnnnnnnnnnnnnnnnnnnnnnnnnnnnnnnnnnnnnnnnnnnnnnnnnnnnnnnnnnnnnnnnnnnnnnnngcctgggtatgcacctgagcgctttgggccaggttctatcgatatagtagcacacggcagcacgctcatagtgnnnnnnnnnnnnnnnnnnnnnnnnnnnnnnnnnnnnnnnnnnnnnnnnnnnnnnnnnnnnnnnnnnnnnnnnnnnnnnnnnnnnnnnnnnnnnnnnnnnnnnnnnnnnnnnnnnnnnnnnnnnnnnnnnnnnnnnnnnnnnnnnnnnnnnnnnnnnnnnnnnnnnnnnnnnnnnnnnnnnnnnnnnnnnnnnnnnnnnnnnnnnnnnnnnnnnnnnnnnnnnnnnnnnnnnnnnnnnnnnnnnnnnnnnnnnnnnnnnnnnnnnnnnnnnnnnnnnnnnnnnnnnnnnnnnnnnnnnnnnnnnnnnnnnnnnnnnnnnnnnnnnnnnnnnnnnnnnnnnnnnnnnnnnnnnnnnnnnnnnnnnnnnnnnnnnnnnnnnnnnnnnnnnnnnnnnnnnnnnnnnnnnnnnnnnnnnnnnnnnnnnnnnnnnnnnnnnnnnnnnnnnnnnnnnnnnnnnnnnnnnnnnnnnnnnnnnnnnnnnnnnnnnnnnnnnnnnnnnnnnnnnnnnnnnnnnnnnnnnnnnnnnnnnnnnnnnnnnnnnnnnnnnnnnnnnnnnnnnnnnnnnnnnnnnnnnnnnnnnnnnnnnnnnnnnnnnnnnnnnnnnnnnnnnnnnnnnnnnnnnnnnnnnnnnnnnnnnnnnnnnnnnnnnnnnnnnnnnnnnnnnnnnnnnnnnnnnnnnnnnnnnnnnnnnnnnnnnnnnnnnnnnnnnnnnnnnnnnnnnnnnnnnnnnnnnnnnnnnnnnnnnnnnnnnnnnnnnnnnnnnnnnnnnnnnnnnnnnnnnnnnacgcagtgggggATTATGAAGCGGGTGGAGGATAAAATCCGCTCATTTTTGGATTTGGACGCGTTTTCGTTCCGCACCTATGTTCTGCAGAACGCGATTTTTGGGAATTTGTTCAATAAGGACCGCAGCAAGCCGCTGACAGTGGGTAACTATTTTGACAATAcctccctctacgtnnnnnnnnnnnnnnnnnnnnnnnnnnnnncggatgcgctgcttcacttgtctcaGTATGATCCGCTTGCGCCAAATAATTTGGGGATTAAAAAGCCTGCGGCAGGGAGtttgctgttccggccggagctggggctagagtttgcaacgccctttttttcgttgcggtgggcgtcgacgccnnnnnnnnnngattcactgtttgtctctgatacttcaatgcgggtgtcgtggagttttgcgtattgaggnnnnnnnnnnnnnnnnnnnnnnnnnnnnnnnnnnnnnnnnnnnnnnnnnnnnnnnnnnnnnnnnnnnnnnnnnnnnnnnnnnnnnnnnnnnnnnnnnnnnnnnnnnnnnnnnnnnnnnnnnnnnnnnnnnnnnnnnnnnnnnnnnnnnnnnnnnnnnnnnnnnnnnnnnnnnnnnnnnnnnnnnnnnnnnnnnnnnnnnnnnnnnnnnnnnnnnnnnnnnnnnnnnnnnnnnnnnnnnnnnnnnnnnnnnnnnnnnnnnnnnnnnnnnnnnnnnnnnnnnnnnnnnnnnnnnccgatccggagtatcagtatgtgatgctacagttcacggtaaaggagcgtccttcggtgaagggcatcaagatggtagggaacagccaaatccgcagtggggaccttttgtctaaaatcctcctgaaaaagggagacatnnnnnnnnnnnnnnnnnnnnnnnnnnnnnnnnnnnnnnnnnnnnnnnnnnnnnnnnnnnnnnnnnnnnnnnnnnnnnnnnnnnnnnnnnnnnnnnnnnnnnnnnnnnnnnnnnnnnnnnnnnnnnnnnnnnnnnnnnnnnnnnnnnnnnnnnnnnnnnnnnnnnnnnnnnnnnnnnnnnnnnnnnnnnnnnnnnnnnnnnnnnnnnnnnnnnnnnnnnnnnnnnnnnnnnnnnnnnnnnnnnnnnnnnnnnnnnnnnnggagaatgcgctggaagcggataagnnnnnnnnnnnnnnnnnCTATGCAGAGAGGGGATACATTGACGCGCGGGTAGAAGGCGTGGCAAnnnnnnnnnnnnnnnnnnnnnnnnnnnnnnnnnnnnnnnnnnnnnnnnnnnnnnnnnnnnnnnnnnnnnnnnnnnnnnnnnnnnnnnnnnnnnnnnnnnnnnnnnnnnnnnnnnnnnnnnnnnnnnnnnnnnnnnnnnnnnnnnnnnnnnnnnnnnnnnnnnnnnnnnnnnnnnnnnnnnnnnnnnnnnnnnnnnnnnnnnnnnnnnnnnnnnnnnnnnnnnnnnnnnnnnnnnnnnnnnnnnnnnnnnnnnnnnnnnnnnnnnnnnnnnnnnnnnnnnnnnnnnnnnnnnnnnnnnnnnnnnnnnnnnnnnnnnnnnnnnnnnnnnnnnnnnnnnnnnnnnnnnnnnnnnnnnnnnnnnnnnnnnnnnnnnnnnnnnnnnnnnnnnnnnnnnnnnnnnnnnnnnnnnnnnnnnnnnnnnnnnnnnnnnnnnnnnnnnnnnnnnnnnnnnnnnnnnnnnnnnnnnnnnnnnnnnnnnnnnnnnnnnnnnnnnnnnnnnnnnnnnnnnnnnnnnnnnnnnnnnnnnnnnnnnnnnnnnnnnnnnnnnnnnnnnnnnnnnnnnnnnnnnnnnnnnnnnnnnnnnnnnnnnnnnnnnnnnnnnnnnnnnnnnnnnnnnnnnnnnnnnnnnnnnnnnnnnnnnnnnnnnnnnnnnnnnnnnnnnnnnnnnnnnnnnnnnnnnnnnnnnnnnnnnnnnnnctgaagctcgggtatgtggaGCGCTGGTTTCTGGGCTCTCCGCTGACGGTGGGCTTTGACTTTGAACTTACGCACAAAAATCTCTTTGTGTACCGCGCAGGTGCAAAAGGCAACGGGCTGCCGCACCCGTACGTGAGCAAGGAGCACTGGGCTAATTCCCCTGGGCTGGCAGAATCGTTTCGCCTCAAGTATTCGCGCTTTGAGTCCGCCATCGGCGCGCACACCGGGTACCAGtggtatccgcgctatgcggtcattagggtgaacgggggggtggannnnnnnnnnnnnnnnnnnnnnnnnnnnnnnnnnnnnnnnnnnnnnnnnnnnnnnnnnnnnnnnnnnnnnnnnnnnnnnnnnnnnnnnnnnnnnnnnnnnnnnnnnnnnnnnnnnnnnnnnnnnnnnnnnnnnnnnnnnnnnnnnnnnnnnnnnnnnnnnnnnnnnnnnnnnnnnnnnnnnnnnnnnnnnnnnnnnnnnnnnnnnnnnnnnnnnnnnnnnnnnnnnnnnnnnnnnnnnnnnnnnnnnnnnnnnnnnnnnnnnnnnnnnnnnnnnnnnnnnnnnnnnnnnnnnnnnnnnnnnnnnnnnnnnnnnnnnnnnnnnnnnnnnnnnnnnnnnnnnnnnnnnnnnnnnnnnnnnnnnnnnnnnnnnnnnnnnnnnnnnnnnnnnnnnnnnnnnnnnnnnnnnnnnnnnnnnnnnnnnnnnnnnnnnnnnnnnnnnnnnnnnnnnnnnnnnnnnnnnnnnnnnnnnnnnnnnnnnnnnnnnnnnnnnnnnnnnnnnnnnnnnnnnnnnnnnnnnnnnnnnnnnnnnnnnnnnnnnnnnnnnnnnnnnnnnnnnnnnnnnnnnnnnnnnnnnnnnnnnnnnnnnnnnnnnnnnnnnnnnnnnnnnnnnnnnnnnnnnnnnnnnnnnnnnnnnnnnnnnnnnnnnnnnnnnnnnnnnnnnnnnnnnnnnnnnnnnnnnnnnnnnnnnnnnnnnnnnnnnnnnnnnnnnnnnnnnnnnnnnnnnnnnnnnnnnnnnnnnnnnnnnnnnnnnnnnnnnnnnnnnnnnnnnnnnnnnnnnnnnnnnnnnnnnnnnnnnnnnnnnnnnnnnnnnnnnnnnnnnnnnnnnnnnnnnnnnnnnnnnnnnnnnnnnnnnnnnnnnnnnnnnnnnnnnnnnnnnnnnnnnnnnnnnnnnnnnnnnnnnnnnnnnnnnnnnnnnnnnnnnnnnnnnnnnnnnnnnnnnnnnnnnnnnnnnnnnnnnnnnnnnnnnnnnnnnnnnnnnnnnnnnnnnnnnnnnnnnnnnnnnnnnnnnnnnnnnnnnnnnnnnnnnnnnnnnnnnnnnnnnnnnnnnnnnnnnnnnnnnnnnnnnnnnnnnnnnnnnnnnnnnnnnnnnnnnnnnnnnnnnnnnnnnnnnnnnnnnnnnnnnnnnnnnnnnnnnnnnnnnnnnnnnnnnnnnnnnnnnnnnnnnnnnnnnnnnnnnnnnnnnnnnnnnnnnnnnnnnnnnnnnnnnnnnnnnnnnnnnnnnnnnnnnnnnnnnCTATTAGGCGAATTGCAGAAAGTGAAGGCTACAGCATCGTCTTAGATCTGCAAAAAAACGCCGGAATACTCTGGTACAGCCACTCGGTCGATATTACCGAAGACGTCCTGCGGGAGCTGAGCAGCTCGTGATGCACcgtgagcaccgcgtcnnnnnnnnnnnnnnnnnnnnnnnnnnnnnnnnnnnnnnnnnnnnnnnnnnnnnnnnnnnnnnnnnnnnnnnnnnnnnnnnnnnnnnnnnnnnnnnnnnnnnnnnnnnnnnnnnnnnnnnnnnnnnnnnnnnnnnnnnnnnnnnnnnnnnnnnnnnnnnnnnnnnnnnnnnnnnnnnnnnnnnnnnnnnnnnnnnnnnnnnnnnnnnnnnnnnnnnnnnnnnnnnnnnnnnnnnnnnnnnnnnnnnnnnnnnnnnnnnnnnnnnnnnnnnnnnnnnnnnnnnnnnnnnnnnnnnnnnnnnnnnnnnnnnnnnnnnnnnnnnnnnnnnnnnnnnnnnnnnnnnnnnnnnnnnnnnnnnnnnnnnnnnnnnnnnnnnnnnnnnnnnnnnnnnnnnnnnnnnnnnnnnnnnnnnnnnnnnnnnnnnnnnnnnnnnnnnnnnnnnnnnnnnnnnnnnnnnnnnnnnnnnnnnnnnnnnnnnnnnnnnnnnnnnnnnnnnnnnnnnnnnnnnnnnnnnnnnnnnnnngagttcggacgtgtccagccgtctgaggttctcctgtctgcttcagtgctccgttcactGCCTGAACTTGCCGCTATCCTCAGTCTCTACCCCCGGCTCGTTCGTACCACCGGCGCAGATGCGCTTnnnnnnnnnnnnnnnnnnnnnnnnnnnnnnnnnnnnnnnnnnnnnnnnnnnnnnnnnnnnnnnnnnnnnnnnnnnnnnnnnnnnnnnnnnnnnnnnnnnnnnnnnnnnnnnnnnnnnnnnnnnnnnnnnnnnncgCGACACCCGCTCTCCCACGTCAGTGCCATCACCCGCTACCATATCCATGACTTTGTAGAAATCGATGACGCTACGCGCAAAAATCTAGAGATACTTCAAAATCTCCACGACAGCACCCATGCGCATTCTCTTTTTGAAACACTCAACTATACACACACCGCCATGGGTACCAGGCTCCTGCGCTATTGGCTGCACCACCCCTTGCGCTCCCAGGAGGAAATTCAAAAACGCCTCAGTGCAGTGGTCTTTTTTCATCACCGTCCCCACATCCTCAAGACACTGCGTGCAACACTCTCGTGTgttcgggatgtggagcgcctagncgcccgcgtggcgttagaaaaggcgcacggacgnnnnnnnnnnnnnnnnnnnnnnnnnnnnnnnnnnnnnnnnnnnnnnnnnnnnnnnnnnnnnnnnnnnnnnnnnnnnnnnnnnnnnnnnnnnnnnGAAGGGGATACCCCGGTGCTGCAGGAACTGTATGGTCTTTTAGAACAGTCTATCAAAGAAGATTGCCCCGTAACGCTAAGCGATGGGAACcttatcaagcgtggtttttctgcgtccttagatgaactgcaccgcgnnnnnnnnnnnnnnnnnnnnnnnnnnnnnnnnnnnnnnnnagaggagcgtgagcgcacgggtatcggtacattaaaaatgaagtacaatcgcatgctcgGTCACTTTCTGGAGGTATCCAAAGGgcatctttctgctgtccctgcgcactttattcgtcgccgttcactgagcaatgcnnnnnnnnnnnnnnnnnnnnnnnnnnnnnnnnnnnnnnnnnnnnnnnnnnnnnnnnnnnnnnnnnnnnnnnnnnnnnnnnnnnnnnnnnnnnnnnnnnnnnnnnnnnnnnnnnnnnnnnnnnnnnnnnnnnnnnnnnnnnnnnnnnnnnnnnnnnnnnnnnnnnnnnnnnnnnnnnnnnnnnnnnnnnnnnnnnnnnnnnnnnnnnnnnnnnnnnnnnnnnnnnnnnnnnnnnnnnnnnnnnnnnnnnnnnnnnnnnnnnnnnnnnnnnnnnnnnnnnnnnnnnnnnnnnnnnnnnnnnnnnnnnnnnnnnnnnnnnnnnnnnnnnnnnnnnnnnnnnnnnnnnnnnnnnnnnnnnnnnnnnnnnnnnnnnnnnnnnnnnnnnnnnnnnnnnnnnnnnnnnnnnnnnnnctcatttgcctgattgcgcaggttggctcctttgTCCCTGCAGAAAAGGCAGAGCTCACCCCCGTCGATCGTATTTTTTGTCGGGTAGGAGCGGCCGATAACCTTGCGCGCGGGGAATCTACCTTCTTGGTAGAAATGAGTGAAACAGCACnnnnnnnnnnnnnnnnnnnnnnnnnnnnnnnnnnnnnnnnnnnnnnnnnnnnnnnnnnnnnnnnnnnnnnnnnnnnnnnnnnnnnnnnnnnnnnnnnnnnnnnnnnnnnnnnnnnnnnnnnnnnnnnnnnnnnnnnnnnnnnnnnnnnnnnnnnnnnnnnnnnnnnnnnnnnnnnnnnnnnnnnnnnnnnnnnnnnnnnnnnnnnnnnnnnnnnnnnnnnnnnnnnnnnnnnnnnnnnnnnnnnnnnncccggttcttgcggcagttcgtacggcatttacgttgcgcgtctggcggggctccctgaatcggtactggcacgcgcgtgtgagnnnnnnnnnnnnnnnnnnnnnnnnnnnnnnnnnnnnnnnnnnnnnnnnnnnnnnnnnnnnnnnnnnnnnnnnnnnnnnnnnnnnnnnnnnnnnnnnnnnnnnnnnnnnnnnnnnnnnnnnnnnnnnnnnnnnnnnnnnnnnnnnnnnnnnnnnnnnnnnnnnnnnnnnnnnnnnnnnnnnnnnnnnnnnnnnnnnnnnnnnnnnnnnnnnnnnnnnnnnnnnnnnnnnnnnnnnnnnnnnnnnnnnnnnnnnnnnnnnnnnnnnnnnnnnnnnnnnnnnnnnnnnnnnnnnnnnnnnnnnnnnnnnnnnnnnnnnnnnnnnnnnnnnnnnnnnnnnnnnnnnnnnnnnnnnnnnnnnnnnnnnnnnnnnnnnnnnnnnnnnnnnnnnnnnnnnnnnnnnnnnnnnnnnnnnnnnnnnnnnnnnnnnnnnnnnnnnnnnnnnnnnnnnnnnnnnnnnnnnnnnnnnnnnnnnnnnnnnnnnnnnnnnnnnnnnnnnnnnnnnnnnnnnnnnnnnnnnnnnnnnnnnnnnnnnnnnnnnnnnnnnnnnnnnnnnnnnnnnnnnnnnnnnnnnnnnnnnnnnnnnnnnnnnnnnnnnnnnnnnnnnnnnnnnnnnnnnnnnnnnnnnnnnnnnnnnnnnnnnnnnnnnnnnnnnnnnnnnnnnnnnnnnnnnnnnnnnnnnnnnnnnnnnnnnnnnnnnnnnnnnnnnnnnnnnnnnnnnnnnnnnnnnnnnnnnnnnnnnnnnnnnnnnnnnnnnnnnnnnnnnnnnnnnnnnnnnnnnnnnnnnnnnnnnnnnnnnnnnnnnnnnnnnnnnnnnnnnnnnnnnnnnnnnnnnnnnnnnnnnnnnnnnnnnnnnnnnnnnnnnnnnnnnnnnnnnnnnnnnnntgaggaacagtgggaagtactgcgccagaaaatgnnnnnnnnnnnnnnnnnnnnnnnnnnnnnnnnnnnnnnnnnnnnnnnnnnnnnnnnnnnnnnnnnaaatgtttcaggacgaatgtttcgtgtggtgtcctacgcggtggaccgagacnnnnnnnnnnnnnnnnnnnnnnnnnnnnnnnnnnnngcaaagcgggaaagaccacttattttacttgccggatacagcgcgtatcctcgttccattaatnnnnnnnnnnnnnnnnnnnnnnnnnnnnnnnnnnnnnnnnnnnnnnnnnnnnnnnnnnnnnnnnnnnnnnnnnnnnnnnnnnnnnnnnnnnnnnnnnnnnnnnnnnnnnnnnnnnnnnnnnnnnnnnnnnnnnnnnnnnnnnnnnnnnnnnnnnnnnnnnnnnnnnnnnnnnnnnnnnnnnnnnnnnnnnnnnnnnnnnnnnnnnnnnnnnnnnnnnnnnnnnnnnnnnnnnnnnnnnnnnnnnnnnnnnnnnnnnnnnnnnnnnnnnnnnnnnnnnnnnnnnnnnnnnnnnnnnnnnnnnnnnnnnnnnnnnnnnnnnnnnnnnnnnnnnnnnnnnnnnnnnnnnnnnnnnnnnnnnnnnnnnnnnnnnnnnnnnnnnnnnnnnnnnnnnnnnnnnnnnnnnnnnnnnnnnnnnnnnnnnnnnnnnnnnnnnnnnnnnnnnnnnnnnnnnnnnnnnnnnnnnnnnnnnnnnnnnnnnnnnnnnnnnnnnnnnnnnnnncctttgacccaaacggcgcatggctcaccagcggactgcgcatcggaacccccgcggtaacgagccttggaatgggnnnnnnnnnnnnnnnnnnnnnnnnnnnnnnGATCGCGCGCGTGCTCGGCGCTGCAACGCCTGTGCGGACAAAGACaggtgcgctaagcaaatcggcggccgaggtgcccggcgaggttagaagctcagtctgctcggaagtgcgggagctgctcgcacgCTTCACGTtgtaccctgaactcgacgaacccttcttgcgcgcacaCTTTACGCGTCGCCCTGCGGACAAAACACCTGCCGACGAAGGGACTTGAACCCTTACGGGGTTACCCCAACAGATTTTGAGTCTGTCGTGTCTGCCAGTTTCACCAnnnnnnnnnnnnnnnnnnnnnnnnnnnnnnnnnnnnnnnnnnnnnnnnnnnnnnnnnnnnnnnnnnnnnnnnnnnnnnnnnnnnnnnnnnnnnnnnnnnnnnnnnnnnnnnnnnnnnnnnnnnnnnnnnnnnnnnnnnnnnnnnnnnnnnnnnnnnnnnnnnnnnnnnnnnnnnnnnnnnnnnnnnnnnnnnnnnnnnnnnnnnnnnnnnnnnnnnnnnnnnnnnnnnnnnnnnnnnnnnnnnnnnnnnnnnnnnnnnnnnnnnnnnnnnnnnnnnnnnnnnnnnnnnnnnnnnnnnnnnnnnnnnnnnnnnnnnnnnnnnnnnnnnnnnnnnnnnnnnnnnnnnnnnnnnnnnnnnnnnnnnnnnnnnnnnnnnnnnnnnnnnnnnnnnnnnnnnnnnnnnnnnnnnnnnnnnnnnnnnnnnnnnnnnnnnnnnnnnnnnnnnnnnnnnnnnnnnnnnnnnnnnnnnnnnnnnnnnnnnnnnnnnnnnnnnnnnnnnnnnnnnnnnnnnnnnnnnnnnnnnnnnnnnnnnnnnnnnnnnnnnnnnnnnnnnnnnnnnnnnnnnnnnnnnnnnnnnnnnnnnnnnnnnnnnnnnnnnnnnnnnnnnnnnnnnnnnnnnnnnnnnnnnnnnnnnnnnnnnnnnnnnnnnnnnnnnnnnnnnnnnnnnnnnnnnnnnnnnnnnnnnnnnnnnnnnnnnnnnnnnnnnnnnnnnnnnnnnnnnnnnnnnnnnnnnnnnnnnnnnnnnnnnnnnnnnnnnnnnnnnnnnnnnnnnnnnnnnnnnnnnnnnnnnnnnnnnnnnnnnnnnnnnnnnnnnnnnnnnnnnnnnnnnnnnnnnnnnnnnnnnnnnnnnnnnnnnnnnnnnnnnnnnnnnnnnnnnnnnnnnnnnnnnnnnnnnnnnnnnnnnnnngcgttatacaatctcttttttacaaagacatttcctgcaacgtatgaaaggtcgcaggatgtgtcgacaaattccaacaaACCGCCTGCTTCGTGCTCAATAATTTCCTGTTTCTTCCTTTTTAGAAATTGTAAGGTTGCAGnnnnnnnnnnnnnnnnnnnnnnnnnnnnnnnnnnnnnnnnnnnnnnnnnnnnnnnnnnnnnnnnnnnnnnnnnnnnnnnnnggtggaggnnnnnnnnnnnnnnnnnnnnnnnnnnnnnnnnnnnnnnnnnnnnnnnnnnnnnnnnnncaagtactaaaatctcctcctgggttttgagatattcaagaaagcgtatccgtgtttcttcatttggcaggggtatgtgaatcttnnnnnnnnnnnnnnnnnnnnnnnnnnnnnnnnnnnnnnnnnnnnnnnnnnnnnnnnnnnnnnnnnnnnnnnnnnnnnnnnnnnnnnnnnnnnnnnnnnnnnnnnnnnnnnnnnnnnnnnnnnnnnnnnnnnnnnnnnnnnnnnnnnnnnnnnnnnnnnnnnnnnnnnnnnnnnnnnnnnnnnnnnnnnnnnnnnnnnnnnnnnnnnnnnnnnnnnnnnnnnnnnnnnnnnnnnnnnnnnnnnnnnnnnnnnnnnnnnnnnnnnnnnnnnnnnnnnnnnnnnnnnnnnnnnnnnnnnnnnnnnnnnnnnnnnnnnnnnnnnnnnnnnnnnnnnnnnnnnnnnnnnnnnnnnnnnnnnnnnnnnnnnnnnnnnnnnnnnnnnnnnnnnnnnnnnnnnCCCGGTTACCGAAGATAACCTCAGATATGTAGTCATTAATGCGAACGAAGGAGAagctgccctgaatatcgcgatgggggaggaaGTCACGGATATTGCCATGTACGAAGTACAGATTGACCGTTTTAGAGCaatacttcttggacaactccnnnnnnnnnnnnnnnnnnnnnnnnnnnnnnnnnnnnnnnnnnnnnnnnnnnnnnnnnnnnnnnnnnnnnnnnnnnnnnnnnnnnnnnnnnnnnnnnnnnnnnnnnnnnnnnnnnnnnnnnnnnnnnnnnnnnnnnnnnnnnnnnnnnnnnnnnnnnnnnnnnnnnnnnnnnnnnnnnnnnnnnnnnnnnnnnnnnnnnnnnnnnnnnnnnnnnnnnnnnnnnnnnnnnnnnnnnnnnnnnnnnnnnnnnnnnnnnnnnnnnnnnnnnnnnnnnaaaggcaagcgacgggaggatcccctatattgagtaggttcctgcaatggtatcaccgccggtgcctgtccagtttgtgtgaaaaaactctcctctcggcgcatctgtgcgctcgtaggtgtgcgcaccaaaataatctcgctgtgccnnnnnnnnnnnnnnnnnnnnnnnnnnnnnnnnnnnnnnnnnnnnnnnnnnnnnnnnnnnnnnnnnnnnnnnnnnnnnnnnnnnnnnnnnnnnnnnnnnnnnnnnnnnnnnnnnnnnnnnnnnnnnnnnnnnnnnnnnnnnnnnnnnnnnnnnnnnnnnnnnnnnnnnnnnnnnnnnnnnnnnnnnnnnnnnnnnnnnnnnnnnnnnnnnnnnnnnnnnnnnnnnnnGAATAATACACCCGCCACGCCACAGCGATGCAATCCGGGAAAAATCCAGTGTCCATCCTCGGCGCTTTGCCGTATGCGATAACAGCTCAAAACCCTGCGCATACGAGACTATTTTCGCGCAATACAGCGCGTCTTCCAGTGCAGACACCAGTTCTTCGCGCTGCTGTGCACttagcgtttctgctttggagactttcacgggagaaccaaaaacgcgatgtgccttgcagcgcgcnnnnnnnnnnnnnnnnnnnnnnnnnnnnnnnnnnnnnnnnnnnnnnnnnnnnnnnnnnnnnnnnnnnnnnnnnnnnnnnncacacgtccacctgcccgtccccttctgtccagcggcaTCTAGAATTTTCTCTAAAAGTGGTGTGCCGTCTGTGTCCTGATGTGCCAGAATAGCCGCGGTAATCTCAATCAGGTACGAGTGTAAGCGGCCCGTGTTCCAGCGGGTAAACGTATGGTGCATGTGCTCATAGCTCATGCCCAGCGCATGCTtcataaaccagtagccctcggcgattatnnnnnnnnnnnnnnnnnnnnnnnnnnnnnnnnnnnnnnnnnnnnnnnnnnnnnnnnnnnnnnnnnnnnnnnnnnnnnnnnnnnnnnnnnnnnnnnnnnnnnnnnnnnnnnnnnnnnnnnnnnnnnnnnnnnnnnnnnnnnnnnnnnnnnnnnnnnnnnnnnnnnnnnnnnnnnnnnnnnnnnnnnnnnnnnnnnnnnnnnnnnnnnnnnnnnnnnnnnnnnnnnnnnnnnnnnnnnnnnnnnnnnnnnnnnnnnnnnnnnnnnnnnnnnnnnnnnnnnnnnnnnnnnnnnnnnnnnnnnnnnnnnnnnnnnnnnnnnnnnnnnnnnnnnnnnnnnnnnnnnnnnnnnnnnnnnnnnnnnnnnnnnnnnnnnnnnnnnnnnnnnnnnnnnnnnnnnnnnnnnnnnnnnnnnnnnnnatggagtgggcgccggtgattcgcttgccatgagcgcgccctgcaagaaatcggtcgaccaccgtggtggtgcgattgaaaactgcgacggaaaaaccgttgcgctcaatgttgagaaccagattctctcccatgacagcaagtccaannnnnnnnnnnnnnnnnnnnnnnnnnnnnnnnnnnnnnnnnnnnnnnnnnnnnnnnnnnnnnnnnnnnnnnnnnnnnnnnnnnnnnnnnnnnnnnnnnnnnnnnnnnnnnnnnnnnnnnnnnnnnnnnnnnnnnnnnnnnnnnnnnnnnnnnnnnnnnnnnnnnnnnnnnnnnnnnnnnnnnnnnnnnnnnnnnnnnnnnnnnnnnnnnnnnnnnnnnnnnnnnnnnnnnnnnnnnnnnnnnnnnnnnnnnnnnnnnnnnnnnnnnnnnnnnnnnnnnnnnnnnnnnnnnnnnnnnnnnnnnnnnnnnnnnnnnnnnnnnnnnnnnnnnnnnnnnnnnnnnnnnnnnnnnnnnnnnnnnnnnnnnnnnnnnnnnnnnnnnnnnnnnnnnnnnnnnnnnnnnnnnnnnnnnnnnnnnnnnnnnnnnnnnnnnnnnnnnnnnnnnnnnnnnnnnnnnnnnnnnnnnnnnnnnnnnnnnnnnnnnnnnnnnnnnnnnnnnnnnnnnnnnnnnnnnnnnnnnnnnnnnnnnnnnnnnnnnnnnnnnnnnnnnnnnnnnnnnnnnnnnnnnnnnnnnnnnnnnnnnnnnnnnnnnnnnnnnnnnnnnnnnnnnnnnnnnnnnnnnnnnnnnnnnnnnnnnnnnnnnnnnnnnnnnnnnnnnnnnnnnnnnnnnnnnnnnnnnnnnnnnnnngcagcagaaacatttaaatctttgcgcgtgcttgtctcggcacataccaagcttatcnnnnnnnnnnnnnnnnnnnnnnnnnnnnnnnnnnnnnnnnnnnnnnnnnnnnnnnnnnnnnnnnnnnnnnnnnnnnnnnnnnnnnnnnnnnnnnnnnnnnnnnnnnnnnnnnnnnnnnnnnnnnnnnnnnnnnnnnnnnnnnnnnnnnnnnnnnnnnnnnnnnnnnnnnnnnnnnnnnnnnnnnnnnnnnnnnnnnnnnnnnnnnnnnnnnnnnnnnnnnnnnnnngtctcgaacaggctgcgcacgagacgagtatcgcacgccgttacctggaggcgctcgagaacgatgagtatgatgtttttcccggcgaaccctacatccttggctttttgcgcaATTACTGCGagtacctccagctggatacggagcagtgcatcgctcgctataaannnnnnnnnnnnnnnnnnnnnnnnnnnnnnnnnnnnnnnnnnnnnnnnnnnnnnnnnnnnnnnnnnnnnnnnnnnnnnnnnnnnnnnnnnnnnnnnnnnnnnnnnnnnnnnnnnnnnnnnnnnnnnnnnnnnnnnnnnnnnnnnnnnnnnnnnnnnnnnnnnnnnnnnnnnnnnnnnngagcacagcgtaccccaagggagctgtctccccccgatgcaacggggnnnnnnnnnnnnnnnnnnnnnnnnnnnnnnnnnnnnnnnnnnnnnnnnnnnnnnnnnnnnnnnnnnnnnnnnnnnnnnnnnnnnnnnnntgaggaaaagtttgannnnnnnnnnnnnnnnnnnnnnnnnnnnnnnnnnnnnnnnnnnnnnnnnnnnnnnnnnnnnnnnnnnnnnnnnnnnnnnnnnnnnnnnnnnnnnnnnnnnnnnnnnnnnnnnnnnnngatctctcttGGTCAGCGCctagtgatggATTTGAATACAGATGTGCAGCCGGACGTAGAAATAAGTGTGGAAGACATTGAAGCACATCAGGCGGACGGGGGCGCGCGTGTTCGCGTGTTTACAGGTAGTCTGGTGCAGACGCTCCGTGATCGCAGTGCTCagagctttgtgcctacaagnnnnnnnnnnnnnnnnnnnnnnnnnnnnnnnnnnnnnnnnnnnnnnnnnnnnnnnnnnnnnnnnnnnnnnnnnnnnnnnnnnnnnnnnnnnnnnnnnnnnnnnnnnnnnnnnnnnnnnnnnnnnnnnnnnnnnnnnnnnnnnnnnnnnnnnnnnnnnnnnnnnnnnnnnnnnnnnnnnnnnnnnnnnnnnnnnnnnnnnnnnnnnnnnnnnnnnnnnnnnnnnnnnnnnnnnnnnnnnnnnnnnnnnnnnnnnnnnnnnnnnnnnnnnnnnnnnnnnnnnnnnnnnnnnnnnnnnnnnnnnnnnnnnnnnnnnnnnnnnnnnnnngatgaggacgccgggcggttcaagttcgtggtcatggaagtagactagcacgcggcggcagcaatcgcgtacgcgttccAGAGCGCGTGGACTGCAGCGCACAGTGCGCTTGCGCGCGCGCGGGAGCCGCTTCTTTTTTTCTCTCTTACAAaaagtacccgtagcgctgcgcccgcagctcctgcaaacagcgtggcgctgcctgcgggccggtGTGCAAGAGCAAAGAGaaggactgacagtacctcgccacaggcgcgtgcagtgcaggaagtggcatggtggcacagacgctcaggtatataggcgcgaaagagcacttcttcgctcagagcatttaaaaaaagccgtacataAAAAGCTCCcccttccnnnnnnnnnnnnnnnnnnnnnnnnnnnnnnnnnnnnnnnnnnnnnnnnnnnnnnnnnnnnnnnnnnnnnnnnnnnnnnnnnnnnnnngccgccgcagcgtgcagtacgtgtggcgtgttgcgcaaaggggagaggnnnnnnnnnnnnnnnnnnnnnnnnnnnnnnnnnnnnnnnnnnnnnnnnnnnnnnnnnnnnnnnnnnnnnnnnnnnnnnnnnnnnnnnnnnnnnnnnnnnnnnnnnnnnnnnnnnnnnnnnnnnnnntttccgtatgcattctttggtgcagtacaggcgtgtgtgctgtGTATTGGGTGTTTGTTGGTCCGCAGTGGTGTGCGGTTCTTTTCtcgttggggtgctgtccgtatctggaggaggtggggaatcgcatacaccagcgtatgtcggtgttgtaatacgctttttttcgtGTTCTGTGGTCTGTGTGTTGCCTGCGTTGCGCGAACCTCCCTCATGGTACAACAAGCTCCGTTGCAAACACTTGCACAACCCCAAAAACTACGCGTTTTGACTATACACCTTTTGCAAGAGCCAAAGCCTGCAGGcacgcgctttcgtgttnnnnnnnnnnnnnngggtgcaggttacatagacgntgcttccttttctgcnnnnnnnnnnnnnnnnnnnnnnnnncctgcagaggtaattttgcagcagtacgctaccgatatgacggacgacgcggatgcccgcgtctgtcagtattacgcgcgtgggttgcgcnnnnnnnnnnnnnnnnnnnnnnnnnnnnnnnnnnnnnnnnnnnnnnnnnnnnnnnnnnnnnnnnnnnnnnnnnnnnnnnnnnnnnnnnnnnnnnnnnnnnnnnnnnnnnnnnnnnnnnnnnnnnnnnnnnnnnnnnnnnnnnnnnnnnnnnnnnnnnnnnnnnnnnnnnnnnnnnnnnnnnnnnnnnnnnnnnnnnnnnnnnnnnnnnnnnnnnnnnnnnnnnnnnnnnnnnnnnnnnnnnnnnnnnnnnnnnnnnnnnnnnnnnnnnnnnnnnnnnnnnnnnnnnnnnnnnnnnnnnnnnnnnnnnnnnnnnnnnnnnnnnnnnnnnnnnnnnnnnnnnnnnnnnnnnnnnnnnnnnnnnnnnnnnnnnnnnnnnnnnnnnnnnnnnnnnnnnnnnnnnnnnnnnnnnnnnnnnnnnnnnnnnnnnnnnnnnnnnnnnnnnnnnnnnnnnnnnnnnnnnnnnnnnnnnnnnnnnnnnnnnnnnnnnnnnnnnnnnnnnnnnnnnnnnnnnnnnnnnnnnnnnnnnnnnnnnnnnnnnnnnnnnnnnnnnnnnnnnnnnnnnnnnnnnnnnnnnnnnnnnnnnnnnnnnnnnnnnnnnnnnnnnnnnnnnnnnnnnnnnnnnnnnnnnnnnnnnnnnnnnnnnnnnnnnnnnnnnnnnnnnnnnnnnnnnnnnnnnnnnnnnnnnnnnnnnnnnnnnnnnnnnnnnnnnnnnnnnnnnnnnnnnnnnnnnnnnnnnnnnnnnnnnnnnnnnnnnnnnnnnnnnnnnnnnnnnnnnnnnnnnnnnnnnnnnnnnnnnnnnnnnnnnnttttttaagttggggtgtgtacttttttggtgaaggactctgtgcggttgtgcgtttttttgcgtgtgcgccgcttgtgtatgtacagagtgcctgcggacatgtgtgtgctgcattattttcttttttactcgGTGGGGGACnnnnnnnnnnnncgcgtcgcgtgcgtgttcacaaggatacatatgtgttgcccgaattataattcggcgcgtgcacttgcacagtttttgacggagcgcggtttgcggatgcataaaaagtgggggcagaattttctgctcgnnnnnnnnnnnnnnnnnnngcnnnnnnnnnnnnnnnnnnnnnngcgtggggaacgtgtatgggaaattggtgcagGCATTGGTGCGATGACCGCACTTTTGGTGCAAAACAGTGATTTTTTAACAGTGTTTGAAATTGATCGCGGCTTTGTGCAGACATTGCGCAAACTTTTTGATGCACACGTCCGTGTGATAGAAGGGGATGTGTTGCAACAGTGGCATGCTGCAGCAGCACAGGAACAACCTGCGTGTGTTCTAGGAAATTTACCCTACAATATTGCTGCCCGTTTTATTGGAAACACGATCGAATCAGGCTATATTTTTAAGCGtatggnggtgaccgttcaaaaagaaatcgggttgagaatgactgcgctccctgcacaaaaatgnnnnnnnnnnnnnnnnnnnnnnnnnnnnnnncagtatgaaGTGCGTGTGATTCGTAACGTTGCGCCTGTCTGTTTTTGGCCGCGTCCTCATGTAGTTTCTCAAGCATTGGTACTCACCAAGCGTAATGCGGTGCCTTCTTGTGTGGATCCTGCGCTTTTTCTGCACGTGACGAAAACTTTGTTTTCTGCGCGGCGTAAAACGGTAAGAAATAATTTACTCACGTGGCAAAAAAGGATGCCAGGCGGTGCAGCTGTGTGTGTAGAAGAACTCTGCGCACGTGCAGGTATTGACGCGCGTGCGCgtgcnnnnnnnnnnnnnnnnnnnnnnnnnnnnnnnnnnnnnnnnnnnnnnnnnnnnnnnnnnnnnnnnnnnnnnnnnnnnnnnnnnnnnnnnnnnnnnnnnnnnnnnnnnnnnnnnnnnnnnnnnnnnnnnnnnnnnnnnnnnnnnnnnnnnnnnnnnnnnnnnnnnnnnnnnnnnnnnnnnnnnnnnnnnnnnnnnnnnnnnnnnnnnnnnnnnnnnnnnnnnnnnnnnnnnnnnnnnnnnnnnnnnnnnnnnnnnnnnnnnnnnnnnnnnnnnnnnnnnnnnnnnnnnnnnnnnnnnnnnnnnnnnnnnnnnnnnnnnnnnnnnnnnnnnnnnnnnnnnnnnnnnnnnnnnnnnnnnnnnnnnnnnnnnnnnnnnnnnnnnnnnnnnnnnnnnnnnnnnnnnnnnnnnnnnnnnnnnnnnnnnnnnnnnnnnnnnnnnnnnnnnnnnnnnnnnnnnnnnnnnnnnnnnnnnnnnnnnnnnnnnnnnnnnnnnnnnnnnnnnnnGTACCAGACCcgatagAACTGGTGCCACTTGATTCTTTTGTTGAGGGGACTGACGATAGCACGTGAAGGTGCACAGGGTGTCGTATTCTTGCAGTGCAGAAAGCACGTCGGTGTGCAGTGTTCATTTTTCCGTTTTTAGAATACCGGGCGCgacgTGTCGGTGTGATgtgttctgcgcnnnnnnngtttttttttgtgcacgatctcagctcaagaggatcgtgcgtgCATTGTGTGTGAATGGGCATACGGCAAAGTTTTCAAGACCTCTTCACGTGCGCGATCGGGTGTCTTTTGAGTGGGTACGCTCAGTGCCCCCGGCGCTCATTCCTGAGAATATATCGCTTTCTATTCTGTTTGAAAACGAAGACATTATTGCggtgaacaaagcgcagggcatgatagtACATCCTGGGGCAGGCCACTGGACGGGAACACTTGTTCAGGCGCTCAGTTTCTACCGGGTGTATCGTGCACGTTTTGAGGATGAGTTTTCTCGTCAATTTCAGAAAGGATTTCCCGATTTTTTCAGTACCCTGCGTCAGGGTATTGTGCACCGTTTGGATAAAGATACATCGGGCGTACTCCTCACTTCGCGCAACATGCATGCTCATGAGGCACTTGTACGTTCGTTTAAAAAAAGACAAGTAAGAAAAGTATATCTTGCGTTATTGCAGGGTGTTCCTGCACGCGGGGTTGgggtgattgaaacaacaatcgtgcgagatagaagacgacgcacgcggtttgttgcgtctgaagatttttcaaaaggaaagtacgcacgtacgcgatacaaggtgatgannnnnnnnnnnnnnnnnnnnnnnnnnnnnnnnnnnnnnnnnnnnnnnnnnnnnnnnnnnnnnnnnnnnnnnnnnnnnnnnnnnnnnnnnnnnnnnnnnnnnnnnnnnnnnnnnnnnnnnnnnnnnnnnnnnnnnnnnnnnnnnnnnnnnnnnnnnnnnnnnnnnnnnnnnnnnnnnnnnnnnnnnnnnnnnnnnnnnnnnnnnnnnnnnnnnnnnnccataccgcttcgttttgttcgactgatacaccgattatcggttaggtagggtgtggcaggtgcgtgcgtatatgcgttttacnnnnnnnnnnnnnnnnnnnnnnnnnnnnnnnnnnnnnnnnnnnnnnnnnnnnnnnnnnnnnnnnnnnnnnnnnnnnnnnnnnnnnnnnnnnnnnnnnnnnnnnnnnnnnnnnnnnnnnnnnnnnnnnnnnnnnnnnnnnnnnnnnnnnnnnnnnnnnnnnnnnnnnnnnnnnnnnnnnnnnnnnnnnnnnnnnnnnnnnnnnnnnnnnnnnnnnnnAAAAAGAGGGGGGAACTGGGACACTGCTTTGTCCGCCGCGCACGCAAAGAGTAAAATGTATTGTGCAGAAGGTAGTAAAGAAGAGaacgtacgacatgcgcaccgtatactctgcgtagtgtgcgcaccgtcaatcacnnnnnnnnnnnnnnnnnnnnnnnnnnnnnnnnnnnnnnnnnnnnnnnnnnnnnnnnnnnnnnnnnnnnnnnnnnnnnnnnnnnnnnnnnnnnnnnnnnnnnnnnnnnnnnnnnnnnnnnnnnnnnnnnnnnnnnnnnnnnnnnnnnnnnnnnnnnnnnnnnnnnnnnnnnnnnnnnnnnnnnnnnnnnnnnnnnnnnnnnnnnnnnnnnnnnnnnnnnnnnnnnnnnnnnnnnnnnnnnnnnnnnnnnnnnnnnnnnnnnnnnnnnnnnnnnnnnnnnnnnnnnnnnnnnnnnnnnnnnnnnnnnnnnnnnnnnnnnnnnnnnnnnnnnnnnnnnnnnnnnnnnnnnnnnnnnnnnnnnnnngcgtatagggtttgannnnnnnnnnnnnnnnnnnnnnnnnnnnnnnnnnnnnnnnnnnnnnnnnnnnnnnnnnnnnnnnnnnnnnnnnnnnnnnnnnnnnnnnnnnnnnnnnnnnnnnnnnnnnnnnnnnnnnnnnnnnnnnnnnnnnnnnnnnnnnnnnnnnnnnnnnnnnnnnnnnnnnnnnnnnnnnnnnnnnnnnnnnnnnnnnnnnnnnnnnnnnnnnnnnnnnnnnnnnnnnnnnnnnnnnnnnnnnnnnnnnnnnnnccgtgtggtatacgtgtgtgcacgcgcttgcatactcggcaggtGAAAAAAAcacacccgcgcgTGTTATTCTCTCTCTCGGATCCATAACGTGAGGAGAAGCGTATAGCCCGGTGTTGAATCCAATTTCATTGAGTATCGCTGCAAGCATacgtgcgctgganccttttcccttcgtgcccgcaacatggatgCtctgatatgcgttgtgtggannnnnnnnnnnnnnnnnnnnnnnnnnnnnnnngtGCAGAGGTGGTGTGCCGCTTGGGGGCATTTTCTCAAGCGTGCGAATGCGCTCAACCCAGGCGTAAAAATCTTGAAAAGAATGCACCGGTATATGTGaaactccgtgtgcgctcggtcgcaCTATAATATGCGGTACGGAAGAGGCAGCAATccttgccgggaaagagaactgatgtacattgctaaggnnnnnnnnnnnnnnnnnnnnnnnnnnnnnnnnnnggggactctaccaaaagatgtgtcaggtatcaagattcacatgattggtatcaagggcactggcatgtctgcgcttgcagagctacTGTGTGCACGGGGTGCCCGTGTGTCAGGTAGTGATGTTGCAGATGtgttttacacggataggattnnnnnnnnnnnnnnnnnnnnnnnnnnnnnnnnnnTTTCTTGCCAGAACCTTGCTGACGCTCCCGATGTGGTTATCCACTCTGCAGCctatgtgcctgaagaaaacgacgagttggcagaggcgtaccggcggggtattcctacccttacctacccagaagcgctGGGGGACATttcctgtgcgcggttttcgtgtggtattgcaggtgttcatggaaagacgaccacgaccgcgatgattgctcaaatggtaaaggagctgcgccttgatgcgtccgtccttgtggggnnnnnnnnnnnnnnnnnnnnnnnnnnnnnnnnnnnnnnnnnnnnnnnnnnnnnnnnnnnnnnnnnnnnnnnnnnnnnnnnnnnnnnnnnnnnnnnnnnnnnnnnnnnnnnnnnnnnnnnnnnnnnnnnnnnnnnnnnnnnnnnnnnnnnnnnnnnnnnnnnnnnnnnnnnnnnnnnnnnnnnnnnnnnnnnnnnnnnnnnnnnnnnnnnnnnnnnnnnnnnnnnnnnnnnnnnnnnnnnnnnnnnnnnnnnnnnnnnnnnnnnnnnnnnnnnnnnnnnnnnnnnnnnnnnnnnnnnnnnnnnnnnnnnnnnnnnnnnnnnnnnnnnnnnnnnnnnnnnnnnnnnnnnnnnnnnnnnnnnnnnnnnnnnnnnnnnnnnnnnnnnnnnnnnnnnnnnnnnnnnnnnnnnnnnnnnnnnnnnnnnnnnnnnnnnnnnnnnnnnnnnnnnnnnnnnnnnnnnnnnnnnnnnnnnnnnnnnnnnnnnnnnnnnnnnnnnnnnnnnnnnnnnnnnnnnnnnnnnnnnnnnnnnnnnnnnnnnnnnnnnnnnnnnnnnnnnnnnnnnnnnnnnnnnnnnnnnnnnnnnnnnnnnnnnnnnnnnnnnnnnnnnnnnnnnnnnnnnnnnnnnnnnnnnnnnnnnnnnnnnnnnnnnnnnnnnnnnnnnnnnnnnnnnnnnnnnnnnnnnnnnnnnnnnnnnnnnnnnnnnnnnnnnnnnnnnnnnnnnnnnnnnnnnnnnnnnnnnnnnnnnnnnnnnnnnnnnnnnnnnnnnnnnnnnnnnnnnnnnnnnnnnnnnnnnnnnnnnnnnnnnnnnnnnnnnnngtgaacatctttttgaattagctaaacggaagcaccggcgggtgtattattacgaggctgtcatgcaggcnnnnnnnnnnnnnnnnnnnnnnnnnnnnnnnnnnnnnnnnnnnnnnnnnnnnnnnnnnnnnnnnnnnnGCAAATTGGGTGAggtgttgttcaattattttaaagaggaggtgtAAAGTTCGGTTGCGGTTTCGCCAACATGGTGGTGGTGCCGGCTGTGGATCTGGTGGATATAGGGTGAAGTGAGACAGGCTGCGAATGGATGGTGATGCAAAGAGCGGAGTGCGGAGGGGTGCGTGAGTAATCGGTGCGATGTGTCTGGAAAtaaggcggtacgcatagcaGTTTCAGGCGCGTCAGGGTGTGGTAATACCACCGTGTCTGCATTGCTTGCGGAAAGACTGGGACTTCCCCTAGTGAATTATACGTTTAGGAATATTGCCCGGGAGTTGGGTATCTCTCTTAGTGaggtgctcgagcgtgcgcggacggatannnnnnnnnnnnnnnnnnnnnnnnnnnnnnnnnnnnnnnnnnnnnnnnnnnnnnnnnnnnnnnnnnnnnnnnnnnnnnnnnnnnnnnnnnnnnnnnnnnnnnnnnnnnnnnnnnnnnnnnnnnnnnnnnnnnnnnnnnnnnnnnnnnnnnnnnnnnnnnnnnnnnnnnnnnnnnnnnnnnnnnnnnnnnnnnnnnnnnnnnnnnnnnnnnnnnnnnnnnnnnnnnnnnnnnnnnnnnnnnnnnnnnnnnnnnnnnnnnnnnnnnnnnnnnnnnnnnnnnnnnnnnnnnnnnnnnnnnnnnnnnnnnnnnnnnnnnnnnnnnnnnnnnnnnnnnnnnnnnnnnnnnnnnnnnnnnnnnnnnnnnnnnnnnnnnnnnnnnnnnnnnnnnnnnnnnnnnnnnnnnnnnnnnnnnnnnnnnnnnnnnnnnnnnnnnnnnnnnnnnnnnnnnnnnnnnnnnnnnnnnnnnnnnnnnnnnnnnnnnnnnnnnnnnnnnnnnnnnnnnnnnnnnnnnnnnnnnnnnnnnnnnnnnnnatgcagaatggcttcatcgttttccttctttggagtctcttgcgtgcgctccatttgcgcacgtgctcnnnnnnnnnnnnnnnnnnnnnnnnnnnnnnnnnnnnnnnnnnnnnnntcagtcggcaaaactcattgttgaaaggtattgtgcnnnnnnnnnnnnnnnnnnnnnnnnnnnnnnnnnnnnnnnnnnnnnnnnnnnnnnnnnnnnnnnnnnnnnnnnnnnnnnnnnnnnnnnnnnnnnnnnnnnnnnnnnnnnnnnnnncnnnnnnnnnnnnnnnnnnnnnnnnnnnnnnnnnnatacgcaccaggtcagtgatcgggagttgctctcgctggtccggtGCACCCTGTATGAGGAAAATCCTCGGCGTTGGTACTACGCACTGATGGATTATGGGGCAGTTCTAAAAAGGaagattacaaatcctaatcgtcgcagcaagcnttannnnnnnnnnnnnnnnnnnnnnnnnnnnnnnnnnnnnnnnnnnnnnnnnnnnnnnnnnnnnnnnnnnnnnnnnnnnnnnnnnnnnnnnnnnnnnnnnnnnnnnnnnnnnnnnnnnnnnnnnnnnnnnnnnnnnnnnnnnnnnnnnnnnnnnnnnnnnnnnnnnnnnnnnnnnnnnnnnnnnnnnnnnnnnnnnnnnnnnnnnnnnnnnnnnnTTATGACTCAAGACGCTTGAGTATTTCACAAATAAAGATGCCTTTCTCTTTTATTTCAATGGCATCTGATGTGAGGATCAGCATGTTGGGCTGCTTTGCGTCAAGGCGAACGCGATCGTTGGAGGTctgtaccaggcgcagtatctttttgnnnnnnnnnnnnnnnnnnnnnnnnnnnnnnnnnnnnnnnnnnnnnnnnnnnnnnnnnnnnnnnnnnnnnnnnnnnnnnnnnnnnnnnnnnnnnnnnnnnnnnnnnnnnnnnnnnnnnnnnnnnnnnnnnnnnnnnnnnnnnnnnnnnnnnnnnnnnnnnnnnnnnnnnnnnnnnnnnnnnnnnnnnnnnnnnnnnnnnnnnnnnnnnnnnnnnnnnnnnnnnnnnnnnnnnnnnnnnnnnnnGTACATATGGGGGATGAACCCTCGGTAATTAAGATCGATGACGGTTTCTATCCTTTGCTCGTTTGGAGCATGTTGGAGGCGTTCTATTGCCTCTTCTAACAGCTGTACATACAGGTnnnnnnnnnnnnnnnnnnnnnnnnnnnnnnnnnnnnnnnnnnnnnnnnnnnnnnnnnnnnnnnnnnnnnnnnnnnnnnnnnnnnnnnnnnnnnnnnnnnnnnnnnnnnnnnnnnnnnnnnnnacctgtaaacgttttattgcaaggtctgaaagtgccacgtcgtgatagtacagcagatacgnnnnnnnnnnnnnnnnnnnnnnnnnnnnnnnnnnnnnnnnnnnnnnnnnnnnnnnnnnnnnnnnnnnnnnnnnnnnnnnnnnnnnnnnnnnnnnnnnnnnnnnnnnnnnnnnnnnnnnnnnnnnnnnnnnnnnnnnnnnnnnnnnnnnnnnnnnnnnnnnnnnnnnnnnnnnnnnnnnnnnnnnnnnnnnnnnnnnnnnnnnnnnnnngtgggcaacgcatatggatagctcaggcacgagtttttggagcatacactttacggattctaagttttcgattctgttatgtnnnnnnnnnnnnnnnnnnnnnnnnnnnnnnnnnnnnnnnnnnnnnnnnnnnnnnnnnnnnnnnnnnnnnnnnnnnnnnnnnnnnnnnnnnnnnnnnnnnnnnnnnnnnnnnnnnntgagcaagctcatgtctctgattttgagcatacccatgtgaagcgttcggggaatgggcgttgcactgagggagagacaatctacattagttttcatctgctttaatttttctttatcctgcacaccgaaacgttgttcctcatcgnnnnnnnnnnnnnnnnnnnnnnnnnnnnnnnnnnnnnnnnnnnnnnnnnnnnnnnnnnnnnnnnnnnnnnnnnnnnnnnnnnnnnnnnnnnnnnnnnnnnnnnnnnnnnnnnnnnnnnnnnnnnnnnnnnnnnnnnnnnnnnnnnnnnnnnnnnnnnnnnnnnnnnnnnnnnnnnnnnnnnnnnnnnnnnnnnnnnnnnnnnnnnnnnnnnnnnnnnnnnnnnnnnnnnnnnnnnnnnnnnnnnnnnnnnnnnnnnnnnnnnnnnnnnnnnnnnnnnnnnnnnnnnnnnnnnnnnnnnnnnnnnnnnnnnnnnnnnnnnnnnnnnnnnnnnnnnnnnnnnnnnnnnnnnnnnnnnnnnnnnnnnnnnnnngatcgtctgtttcttcgtaggggaatgctgcttcaaacgcatactgccattcgTCATCTTTTGGGAAGGCGTGGCCGCGCGTAGTTTTTCGCAGAGAGTAGAGTTCCACTAGTTtttgcgcgatgttttcaacagattttttgacacgtgcttttctcgtttcccatgactttgacccaaggctatctaagtgaggtttgttccnnnnnnnnnnnnnGTAACGTTGCACCAGATGTGCCTGCTCAATAGGGAtaaggatcgtttcttcctgtgcatagaggaggtttacgtaatcaCGTTCTGACTGTGCTGTTTTTATGCGCTCTATTCCCTTAAATAAACCGATGCCGTACTGCGCATGCACCACGTAATCCCCGGGATTTAATTCCACAAATGTGTCGATAGGCGTGCTCCGTGCGCGTTGCACTGATTGAGGAGTTTTTCTGCGGCGACCGAAGATTTCGCCTTCTTGAACGATCAGTATTTTGAGAGCAGGAATGCTAAATCCTGCAGAAAGCGCGCAAGGTAGCACAGTGACGTCGCAACCTTTGACTAGTGCTCTGATGCGCAGTGCCTGCTGCTCACTTTCTGCAAAGACGAAAACgnnnnnnnnnnnnnnnnnnnnnnnnnnnnnnnnnnnnnnnnnnnnnnnnnnnnnnnnnnnnnnnnnnnnnnCAGGATCGCTTGCCAAGCATATACTTTCGCACGCTGGCAGCTGTGGAAAAAAGTGAGTGAAATACACCGTGTGCAGGTGGnGCGCGCAGACAGCGGAAAAATCGAGCACTATGTGTTCTGGTTGAGGATACCAGCGCGCAGGTACGTGTTCGTGCGCGAGTTGCATTTTATGGTAGAGGTTCCGACACTCGTCTTGGAGCGCGCGTGCACCGTTGTGCTGGCGTTCGTAGTCAAGATAAAAGACGCTTGGGGGTGAAGGGnnnnnnnnnnnnnnnnnnnnnnnnnnnnnnnnnnnnnnnnnnnnnnnnnnnnnnnnnntttcctccccttcatncgtttttctgtgggtgagtncttcgannnnnnnnnnnnnnnnnnnnnnnnnnnnnnnnnnnnnnnnnnnnnnnnnnnnnnnnnnnnnnnnnnnnnnnnnnnnnnnnnnnnnnnnnnnnnnnnnnnnnnnnnnnnnnnnnnnnnnnnnnnnnnnnnnnnnnnnnnnnnnnnnnnnnnnnnnnnnnnnnnnnnnnnnnnnnnnnnnnnnnnnnnnnnnnnnnnnnnnnnnnnnnnnnnnnnnnnnnnnnnnnnnnnnnnnnnnnnnnnnnnnnnnnnnnnnnnnnnnnnnnnnnnnnnnnnnnnnnnnnnnnnnnnnnnnnnnnnnnnnnnnnnnnnnnnnnnnnnnnnnGAGCGTGTGGATCTCgatgtgctctcccacacggaaggagcgtttgagggtacgcacataatcgnnnnnnnnnnnnnnnnnnnnnnnnnnnnnnnnnnnnnnnnnnnnnnnnnnnnnnnnnnnnnnnnnnnnnnnnnnnnnnnnnnnnnnnnnnnnnnnnnnnnnnnnnnnnnnnnnnnnnnnnnnnnnnnnnnnnnnnnnnnnnnnnnnnnnnnnnnnnnnnnnnnnnnnnnnnnnnnnnnnnnnnnnnnnnnnnnnnnnnnnnnnnnnnnnnnnnnnnnnnnnnnnnnnnnnnnnnnGCACTATGTGTGCGCAACACGTGCGCACGTATTCGCCAAAAAAGTAGGACCGCAGTCCACCGGTGCACCCTTCAACGCGGTAGGGAAAGCGTGCGGCACGCAGCGAGCGCAGCGCATGCTGGAGCTCATTCCAAGAATGGAGTATGCCACGCACAGGCTCCTGGGtacgagcgtgcatttcttgagcgcgtgttgacacacggccgagtgtaacnnnnnnnnnnnnnnnnnnnnagtccctgagcgagaaggatgcagcaggagagggcaagggagganaacaggAAGGAGCACCGGTAGTAGTTGATGGGGTTAATTTTTTTCCGTTATGATCGCTGCTATATGTTGTCGGTGTTGTCGTATCTTCACGTGTACTTTGGGCCGTTTCGTCTGTTGCAATCTTATGCGGTGTTGATGGGGATTGCCCTGTATGCGGGnnnnnnnnnnnnGTATGGGGTGTTGCCCAGTGCGTATCGCTTTTTGCCCCAAGACAGGGGGCGTGCGTTTGCGCCGTGTGCACAGGAAGCAGCGGGTAAACCCACAGGGGCAGGAGTGATTTTTGTGTCCGTCTTTGTGTTGTTAGTGTACCTGCTTATGCGTCCGAGTTTTGTGcatgcgcttatattgctgctgacgtggggggtgatgctcaccggntacttannnnnnnnnnnnnnggtgtgctggggnnnnnnnnnnnnnnnnnnnnnnnnnnnnnnnnnnnnnnnnnnnnnnnnnnnnnnnnnnnnnnnnnnnnnnnnnnnnnnnnnnnnnnnnnnnnnnnnnnnnnnnnnnnnnnnnnnnnnnnnnnnnnnnnnnnnnnnnnnnnnnnnnnnnnnnnnnnnnnnnnnnnnnnnnnnnnnnnnnnnnnnnnnnnnnnnnnnnnnnnnnnnnnnnnnnnnnnnnnnnnnnnngttgatggcgcttctttcgatgggtacgattttttactttttgttgggaaatgtgcgtgcggcgcagtacctactggtgccgtttgtagnggnnnnnnnnnnnnnnnnnnnnnnnnnnnnnnnnnnnnnnnnnnnnnnnnnnnnnnnnnnnnnnnnnnnnnnnnnnnnnnnnnnnnnnnnnnnnnnnnnnnnnnnnnnnnnnnnnnnnnnnnnnnnnnnnnnnnnnnnnnnnnnnnnnnnnnnnnnnnnnnnnnnnnnnnnnnnnnnnnnnnnnnnnnnnnnnnnnnnnnnnnnnnnnnnnnnnnnnnnnnnnnnnnnnnnnnnnnnnnnnnntgtgcggatcctgagccgggtgcgctttccgctccatgatcacatgcgtgagaattggcactggtctacggcgcaggtattgctgaggtttatgattttacagggactgctnnnnnnnnnnnnnnnnnnnnnnnnnnnnnnnnnGCGGTAGaGGGAGGGCACCCCTTGCGGGGCACGCCGGGCCGAGCGAGGGCGACGGTGCGGAGTATCCGGCGCCTTGACGTGCGTTTATTTCTTTTGctagcctgcccctaattgctttccgtttccggaatgatggtagaGGAGACAGGGCGGAAGGCGTGGGGTGTGTATGGTGCCGGTGAGAAGGTTCATGGCAGTGTGTGCGGTGGCGGCGTGTGCCGGGCCGTGTTTTTGCGttcaagcgtttatctcttctcggatcgggtatgggcactttgggatatatgggaannnnnnnnnnnnntcctactacaaacatgttccgatgacgggactaggggttgacgtggtaacgtcttcaggcgttgcgatggtgttcaatgtggagactgcgttgacgcagctcatgnnnnnnnnnnnnnnnnnnnnnnnnnnnnnnnnnnnnnnnnnnnnnnnnnnnnnnnnnnnnnnnnnnnnnnnnnnnnnnnnnnnnnnnnnnnnnnnnnnnnnnnnnnnnnnnnnnnnnnnnnnnnnnnnnnnnnnnnnnnnnnnnnnnnnnnnnnnnnnnnnnnnnnnnnnnnnnnnnnnnnnnnnnnnnnnnnnnnnnnnnnnnnnnnnnnnnnnnnnnnnnnnnnnnnnnnnnnnngcgaagattctcatcacgcatacagtgacgcatggactggctaatcgctggatatcaggtccccgctggtggaatnnnnnnnnnnnnnnnnnnnnnnnnnnnnnnnnnnnnnnnnnnnnnnnnnnnnnnnnnnnnnnnnnnnnnnnnnnnnnnnnnnnnnnnnnnnnnnnnnnnnnnnnnnnnnnnnnnnnnnnnnnnnnnnnnnnnnnnnnnnnnnnnnnnnnnnnnnnnnnnnnnnnnnnnnnnnnnnnnnnnnnnnnnnnnnnnnnnnnnnnnnnnnnnnnnnnnnnnnnnnnnnnnnnnnnnnnnnnnnnnnnnnnnnnnnnnnnnnnnnnnnnnnnnnnnnnnnnnnnnnnnnnnnnnnnnnnnnnnnnnnnnnnnnnnnnnnnnnnnnnnnnnnnnnnnnnnnnnnnnnnnnnnnnnnnnnnnnnnnnnnnnnnnnnnnnnnnnnnnnnnnnnnnnnnnnnnnnnnnnnnnnnnnnnnnnnnnnnnnnnnnnnnnnnnnnnnnnnnnnnnnnnnnnnnnnnnnnnnnnnnnnnnnnnnnnnnnnnnnnnnnnnnnnnnnnnnnnnnnnnnnnnnnnnnnnnnnnnnnnnnnnnnnnnnnnnnnnnnnnnnnnnnnnnnnnnnnnnnnnnnnnnnnnnnnnnnnnnnnnnnnnnnnnnnnnnnnnnnnnnnnnnnnnnnnnnnnnnnnnnnnnnnnnnnnnnnnnnnnnnnnnnnnnnnnnnnnnnnnnnnnnnnnnnnnnnnnnnnnnnnnnnnnnnnnnnnnnnnnnnnnnnnnnnnnnnnnnnnnnnnnnnnnnnnnnnnnnnnnnnnnnnnnnnnnnnnnnnnnnnnnnnnnnnnnnnnnnnnnnggtttattaccttcgctctgaccgttgttgccgagggattcagttggattgtgtnnnnnnnnnnnnnnnnnnnnnnnnnnnnnnnnnnnnnnnnnnnnnnnnnnnnnnnnnnnnnnnnnnnnnnnnnnnnnnnnnnnnnnnnnnnnnnnnnnnnnnnnnnnnnnnnnnnnnnnnnnnnnnnnnnnnnnnnnnnnnnnnnnnnnnnnnnnnnnnnnnnnnnnnnnnnnnnnnnnnnnnnnnnnnnnnnnnnnnnnnnnnnnngtcgtgcgccgcgcagcttgaacgagctcagccggtgcatgagcgaaagcgcgtggacgactgtctgcccgaactttcccggcaggtgcggcggcacgcgcagtatccactttgaaagGTATACCATCCCCGAGGCCnnnnnggcctggcgcgctcctgccgAAAGCGCACCGCTCGTCAGGGGGACCCCAAGCACCGTTGCCCACACGCGTCCAACCGGCGGTCTAAGGTAGCAGGCGAGCGTTCCCACCCACAGCCCTGCCAAGGTCatccagcggatgcgtccccccgttnnnnnnnnnnnnngcagtgcnnnnnnnnnnnnnnnnnnnnnnnnnnnnnnnnnnnnnnnnnnnnnnnnnnnnnnnnnnnnnnnnnnnnnnnnnnnnnnnngtacataacgcagcacacgcggcctaagcccgtgttccgaacacggcgccgtgcattcacacccnnnnnnnnnnnnnnnnnnnnnnnnnnnnnnnnnnnnnnnnnnnnnnnnnnnnnnnnnnnnnnnnnnnnnnnnnnnnnnccactgggnnnnnnnnnnnnnnnnnncggttagcgctccgaGTAAGGTACTGCTCACTGCCCCCAGCGCGCAAAATAGCGGGAGTACGTACCACACCCCCGCGCCAAAAACGAGCACCCGAGCAAGCGTAAGCTGTATCACGTTTGAACAGAACGCACCCATCACGCTTATCCCCACGCACGAAAGGTACCGGCACGGGACGAACCGCAGCGCATACATGAGCGCACCCGAAGCGGTGCTCCCTGCAAGCGAGAGGACAAATACATAAGAAAAGAGCGTCCCACTCACCAGAGCCTGCCCTATTACCTTCAGGAATACTAAACGCGCGTACGCACAGAAAGGGAGCAGATCCGGCGAGATCAACAGCGGCAAATTCGCAAGCCCCACGCGAAAGAAAGGCAGCGGCTTTGGAATGACGTGTTCAACCGTAGAGAGAAAGAAACACATGCCGCCTAAAAGCGACACTAACTCatcgcgtacgtctagtggcnnnnnnnnnnnnnnnnnnnnnnnnnnnnnnnnnnnnnCCCACAGCCACCGCCGCTGGTGCTTTCCCGAAACAGAAGTGCAGCTAAGTCATCGTCCGTCGCCTCTCGCACAGAGCGCACAGCCACCTCAAAGTGGAGCGTCTTCCCCGCGAGGGGATGATTTCCATCTACAATAATCGTTTCACCTTGCACGTCAGTGACGGTCACCGGTCGACTGTCACCCCCGCTTCCTGCATCAAACCGCATGCCCACCTCTATTGGCACGTTTGGAGGAAACTGATCTCGCCCCACTGTCATGCGCAAGTCCTCCTGCACCTCTCcatacgctcctaccggaggaatGGTTACTGAAAACTCCTCCCCCTCTTCTCGGTTAATTAAGGCGGTCTCGAGGCCAGGAATGATCATGCCGTGCCCCTGAACATACTCGAGCGCACCCATCACGTCGGAAGAATCGATGATCTCCCCCGTGTCATCTCGCAGGGTGTACTCGATGTTCACCACACACTCATTTGCGATTTTCATGCGCGGCATGctagcacaggcaagatgctcacggcaagggcagtttctgtgccgtgtgcccttgacagaatnnnnnnnnnnnnnnnnnnnnnnnnnnnnnnnnnnnnnnnnnnnnnnnnnnnnnnnnnnnnnnnnnnnnnnnnnnnnnnnnnnnnnnnnnnnnnnnnnnnnnnnnnnnnnnnnnnnnnnnnnnnnnnnnnnnnnnccccattatggtagaggtctacgcgcggttgcgcgCTGCTGTTGCTCGGTTGGCGGTCTGTAGCGCTGCGGAGAAGGACGGTGCCCTGCGCGCTGTGCGCGATGCGCTACATGCGCAGCGGGAGGATATCCTGCGTGCAAATGCGCAGGATcttgcgcgggcgcgtgaggcgggtcttgccgcaccnnnnnnnnnnnnnnnnnnnnnnnnnnnnnnnnnnnnnnnnnnnnnGTTGCGGTCTTTGACtgttctttcgcttcagcgggatcctatcggGGAAATTATAGAAGGGTACACTCTTGCGAATGGACTGGAAATCCGGAAGGTACGTGTTCCTCTGGGGGTGGTGGCTGTCATCTACGAGTCTCGGCCCAACGTGACCGTAGATGCGTTTGCACTTGCGTACAAAAGCGGCAATGCGGTGCTCctgcgcgcaggttctgcagcGAGTTATTCAAATGCCGCGCTTTTGCGCGCAATTCACGTGGGTTTGAAGAAAGCGCATGGTGTCGTGGACGCGGTGGCTGTTCCTCCCGTTTTGnnnnnnnnnnnnnnnnnnnnnnnnnnnnnnnnnnnnnnnnnnnnnnnnnnnnnnnnnnnnnnnnnnnnnnnnnnnnnnnnnnnnnnnnnnnnnnnnnnnnnnnnnnnnnnnnnnnnnnnnnnnnnnnnnnnnnnnnnnnnnnnnnnnnnnnnnnnnnnnnnnnnnnnnnnnnnnnnnnnnnnnnnnnnnnnnnnnnnnnnnnnnnnnntgcaannnnnnnnnnnnnnnnnnnnnnnnnnnnnnnnnnnGGTGCATTGTGCGGTTGCGCGTCCTTTTTTGCACCGTGTACAGGAGATTTTTGCCACCTGTGAGGAGACTACGCGCAAGCCCGGTGGTGTGGATTTTTTTTGTGATGCTGAGTCTTTCTCCCTTCTCACAGAAAGGGGCGCGAGAAAAAATGTTTTTCATGCACAGGCAGAGACCTGGGATCGGGAATACCTGGACTATCAGGTATCCGTGCGGGTGGTGCCAAACCTTGAAGAAGCACTCAGGCACATTGCTCGTCATTCTACGAAACACTCAGAGGTTATTGTCACGCGCGATCGTGCCCGTGCGCGTCGTTTTCATCAGGAAGTAGATGCTGCCTGTGTATATGTCAATGCTTCAAGTAGGTTTACCGATGGAGGGCAGTTTGGCATGGGAGCAGAGATTGGGGTCAGTACGCAAAAATTGCACGCGCGCGGTCCGATGGGTTTGTGTGCACTGACTACTTCAAAATATCTGATTGATGGAGAGGGGCAGGTGCGTCCGTGATCCGTGCGCTTTTTGCTGCGGCAAAAAAAATTGTGATAAAGATTGGGTCAAATACGCTTGCGCAGGCAGATGGTACTCCnnnnnnnnnnnnnnnnnnnnnnnnngctcgcgccnnnnnnnnnnnnnnnnnnnnnnnnnngcagatagttgtggtgtcgtctggcgctcaggttgcagggatttctgcgctccattgcctTTCATCTCCTCCTCAGGGGGCGGGTTTAGAGCGTCACGAATCGCGCGGCGTTATTCcgggtgatggtgcgtcctgcaaacaggcgttgtgtnnnnnnngtcaggcggagttgataagtcgttggcgttctgcgtttgcagcgcaccagcagnnnnnnnnnnnnnnnnnnnnnnnnnnnnnnnnnnnnnnnnnnnnnnnnnnnnnnnnnnnnnnnnnnnnnnnnnnnnnnnnnnnnnnnnnGCGCAGGGTagtacctatccttaatgaaaatgacgcgctctgttgcagcgacgtcccctctgtacccgccgaccggcgggtgtccctatcacnnnnnnnnnggattggagataatgacagtctgtccgcgtttgtagcgctgttgtggcnnnnnnnnnnnnnnnnnnnnnnnnnnnnnnnnnnnnnnnnnnnnnnnnnnnnnnnnnnnnnnnnnnnnnnnnnnnnnnnnnnnnnnnnnnnnnnnnnnnnnnnnnnnnnnnnnnnnnnnnnnnnnnnnnnnnnnnnnnnnnnnnnnnnnnnnnnnnnnnnnnnnnnnnnnnnnnnnnnnnnnnnnnnnnnnnnnnnnnnnnnnnnnnnnnnnnnnnnnnnnnnnnnnnnnnnnnnnnnnnnnnnnnnnnnnnnnnnnnnnnnnnnnnnnnnnnnnnnnnnnnnnnnnnnnnnnnnnnnnnnnnnnnnnnnnnnnnnnnnnnnnnnnnnnnnnnnnnnnnnnnnnnnnnnnnnnnnnnnnnnnnnnnnnnnnnnnnnnnnnnnnnnnnnnnnnnnnnnnnnnnnnnnnnnnnnnnnnnnnnnnnnnnnnnnnnnnnnnnnnnnnnnnnnnnnnnnnnnnnnnnnnnnnnnnnnnnnnnnnnnnnnnnnnnnnnnnnnnnnnnnnnnnnnnnnnnnnnnnnnnnnnnnnnnnnnnnnnnnnnnnnnnnnnnnnnnnnnnnnnnnnnnnnnnnnnnnnnnnnnnnnnnnnnnnnnnnnnnnnnnnnnnnnnnnnnnnnnnnnnnnnnnnnnnnnnnnnnnnnnnnnnnnnnnnnnnnnnnnnnnnnnnnnnnnnnnnnnnnnnnnnnnnnnnnnnnnnnnnnnnnnnnnnnnnnnnnnnnnnnnnnnnnnnnnnnnnnnnnnnnnnnnnnnnnnnnnnnnnnnnnnnnnnnnnnnnnnnnnnnnnnnnnnnnnnnnnnnnnnnnnnnnnnnnnnnnnnnnnnnnnnnnnnnnnnnnnnnnnnnnnnnnnnnnnnnnnnnnnnnnnnnnnnnnnnnnnnnnnnnnnnnnnnnnnnnnnnnnnnnnnnnnnnnnnnnnnnnnnnnnnnnnnnnnnnnnnnnnnnnnnnnnnnnnnnnnnnnnnnnnnnnnnnnnnnnnnnnnnnnnnngtgcgttgatgaccgcgcacagttccatgcgattgtttgtgctcgggtaggcgcnnnnnnnnnnnnnnnnnnnnnnnnnnnnnnnnnnnnnnnnnnnnnnnnnnnnnnnnnnnnnnnnnnnnnnnnnnnnnnnnnnnnnnnnnnnnnnnnnnnnnnnnnnnnnnnnnnnnnnnnnnnnnnnnnnnnnnnnnnnnnnnnnnnnnnnnnnnnnnnnnnnnnnnnnnnnnnnnnnnnnnnnnnnnnnnnnnnnnnnnnnnnnnnnnnnnnnnnnnnnnnnnnnnnnnnnnnnnnnnnnnnnnnnnnnnnnnnnnnnnnnnnnnnnnnnnnnnnnnnnnnnnnnnnnnnnnnnnnnnnnnnnnnnnnnnnnnnnnnnnnnnnnnnnnnnnggcggnnnnnnnnnnnnnnnnnnnnnnnnnnnnnnggtgtacatgctgcactgtttttagaggaaagctaacacggagagggcacagatgaatattctgcataactttgttgtattcgaaggtattgatggcacaggcacgagtacacagtnnnnnnnnnnnnnnnnnnnnnnnnnnnnnnnnnnnnnnnnnnnnnnnnnnnnnnnnnnnnnnnnnnnnnnnnnnnnnnnnnnnnnnnnnnnnnnnnnnnnnnnnnnnnnnnnnnnnnnctctaaggcattgggattgctctttgccgcagatagacacgagcacttggaaggtgcaggaggcattaacgattgtcttgcagaaggaaagatagtgctctgcgatcggtatgttttttccagtttggtgtaccAAggcatggcggtgtcgnnnnnnnnnnnnnnnnnnnnnnnnnnnnnnnnnnnnnnnnnnnnnnnnnnnnnnnnnnnnnnnnnnnnnnnnnnnnnnnnnnnnnnnnnnnnnnnnnnnnnnnnnnnnnnnnnnnnnnnnnnnnnnnnnnnnnnnnnnnnnnnnnnnnnnnnnnnnnnnnnnnnnnnnnnnnnnnnnnnnnnnnnnnnnnnnnnnnnnnnnnnnnnnnnnnnnnnnnnnnnnnnnnnnnnnnnnnnnnnnnnnnnnnnnnnnnnnnttcttcaccatctgcgcgaatacaggcgtctaaaatagtgtgtggacgTAGATACACTATCTGAGGAGCAGTGGAGAGTATATATCAGGAACGTgctttgcaagcggaaggcgcgtgctcggtaaaacggtgctgcaccggcgcagcataannnnnnnnnnnnnnnnnnnnnnnnnnnnnnnnnnnnnnnnnnnnnnnnnnnnnnnnnnnnnnnnnnnnnnnnnnnnnnnnnnnnnnnnnnnnnnnnnnnnnnnnnnnnnnnnnnnnnnnnnnnnnnnnnnnnnnnCGGGGACATGCTGCCGTTGGCAGACGTTGGGTGTGACGGGTGTTTCTCTGGTGTGTAAGAGGAAGATATATTCCCCTTTTGTATCTGCACTGACCCCTGCACGGGGTACAGGCTATTGACGCTTCCTTTCGTCTGTGTGTCTTCACTGTTGcgtgtacggcgcgtgaacgggccatatannnnnnnnnnnnnnnnnnnnnnnnnnnnnnnnnnnnnnnnnnnnnnnnnnnnnnnnnnnnnnnnnnnnnnnnnnnnnnnnnnnnnnnnnnnnnnnnnnnnnnnnnnnnnnnnnnnnnnnnnnnnnnnnnnnnnnnnnnnnnnnnnnnnnnnnnnnnnnnnnnnnnnnnnnnnnnnnnnnnnnnnnnnnnnnnnnnnnnnnnnnnnnnnnnnnnnnnnnnnnnnnnnnnnnnnnnnnnnnnnnnnnnnnnnnnnnnnnnnnnnnnnnnnnnnnnnnnnnnnnnnnnnnnnnnnnnnnnnnnnnnnnnnnnnnnnnnnnnnnnnnnnnnnnnnnnnnnnnnnnnnnnnnnnnnnnnnnnnnnnnnnnnnnnnnnnnnnnnnnnnnnnnnnnnnnnnnnnnnnnnnnnnnnnnnnnnnnnnnnnnnnnnnnnnnnnnnnnnnnnnnnnnnnnnnnnnnnnnnnnnnnnnnnnnnnnnnnnnnnnnnnnnnnnnnnnnnnnnnnnnnnnnnnnnnnnnnnnnnnnnnnnnnnnnnnnnnnnnnnnnnnnnnnnnnnnnnnnnnngtgtttggttcctgttaggaaCCCCTTCGGGGCTTCTGTCTATTTTGCTCCCAAGACTGCTAGTACTATGGATGAGGCTGCGTCTCGCGCCCAGGGTTGCGAnnnnnnnnnnnnnnnnnnnnnnnnnnnnnnnnnnnnnnnnnnnnnnnnnnnnnnnnnnnnnnnnnnnnnnnnnnnnnnnnnnnnnaatcttttgtgtactgtcgttttgcggcgtgttgctttccctgcgttttcgctctGTGTTGGATATGCGGTCGCTCTTGCATACATGGCCTTCCTCGGAGGTGTGTGCGCACCGCGCATTAAGTGGCCCAACGACGTCCTGGTGTGCGatcgtaagatcgccggggtGCTCTGCCAGGTGCGCGCGGGAGCGCTTCTCGTGGGTATTGGGTGTAACCTCCTGCAGGTGAAGTTTCCGCCCGAGTTGTCGCATGCCTGCTCTCTCGCGCAGATTGTCGGAGGCGAGCGGTGCCCTGATCCGTTTGCTTTCCTTCCTGTGCTGTTGGATCGGCTGTATGCCTGTGTCATGGCGCCGCCGAGTATCGGCGTGCTTGAGTCGTGTCTGTGGAAGCAGGGTGAGTACGTCTGTTTCCGCGAATGTGCGGGAACACGTCCTCCGATTCTGGGTCGTGTTGTCGGATTGGCCGCGGACGGCGCGCTGTGCATAGATAGCGCnnnnnnnnnnnnnnnnnnnnnnnnnnnnnnnnnnnnnnnnnnnnnnnnnnnnnnnnnnnnnnnnnnnnnnnnnnnnnnnnnnnnnnnnnnnnnnnnnnnnnnnnnnnnnnnnnnnncattagccactgtgcacttacctCTCCGGAGCCGAGCGCCTCGTATACCCTCGTAAAGGCCTTGAGGTGGTCGGCGCACTCGCTAGCGGCGTACTCGGGGTAGCGACAATGGTTCCCTnnnnnnnnnnnnnnnnnnnnnnnncacaaaagcnnnnnnnnnnnnnnnnnnnnnnnnnnnnnnnnnnnnnnnnnnnnnnnnnnnnnnnnnnnnnnnnnnnnnnnnnnnnnnnnnnnnnnnnnnnnnnnnnnnnnnnnnnnnnnnnnnnnnnnnnnnnnnnnnnnnnnnnnnnncnnnnnnnntccccccagtgaagatccaaaaaagggttcaatgattgacacccccactgcggggcaggtgagcgccgccgtacaggacacgcgcatttgcgcgctttctgcaacggtgcgaaatacggcttcaagccaatccatcccctctnnnnnnnnnnnnnnnnnnnnnnnnnnnnnnnnnnnnnnnnnnnnnnnnnnnnnnnnnnnnncggttgcgtggactactttttcaaatatttctgtccgattacgcacgaacgcccgcncatcagcgacacacgtgcgttgcgctttttcttgctcatacggcgtgcctgaacgggcctggtacagatacccagtagccatgcgttccttgtgnnnnnnnnnnnnnnnnnnnnnnnnnnnnnnnnnnnnnnnnnnnnnnnnnnnnnnnnnnnnnnnnnnnnnnnnnnnnnnnnnnnnnnnnnnnnnnnnnnnnnnnnnnacttctgcagtggCGACACGGTCCTTTCCTAAGCACCACAAGCCATTGGACGTCTGTGCAGGCTCAAAGATGCCACGTCGGGGTACGCGAGTGCCAAACAGGAAACTATGTGTTTCCAATATGGTGTACGAGAATCCGTATGATTTTATAGTGCGCTCAAGCGCTGGTGCATAGCCAAGTTCAGGTAAATAAAAACCGCGGGGAATTGAGGAAAAATGTTTGCGGTAATTAATAAGCCCCATTTCGATTTGGGCGGATATAGATTCGGGCATGTCTTGGTAGAAGGGTAAGAAACAATTAACTGCCGTTGTTGCGAGTAATTCAATGGAACCTGTGCGGAAAAAGTGATTGATGCGTTCAAGGAGTGCCCCATCGCAGTGATCAAAATAATCCCGGTGAGAGCGAAGAGACCGAAGCACTGCTTCAGCTTGCACGCGCTCTTGGAGACtgttcctcnnnnnnnnnnnnnnccgttcTCCGAATTCGATGAGTGCGTCGAGTGCACGCCGGTATCGGTCCATAAGCACGCGGTTAGCGAGCATTTCGCACAGAACGGGCCCGATAGCGAGGGAGATGTTAAAAGGAACACGCTCACGTTCGAGTGTTTCGCATAAGCGGAGTAGGGGGaggtaggtATAGGAAATCTCGAGAAAAAAACGGGATTCAGCGAGGAGAGAAGATGCGCCGGCCCCTCGGACGAAGGGAAGATTACAATCGAGAACAAATGCAAGAGAGTGCACTGGCATTTTCTTCATGAAGGTCCCACTTATGAGCACACAGGAAGAGACGTGCTTGGGTAGGTTTCCTCAAGCAGGGGAAGCCCACAGAGGctgagaacttctgcgccggttggatcctcggggttaagactcngttctatGACGCTGCGCTGGAGCGTGACAACTCTGGATnnnnnnnnnnnnnnnnnnnnnnnnnnnnnnnnnnnnnnnnnnnnnnnnnnnnnnnnnnnnnnnnnnnnnnnnnnnnnnnnnnnnnnnnnnnnnnnnnnnnnnnnnnnnnnnnnnnnnnnnnnnnnnnnnnnnnnnnnnnnnnnnnnnnnnnnnnnnnnnnnnnnnnnnnnnnnnnnnnnnnnnnnnnnnnnnnnnnnnnnnnnnnnnnnnnnnnnnnnnnnnnnnnnnnnnnnnnnnnnnnnnnnnnnnnnnnGGTCACGGGCAAGGATATGGATTTCGGTGGTATTGTAGGCACACGAagtcccctttgattcaagGGACTGGGGagcgcacggagggttcaaatcaagagcgctgtggtaacaatcaaggatnnnnnnnnnnnnnnnnnnnnnnnnnnnnnnnnnnnnnnnnnnnnnnnnnnnnnnnnnnnnnnnnnnnnnnnnnnnnnnnnnnnnnnnnnnnnnnnnnnnnnnnnnnnnnnnnnnnnnnnnnnnnnnnnnnnnnnnnnnnnnnnnnnnnnnnnnnnnnnnnnnnnnnnnnnnnnnnnnnnnnnnnnnnnnnnnnnnnnnnnnnnnnnnnnnnnnnnnnnnnnnnnnnnnnnnnnnnnnnnnnAGTGTCGTGTCTTCCCCCTAGAATTCGCCATACGCTTGACCAAAAGGACAATGTGATGCAGCCTGGCGAGCGAGCTACTAGGAGGTATGAAAATGGCTGGTGCCAGCAAAAATTCGCGTACTGCGGCGGCGACGCAGCGCTTTAACnnnnnnnnnnnnnnnnnnnnnnnnnnnnnnnnnnnnnnnnntaacggaaagGTGAAGAACATCGCTGAGTGTCCTAAGTGCAGGCGTGTTGAGCGTCGGCCGCGTGACTTTAACTAGGAGTCTTTGGAGGCGCGCGTGAGGCACTCGTCTTGAGGACAGGTGGTCTCCGAGCGCCAGACGTCTAGCTGCGCTTGAATGAGCGCTTTTGCCTCGCGTAGGAGGGAGAGCTGTTCGTCTTTGTTGTGGGGTGCGGGGTAAACGCCGAGCACTTTGACGCGGTAAGCGCTTCCTTTAAGCTTCTGTCCTATTTTCAGCAACGGGCGTAGGTTCCACCCTCCGTCCAACGCGCATACAACTACGGGGAGTGGGAGCCGTGCCGTGAGCGCACGGAAGCCTGCGGCGTGGAAGATGCGCAGGGCGCCGTTTCTTGATCGTGTCCCTTCAGGGAAGATCACAGGGAGCCATCGCTCTCGCATGACGTGGTGTGTAAACGTTTCGAGCGCTTGCATCGCCTGTGCTCCTGACTGATGCCGGTCTACCAGGCAGTGCCGACCGCTTTTGAGCATGGTGGACACAAGGGGGACGAAGCGCGCGAGCTCTCGCTTTGCTACGAAGCGCAACCTCGGCGCATCAATATAGCCAAAGTAACGCATGAGTGCAGGGGTGTCTAGCACACTTTGATGATTTGAGACTACAAGGTACTGCGTGGGCAGGTGCTCAGCGTGGGTGTAGTCgctgtaaaacttaagtcttccgtaggtatgtagnnnnnnnnnnnnnnnnnnnnnnnnnnnnnnnnnnnnnnnnnnnnnnnnnnnnnnnnnnnnnnnnnnGTACAGTGCGTAGCAGAGGGTGTTTGCAACTGCCAGCGGTGCAAGAACAGCGATACTTACGACGATGGAGAGAATTCCGCACATGAGGGCGCTCCTTCTGCGTTGGTTGACCACCGCACAGGGGAGCATTATGCCATAAAGCGCGCGTGCGCGCCTTTACTCTGGCGGACCTTTTCTTTATCATCGGCGGCGGAGACATGAACGAtggcaagaagatGTGGACTGTGTGGAAAGGGGACGAnCAGCGGGTGCGCCGTGAGTAAATCGATGCACCACTGCAAGCGGGTGTGGAAACCGAACCTGCTCGCAGTGAGAGTAGTTGTAGATGGCTCTGCGCTGAACATGCGGATTTGCGCGCGCTGCCTACGCagcaaccctcttatgaaaaaggctcagccgnnnnnnnnnnnnnnnnnnnnnnnnnnnnnnnnnnnnnnnnnnnnnnnnnnnnnnnnnnnnnaagagaagcacgccgcaTGCGCCGCTGGCTACGGCCCTGAGAGAGAAGCCGCAGGCTCCCATGCTGGAGTAGGTCGCCGCGGACCAGAGAACGACGGGGGCTATCCACATCGTGCTGTCGTCTCTGAAGGTGCGCACGTCCCGAACGGTCAAGAGCACCGCGCCGAGGAAGAGGCTGGCGTGTCcgccaatcgatacccaagacgaccgcgcgtgggtagaannnnnnnnnnnnnnnnnnnnnnnnnnnnnnnnnnnnnnnnnnnnnnnnnnnnnnnnnnnnnnnnnnnnnnggaccagtcccgcggcatacaagGagtaaccaccgagggcgagaaaccgacctgacaaacgtacctgcgcgcgggccatggcggaGTCTATTCTCGGCTGATGGAAGTGTCAAGGGATGCAGCGGGTGGCGGGGTCTTTTGGACGTTGCCTTGACCAAAGGCTGAGAATGCGGGaagattgtgcccatgagcgattaccTCGACGCGGGCAACGAAGAGTTGCTGAAGGATTTTTTcagCGAGGCGGAGCAACAGGTTGagcagctcgaaagcaatatactcgtcattgagcaggatcccaccaatcgggacgctgtagacgagattttccgtgcggcgcACACCCTTAAggggggtgctgcgactgttgagatgcacgagtngtccggcnnnnnnnnnnnnnnnnnnnnnnnnnnGGATGGGATTCGCTCAGAGAAGGTAACTGTTGACGGGGCGGTTGTGGACTTGCTCCTGACGTCGTTGGATGTAATtaaggcnnnnnnnnnnnnnnnnnnnnnnnnnnnnnnnnnnnnnnnnnnnnnnnnnnnnnnnnnnnnnnnnnnnnnnnnnnnnnnnnnnnnnnnnnnnnnnnnnnnnnnnnnnnnnnnnnnnnnnnnnnnnnnnnnnnnnnnnnnnnnnnnnnnnnnnnnnnnnnnnnnnnnnnnnnnnnnnnnnnnnnnnnnnnnnnnnnnnnnnnnnnnnnnnnnnnnnnnnnnnnnnnnnGAATACGATCGGTTGGAATTGCGCGAGATCGTTCCGCCnnnnnnnnnnnnnnnnnnnnnnnnnnnnnnnnnnnnnnnnnnnnnnnnnnnnnnnnnnnnnnnnnnnnnnnnnnnnnnnnnnnnnnnnnnnnnnnnnnnnnnnnnnnnnnnnnnnnnnnnnnnnnnnnnnnnnnnGTATCAGGACATGTTCCATGAGTATGTTGTGTACTTCGTTTCCACTGTGCAGGATTCAGTATGTGTGTCGCAGGTTGCTTCGATTCCGGACGTTACGCTTTCGGTGagtgtagcggaagttgcgcttgcagatctgtgnactcccctctctgaacatggggagctgggtgtttgcgcagacgcggggacaggtggtgtgtccgaagggagcggcgctgccgtgcgcagcggaggggaacgcgttcctannnnnnnnnnnnnnnnnnnnnnnnnnnnnnnnnnnnnnnnnnnnnnnnnnnnnnnnnnnnnnnnnnnnnnnnnnnnnGGCTCGGGGTCCGTGTTGCGCGTAGATGCGAAACGCATCGATTATCTTCTGAATTTGGTAAGTGAGACGGTGATTATCAAGGCCTCGCTCAATCAGAGTGCGCTTGAATTTGGGGAGGTGTACACCCTATTCCAAAACGCTAATGGCGCGTACAAGGAGCGTTTGCGTAAGTTTTTTGATAGGGTTCCCGCTTACTTAGAAAAGGTAAAGAACGGTCAGGACGCAGATGCGGTGCGCAAGGGGATGATAGCAGAGGCTGTCGGTGTCTTTGACATTTTTTCTTCGTTTGAGAATGGACTGAAACAGTCCGTCACTAAGTTTCGGTCTTCTGCTCAGAATTTGGGGCGTATTTCTGGTGagcttcaagaaggtgtgatgnnnnnnnnnnnnnnnnnnnnnnnnnnnnnnnnnnnnnnnnnnnnnnnnnnnnnnnnnnnnnnnnnnnnnnnnnnnnnnnnnnnnnnnnnnnnnnnnnnnnnnnnnnnnnnnnnnnnnnnnnnnnnnnnnnnngttgaagatttgctcgatcccattatgcactgcgtgcgtaattctctcgaccacggcatagaagcgcctgaagttnnnnnnnnnnnnnnnnnnnnnncgcaaggtacgcttctcctgcgcgcaagcaacgaaggaaatatgatcgttattgnnnnnnnnnnnnnnnnnnnnnnnnnnnnnnnnnnnnnnnnnnnnnnnnnnnnnnnnnnnnnnnnnnnnnnnnnnnnnnnnnnnnnnnnnnnnnnnnnnnnnnnnnnnnnnnnnnnnnnnnnnnnnnnnnnnnnnnnnnnnnnnnnnnnnnnnnnnnnnnnnnnnnnnnnnnnnnnnnnnnnnnnnnnnnnnnnnnnnnnnnnnnnnnnnnnnnnnnnnnnnnnnnnnnnnnnnnnnnnnnnnnnnnnnnnnnnnnnnnnnnnnnnnnnnnnnnnnnnnnnnnnnnnnnnnnnnnnnnnnttgatacgggtggggcaggaggtgtactcgataccgatagcttcggtcatcgaaagCCATCGTATCAAGAGTGAAGAGATTAACCGGATTGATAACTACGAGGTGTTCAATGTGCGGAACGAGGTCATTAGCTTGCTGCGTCTCGATAGGCTGTTCGGTATCAGCTGCGATGATGAGGTGACCGGTCAGTATCACTATGTGGTTATAGTTggtgcggcagagaaaaaggtgggGCTCATGGTGGATGCGCTGATTGGTGAGGAGGACGTAGTCATCAAGCCACTGCGGGATCAATTCACTAGTTCCCCTGGTATTGCAGGGGCATCTATCCTGGGTnnnnnnnnnnnnnnnnnnnnnnnnnnnnnnnnnnnnnnnnnnnnnnnnnnnnnnnnnnnnnnnnnnnnnnnnnnnnnnnnnnnnnnnnnnnnnnnnnnnnnnnnnnnnnnnnnnnnnnnnnnnnnnctatgatagagcatatggaagcagagatcggcattcgggaaggtttcgacgggggcgtacgtgagccgcttgcggtcatagaCttcaagatggttaCCTTTTCCCTCGCGGGGAAGGACTACGCGGTAGATATCATGCAGGTGAAGGAAATTGCAAAGGCTGGGAGCTTTACCTATGTGCCCAATACGTCTCCGTTTGTTCTGGGGGTGTATAACTTACGGGGGGATATTATTCCCATAATTGATTTaaGGAGATTTTttaatattcccgctccgcgcaagtcccggcaggcgatcgagaatatggtgatcgtcacagtggaagatcagACATTcggggttgtagtagatggcatcgataaggtaattggggtgtcaaaaacaactattcagccgccacannnnnnnnttggggacancancataaagtatatccggggggtggttgaggaggcgggaaagctgtacatcctacttgatgnnnnnnnnnnnnnnnnnnnnnnnnnnnnnnnnnnnnnnnnnnnnnnnnnnnnnnnnnnnnnnnnnnnnnnnnnnnnnnnnnnnnnnnnnnnnnnnGTGCCGCCGGGGGATGAAGAAAATTTAAATGTTGGTTTCATTAGCGATACGTTGGCCGCGTTTGGCCGTTTCTTTACCAGTGCAGTGAATGAGGGTTGGTTGCGCAGCCGGTATCTTGTGTGGCGTGACGTGCGCTCTGGAGCTGAGGTACAGCTTCAGCATGAGGAGGATGTCGCCGAGTTCTTGAGTACATTTCCTTCCCCGGACACAGGTGTGTTTTGGTCGGGGGAGTATGCGGCGAGTGTGGGATCTGTTCTTTCTCGGATGCAGGTGGGAAAGGTGGTGACGGTGTGGAATATCGGnnnnnnnnnnngtcacgaaagttacagtcttgcggtgcttctcagaaaaaccttccccgacgcggtggttnnnnnnnnnnnnnnnnnnnnnnnnnnnnnnnCCATTTCCAATGCTCCCATGCTCACTGTTCCTGAGCATGTGATCGGTGATTGGTATAAGCCCTATGTGGTGAAGGGGGTGAGTGGTTCATACACCTTCTCCCAnGAAATTAAGGAGATGGTCCTGTTTGAGTACCACGATTGTACGCATCCGAGTGCGCTTCCAGACGTCGATCTTATCGTGGCGCGGGACGTACTGTCATCTCTtgcggttccagtgcagcacaccctgttgaAGGAGTTTTCTGAGAAGTTGAAGGCAACAGGAGTTGTTCtgctcggtcagaacgaggtgatgcctaaggatacaggatggttgcggcagattgaaggcaccgttgcggtGTtcagcaaggaataattagcgcatgaggagtggtgtatgcgnnnnnnnnnnnnnnnnnnnnnnnnnnnnnnnnnnnnnnnnnnnnnnnnnnnnnnnnnnnnnnnnnnnnnnnnnnnnnnnnnnnnnnnnnnnnnnnnnnnnnnnnnnnnnnnnnnnnnnnnnnnnnnnnnnnnnnnnnnnnnnnnnnnnnnnnnnnnnnnnnnnnnnnnnnnnnnnnnnnnnnnnnnnnnnnnnnnnnnnnnnnnnnnnnnnnnnnnnnnnnnnnnnnnnnnnnnnnnnnnnnnnnnnnnnnnnnnnnnnnnnnnnnnnnnnnnnnnnnnnnnnnnnnnnnnnnnnnnnnnnnnnnnnnnnnnnnnnnnnnnnnnnnnnnnnnnnnnnnnnnnnnnnnnnnnnnnnnnnnnnnnnnnnnnnnnnnnnnnnnnnnnnnnnnnnnnnnnnnnnnnnnnnnnnnnnnnnnnnnnnnnnnnnnnnnnnnnnnnnnnnnnnnnnnnnnnnnnnnnnnnnnnnnnnnnnnnnnnnnnnnnnnnnnnnnnnnnnnnnnnnaacgatcgggttcccgcaggacaaaaaccgaatggggcgccctatcgnnnnnnnnnnnnnnnnnnnnnnnnnnnnntttcaaagcagattggtcaaatcttgacaagtgaaggctacgaggttgcagatactgcggtggacggcgttgatgnnnnnnnnnnnnnnnnnnnnnnnnnnnnnnnnnnnnnnnnnnnnnnnnnnnnnnnnnnnnnnnnnnnnnnnnnnnnnnnnnnnnnnnnnnnnnnnnnnnnnnnnnnnnnnnnnnnnnnnnnnnnnnnnnnnnnnnnnnnnnnnnnnnnnnnnnnnnnnnnnnnnnnnnnnnnnnnnnnnnnnnnnnnnnnnnnnnnnnnnnnnnnnnnnnnnnnnnnnnnnnnnnnnnnnnnnnnnnnnnnnnnactaaagtgagggcggatgtgtcctgggggctgtCTCGTACGGTTTGCCCGCTTGCGTGTGTGGATGGTTTCTTGAGGTTTTTGCCTTCGCGCGCGGAGTGCCCGTCTCTCnnnnnnnnnnnnnnnnnngtgtgtgccgcaagaggagaagnnnnnnnnnnnnnnnnnnnnnnctcggggccctgtggtgcctttcctgcggtgtagtttctatactccttcgtagttcctagttggtttggttggaaagggttcggttcgATTTTGAAGAGGTGCACACGTTGTATTGTGCGCATGAAAGAGGGAGCGGTGTGTGCTCTTCCTGAAAACGCTTGAGGTATTTGGCTTTAAGTCGTTTGCAGATCGCGTTCGCGTTGAGTTTGCAGATGGCGTCACTGCGCTGTTGGGCCCAAACGGCTGTGgcaaaagcaatgtcgttgacgccataaagtgggtcctcggagnnnnnnnnnnnnnnnnnnnnnnnnnnnnnnnnnnnnnnnnnnnnnnnnnnnnnnnnnnnnnnnnnnnnnnnnnnnnnnnnnnnnnnnnnncctCTCTTACCGTTTGCGATGAAGCTGGTATCCTTTCGCTCGATGTGCCAGAGATTTTAATTAAACGCAGACTCTATCGTTCCGGGGAAAGTGAGTACTTTCTTAACGGGAATGCCGTCCGTCTAAAGGAGATCCGCGAGCTCTTTTGGGAtacnnnnnnnnnnnnnnnnnnnnnnnnnnnnnnnnnnnnnnnnnnnnnnnnnnnnnnnnnnnnnnnnnnnnnnnnnnnnnnnnnnnnnnnnnnnnnnnnnnnnnnnnnnnnnnnnnnnnnnnnnnnnnnnnnnnnnnnnnnnnnnnnnnnnnnnnnnnnnnnnnnnnnnnnnnnnnnnnnnnnnnnnnnnnnnnnnnnnnnnnnnnnnnnnnnnnnnnnnnnnnnnnnnnnnnnnnnnnnnnnnnnnnnACGTTACCGCATGCTCAAAGAGGAGATTTTTGCGCGAGATCGCGATCTTGGTCtgttgcgnctgcgtgggtttttagaaaaccaagcccgagcggatggaGCACTCCAGCGCAATCGCGCGCGGCGCGACGCGTTGCAAACACAGGTGGAGGAAGCACAGCAGACGCTTTCTGCTCGCATAGGCGAGATCAATGAtatgGAAAAGCGCGTTGACGCGCTCCAAAAGGAAATCTATGGCCTTGCAATTGAACagaaagcgaagcaaaacgaggcatcgctacatcnnnnnnnnnnnnnnnnnnnnnnnnnntcgattggtcagatagaaatgcgcaagattgGTGTAGAAAgtcgcgtgcagaatttggaagaagaagtagcagagcaAGACGCACACGTGTATCAGTTAGGCAGTGCTCTATCCTCTGTTGAAGAGCATATTGAATCGTTTGCGCGGAGCTTGCACGTTGCAAGTGAGCACGTCTCAGAGAATGATCAAACGCTTCGCGACATACAGGGACAGATGCAAGAGATAAGTGCCGCGTGTGTTGAACTTGAAGCgtccctacgtgacgtggcagaagatattgCCGCAGAGCTTGACACGCGCCTGAGTGCAGCCGGGTACTCTGCGCGCAATCGGGCAGAGGCTGAGCGTACGTTGGTAGCGGGGGTACAGCGCCnnnnnnnnnnnnnnnnggGGAGAGCACGTATTGTTTCAGACTTTCTGGTGGTAGATACCCACACTGAAGGGGAGCTGTGCCGGATGCTGACTACAGTTGTGGACGCGTTCAATGAGGCGGTAAAGATAGTGCACTGCGTTGAGTCAGACATAGCAGAATATGCGCGTGTTTCTGCCCGGTTTATCGATGnnnnnnnnnnnnnnnnnnnnnnnnnnnnnnnnnnnnnnnnnnnnnnnnnnnnnnnnnnnnnnnnnnnnnnnnnnnnnnnnnnnnnnnnnnnnnnnnnnnnnnnnnnnnnnnnnnnnnnnnnnnnnnnnnnnnnnnnnnnnnnnnnnnnnnnnnnnnnnnnnnnnnnnnnnnnnnnnnnnnnnnnnnnnnnnnnnnnnnnnnnnnnnnnnnnnnnnnnnnnnnnnnnnnnnnnnnnnnnnnnnnnnnnnnnnnnnnnnnnnnnnnnnnnnnnnnnnnnnnnnnnnnnnnnnnnnnnnnnnnnnnnnnnnnnnnnnnnnnnnnnnnnnnnnnnnnnnnnnnnnnnnnnnnnnnnnnnnnnnnnnnnnnnnnnnnnnnnnnnnnnnnnnnnnnnnnnnnnnnnnnnnnnnnnnnnnnnnnnnnnnnnnnnnnnnnnnnnnnnnnnnnnnnnnnnnnnnnnnnnnnnnnnnnnnnnnnnnnnnnnnnnnnnnnnnnnnnnnnnnnnnnnnnnnnnnnnnnnnnnnnnnnnnnnnnnnnnnnnnnnnnnnnnnnnnnnnnnnnnnnnnnnnnnnnnnnnnnnnnnnnnnnnnnnnnnnnnnnnnnnnnnnnnnnnnnnnnnnnnnnnnnnnnnnnnnnnnnnnnnnnnnnnnnnnnnnnnnnnnnnnnnnnnnnnnnnnnnnnnnnnnnnnnnnnnnnnnnnnnnnnnnnnnnnnnnnnnGGTAAAGGAGCGCTACGAGTTTCTCGTTGCGCAGGTTGCGGACCTTGAAAAGGCGCGCGCAGATCTGCAGCGGGTAACCGATAAAATTAAGACTGAATCTGCAGAACTTTTCTTGGCAACATACCGACGGATTCGTAAGAATTTTCACGAGnnnnnnnnncgtctgtttgggggaggtcgcgcagagatacgtctttcagatcctgcagcggnnnnnnnnnnnnnnnnnnnnnnnnnnnnnnnnnnnnnnnnnnnnnnnnnnnnnnnnnnnnnnnnnCTTTCTGGTGGAGAAAAGGCAATGACTGCAGTAGcgttgctctttgcaacgtaTATGGTGAAGCCTGCGCCGTTTTGTCTTTTGGATGAAATCGACGCAGCGTTGGATGAGCATAATGTAGCTCGTTTTGTTGGGATGCTTGATGAGTTTTctgacgtcagtcaatatatcgtaatcacgcacaatcggcggacgGTTTTGGGTGCACGCACCATGCTtgGGGTAACAATGGAAGAGCCGGGGGTATCGAAAGTGGTTTCGATTGCACTTGAATCTGCTTCTGAGCGACCGGCTAACGGCGAGGCAGGAGGAGCCATTTGATGCGTCTGCGTGGGGTGGCAGGTGCCCTGTTGGGTGCGGTAGTGCTTGTGGCGTTGGGGCTGATGGGCGTCTGGTGGGtgttctaTCCAAAAAAAGGGGACCGTGGGGCGGCTGTGGCTCGCGAGCCAGTGTTGTTGCACATAGATCCTGCACAGATGGAGGCAGCTGATGAACCGTTGACGCTTCCCCCTATCGAGCGTTCCCGTGAGCGGATGTCGGCGTGGAGTGAGCAGGAGnnnnnnnnnnngcttgagtatccgacggaaaaggcggtgcaggcattagagcacgcaaaCGAGAAACGTATACAGCAGATGCTAGAGGCAGTACCGTGAGTGTGTGGGTGGCGCTCGCCTTGCTGGGAATGTGTGTTTCGTGTACGCACGTGCCTCCGCCTCGTGCCCTCATCGTTTCAAAGGAGCCGCCTCCAGCGTTGGATTCTGCGCCGCGCCCTGCGATTCCAGAAGCAGTTCCTCTTCCGTCCCCTGTGGAGGAAGAAATCGCCGGTCGCCTCCCTCCTGCACCTGCCGCTGCACCTGAGCGCGTTCCTGAGTCCTCACAGGAGCGGGAACAGAAACCTGAGTCTTCGAAGCCTCAGGTGGTAGAGCCGGTGTCGCTTGCCTCTCCGGTGAAGCCTCGCGAGGCTGGGAGTGTACCTGATGTTCTTCCAGTACCTGAAGTGTCGTCGCTGCACGTTGCGCCGCCGGCACCCCCTGCGCCGACAGCTCCCCGGCCGCAnCGTCCCTCCCCTCCGCCTGTATCGCCTTCTGCATCCAAACCAAAGCAGCGCGCTGTACCTCCTTCTCCGCCCCCTGCATCAGAGCCTCCTCGTGAGGcggaggtgnnnnnnnnnnnnnnnnnnnnnnnnnnnnnnnnnnnnnnnatggtGCCTGAAGAACCGCCTGAGGATGAGGTGCCGCGCGTTTCGCGCGCGGTACAGCTTGCAGTGGGGCAAAAACTTGAGGTTTTGTATCCGGGCGAAGGTTGGGTGnnnnnnnnnnnnnnnnnnnnnnnnnnnnnnnnnngctatcaccnnnnnnnnnnnnnnnnnnnnnnnnnnnnnnnnnnnnnnnnnnnnnnnnnnnnnnnnnnnnnnnnnnnnnnnnnnnnnnnnnnnnnnnnnnnnnnnnnnnnnnnnnnnnnnnnnnnnnnnnnnnnnnnnnnnnnnnnnnnnnnnnnnnnnnnnnnnnnnnnnnnnnnnnnnnnnnnnnnnnnnnnnnnnnnnnnnnnnnnnnnnnnnnnnnnnnnnnnnnnnnnnnnnnnnnnnnnnnnnnnnnnnnnnnnnnnnnnnnnnnnnnnnnnnnnnnnnnnnnnnnnnnnnnnnnnnnnnnnnnctggagcgcctgtccggcaggatcaGACGGATACTGCTGTTGCAGAGAAAGCTCAGCACGGTACGCCCCGTCCTGATGAAAAAAAAGATCGGGAACCCACCGTAGGAGGACGTGACCCTGTGCCGTCGGATGCAGTAGCACAGGGTGTGTCggagcgatactctccgcggaagattnnnnnnnnnnnnnnnnnnnnnnnnnnnnnnnnnnnnnnnnnnnnnnnnnnnnnnnnnnnnnnnnnnnnnnnnnnnnnnnnnnnnnnnnnnnnnnnnnnnnnnnnnnnnnnnnnnnnnnnnnnnnnnnnnnnnnnnnnnnnnnnnnnnnnnnnnnnnnnnnnnntctttgcgcagttcccttctcacgaacgaatggacgaagcgtggttcctgcgtggacaagcctatgagatcnnnnnnnnnnnnnnnnnnnnnnnnnnnnnnnnnnnnnnnnnnnnnnnnnnnnnnnnnnnnnnnnnnnnnnnnnnnnnnnnnnnnnnnnnnnnnnnnnnnnnnnnnnnnnnnnnnnnnnnnnnnnnnnnnnnnnnnnnnnnnnnnnnnnnnnnnnnnnnnnnnnnnnnnnnnnnnnnnnnnnnnnnnnnnnnnnnnnnnnnnnnnnnnnnnnnnnnnnnnnnnnnnnnnnnnnnnnnnnnnnnnnnnnnnnnnnnnnnnnnnnnnnnnnnnnnnnnnnnnnnnnnnnnnnnnnnnnnnnnnnnnnnnnnnnnnnnnnnnnnnnnnngtcgctgtcttcaaagtcgtcctcgtagtcgtccgagccttggnnnnnnnnnnnnnnnnnATACAGGGCGCGTGCGAGTAACTTGTTTCTCTCGTTCATATGGCTGACCTCTGCTTGTGGTATGGTGAAAGCTAGGGGTGGATCGTAGAGGggagcgcacacgctgtcaactGtgcctaataggcacaggtattttttctCTTGTGTTTTGGGnnnnnnnnnnnnnnnnnnnnnnnnnnnnnnnnnnnnnnnnnnnnnnnnnnnnnnnnnnnnnnnnnnnnnnnnnnnnnnnnnnncgcaaaGGTGAACATGCATCTGTGGGTTGGTGCACGTCGGGCCGATGGTCTTCACTCGATTGAGAGTGTAATGCAGCGCATTACGCTTGCGGATTCTTTGTCTCTTTCGCGCCTGGATATCCCTGGCCGGTGTGAGGTGTGTTCGCCTTATATGGCTCTGCCTCGAGAAAAnACGCTGACCCGTGCGTATGCGCGGTTTTGTCAGGTAACTGGCGTTCACGATGGTGTGCGCGTGCGCGTAGTGAAGCGTATCCCTGCAGGGTCTGGACTTGGTGGGGGGTCTGCTGATGnnnnnnnnnnnnnnnnnnnnnnnnnnnnnnnnnnnnnnnnnncccTTTCTGctcgGGTTCTACGAGAAGTGGCGTATAGTGTCGGCAGTGATGTCCCTTTTTTCCTGGCTTCCCAGGCTGCGTGCGTGCTAGGTGGTGGTGAGCAGCTCGTTCCCCTCGTTCCAAAGACTGGCTATCTGGGGCTTTTGGTCTGGCCGGGATTGCACAGCGGTTCTGCACAGGCCTATGAGGATTTGGACAGGTTGCGTGCGTGCGGCGTACATGCTGCCGACGGCGAGCAGTACTCTCTGCGTGGGGCTACCGCGCTTTCTGCGCACTATGCACAGGATTGTGCGCGGTGGCGGTTTTTTAATAGCTTAGATacccctGTACAGCGTCGGTACCCTGTAGTCGCCCTTGCGCGTTGGGATCTGGCCCGTGCGGGTGCGTGCtTTACAGCGATGAGTGGAAGCGGGAGCGCGGTTTTTGGTCTGTATCGGGATGAGGAGGAGCTTCGGCGCGCGCATAAGCTGCTTGCAAagcggtGGTGTTGGTGTGTGCGTGTCCGGTTATGTGGGTAGGATATCTTTGGGACGTCGGCAAGTGGTAAGCCAACGGTTTTTGGTACCGTCATTCCGTAGGTTCGAATCCTACCGTCCCAGTTTGCGGCGTGTTCGCTTTCTGTGCATGGAGGATTGGATTGTGGATGAAAGGCGTTTGAAGGGGAAAAGGCGCGTCCAGTTGGGGAAGnnnnnnnnnnnnnnnnnnnnnnnnnnnnnnnnnnnnnnnnnnnnnnnnnnnnnnnnnnngggcgtgtccgttccccttgaacttgcgcagcaagacttcgatagattgtttcgtgccttaaccaggaGTACTGTTCTGTCTTTGGAACTGGATGGTGGCGAGGTCTTTTGCGTTTTTGTTAAGGACTATCAGCATAACATGGTCAGTGACCGTGTGGAGCATGTGGATTTCTACGCAGTTGAAGAGAGTGTTCCGTTGCGAATGCGAATCCGGCnGCAGTTGTGTGGTTCTCCTGAAGGAGTGCGCTATggggctcgcttggaaaaggggctctcatatatcnnnnnnnnnnnnnnnnnnnnnnnnnnnnnnnnnnnnnnnnnnnnnnnnnnnnnnnnnnnnnnnnnnnnnnnnnnnnnnnnnnnnnnnnATGTTCCTCTGCCCGCGTCGGTGGTGGTTCTCAGCGATCCTGATGCGGTGATTGTGGCACTCAGTTCTTCTGCTTCCGAAGCGGGTTCACCCGCTGGCGCGAGGACCGGCTAGATGGTGCGCGGTGTAACGCGCGCGGTGCGGCACACtgtgcagcgtgcccgtctttatnnnnnnnnnagtctAGGCAGAAGCTTCACCCGCTGCTCGTCCACGTGGCGCGTAGCTTTGGGCATTTCCTTGTGCCGAGAAAGCCTTCTTGCTTGCTCGTGGCGGTTTCAGGAGGTGCCGATTCGCTTGCGCTTCTTTATGCGGCGCACGAGCTCGCTCCTGACTTTGGGGTGTGTGCGTGCGCGGTCACGGTTGATCACAGTCTGCGCGCTCAGGAAGGTGCGCTCGATGCGCGTTTTGTGCGTGCGCtGTGTGCTCGTTTTTCTCCTCCCCTTCCGTGTTTCGTGCAGCAGATTTCTGCCGGTGCGGTGCACGCGTGTGCTAAAATTCGTGGCCGCGGTGTGCAGgacgcggcgcgtgcactgcgctataaggtctttgaccacgtggctgctcgCTGCGGAGCACAGGTGGTTCTAACCGCGnnnnnnnnnnnnnnnnnnnnnnnnnnnnnnnnnnnnnnnnnnnnnnnnnnngcggccgcgtccgcgttgcaaggtatacgagctgcgcgtggacggtacgtgcgtccgctgctaaaggtGAGCCGCACGTGTGTTGAAGATTTCTTGCAGACGCGTGGTGTGCGTTGGCGCGAAGACGCATCAAATACGTGCaggaaatatgnnnnnnnnnnnnnnnnnnnnnnnnnnnnnnnnnnnnnnnnnnnnnnnnnnnnngggtggcgctctGGCTTAGACAAAACGTTTGCGGGGATTAGTGCAGAACACAGCTTTTGTGTTGCTGCCTTGACGCGGTGGCGTGAAGGTTGTTCGCATGCGTGGGAACCAGTACCACGTGCGTTGGGCACGCGTCTGCGGATGCCTCGGTCGGACTTTCTCGCCGCTGAATTTATATTGCGCTTCTTTCTTTTGCAGGAGGCTTGCGTTCGtttgggagtttcgcacnnnnnnnnnnnnnnnnnnnnnnnnnnnnnnnnnnnnnnnnnnnnnnnnnnnnnnnnnnnnnnnnnnnnnnnnnnnnnnnnnnnnnnnnnnnnnnnnnnnnnnnnnnnnnnnnnnnnnnnnnnnnnnnnnnnnnnnnnnnnnnnnnnnnnnnnnnnnnnnnnnnnnnnnnnnnnnnnnnnnnnnnnnnnnnnnnnnnnnnnnnnnnnnnnnnnnnnnnnnnnnnnnnnnnnnnnnnnnnnnnnnnnnnnnnnnnnnnnnnnnnnnnnnnnnnnnnnnnnnnnnnnnnnnnnnnnnnnnnnnnnnnnnnnnnnnnnnnnnnnnnnnnnnnnnnnnnnnnnnnnnnnnggagacaccatctccatccgcggtggtcataagnnnnnnnnnnnnnnnnnnnnnnnnnnnnnnnnnnnnnnnnnnnnnnnnnnnGTGTTACCGATGATAGAGCAGGATGGTGTGTTGCGTGCGCTCTATGGCGCAGCACTTGGTTACCAAAatcgttacgcggagaggactcctcatgagtaagggnnnnnnnnnnnnnnnnnnnnnnnnnnnnnnnnnnnnnnnnnnnnnnnnnnnnnnnnnnnnnnnnnnnnnnnnnnnnnnnnnnnnnnnnnnnnnnnnnnnnnnnnnnnnnnnnnnnnnnnnnnnnnnnnnnnnnnnnnnnnnnnnnnnnnnnnnnnnnnnnnnnnnnnnnnnnnnnnnnnnnnnnnnnnnnnnnnnnnnnnnnnnnnnnnnnnnnnnnnnnnnnnnnnnnnnnnnnnnnnnnnnnnnnnnnnnnnnnnnnnnnnnnnnnnnnnnnnnnnnnnnnnnnnnnnnnnnnnnnnnnnnnnnnnnnnnnnnnnnnnnnnnnnnnnnnnnnnnnnnnnnnnnnnnnnnnnnnnnnnnnnnnnnnnnnnnnnnnnnnnnnnnnnnnnnnnnnnnnnnnnnnnnnnnnnnnnnnnnGTCTGCAGAtgcagattggttgcgtgcccgggcacgttacgggctcggacaagttgagcacgcgcaggagctgatcgAAAAAGCGTTGGAACGTTGGGCGCTTGACGCGCGCTTTGCAAAGTTGTTTTTTGCGCAAGAGCGTTCACGACGTCCCTCGTCGCGGTCAAAAAAGATAGCAGATTCCATCCTGTCACGTCTTTCTGTATGGCAGGAGCAGGACCCATCGCTGCTTGTAGAGGCGGCTCTATTTGAACCTCGAACGAnCATGGCATTTCGATannnnnnnnnnnnnnnnnnnnnnnnnnnnnnnnnnnnnnnnnnnnnnnnnnnnnnnnnnnnnnnnnnnnnnnnnnnnnnnnnnnnnnnnnnnnnnnnnnnnnnnnnnnnnnnnnnnnnnnnnnnnnnnnnnnnnnnnnnnnnnnnnnnnnATGGAGTGCTGGATGAACAACGCGCGATGGAGATGTTCTGTACGCTGAATTCCCCTCTGGCAGTTCCTGCGGTTGACCCGACCGATGATGTTTCAAGCTTCGGTGTGCATGCGTCGTCtgtgtcttcatccactcccttgcaGCGCGTGGTnnnnnnnnnnnnnnnnnnnnnnnnnnnnnnnnnnnnnnnnnnnnnnnnnnnnnnnnnnnnnnnnnnnnnnnngcggtttcttgcggagttcgaaggtgtgttgtataccgatgaaaatcgggacggcattgtttctgcgcgnnnnnnnnnnnnnnnnnnnnnnnnnnnnnnnnnnnnnnnnnnnnnnnnnnnnnnnnnnnnnnnnnAGTATGAAGTCTATGCGaatgacggtgcgccaacgnnnnnnnnnnnnnnnnnnnnnnnnnnnnnnnnnnnnnnnnnnnnnnCTTCTGTGTTCCCCATACCCCAGAATATTGCCGCGCACGACCATGAGCGTGCTGCTGAGCGCTGGATTGCCCCGCGCGTGTCGCTTCCTGCAGATAATGGGGTGGAGGGAGAAACTCAGGCGCGTGTTCCTTTGACGCAGCAAGGCTACAGAGTGTGTTACGATCGCTACCCTGAAGTACACCAGGTGGGGTGGGAGGATAAAACGTACGTATTGCGTCCTCGTGCACTTCGGTGGCAGCCGGTACGCATGCAGTCCttagatcttgcgcgaGATTTAGAAGGTGTGCGGTCGCACGATTTTTTnnnnnnnnnnnnnnnnnnnnnnnnnnnCCCTACTGAGCAGCAGATCACAGTTTCTTCCCTTTATTACGAaaagcCTGATTCCCTGTTTGAGCGTGCCCGCGTAAGGACGTACCTTGATGaagggctgcctttattttctgaaactcatgttggatcgCGTTTTCGTGCGCGTACGCACTAtGTGGATGGACGCGCAACGCGACGGGATAGCGATCGGGATGATGATGGATTTTTTGAAACGCGGGAATACTACAATGCTCAAGGGGCGGTACGTGCGTTGTCAGTTGACGTGCACAAGGATCGAAGTTTTGCCTATCAAGAAGAGTATGGAGCGAAGGGACAGAAGGTGCAAAAATGGTATGGCCGTGGACGTGTGACGATTTCGCACACAGAACTACCTACCGGTCATGCGCGTACTGAGTGGTTGCACCCGGTGACAGGTAGGCACGTGACAGTAGATTTTGTGCAGGGGGTGCCTAAAAGGCTGCTAGTGGATGGGGAGGTGCACGCGCTTACCAAAGACCCGCGTAACGCTGCGTTATATTGGGTGCGGCGGATCCCGCGGAACGGGGACGAAGTGGGACAAAGAATAGTTGAATCTTTCCGCGCGGCGACCTCACCGGTGGTGTCCGAGTATTTCAGGACGGGTGGGAGCGTGGTGCGAGCGGTCCGCTCCGGAGGAGTTGTCTTCGCCGAGGAGcttgaacnnnnnnnnnnnnnnnnnnnnnnnnnnnnnnnnnnnnnnnnnnnnnnnnnnnnnnnnnnnnnnnnnnnnnnnnnnnnnnnnnnnnnnGGTGTACTTTGAAATGCCGAAGTAGTGGGAGACCCTGTCTTCCGCGCGTGTAATGAGAAACGCCCCTATCTGGCTGAGAAAGTGGATGGCATCCGTTnnnnnnnnnnnnCTCATGAGcgctgcaggttttcctactctttctacagactcttctatcaggtcgtcgaggaacGCACTGACGCTCTTAAGGT
[truncated: 737,818 more chars]
